# A Database of microRNA Expression Patterns in *Xenopus laevis*

**DOI:** 10.1371/journal.pone.0138313

**Published:** 2015-10-27

**Authors:** Ayisha Ahmed, Nicole J. Ward, Simon Moxon, Sara Lopez-Gomollon, Camille Viaut, Matthew L. Tomlinson, Ilya Patrushev, Michael J. Gilchrist, Tamas Dalmay, Dario Dotlic, Andrea E. Münsterberg, Grant N. Wheeler

**Affiliations:** 1 School of Biological Sciences, University of East Anglia, Norwich Research Park, Norwich, NR4 7TJ, United Kingdom; 2 The Genome Analysis Centre (TGAC), Norwich Research Park, Norwich, NR4 7UH, United Kingdom; 3 The Francis Crick Institute, Mill Hill Laboratory, The Ridgeway, Mill Hill, London, NW7 1AA, United Kingdom; The Rockefeller University, UNITED STATES

## Abstract

MicroRNAs (miRNAs) are short, non-coding RNAs around 22 nucleotides long. They inhibit gene expression either by translational repression or by causing the degradation of the mRNAs they bind to. Many are highly conserved amongst diverse organisms and have restricted spatio-temporal expression patterns during embryonic development where they are thought to be involved in generating accuracy of developmental timing and in supporting cell fate decisions and tissue identity. We determined the expression patterns of 180 miRNAs in *Xenopus laevis* embryos using LNA oligonucleotides. In addition we carried out small RNA-seq on different stages of early Xenopus development, identified 44 miRNAs belonging to 29 new families and characterized the expression of 5 of these. Our analyses identified miRNA expression in many organs of the developing embryo. In particular a large number were expressed in neural tissue and in the somites. Surprisingly none of the miRNAs we have looked at show expression in the heart. Our results have been made freely available as a resource in both XenMARK and Xenbase.

## Introduction

MicroRNAs (miRNAs) were first discovered in genetic screens in *Caenorhabditis elegans*. Later it was shown that short RNAs with the size of 20–25 nucleotides are involved in posttranscriptional gene silencing in plants and in RNA interference in animals. In recent years they have been shown to play a major role in many developmental processes such as early patterning and muscle development [1–3].

Transcription of miRNA genes by RNA polymerase II produces longer primary miRNA transcripts [4]. In animals, an RNaseIII type enzyme—Drosha—cleaves the primary transcripts in the nucleus and releases a shorter (60–80 nt) precursor molecule [5]. These precursors have a characteristic stem-loop structure and they are translocated to the cytoplasm where Dicer cleaves the loop region of the precursor and liberates a ~22bp long miRNA duplex [6–9]. One or both strands of the duplex are incorporated into a protein complex called RISC (RNA Induced Silencing Complex).

miRNAs display partial sequence complementarity to their targets which allows RISC to be guided to the target site on the mRNA. Animal miRNAs usually show limited binding to their target mRNA typically occuring in the 5’ portion of the miRNA between positions 2–7 known as the miRNA ‘seed’ sequence [10]. This interaction usually leads to translational inhibition, deadenylation and subsequent RNA degradation [11]. Since miRNA targeting is reliant on only a short 7mer seed match it makes predicting miRNA targets extremely challenging. However, for many tissue specific miRNAs important targets have been characterized, see for example [3, 12, 13]. Hundreds of miRNAs have been identified and deposited into the miRNA registry miRBase [14] yet limited data is available on their expression patterns during key developmental stages. This is important information to consider when attempting to determine miRNA function in development and disease.

### Expression analysis using whole mount in situ hybridisation (WISH)

In order to begin to understand the function of miRNAs during embryogenesis, it is important to characterize their spatiotemporal expression pattern throughout development. WISH is a powerful method to determine such expression profiles and has been used extensively over the last 25 years [15].

Technically it is difficult to apply WISH to miRNAs owing to their small size. Initially probes against the longer, but short-lived, primary transcripts had to be used [16]. The alternative, Northern blot analysis, only provided limited spatial and temporal resolution. Technology developed by Exiqon, using modified oligos containing miRCURY^™^ LNA (locked nucleic acid) nucleotides as probes (http://www.exiqon.com/), has enabled the clear identification of specific expression patterns. LNA probes bind more strongly to complementary sequences and provide the required high specificity for detection of mature microRNAs by WISH [17–19].

The expression patterns of miRNAs in zebrafish and chicken have been relatively well documented [17, 18]. In Xenopus the expression of some miRNAs has been shown however, the data is incomplete. We presented the first WISH of a Xenopus miRNA, miR-206 in a paper showing its expression in the somites of chick, mouse and Xenopus [20]. Around the same time the expression profile of 24 miRNAs was shown by Northern analysis [21]. miRNAs in Xenopus have since been shown to be necessary for controlling Nodal signalling and induction of Spemann’s organiser [22] as well as playing roles in kidney development [23]. The expression patterns of 18 *Xenopus tropicalis* miRNAs have been published [24] using probes used against the longer primary transcripts. In the study the authors also tried to detect 42 other miRNAs and obtained negative results. This could be due to a lack of expression or a number of technical reasons. These include very rapid processing of the primary transcript, low levels of expression, expression at a different stage of development, or a non-optimised hybridisation probe or procedure. The use of LNA probes overcomes some of these problems.

In this paper we present the expression patterns of 180 miRNAs in Xenopus laevis. These have been made freely available and can be accessed using the XenMARK and Xenbase websites [25, 26]. In addition we have carried out small RNA sequencing of Xenopus laevis embryonic tissue. This identified 44 miRNA precursors belonging to 29 new miRNA families. We present expression for 5 of these.

## Materials and Methods

### Wholemount in situ hybridization

All experiments were performed in compliance with the relevant laws and institutional guidelines at the University of East Anglia. The research has been approved by the ethics committee of the University of East Anglia. *Xenopus laevis* embryos were obtained as previously described [27]. Embryos were staged using the Nieuwkoop and Faber normal table of *Xenopus* development [28]. Wholemount in situ Hybridisation using LNA probes was carried out as described [20]. LNA probes were provided by Exiqon. Each probe was preadsorbed against Xenopus and chick embryos at least 6 times before carrying out a full WISH.

### Cryosectioning

Embryos were fixed in MEMFA for 1 hour, washed twice for 5 minutes in PBST and then put in 30% sucrose for 4 hours at room temperature. Embryos were then transferred to cryomoulds filled with optimal cutting temperature (OCT) compound and left at 4°C overnight. Embryos were then positioned appropriately for sectioning and left at -20°C overnight. Embryos were sectioned using the LEICA CM 1950 Cryostat and sections were placed on 5% TESPA slides. Slides were then washed for 5 minutes in PBST and covered with a coverslip using hydromount. Images were taken using a Zeiss CCD upright microscope with colour camera.

### RNA extraction and miRNA Sequencing

To characterise the population of miRNA during *Xenopus* embryo development, different developmental stages were considered: unfertilised eggs, and developmental embryos at stages 7, 11–12, 18–20, 34–36 and 56. Total RNA was isolated using TRIzol^®^ (15596–026, Life technologies, UK) according to manufacturer´s recommendations and DNAse treated. RNA integrity and purity were assessed in 1% (w/v) agarose gel stained with ethidium bromide and on a Nanodrop ND-1000 (Thermo Scientific, DE, USA), respectively. Ratios of Abs 260/280 and Abs 260/230 were above 2.0 for all samples.

The sRNA libraries were prepared as described before [29] using total RNA from the different developmental stages. Briefly, the RNA was ligated to the adaptors, reverse transcribed, PCR amplified and purified from a polyacrylamide gel. cDNA libraries were sequenced using an Illumina Genome Analyzer II. Sequences can be accessed on NCBI SRA under the project number PRJNA292052.

#### Analysis of Sequences

Small RNA libraries were converted from FASTQ to FASTA format and adaptors were trimmed using the UEA Small RNA Workbench [30]. New miRNAs were predicted using miRCat tool with default parameters and release 6.1 of the *Xenopus laevis* genome sequence. All small RNA sequences were mapped to the predicted pre-miRNA hairpins and aligned using a custom Perl script. Secondary structures were predicted using RNAfold [31] and converted to pdf format with 5p and 3p miRNAs highlighted. Alignments and secondary structures were checked manually to ensure that both the structures and read distribution across the hairpin was consistent with Drosha and Dicer processing. Plots showing small RNA coverage across each hairpin were generated using a custom Perl script.

## Results

At the time of starting this project there were 174 miRNAs identified in *Xenopus laevis* and *tropicalis* as shown in miRBase [14]. In collaboration with Exiqon we generated 174 LNA probes against these miRNAs. The probes were designed to work at the same hybridisation temperature of 50°C. This enabled us to perform WISH in a high throughput manner. In order to see a genuine specific expression pattern we found we needed to preabsorb each probe at least 6 times by hybridising it to Xenopus and chick embryonic tissue before carrying out a complete WISH.

In order to observe developmental expression patterns we tested each probe on embryos at pre-gastrula (stages 8–9), gastrula (stages 10.5–12), neurula (stages 15–20), tailbud (stages 30–34) and tadpole stages (stages 35–37). Each probe was tested at least 3 times and the expression patterns were documented and photographed. Surprisingly, out of the 174 miRNAs tested 140 showed expression in the developing embryo at some stage. Selected photos showing expression of specific miRNAs can be freely accessed using XenMARK [26] and Xenbase [25, 32]. In Xenbase each miRNA is listed by name (ie miR-206) and clearly states at which stages of development expression is seen. In XenMARK the miRNAs can be searched for according to where and when they are expressed in the embryo and also by name.

**Fig 1:**
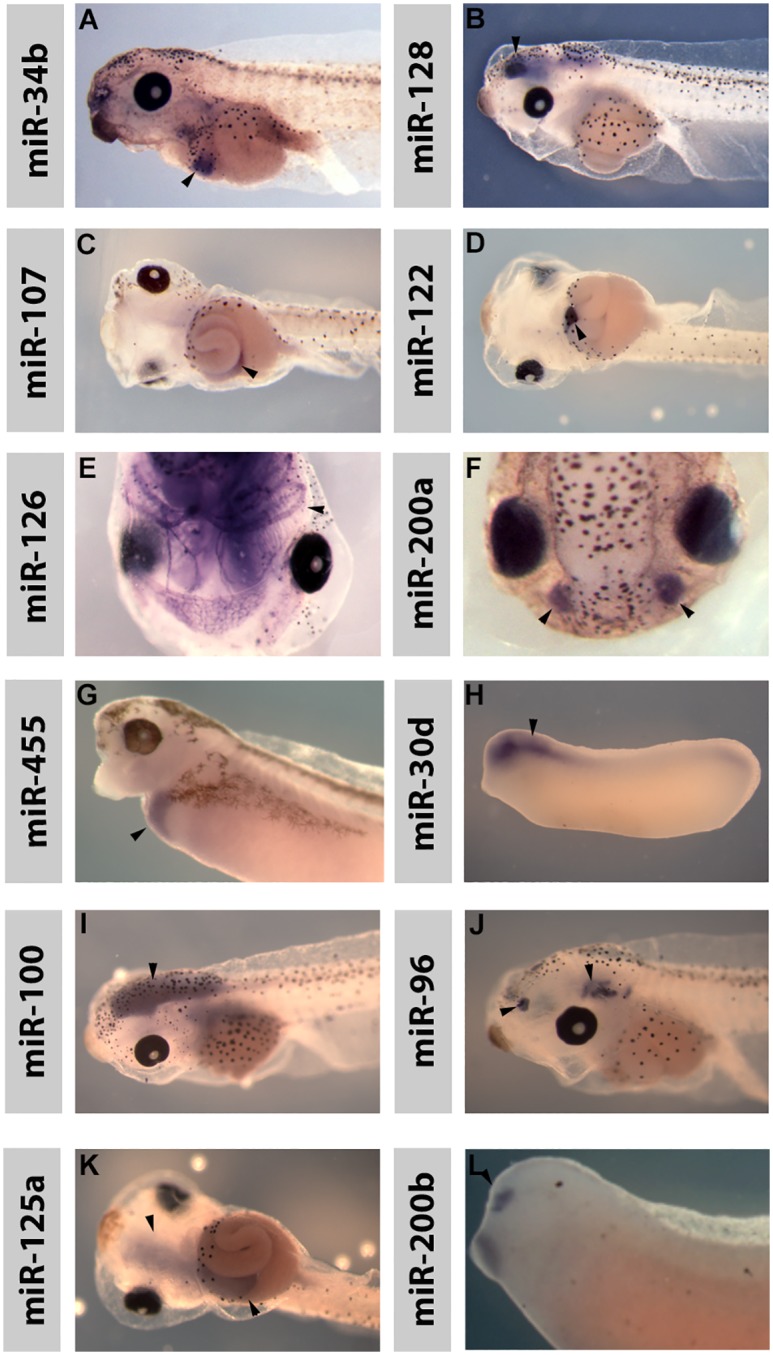
*Xenopus* MicroRNAs show expression in a diverse number of developing organs and cell types. Expression patterns of *Xenopus* laevis miRNAs are shown at varying stages. Arrowheads point to relevant expression. Views are lateral with anterior to left except (C, D and K) which is a ventral view with anterior to the left and (E) and (F) which are dorsal views with anterior to the bottom. MiR-34b, pancreas; miR-128, brain; miR-107, gut; miR-122, liver; miR-126, blood vessels; miR-200a, olfactory placodes; miR-455, liver; miR-30d, brain; miR-100, brain; miR-96, brain and olfactory placodes, miR-125, brain and gut, miR-200b, olfactory placode.

Analysis of the expression patterns showed that many of the miRNAs were expressed during development and many of them were also restricted in their expression. Fig 1A–1L shows examples of some of the miRNAs with very specific expression patterns. S1 Table shows the sequence of each miRNA plus a general description of the expression seen.

**Fig 2:**
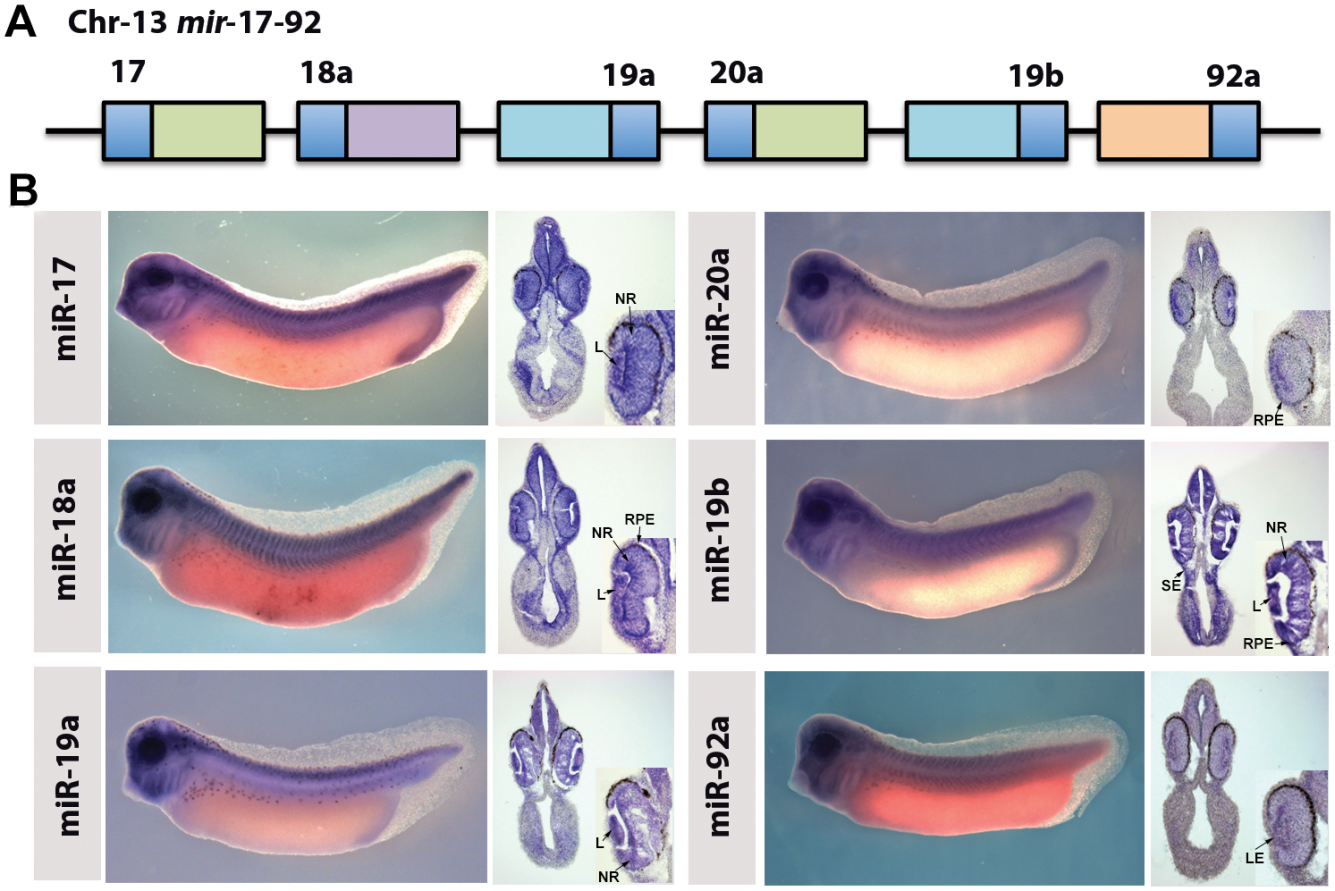
MicroRNA cluster 17–92 shows expression in different cell types of the developing eye. (A) the miR-17-92 cluster. (B) WISH of stage 33 embryos showing the wholemount embryo and 30uM sections in the region of the eye of the miRNAs from the mir-17-92 cluster. Inset panels show a close up of the eye. L, Lens; NR, Neural Retina; RPE, Retinal Pigmented epithelium; LE, Lens Epithelia; SE, Surface Ectoderm.

A number of miRNAs were expressed in the eye. The mir-17-92 cluster has been well characterised in other species. Fig 2 shows the expression of miR-17-5p, 18a, 19a, 20a, 19b and 92a. As well as expression elsewhere in the embryo, all members of this cluster show expression in the eye, which was confirmed by sectioning (Fig 2A and 2B). Other miRNAs that show expression in the eye are listed in S1 Table.

**Fig 3:**
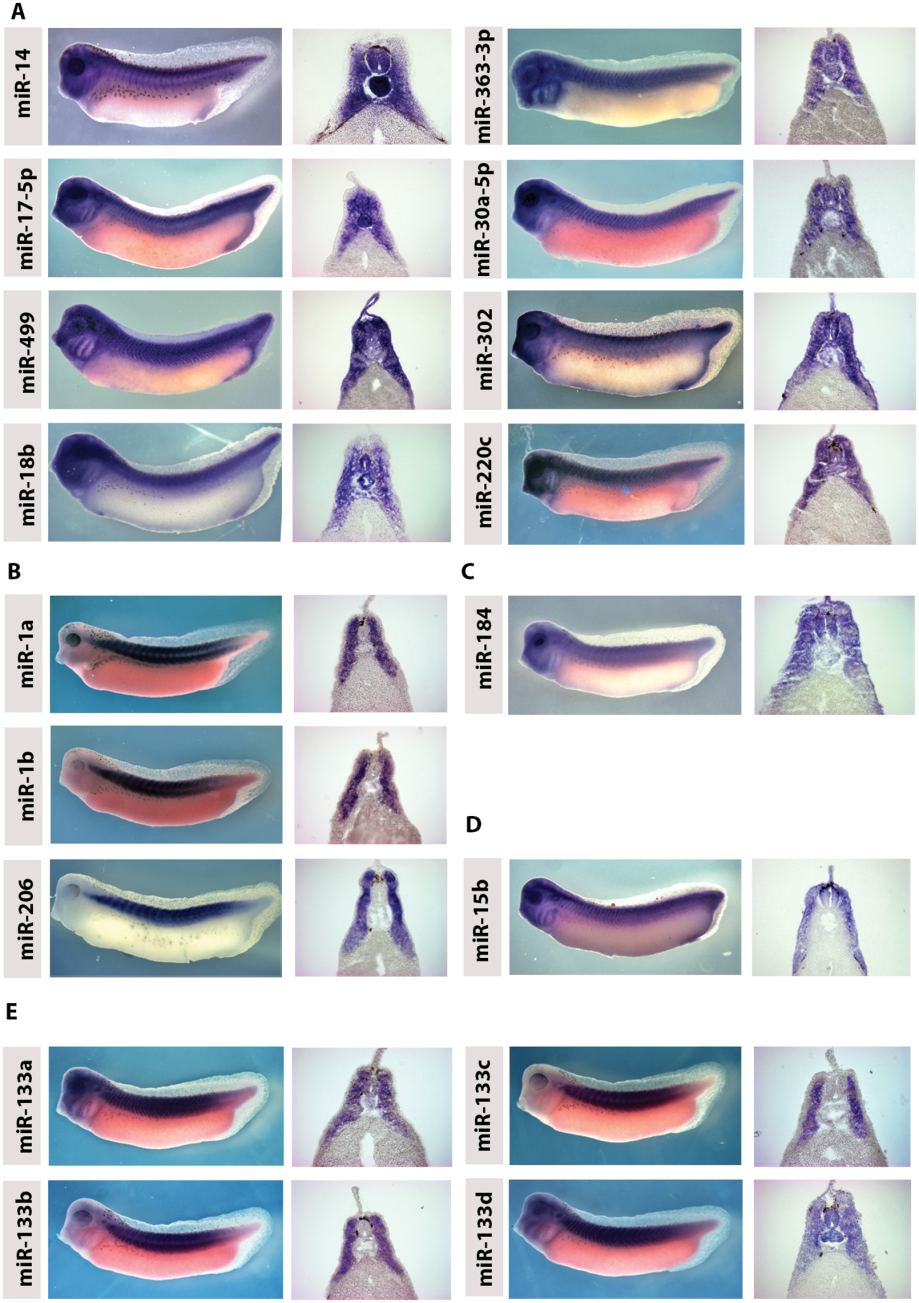
Expression of *Xenopus* MicroRNAs in the developing somites. Expression patterns of *Xenopus* laevis miRNA are shown at stage 33/34 both in wholemounts and transverse sections from the trunk. All embryos are lateral views with anterior to the left. (A) miRs-14, 17-5p, 499, 18b, 363-3p, 30a-5p, 302 and 220c show expression in the head and trunk. Sections show expression in the somites, neural tube and notochord. miR-499 and 302 also shows expression in the surface ectoderm. (B) miRs 1a, 1b and 206 show expression only in the trunk which is confined to the somites in sections. (C) miR-184 shows expression in the head and trunk. sections show expression in the nural tube and somites but bot the notochord. (D) mir-15b shows expression in the head and trunk. Sections show expression in the somites and dorsal neural tube. (E) mir133 family shows differential expression. Mir-133a and d show expression in the head and trunk and in the somites, neural tube and notochord. miR-133b and c show expression in the trunk and sections show only expression in the somites.

As expected many miRNAs were expressed in neural tissues. Remarkably, however, many microRNAs were detected in developing somites. S2 Table lists all miRNAs, which show expression in somites along with other tissues they are expressed in. We show examples of miRNAs expressed in the neural tube, somite and notochord (Fig 3A), or expressed in somites only (Fig 3B). Fig 3C shows expression for miR-184 in the somite and neural tube but not the notochord. Fig 3D shows miR-15b expression in the more dorsal neural tube and somite and again not in the notochord. Fig 3E shows the expression pattern of the miR-133 family. miR-133a and 133d are expressed in the somites, neural tube and notochord. miR-133b and c show expression in the somite only. To examine whether the large number of miRNAs we found expressed in somites is conserved, we compared the expression of these miRNAs reported in chick. We found that 23 miRNAs expressed in somites in Xenopus were also expressed in chick somites (see S2 Table and GEISHA database). In addition, we carried out in situs using LNA probes on chick embryos. S2 Table indicates which miRNAs are also expressed in chick somites. S1 Fig shows WISH and sections of chick embryos probed for these miRNAs. Interestingly none of the Xenopus somitic miRNAs showed any expression in the heart. In particular miR-1 and miR-133 were not detected in the Xenopus heart whereas their heart expression has been well characterised in both chick and mouse [13, 33]

To identify novel miRNAs we carried out small RNA sequencing of different stage embryos. We collected RNA from unfertilised eggs, Stage 7–8, stage 12, Stage 18–20, Stage 34–36 and stage 56. Small RNA libraries were generated and high-throughput sequencing carried out. S2 Fig shows the normalised temporal expression of over 300 known miRNAs identified in the *Xenopus laevis* genome. The results show at which stages these miRNAs are expressed and which strand of the hairpin loop is preferentially expressed.

**Fig 4:**
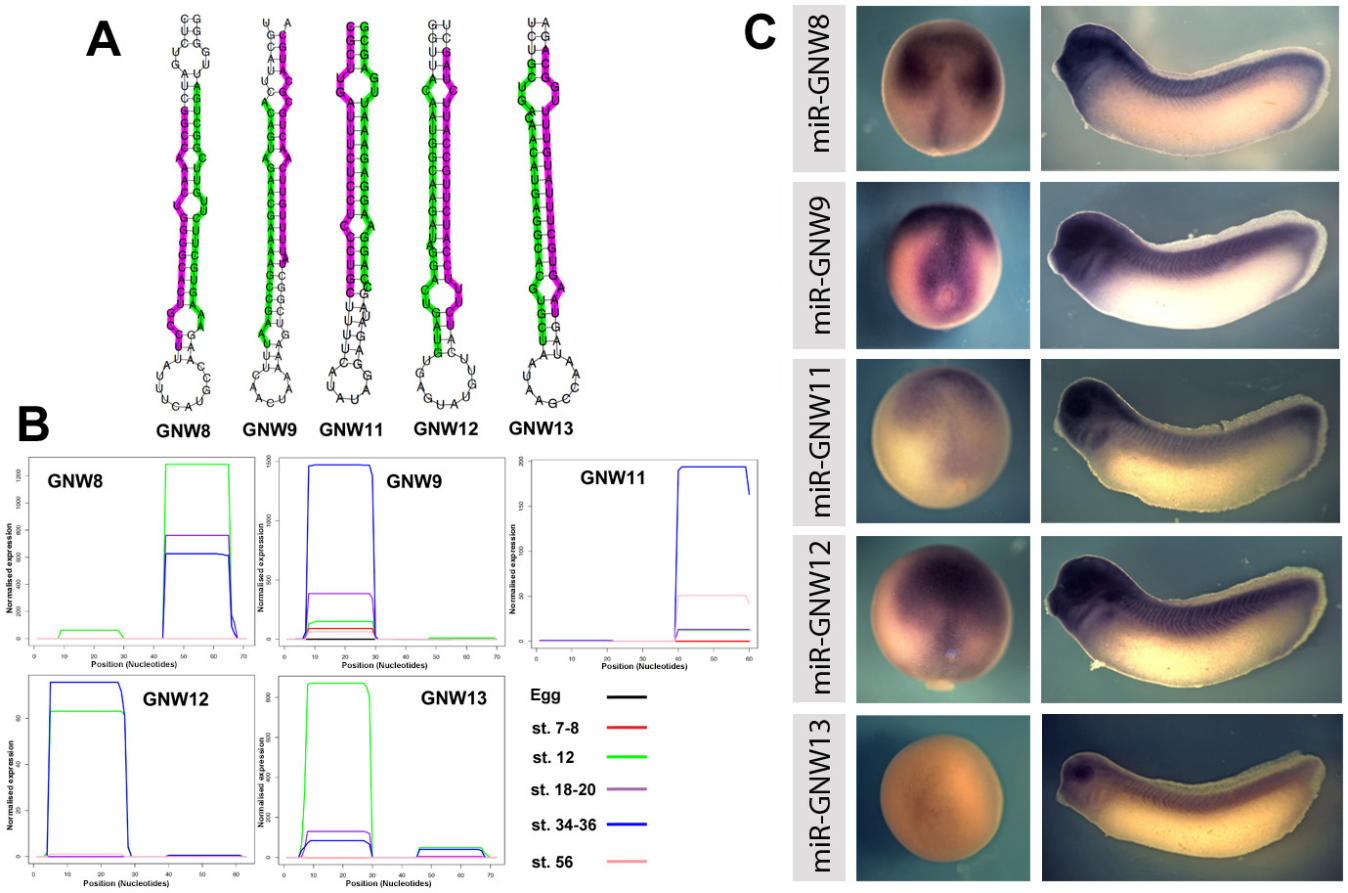
Identification and expression of novel *Xenopus* MicroRNAs. (A) Predicted pre-miRNA structures of *Xenopus* laevis mir-GNW8, 9, 11, 12 and 13. The 5p and 3p miRNAs are highlighted on the predicted hairpin precursor with the most abundant sequence highlighted in green and the lower abundance sequence in pink (B) Normalised coverage plots showing the relative expression by number of reads of the 3p and 5p miRNAs from the libraries made from unfertilised egg, stage 7–8, stage 12, stages 18–20, stages 34–36 and stage 56 *Xenopus* laevis embryos. (C) Expression patterns of the novel miRNAs at stage 12/13 and stage 28/29.

In different embryonic stages we identified 29 novel miRNAs by sequence these mapped to 44 genomic loci. Their sequences and other details are shown in S3 Table. For 5 of the more highly expressed miRNAs we show their 3p and 5p sequence along with the hairpin loops (Fig 4A). We also show which of the strands is preferentially expressed as determined by the normalised read sequence (Fig 4B). We next looked at their expression patterns in developing *Xenopus* embryos (Fig 4C). Many of these novel microRNAs show expression in late gastrula embryos in more dorsal regions including the mesoderm and neuroectodem. At later tailbud stages expression was seen in the head, branchial arches and trunk. Alignments of the other novel small RNAs identified, predicted hairpins and the small RNA coverage plots showing expression in the different stages are shown in S3 Fig.

## Discussion

miRNAs have important functions in fine-tuning many biological processes including the regulation of embryonic development [1]. Increasing numbers are being identified using ever more sophisticated sequencing and bioinformatics techniques. However for many miRNAs there is as yet little to no functional data. An important start to determining the function of a miRNA is to know where and when it is expressed during development. We have determined the expression of 179 miRNAs in *Xenopus laevis* and found that many of these are expressed in the embryo in specific expression patterns. These results mirror results from other vertebrate species and in some cases highlight important differences. For example, miR-1 and miR-133 are not expressed in the heart in zebrafish or *Xenopus*, whereas they are in amniotes [13, 17, 33]. Furthermore, we used small RNA sequencing to look at the temporal expression of over 300 known miRNAs during *Xenopus* development in addition to identifying a number of novel miRNAs, the expression of some of which are presented here.

These experiments were carried out to provide a resource to the *Xenopus* community. We have therefore made our expression results available both on Xenbase and XenMARK and the RNAseq data available on NCBI (project no. PRJNA292052). XenMARK in particular is a useful informatics resource that allows searching for genes expressed in various regions of the embryo at different time-points in development.

An interesting observation from our results is the large numbers of miRNAs expressed both in neural tissues and also more surprisingly in the somites. It has been noted previously that a large proportion of the genome is expressed at some point in the brain and nervous system [34]. However the large number of miRNAs expressed in somites has not previously been noted and the functional significance of this remains to be determined. We have confirmed our results in *Xenopus* by comparing them with chick miRNA expression from GEISHA and our own data. It is not clear at present why so many miRNA are expressed in somites along with expression in neural tissue and the notochord.

## Supporting Information

S1 Fig Expression of miRs in chick somites. (DOCX)

S2 Fig *Xenopus* miRs normalised coverage. (PDF)

S3 Fig New miRs normalised coverage. (PDF)

S1 Table All miRNAs tested including description of expression. (XLSX)

S2 Table Full list of *Xenopus* miRNAs showing expression in the developing somites as determined by wholemount in situ hybridisation (WISH). In addition a list of chicken miRs that are expressed in somites as confirmed by WISH is included.
(XLSX)

S3 Table List of novel miRNAs identified. (XLSX)
