# Supplementary material for: A Database of microRNA Expression Patterns in Xenopus laevis
Source: PLoS One. 2015 Oct 27;10(10):e0138313. doi: 10.1371/journal.pone.0138313 (PMC4624429; doi:10.1371/journal.pone.0138313)
Supplement: S1 Fig — (DOCX) [file pone.0138313.s001.docx]

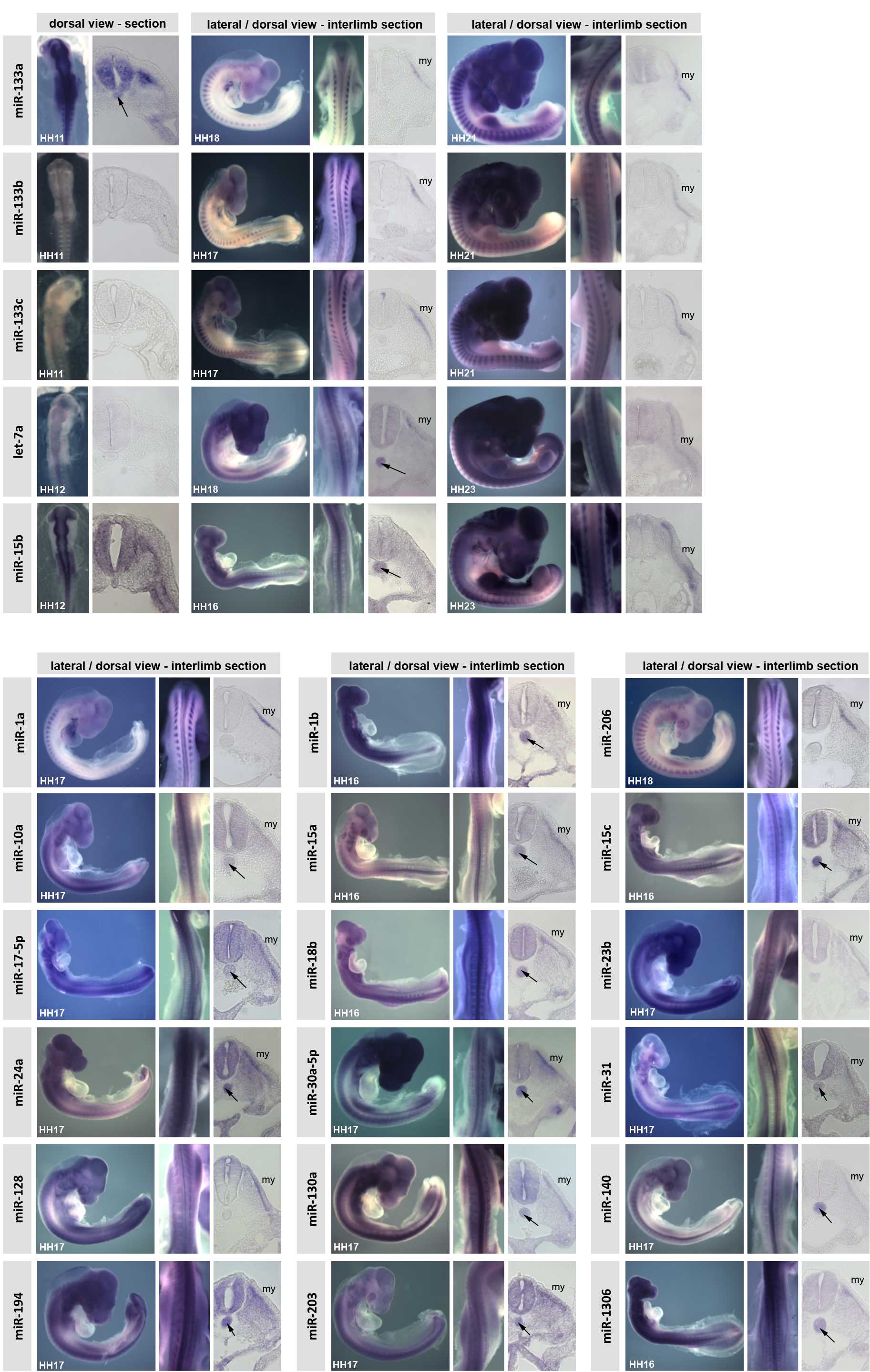
**Supplemental Figure 1**

Wholemount in situs of chick miRNAs expressed in somites. The miRNA probes used are indicated on the left of each panel. Lateral and dorsal views of whole mount embryos are shown alongside transverse sections.
