# Supplementary material for: A Database of microRNA Expression Patterns in Xenopus laevis
Source: PLoS One. 2015 Oct 27;10(10):e0138313. doi: 10.1371/journal.pone.0138313 (PMC4624429; doi:10.1371/journal.pone.0138313)
Supplement: S2 Fig — (PDF) [file pone.0138313.s002.pdf]

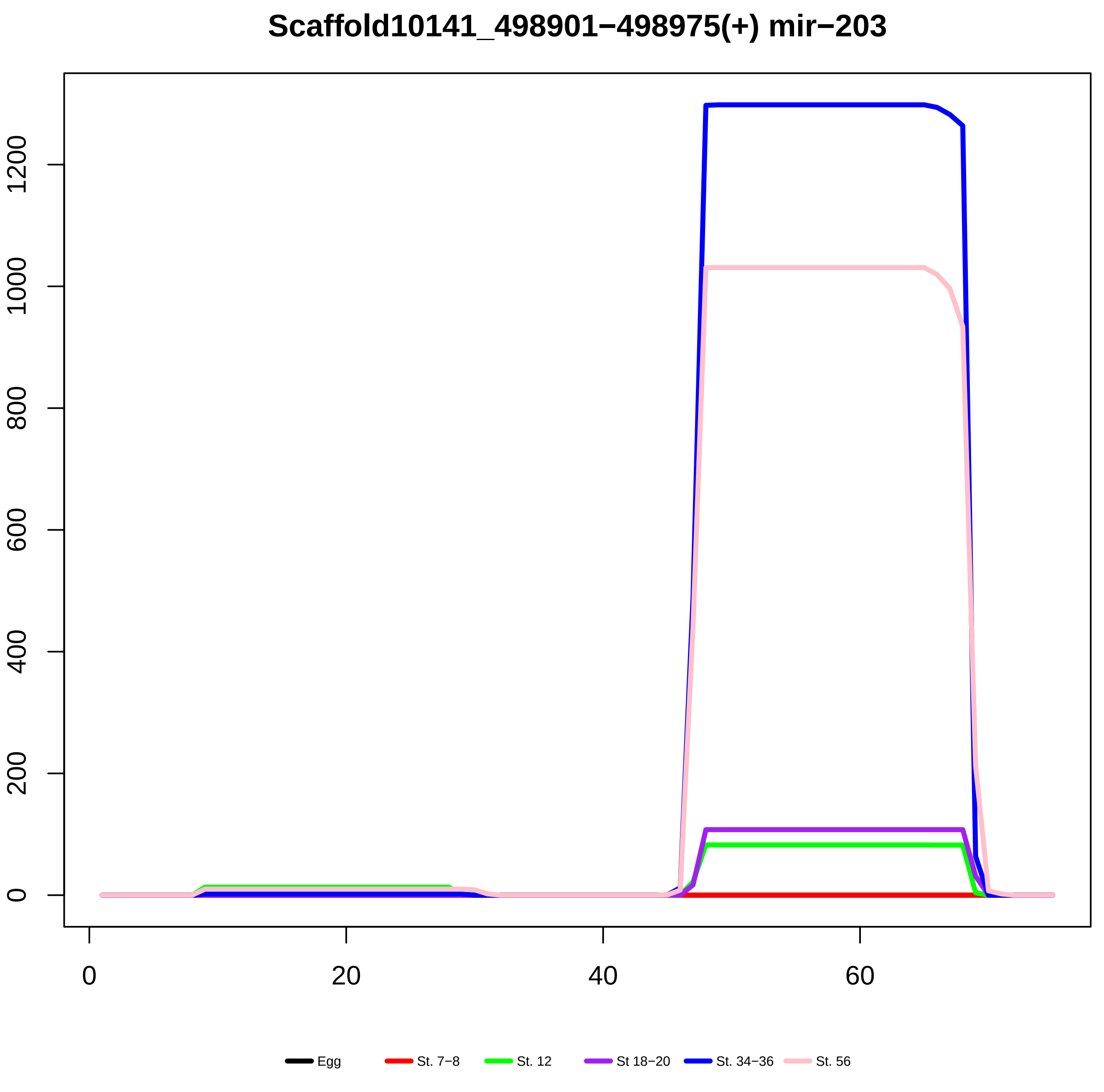
Scaffold10141_498901−498975(+) mir−203
0
200
400
600
800
1000
1200
0
20
40
60
Egg
St. 7−8
St. 12
St 18−20
St. 34−36
St. 56

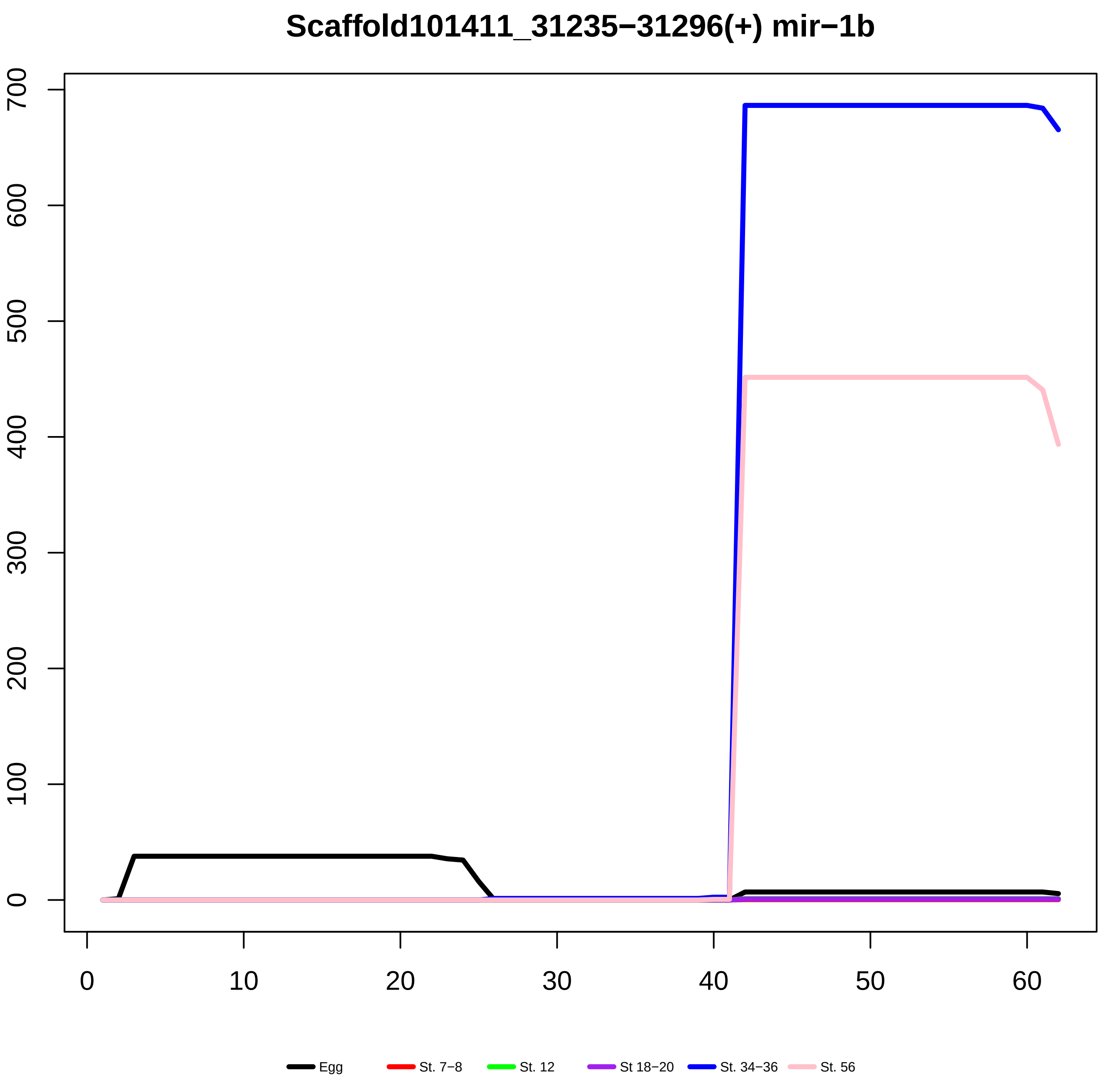

Scaffold101411_31235−31296(+) mir−1b
0
100
200
300
400
500
600
700
0
10
20
30
40
50
60
Egg
St. 7−8
St. 12
St 18−20
St. 34−36
St. 56

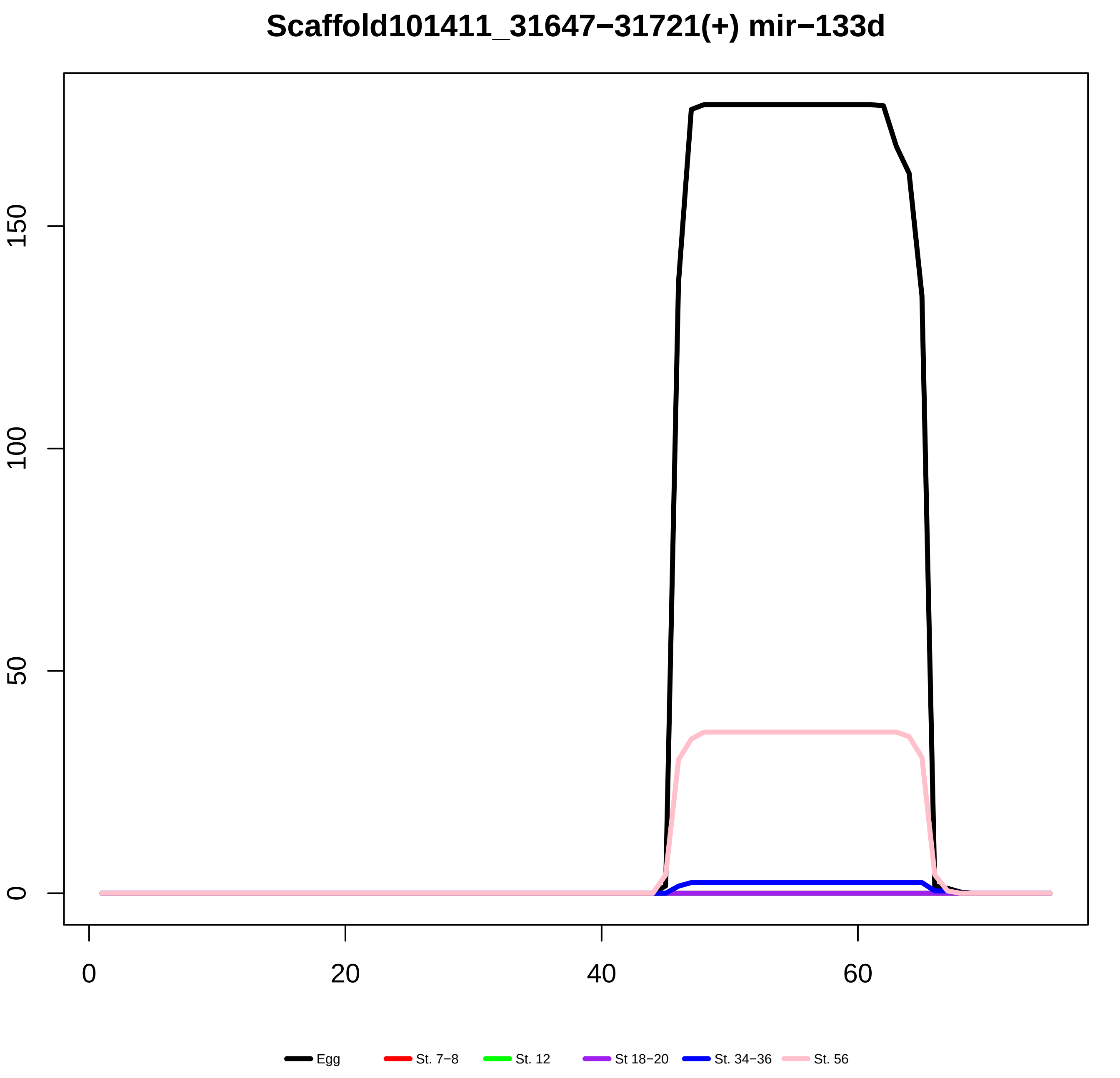
Scaffold101411_31647−31721(+) mir−133d
0
50
100
150
0
20
40
60
Egg
St. 7−8
St. 12
St 18−20
St. 34−36
St. 56

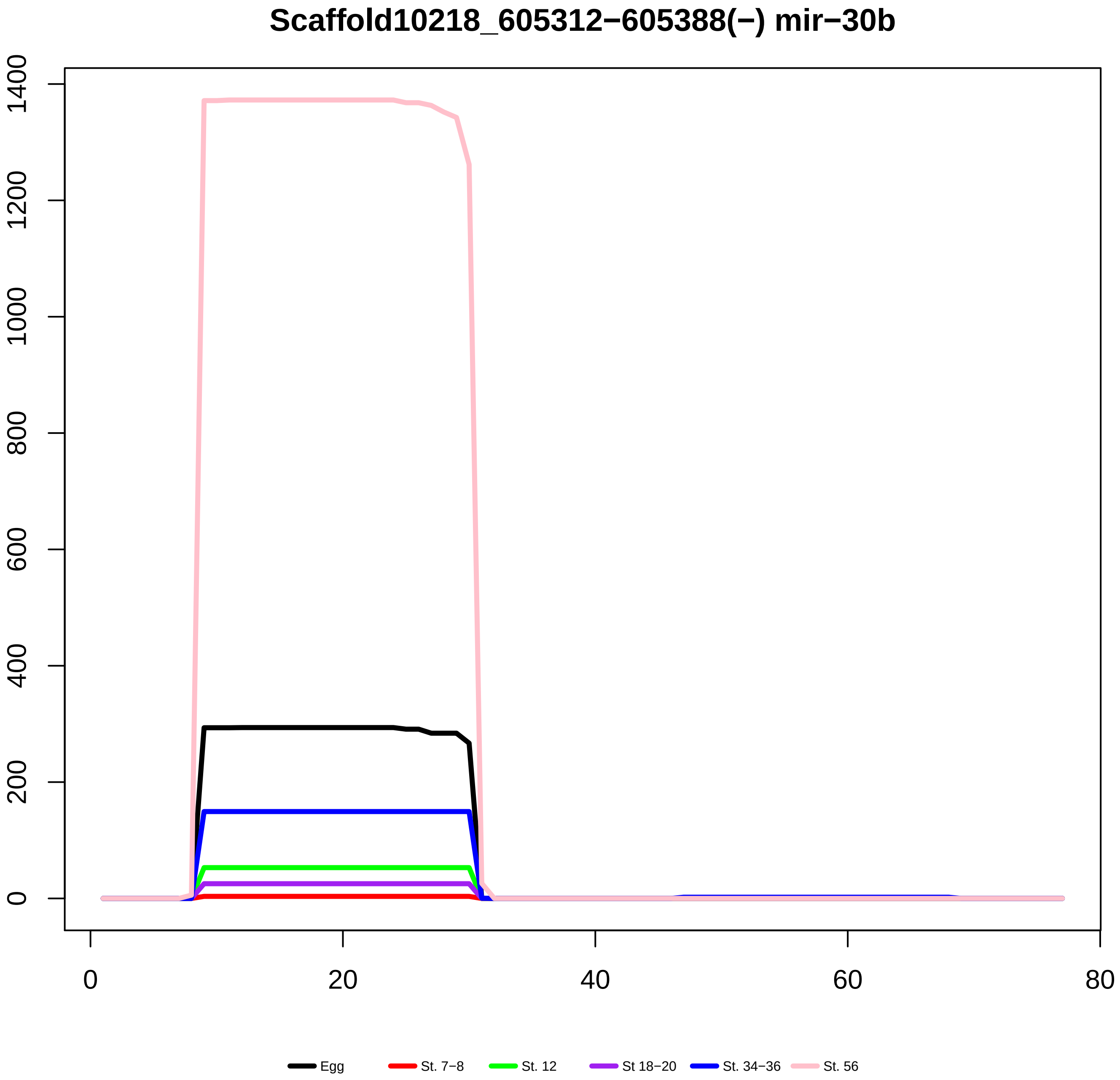

Scaffold10218_605312−605388(−) mir−30b
0
200
400
600
800
1000
1200
1400
0
20
40
60
80
Egg
St. 7−8
St. 12
St 18−20
St. 34−36
St. 56

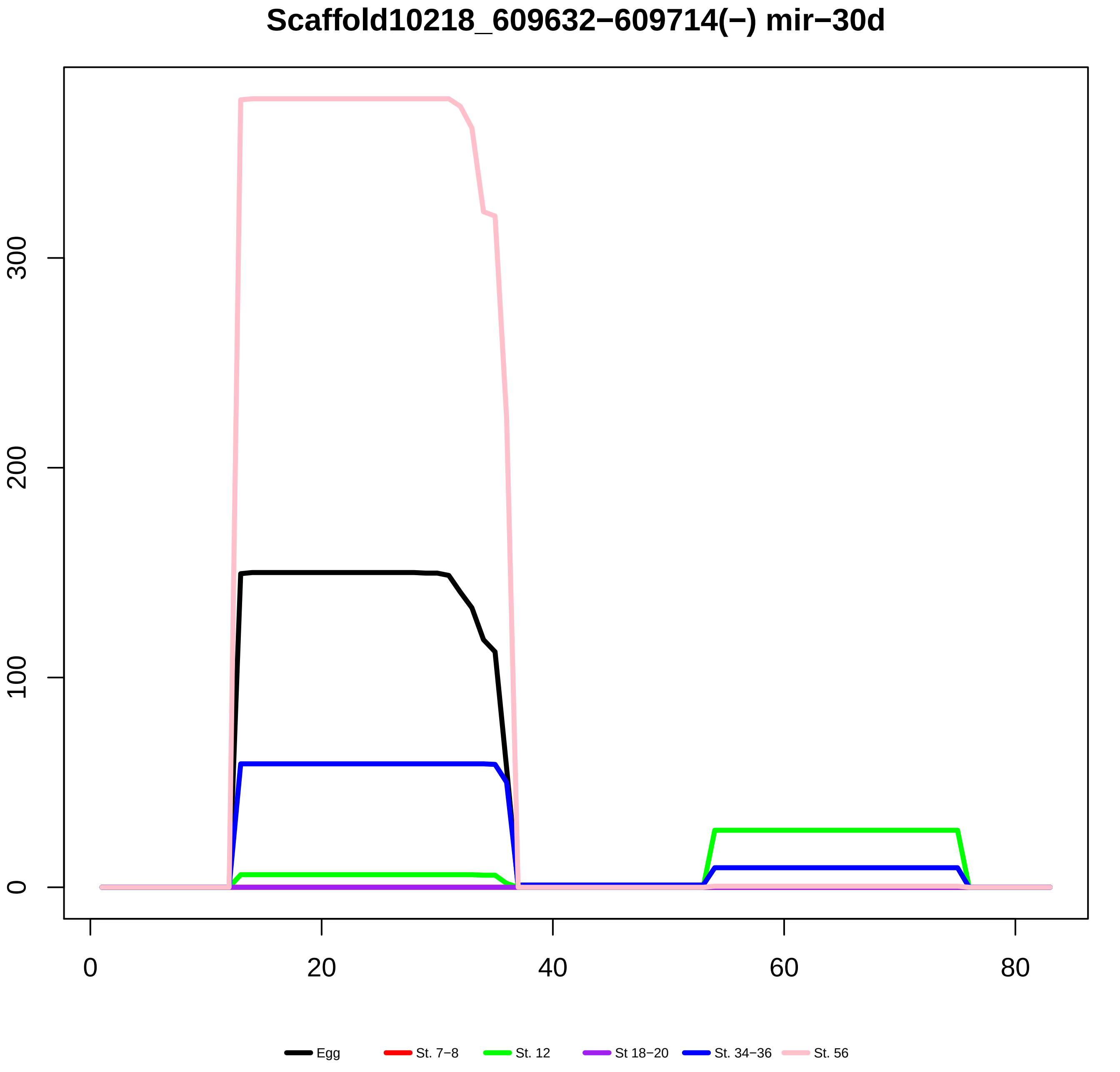
Scaffold10218_609632−609714(−) mir−30d
0
100
200
300
0
20
40
60
80
Egg
St. 7−8
St. 12
St 18−20
St. 34−36
St. 56

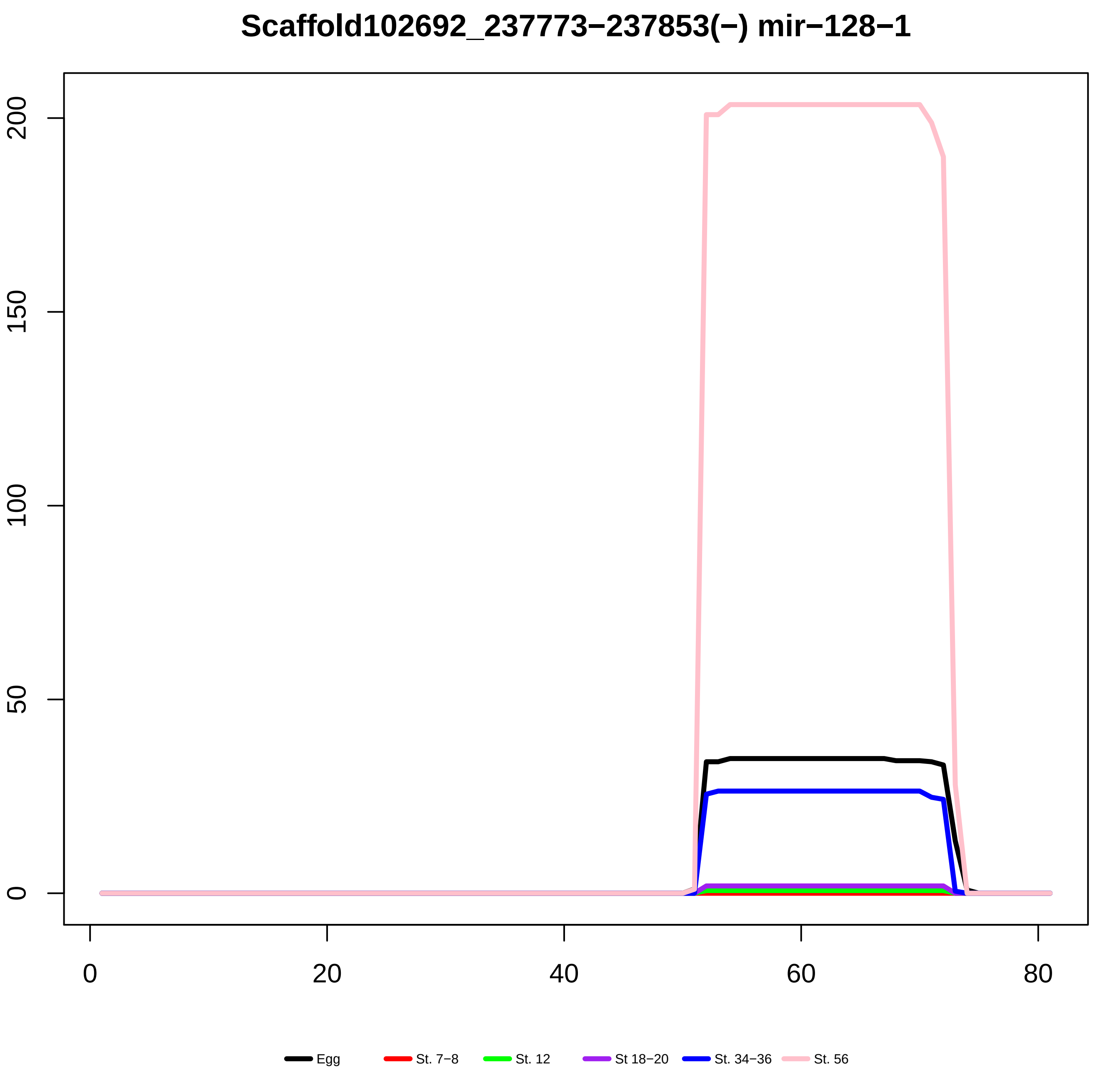

Scaffold102692_237773−237853(−) mir−128−1
200
150
100
50
0
0
20
40
60
80
Egg
St. 7−8
St. 12
St 18−20
St. 34−36
St. 56

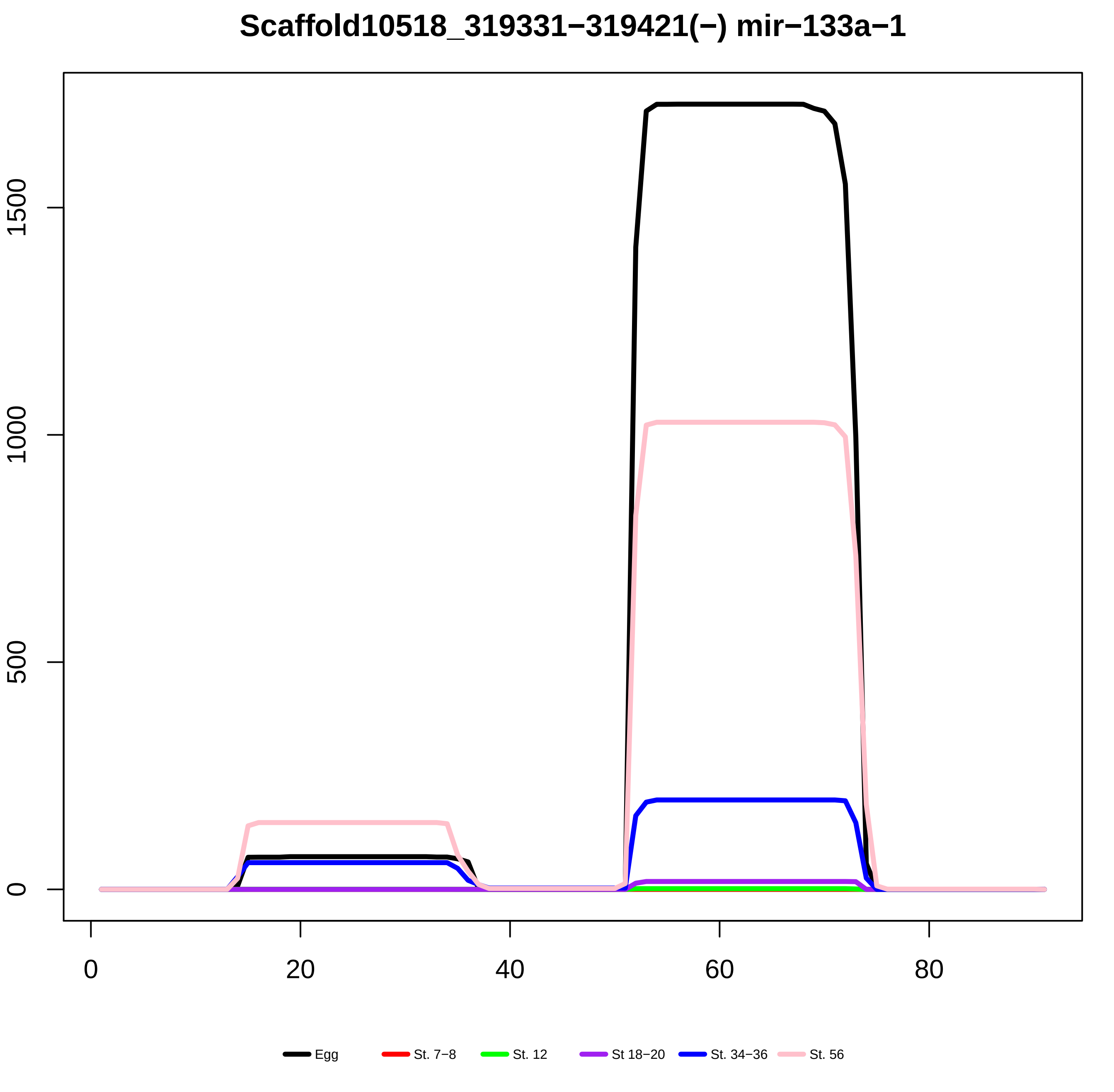
Scaffold10518_319331−319421(−) mir−133a−1
0
500
1000
1500
0
20
40
60
80
Egg
St. 7−8
St. 12
St 18−20
St. 34−36
St. 56

Scaffold10518_337718−337789(−) mir−1a−1

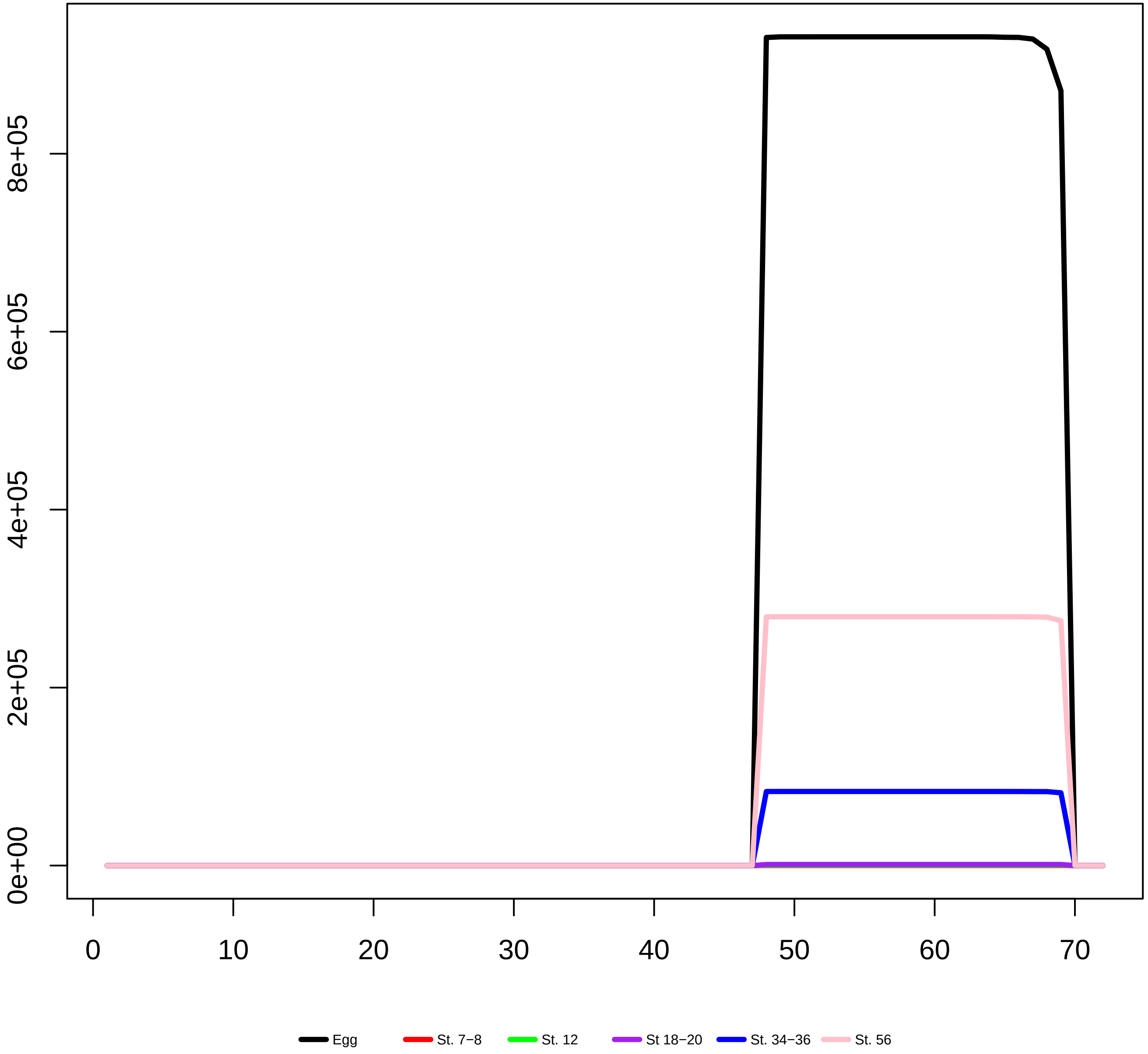

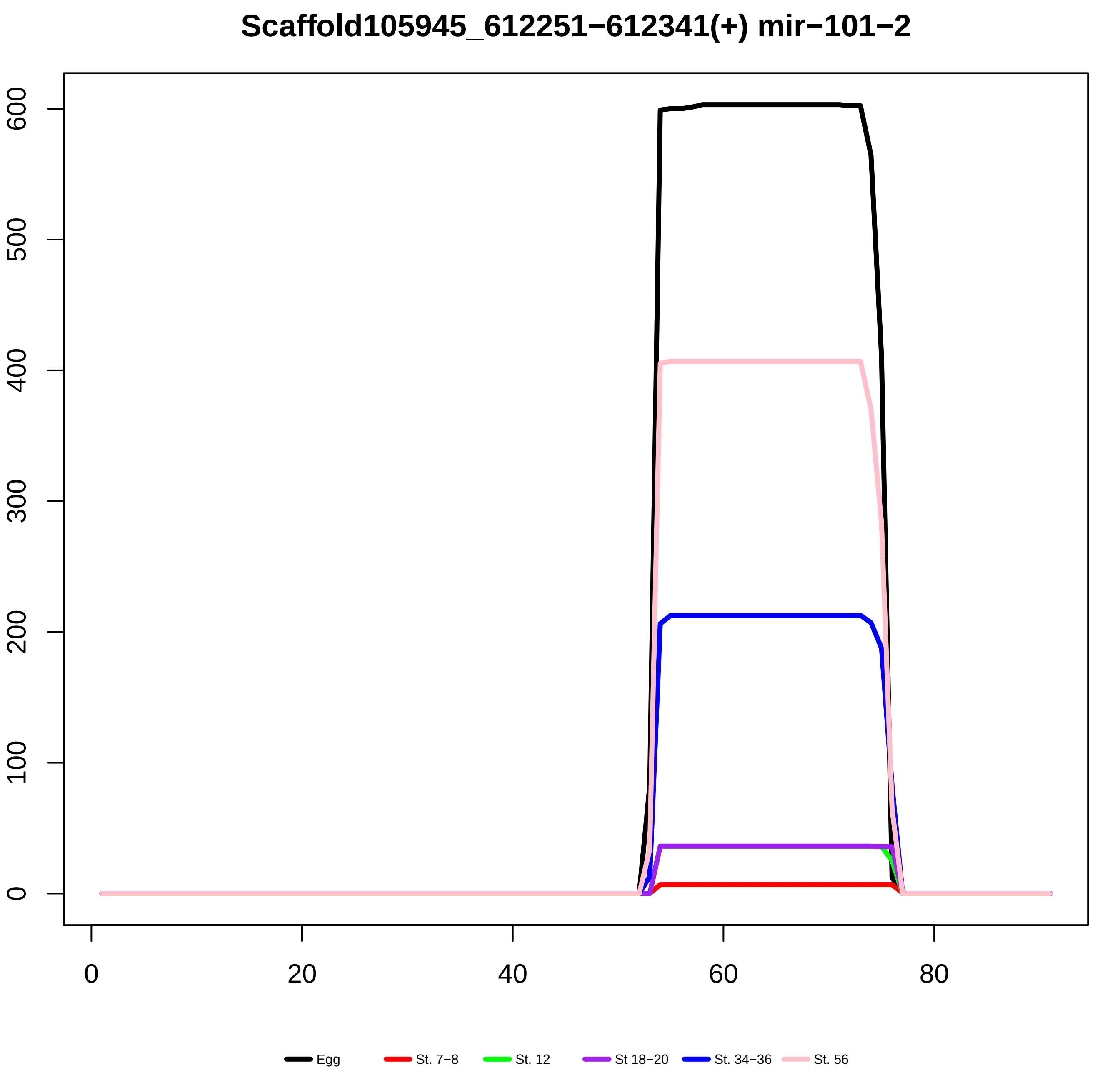

Scaffold105945_612251−612341(+) mir−101−2
0
100
200
300
400
500
600
0
20
40
60
80
Egg
St. 7−8
St. 12
St 18−20
St. 34−36
St. 56

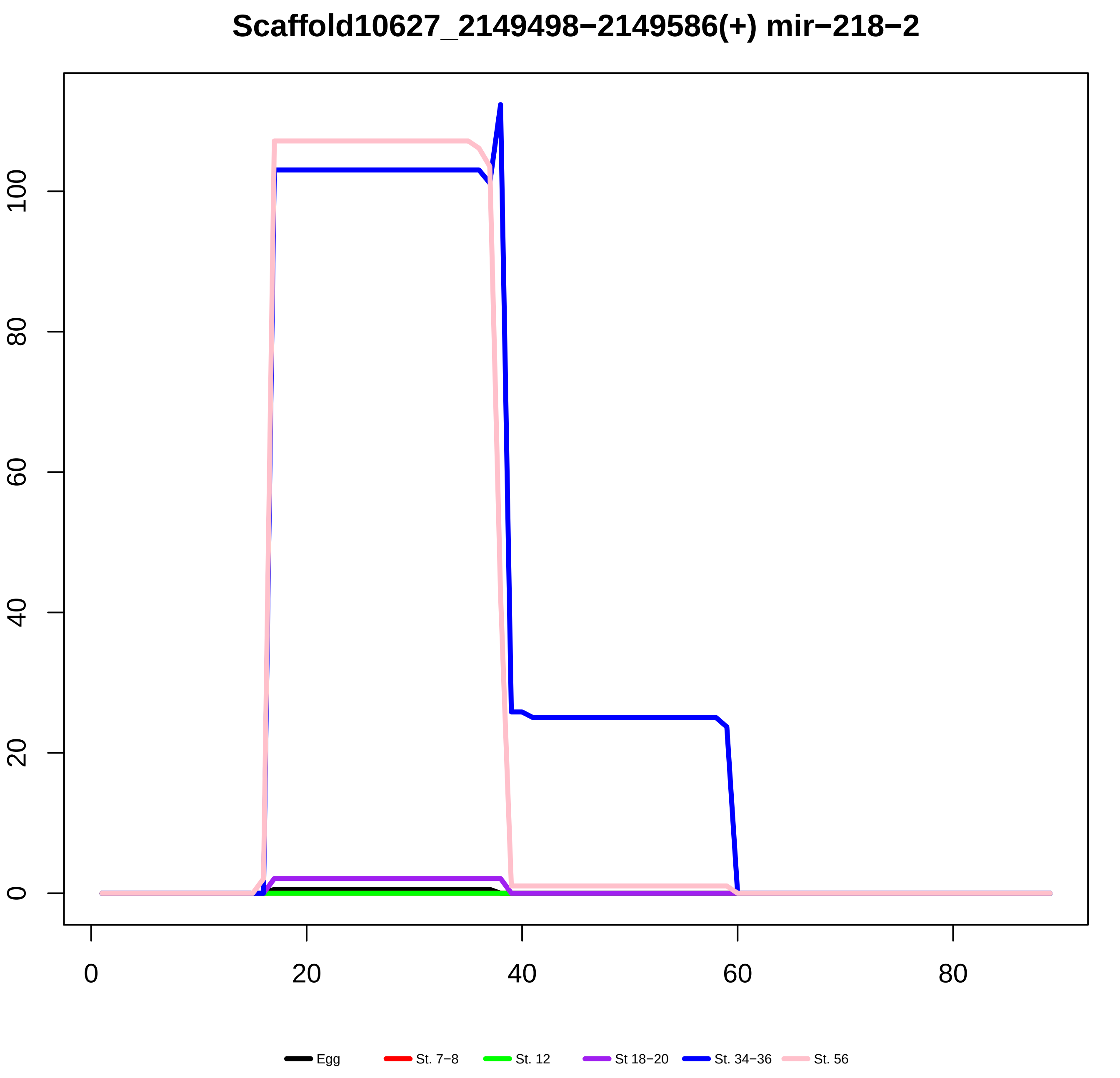
Scaffold10627_2149498−2149586(+) mir−218−2
0
20
40
60
80
100
0
20
40
60
80
Egg
St. 7−8
St. 12
St 18−20
St. 34−36
St. 56

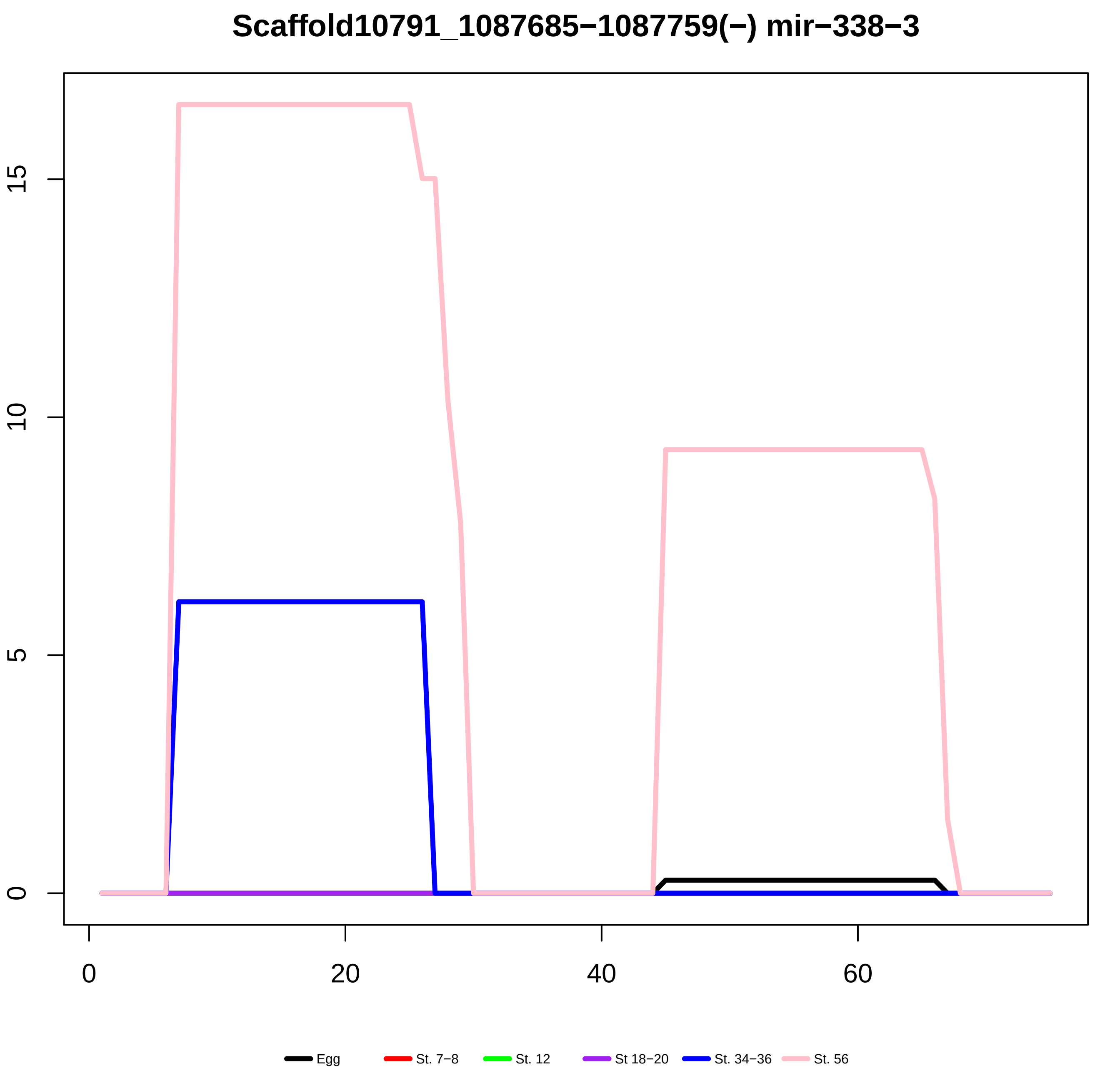
Scaffold10791_1087685−1087759(−) mir−338−3
0
5
10
15
0
20
40
60
Egg
St. 7−8
St. 12
St 18−20
St. 34−36
St. 56

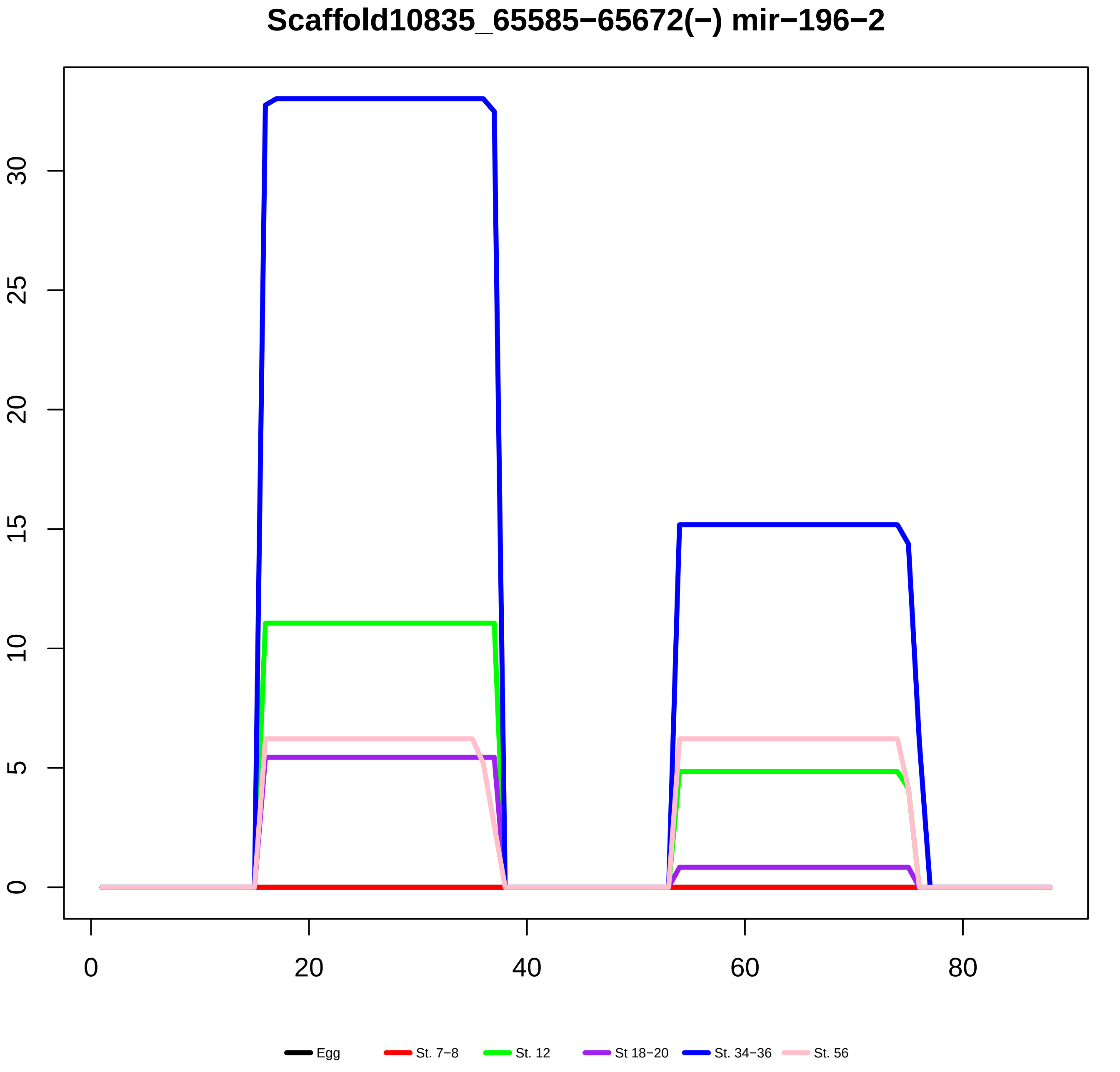
Scaffold10835_65585−65672(−) mir−196−2
0
5
10
15
20
25
30
0
20
40
60
80
Egg
St. 7−8
St. 12
St 18−20
St. 34−36
St. 56

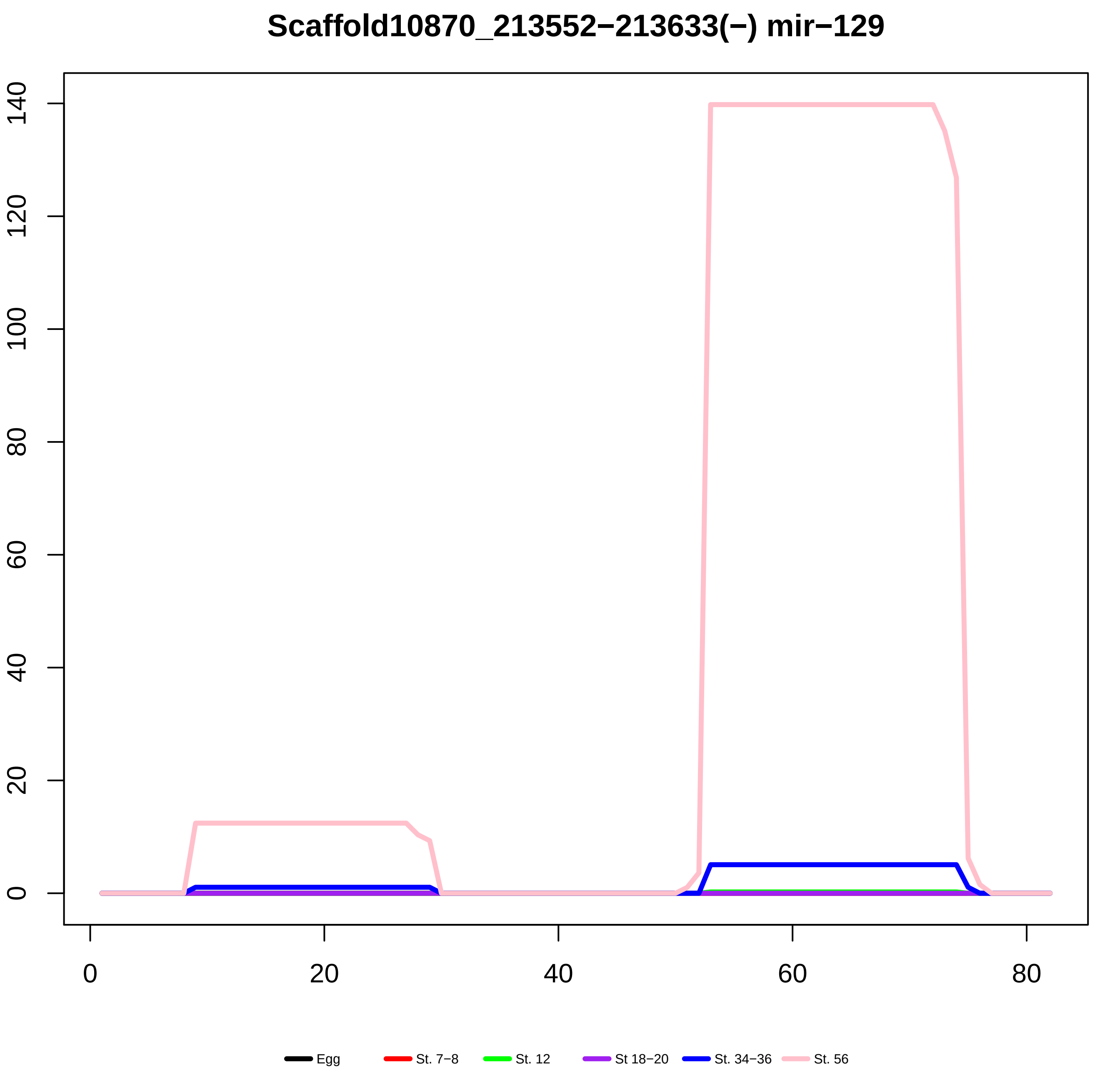
Scaffold10870_213552−213633(−) mir−129
0
20
40
60
80
100
120
140
0
20
40
60
80
Egg
St. 7−8
St. 12
St 18−20
St. 34−36
St. 56

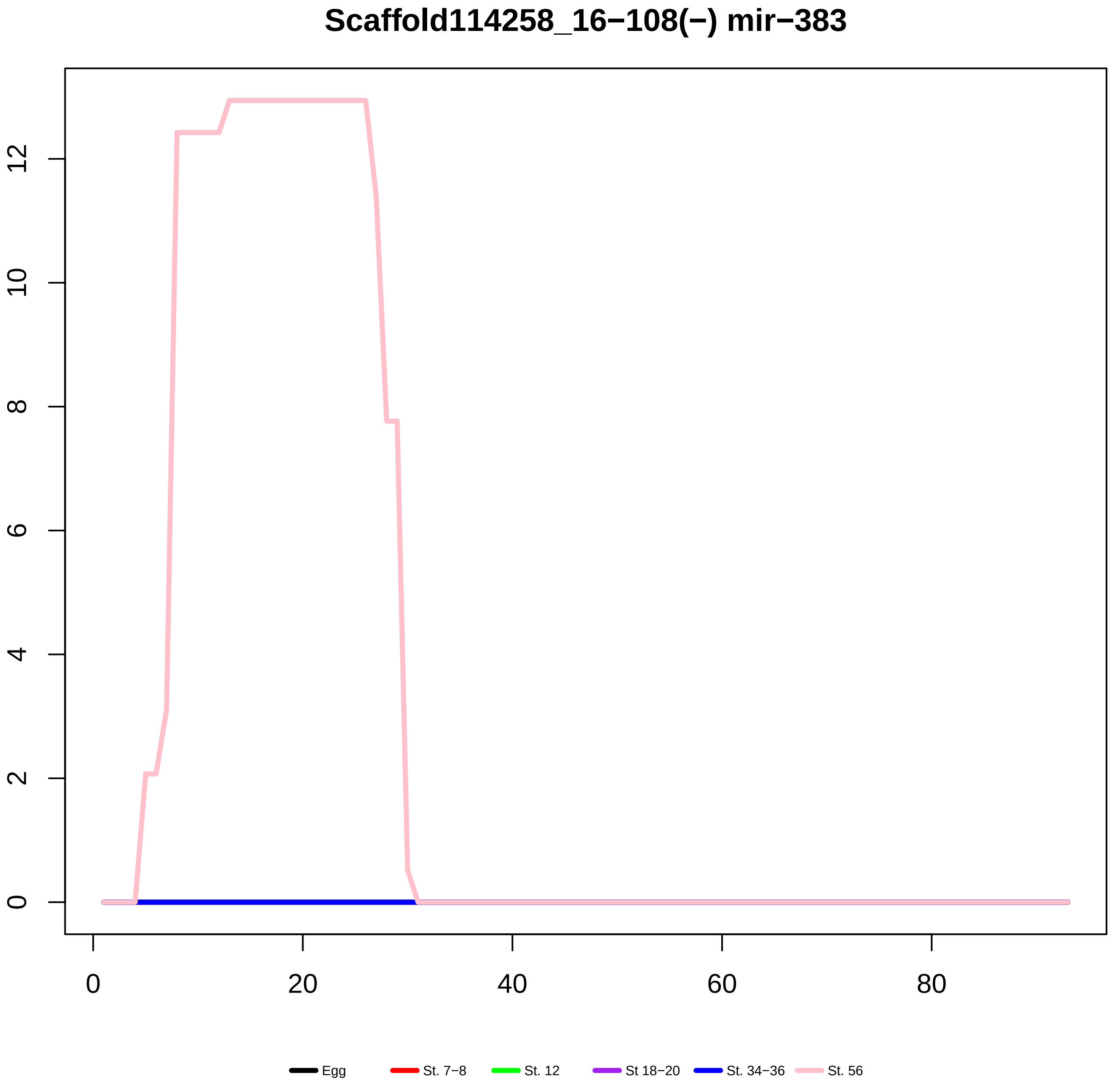
Scaffold114258_16−108(−) mir−383
0
2
4
6
8
10
12
0
20
40
60
80
Egg
St. 7−8
St. 12
St 18−20
St. 34−36
St. 56

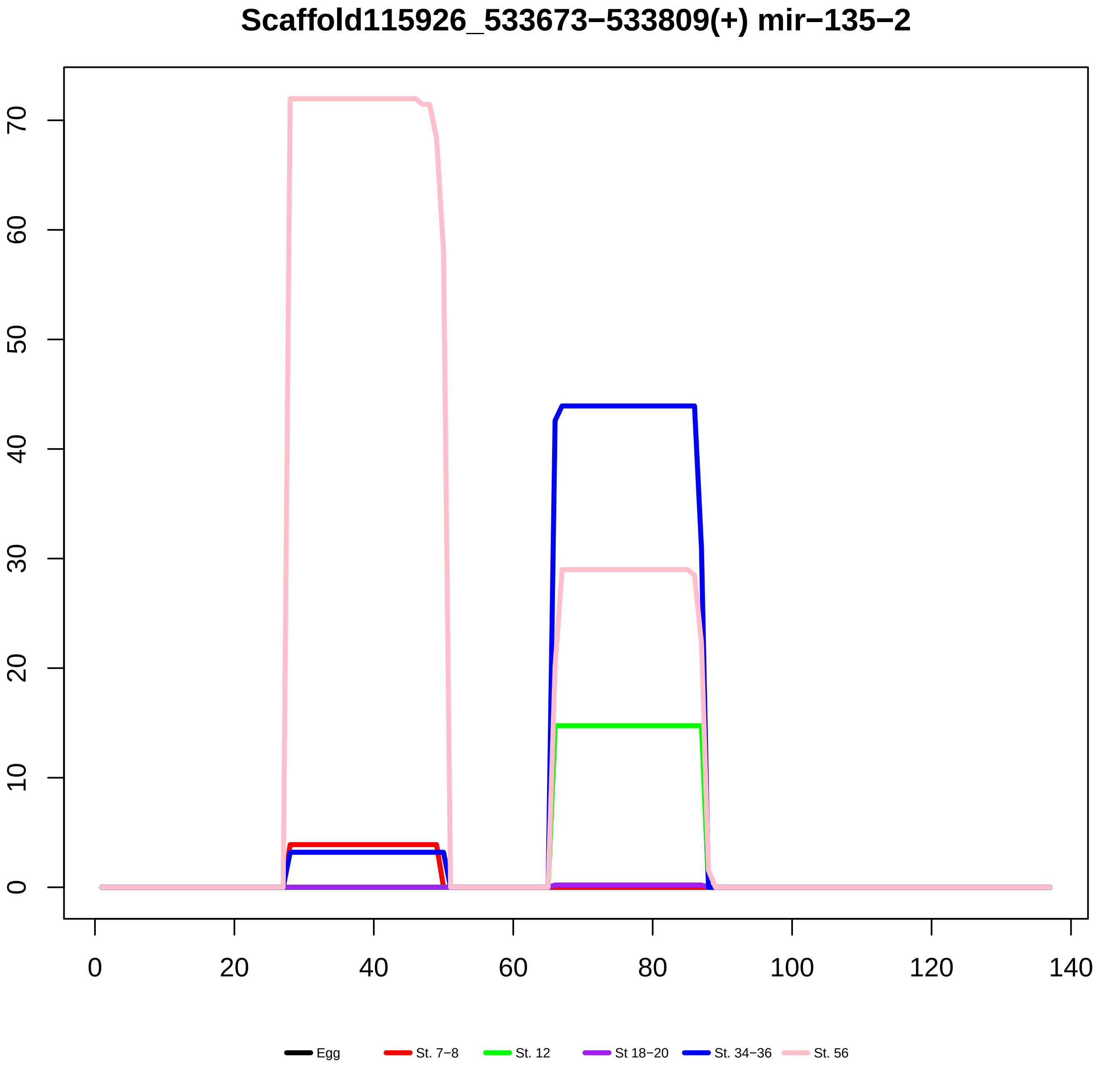

Scaffold115926_533673−533809(+) mir−135−2
0
10
20
30
40
50
60
70
0
20
40
60
80
100
120
140
Egg
St. 7−8
St. 12
St 18−20
St. 34−36
St. 56

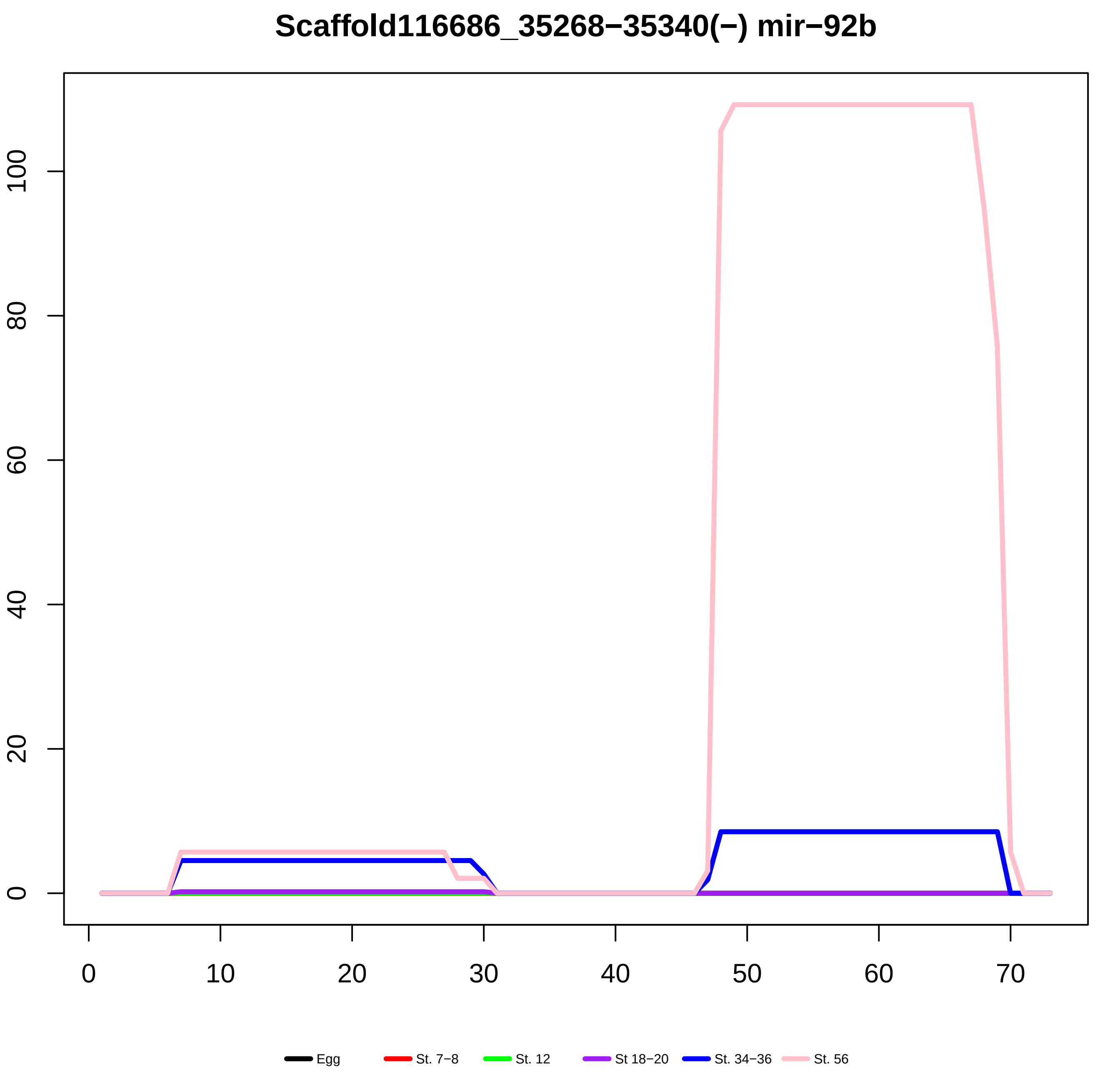

Scaffold116686_35268−35340(−) mir−92b
0
20
40
60
80
100
0
10
20
30
40
50
60
70
Egg
St. 7−8
St. 12
St 18−20
St. 34−36
St. 56

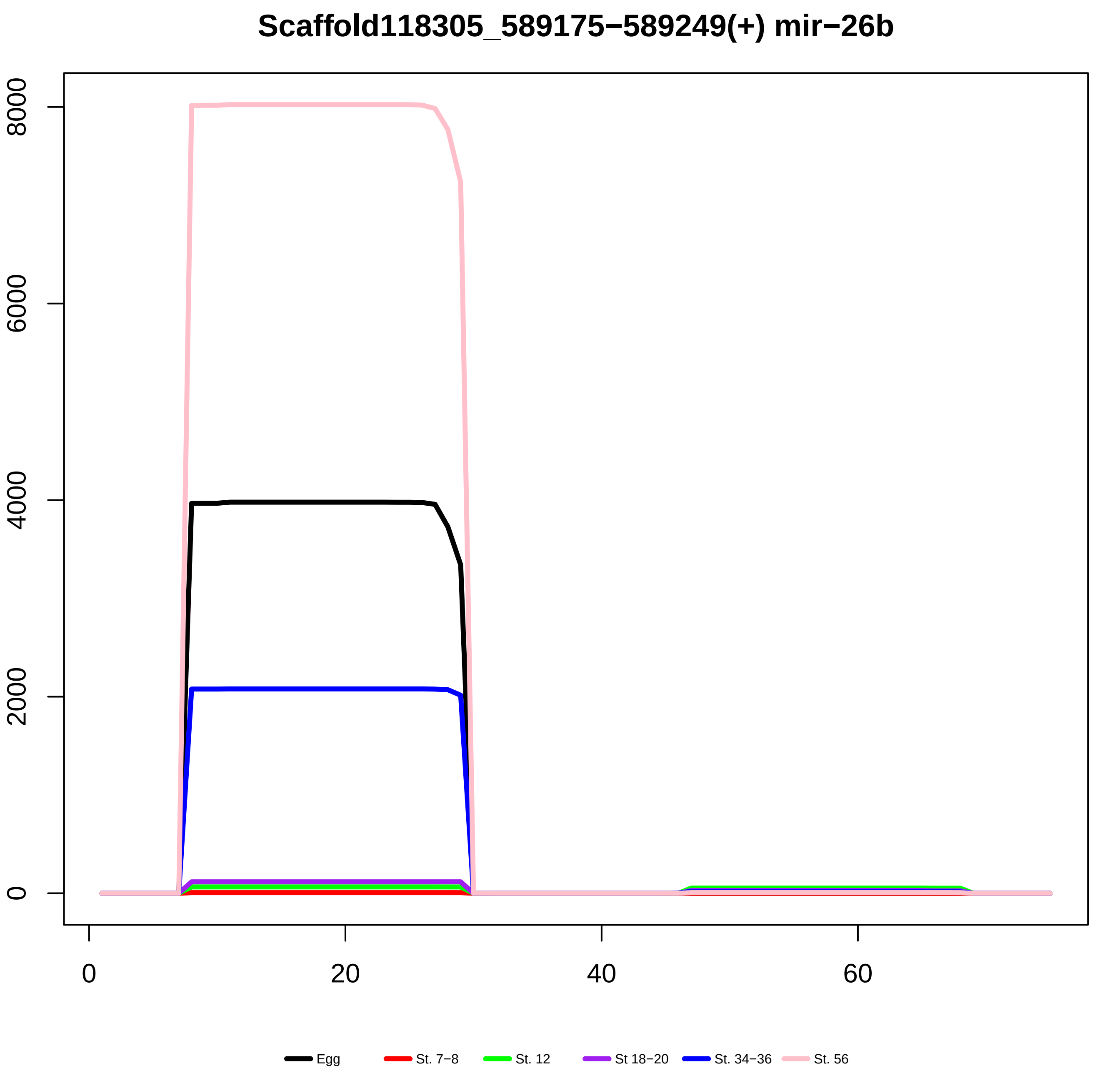
Scaffold118305_589175−589249(+) mir−26b
8000
6000
4000
2000
0
0
20
40
60
Egg
St. 7−8
St. 12
St 18−20
St. 34−36
St. 56

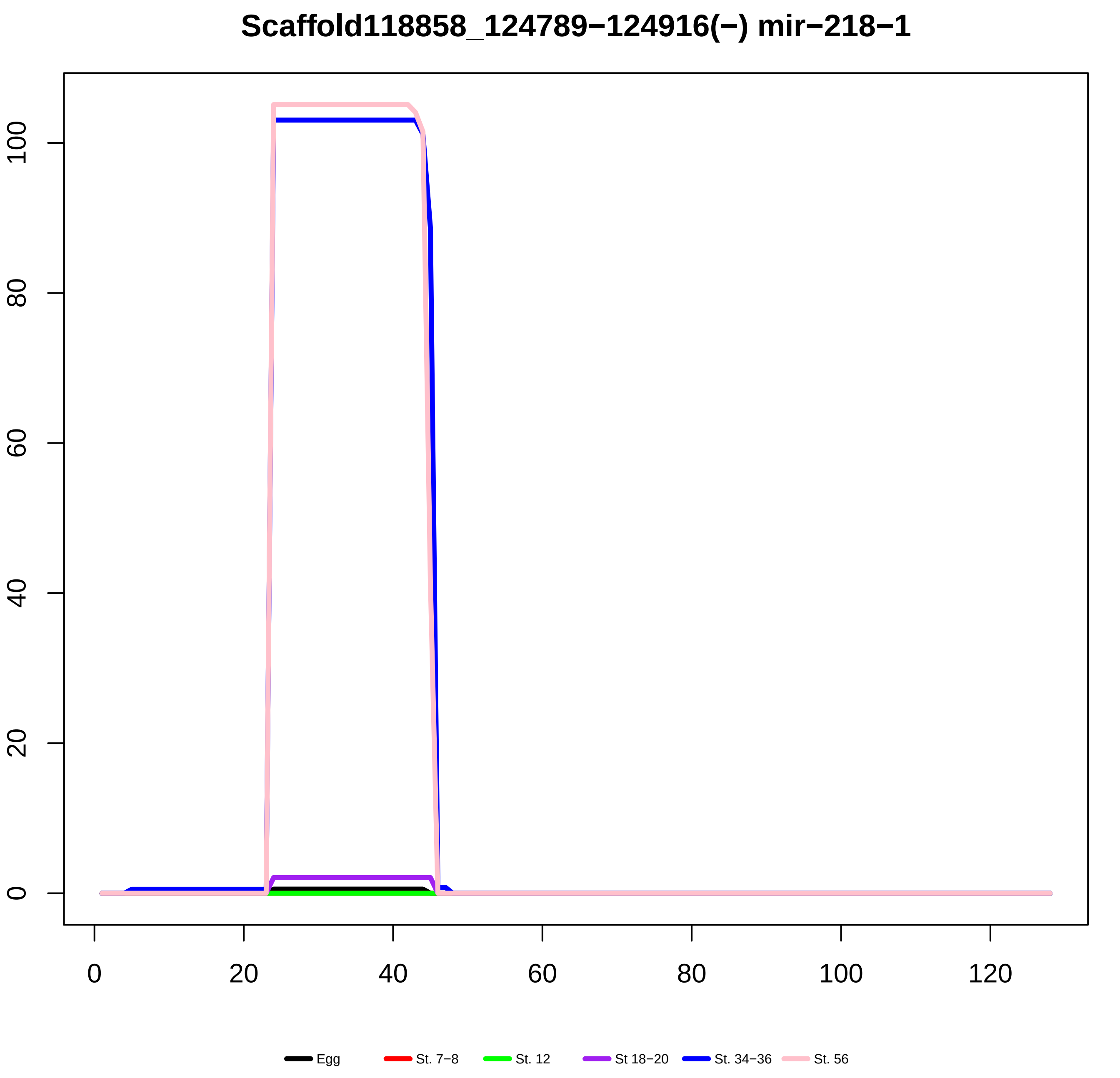

Scaffold118858_124789−124916(−) mir−218−1
0
20
40
60
80
100
0
20
40
60
80
100
120
Egg
St. 7−8
St. 12
St 18−20
St. 34−36
St. 56

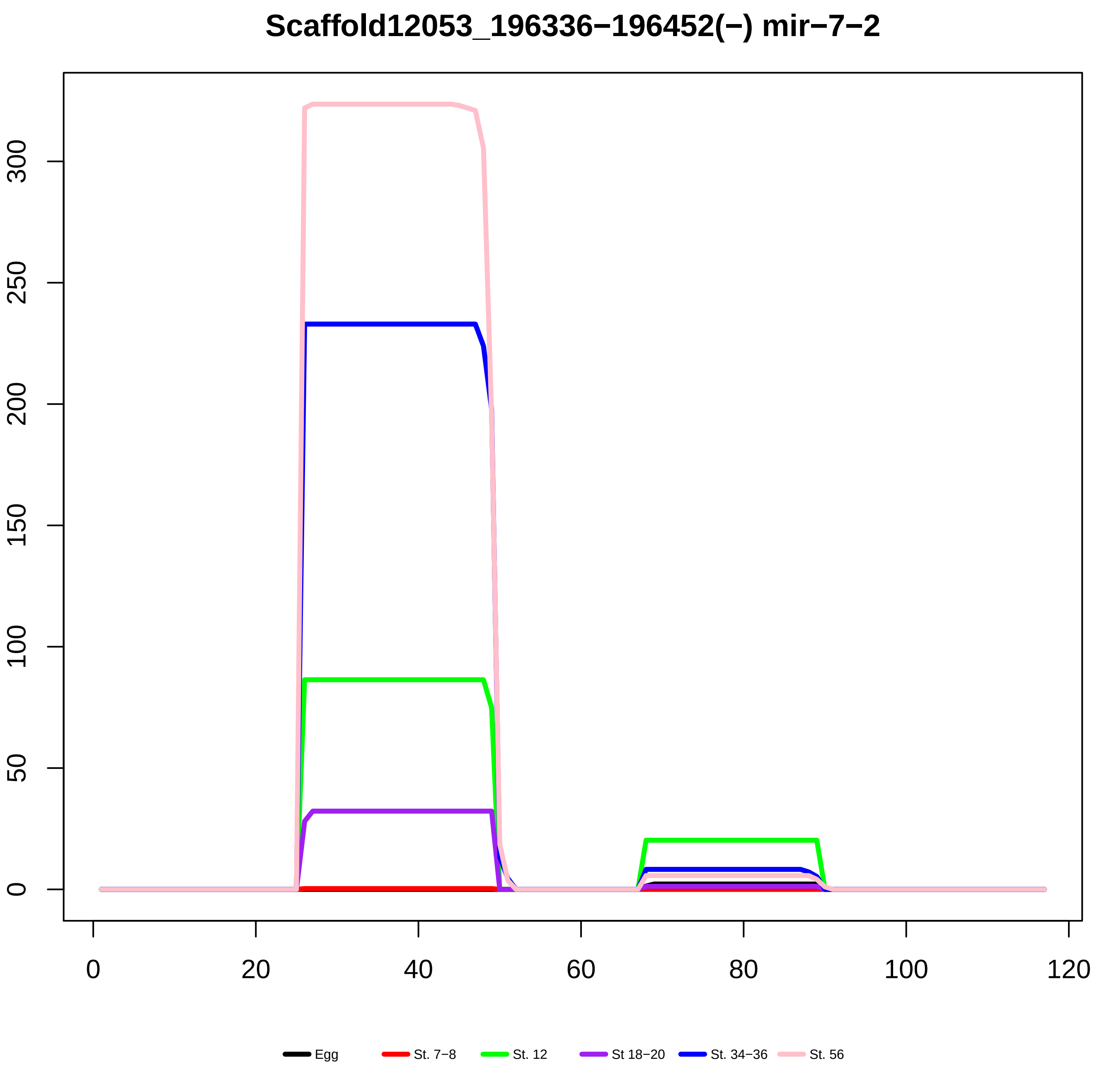
Scaffold12053_196336−196452(−) mir−7−2
0
50
100
150
200
250
300
0
20
40
60
80
100
120
Egg
St. 7−8
St. 12
St 18−20
St. 34−36
St. 56

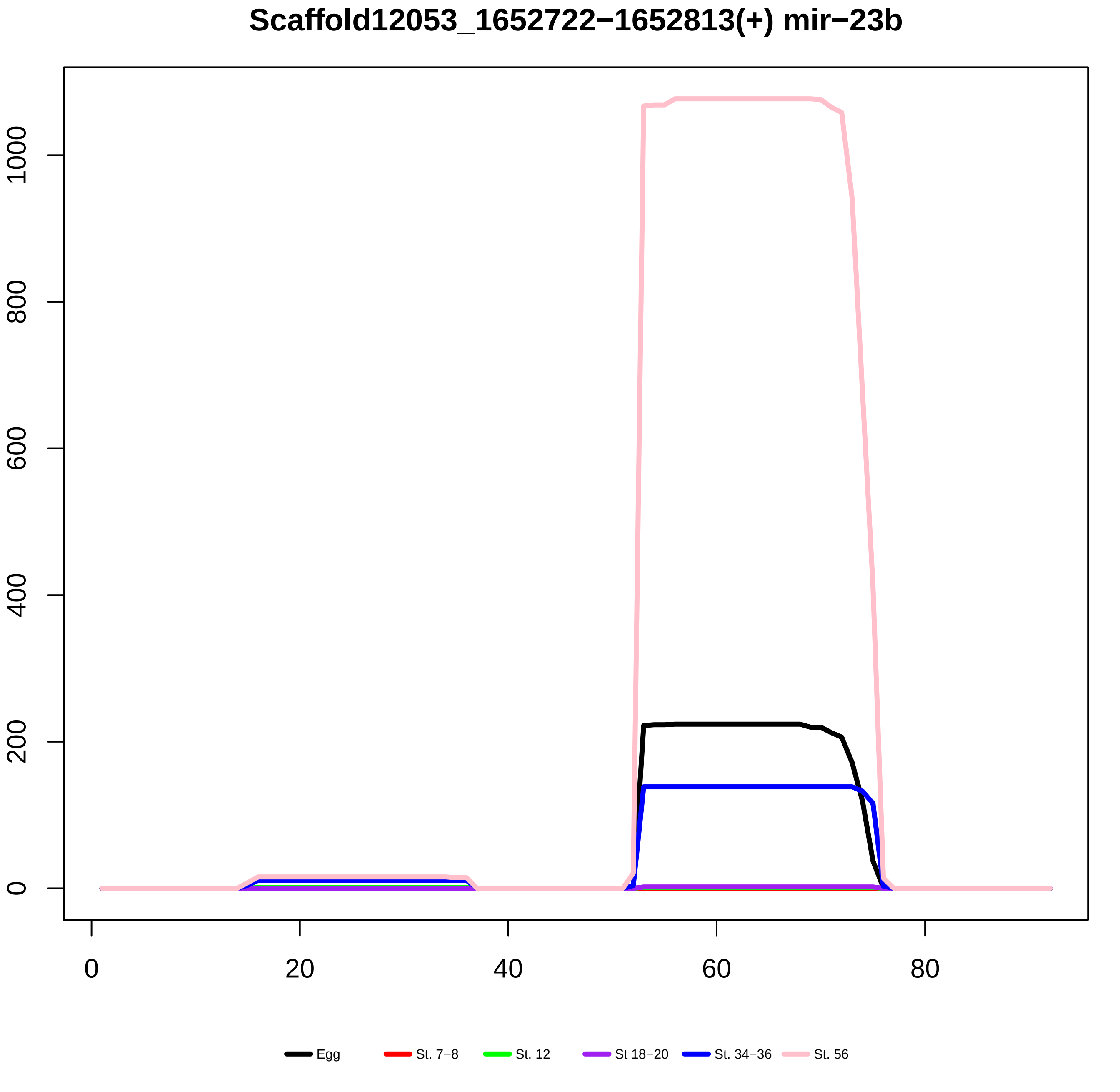

Scaffold12053_1652722−1652813(+) mir−23b
0
200
400
600
800
1000
0
20
40
60
80
Egg
St. 7−8
St. 12
St 18−20
St. 34−36
St. 56

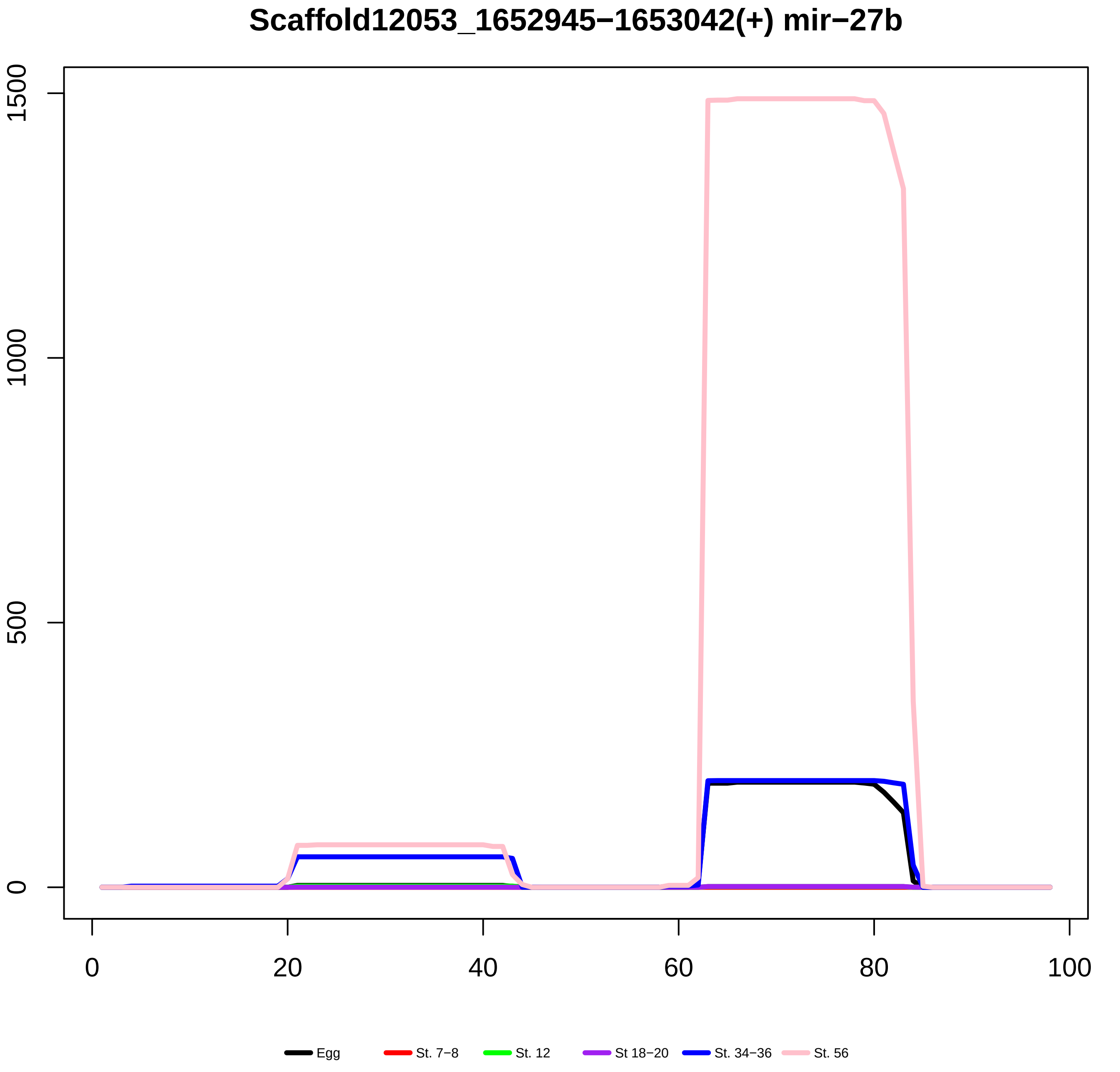

Scaffold12053_1652945−1653042(+) mir−27b
1500
1000
500
0
0
20
40
60
80
100
Egg
St. 7−8
St. 12
St 18−20
St. 34−36
St. 56

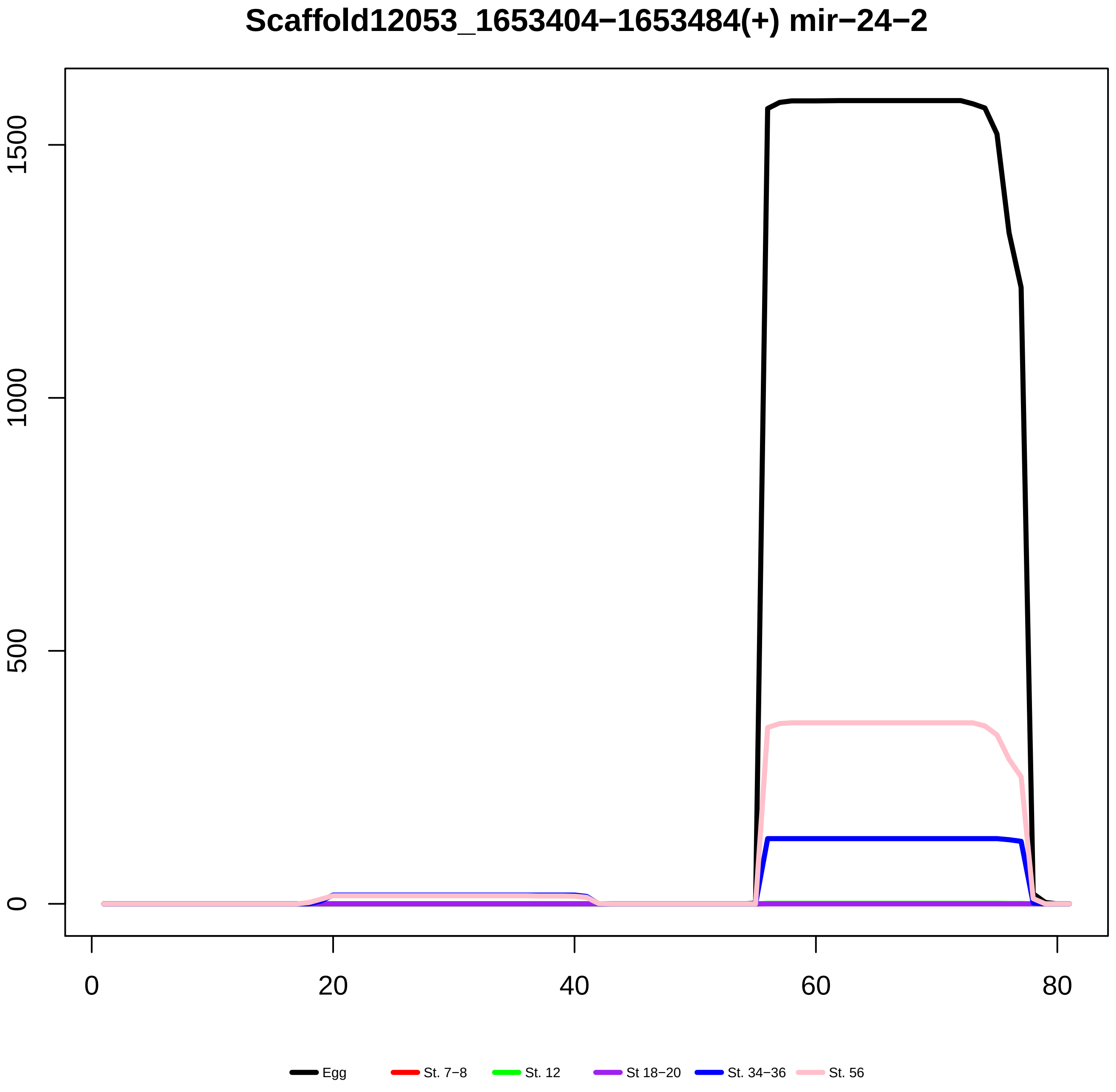

Scaffold12053_1653404−1653484(+) mir−24−2
0
500
1000
1500
0
20
40
60
80
Egg
St. 7−8
St. 12
St 18−20
St. 34−36
St. 56

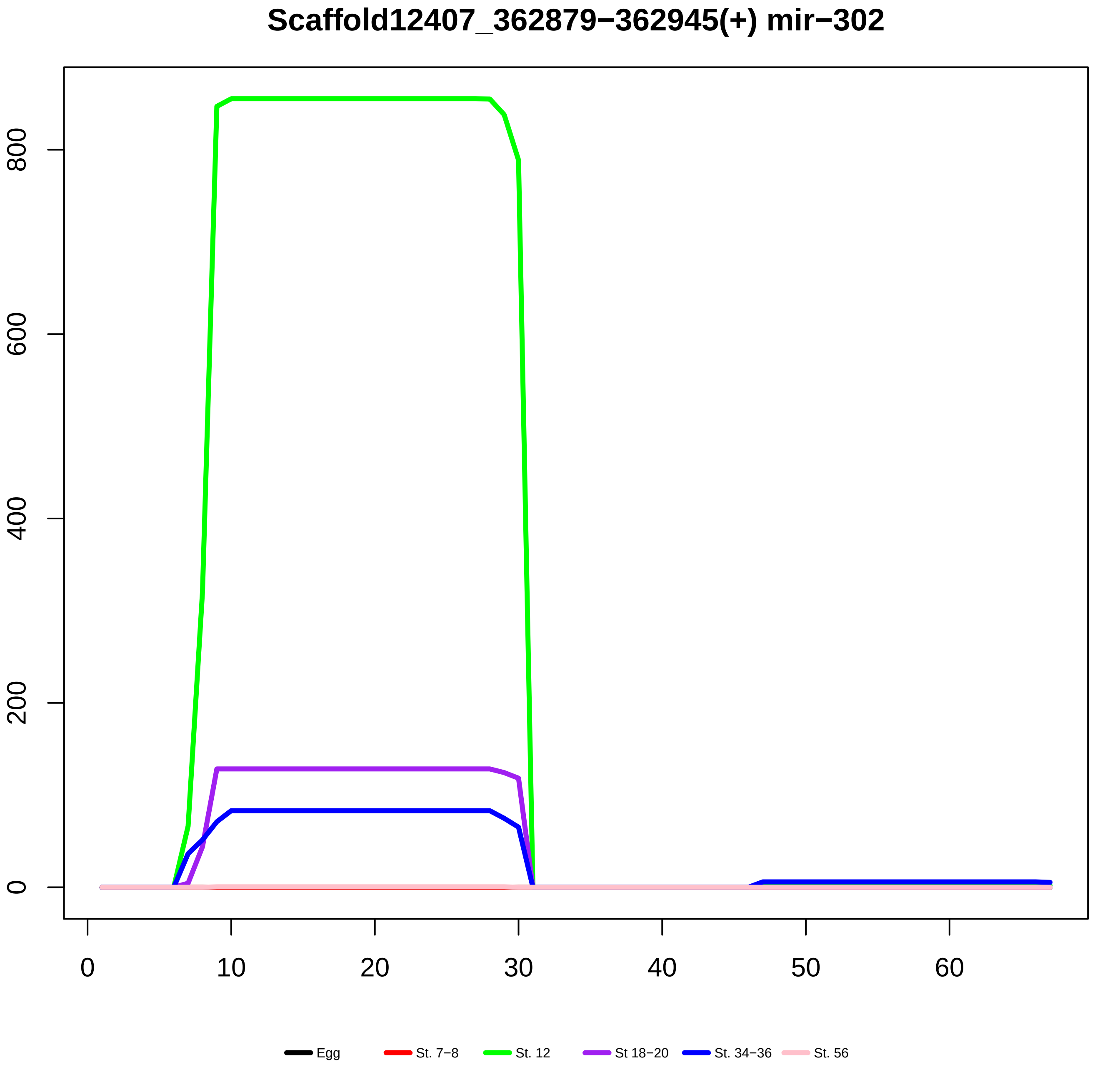

Scaffold12407_362879−362945(+) mir−302
0
200
400
600
800
0
10
20
30
40
50
60
Egg
St. 7−8
St. 12
St 18−20
St. 34−36
St. 56

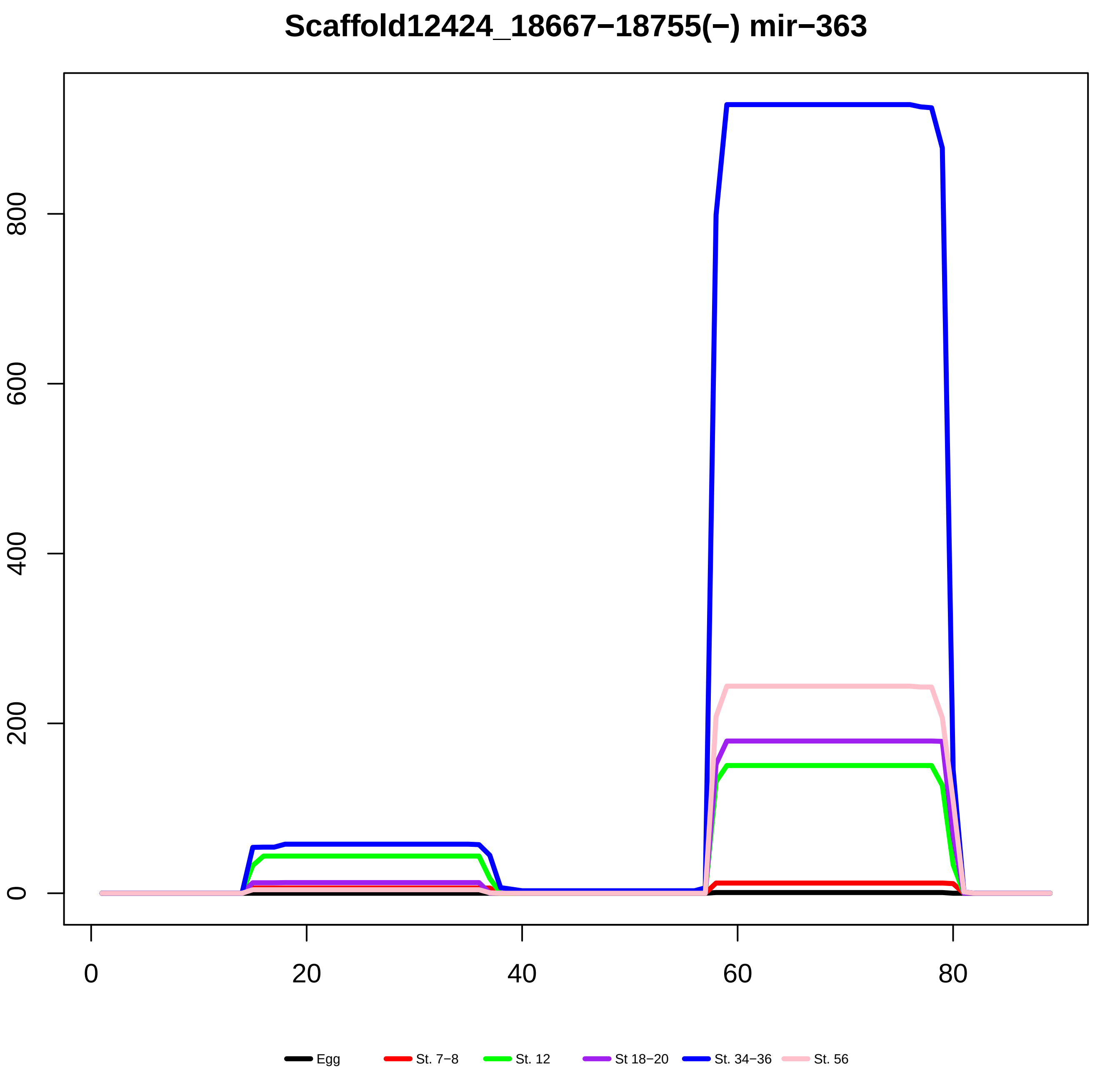

Scaffold12424_18667−18755(−) mir−363
0
200
400
600
800
0
20
40
60
80
Egg
St. 7−8
St. 12
St 18−20
St. 34−36
St. 56

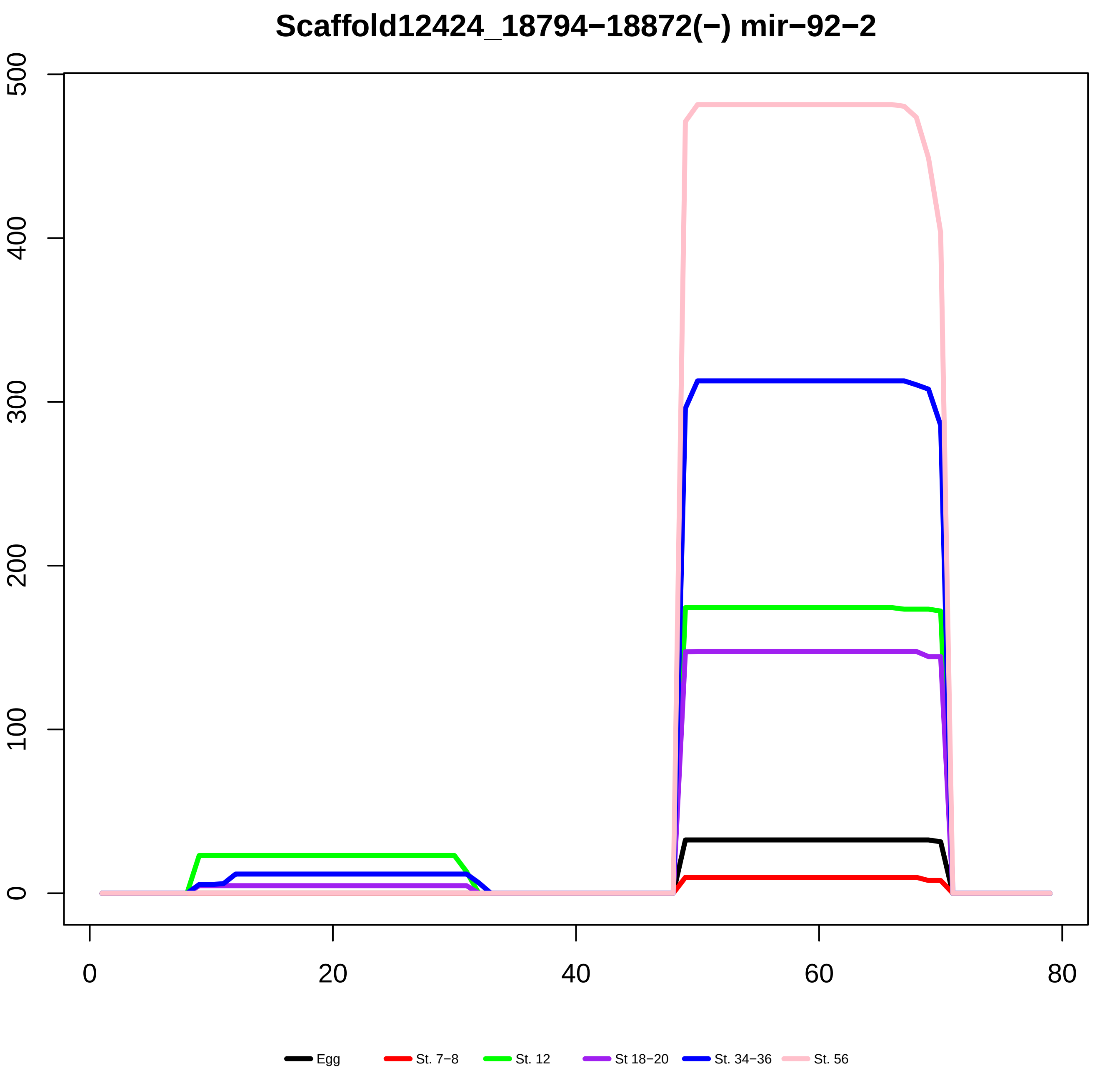
Scaffold12424_18794−18872(−) mir−92−2
0
100
200
300
400
500
0
20
40
60
80
Egg
St. 7−8
St. 12
St 18−20
St. 34−36
St. 56

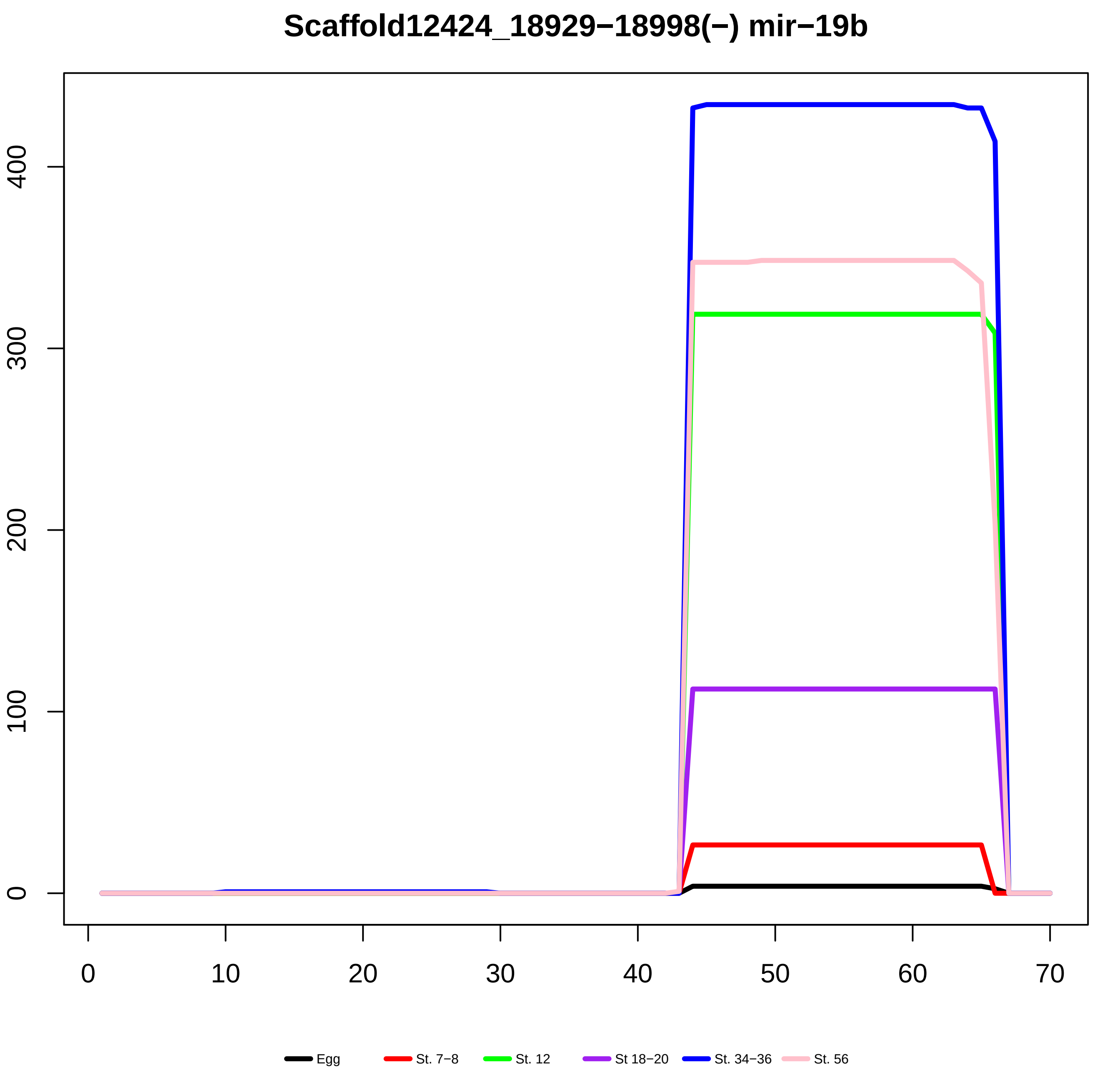

Scaffold12424_18929−18998(−) mir−19b
0
100
200
300
400
0
10
20
30
40
50
60
70
Egg
St. 7−8
St. 12
St 18−20
St. 34−36
St. 56

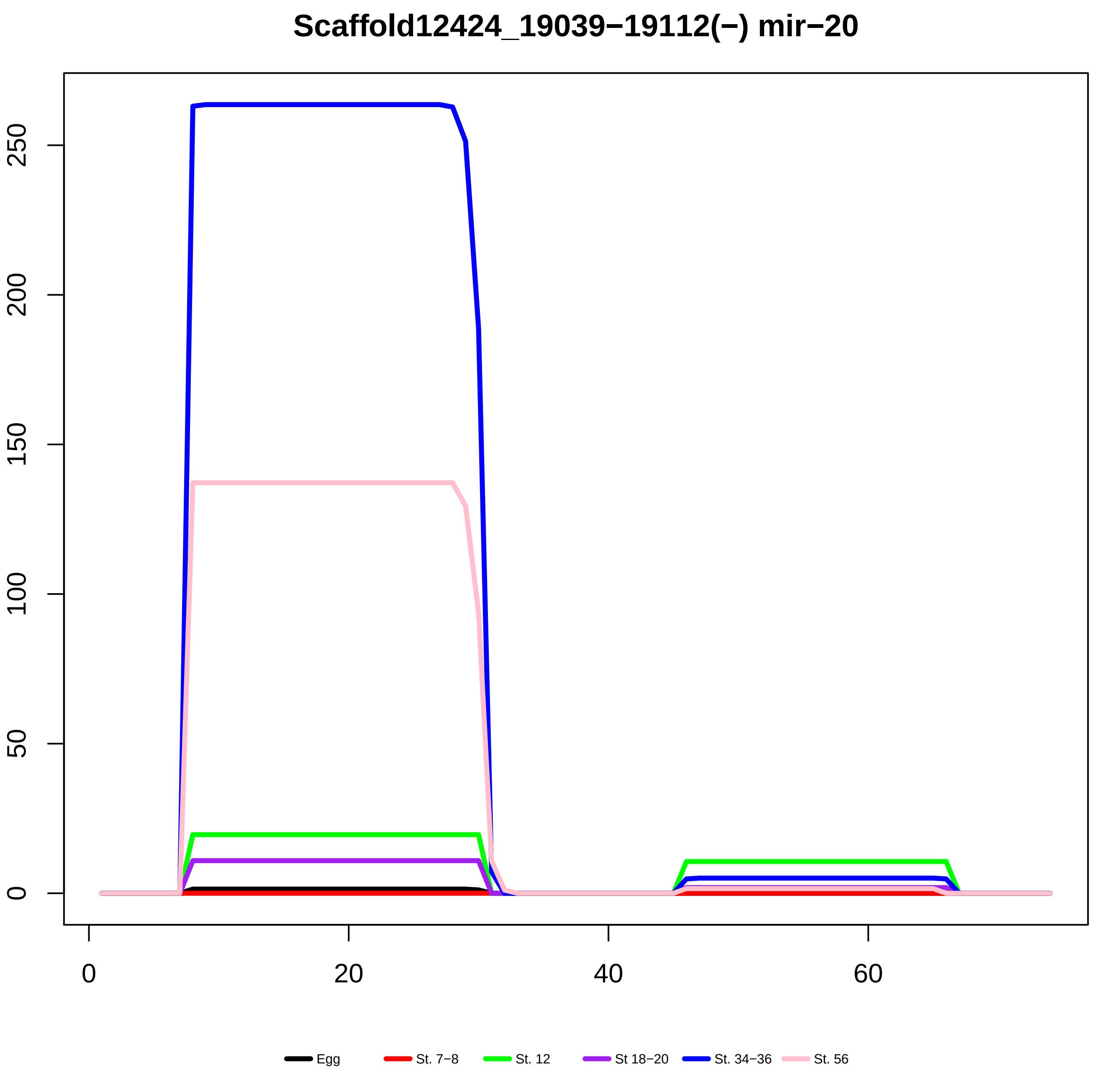

Scaffold12424_19039−19112(−) mir−20
0
50
100
150
200
250
0
20
40
60
Egg
St. 7−8
St. 12
St 18−20
St. 34−36
St. 56

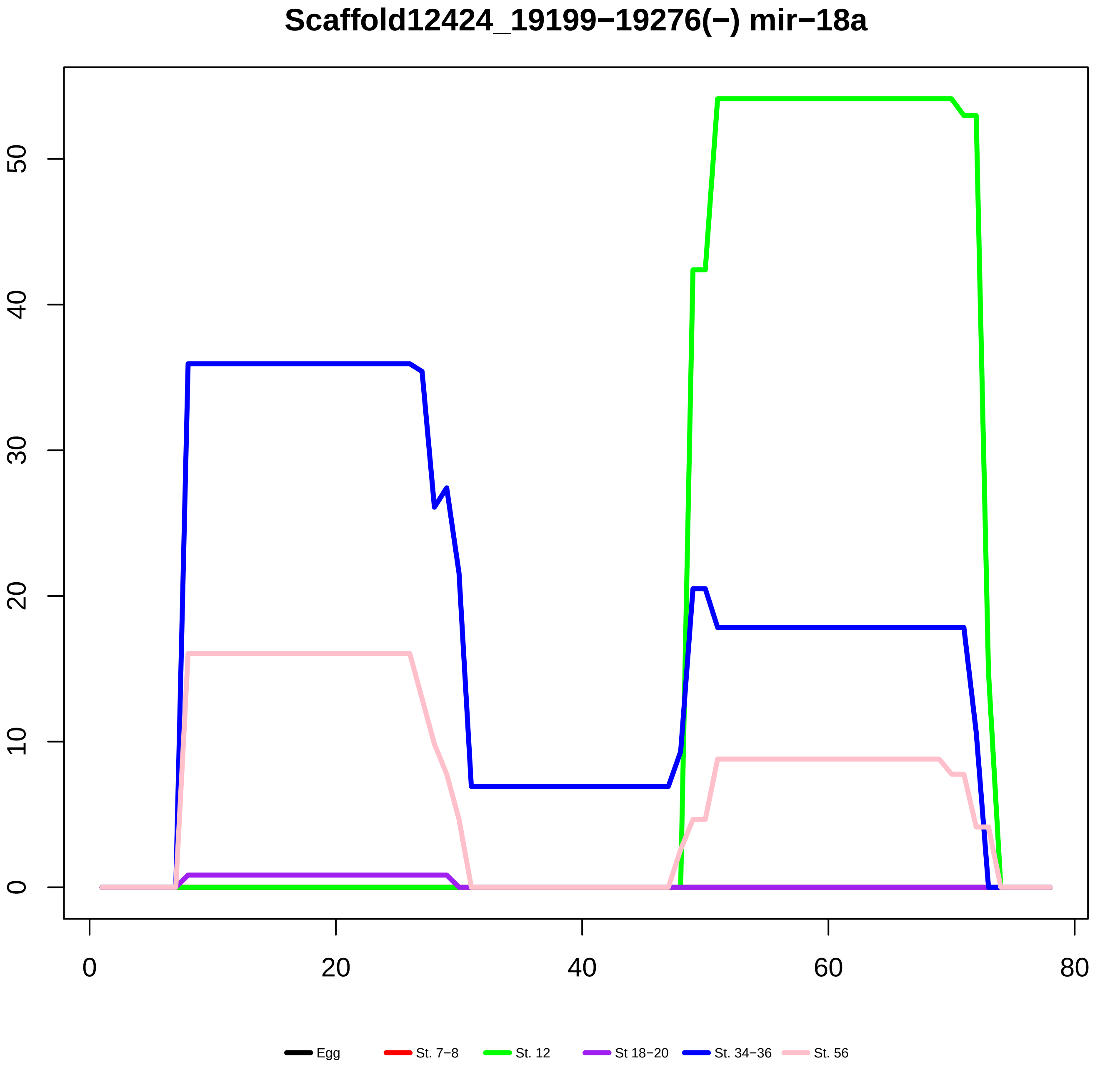

Scaffold12424_19199−19276(−) mir−18a
0
10
20
30
40
50
0
20
40
60
80
Egg
St. 7−8
St. 12
St 18−20
St. 34−36
St. 56

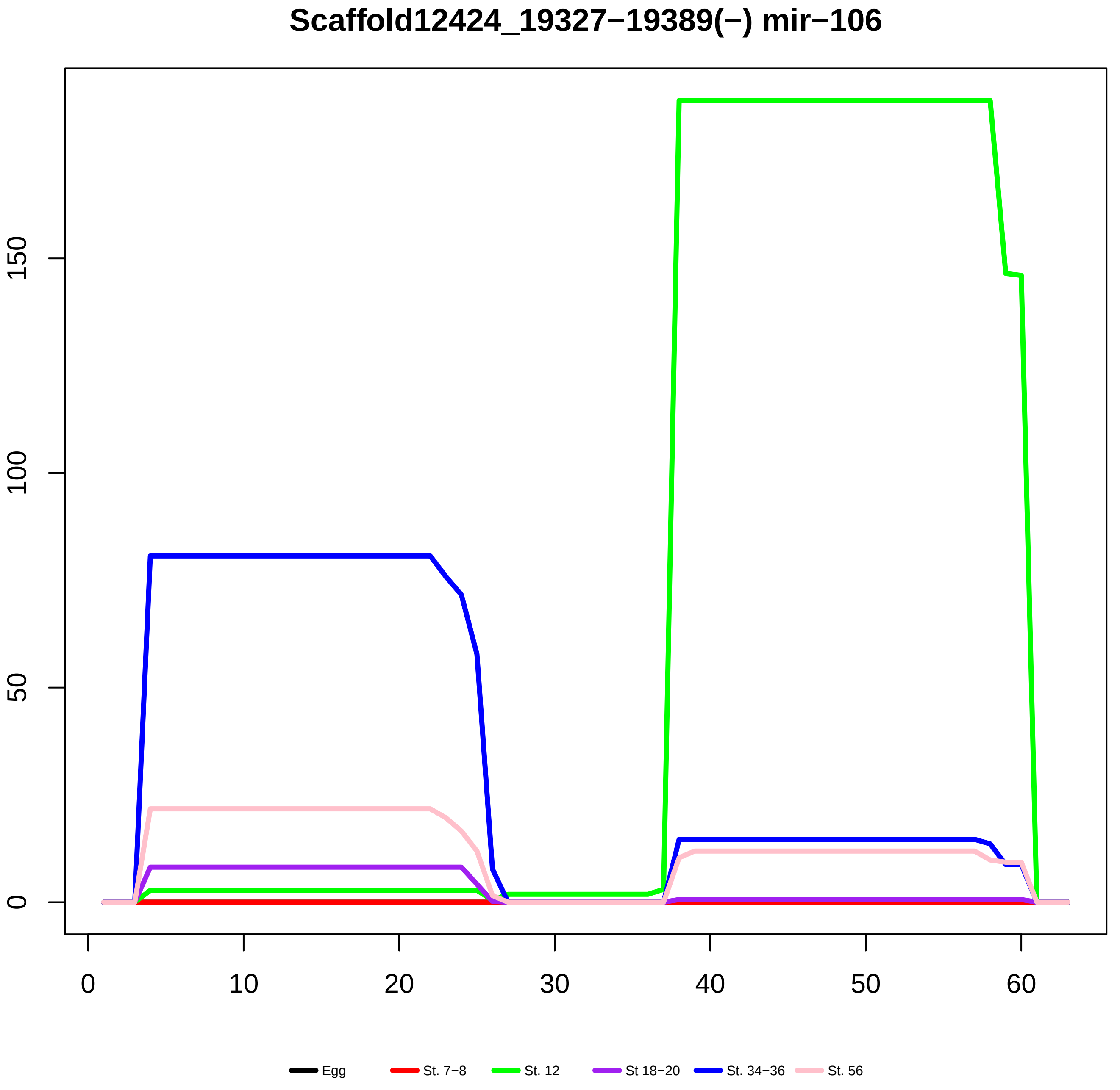
Scaffold12424_19327−19389(−) mir−106
0
50
100
150
0
10
20
30
40
50
60
Egg
St. 7−8
St. 12
St 18−20
St. 34−36
St. 56

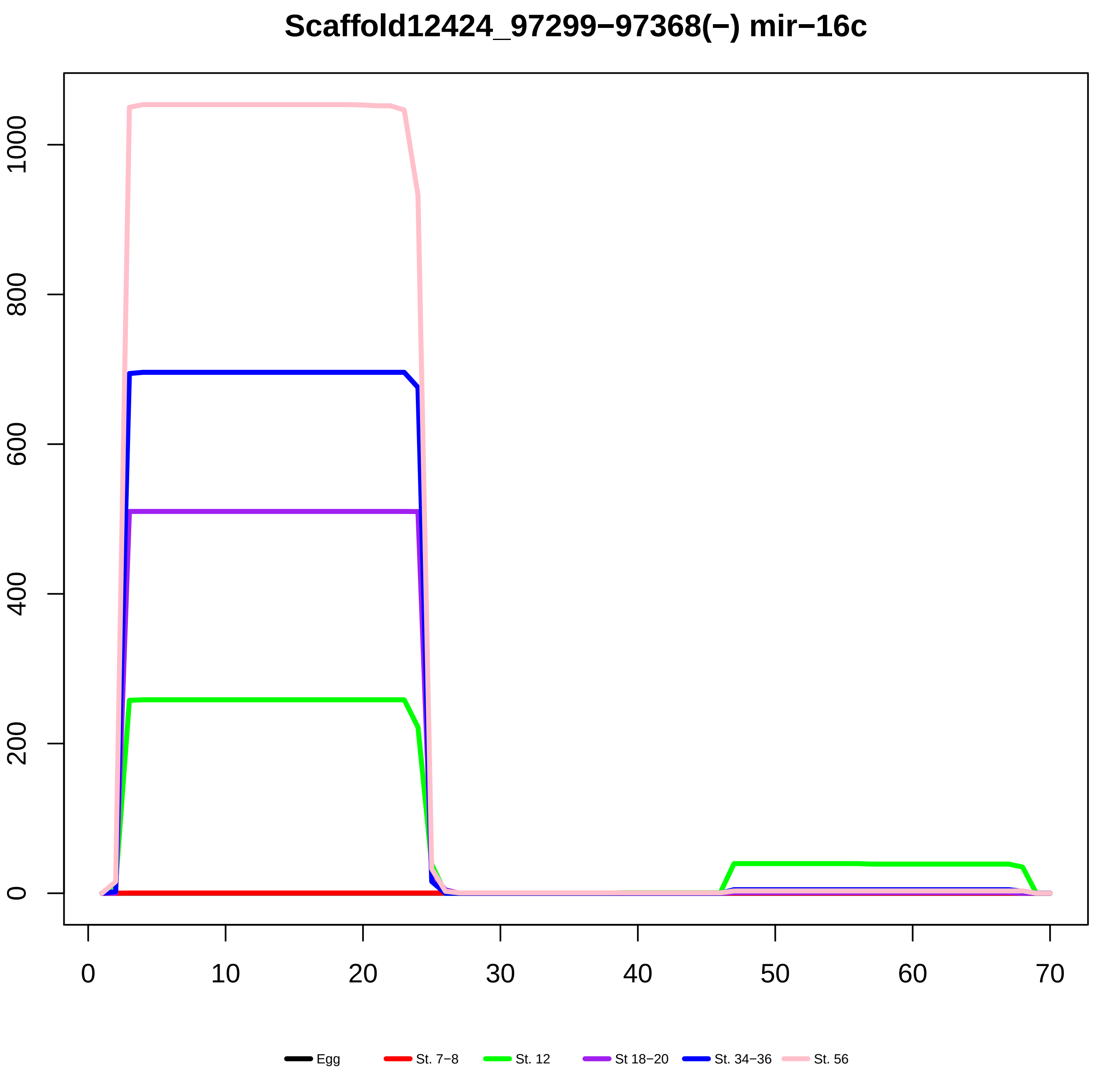

Scaffold12424_97299−97368(−) mir−16c
0
200
400
600
800
1000
0
10
20
30
40
50
60
70
Egg
St. 7−8
St. 12
St 18−20
St. 34−36
St. 56

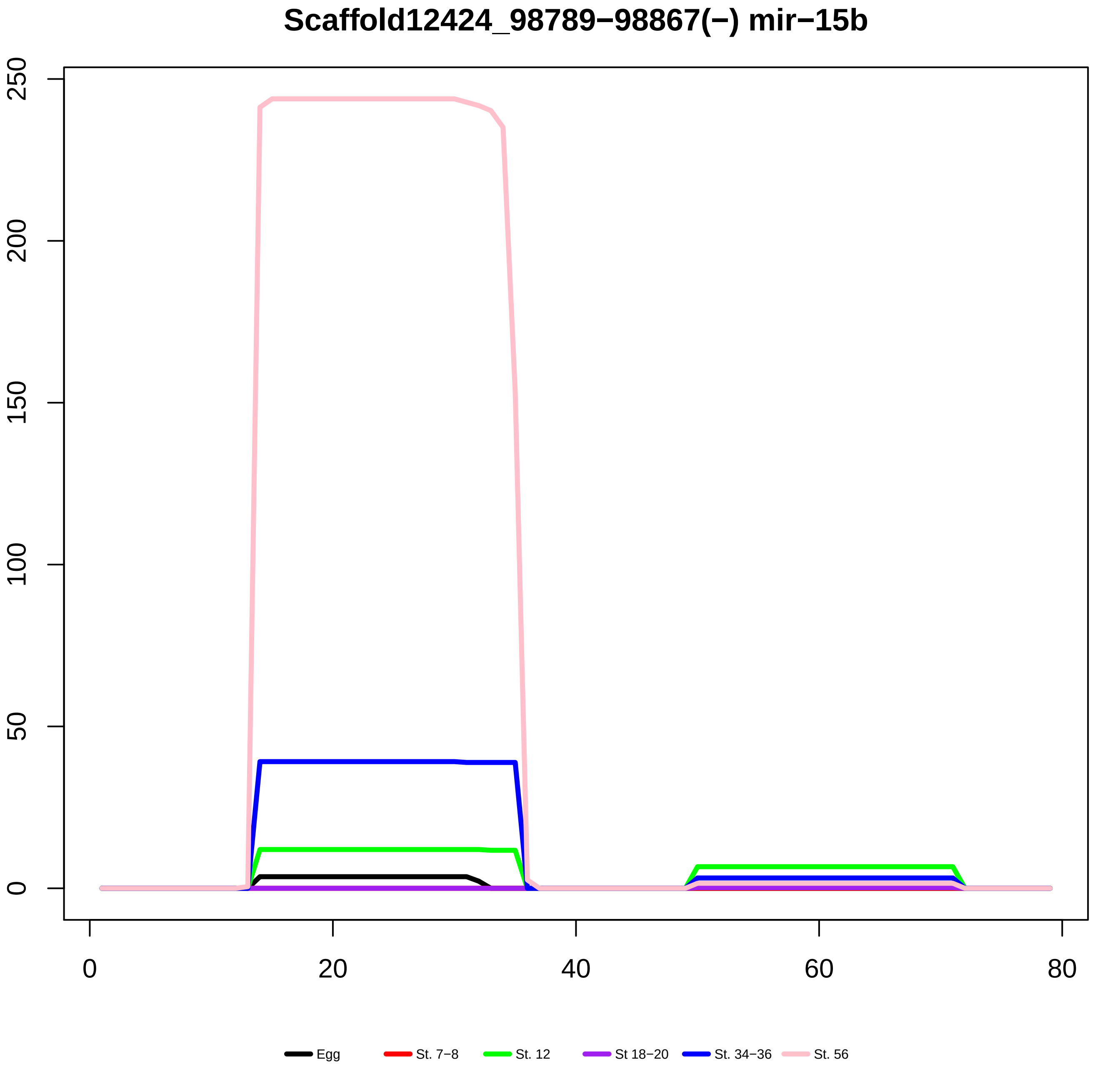
Scaffold12424_98789−98867(−) mir−15b
250
200
150
100
50
0
0
20
40
60
80
Egg
St. 7−8
St. 12
St 18−20
St. 34−36
St. 56

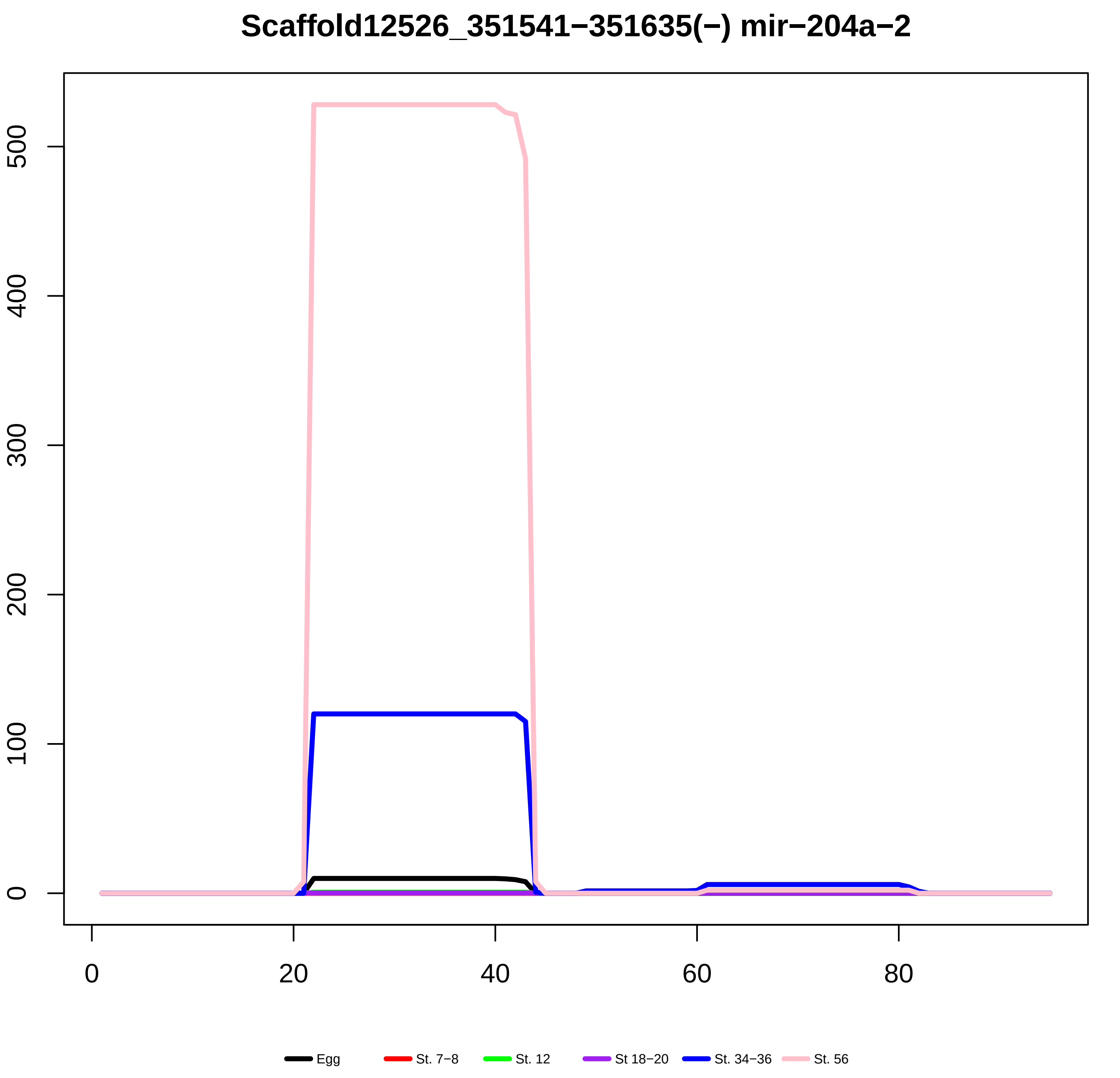

Scaffold12526_351541−351635(−) mir−204a−2
0
100
200
300
400
500
0
20
40
60
80
Egg
St. 7−8
St. 12
St 18−20
St. 34−36
St. 56

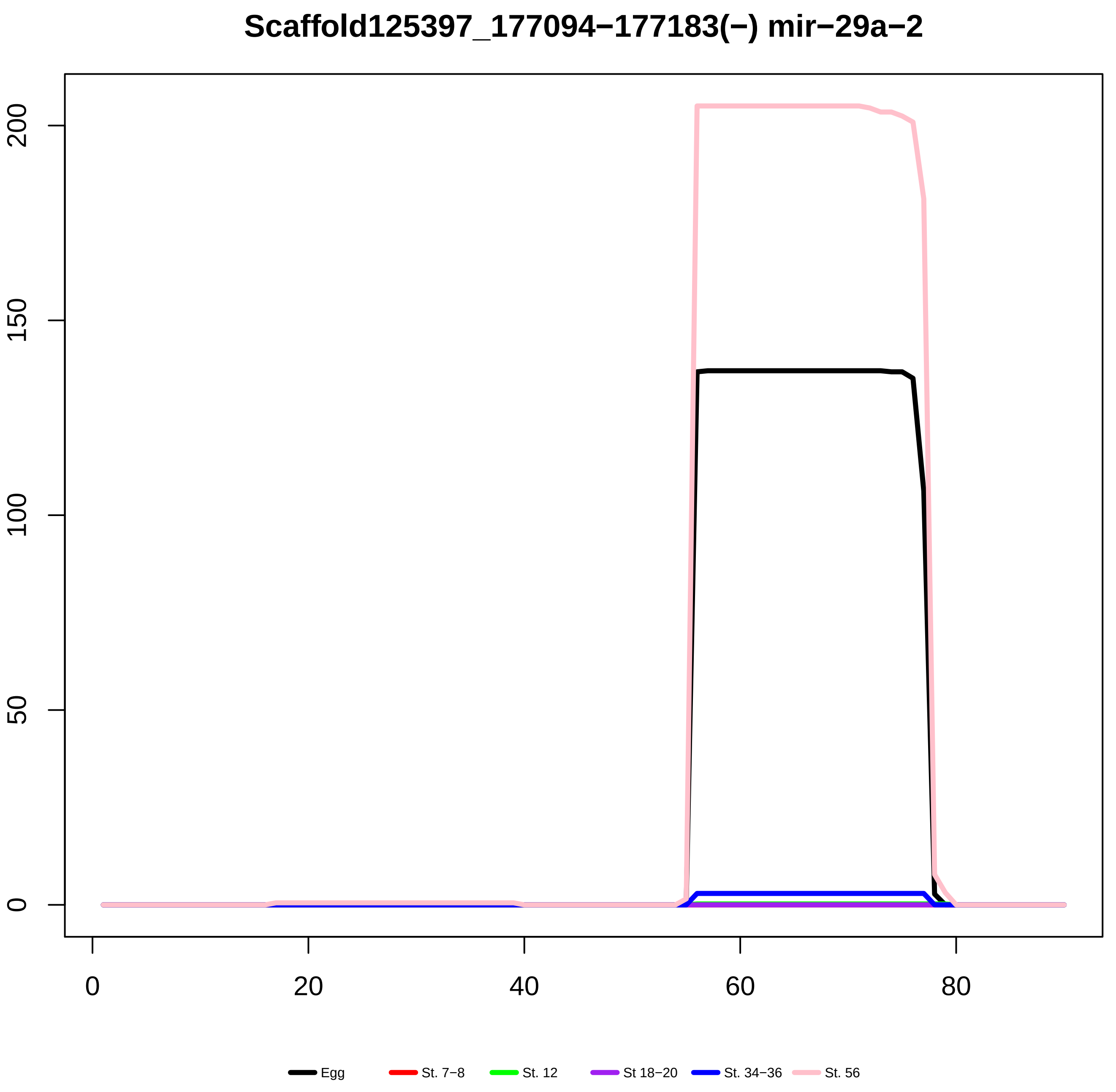

Scaffold125397_177094−177183(−) mir−29a−2
0
50
100
150
200
0
20
40
60
80
Egg
St. 7−8
St. 12
St 18−20
St. 34−36
St. 56

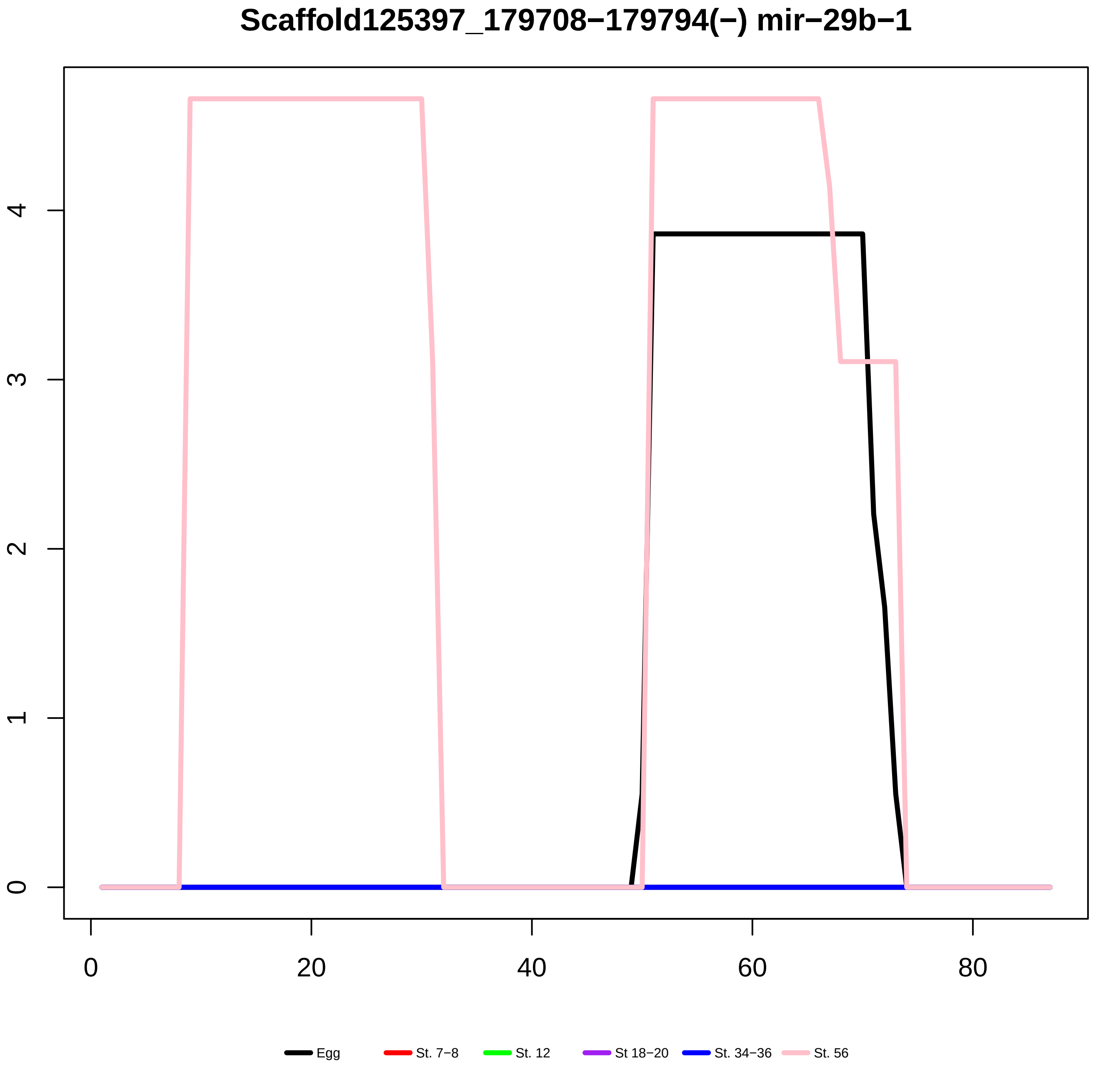

Scaffold125397_179708−179794(−) mir−29b−1
0
1
2
3
4
0
20
40
60
80
Egg
St. 7−8
St. 12
St 18−20
St. 34−36
St. 56

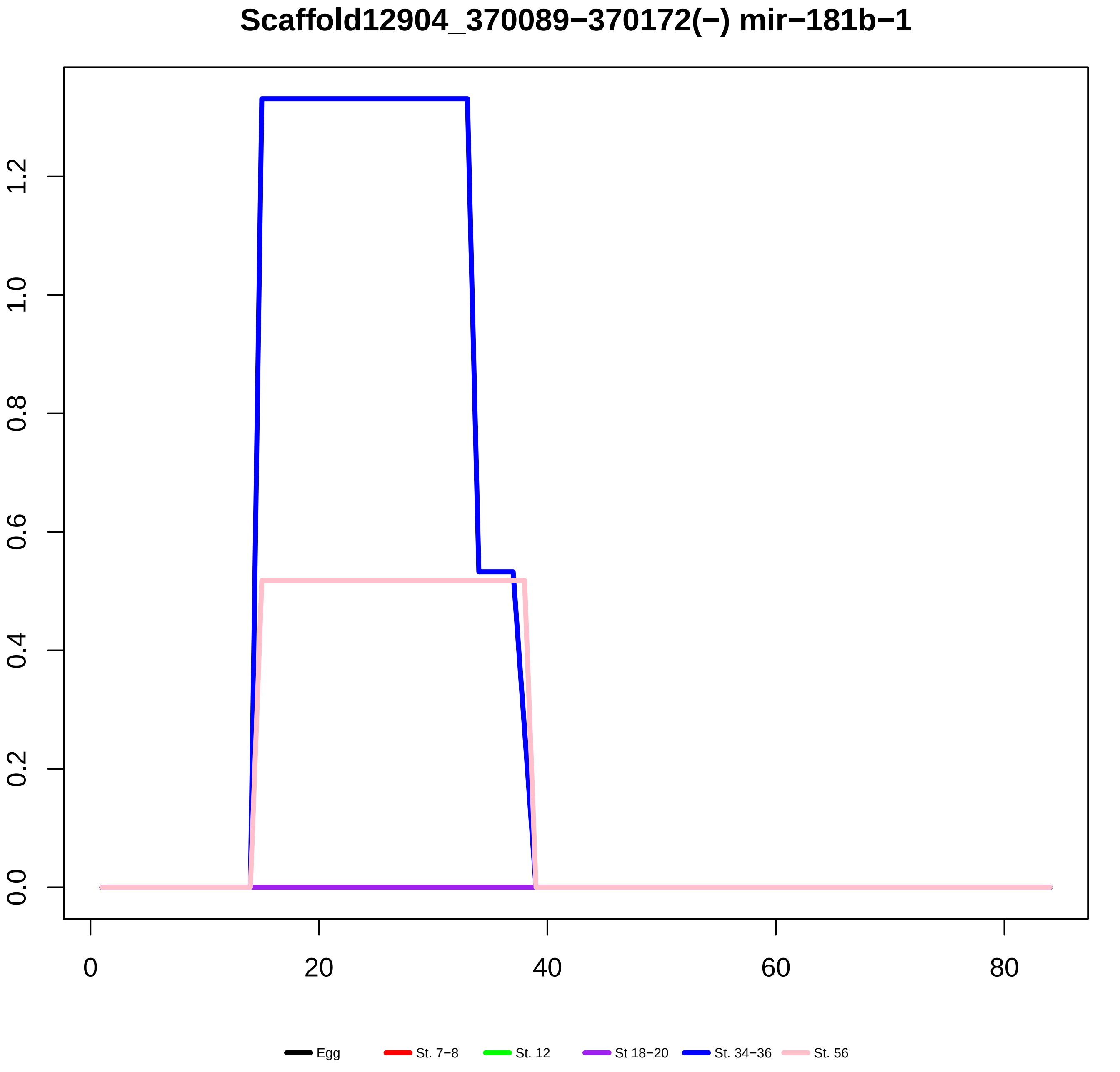
Scaffold12904_370089−370172(−) mir−181b−1
0.0
0.2
0.4
0.6
0.8
1.0
1.2
0
20
40
60
80
Egg
St. 7−8
St. 12
St 18−20
St. 34−36
St. 56

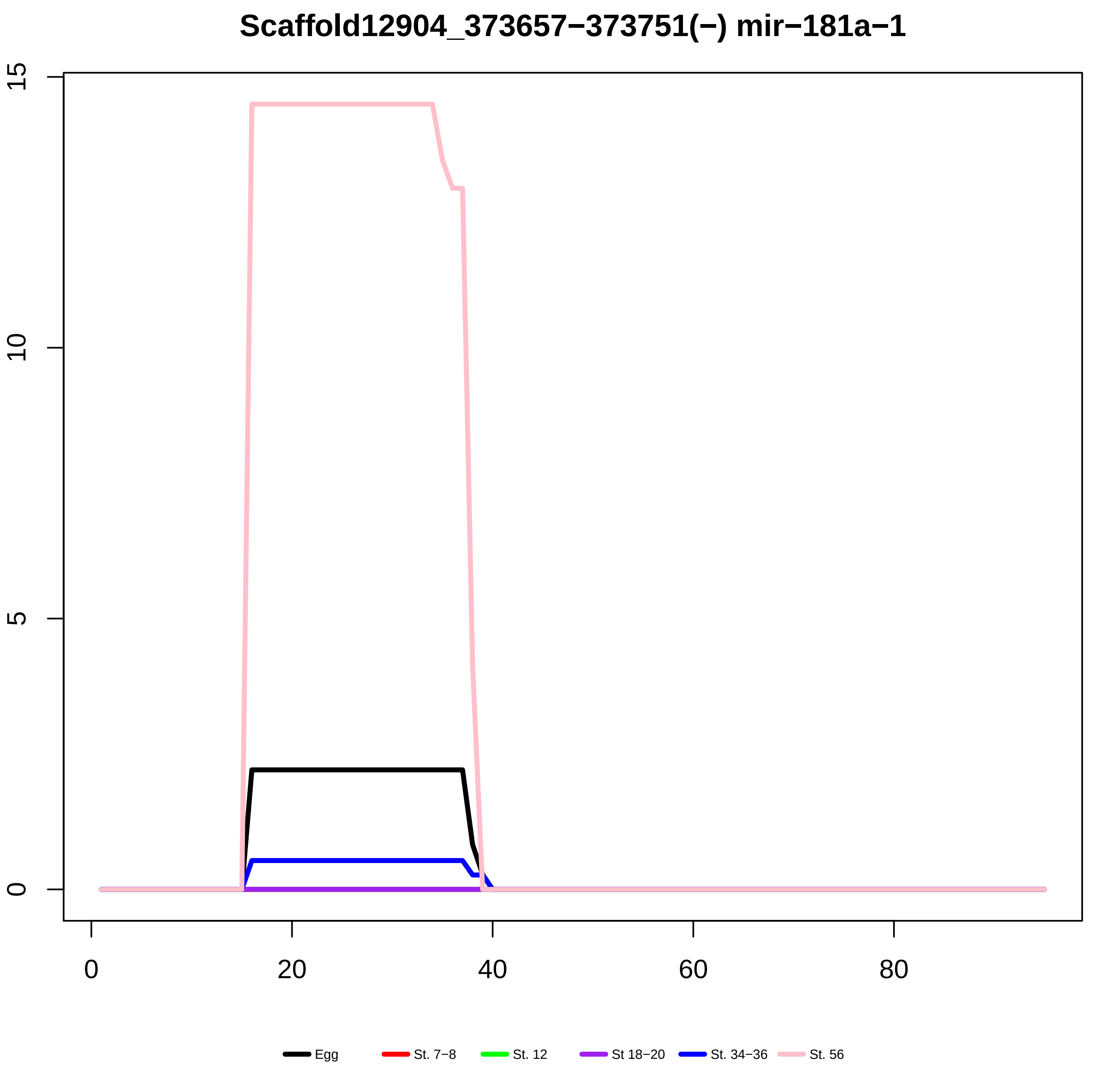
Scaffold12904_373657−373751(−) mir−181a−1
0
5
10
15
0
20
40
60
80
Egg
St. 7−8
St. 12
St 18−20
St. 34−36
St. 56

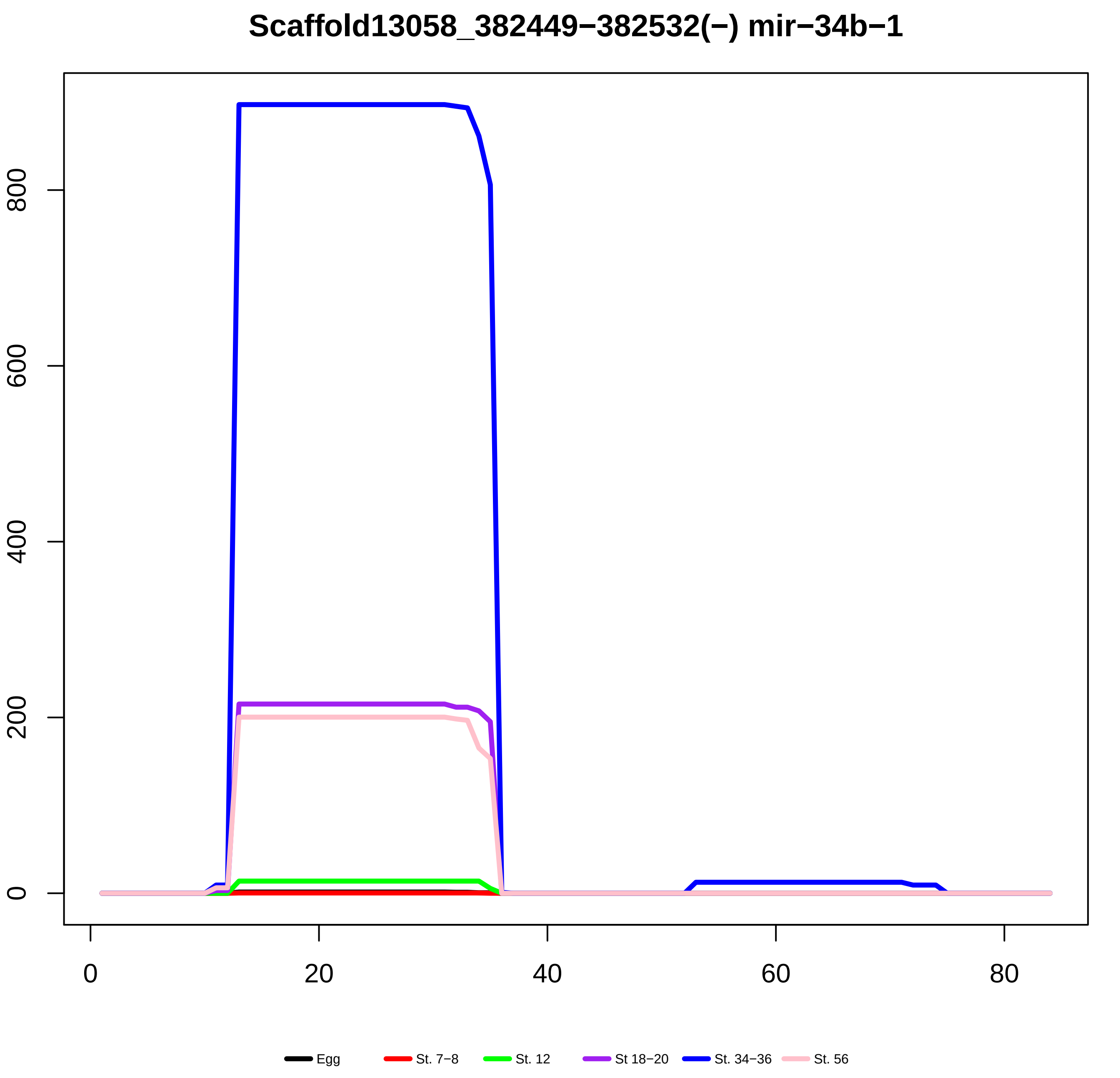
Scaffold13058_382449−382532(−) mir−34b−1
0
200
400
600
800
0
20
40
60
80
Egg
St. 7−8
St. 12
St 18−20
St. 34−36
St. 56

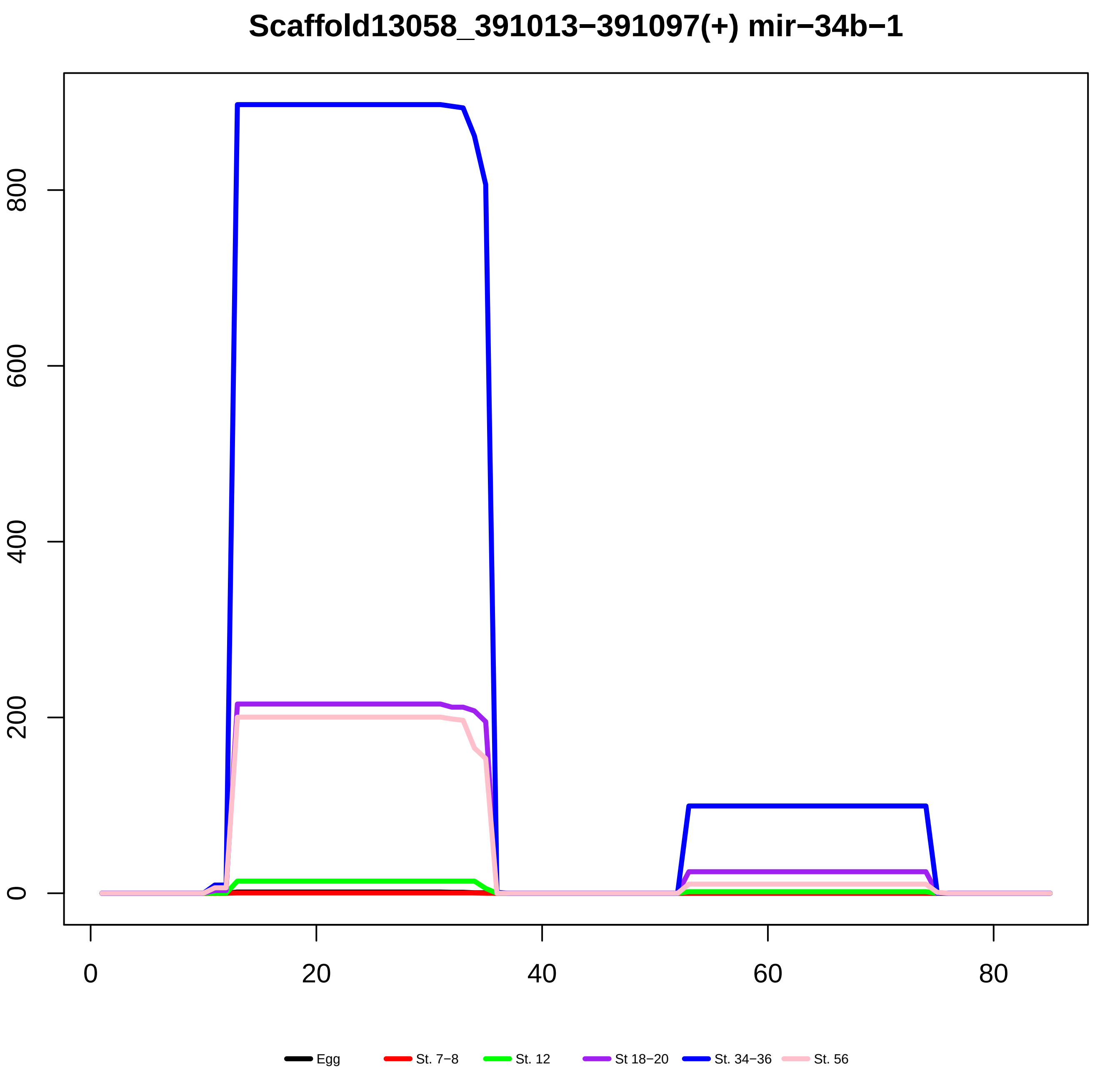
Scaffold13058_391013−391097(+) mir−34b−1
0
200
400
600
800
0
20
40
60
80
Egg
St. 7−8
St. 12
St 18−20
St. 34−36
St. 56

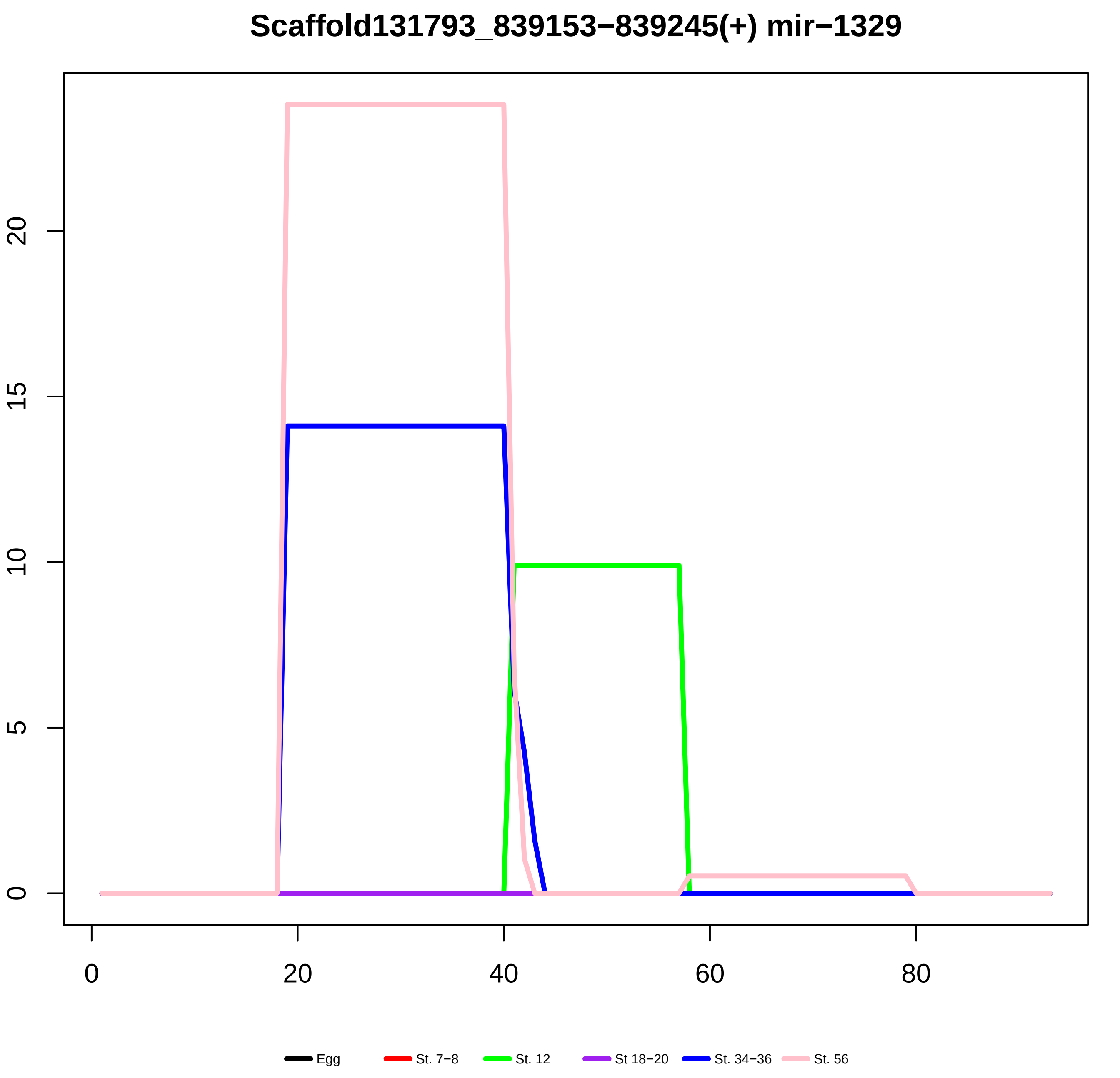

Scaffold131793_839153−839245(+) mir−1329
0
5
10
15
20
0
20
40
60
80
Egg
St. 7−8
St. 12
St 18−20
St. 34−36
St. 56

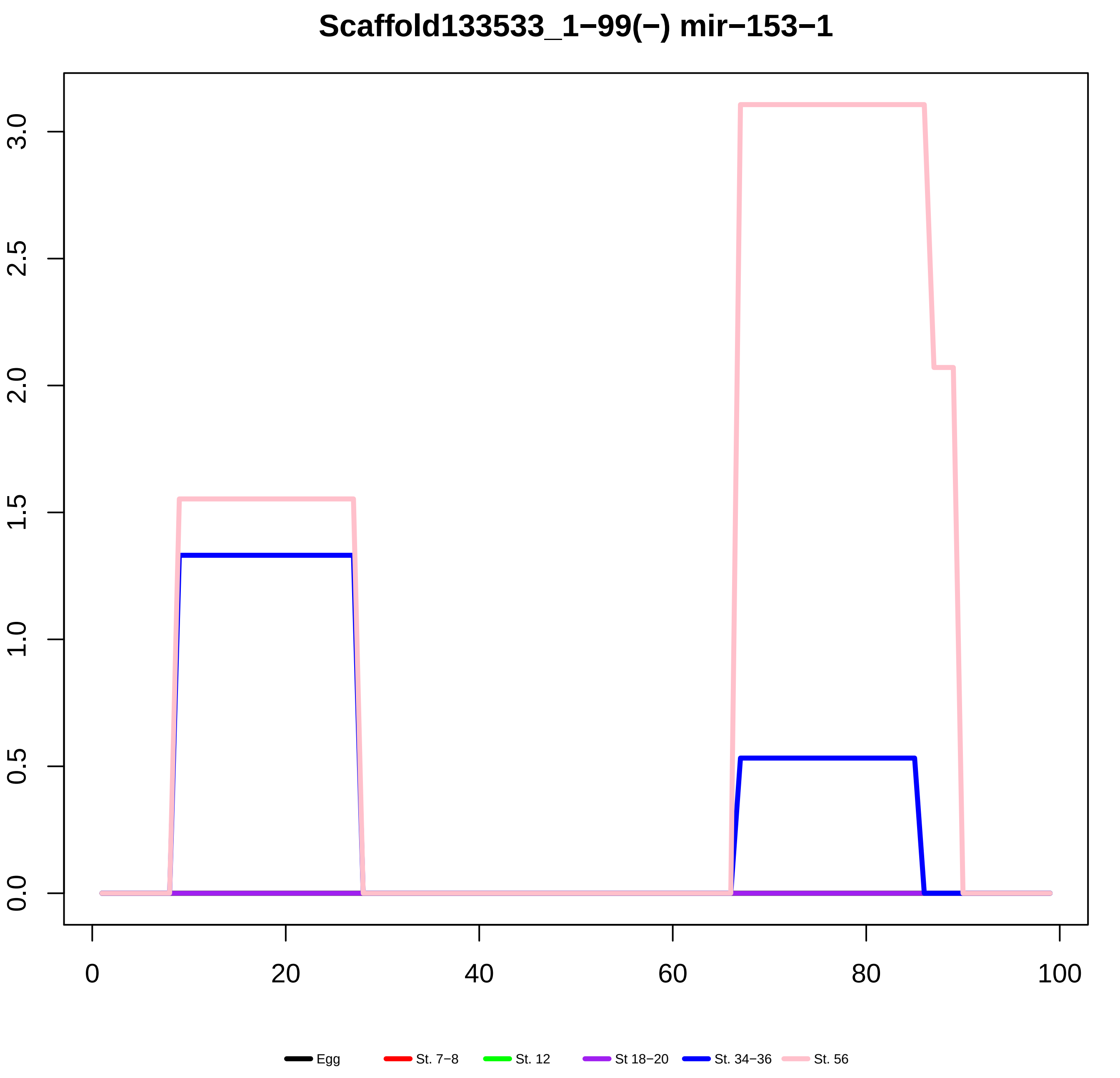

Scaffold133533_1−99(−) mir−153−1
0.0
0.5
1.0
1.5
2.0
2.5
3.0
0
20
40
60
80
100
Egg
St. 7−8
St. 12
St 18−20
St. 34−36
St. 56

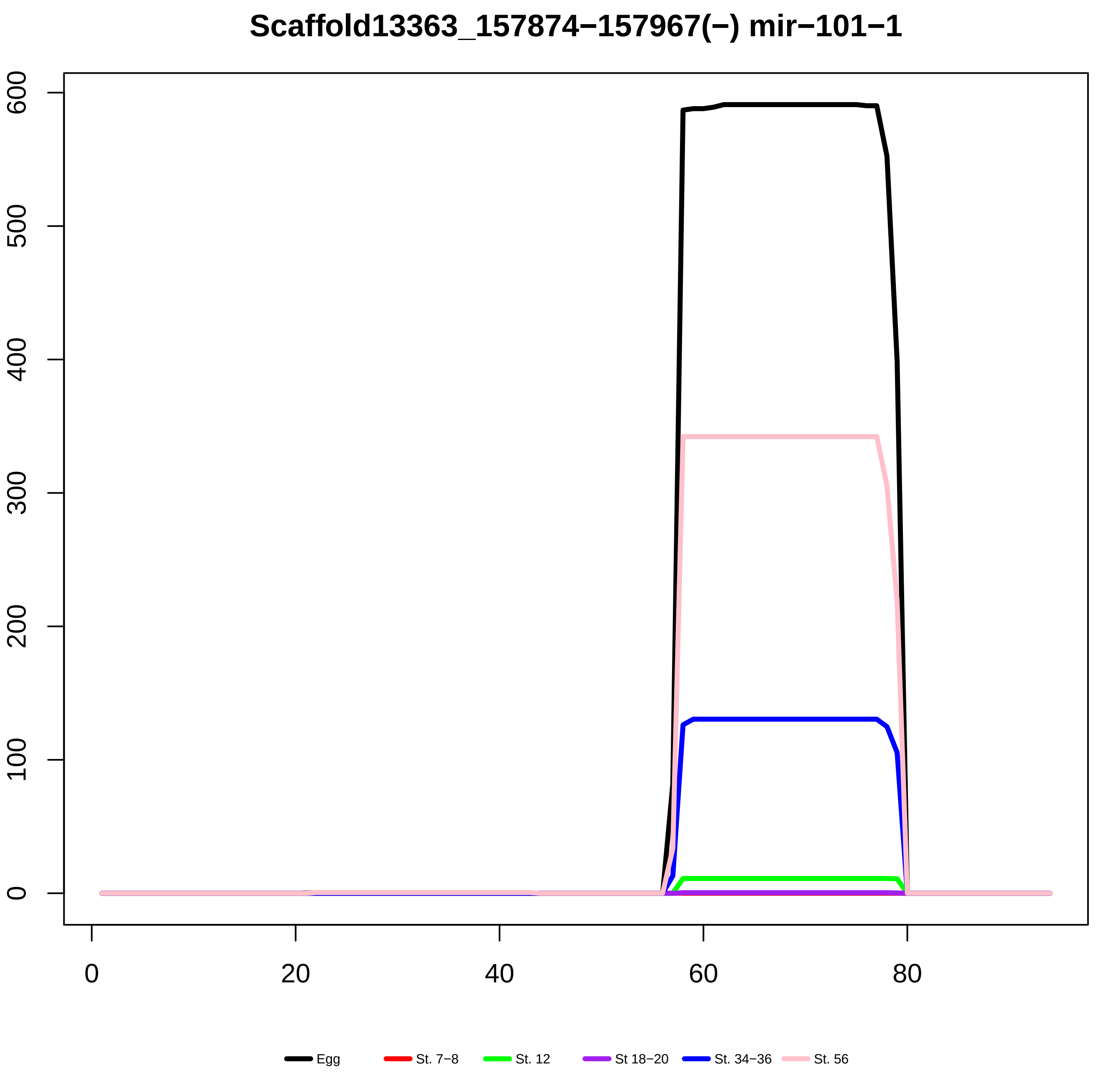
Scaffold13363_157874−157967(−) mir−101−1
0
100
200
300
400
500
600
0
20
40
60
80
Egg
St. 7−8
St. 12
St 18−20
St. 34−36
St. 56

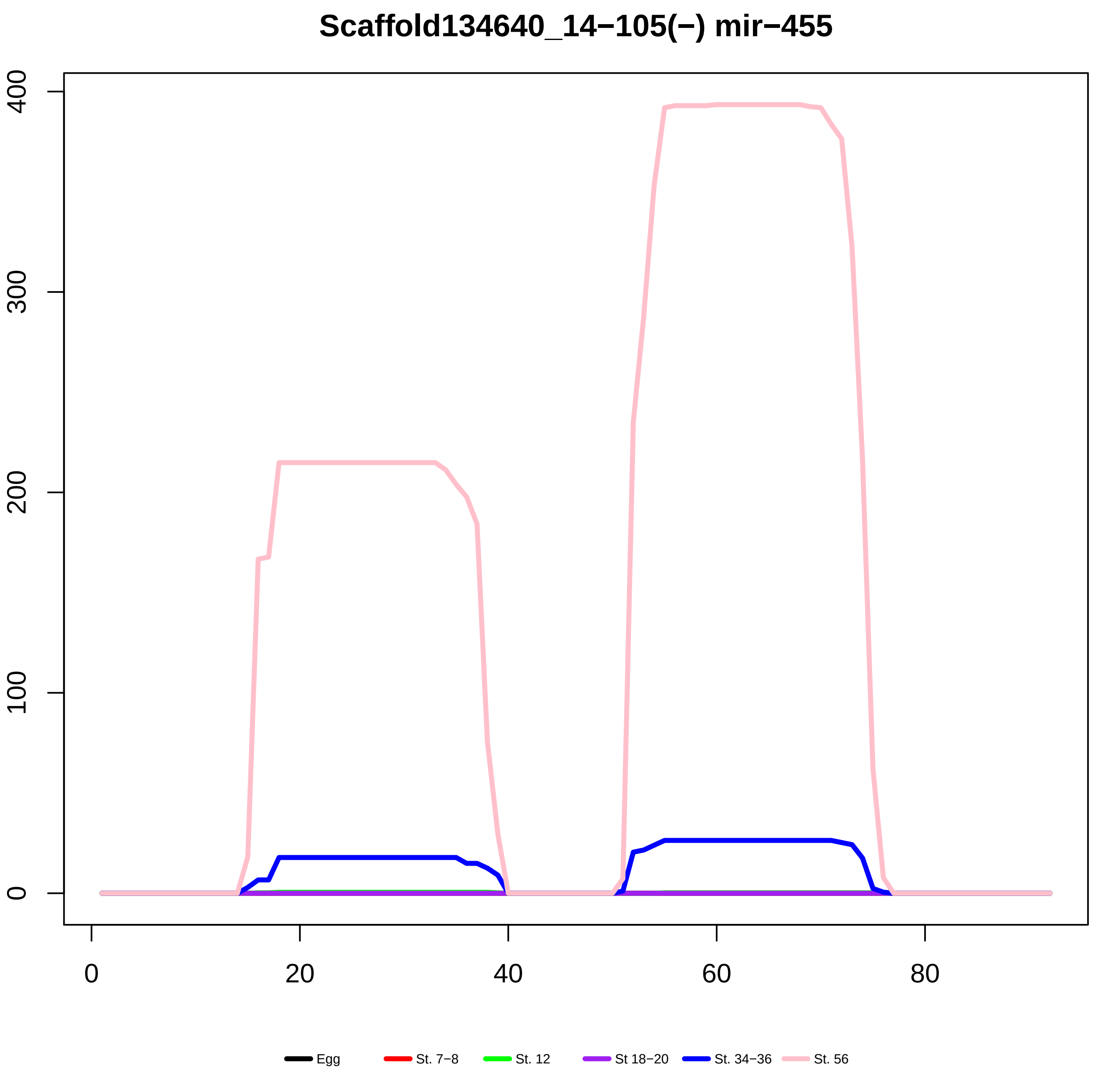
Scaffold134640_14−105(−) mir−455
0
100
200
300
400
0
20
40
60
80
Egg
St. 7−8
St. 12
St 18−20
St. 34−36
St. 56

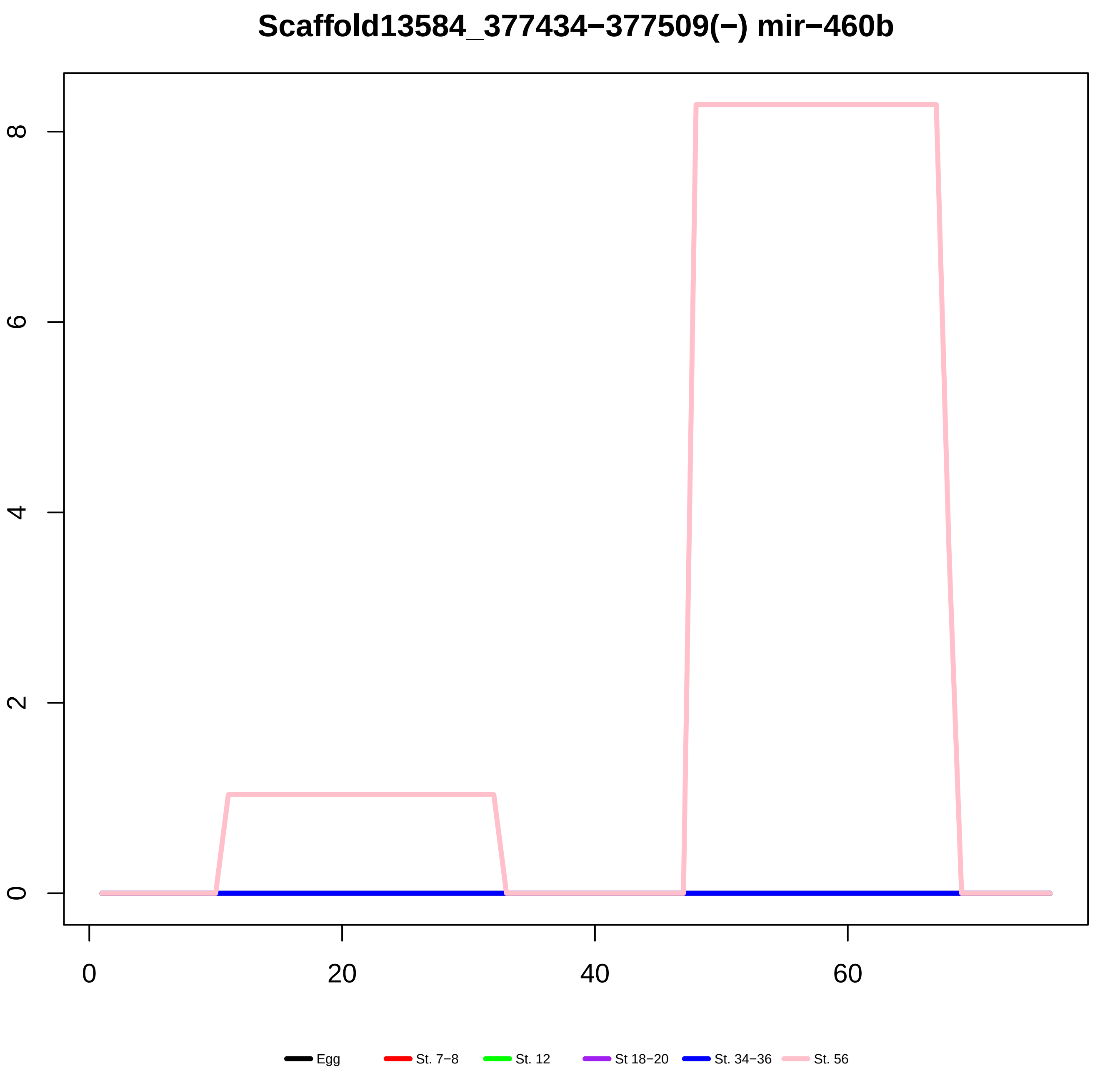
Scaffold13584_377434−377509(−) mir−460b
0
2
4
6
8
0
20
40
60
Egg
St. 7−8
St. 12
St 18−20
St. 34−36
St. 56

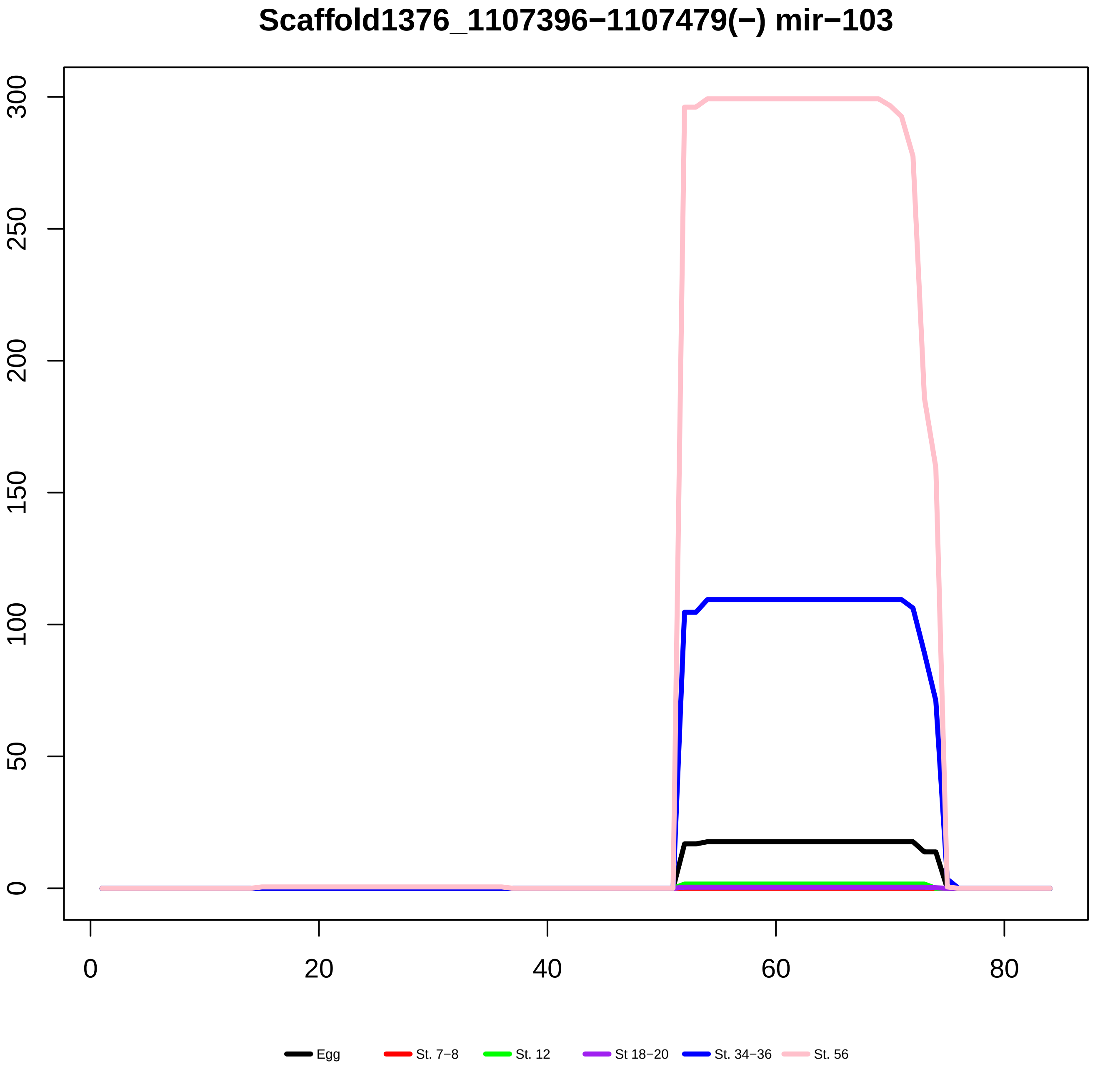

Scaffold1376_1107396−1107479(−) mir−103
0
50
100
150
200
250
300
0
20
40
60
80
Egg
St. 7−8
St. 12
St 18−20
St. 34−36
St. 56

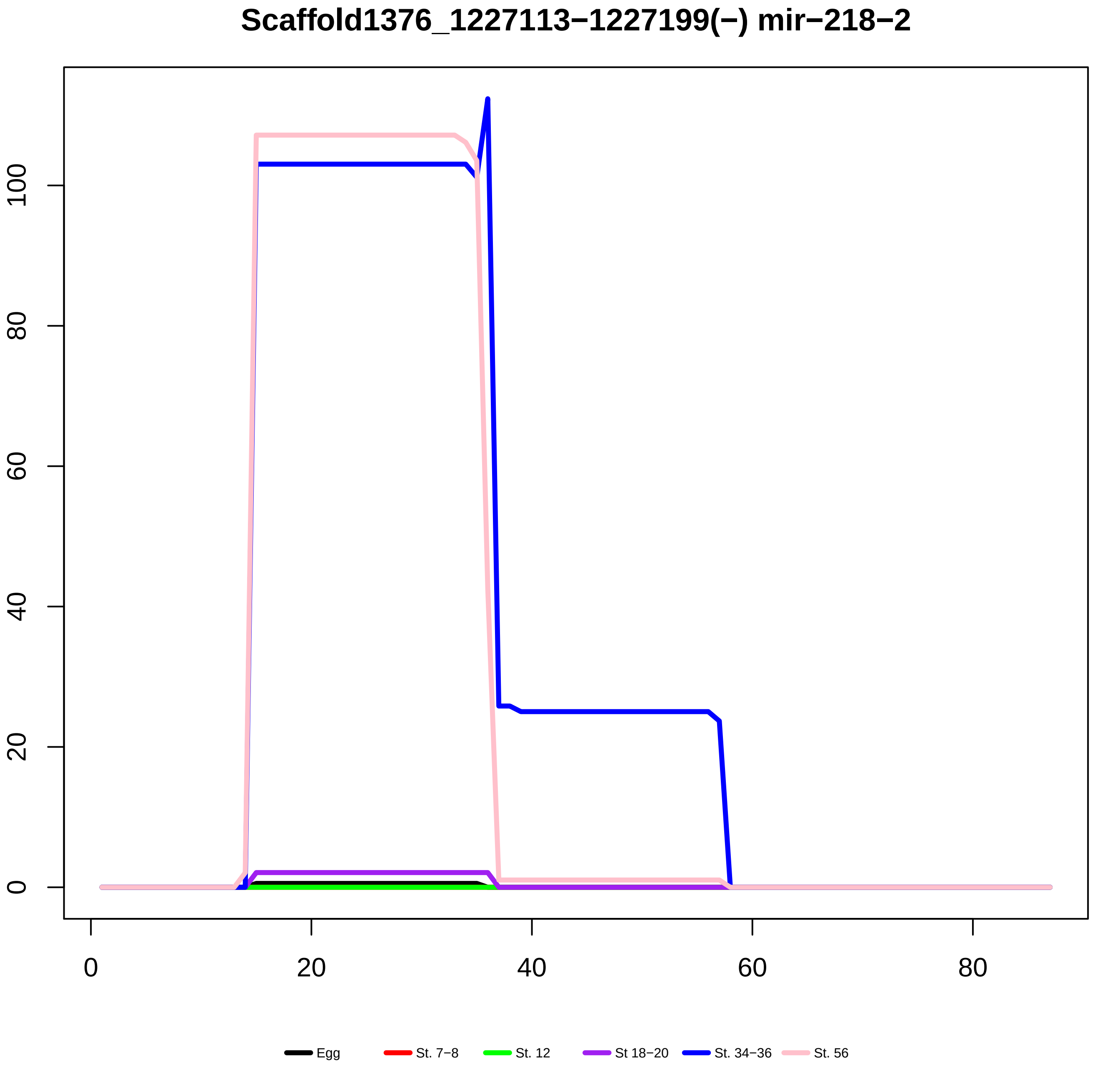
Scaffold1376_1227113−1227199(−) mir−218−2
0
20
40
60
80
100
0
20
40
60
80
Egg
St. 7−8
St. 12
St 18−20
St. 34−36
St. 56

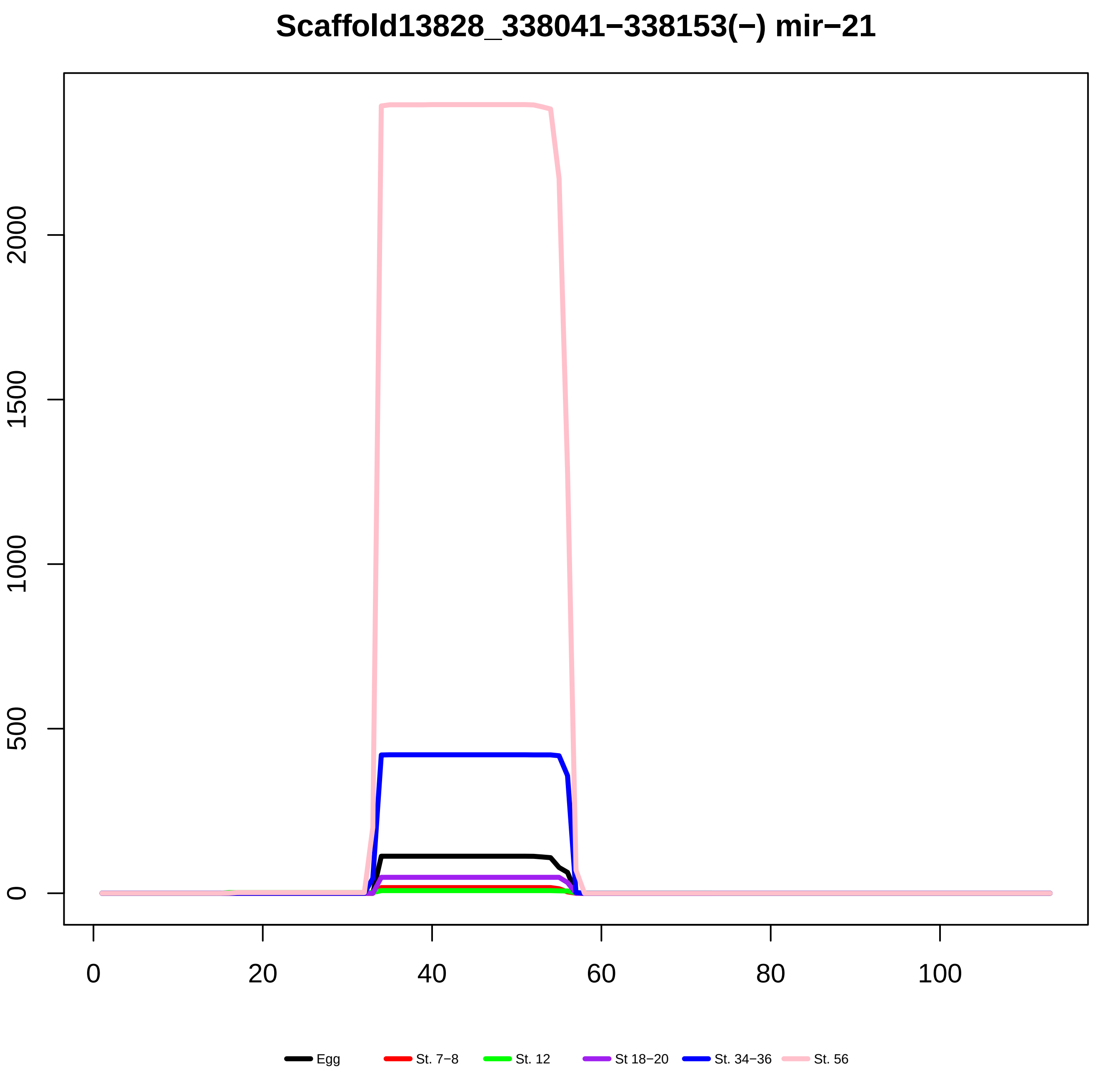

Scaffold13828_338041−338153(−) mir−21
0
500
1000
1500
2000
0
20
40
60
80
100
Egg
St. 7−8
St. 12
St 18−20
St. 34−36
St. 56

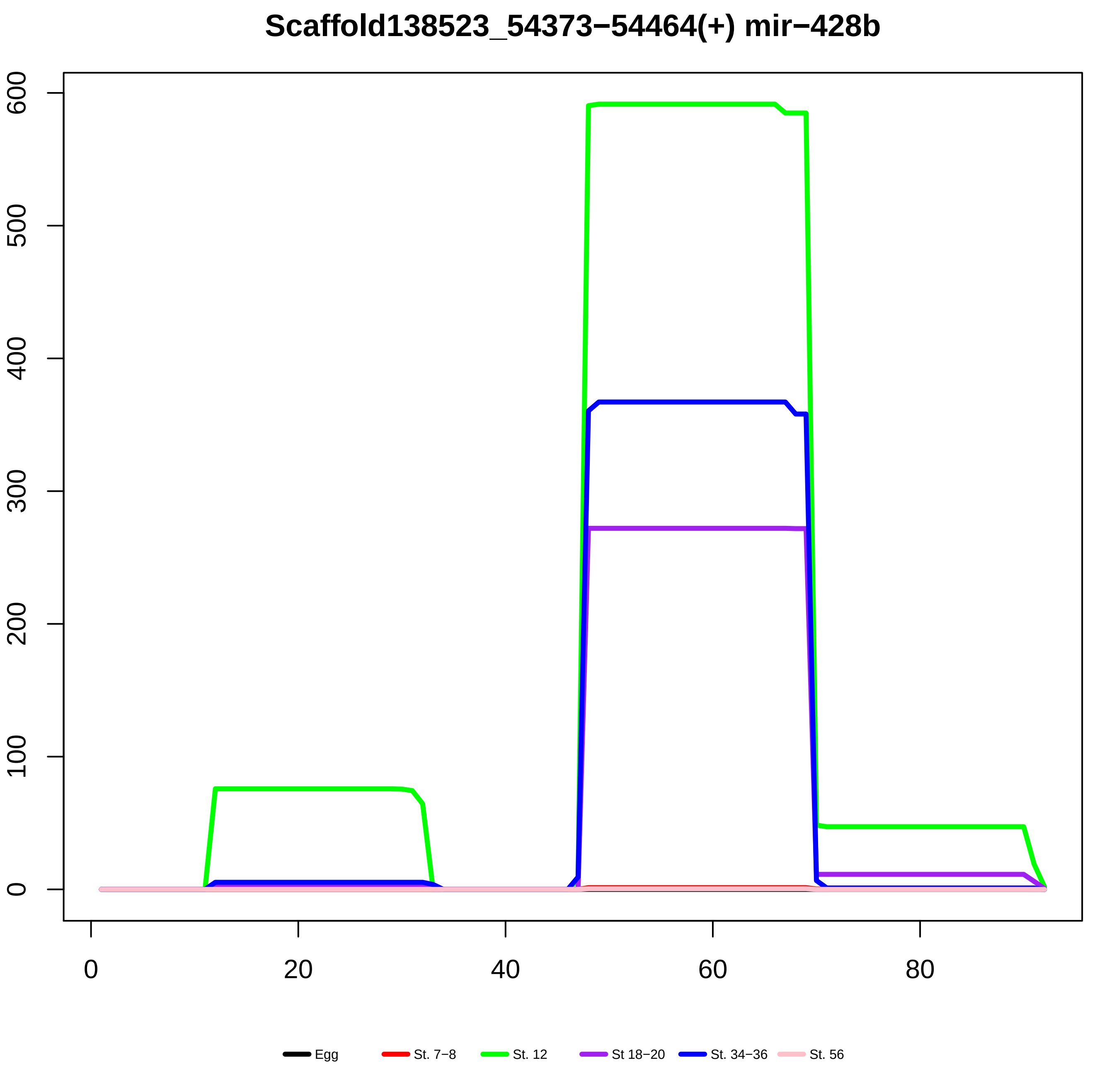

Scaffold138523_54373−54464(+) mir−428b
0
100
200
300
400
500
600
0
20
40
60
80
Egg
St. 7−8
St. 12
St 18−20
St. 34−36
St. 56

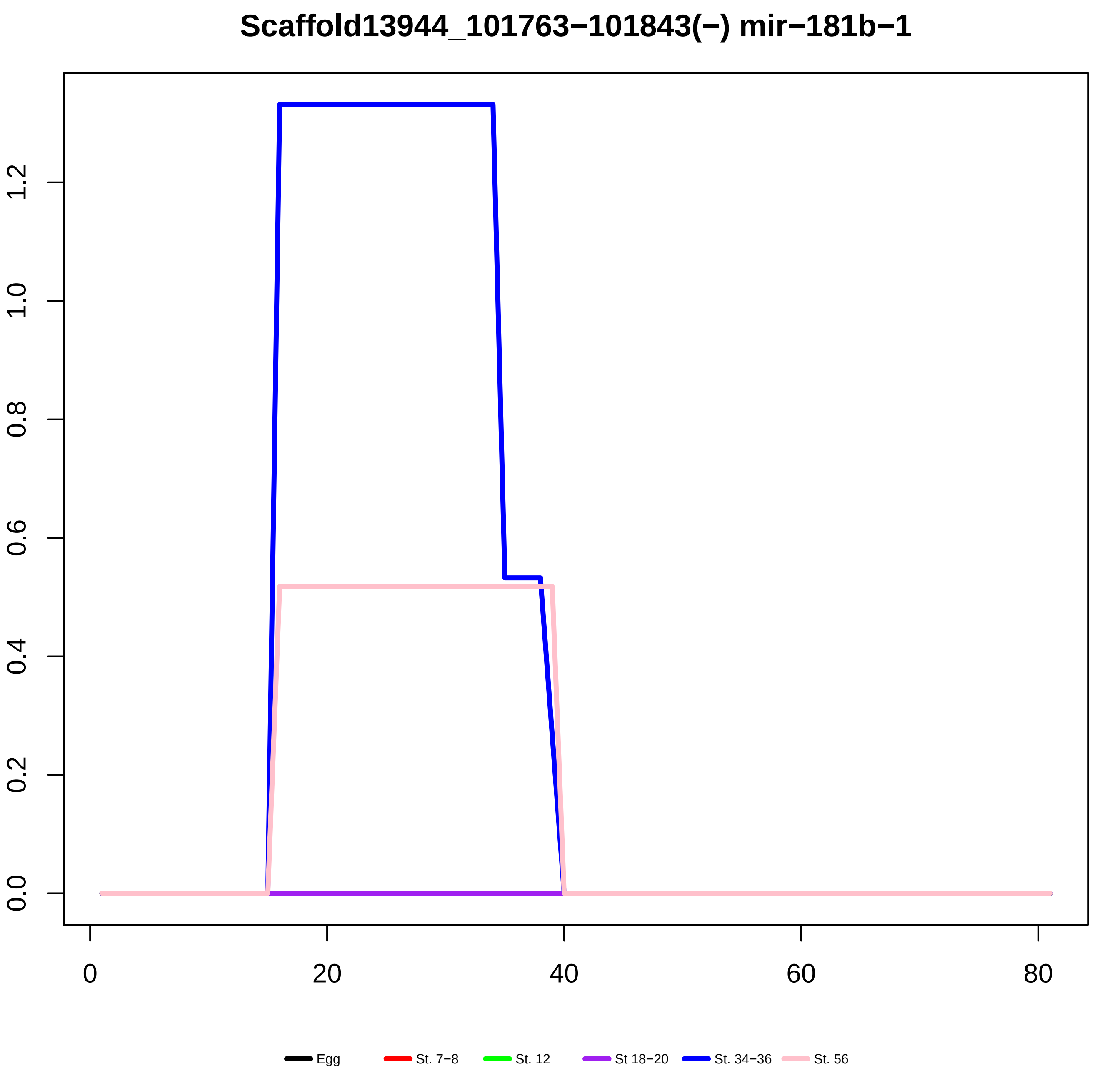
Scaffold13944_101763−101843(−) mir−181b−1
0.0
0.2
0.4
0.6
0.8
1.0
1.2
0
20
40
60
80
Egg
St. 7−8
St. 12
St 18−20
St. 34−36
St. 56

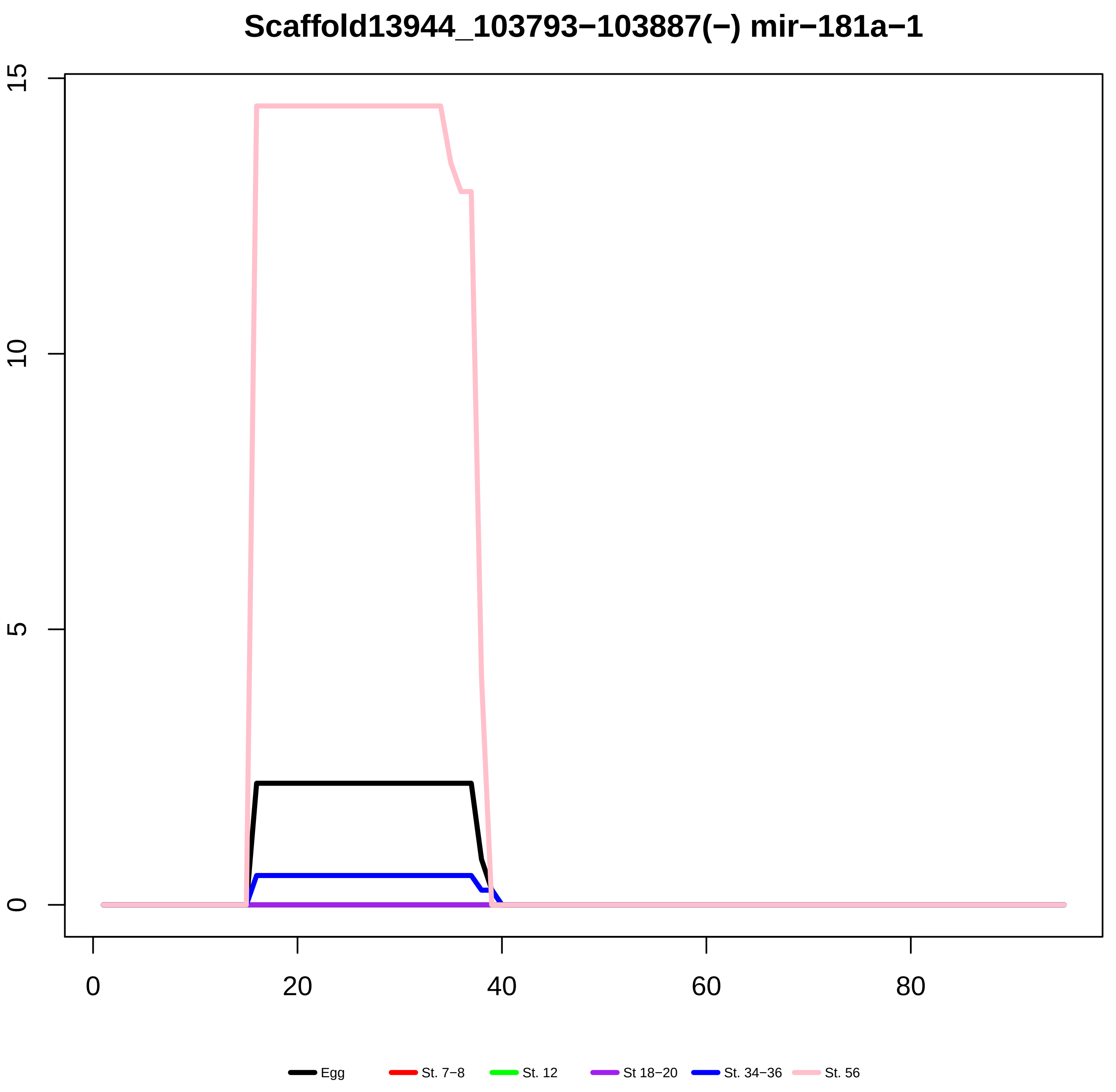
Scaffold13944_103793−103887(−) mir−181a−1
0
5
10
15
0
20
40
60
80
Egg
St. 7−8
St. 12
St 18−20
St. 34−36
St. 56

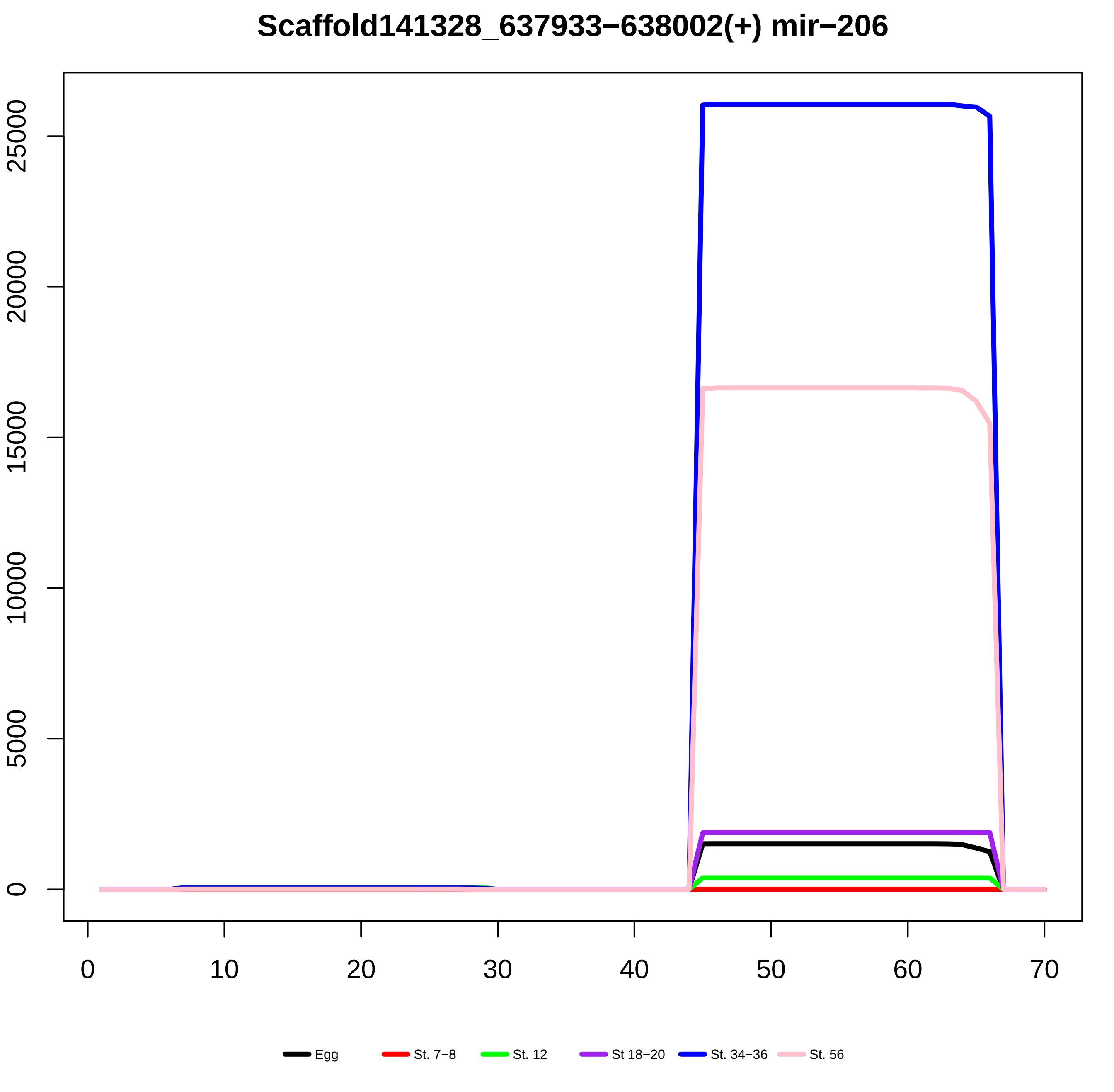

Scaffold141328_637933−638002(+) mir−206
0
5000
10000
15000
20000
25000
0
10
20
30
40
50
60
70
Egg
St. 7−8
St. 12
St 18−20
St. 34−36
St. 56

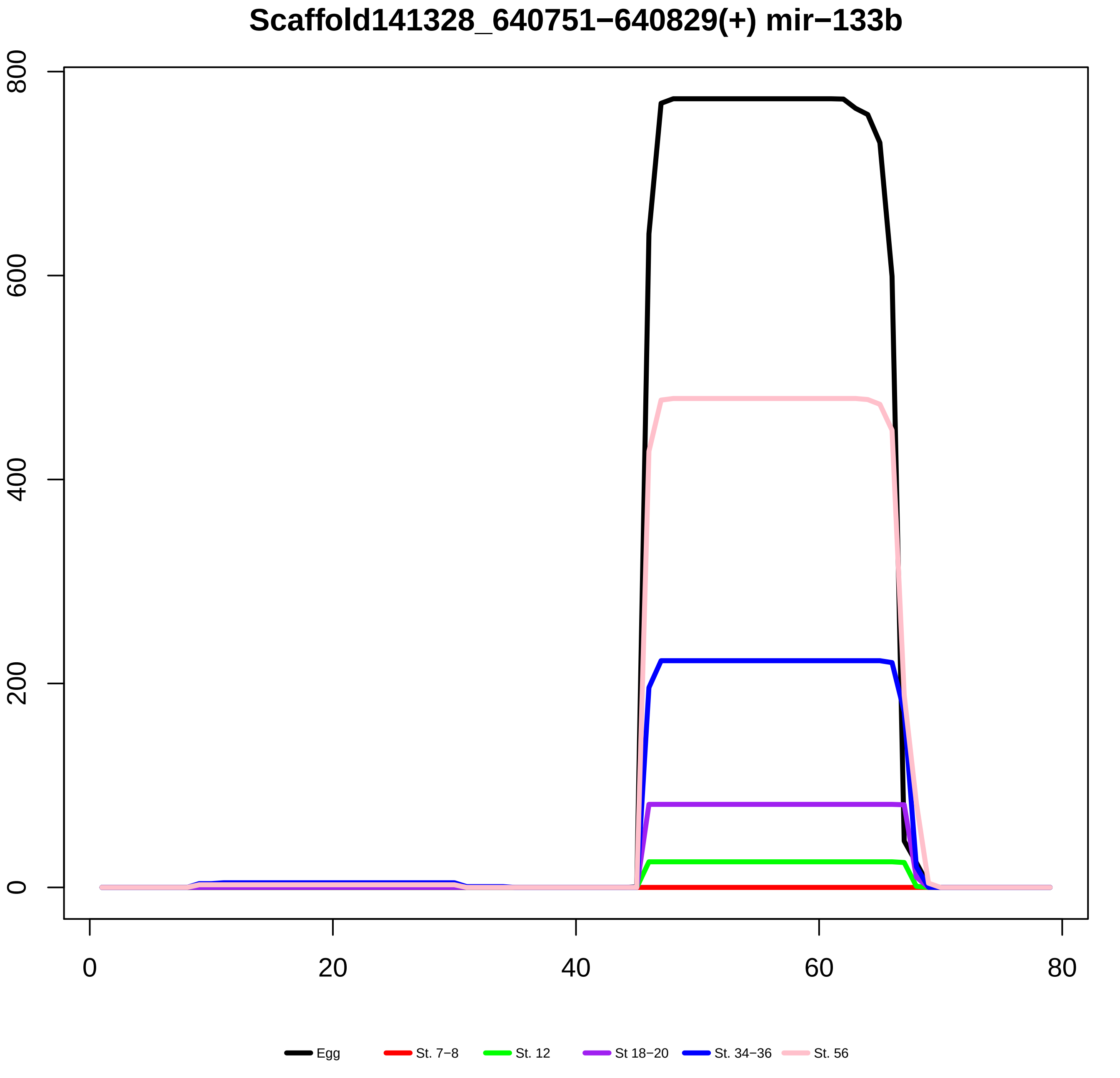
Scaffold141328_640751−640829(+) mir−133b
0
200
400
600
800
0
20
40
60
80
Egg
St. 7−8
St. 12
St 18−20
St. 34−36
St. 56

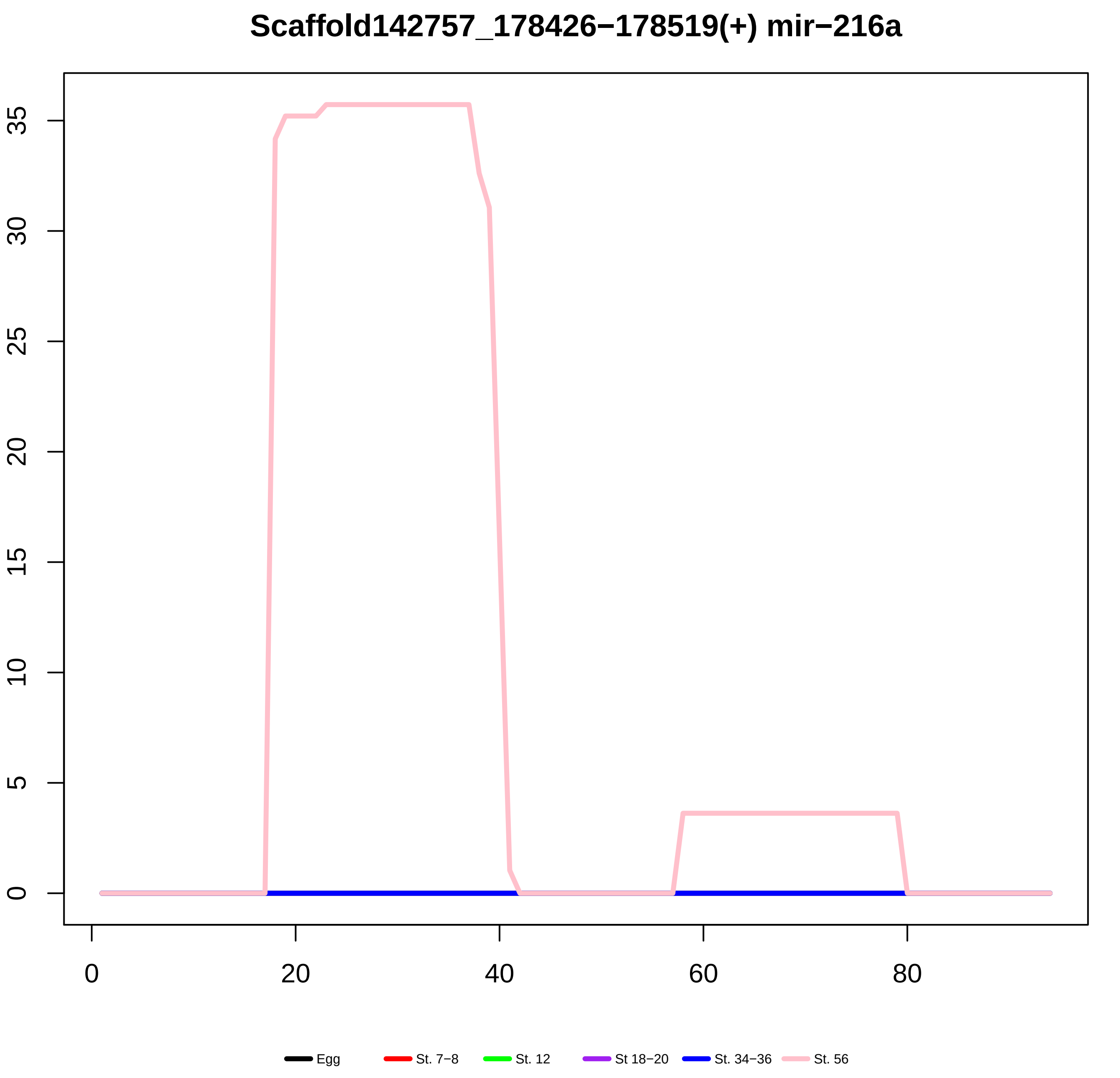

Scaffold142757_178426−178519(+) mir−216a
0
5
10
15
20
25
30
35
0
20
40
60
80
Egg
St. 7−8
St. 12
St 18−20
St. 34−36
St. 56

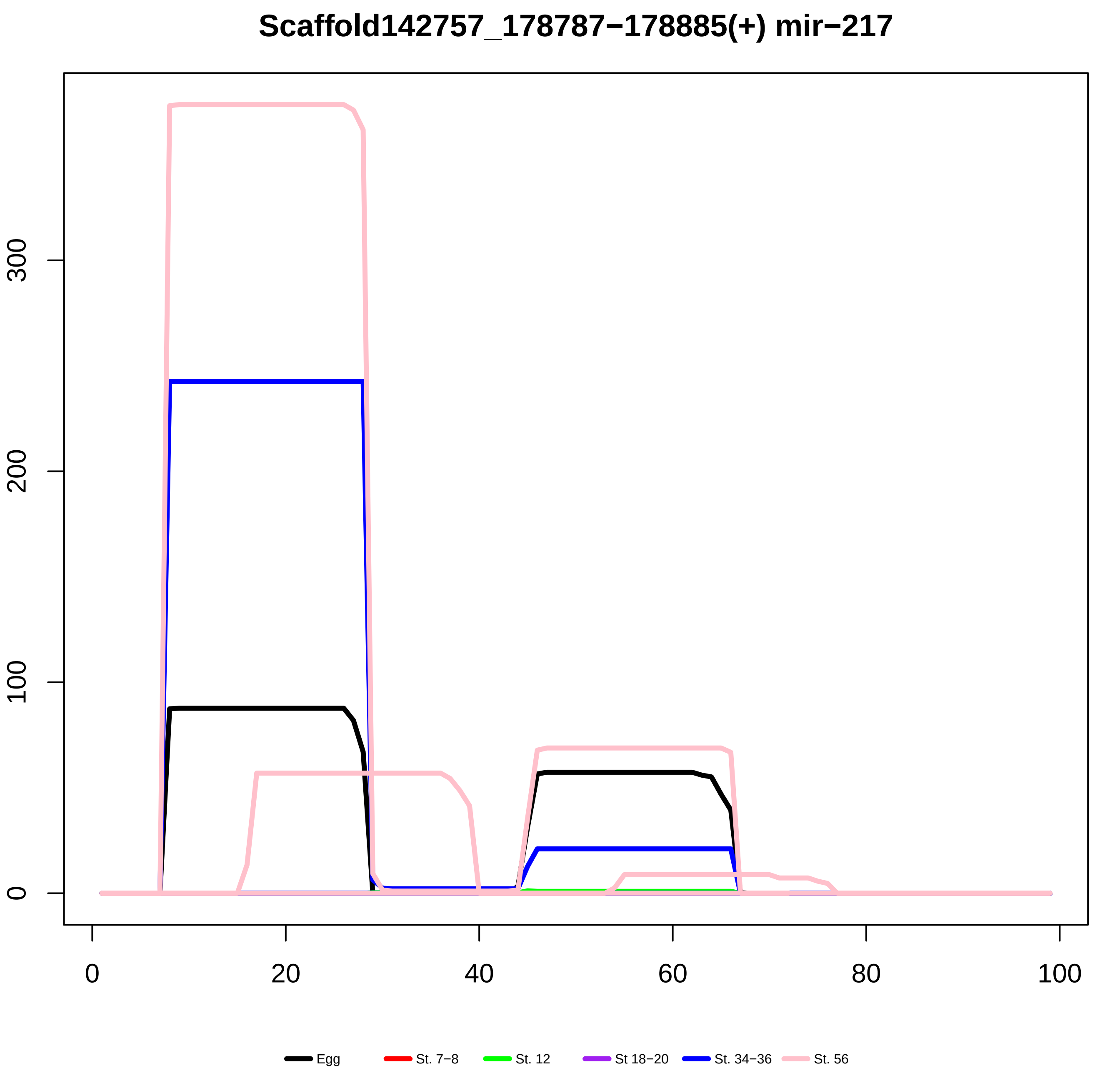

Scaffold142757_178787−178885(+) mir−217
0
100
200
300
0
20
40
60
80
100
Egg
St. 7−8
St. 12
St 18−20
St. 34−36
St. 56

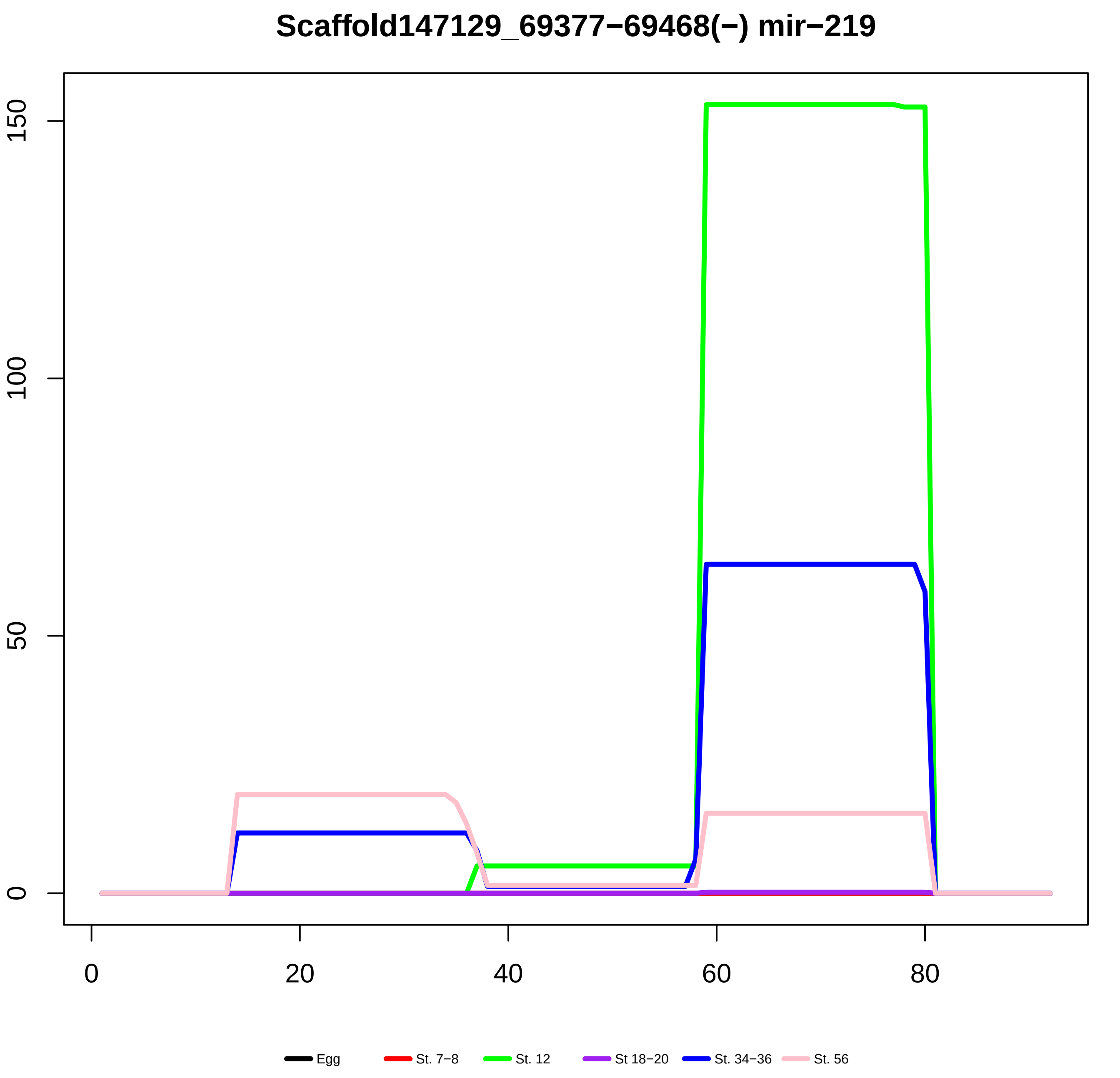

Scaffold147129_69377−69468(−) mir−219
150
100
50
0
0
20
40
60
80
Egg
St. 7−8
St. 12
St 18−20
St. 34−36
St. 56

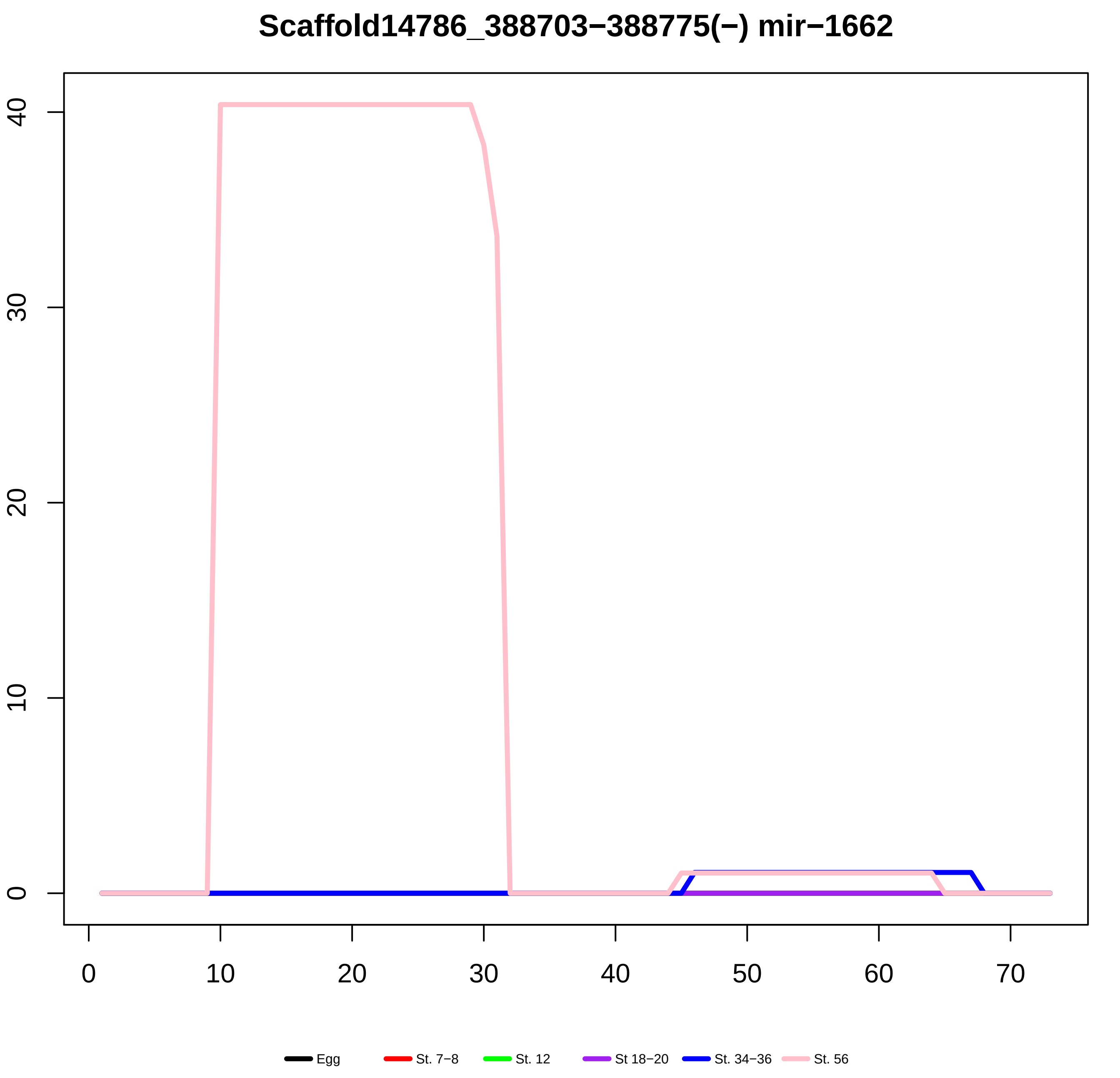

Scaffold14786_388703−388775(−) mir−1662
0
10
20
30
40
0
10
20
30
40
50
60
70
Egg
St. 7−8
St. 12
St 18−20
St. 34−36
St. 56

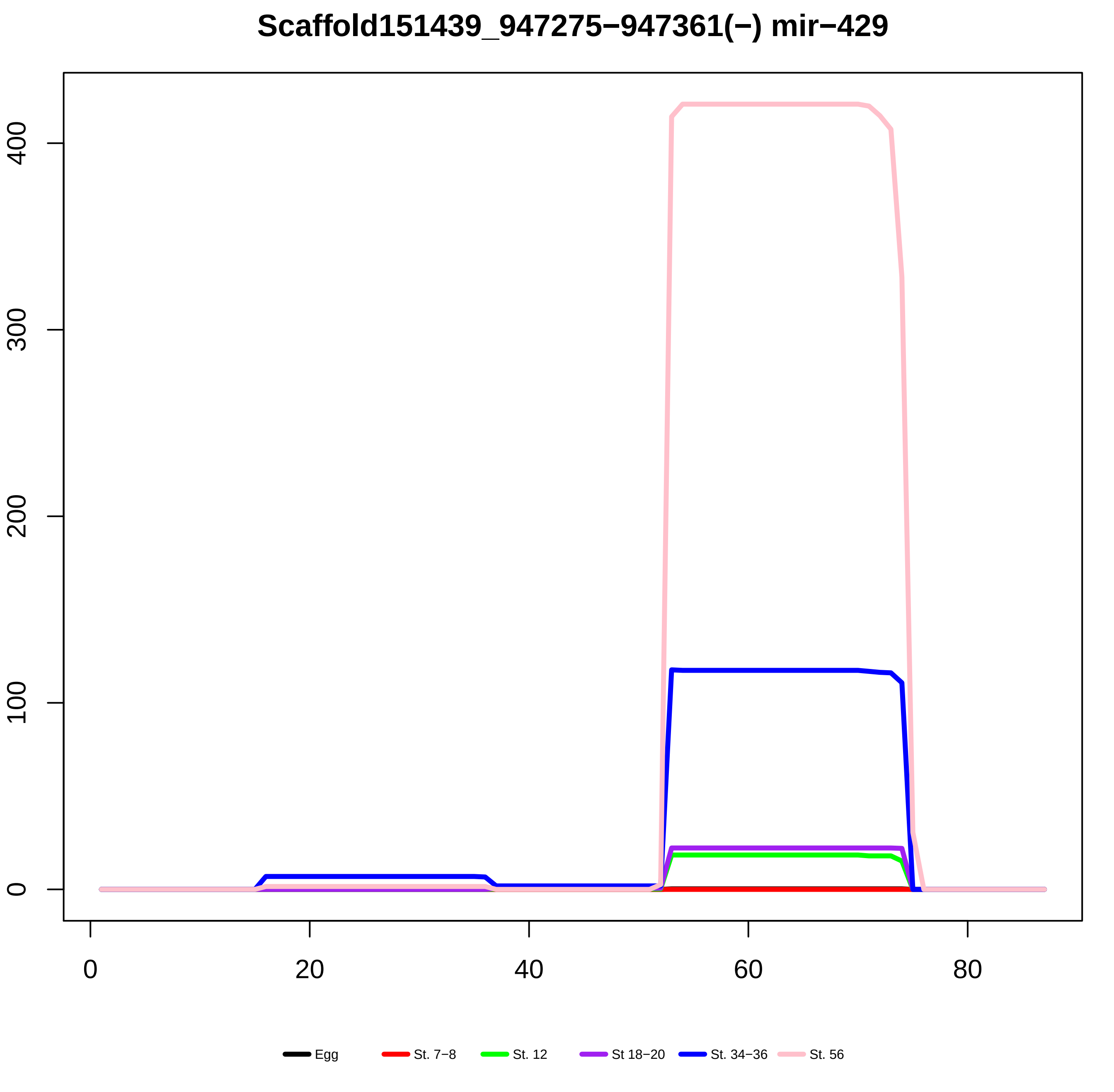
Scaffold151439_947275−947361(−) mir−429
0
100
200
300
400
0
20
40
60
80
Egg
St. 7−8
St. 12
St 18−20
St. 34−36
St. 56

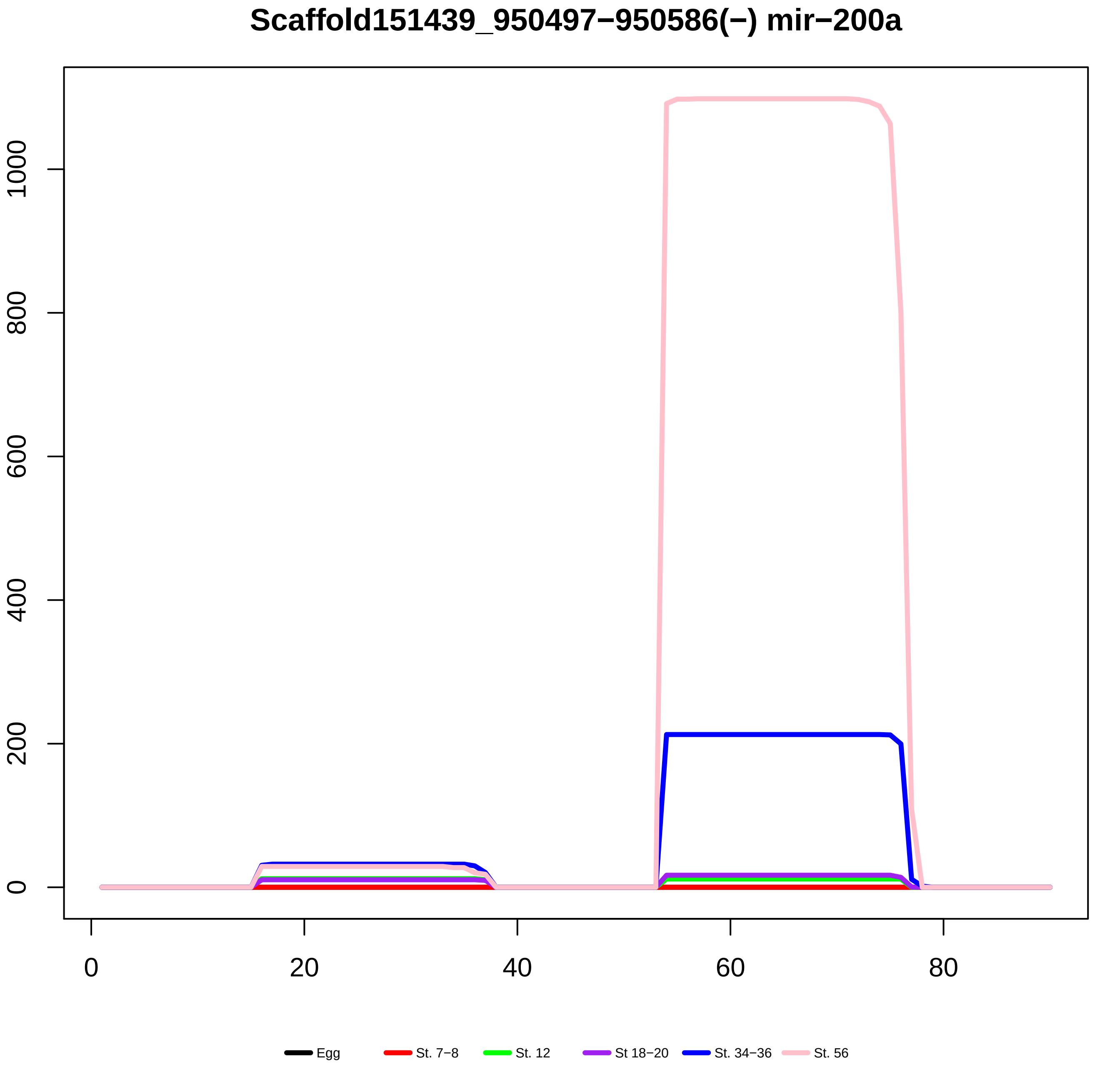

Scaffold151439_950497−950586(−) mir−200a
0
200
400
600
800
1000
0
20
40
60
80
Egg
St. 7−8
St. 12
St 18−20
St. 34−36
St. 56

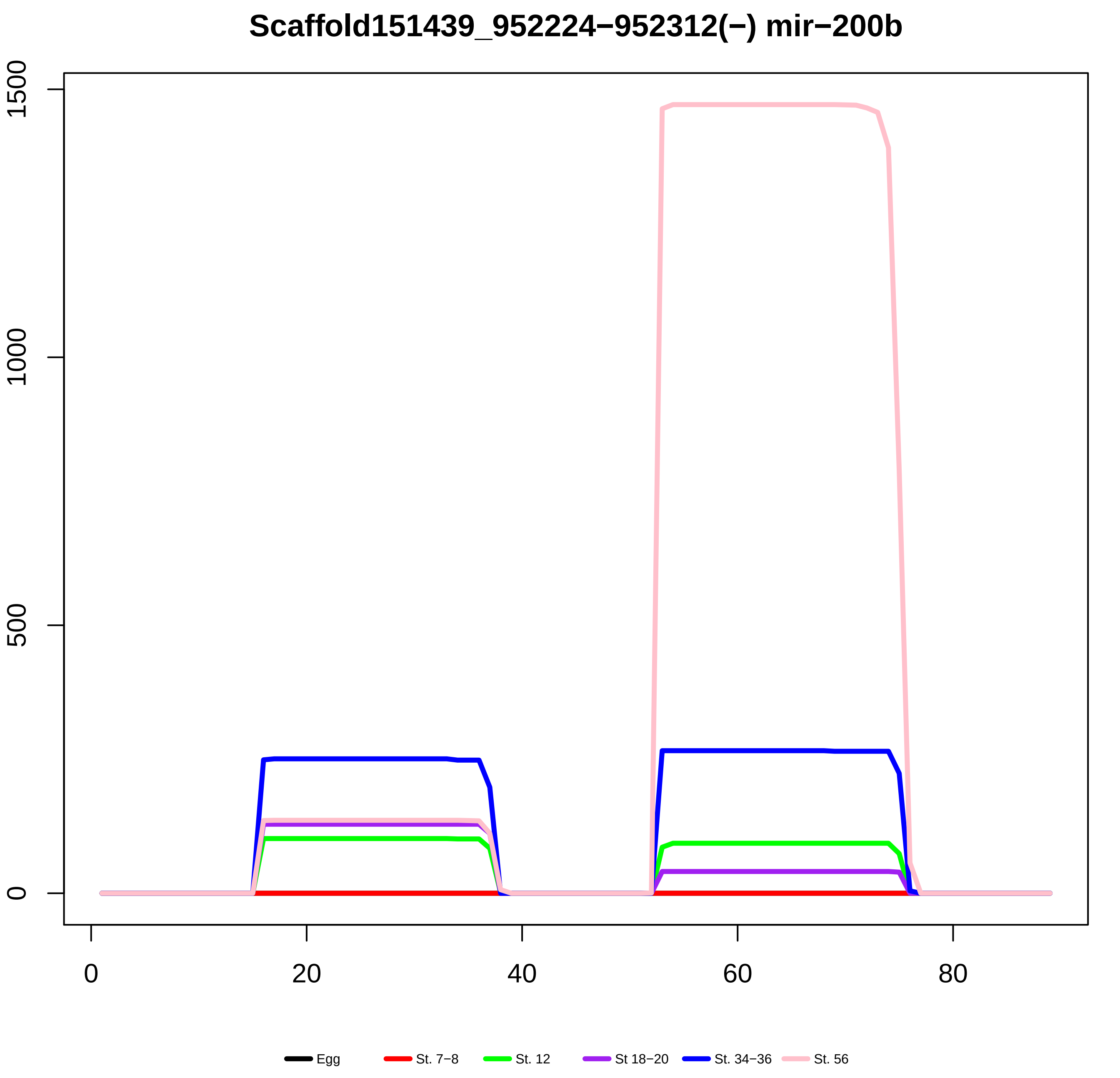
Scaffold151439_952224−952312(−) mir−200b
1500
1000
500
0
0
20
40
60
80
Egg
St. 7−8
St. 12
St 18−20
St. 34−36
St. 56

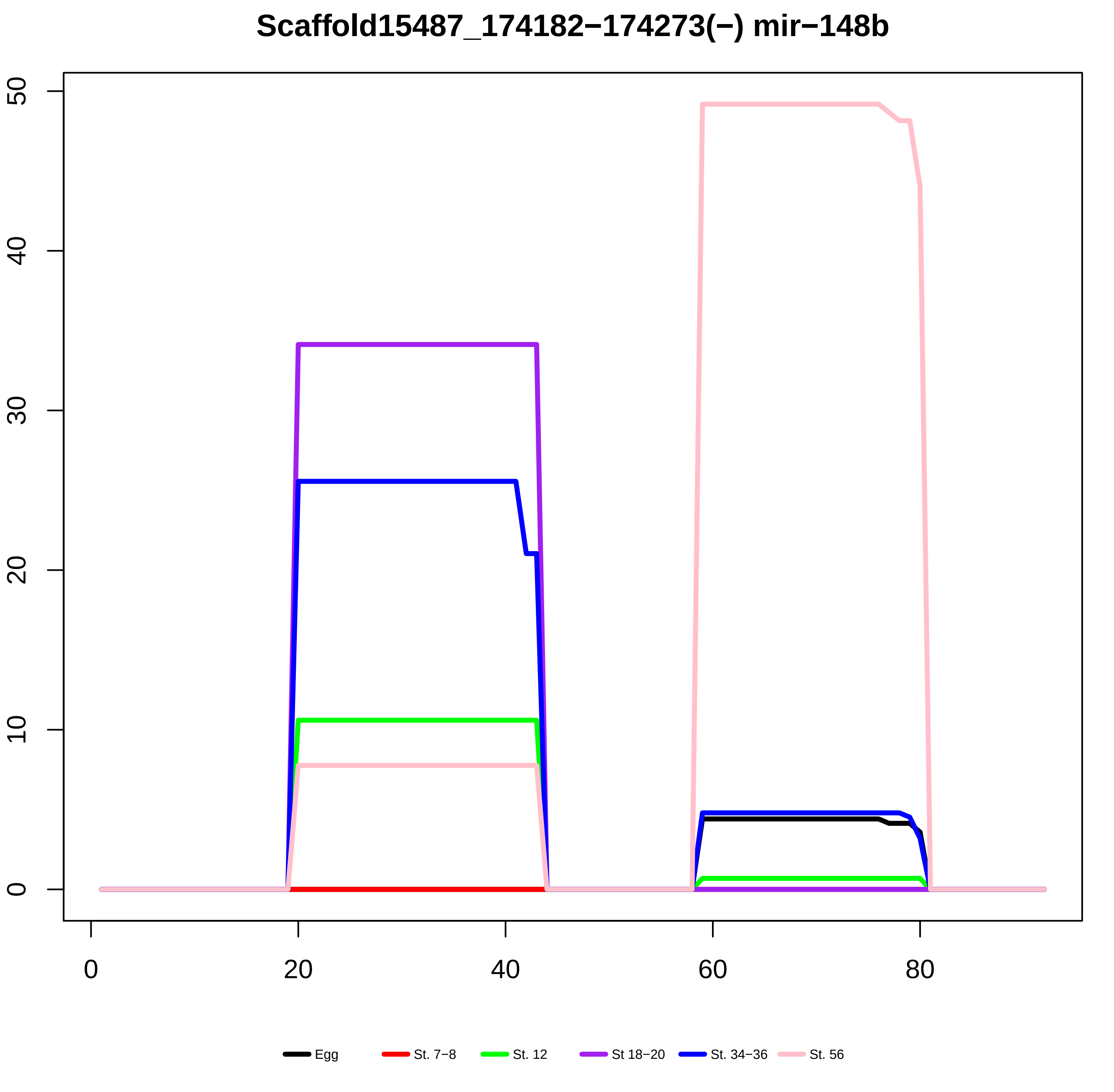
Scaffold15487_174182−174273(−) mir−148b
0
10
20
30
40
50
0
20
40
60
80
Egg
St. 7−8
St. 12
St 18−20
St. 34−36
St. 56

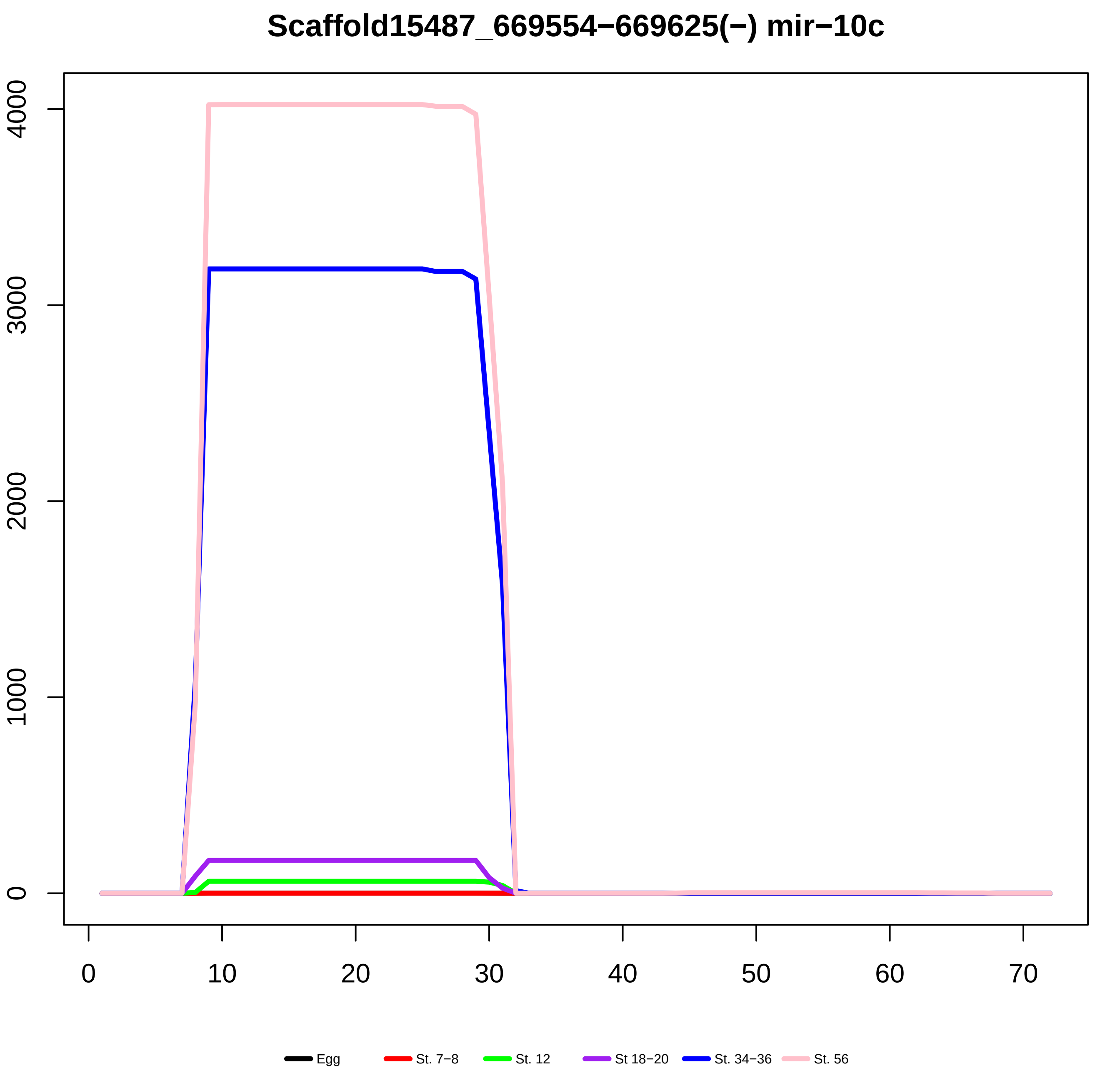
Scaffold15487_669554−669625(−) mir−10c
0
1000
2000
3000
4000
0
10
20
30
40
50
60
70
Egg
St. 7−8
St. 12
St 18−20
St. 34−36
St. 56

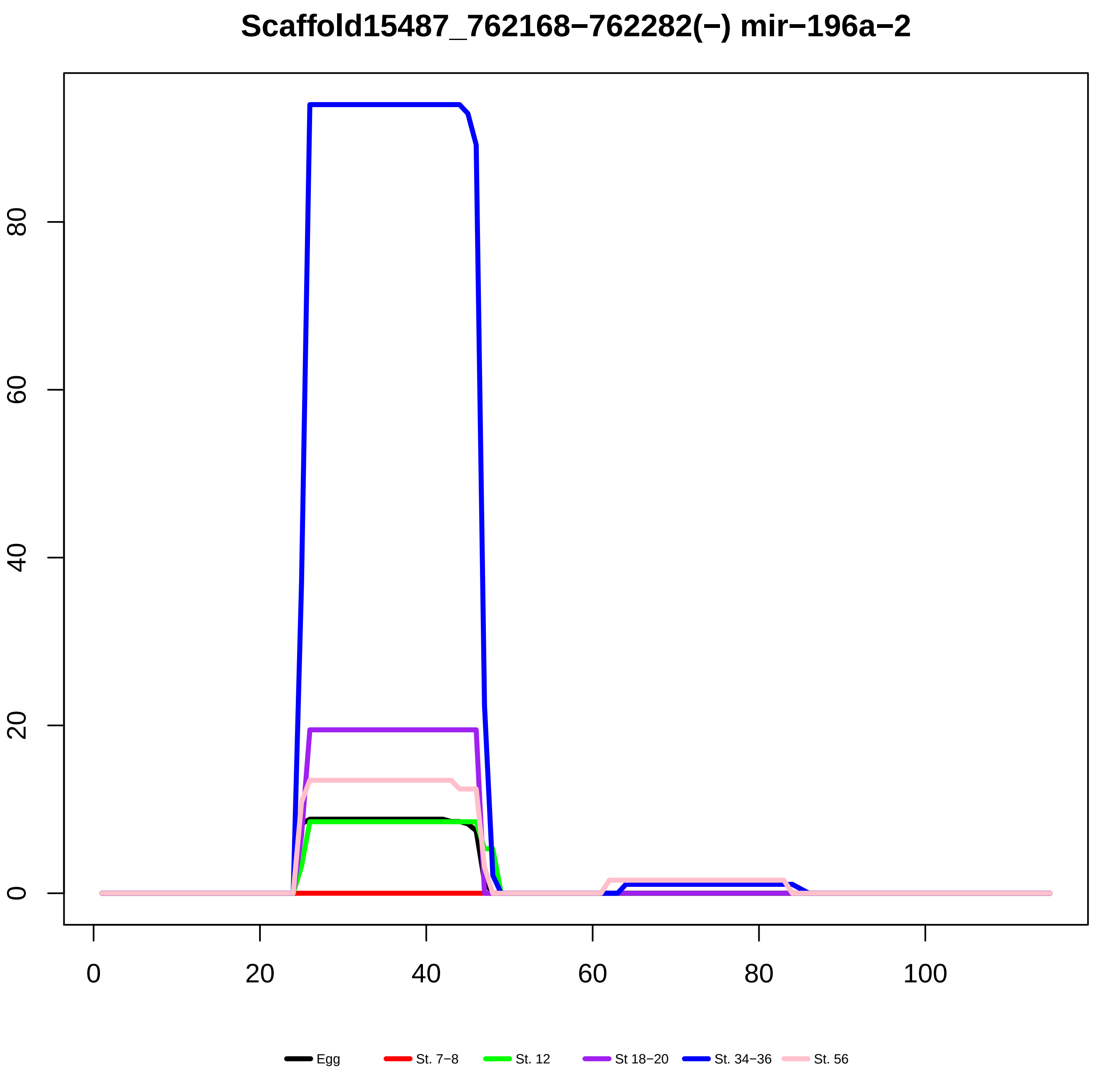

Scaffold15487_762168−762282(−) mir−196a−2
0
20
40
60
80
0
20
40
60
80
100
Egg
St. 7−8
St. 12
St 18−20
St. 34−36
St. 56

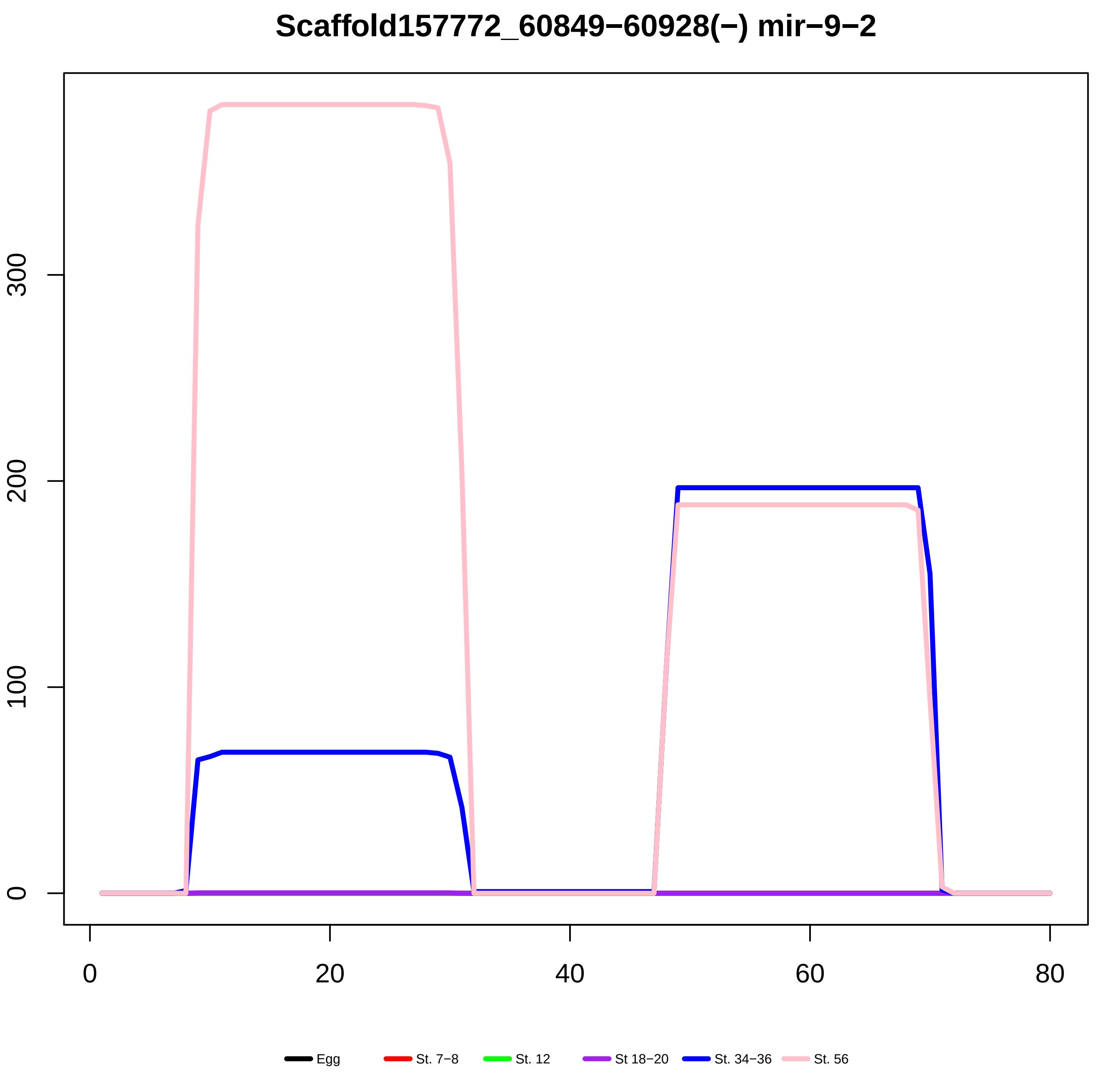

Scaffold157772_60849−60928(−) mir−9−2
0
100
200
300
0
20
40
60
80
Egg
St. 7−8
St. 12
St 18−20
St. 34−36
St. 56

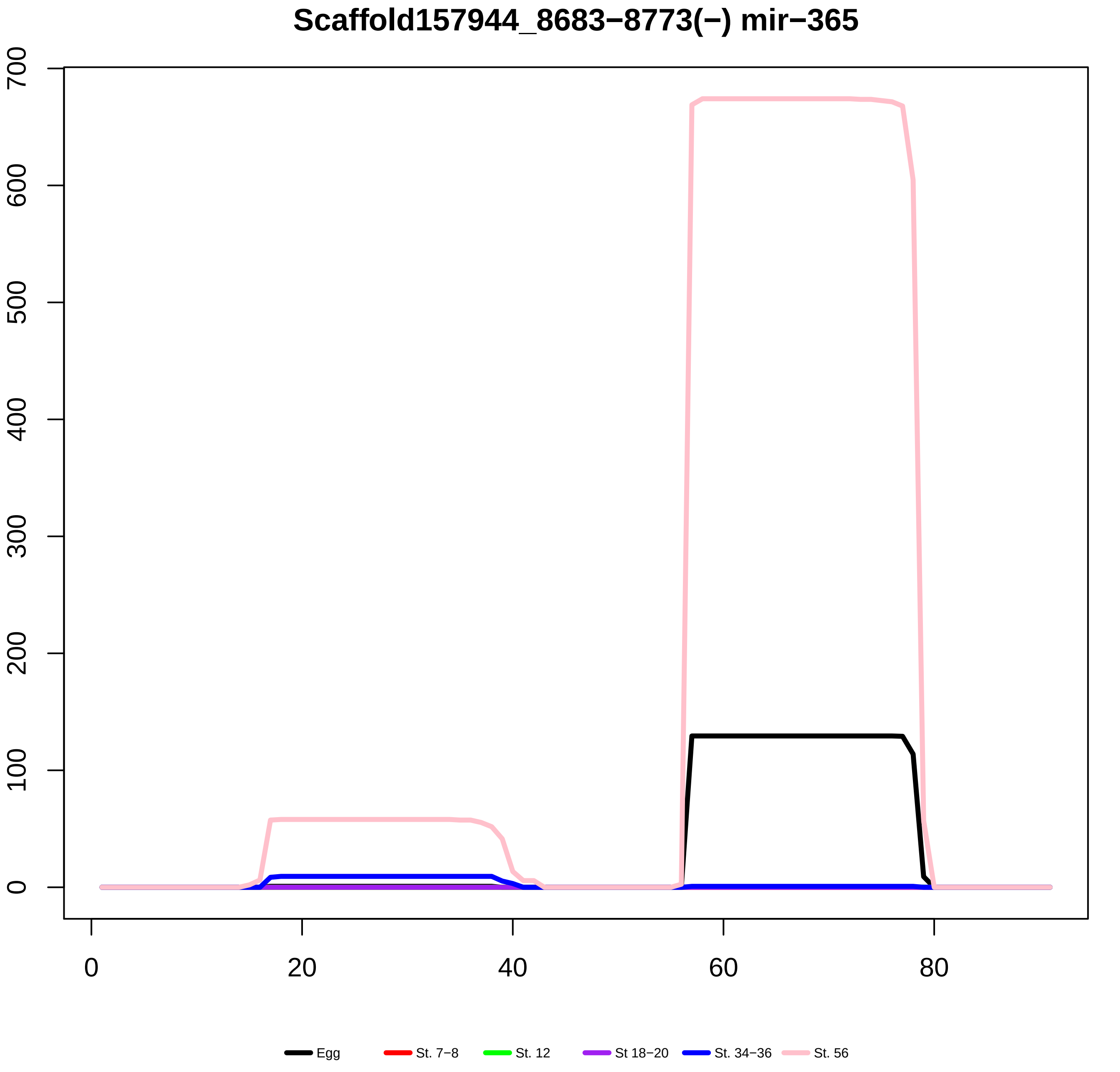
Scaffold157944_8683−8773(−) mir−365
0
100
200
300
400
500
600
700
0
20
40
60
80
Egg
St. 7−8
St. 12
St 18−20
St. 34−36
St. 56

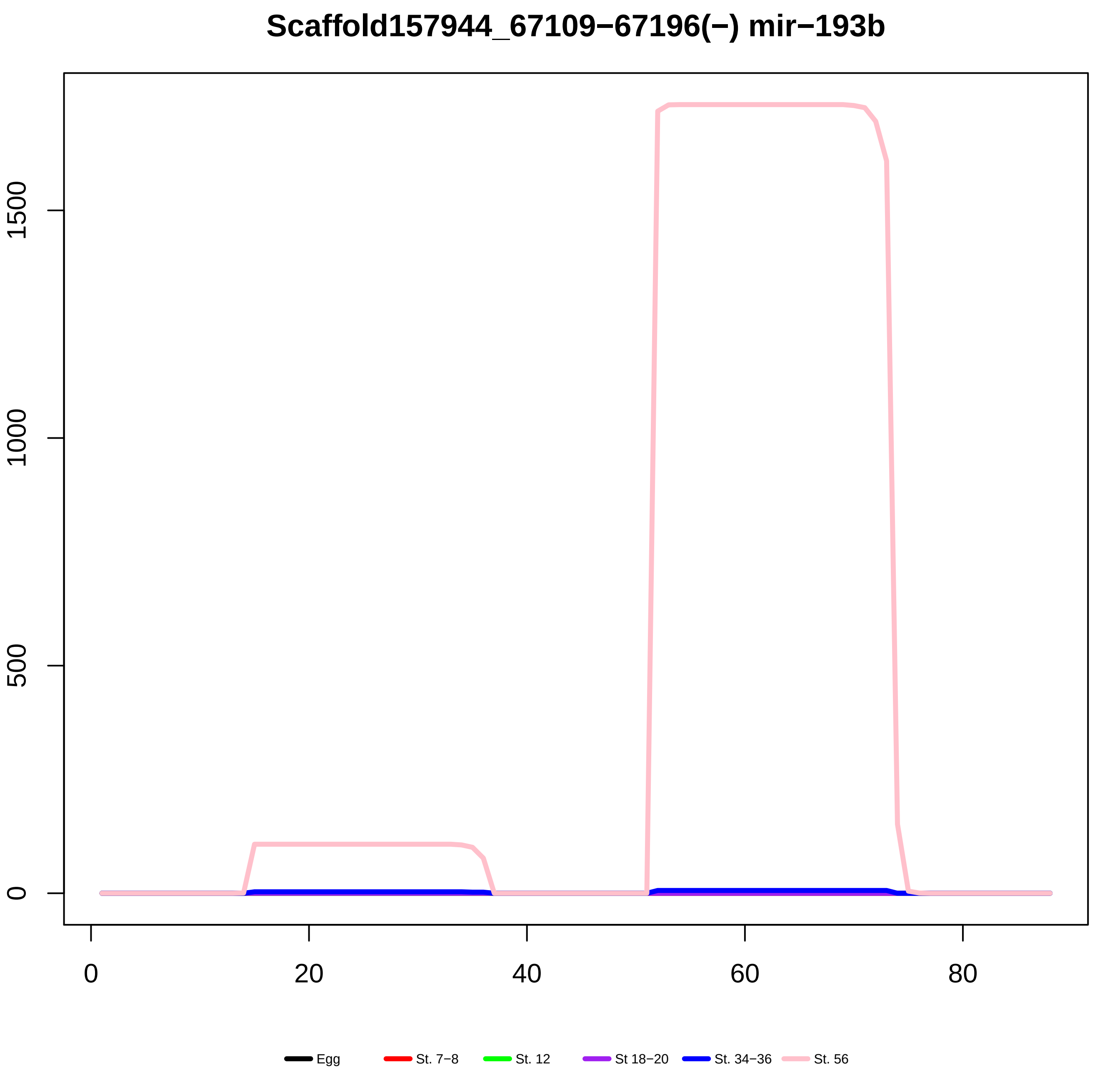

Scaffold157944_67109−67196(−) mir−193b
0
500
1000
1500
0
20
40
60
80
Egg
St. 7−8
St. 12
St 18−20
St. 34−36
St. 56

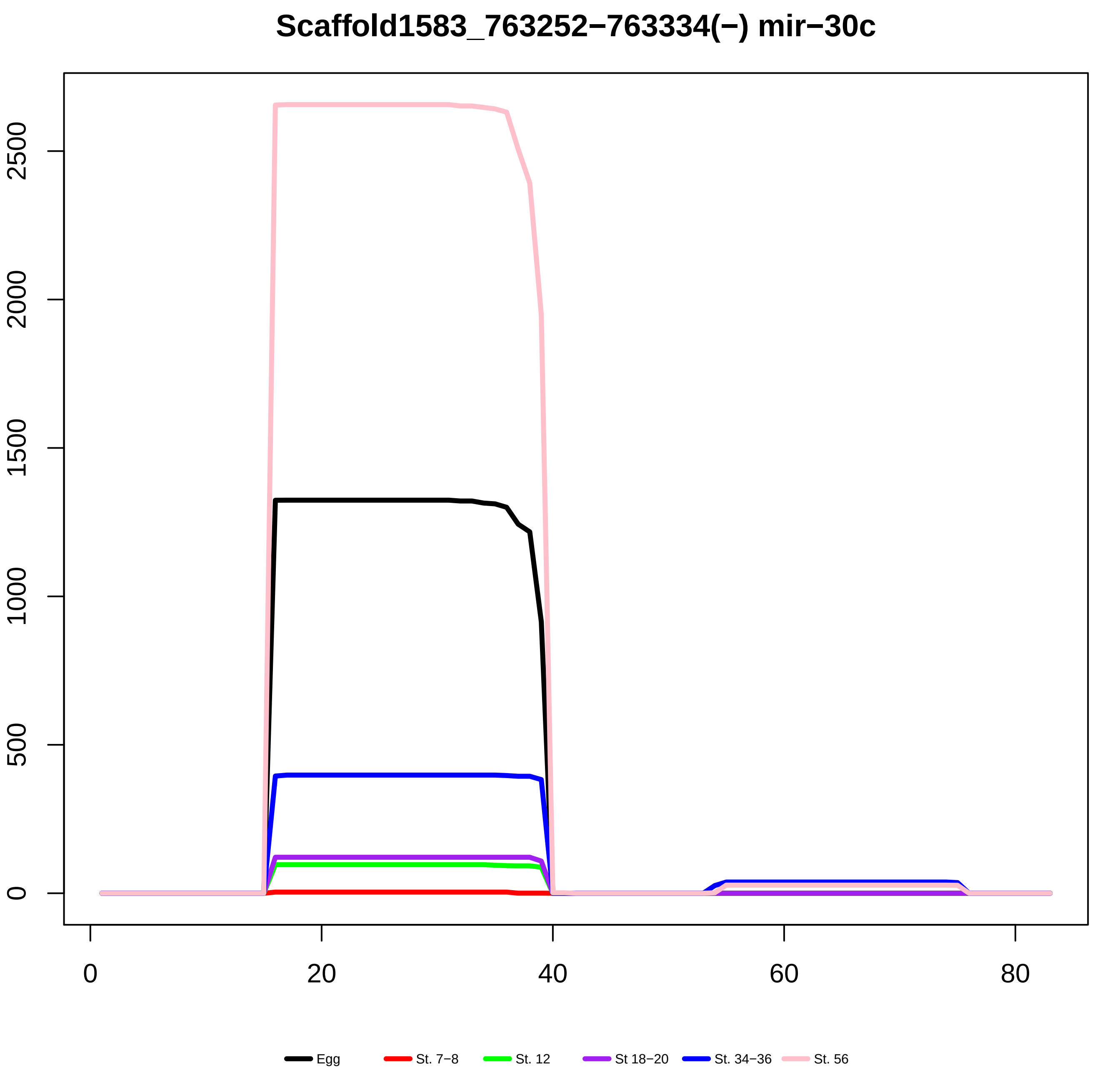

Scaffold1583_763252−763334(−) mir−30c
0
500
1000
1500
2000
2500
0
20
40
60
80
Egg
St. 7−8
St. 12
St 18−20
St. 34−36
St. 56

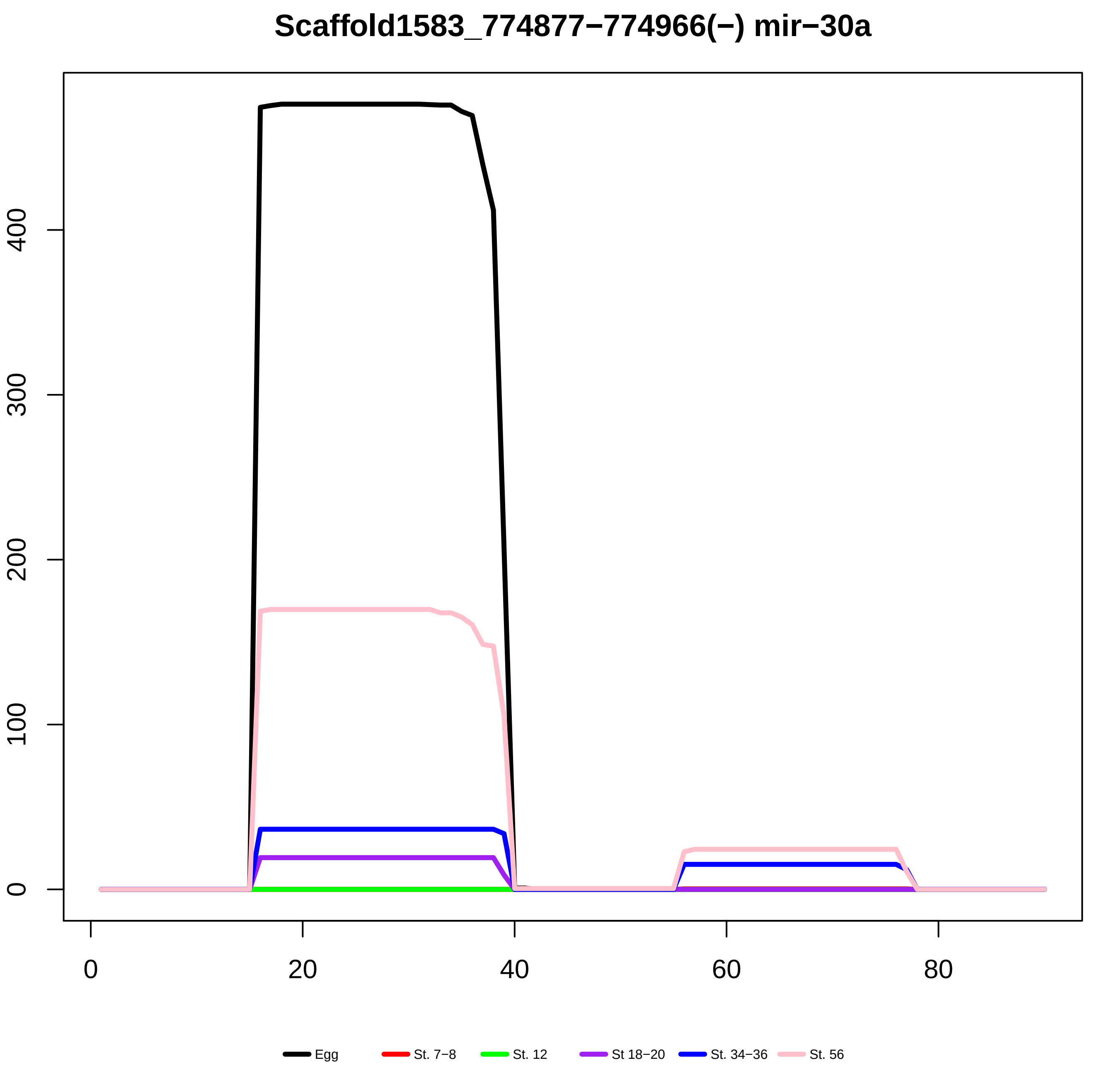
Scaffold1583_774877−774966(−) mir−30a
0
100
200
300
400
0
20
40
60
80
Egg
St. 7−8
St. 12
St 18−20
St. 34−36
St. 56

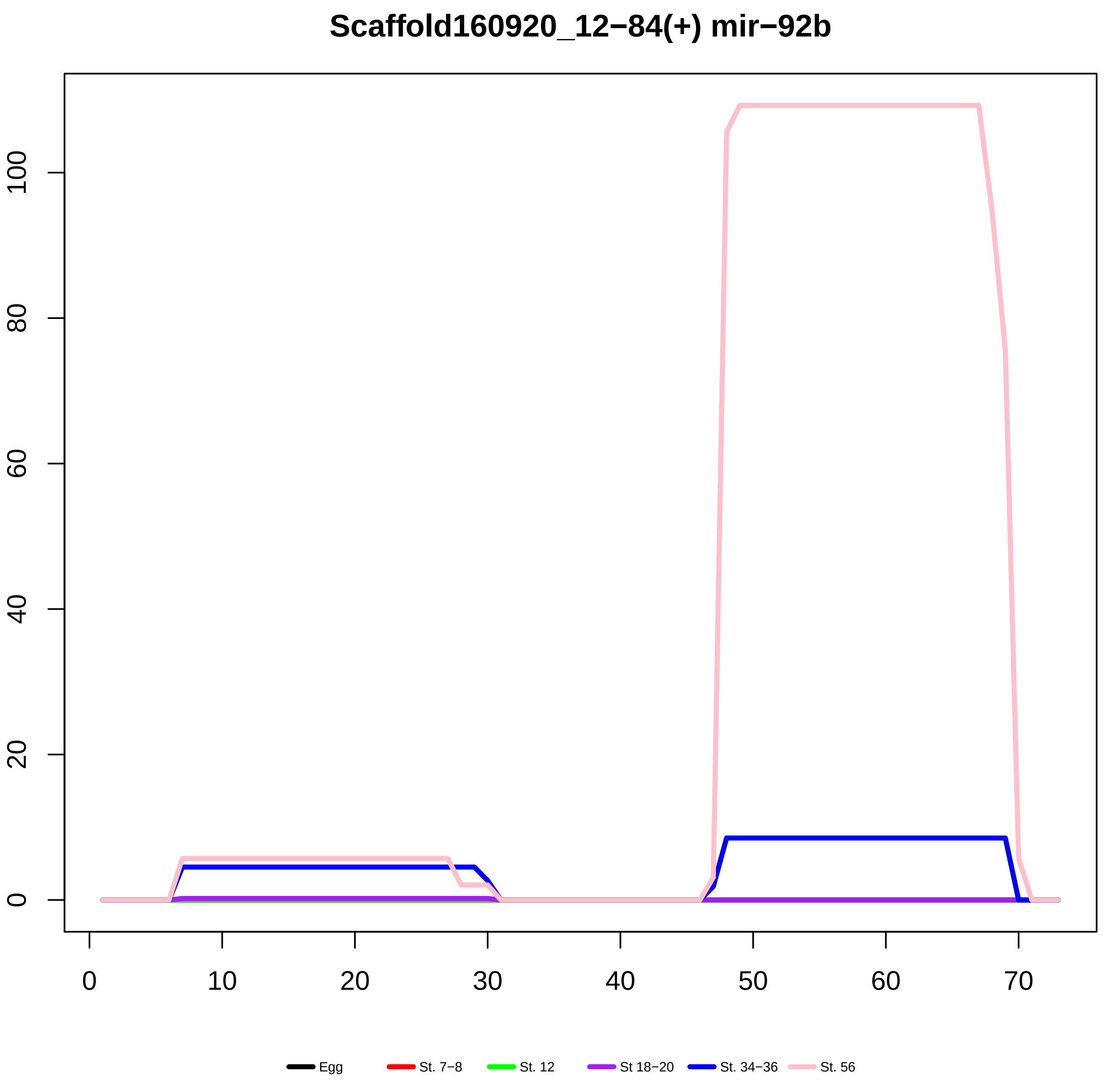
Scaffold160920_12−84(+) mir−92b
0
20
40
60
80
100
0
10
20
30
40
50
60
70
Egg
St. 7−8
St. 12
St 18−20
St. 34−36
St. 56

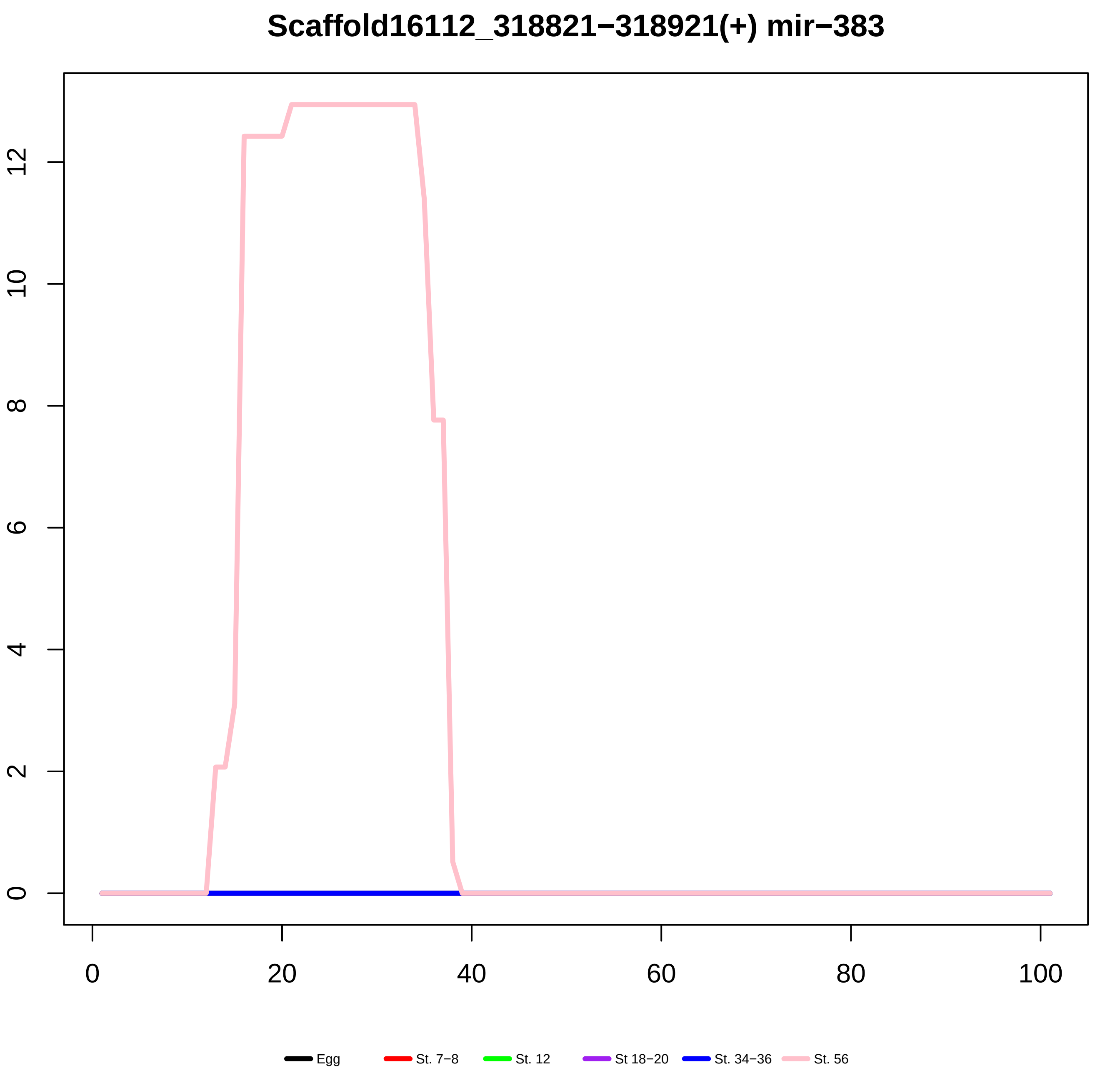

Scaffold16112_318821−318921(+) mir−383
0
2
4
6
8
10
12
0
20
40
60
80
100
Egg
St. 7−8
St. 12
St 18−20
St. 34−36
St. 56

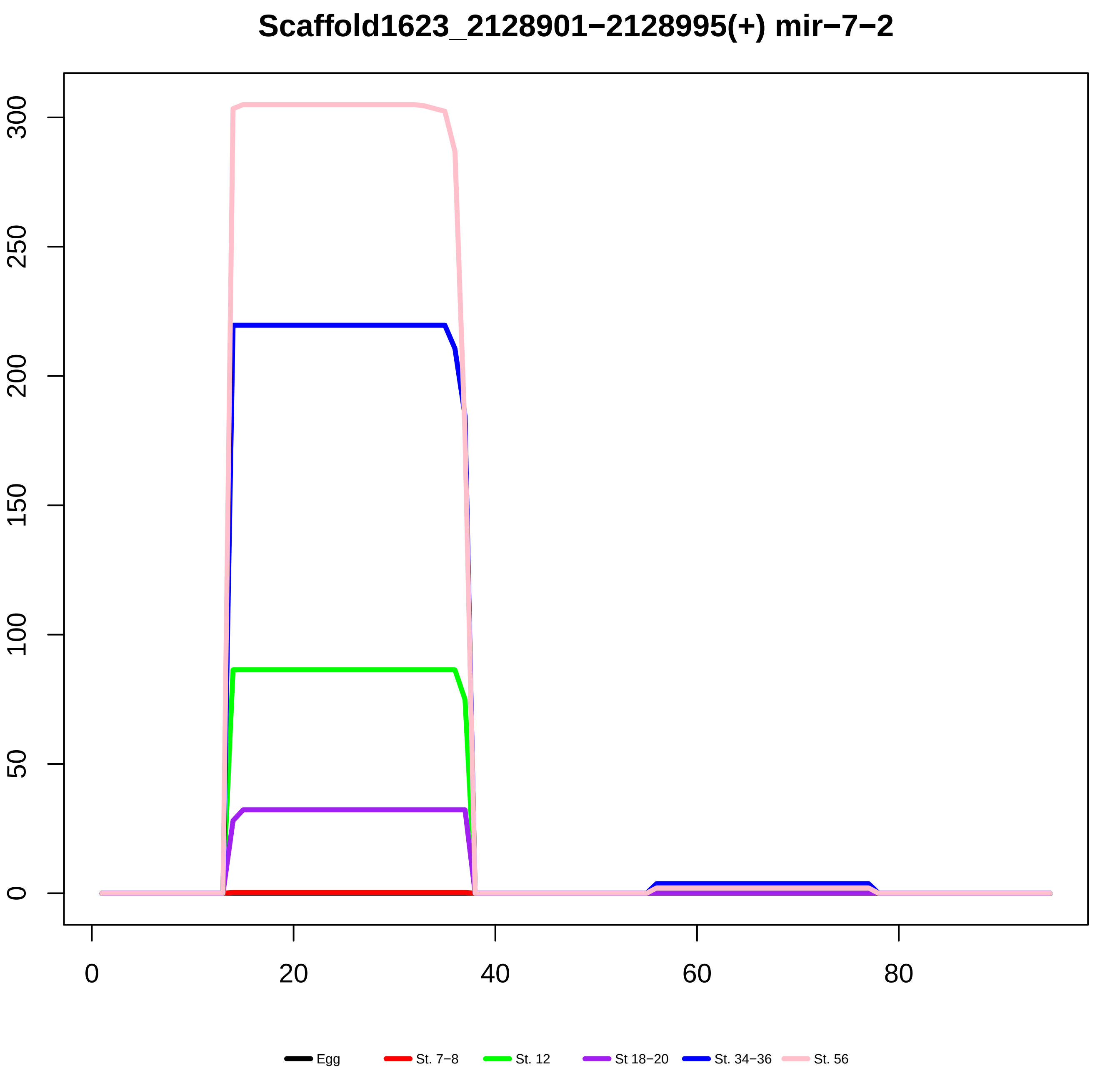
Scaffold1623_2128901−2128995(+) mir−7−2
0
50
100
150
200
250
300
0
20
40
60
80
Egg
St. 7−8
St. 12
St 18−20
St. 34−36
St. 56

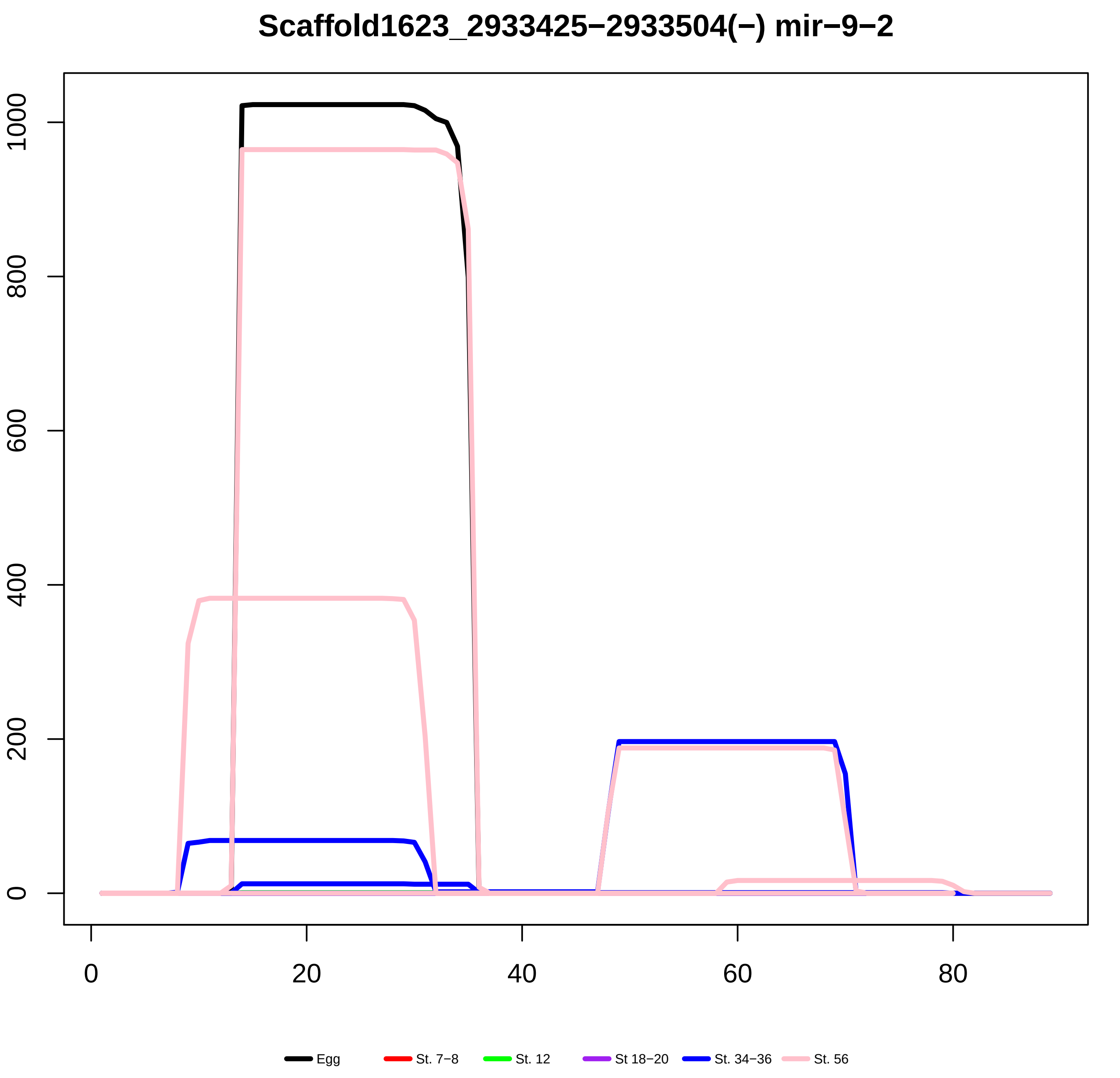
Scaffold1623_2933425−2933504(−) mir−9−2
0
200
400
600
800
1000
0
20
40
60
80
Egg
St. 7−8
St. 12
St 18−20
St. 34−36
St. 56

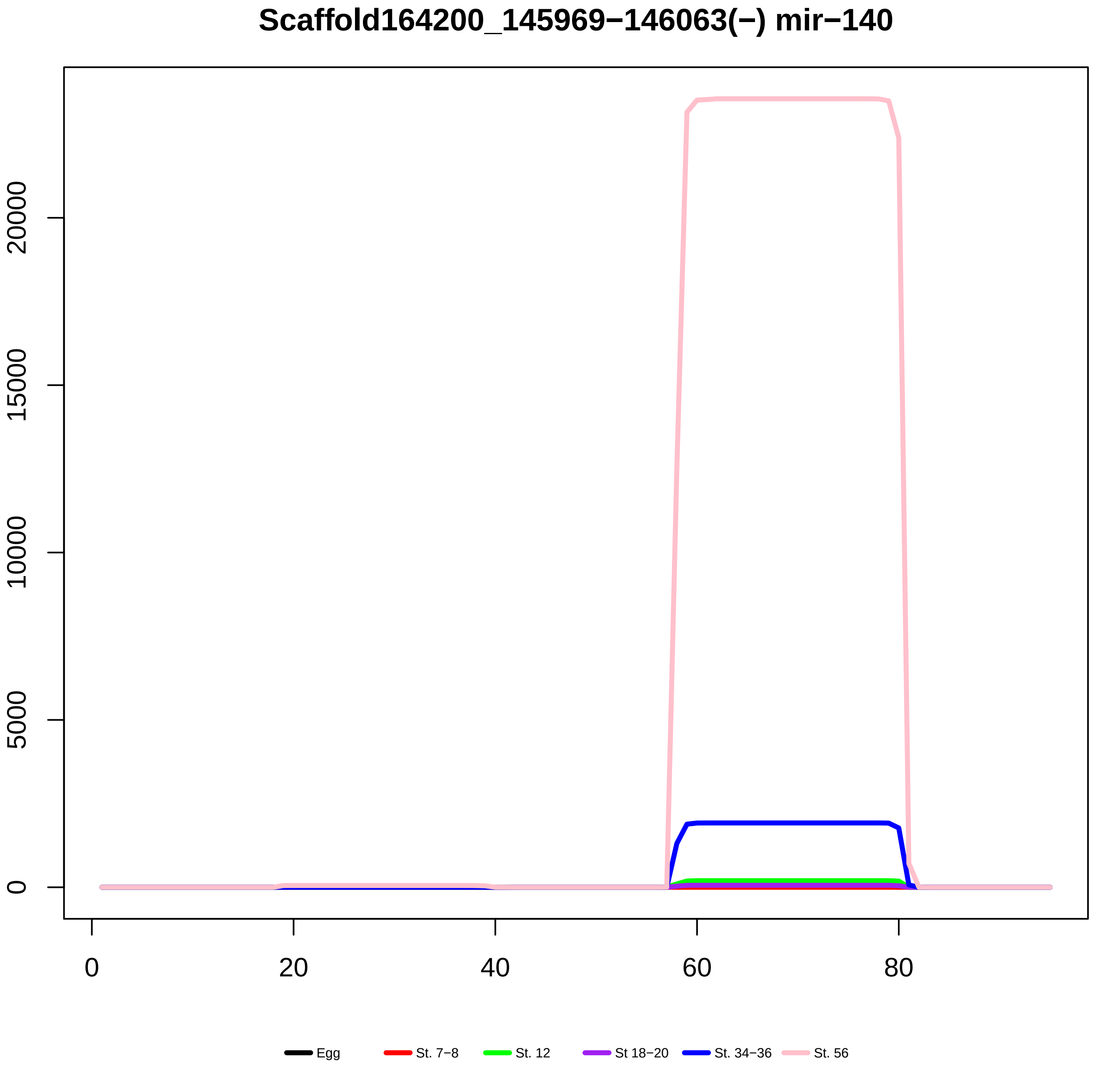

Scaffold164200_145969−146063(−) mir−140
0
5000
10000
15000
20000
0
20
40
60
80
Egg
St. 7−8
St. 12
St 18−20
St. 34−36
St. 56

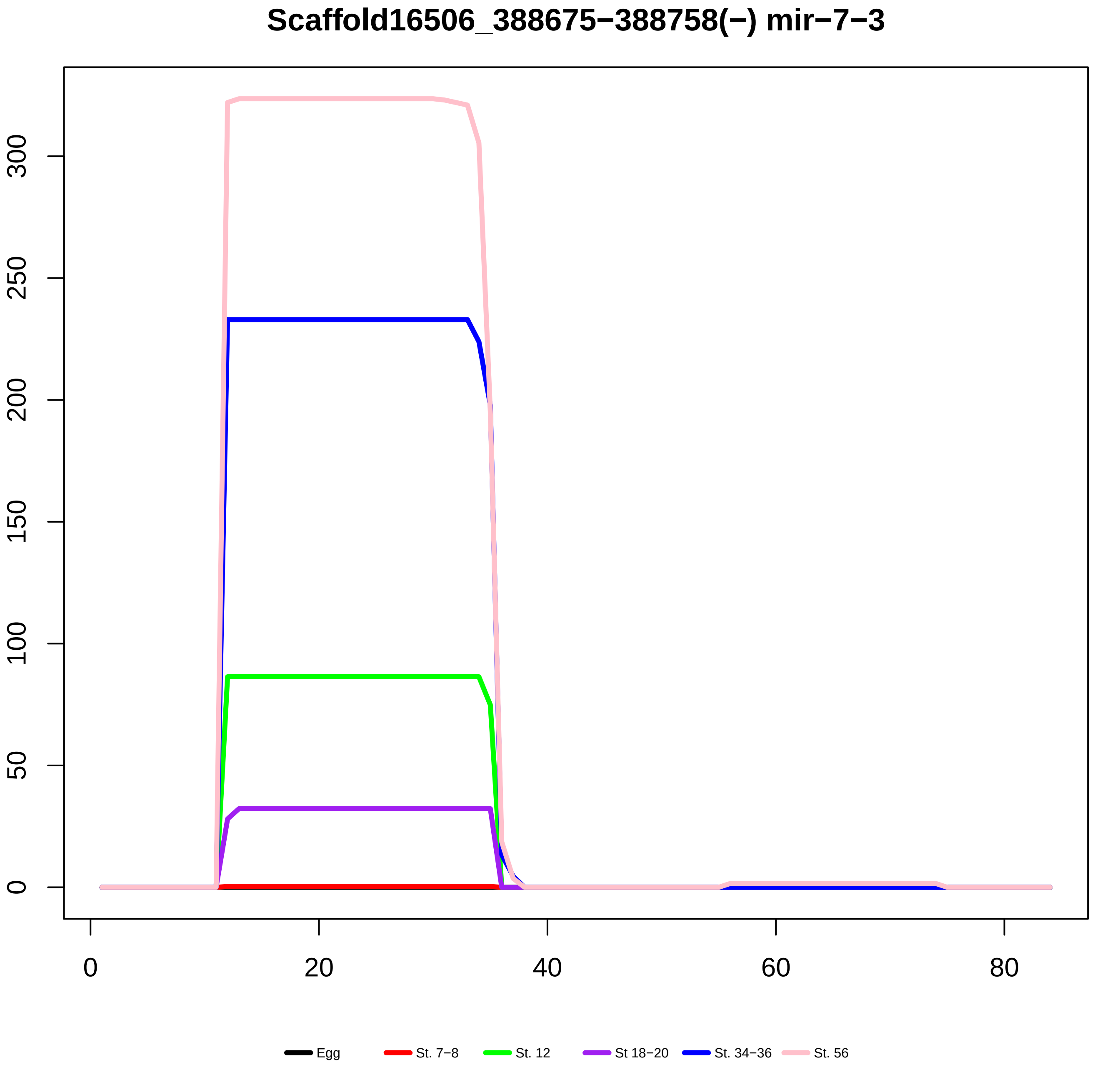

Scaffold16506_388675−388758(−) mir−7−3
0
50
100
150
200
250
300
0
20
40
60
80
Egg
St. 7−8
St. 12
St 18−20
St. 34−36
St. 56

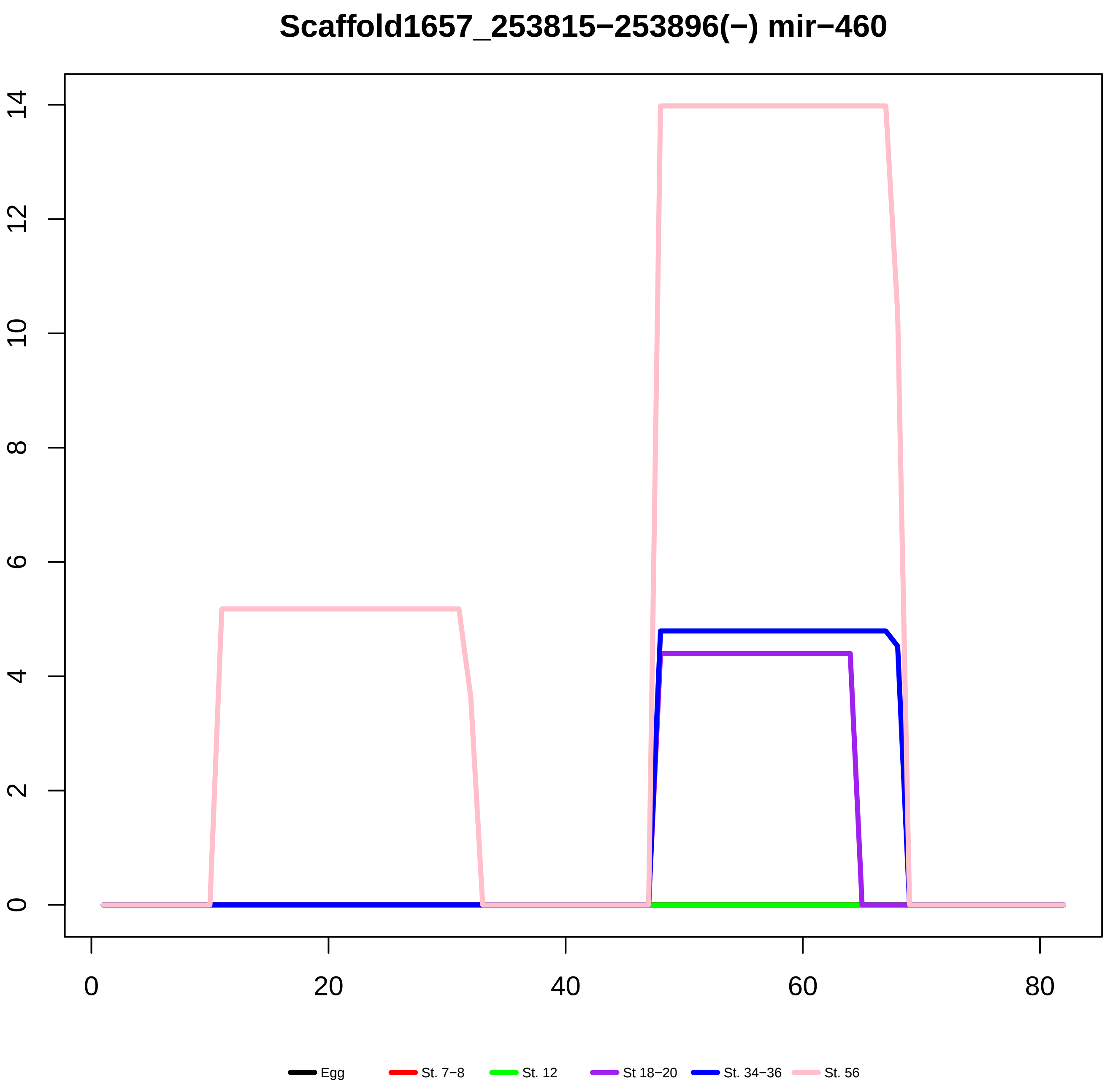

Scaffold1657_253815−253896(−) mir−460
0
2
4
6
8
10
12
14
0
20
40
60
80
Egg
St. 7−8
St. 12
St 18−20
St. 34−36
St. 56

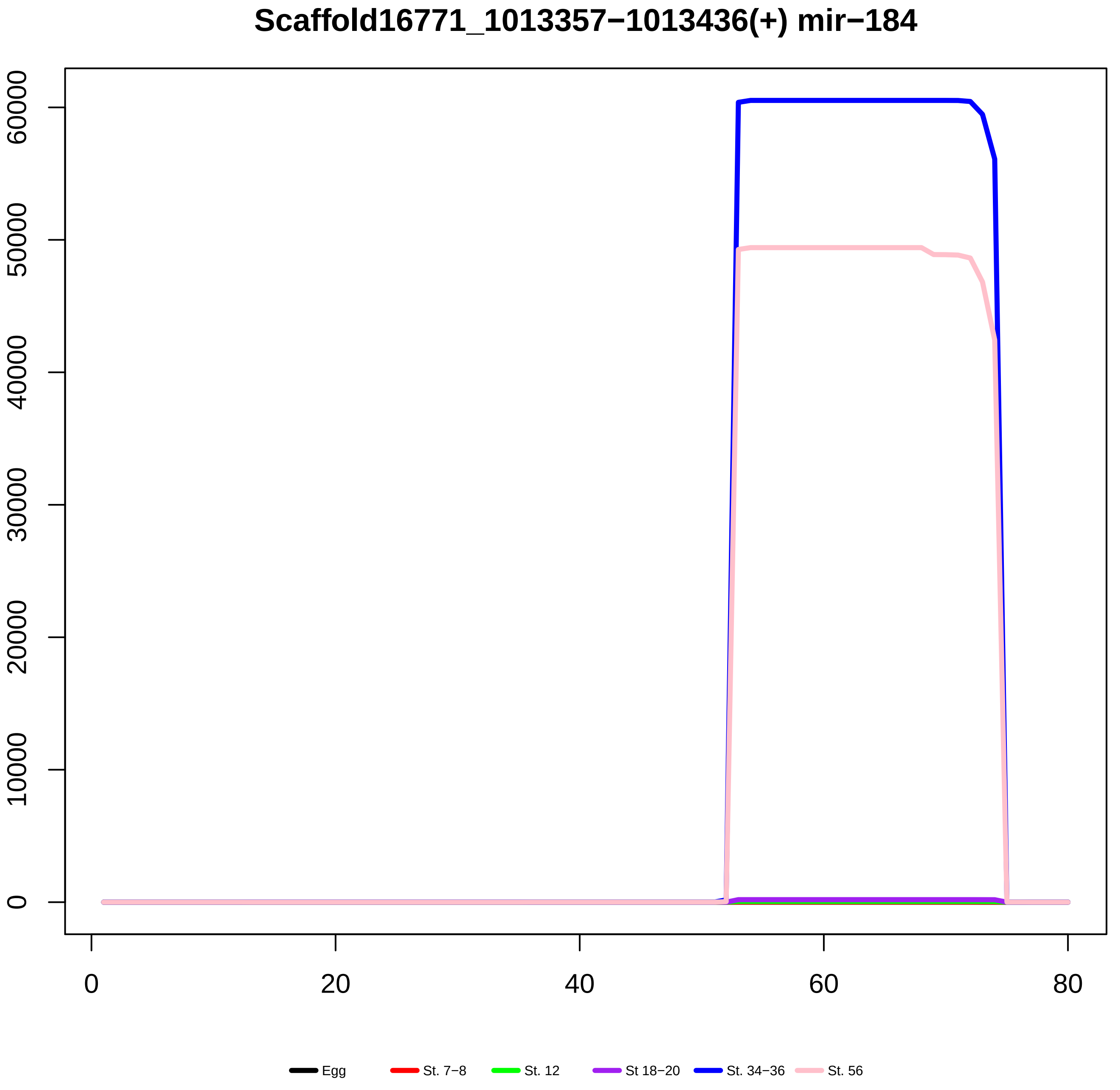
Scaffold16771_1013357−1013436(+) mir−184
0
10000
20000
30000
40000
50000
60000
0
20
40
60
80
Egg
St. 7−8
St. 12
St 18−20
St. 34−36
St. 56

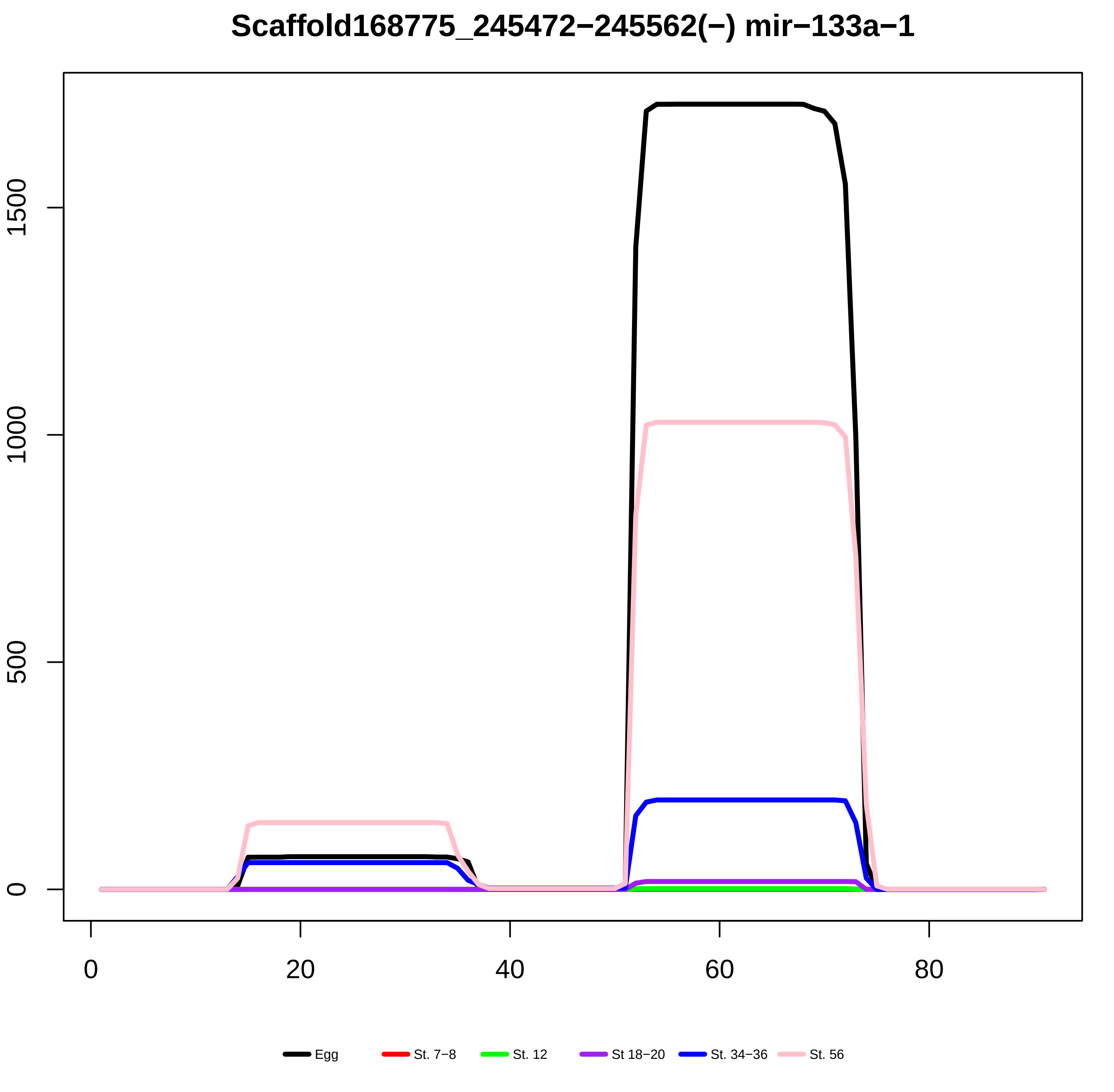
Scaffold168775_245472−245562(−) mir−133a−1
0
500
1000
1500
0
20
40
60
80
Egg
St. 7−8
St. 12
St 18−20
St. 34−36
St. 56

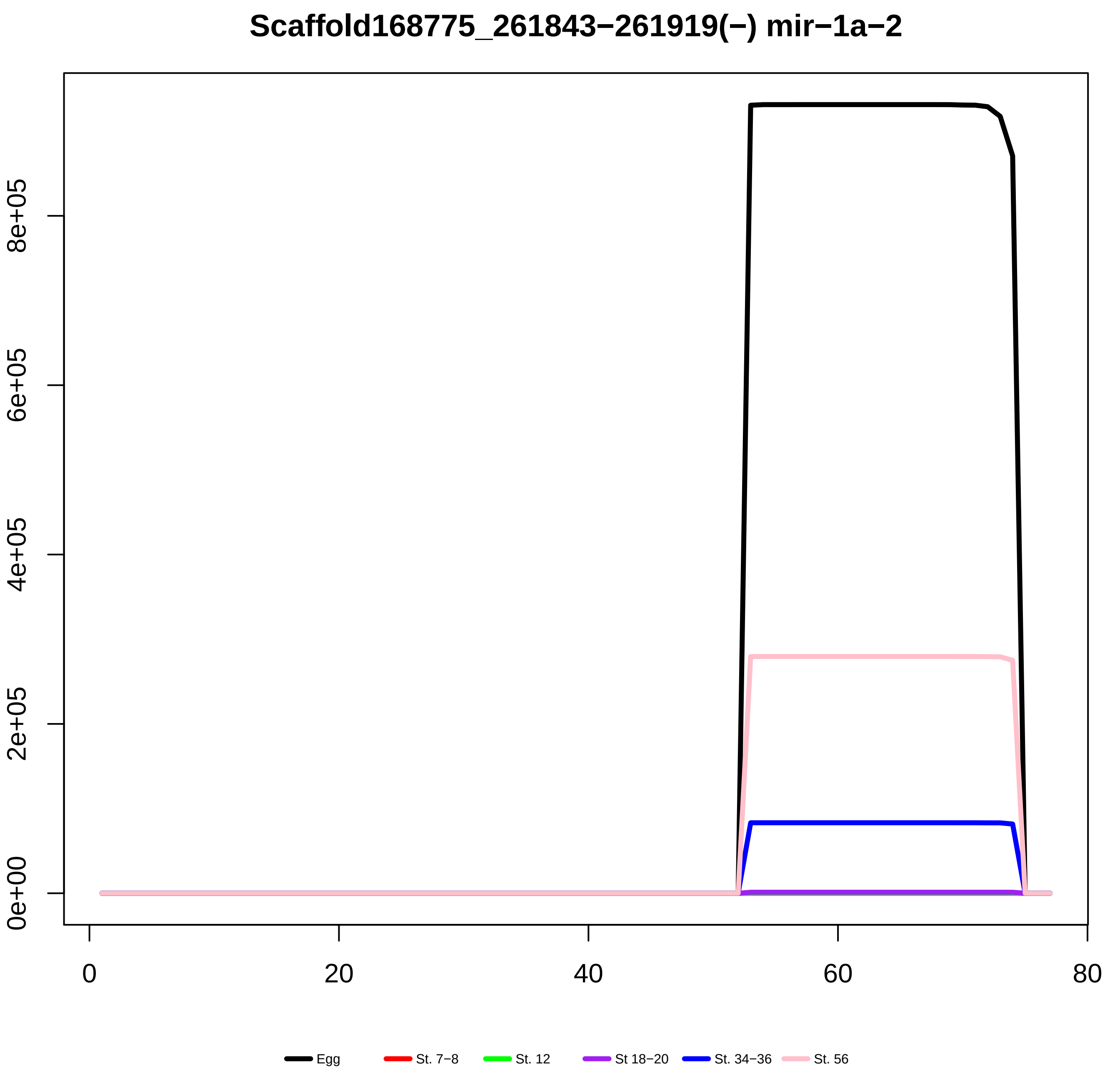
Scaffold168775_261843−261919(−) mir−1a−2
0e+00
2e+05
4e+05
6e+05
8e+05
0
20
40
60
80
Egg
St. 7−8
St. 12
St 18−20
St. 34−36
St. 56

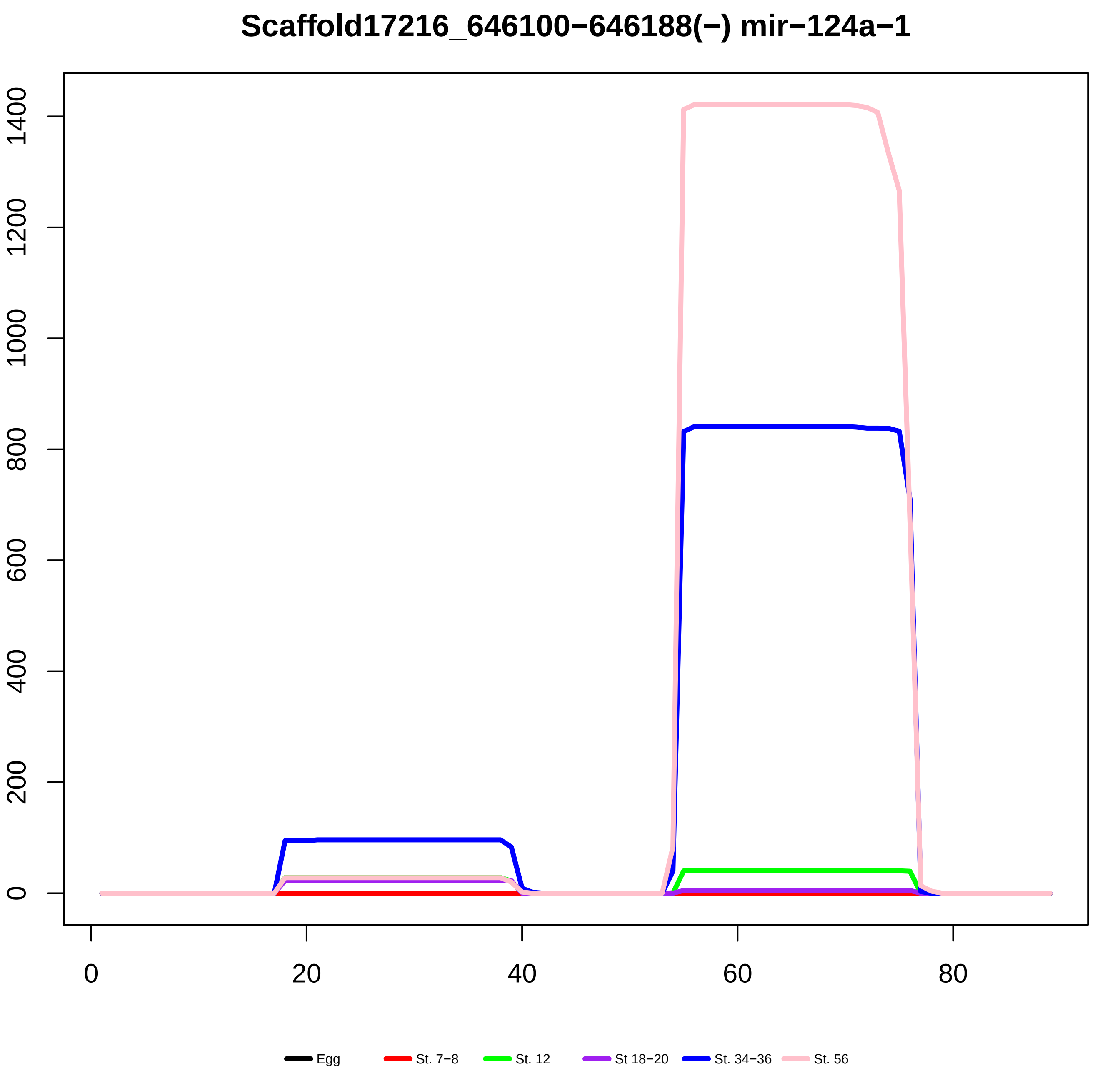
Scaffold17216_646100−646188(−) mir−124a−1
0
200
400
600
800
1000
1200
1400
0
20
40
60
80
Egg
St. 7−8
St. 12
St 18−20
St. 34−36
St. 56

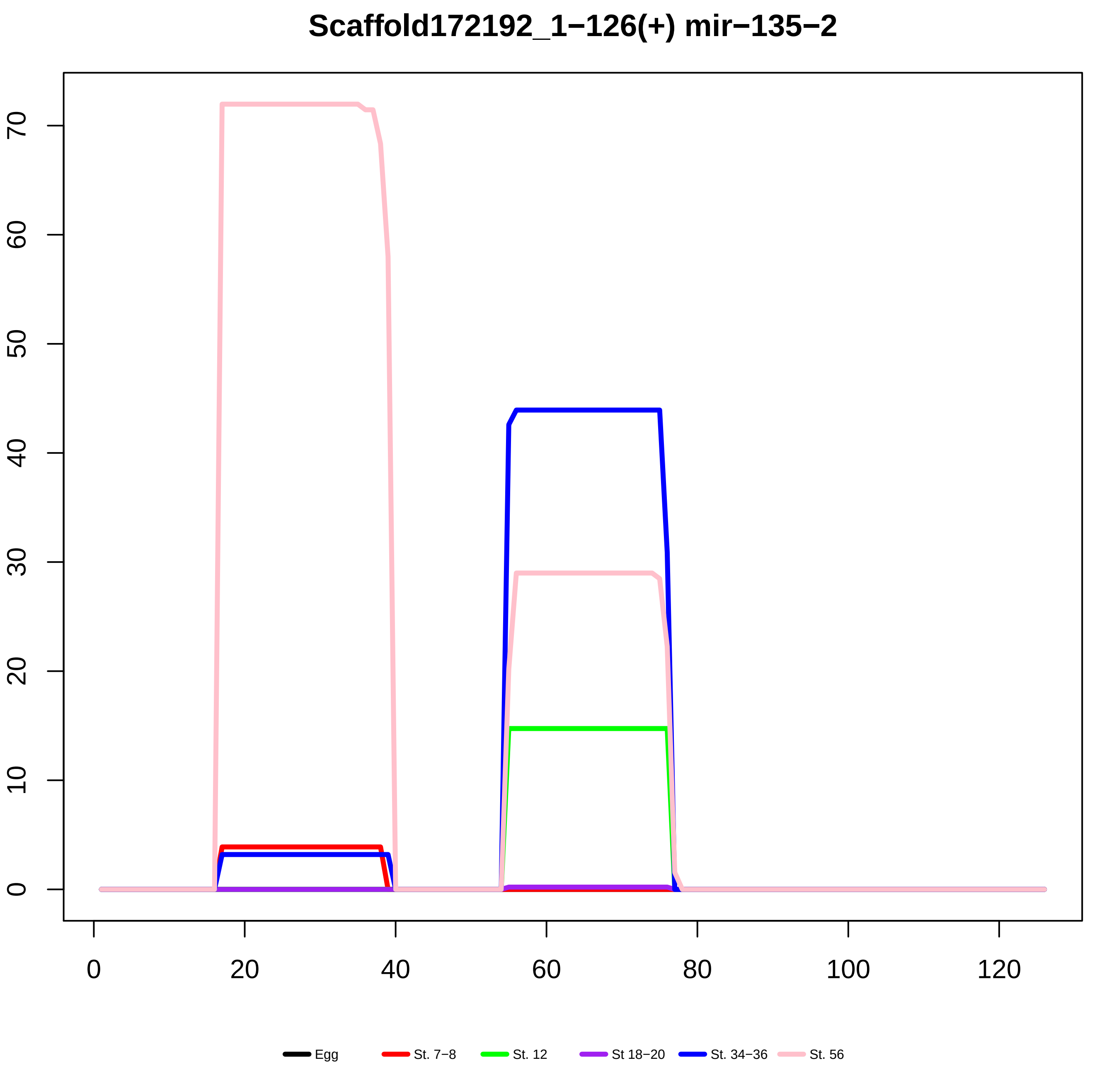

Scaffold172192_1−126(+) mir−135−2
0
10
20
30
40
50
60
70
0
20
40
60
80
100
120
Egg
St. 7−8
St. 12
St 18−20
St. 34−36
St. 56

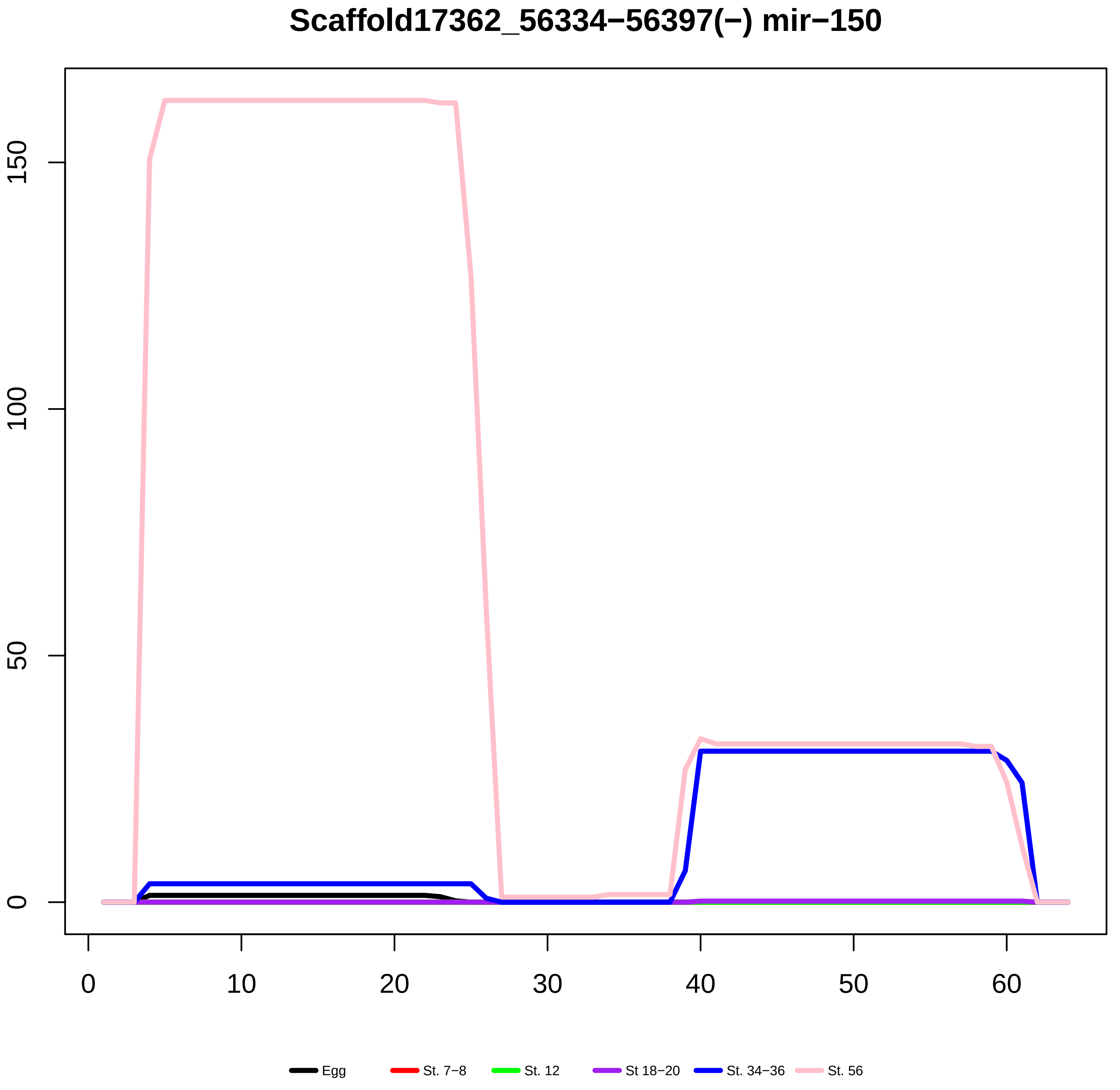
Scaffold17362_56334−56397(−) mir−150
0
50
100
150
0
10
20
30
40
50
60
Egg
St. 7−8
St. 12
St 18−20
St. 34−36
St. 56

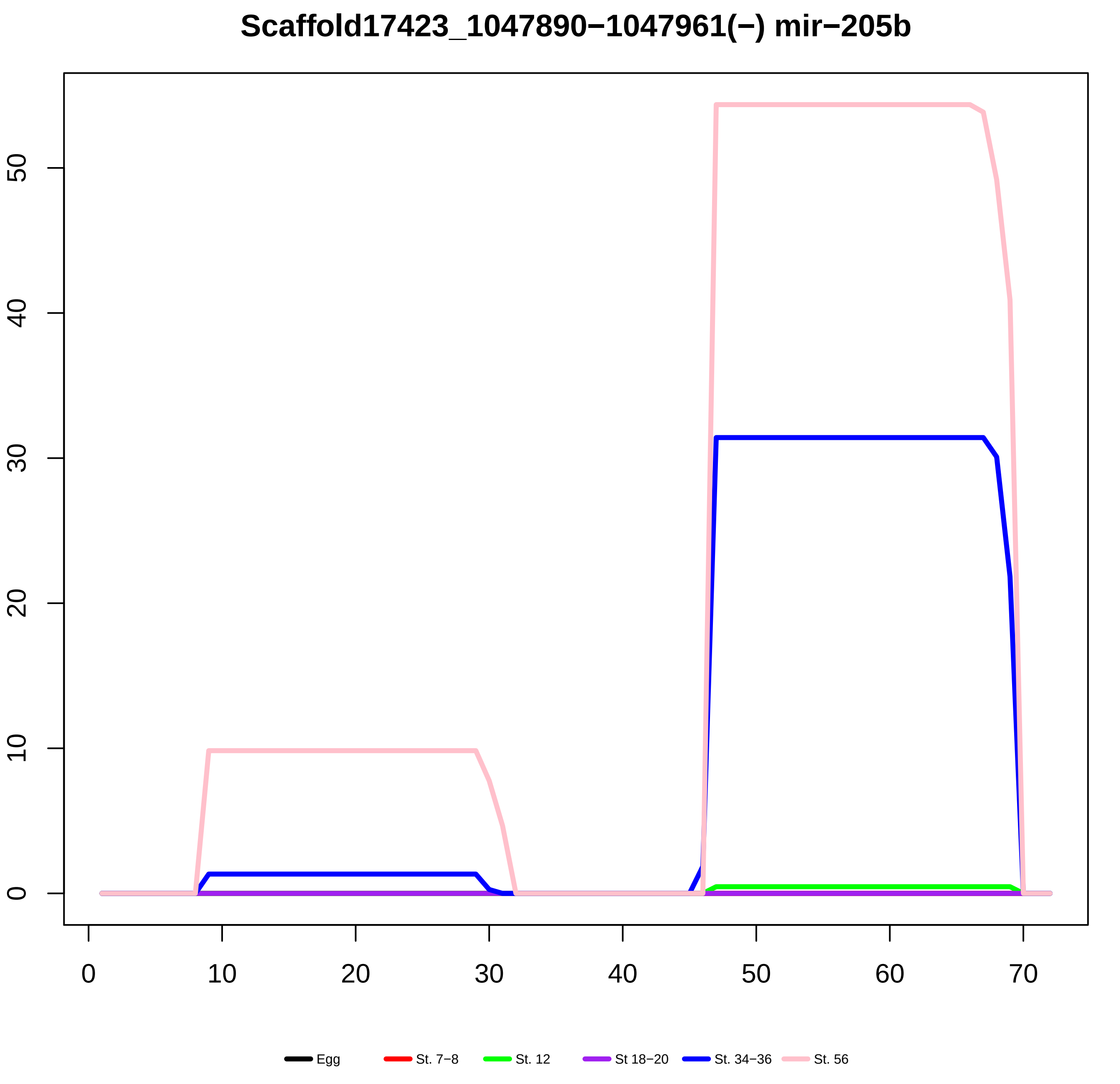

Scaffold17423_1047890−1047961(−) mir−205b
0
10
20
30
40
50
0
10
20
30
40
50
60
70
Egg
St. 7−8
St. 12
St 18−20
St. 34−36
St. 56

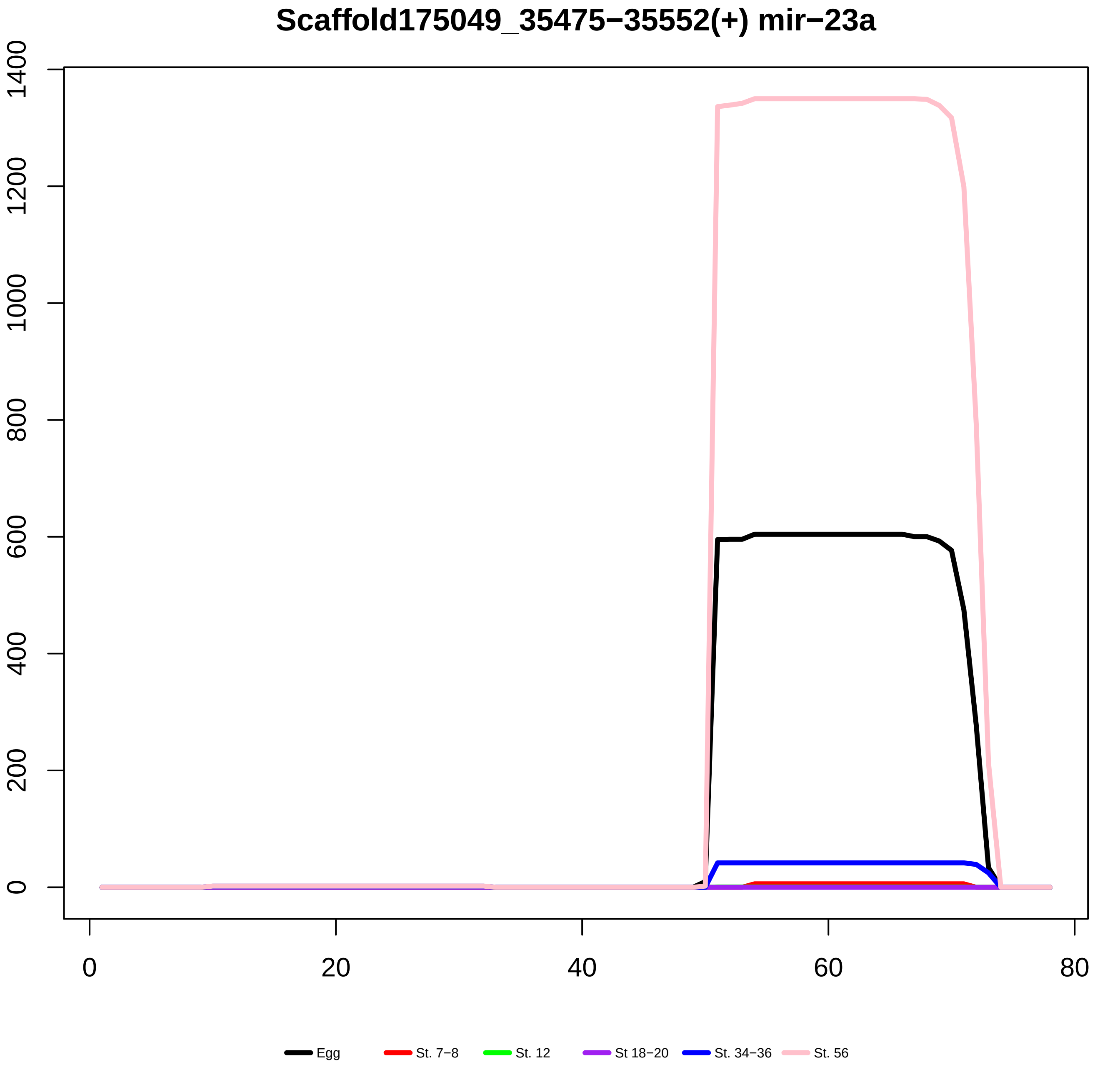

Scaffold175049_35475−35552(+) mir−23a
0
200
400
600
800
1000
1200
1400
0
20
40
60
80
Egg
St. 7−8
St. 12
St 18−20
St. 34−36
St. 56

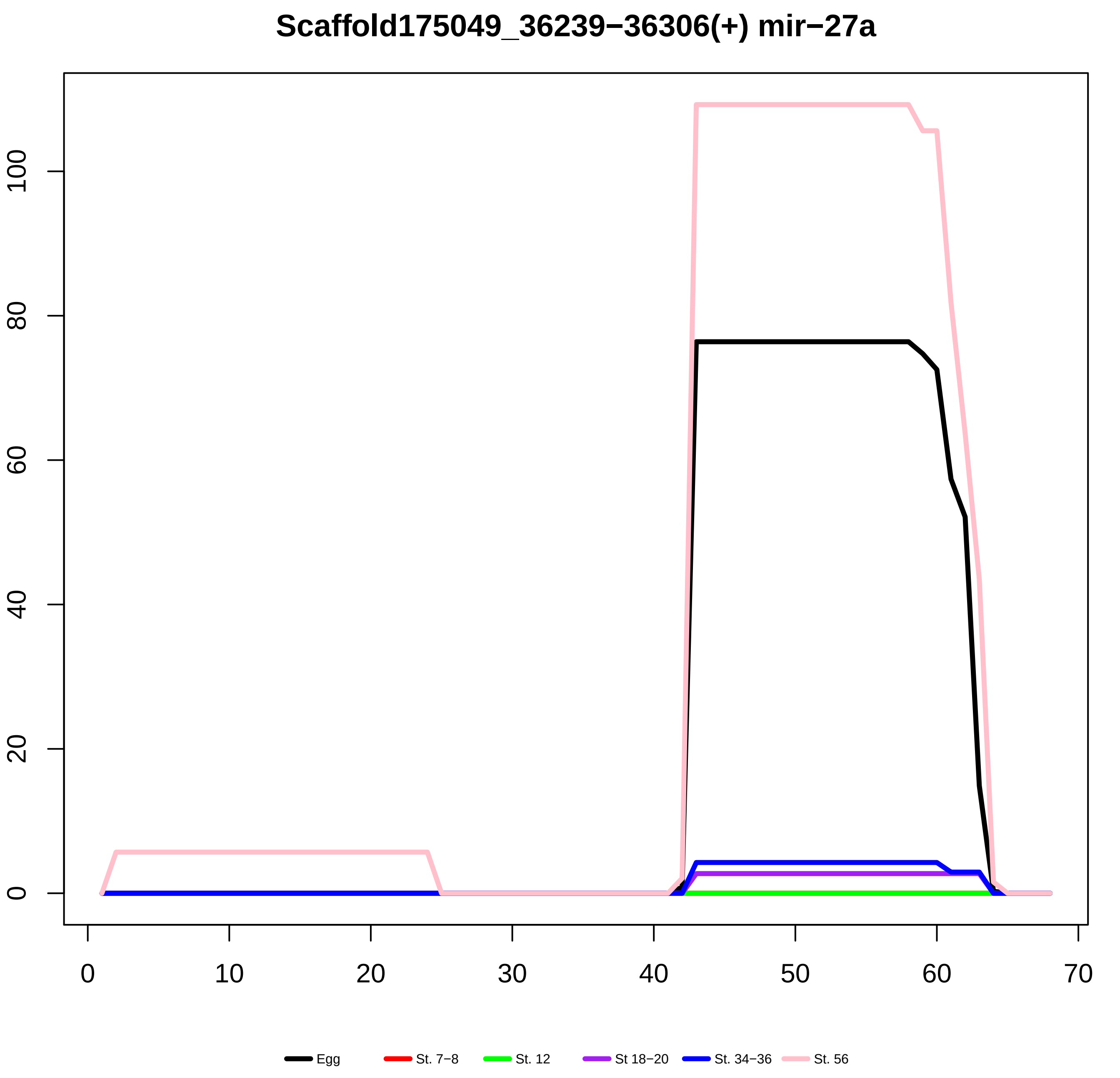
Scaffold175049_36239−36306(+) mir−27a
0
20
40
60
80
100
0
10
20
30
40
50
60
70
Egg
St. 7−8
St. 12
St 18−20
St. 34−36
St. 56

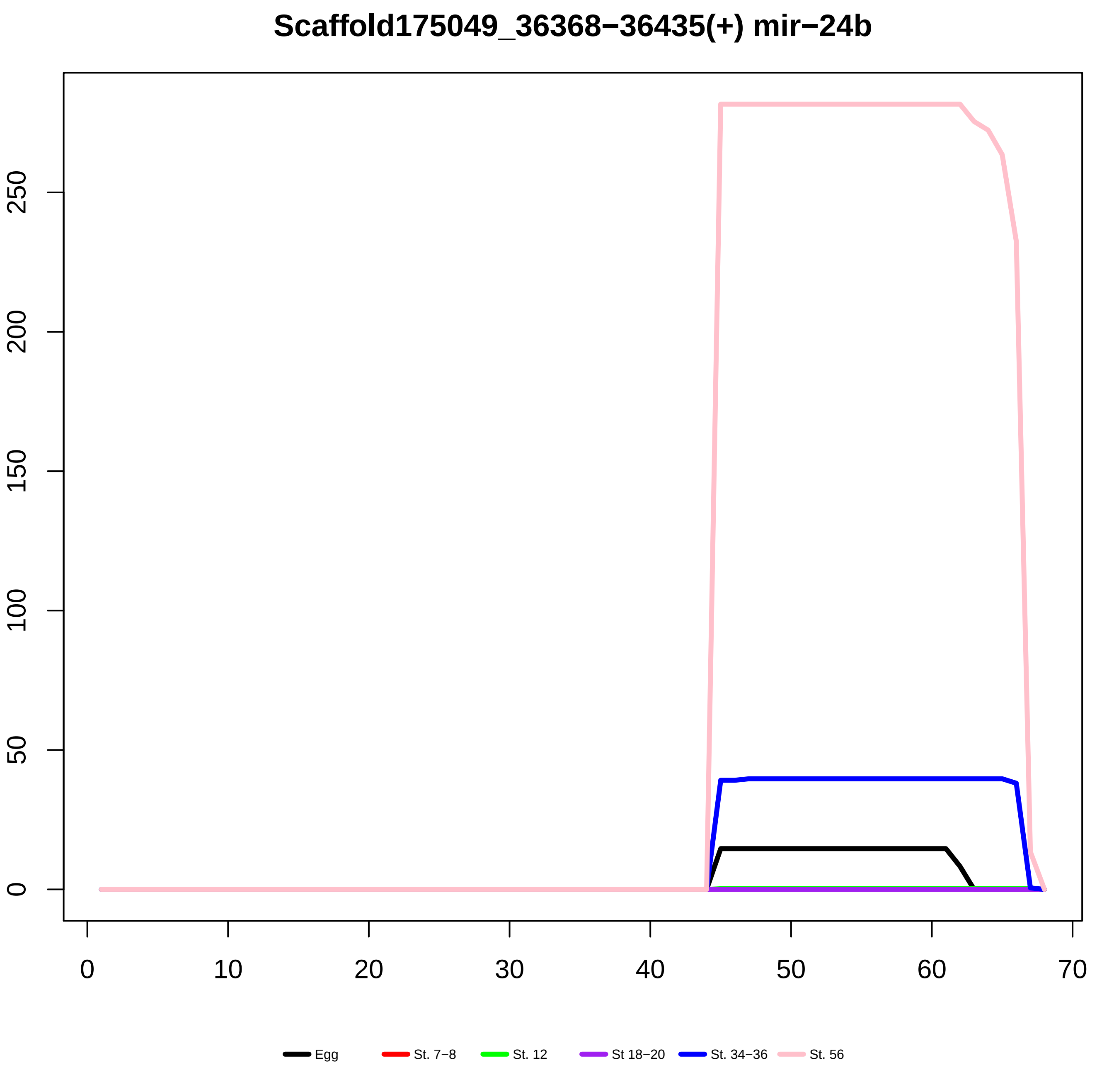
Scaffold175049_36368−36435(+) mir−24b
0
50
100
150
200
250
0
10
20
30
40
50
60
70
Egg
St. 7−8
St. 12
St 18−20
St. 34−36
St. 56

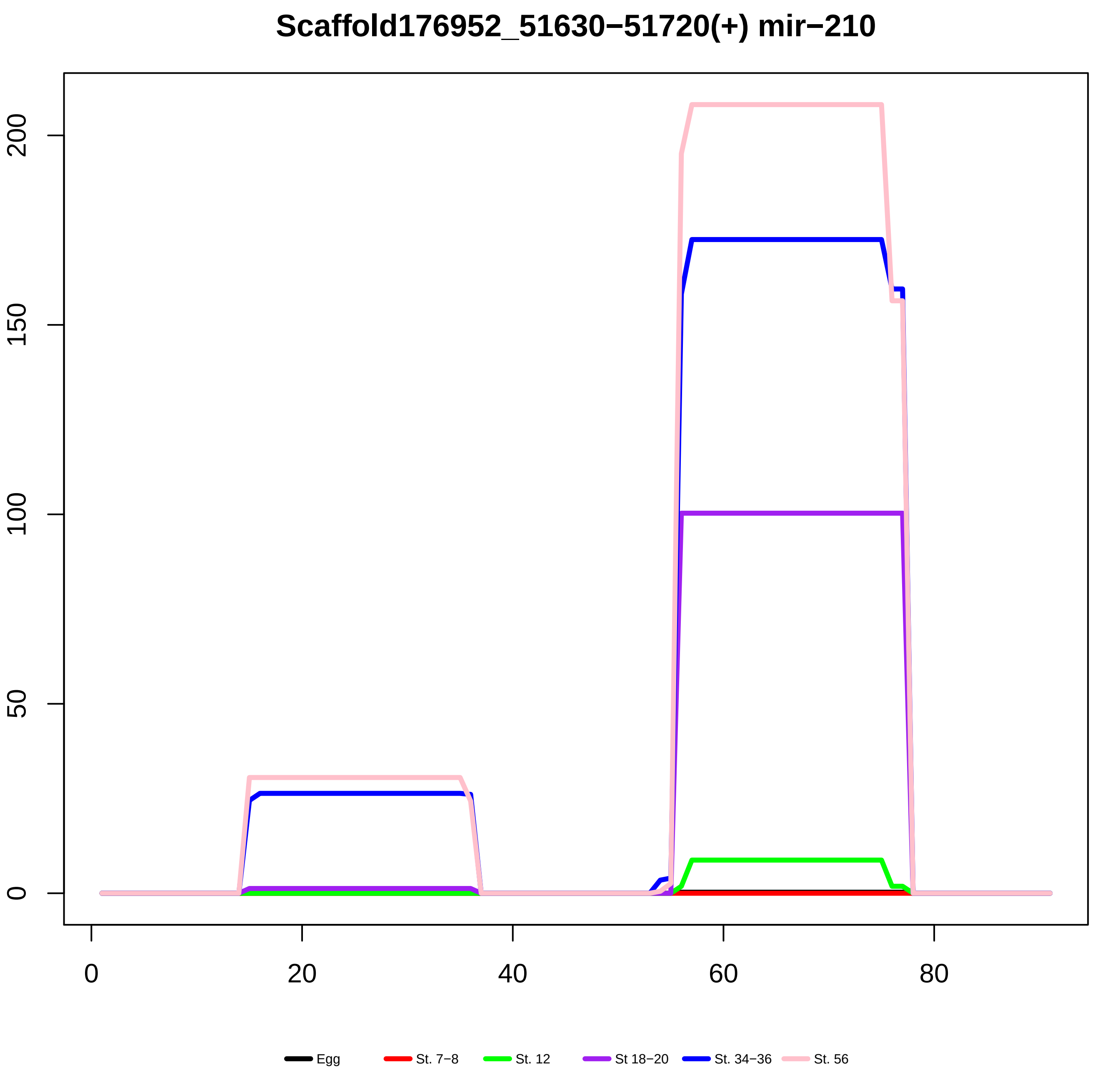

Scaffold176952_51630−51720(+) mir−210
0
50
100
150
200
0
20
40
60
80
Egg
St. 7−8
St. 12
St 18−20
St. 34−36
St. 56

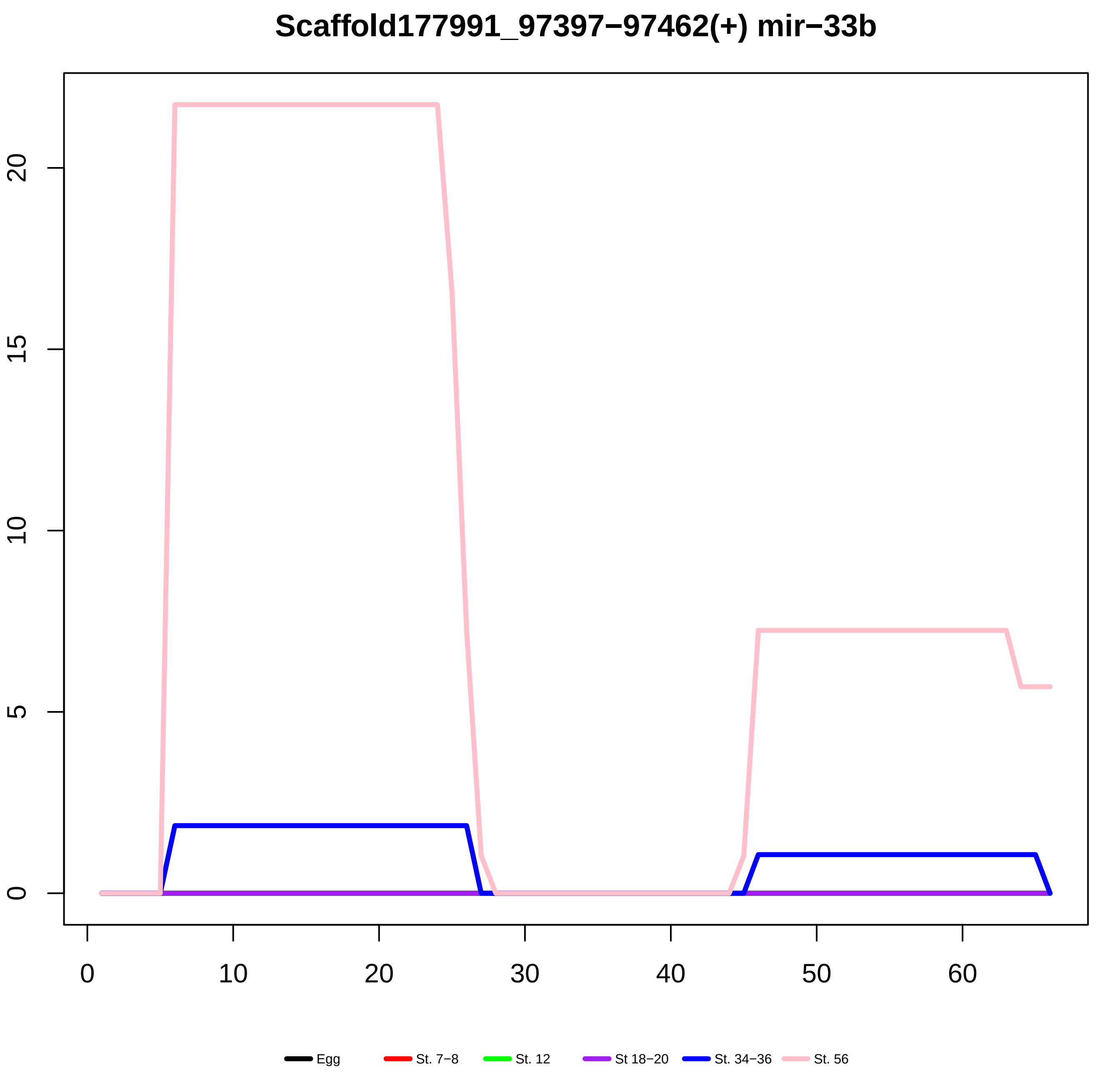
Scaffold177991_97397−97462(+) mir−33b
0
5
10
15
20
0
10
20
30
40
50
60
Egg
St. 7−8
St. 12
St 18−20
St. 34−36
St. 56

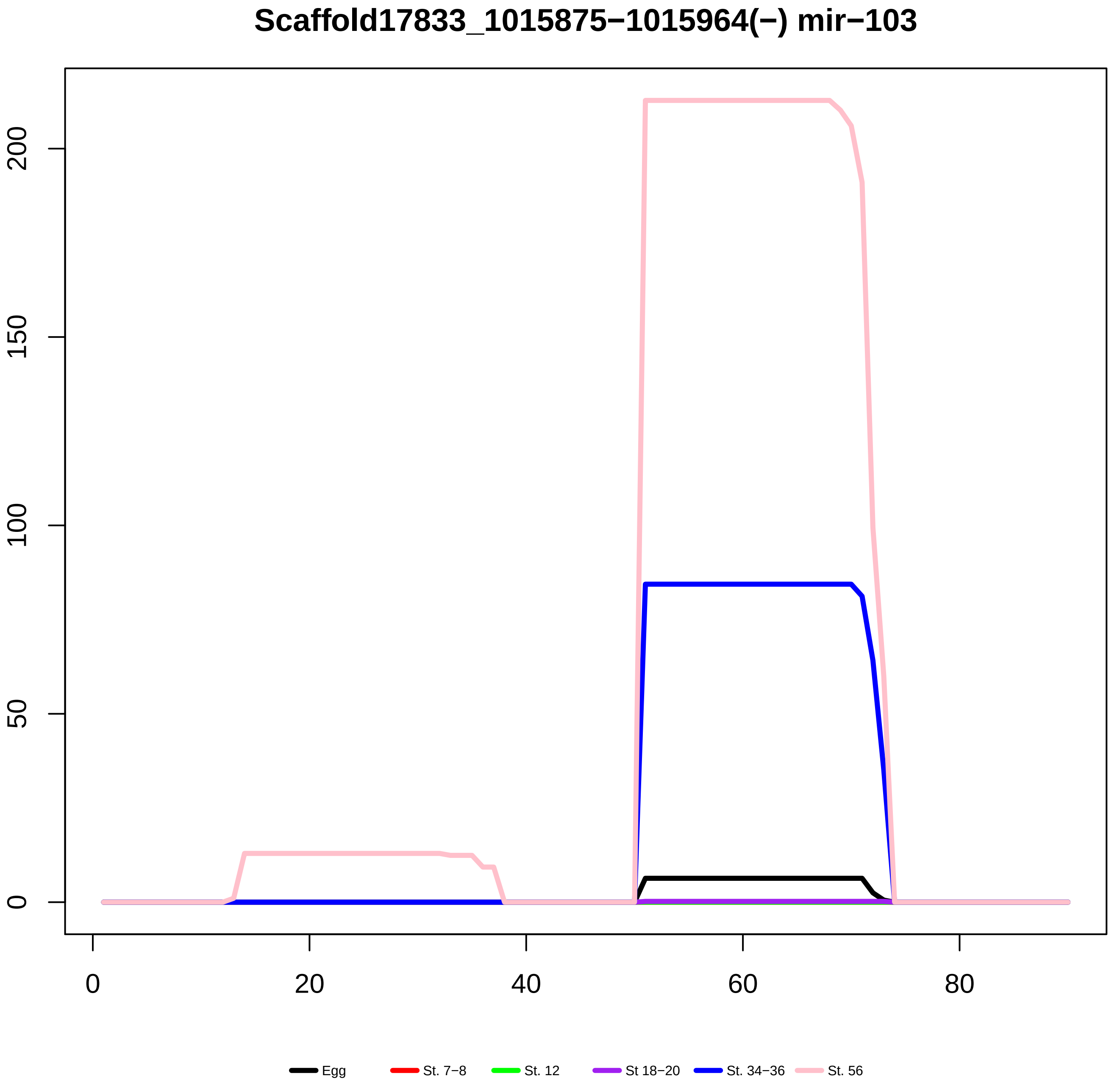
Scaffold17833_1015875−1015964(−) mir−103
0
50
100
150
200
0
20
40
60
80
Egg
St. 7−8
St. 12
St 18−20
St. 34−36
St. 56

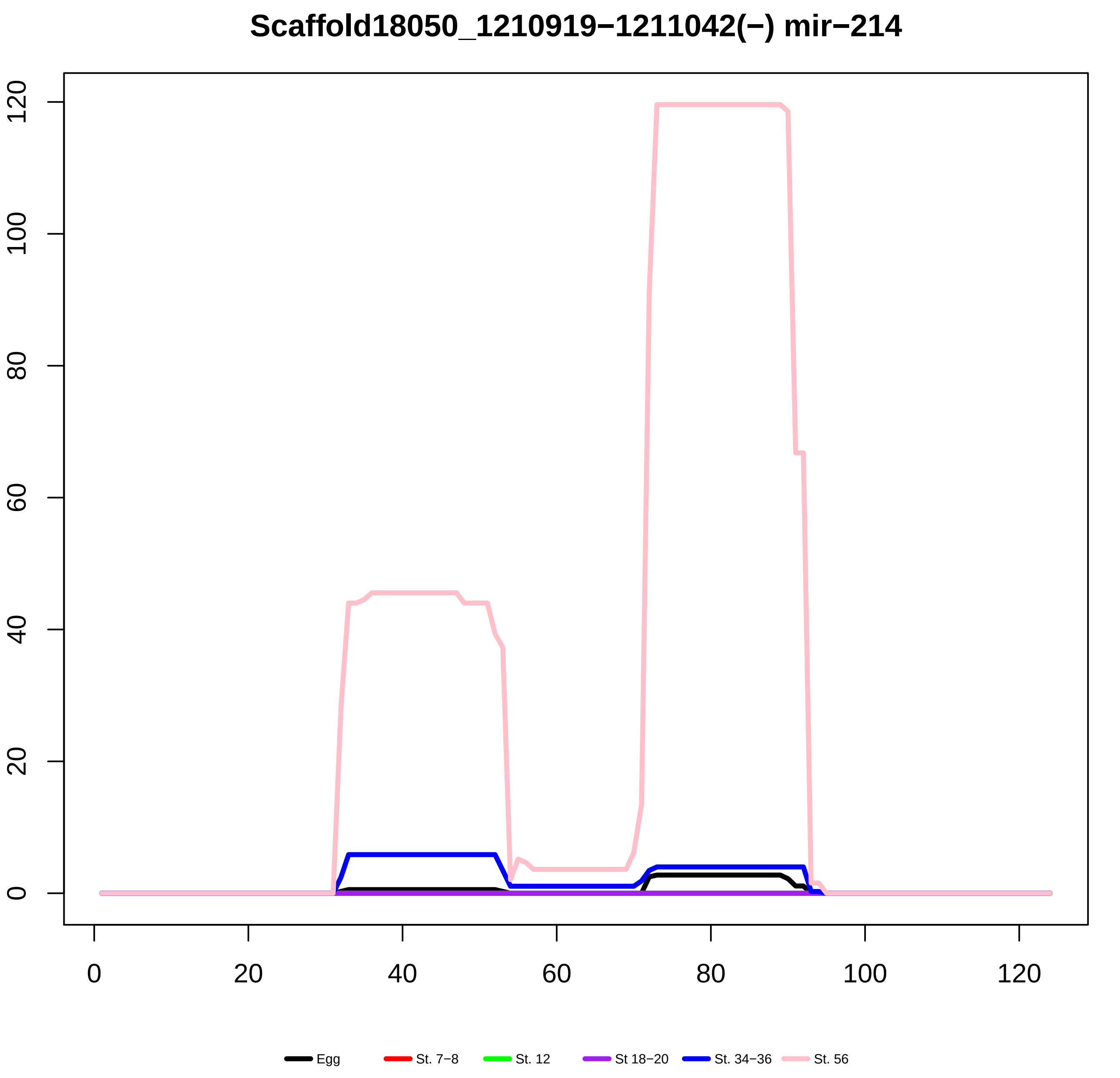

Scaffold18050_1210919−1211042(−) mir−214
0
20
40
60
80
100
120
0
20
40
60
80
100
120
Egg
St. 7−8
St. 12
St 18−20
St. 34−36
St. 56

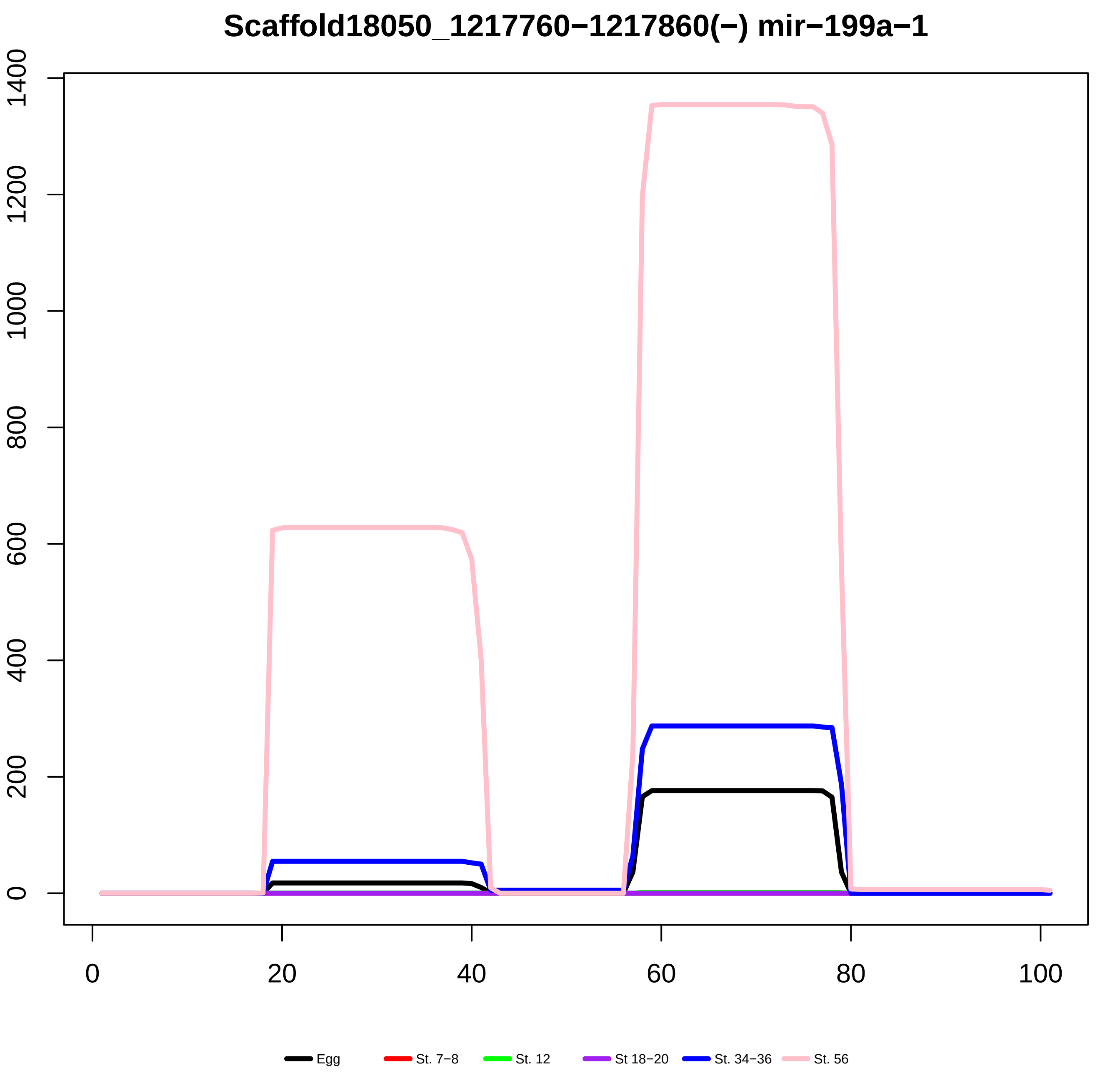

Scaffold18050_1217760−1217860(−) mir−199a−1
0
200
400
600
800
1000
1200
1400
0
20
40
60
80
100
Egg
St. 7−8
St. 12
St 18−20
St. 34−36
St. 56

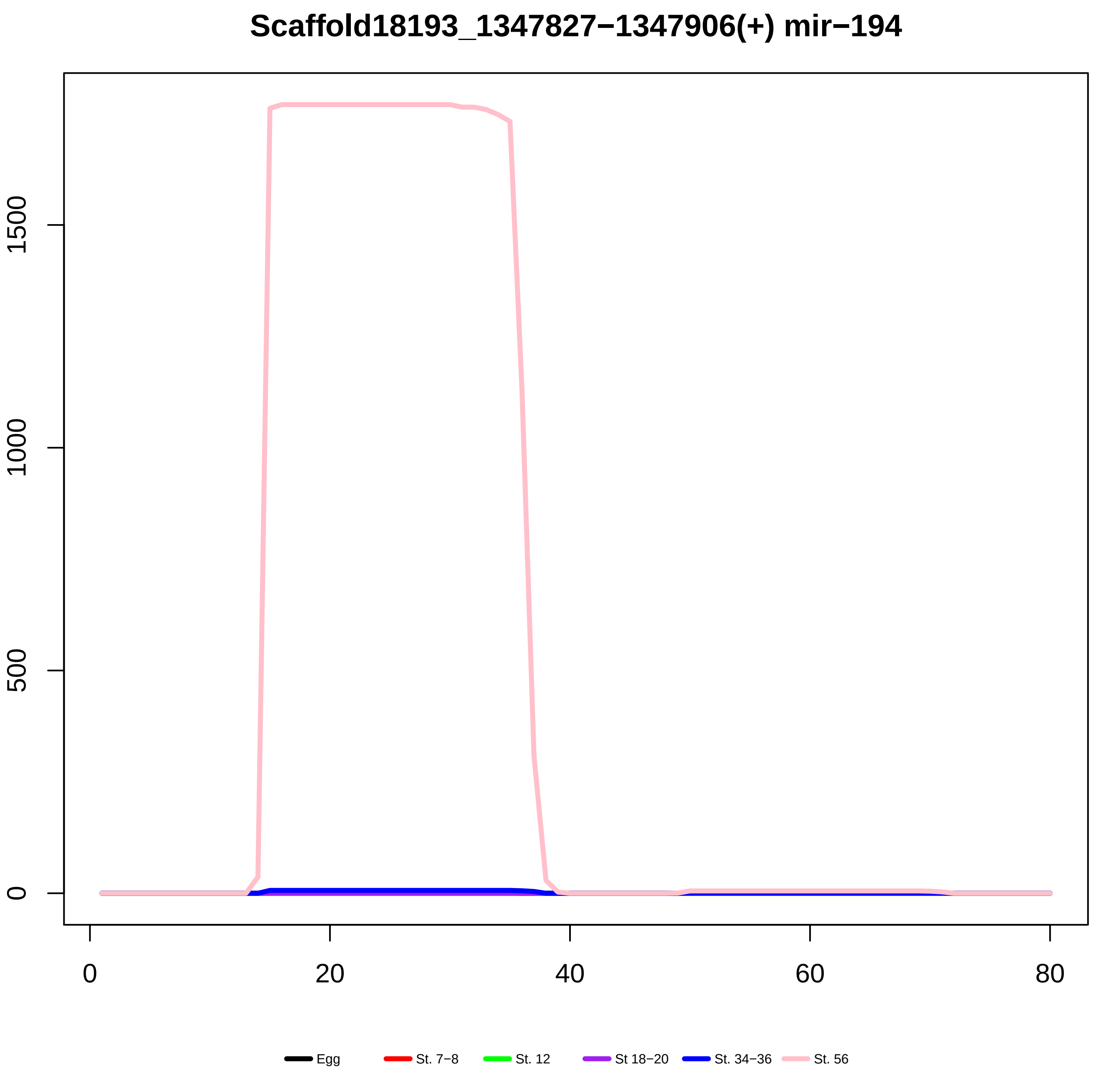

Scaffold18193_1347827−1347906(+) mir−194
0
500
1000
1500
0
20
40
60
80
Egg
St. 7−8
St. 12
St 18−20
St. 34−36
St. 56

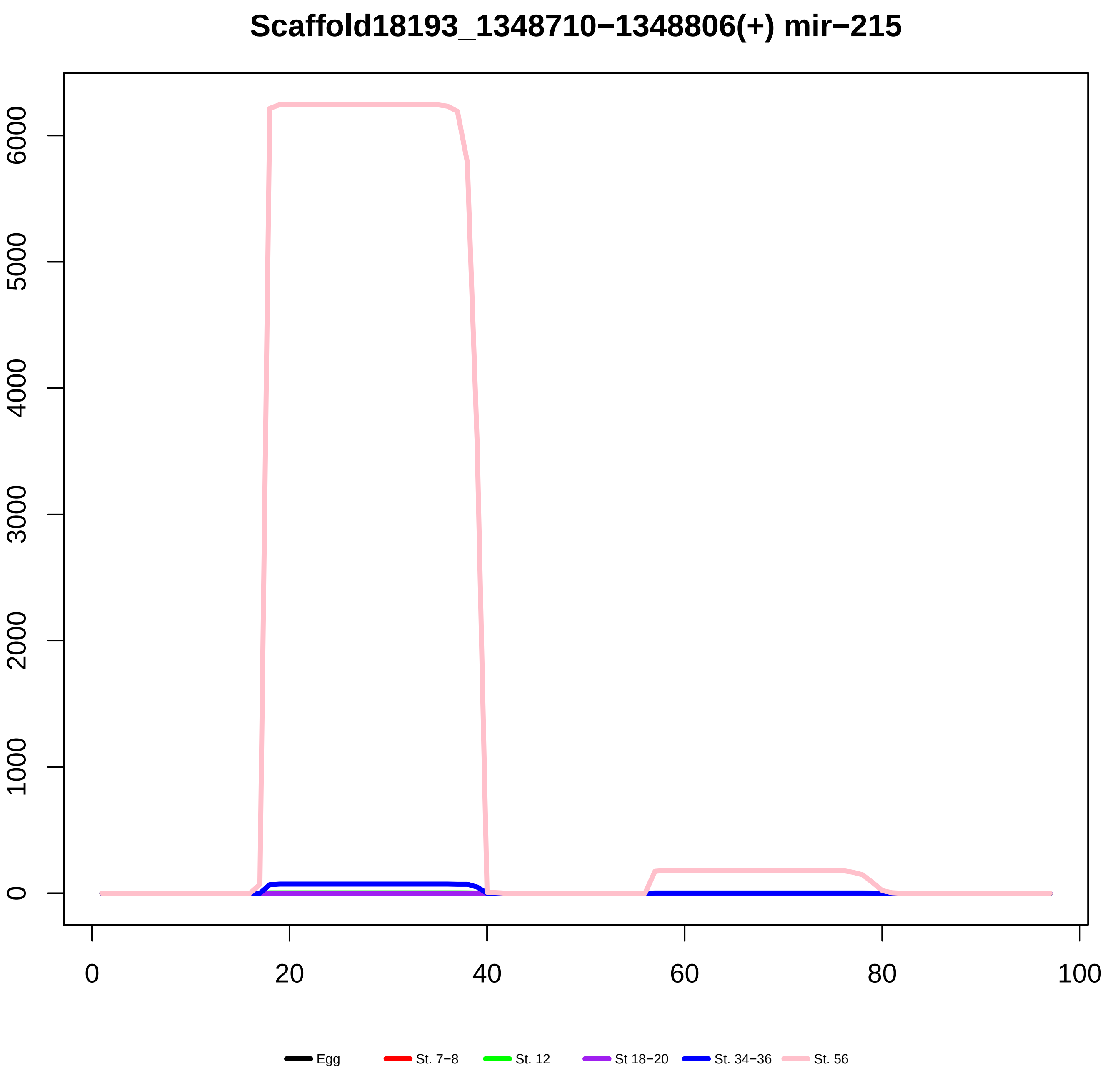
Scaffold18193_1348710−1348806(+) mir−215
0
1000
2000
3000
4000
5000
6000
0
20
40
60
80
100
Egg
St. 7−8
St. 12
St 18−20
St. 34−36
St. 56

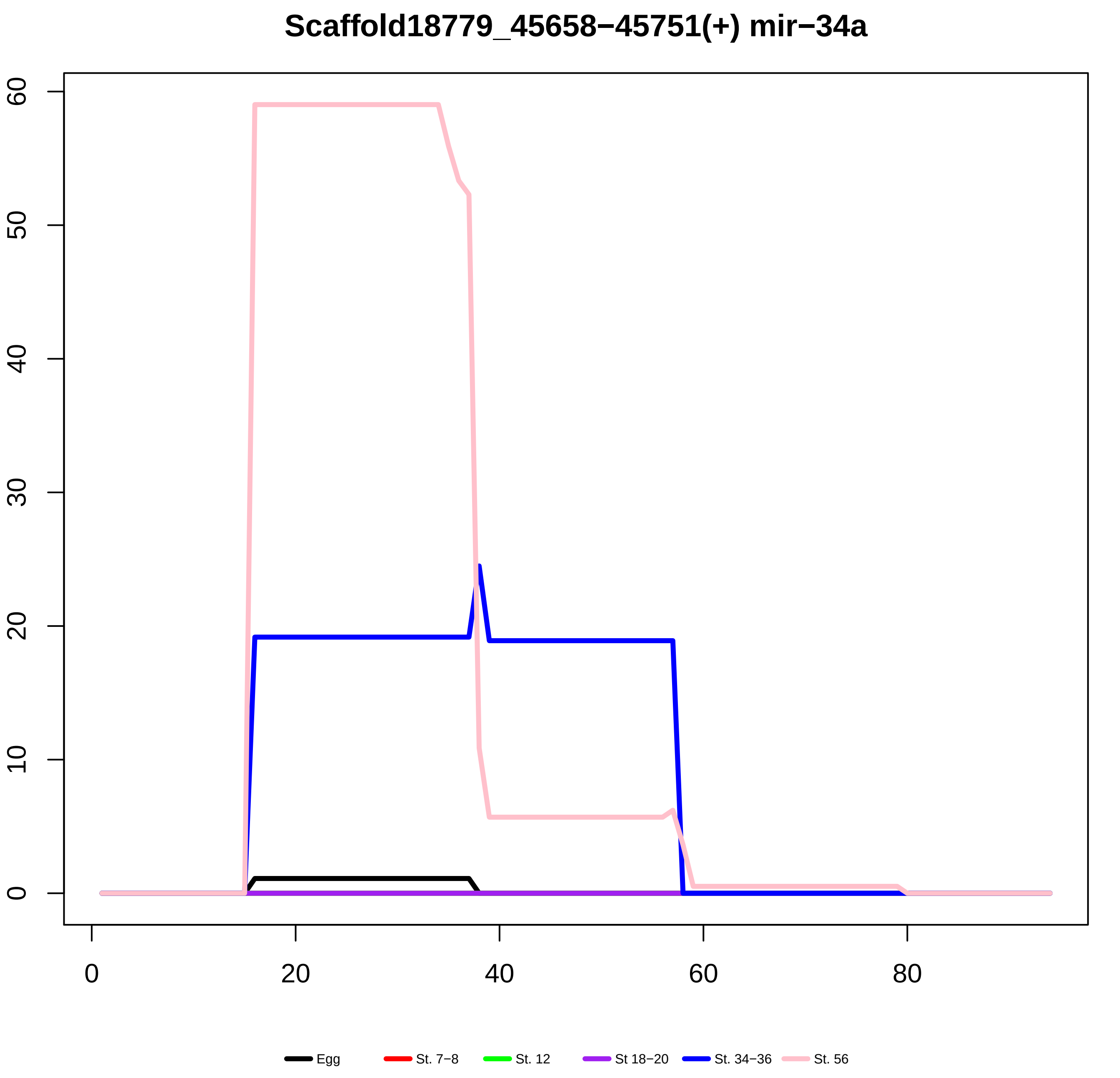

Scaffold18779_45658−45751(+) mir−34a
0
10
20
30
40
50
60
0
20
40
60
80
Egg
St. 7−8
St. 12
St 18−20
St. 34−36
St. 56

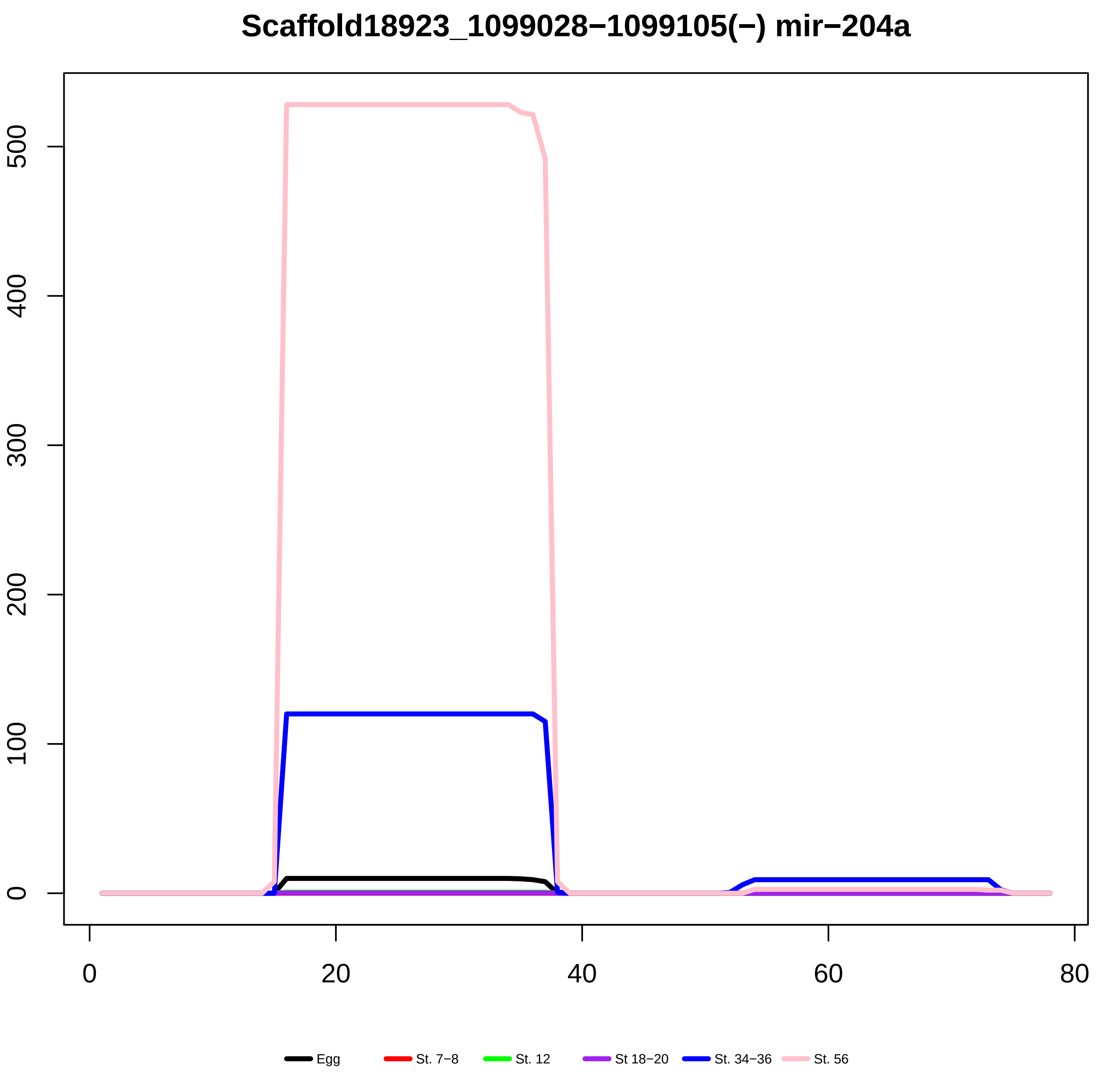

Scaffold18923_1099028−1099105(−) mir−204a
0
100
200
300
400
500
0
20
40
60
80
Egg
St. 7−8
St. 12
St 18−20
St. 34−36
St. 56

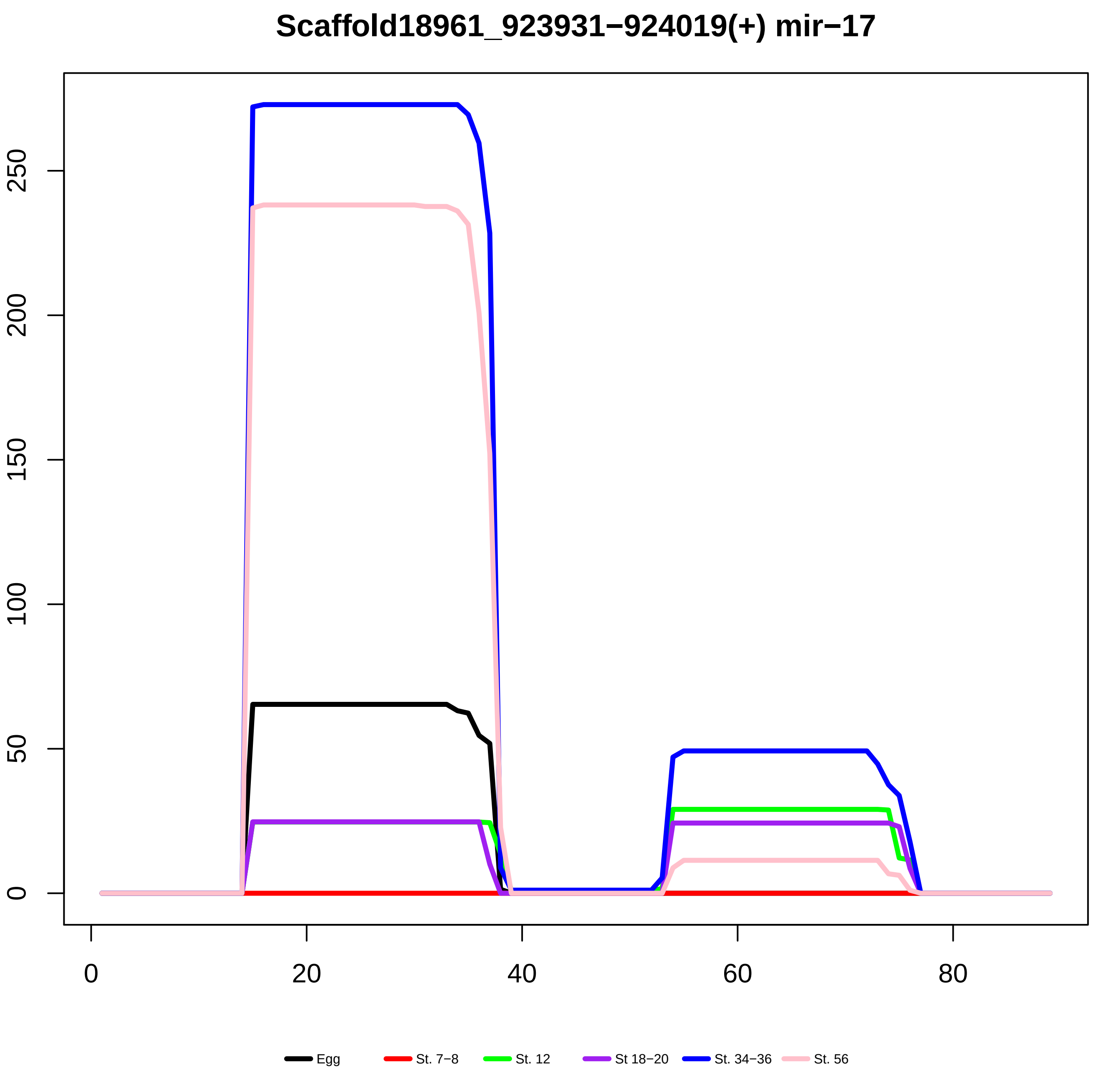
Scaffold18961_923931−924019(+) mir−17
0
50
100
150
200
250
0
20
40
60
80
Egg
St. 7−8
St. 12
St 18−20
St. 34−36
St. 56

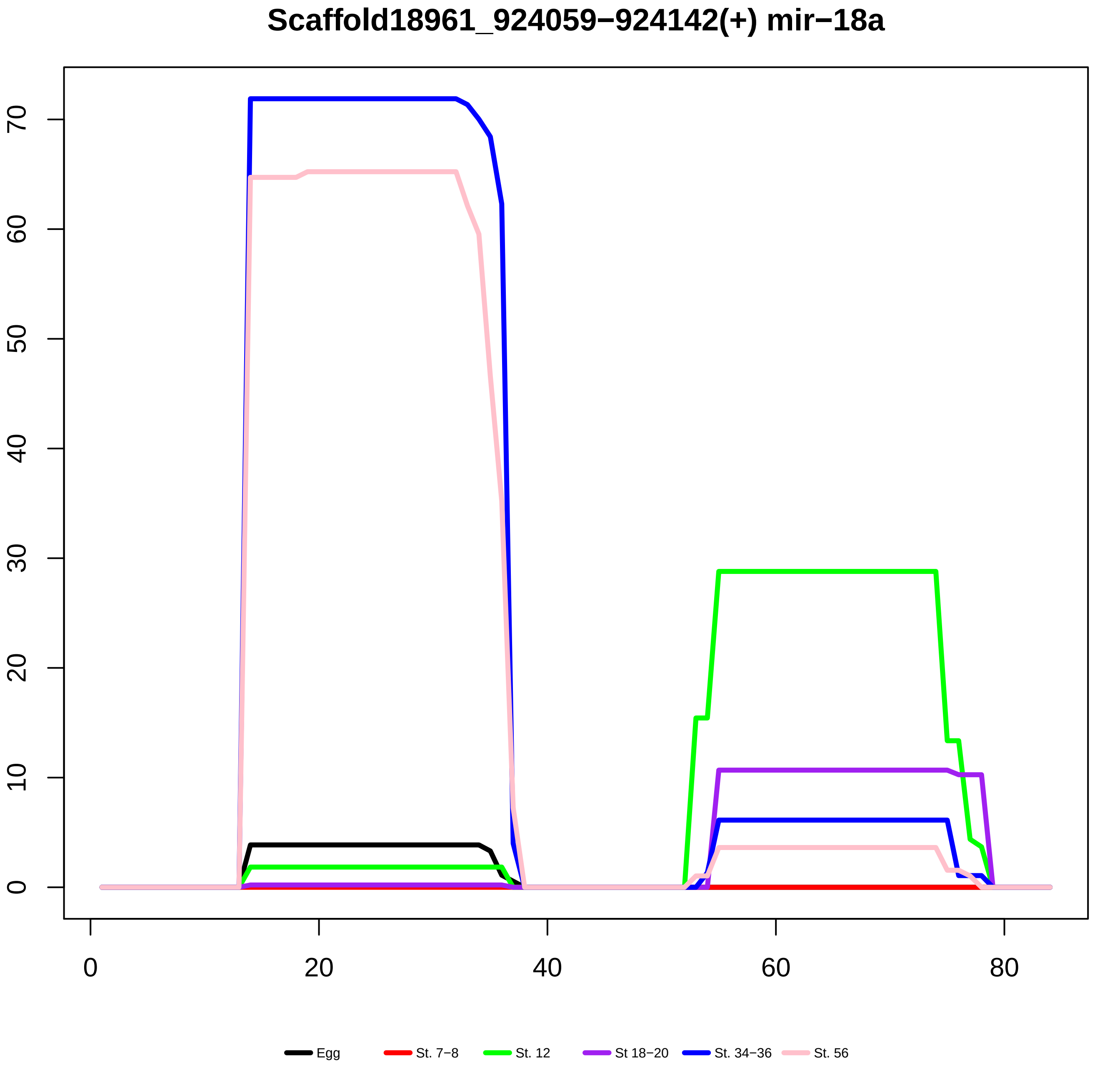

Scaffold18961_924059−924142(+) mir−18a
0
10
20
30
40
50
60
70
0
20
40
60
80
Egg
St. 7−8
St. 12
St 18−20
St. 34−36
St. 56

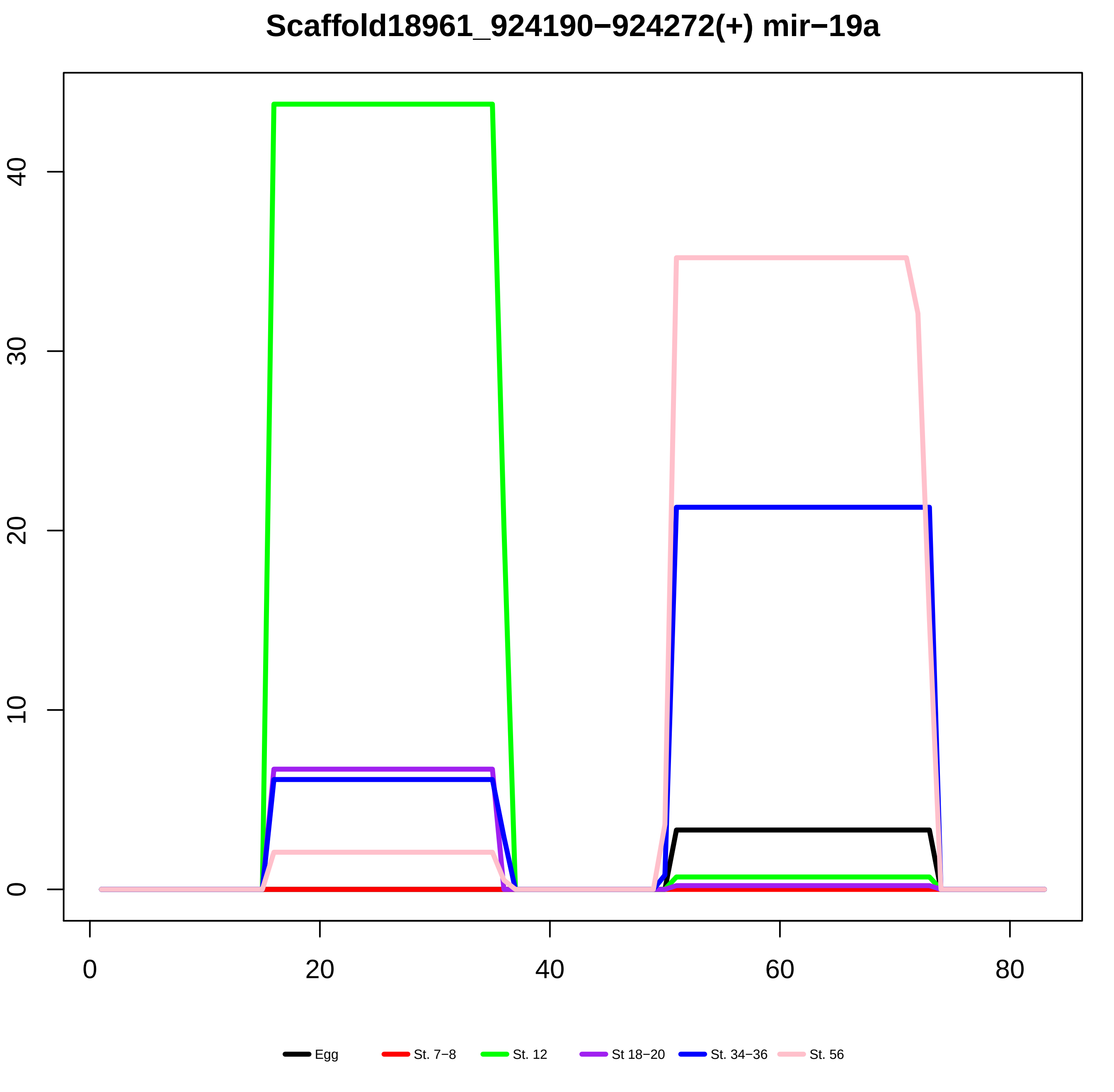

Scaffold18961_924190−924272(+) mir−19a
0
10
20
30
40
0
20
40
60
80
Egg
St. 7−8
St. 12
St 18−20
St. 34−36
St. 56

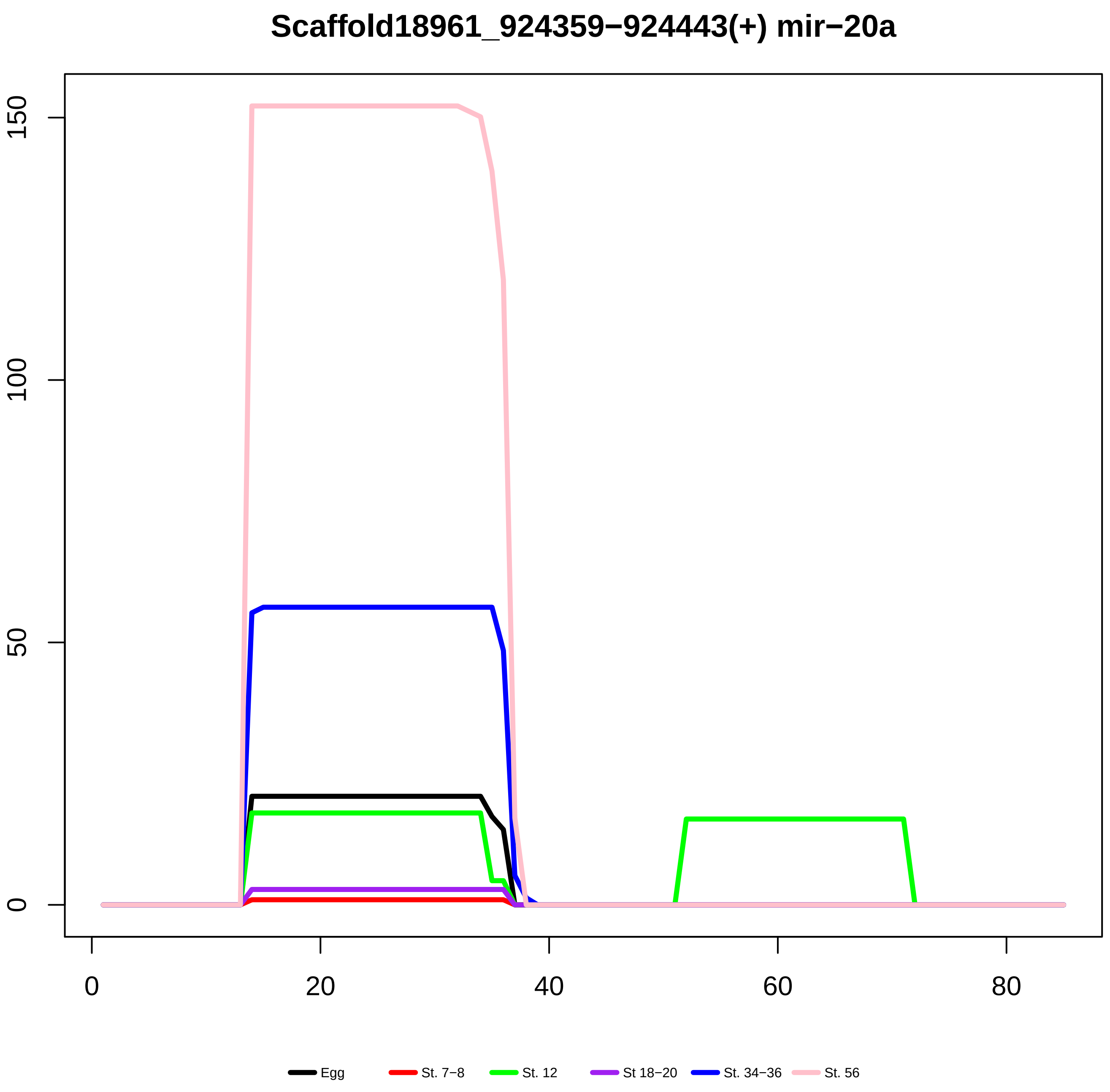

Scaffold18961_924359−924443(+) mir−20a
150
100
50
0
0
20
40
60
80
Egg
St. 7−8
St. 12
St 18−20
St. 34−36
St. 56

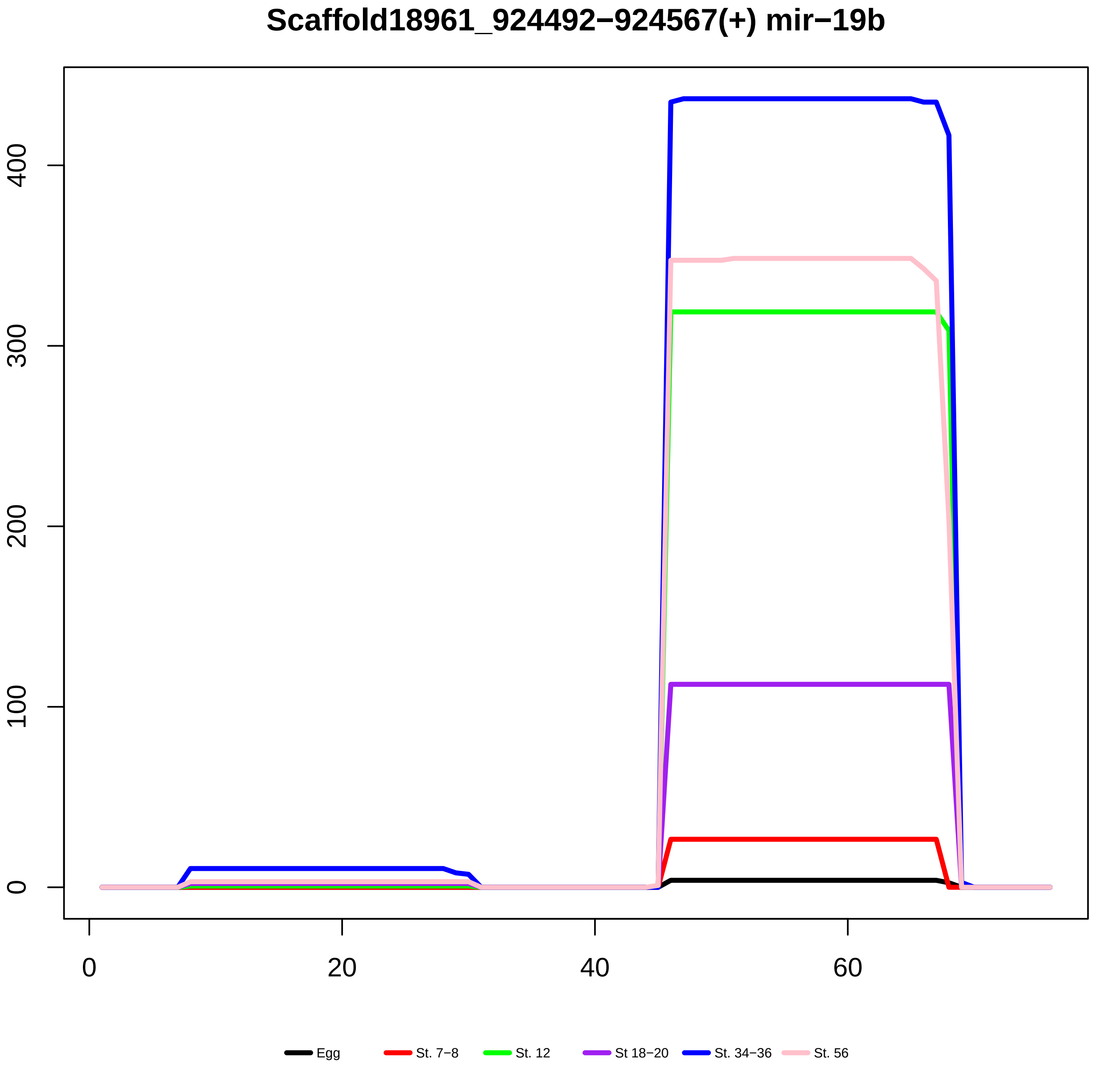
Scaffold18961_924492−924567(+) mir−19b
0
100
200
300
400
0
20
40
60
Egg
St. 7−8
St. 12
St 18−20
St. 34−36
St. 56

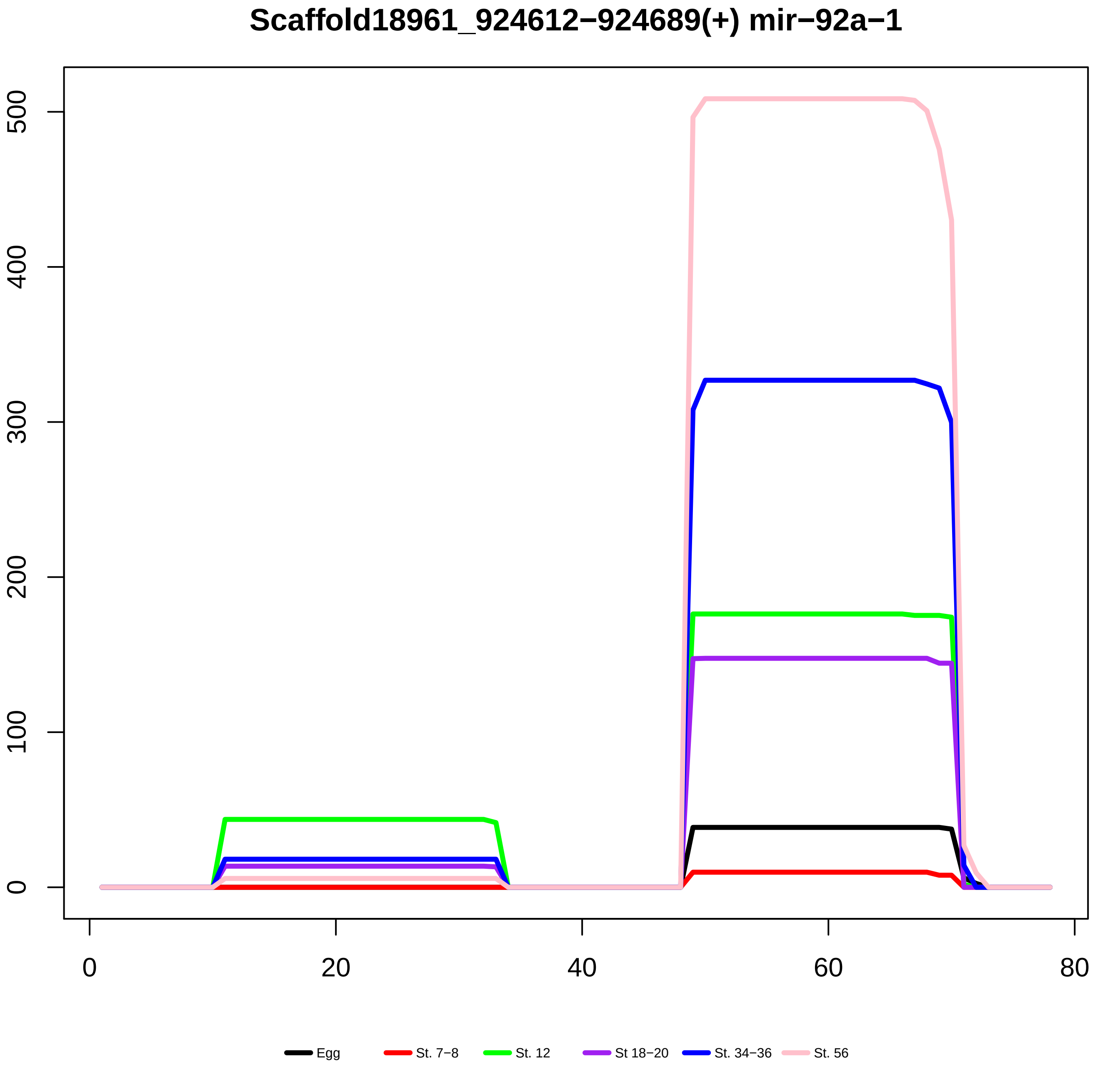

Scaffold18961_924612−924689(+) mir−92a−1
0
100
200
300
400
500
0
20
40
60
80
Egg
St. 7−8
St. 12
St 18−20
St. 34−36
St. 56

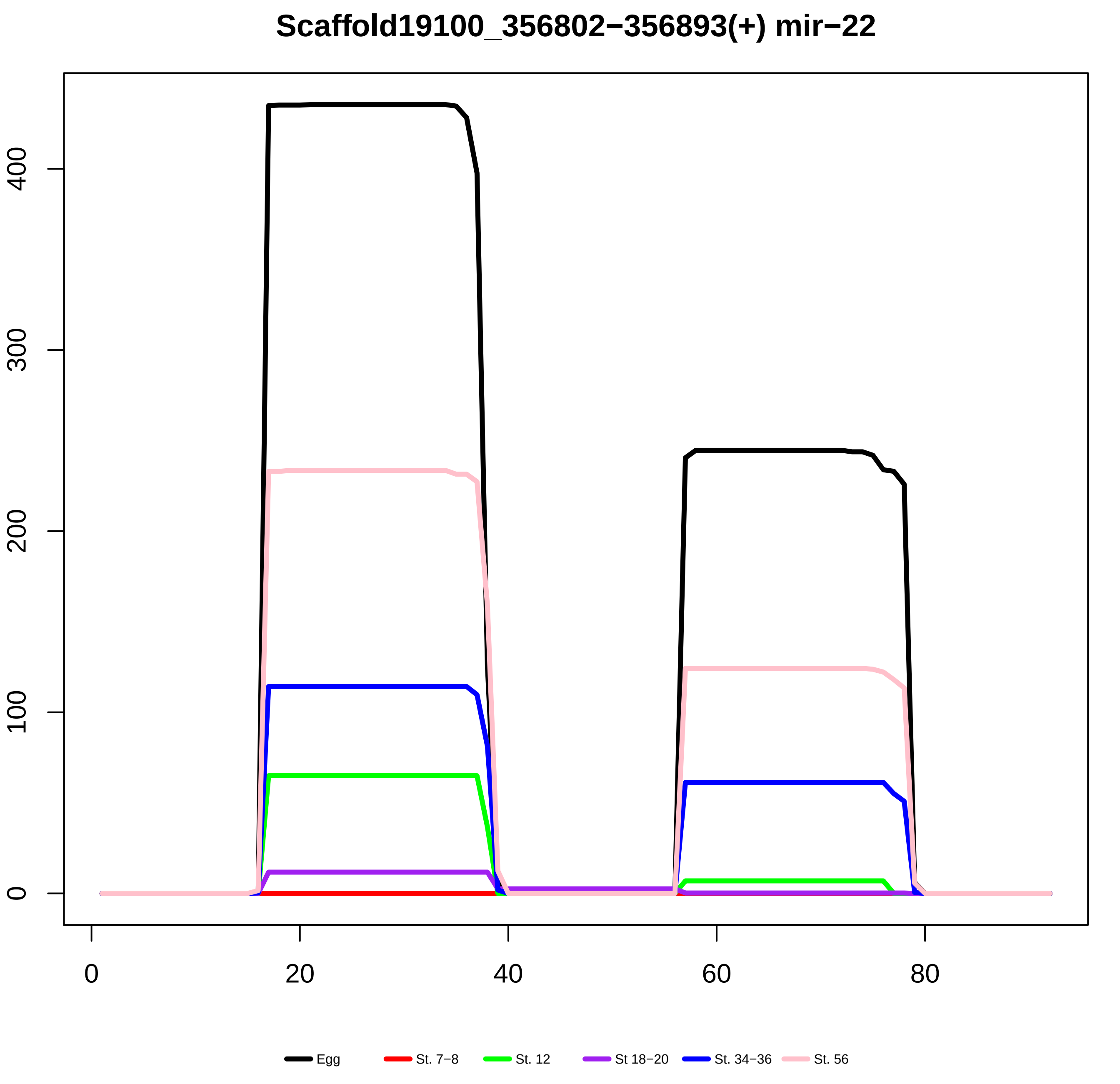
Scaffold19100_356802−356893(+) mir−22
0
100
200
300
400
0
20
40
60
80
Egg
St. 7−8
St. 12
St 18−20
St. 34−36
St. 56

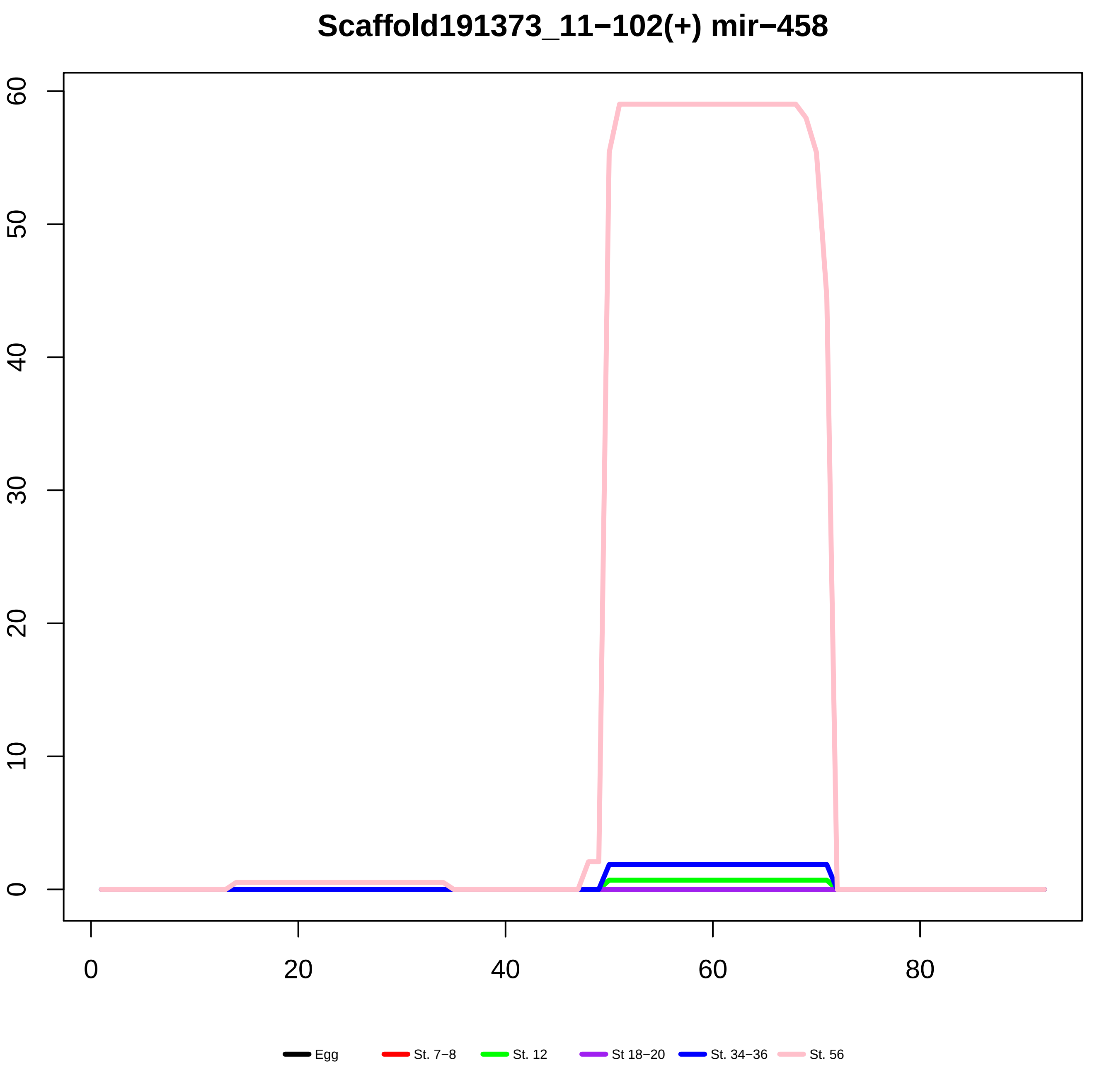
Scaffold191373_11−102(+) mir−458
0
10
20
30
40
50
60
0
20
40
60
80
Egg
St. 7−8
St. 12
St 18−20
St. 34−36
St. 56

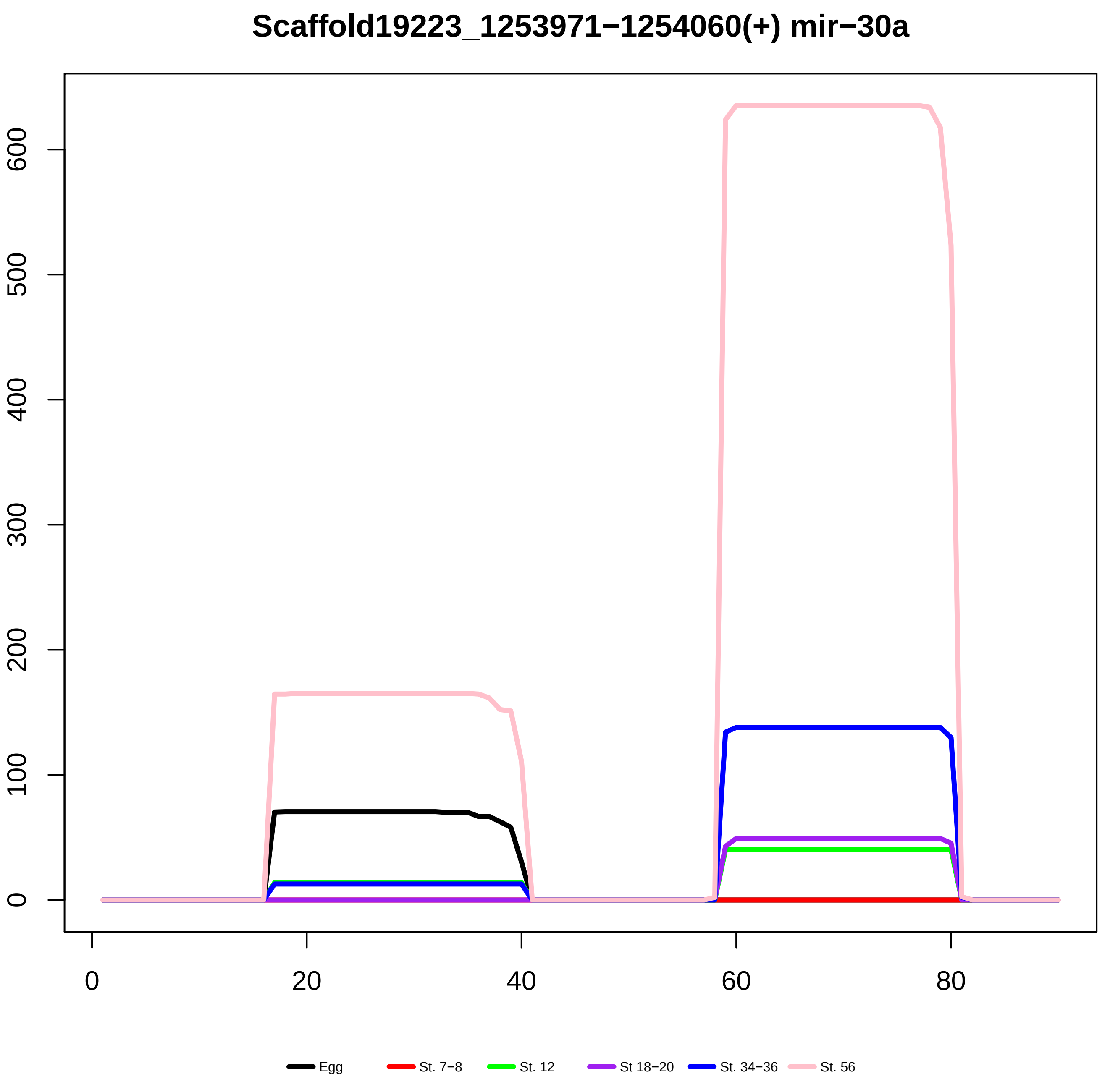

Scaffold19223_1253971−1254060(+) mir−30a
0
100
200
300
400
500
600
0
20
40
60
80
Egg
St. 7−8
St. 12
St 18−20
St. 34−36
St. 56

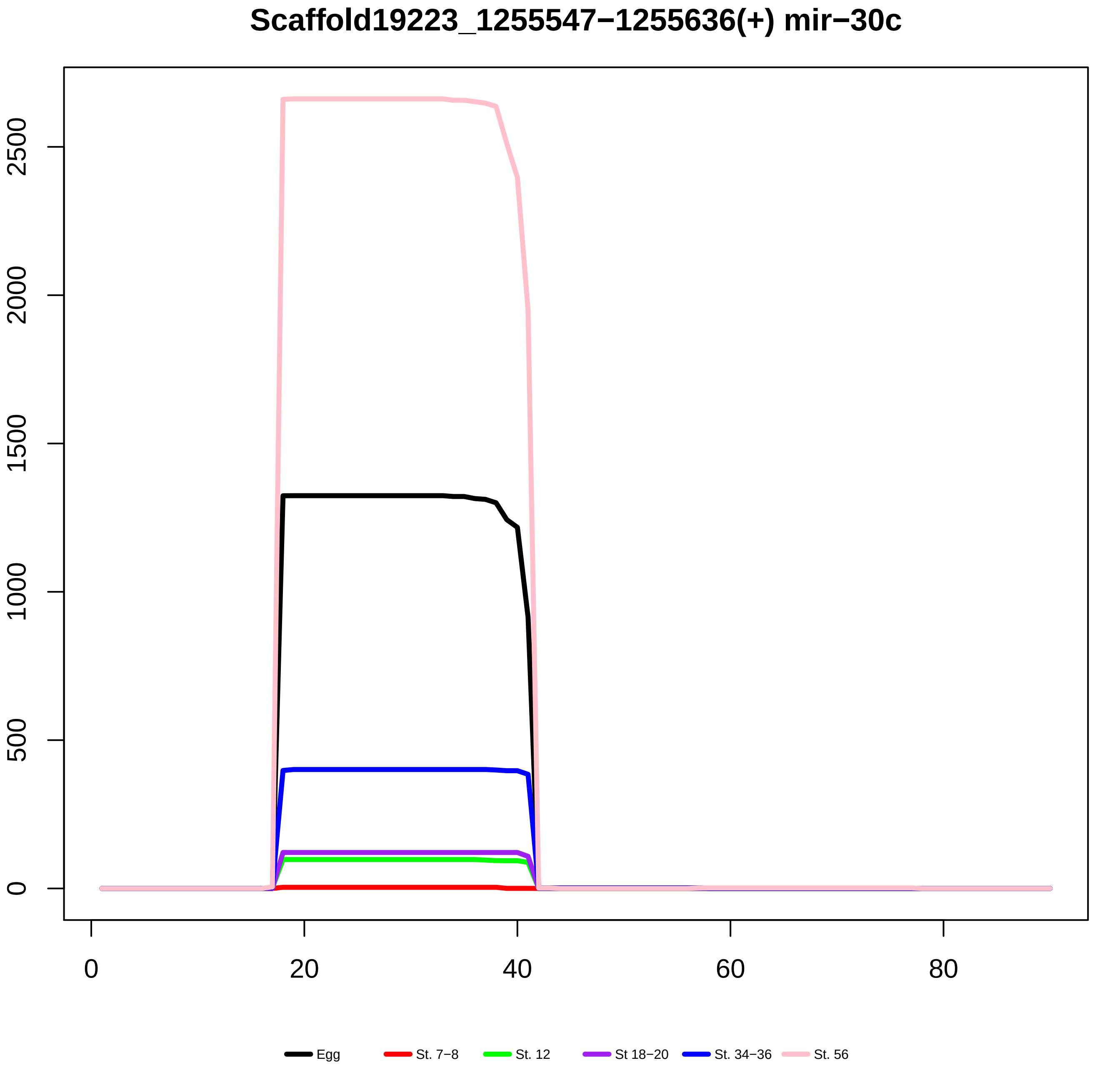
Scaffold19223_1255547−1255636(+) mir−30c
0
500
1000
1500
2000
2500
0
20
40
60
80
Egg
St. 7−8
St. 12
St 18−20
St. 34−36
St. 56

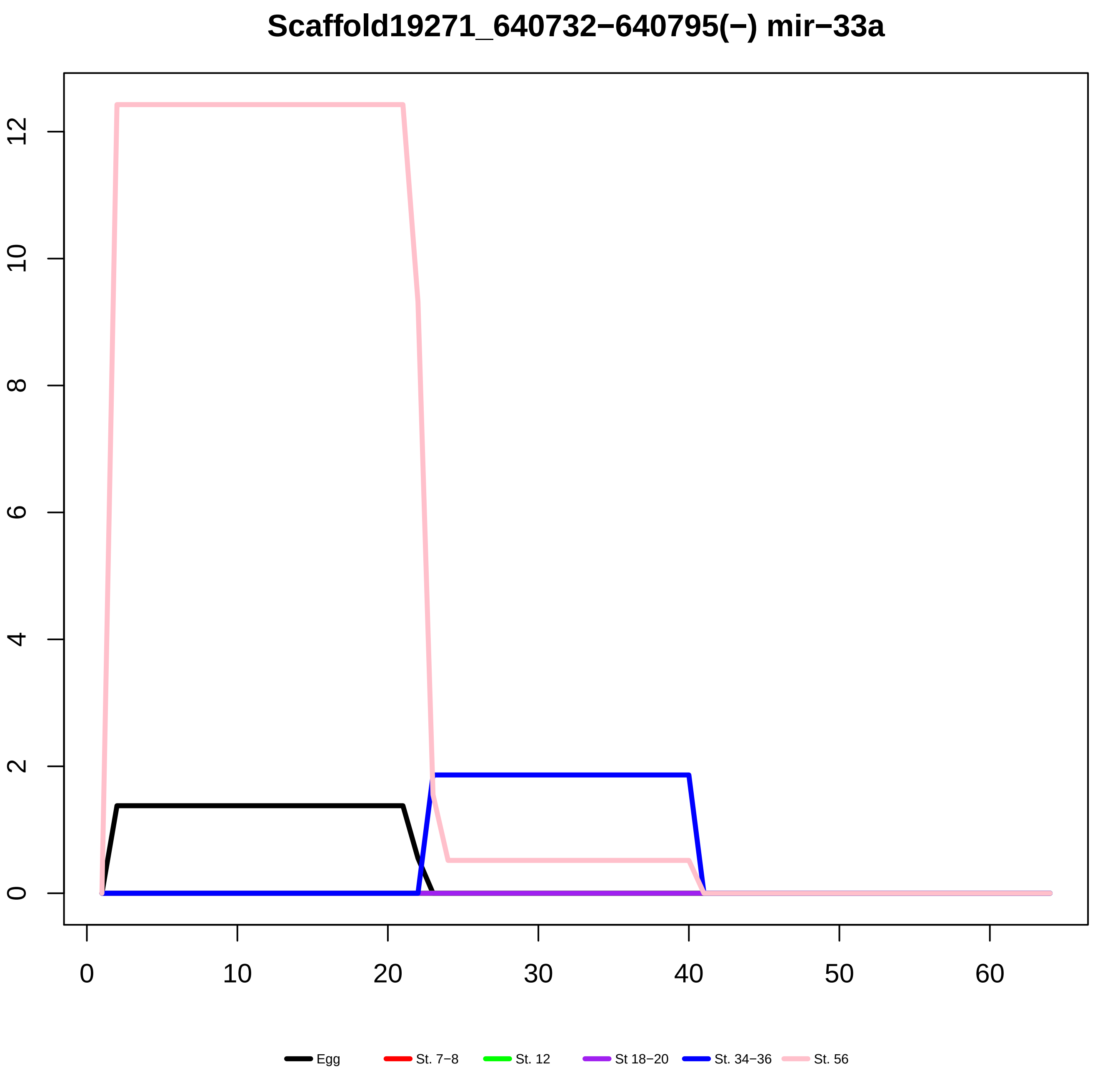

Scaffold19271_640732−640795(−) mir−33a
0
2
4
6
8
10
12
0
10
20
30
40
50
60
Egg
St. 7−8
St. 12
St 18−20
St. 34−36
St. 56

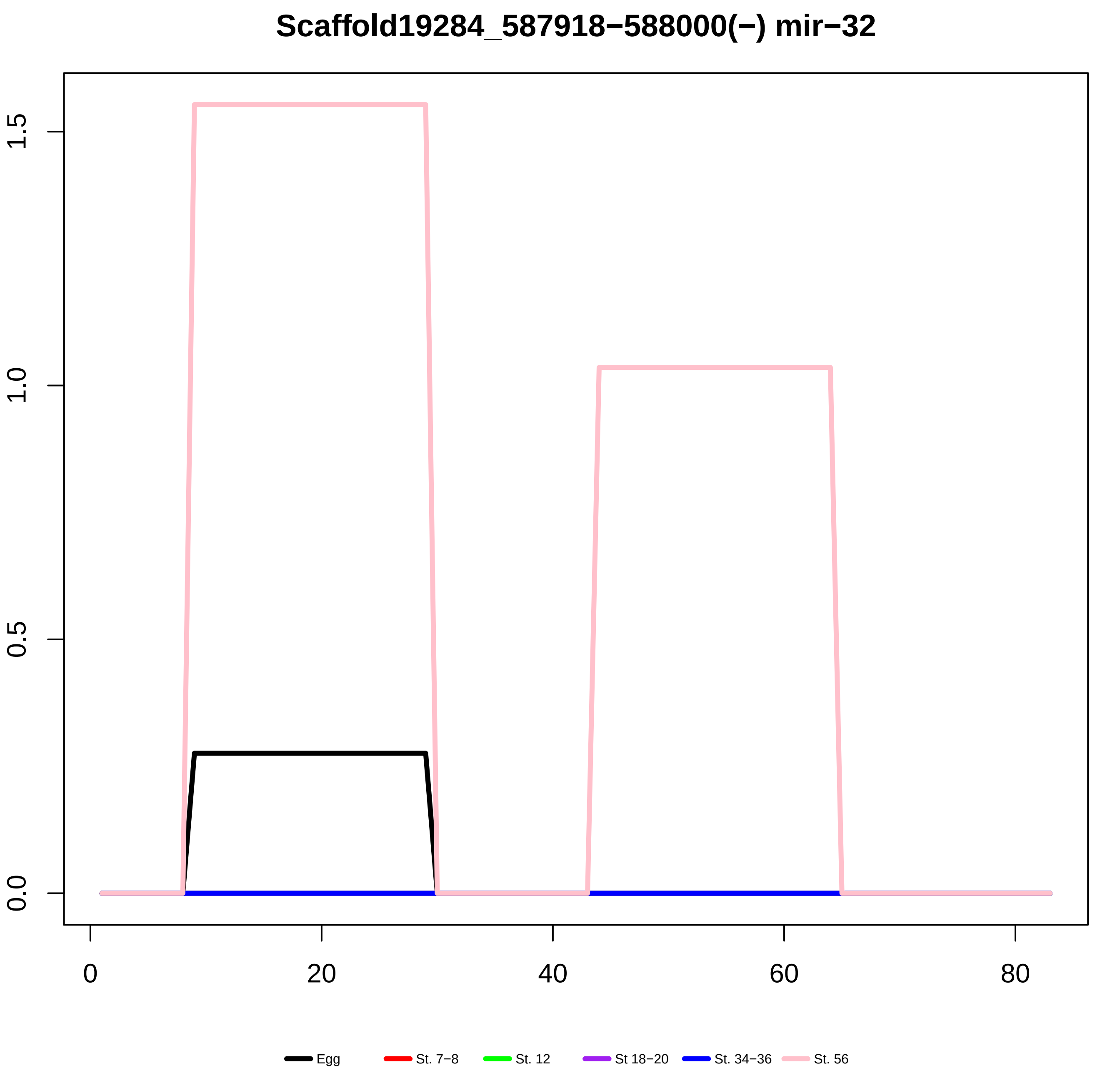

Scaffold19284_587918−588000(−) mir−32
0.0
0.5
1.0
1.5
0
20
40
60
80
Egg
St. 7−8
St. 12
St 18−20
St. 34−36
St. 56

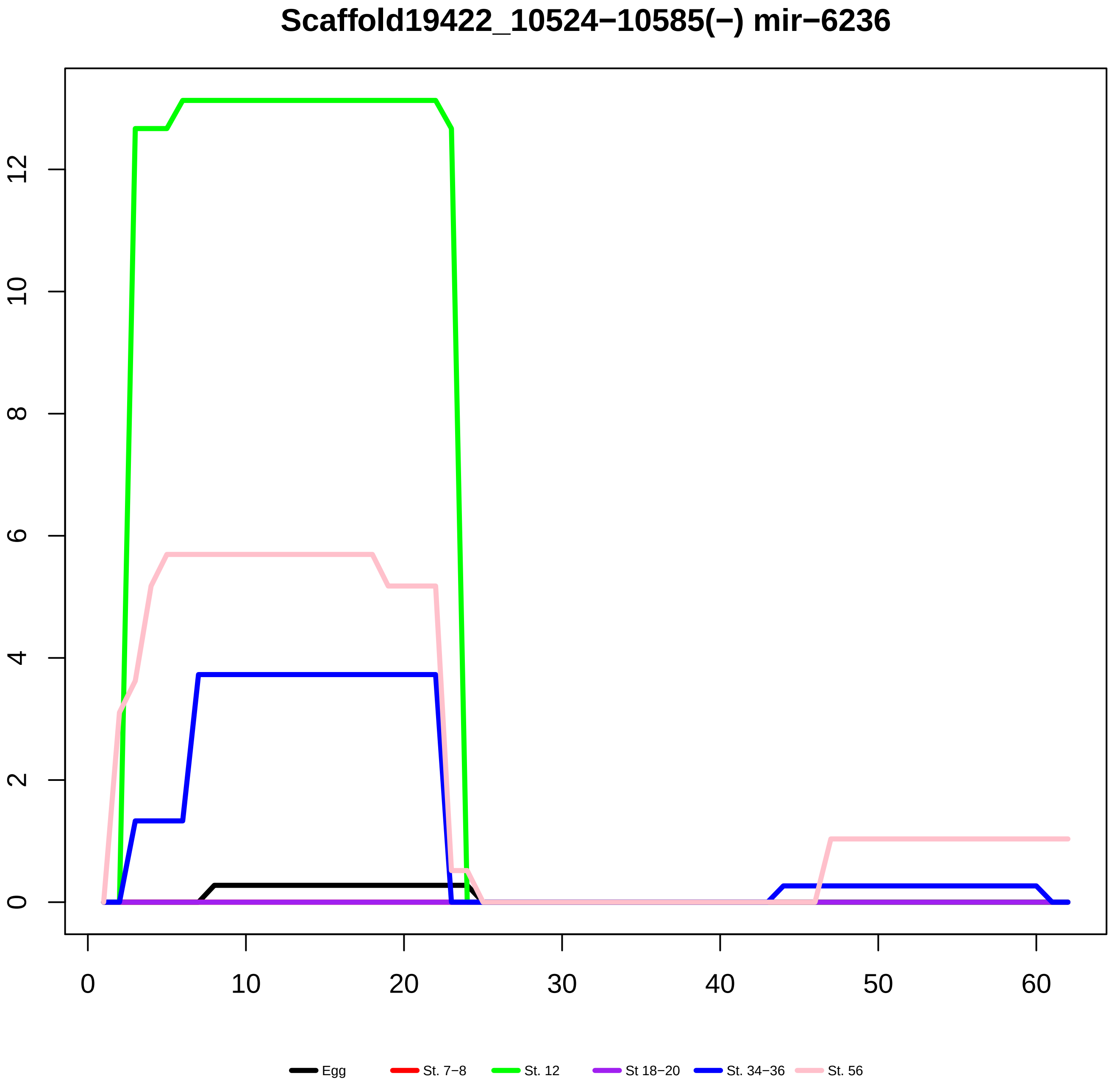

Scaffold19422_10524−10585(−) mir−6236
0
2
4
6
8
10
12
0
10
20
30
40
50
60
Egg
St. 7−8
St. 12
St 18−20
St. 34−36
St. 56

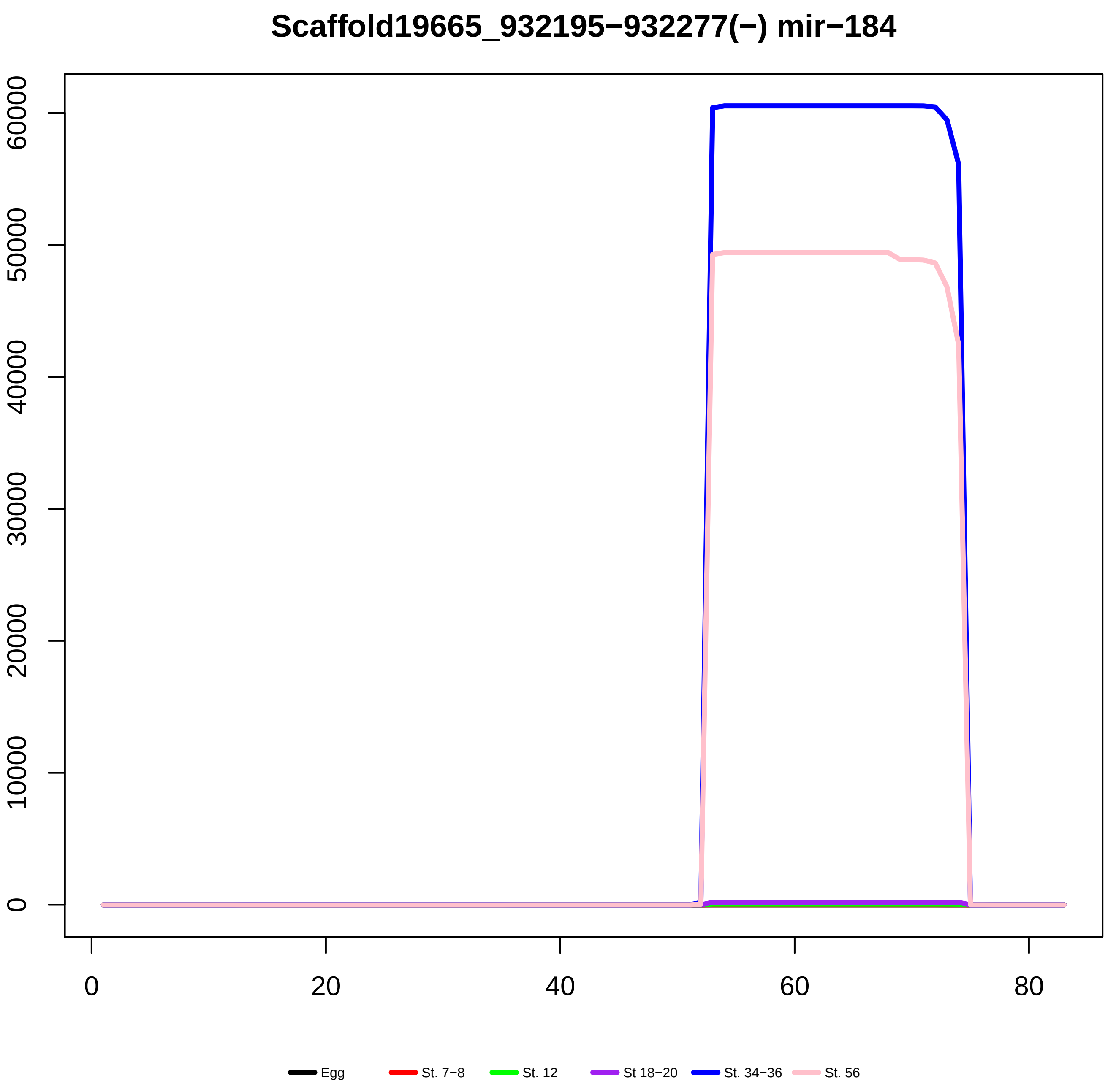
Scaffold19665_932195−932277(−) mir−184
60000
50000
40000
30000
20000
10000
0
0
20
40
60
80
Egg
St. 7−8
St. 12
St 18−20
St. 34−36
St. 56

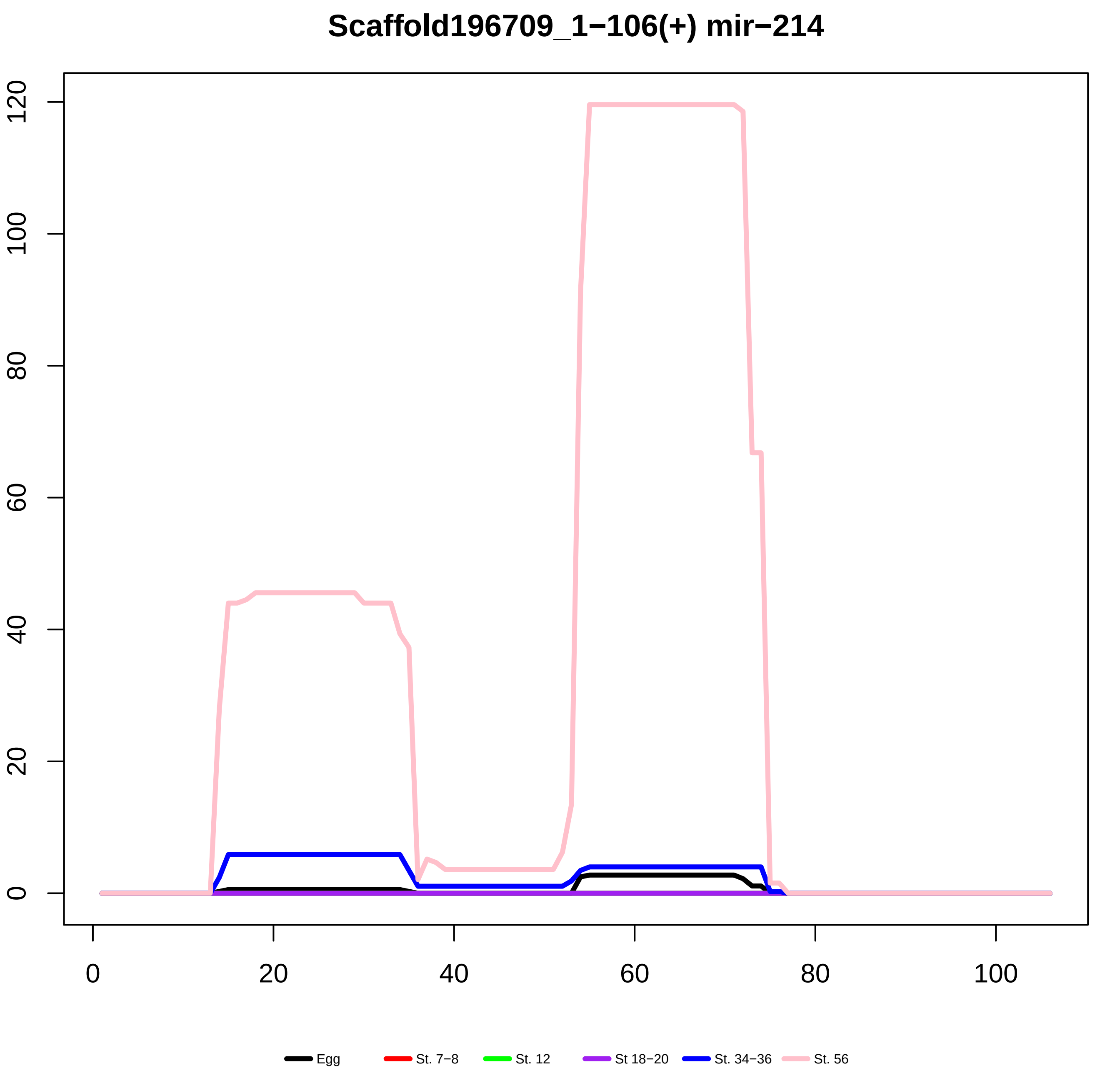

Scaffold196709_1−106(+) mir−214
0
20
40
60
80
100
120
0
20
40
60
80
100
Egg
St. 7−8
St. 12
St 18−20
St. 34−36
St. 56

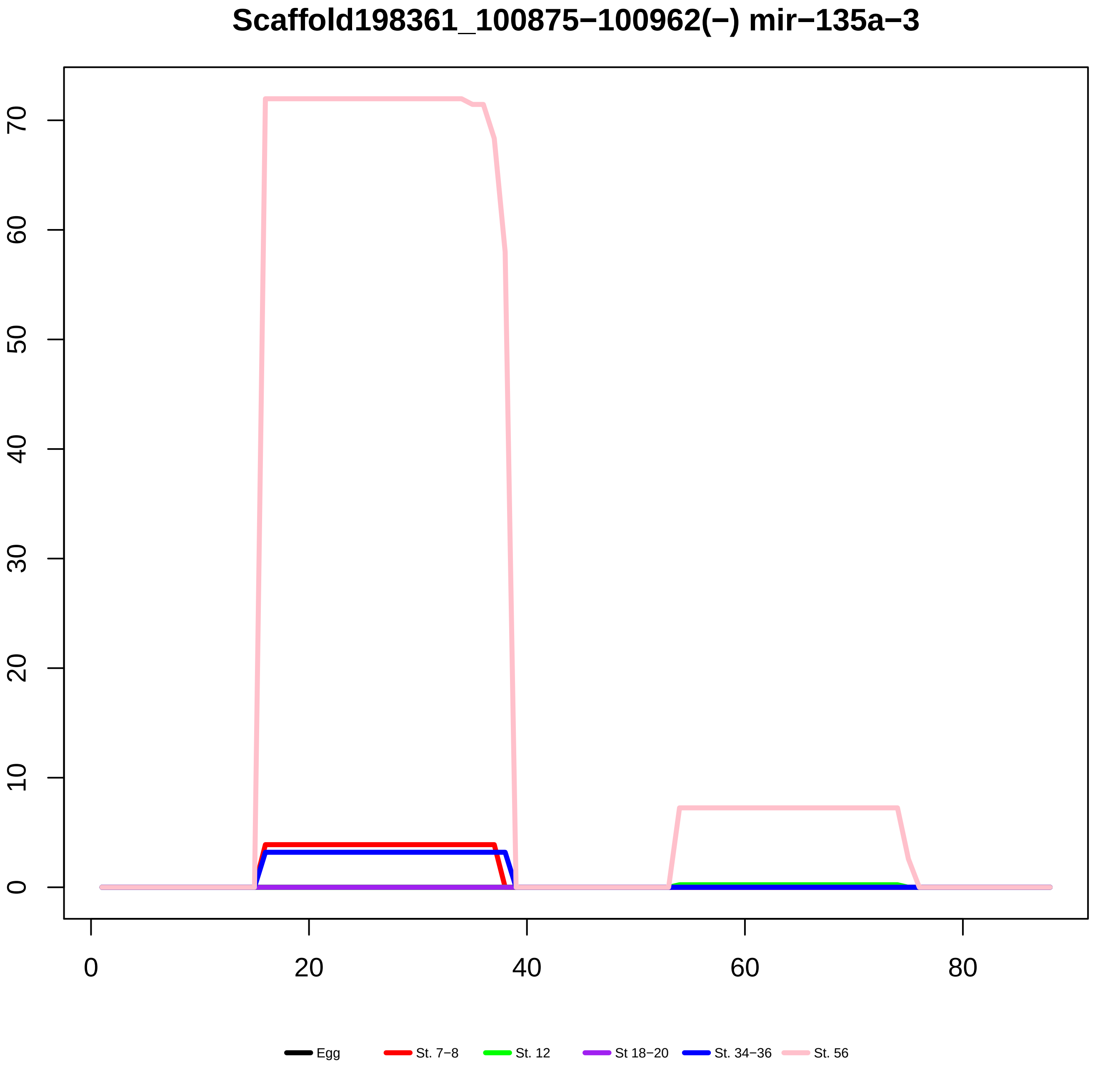
Scaffold198361_100875−100962(−) mir−135a−3
0
10
20
30
40
50
60
70
0
20
40
60
80
Egg
St. 7−8
St. 12
St 18−20
St. 34−36
St. 56

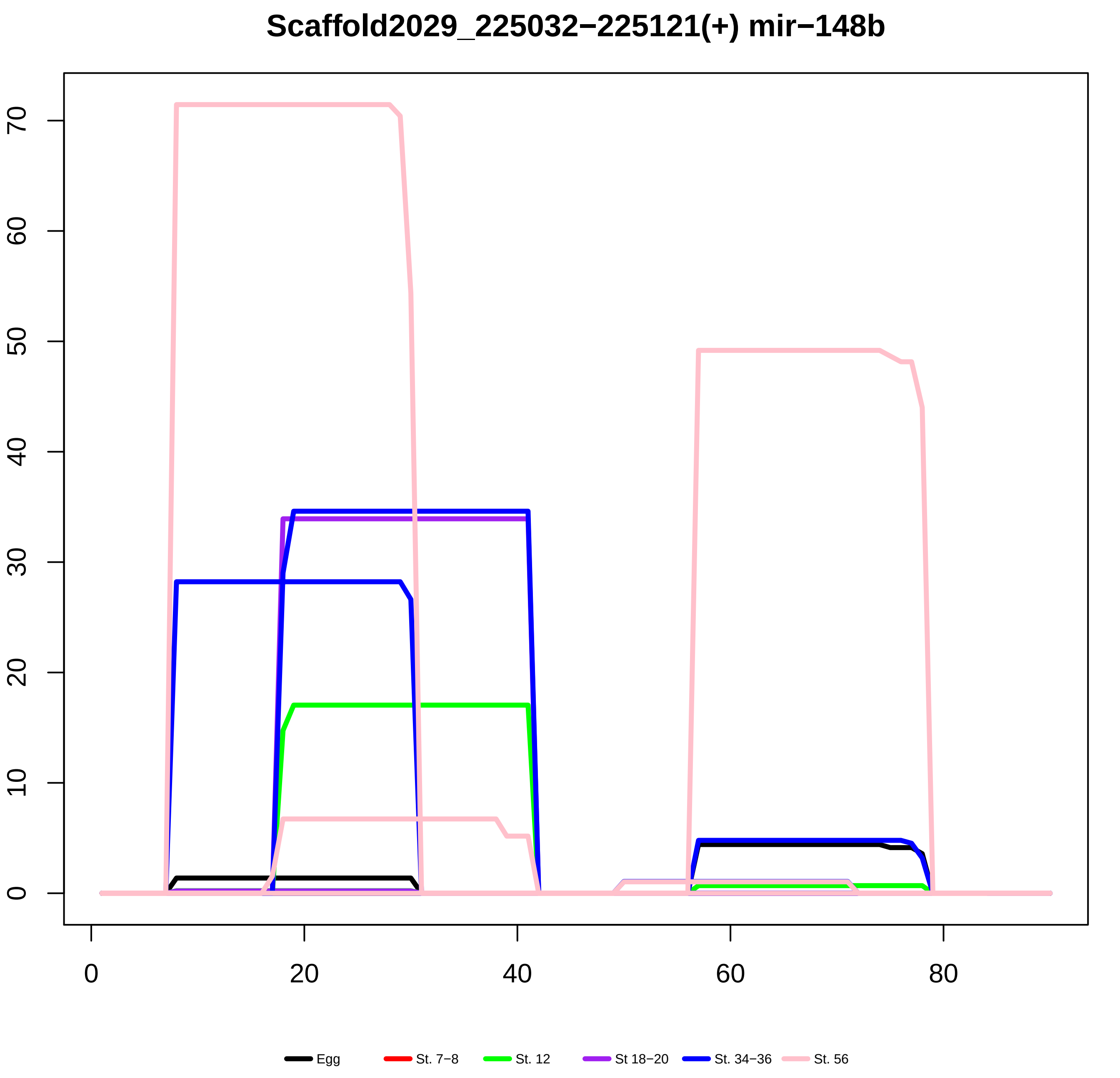
Scaffold2029_225032−225121(+) mir−148b
0
10
20
30
40
50
60
70
0
20
40
60
80
Egg
St. 7−8
St. 12
St 18−20
St. 34−36
St. 56

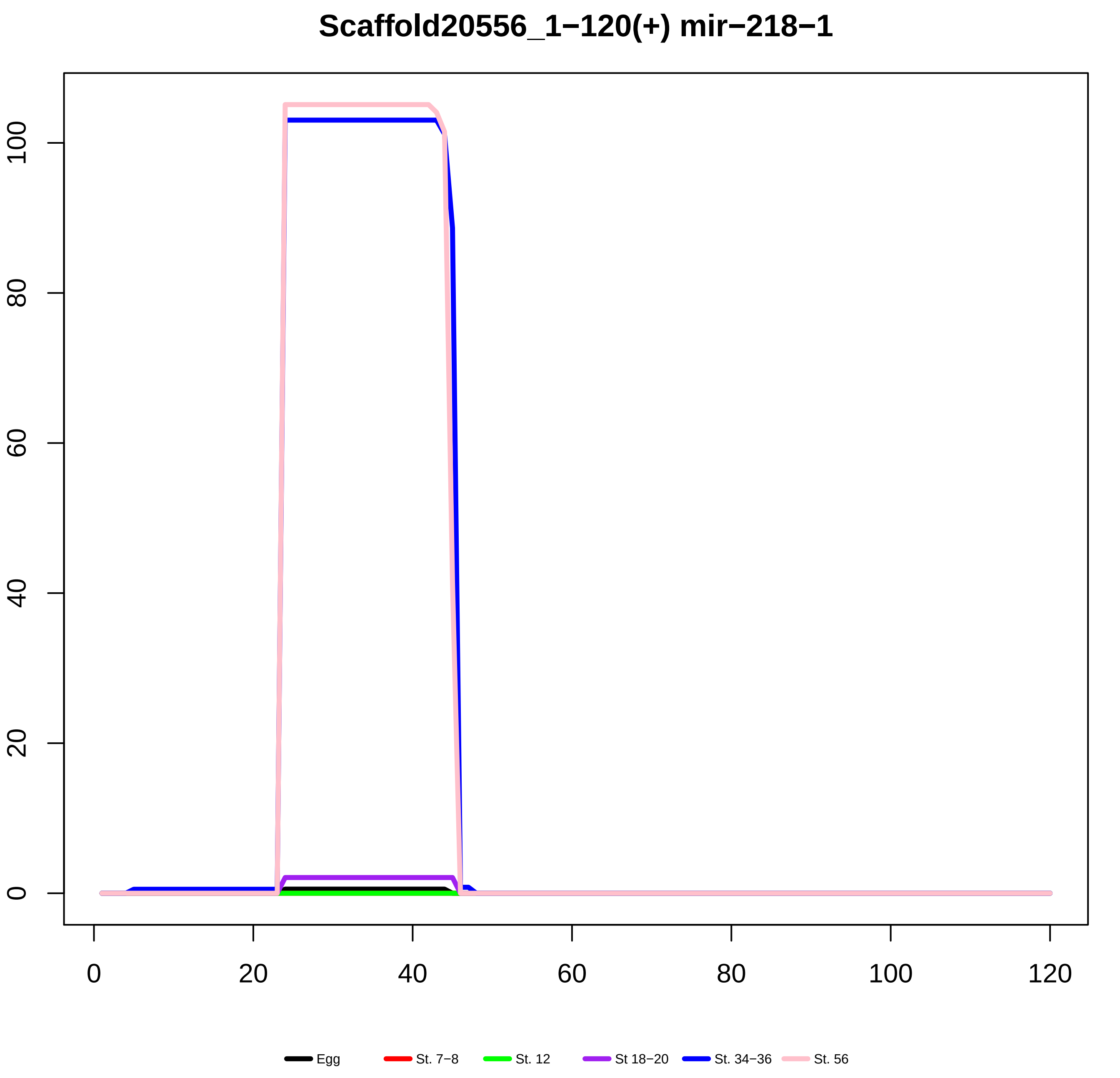

Scaffold20556_1−120(+) mir−218−1
0
20
40
60
80
100
0
20
40
60
80
100
120
Egg
St. 7−8
St. 12
St 18−20
St. 34−36
St. 56

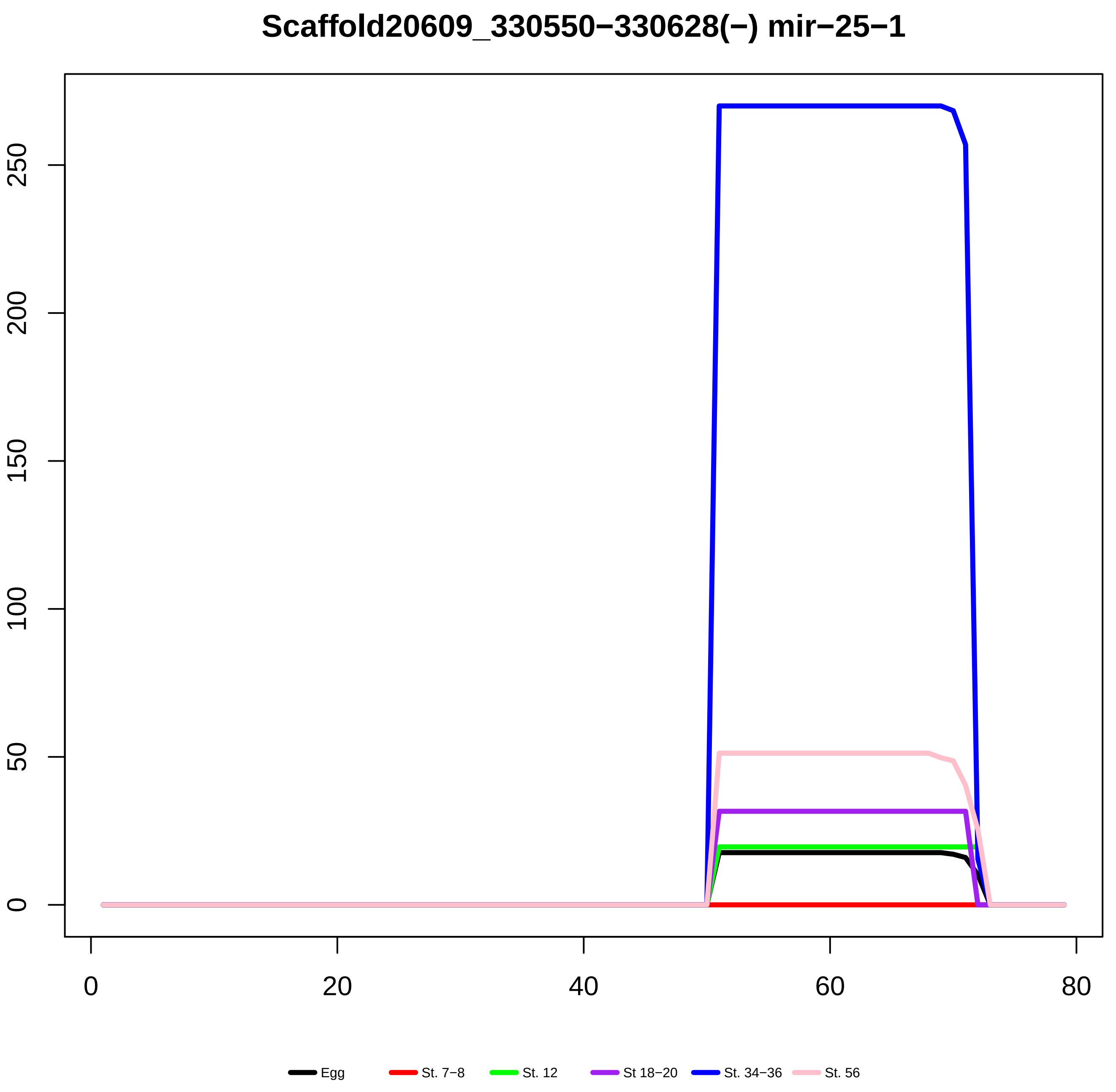
Scaffold20609_330550−330628(−) mir−25−1
0
50
100
150
200
250
0
20
40
60
80
Egg
St. 7−8
St. 12
St 18−20
St. 34−36
St. 56

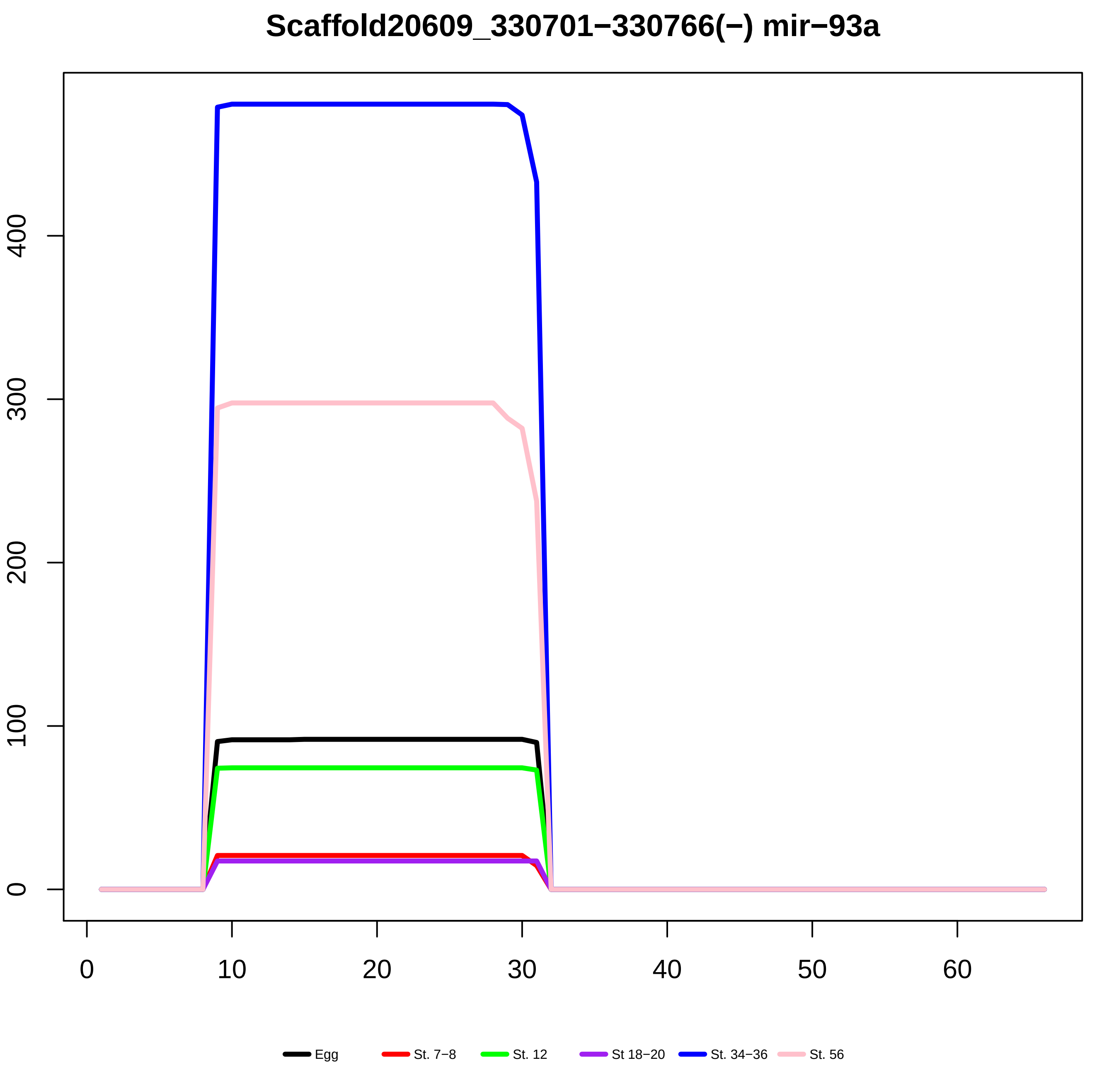

Scaffold20609_330701−330766(−) mir−93a
0
100
200
300
400
0
10
20
30
40
50
60
Egg
St. 7−8
St. 12
St 18−20
St. 34−36
St. 56

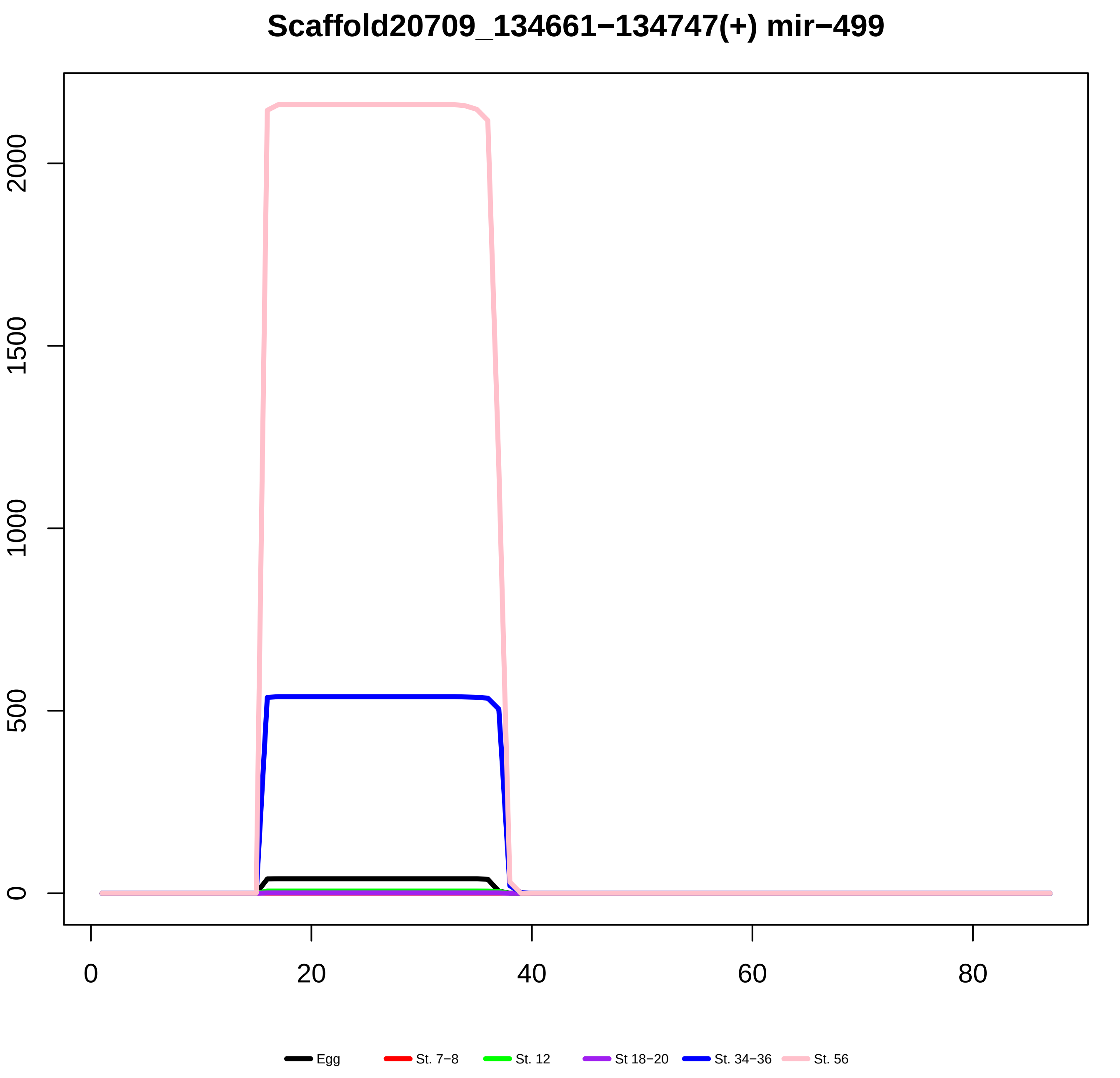
Scaffold20709_134661−134747(+) mir−499
0
500
1000
1500
2000
0
20
40
60
80
Egg
St. 7−8
St. 12
St 18−20
St. 34−36
St. 56

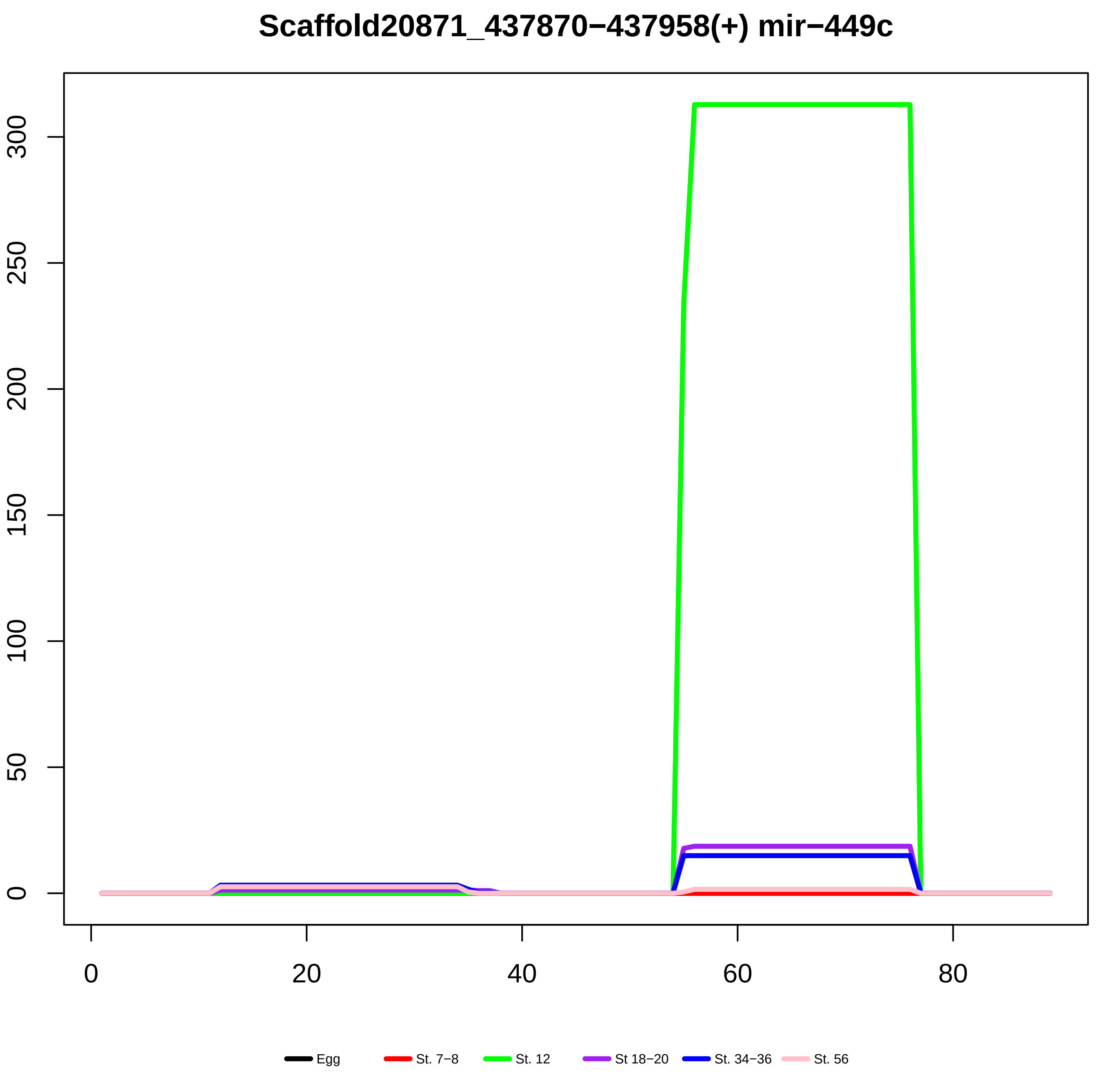
Scaffold20871_437870−437958(+) mir−449c
0
50
100
150
200
250
300
0
20
40
60
80
Egg
St. 7−8
St. 12
St 18−20
St. 34−36
St. 56

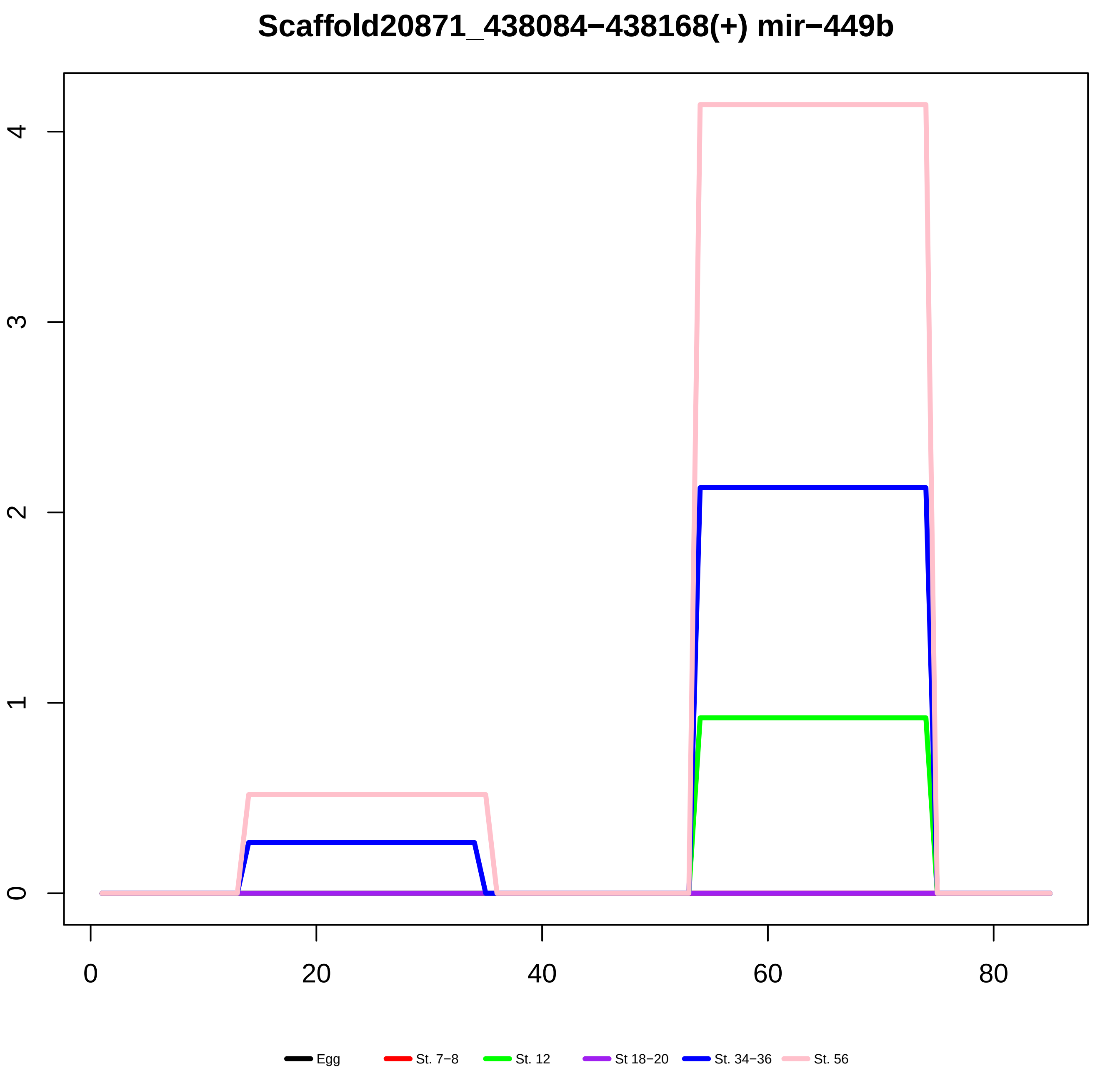
Scaffold20871_438084−438168(+) mir−449b
0
1
2
3
4
0
20
40
60
80
Egg
St. 7−8
St. 12
St 18−20
St. 34−36
St. 56

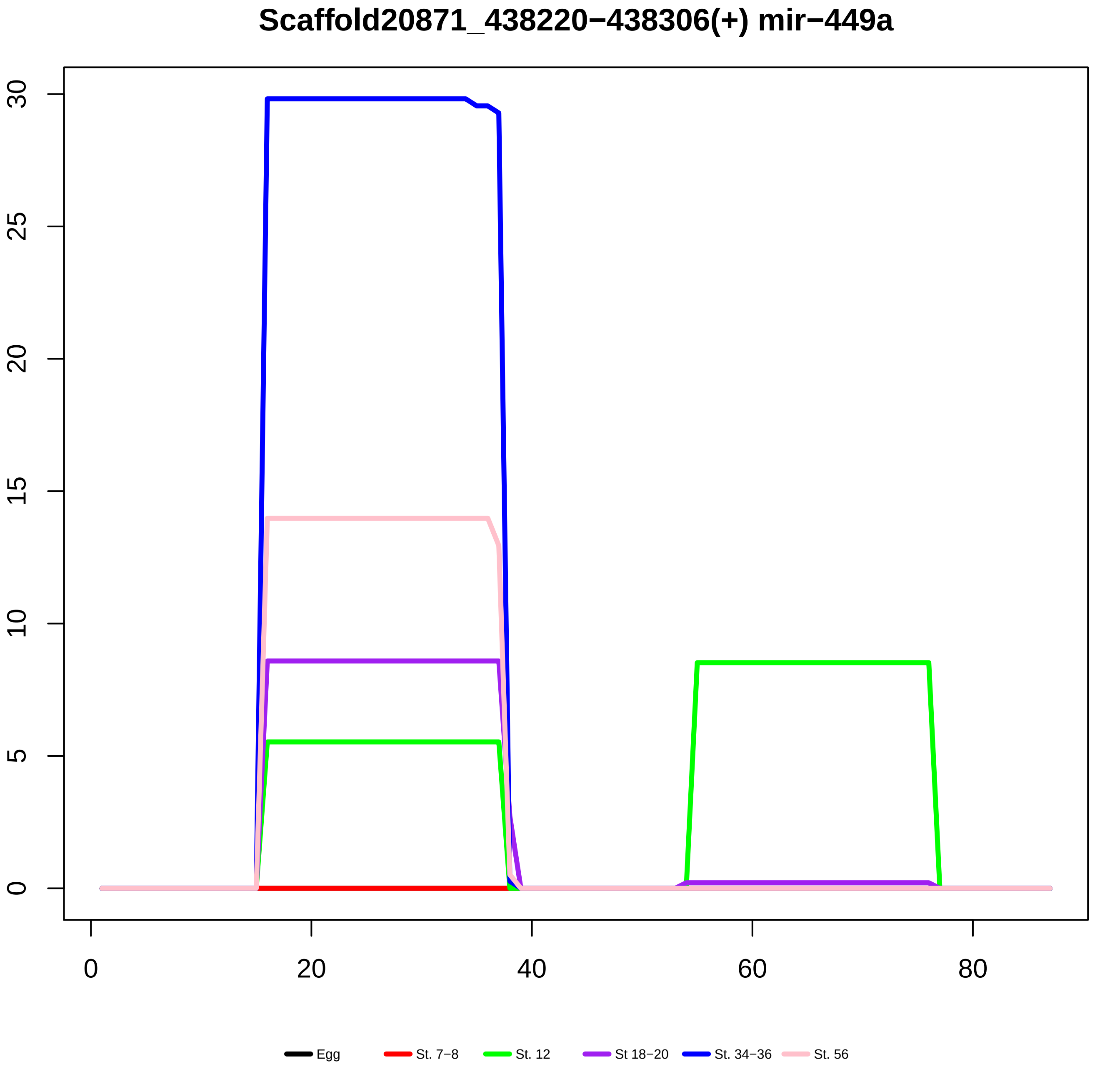
Scaffold20871_438220−438306(+) mir−449a
0
5
10
15
20
25
30
0
20
40
60
80
Egg
St. 7−8
St. 12
St 18−20
St. 34−36
St. 56

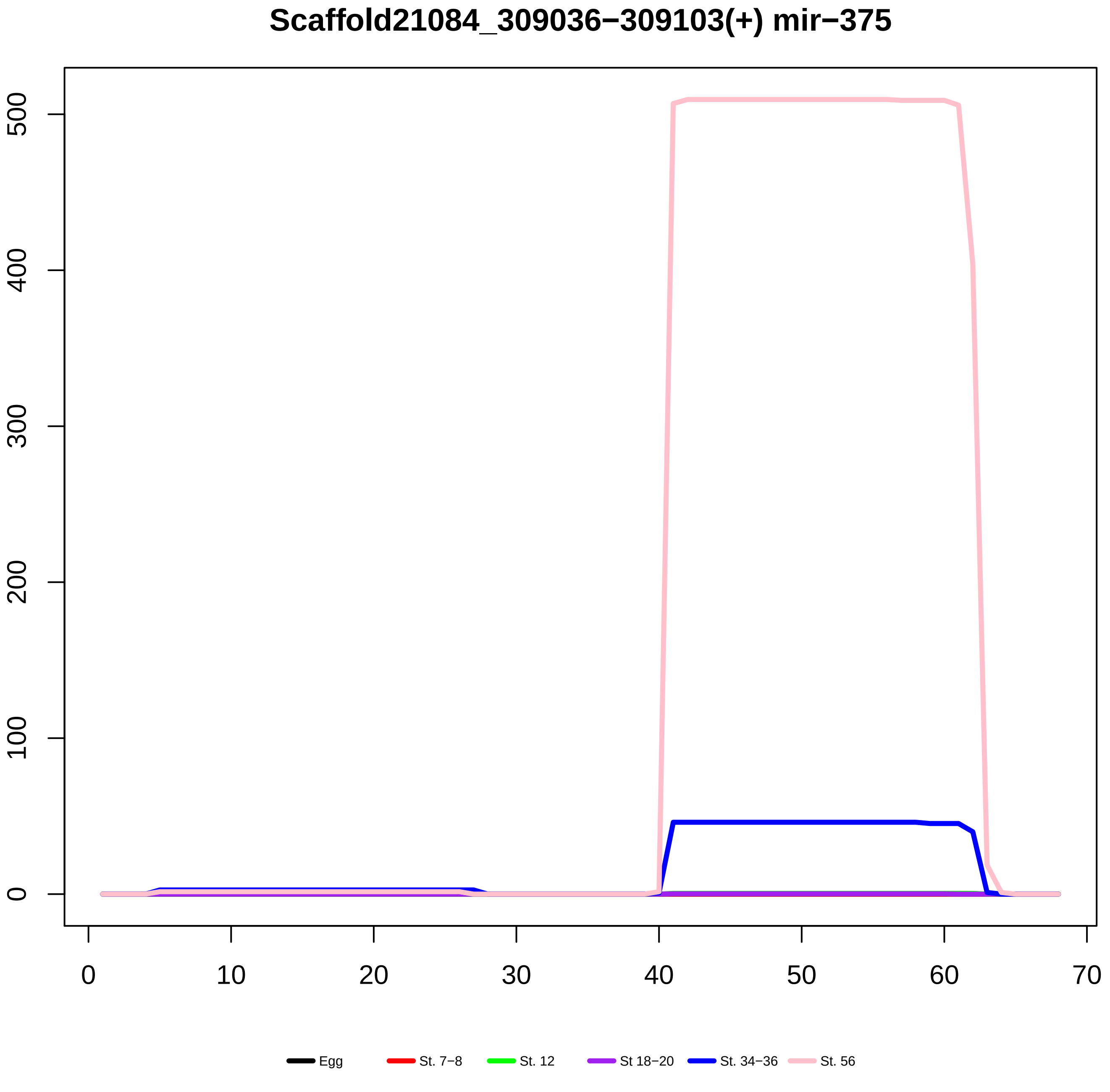

Scaffold21084_309036−309103(+) mir−375
0
100
200
300
400
500
0
10
20
30
40
50
60
70
Egg
St. 7−8
St. 12
St 18−20
St. 34−36
St. 56

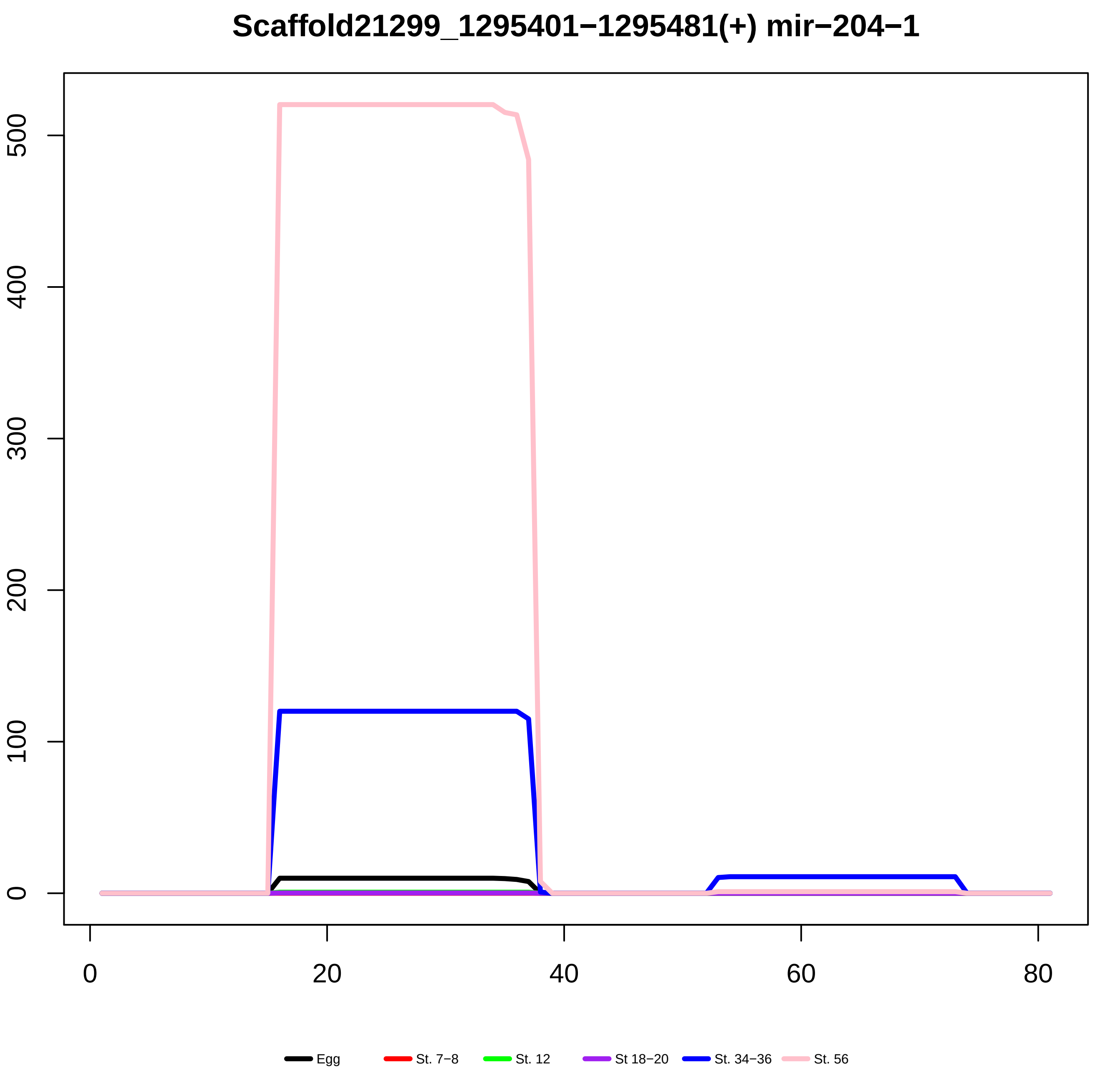
Scaffold21299_1295401−1295481(+) mir−204−1
0
100
200
300
400
500
0
20
40
60
80
Egg
St. 7−8
St. 12
St 18−20
St. 34−36
St. 56

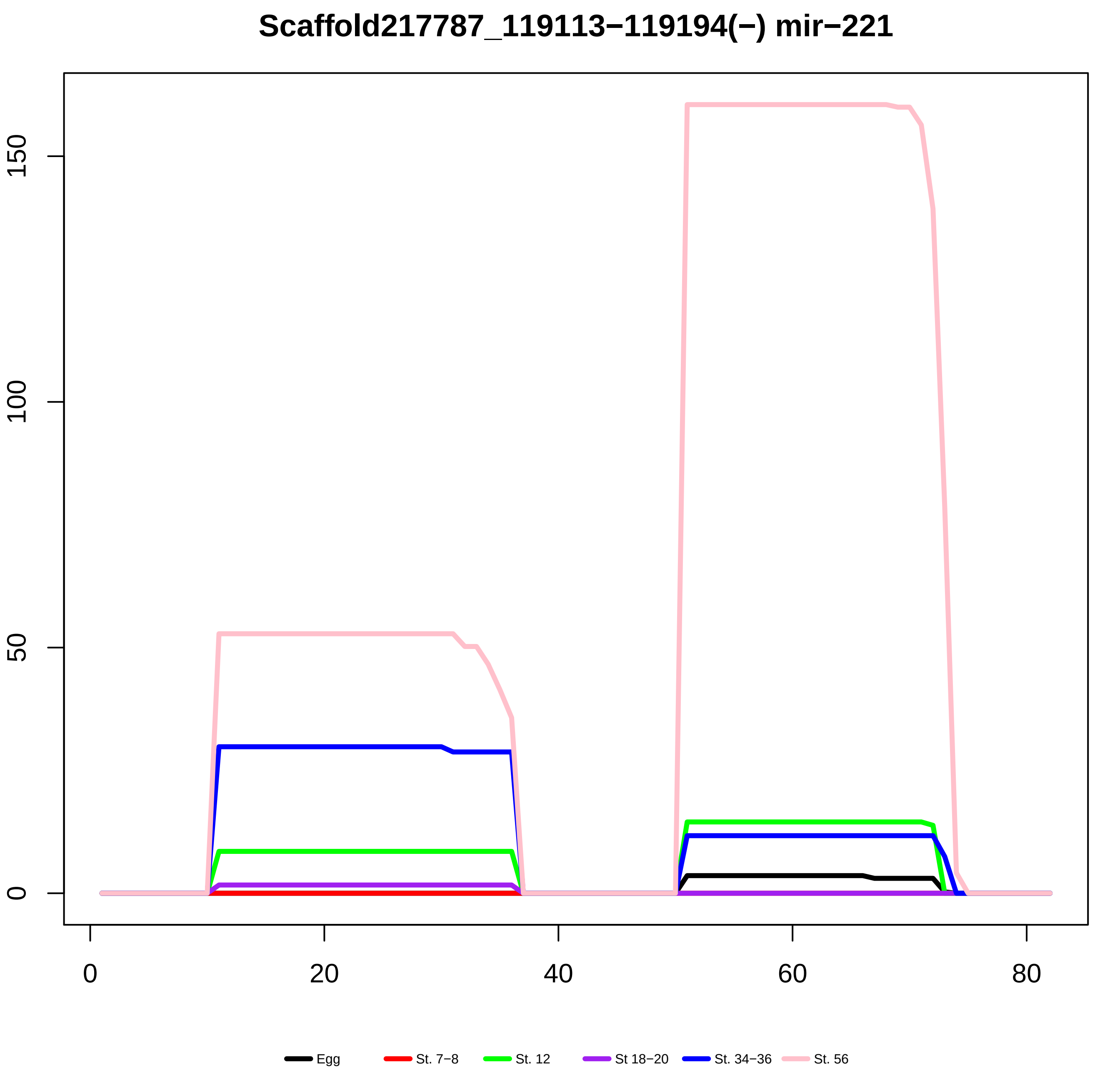

Scaffold217787_119113−119194(−) mir−221
0
50
100
150
0
20
40
60
80
Egg
St. 7−8
St. 12
St 18−20
St. 34−36
St. 56

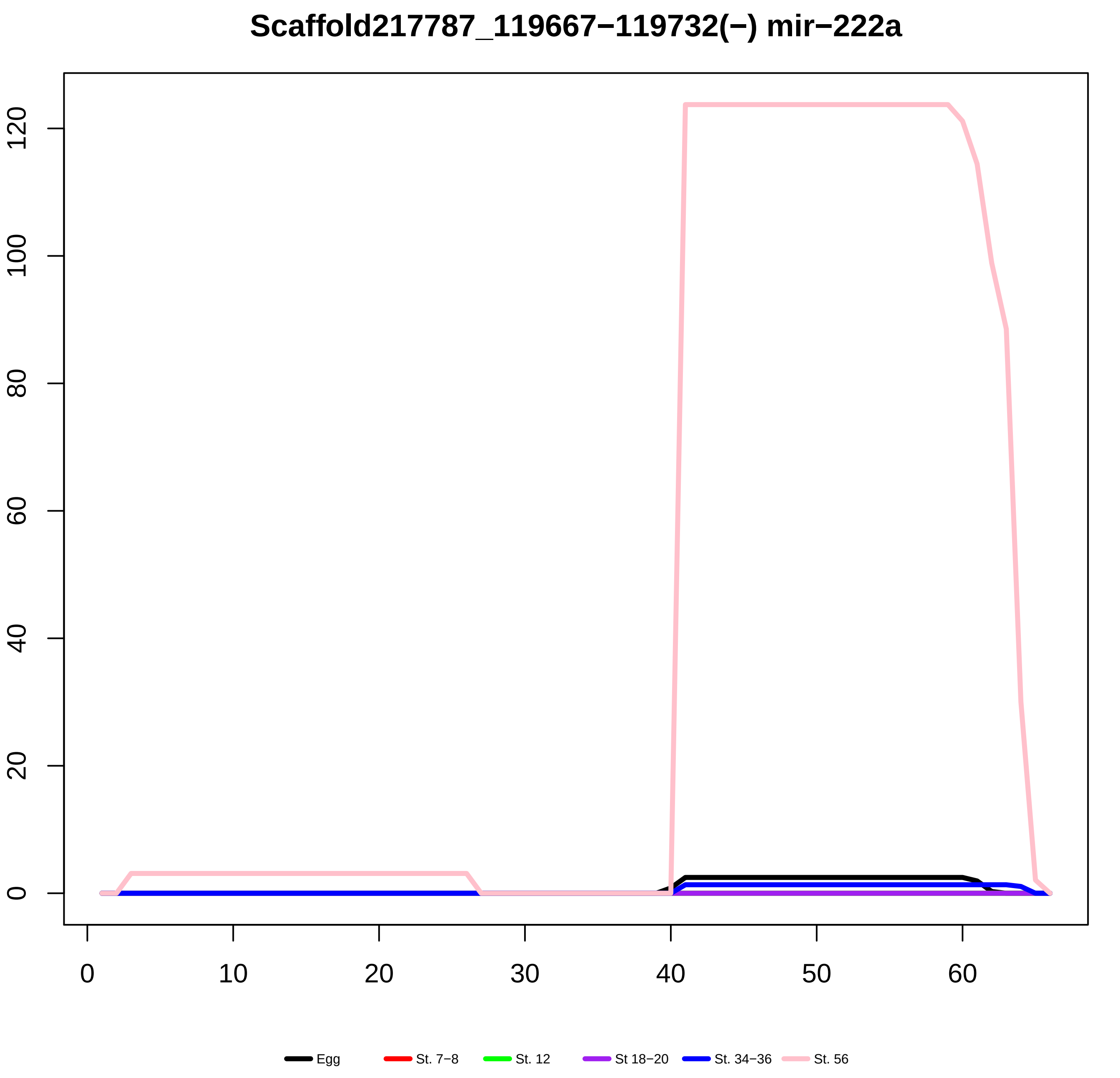

Scaffold217787_119667−119732(−) mir−222a
0
20
40
60
80
100
120
0
10
20
30
40
50
60
Egg
St. 7−8
St. 12
St 18−20
St. 34−36
St. 56

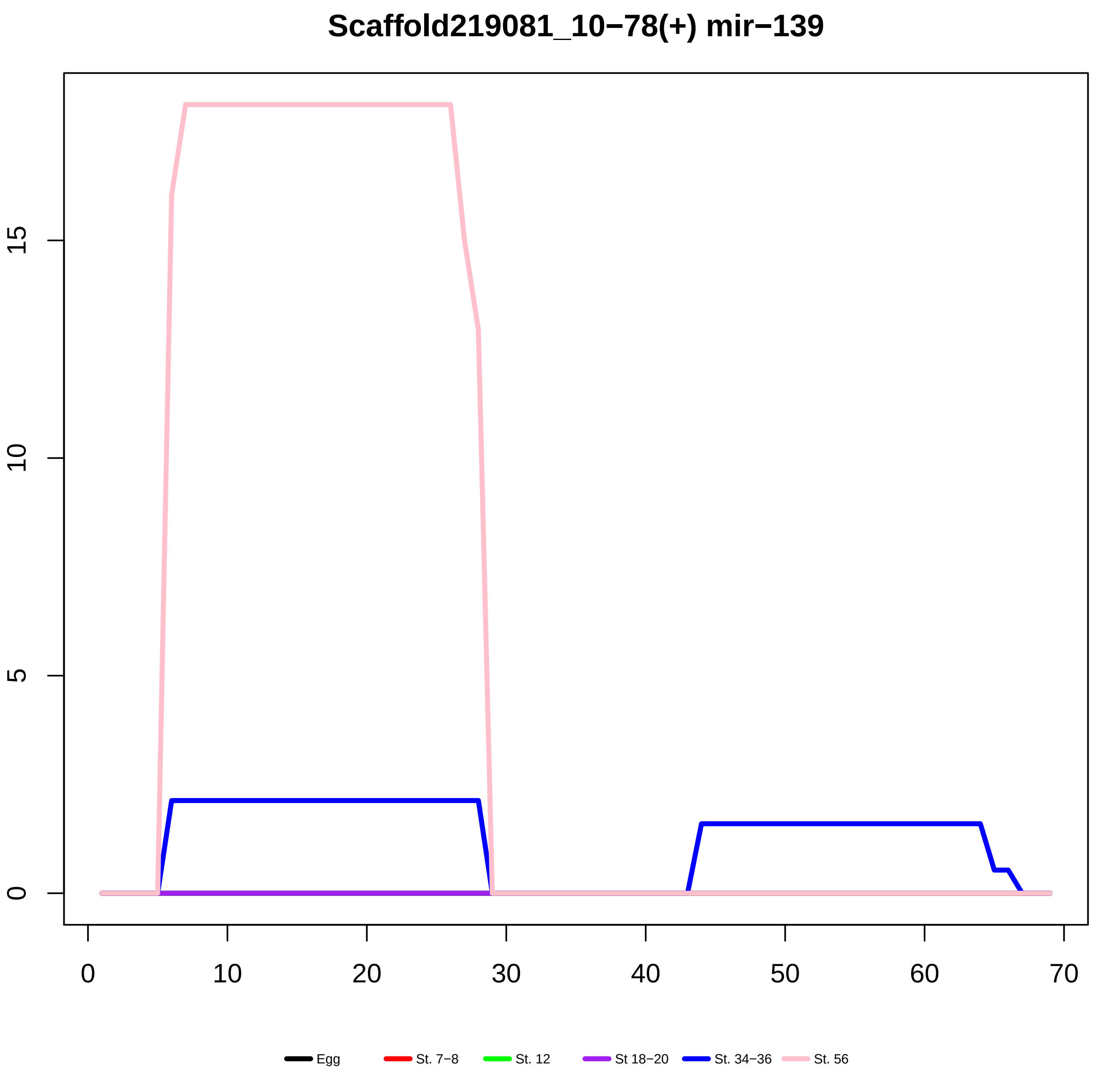

Scaffold219081_10−78(+) mir−139
0
5
10
15
0
10
20
30
40
50
60
70
Egg
St. 7−8
St. 12
St 18−20
St. 34−36
St. 56

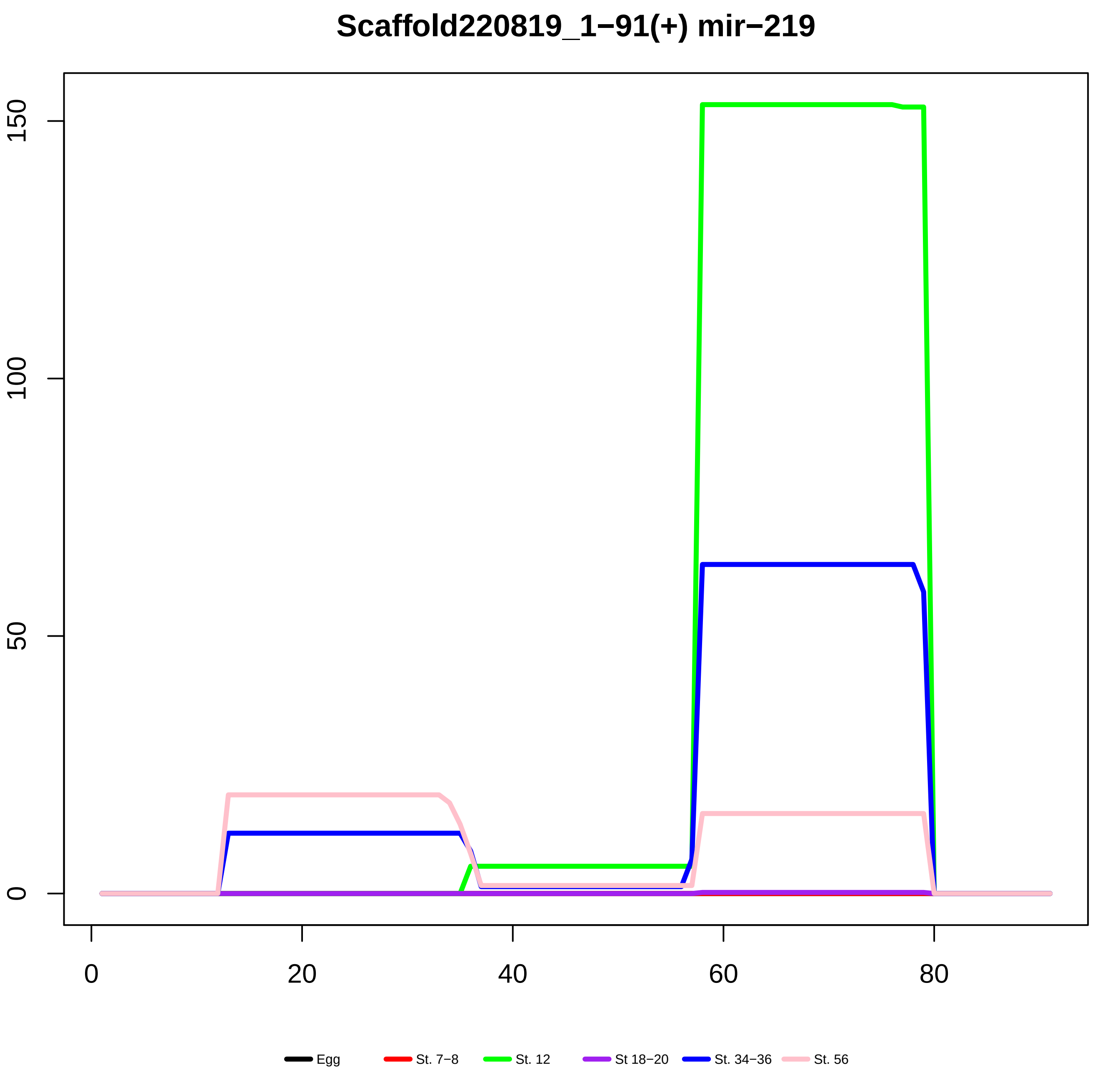

Scaffold220819_1−91(+) mir−219
0
50
100
150
0
20
40
60
80
Egg
St. 7−8
St. 12
St 18−20
St. 34−36
St. 56

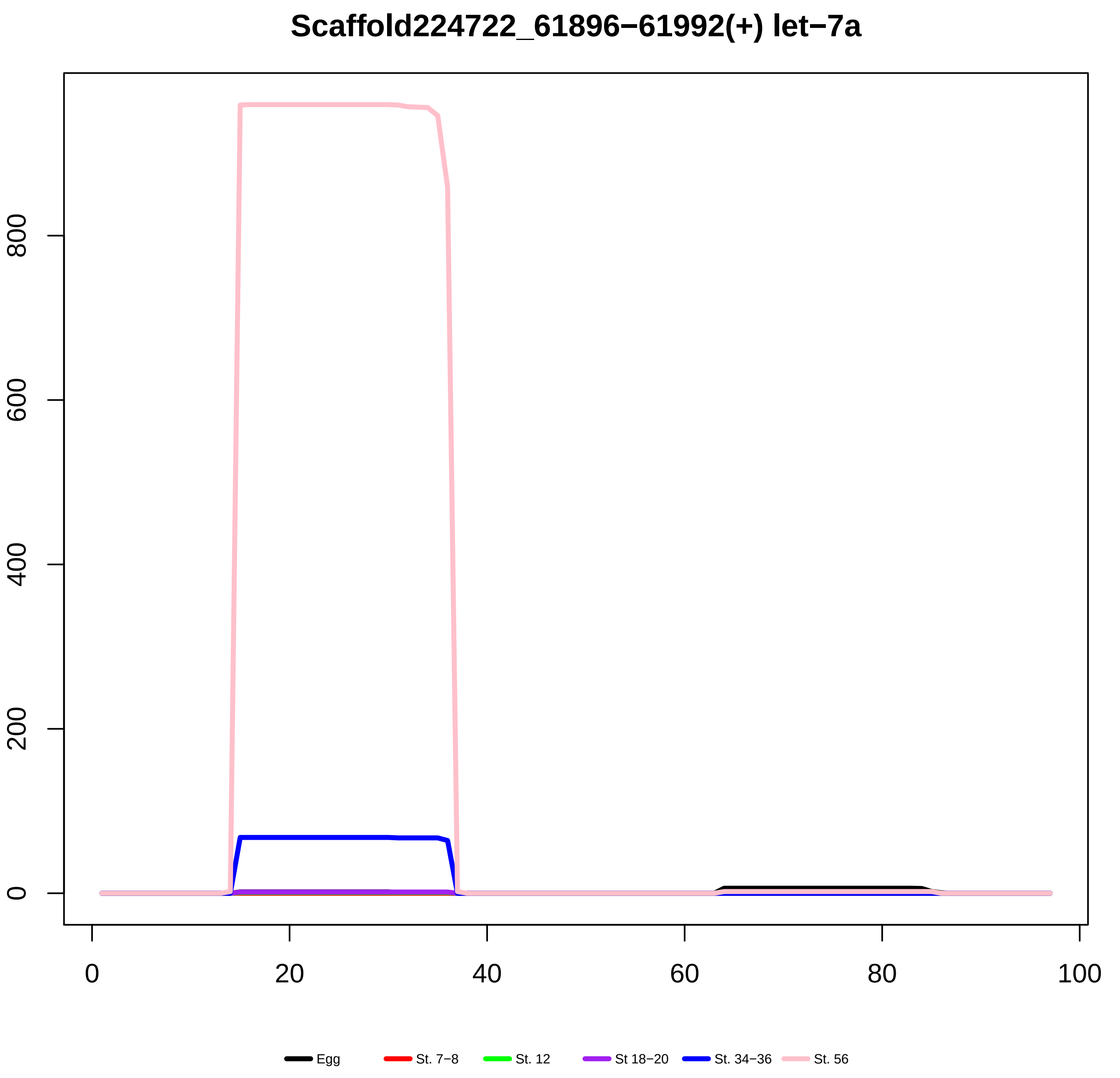

Scaffold224722_61896−61992(+) let−7a
0
200
400
600
800
0
20
40
60
80
100
Egg
St. 7−8
St. 12
St 18−20
St. 34−36
St. 56

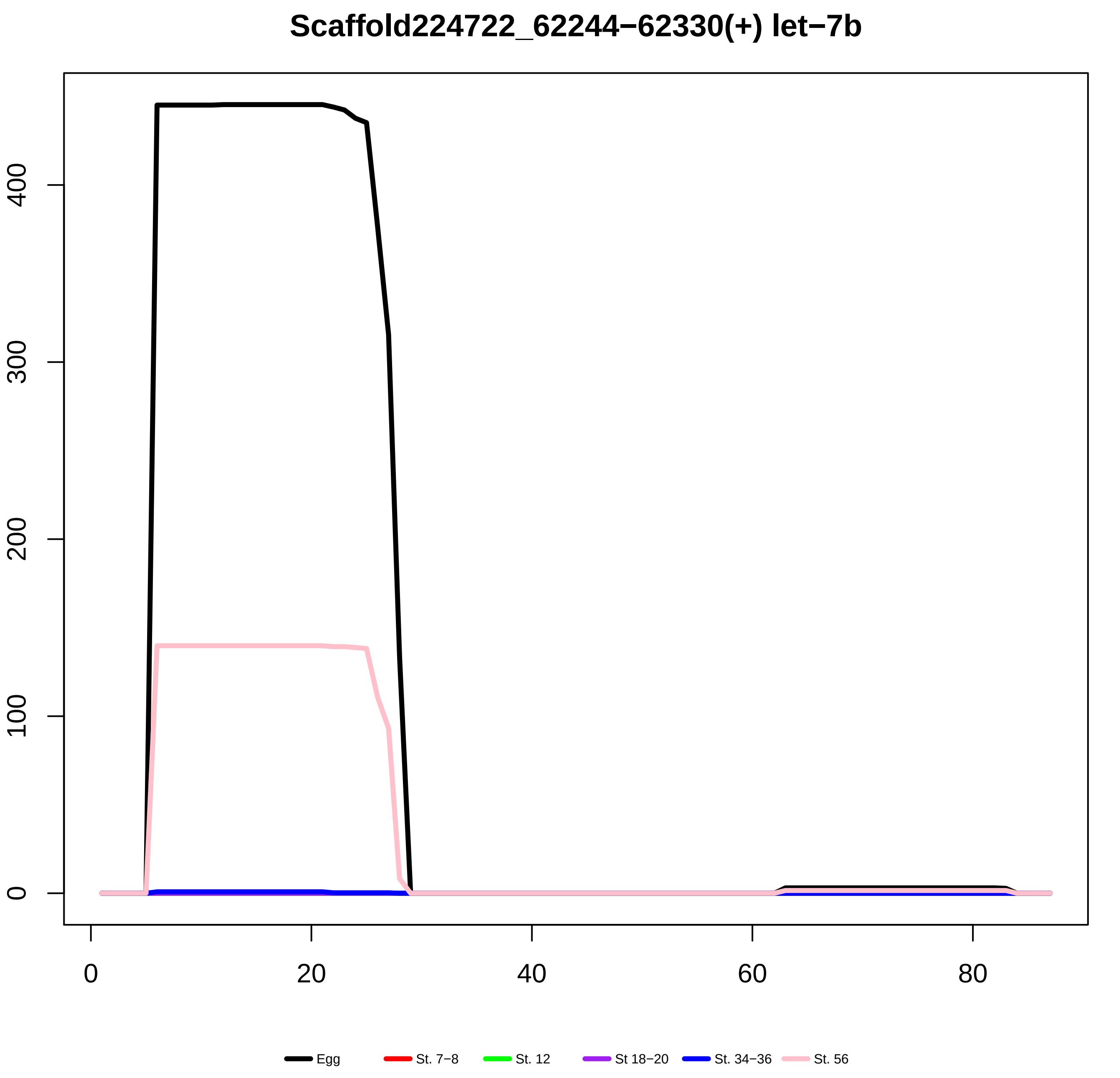
Scaffold224722_62244−62330(+) let−7b
0
100
200
300
400
0
20
40
60
80
Egg
St. 7−8
St. 12
St 18−20
St. 34−36
St. 56

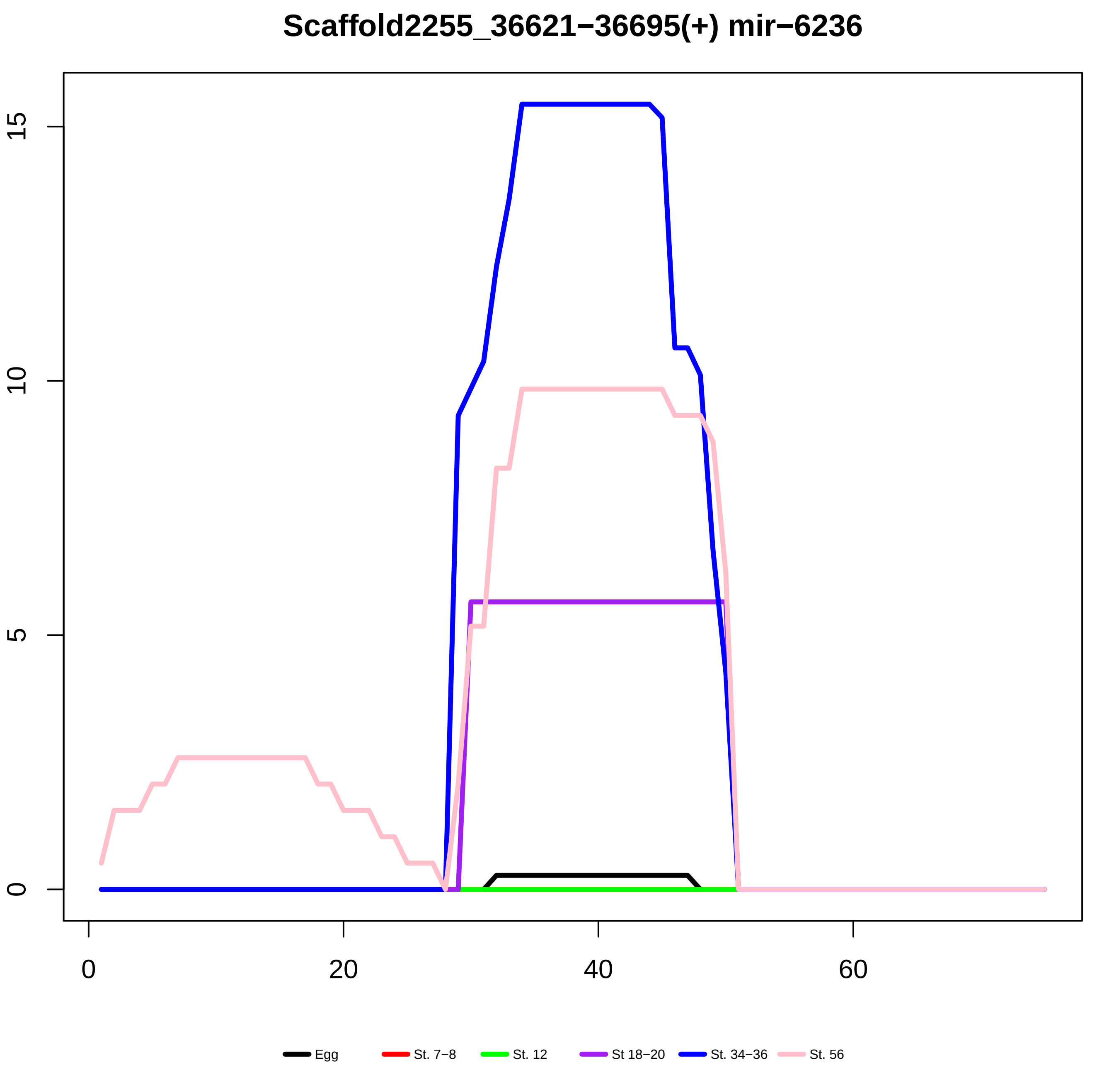
Scaffold2255_36621−36695(+) mir−6236
0
5
10
15
0
20
40
60
Egg
St. 7−8
St. 12
St 18−20
St. 34−36
St. 56

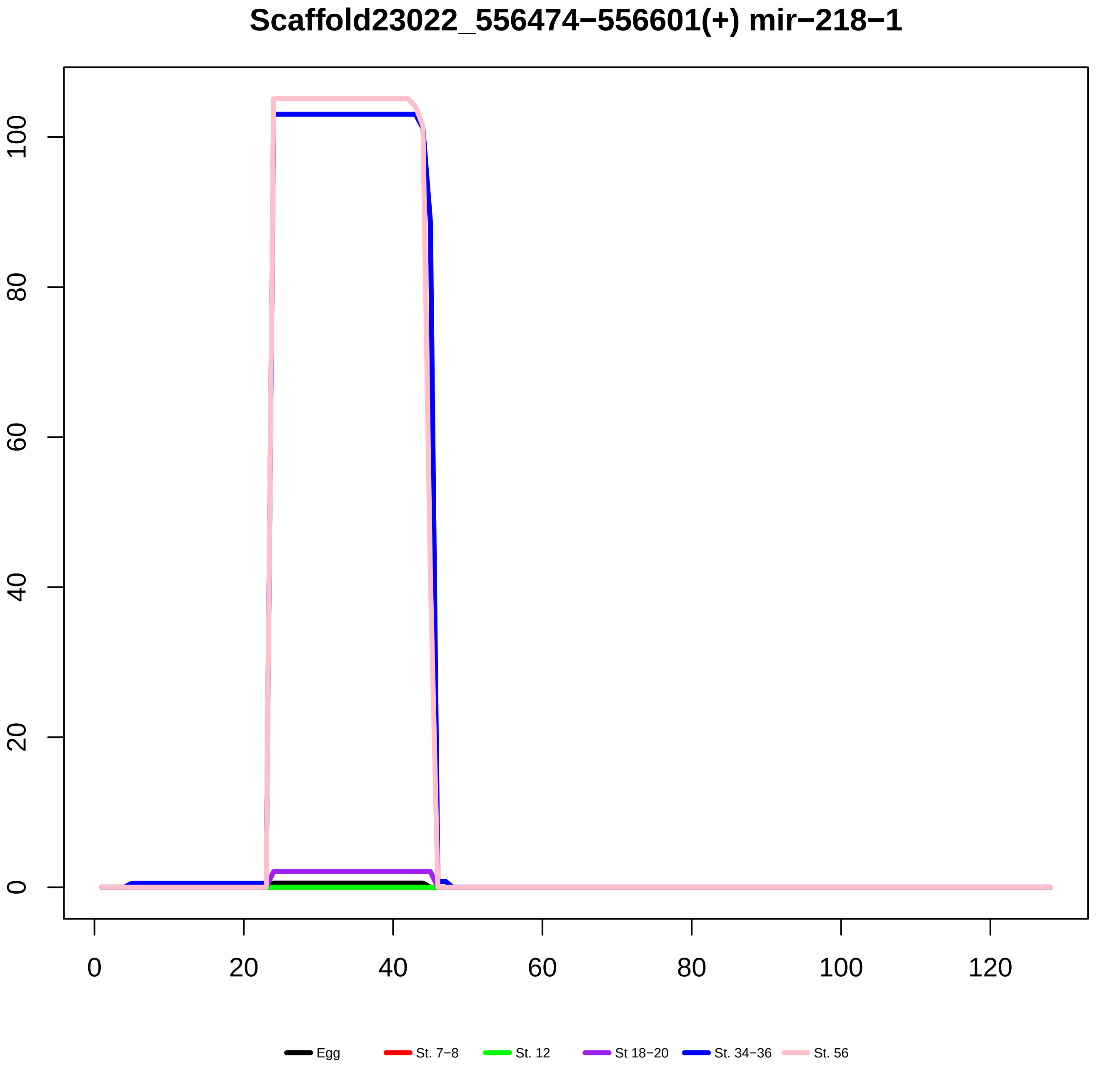

Scaffold23022_556474−556601(+) mir−218−1
0
20
40
60
80
100
0
20
40
60
80
100
120
Egg
St. 7−8
St. 12
St 18−20
St. 34−36
St. 56

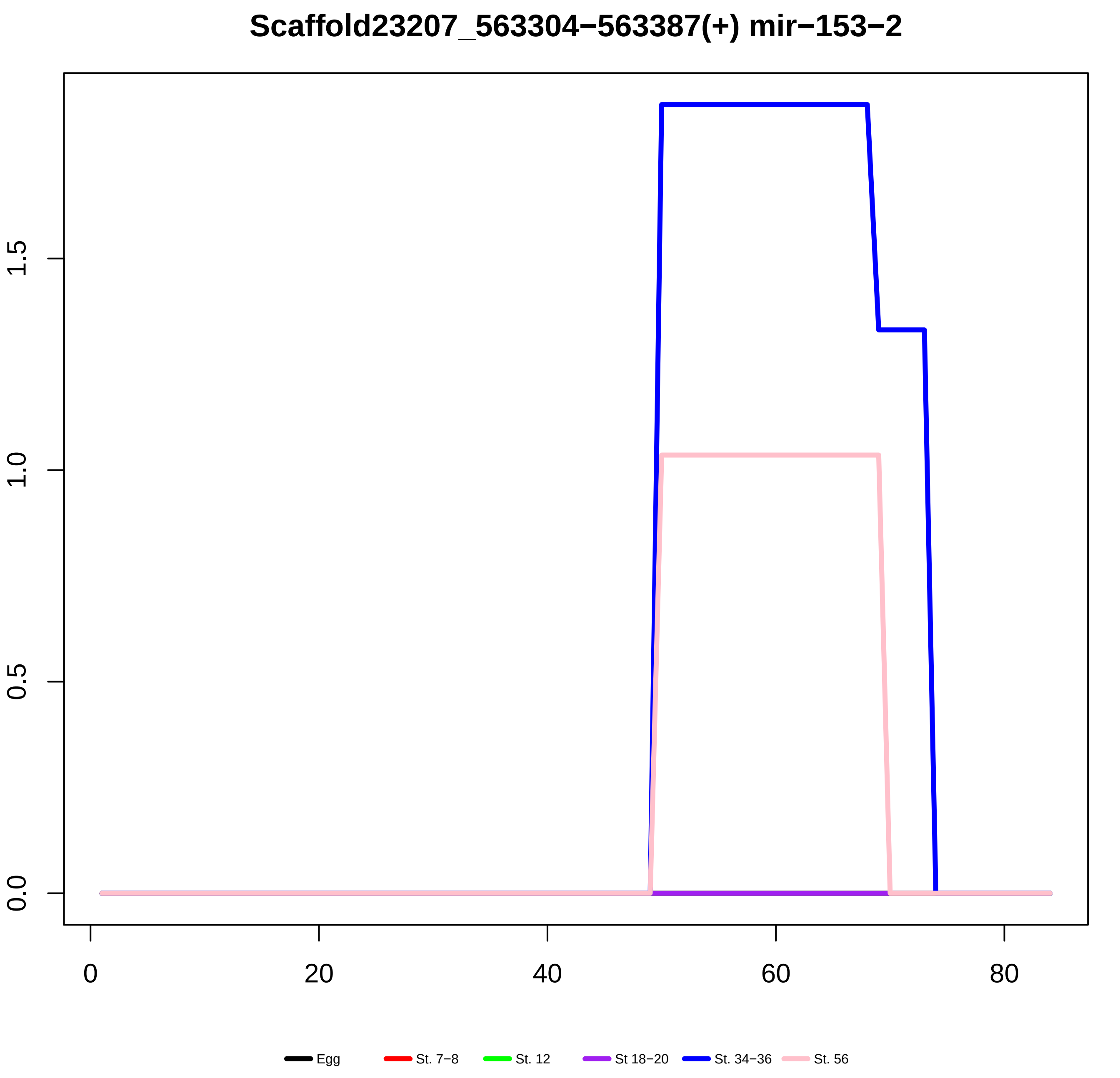

Scaffold23207_563304−563387(+) mir−153−2
0.0
0.5
1.0
1.5
0
20
40
60
80
Egg
St. 7−8
St. 12
St 18−20
St. 34−36
St. 56

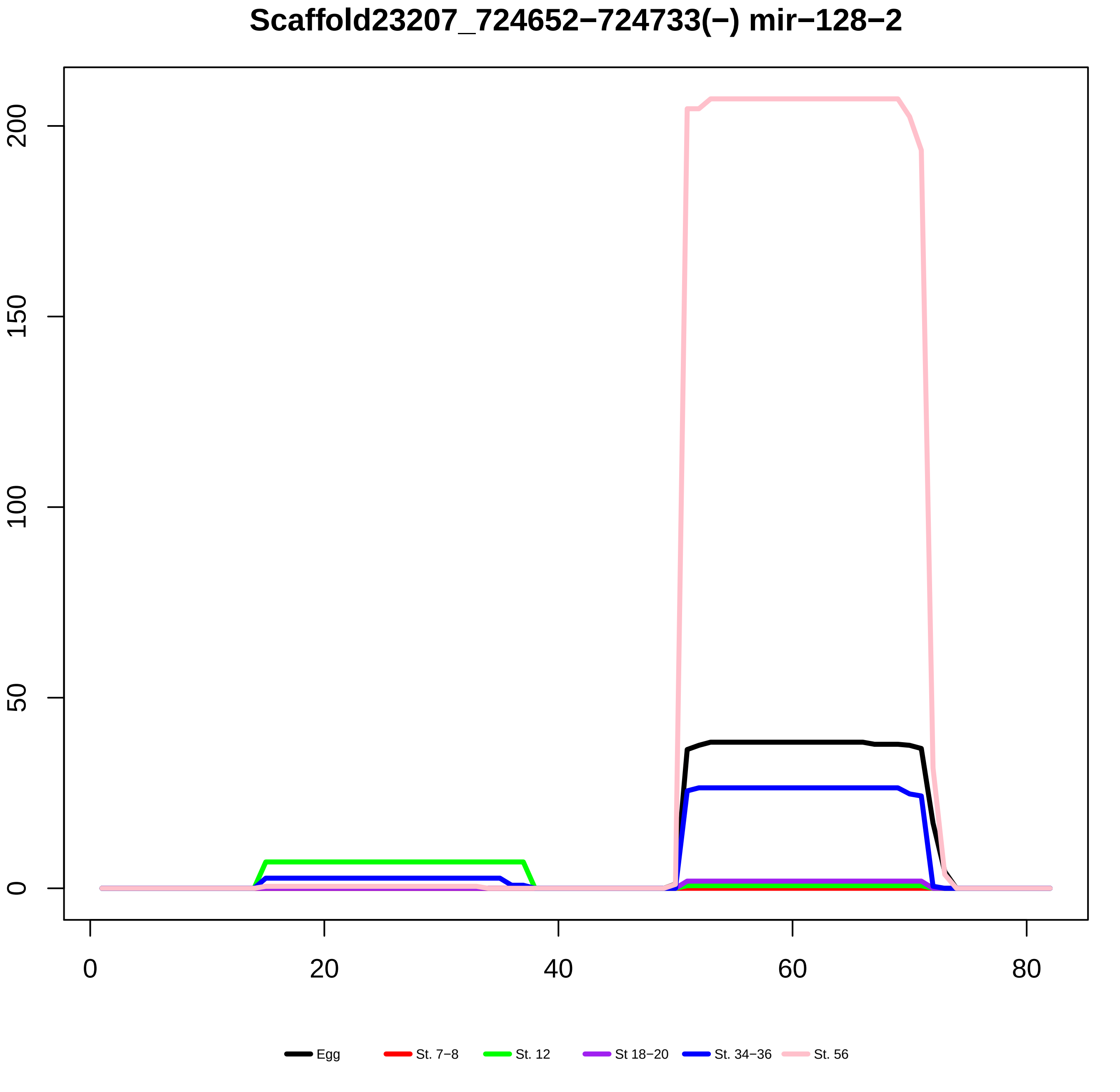
Scaffold23207_724652−724733(−) mir−128−2
0
50
100
150
200
0
20
40
60
80
Egg
St. 7−8
St. 12
St 18−20
St. 34−36
St. 56

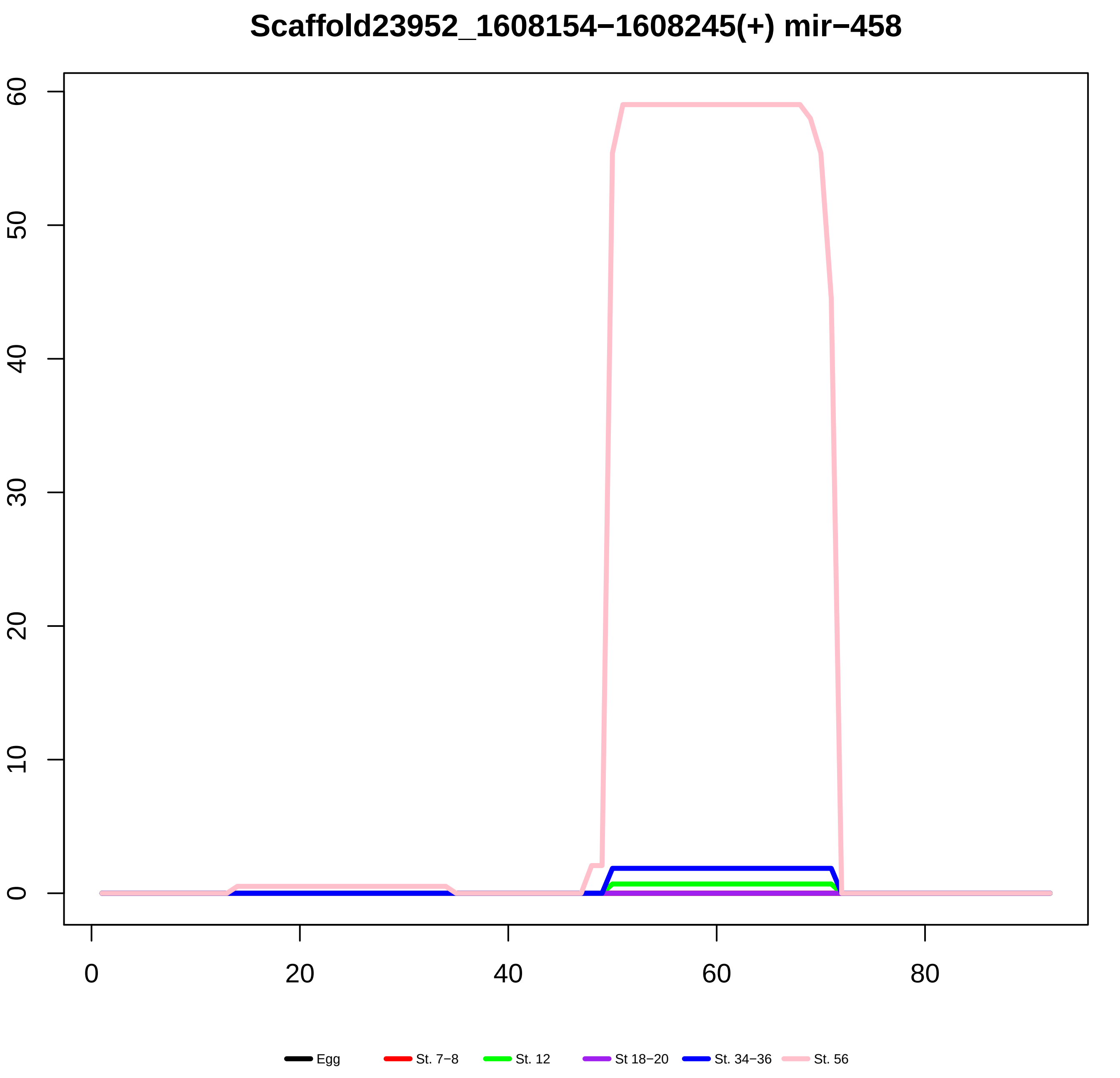

Scaffold23952_1608154−1608245(+) mir−458
60
50
40
30
20
10
0
0
20
40
60
80
Egg
St. 7−8
St. 12
St 18−20
St. 34−36
St. 56

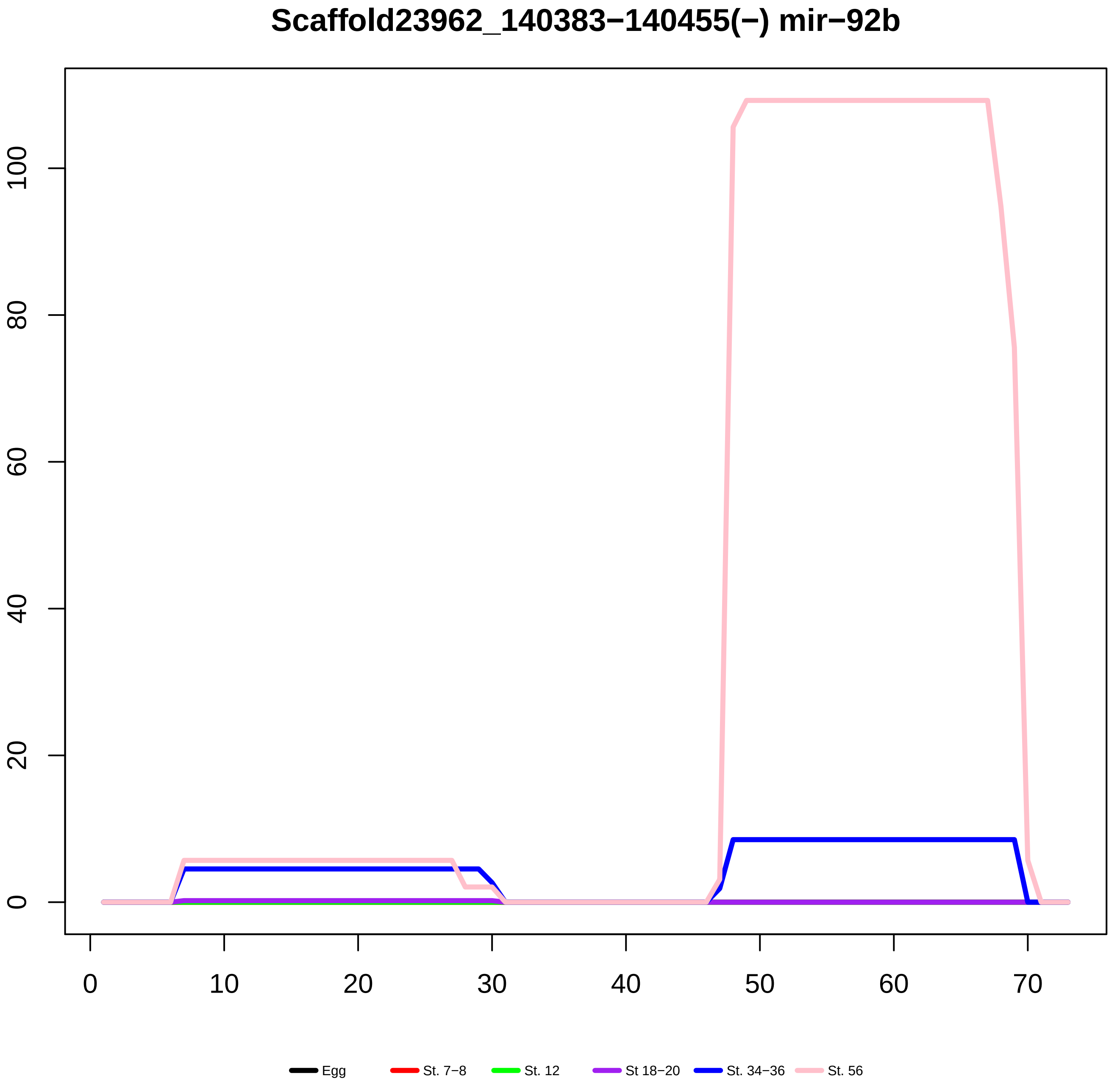

Scaffold23962_140383−140455(−) mir−92b
0
20
40
60
80
100
0
10
20
30
40
50
60
70
Egg
St. 7−8
St. 12
St 18−20
St. 34−36
St. 56

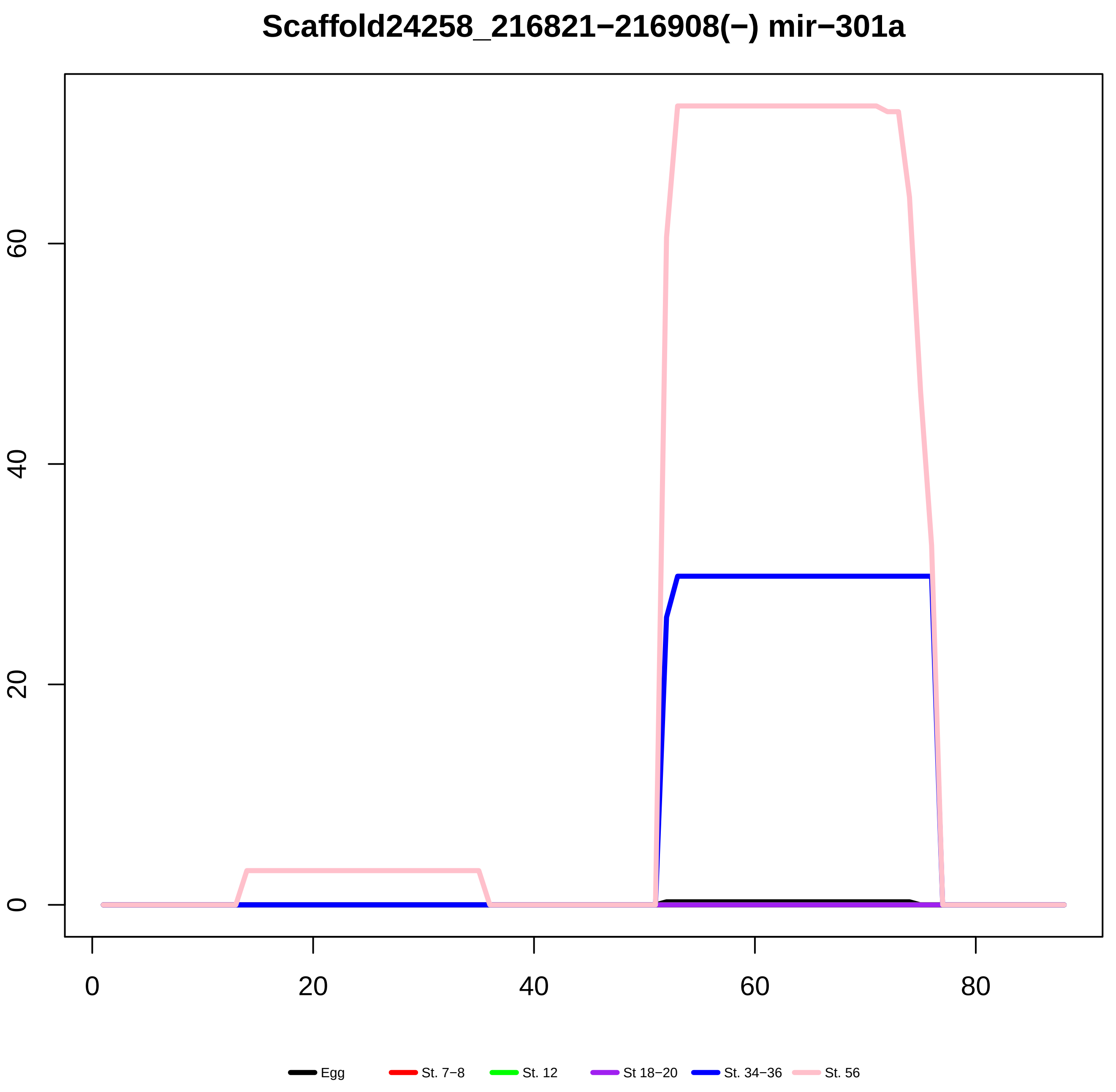

Scaffold24258_216821−216908(−) mir−301a
0
20
40
60
0
20
40
60
80
Egg
St. 7−8
St. 12
St 18−20
St. 34−36
St. 56

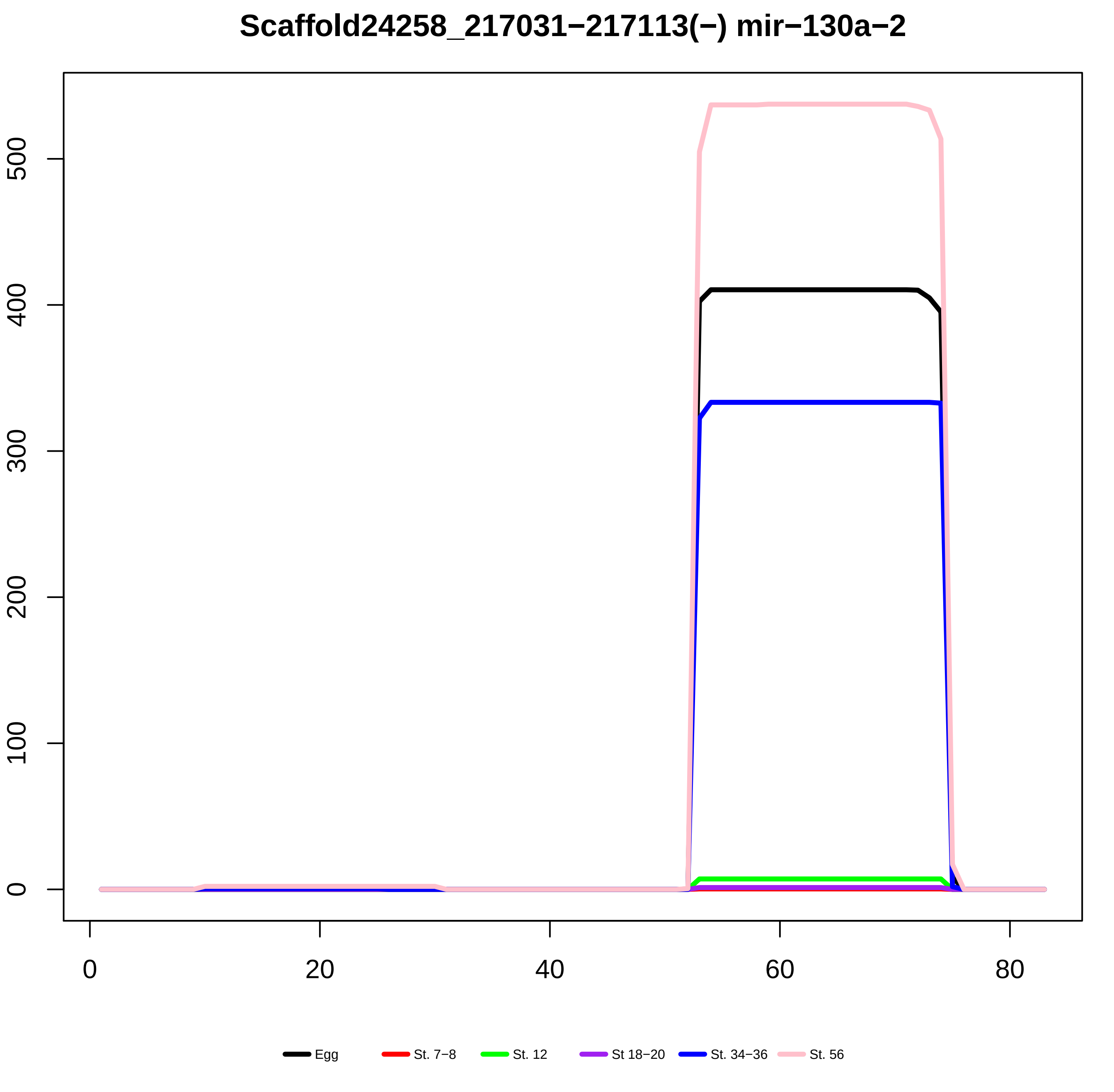
Scaffold24258_217031−217113(−) mir−130a−2
0
100
200
300
400
500
0
20
40
60
80
Egg
St. 7−8
St. 12
St 18−20
St. 34−36
St. 56

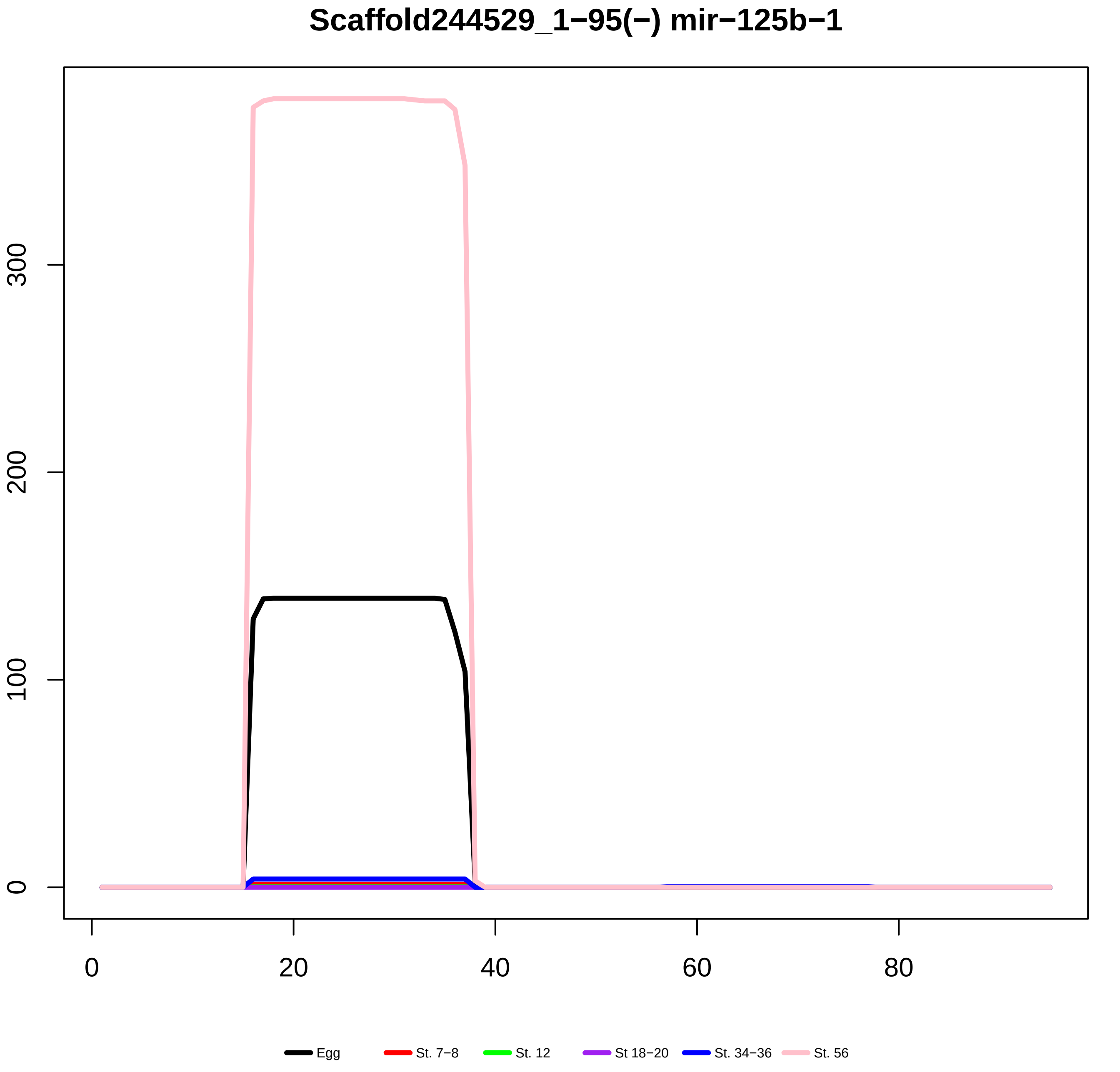
Scaffold244529_1−95(−) mir−125b−1
0
100
200
300
0
20
40
60
80
Egg
St. 7−8
St. 12
St 18−20
St. 34−36
St. 56

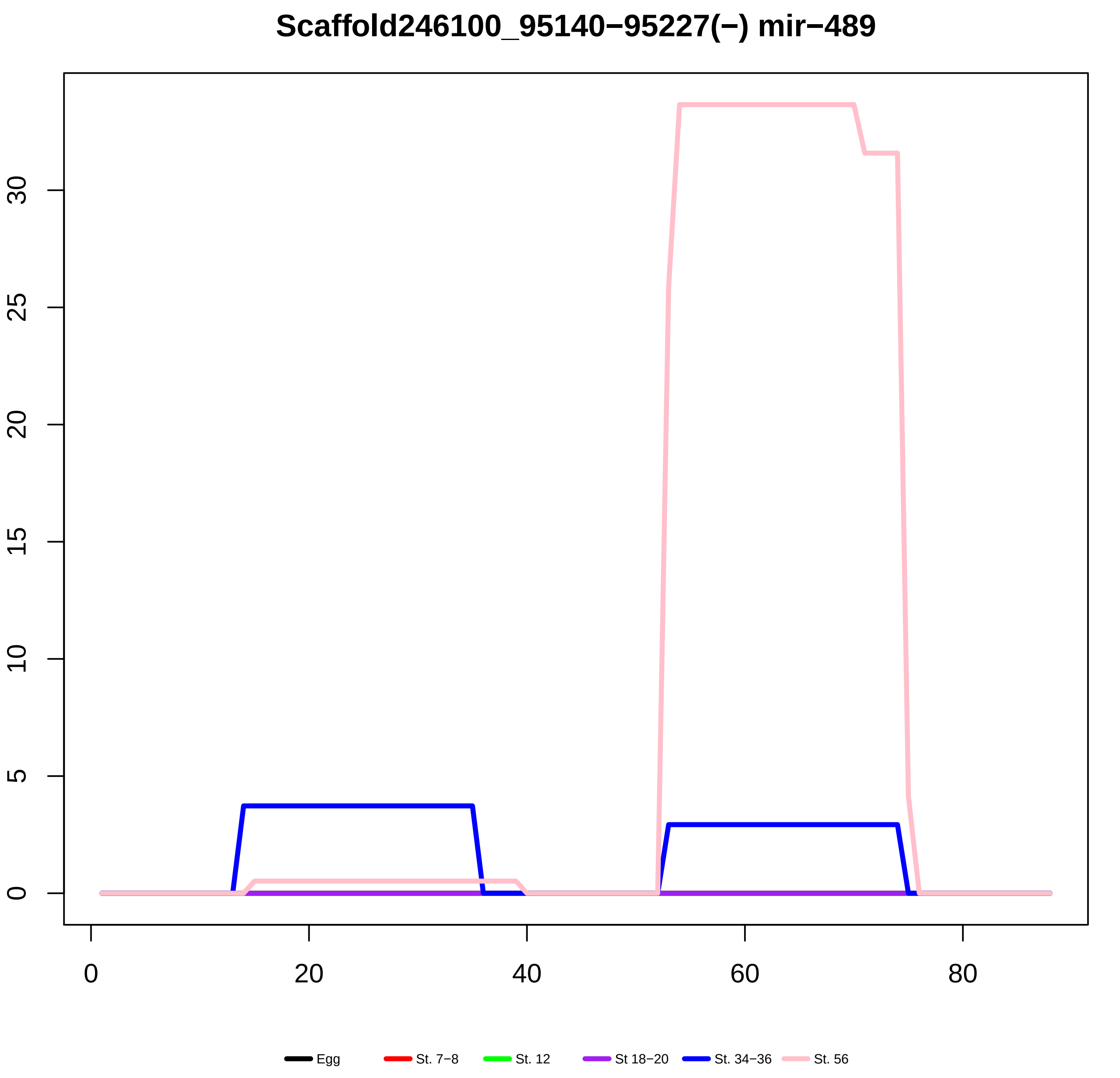

Scaffold246100_95140−95227(−) mir−489
0
5
10
15
20
25
30
0
20
40
60
80
Egg
St. 7−8
St. 12
St 18−20
St. 34−36
St. 56

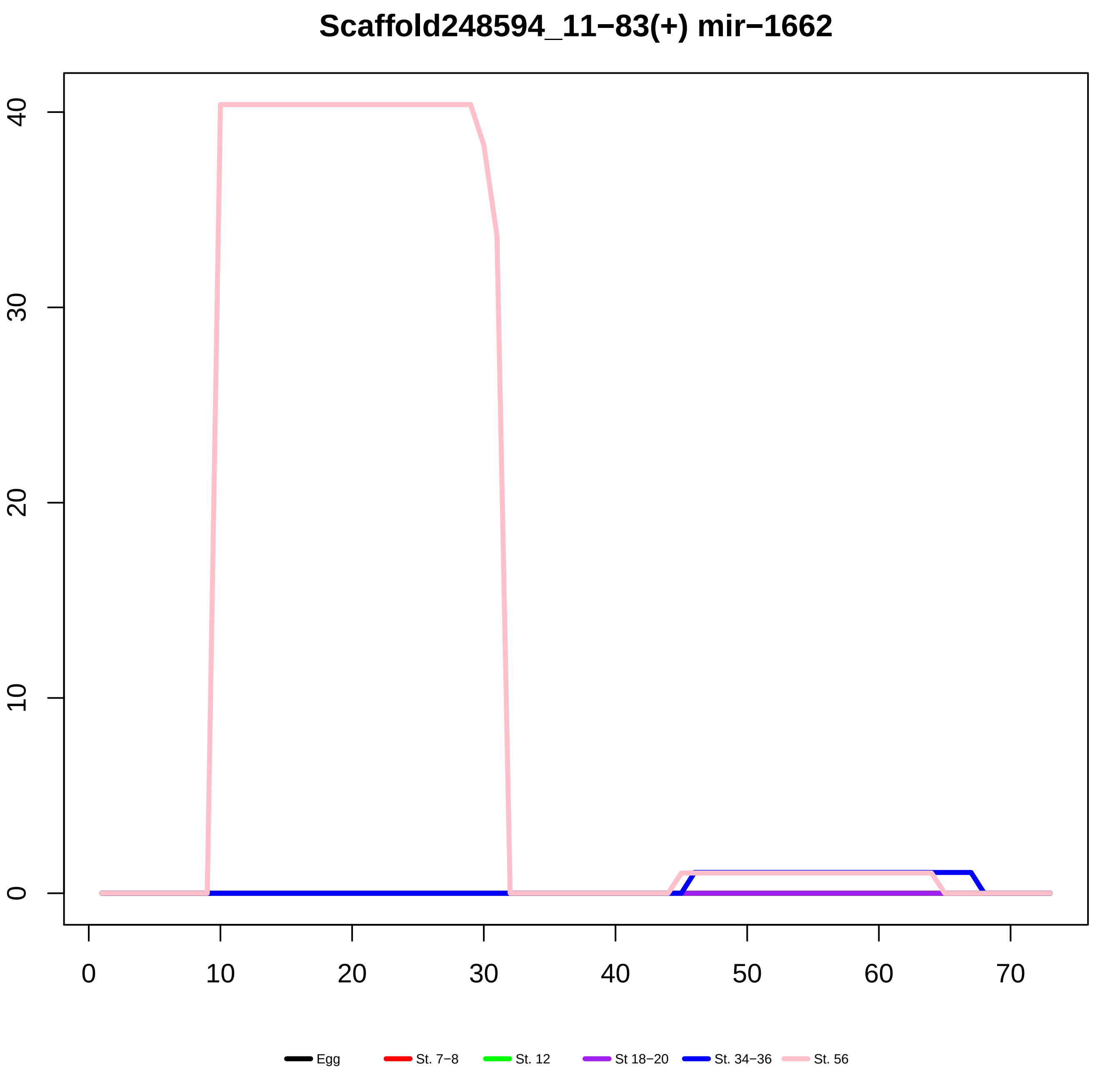

Scaffold248594_11−83(+) mir−1662
0
10
20
30
40
0
10
20
30
40
50
60
70
Egg
St. 7−8
St. 12
St 18−20
St. 34−36
St. 56

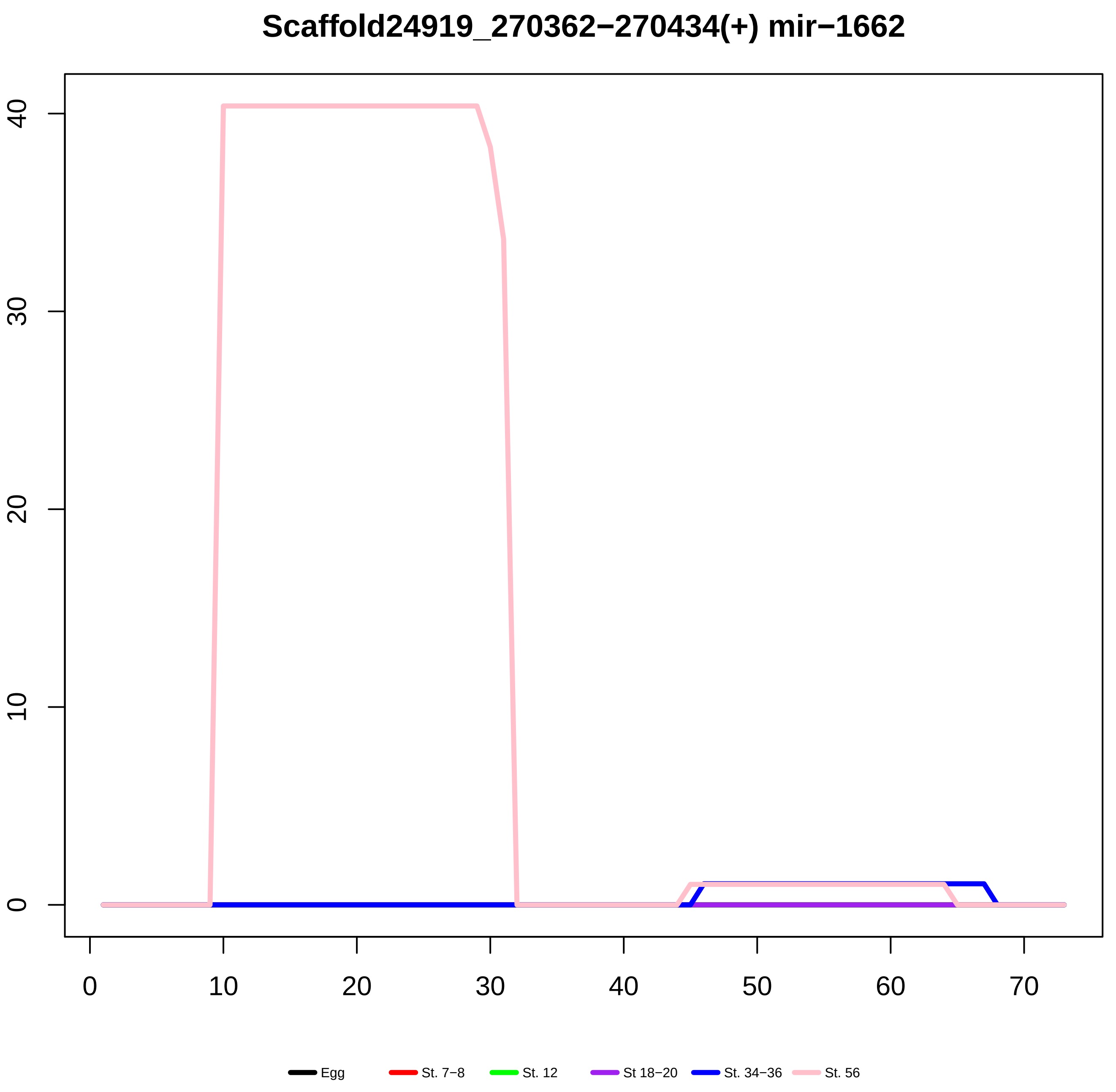

Scaffold24919_270362−270434(+) mir−1662
0
10
20
30
40
0
10
20
30
40
50
60
70
Egg
St. 7−8
St. 12
St 18−20
St. 34−36
St. 56

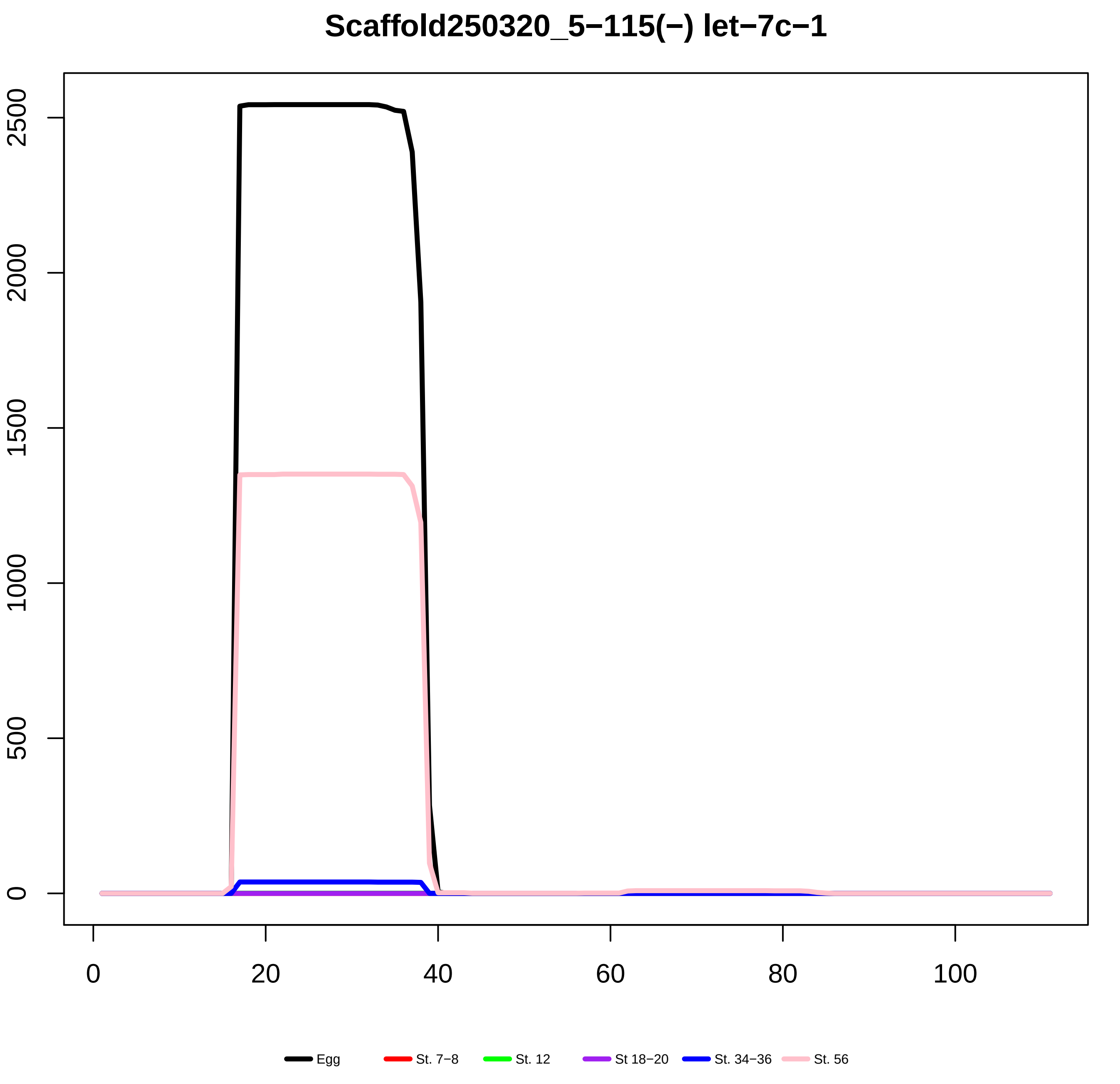
Scaffold250320_5−115(−) let−7c−1
0
500
1000
1500
2000
2500
0
20
40
60
80
100
Egg
St. 7−8
St. 12
St 18−20
St. 34−36
St. 56

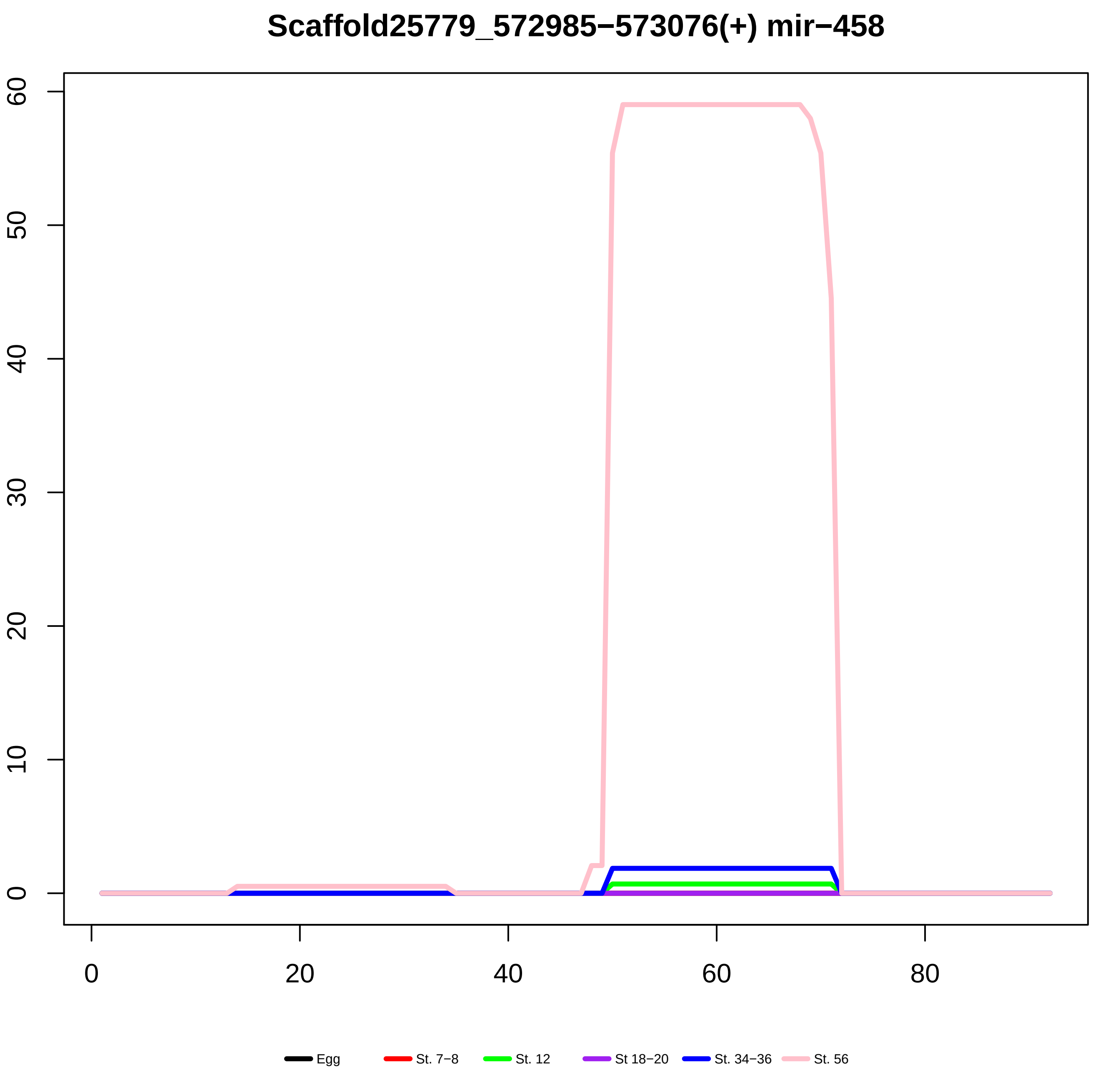

Scaffold25779_572985−573076(+) mir−458
0
10
20
30
40
50
60
0
20
40
60
80
Egg
St. 7−8
St. 12
St 18−20
St. 34−36
St. 56

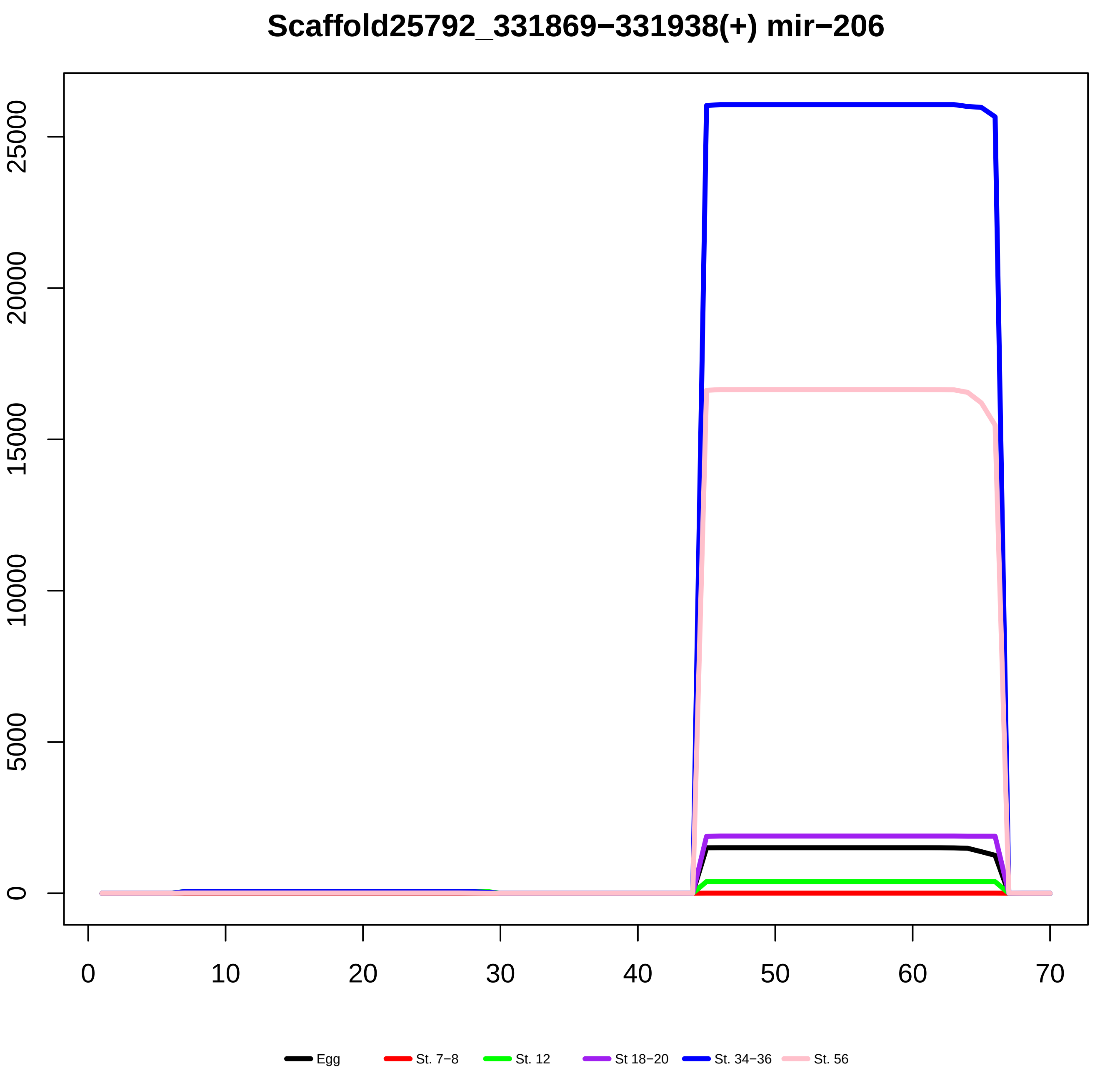
Scaffold25792_331869−331938(+) mir−206
0
5000
10000
15000
20000
25000
0
10
20
30
40
50
60
70
Egg
St. 7−8
St. 12
St 18−20
St. 34−36
St. 56

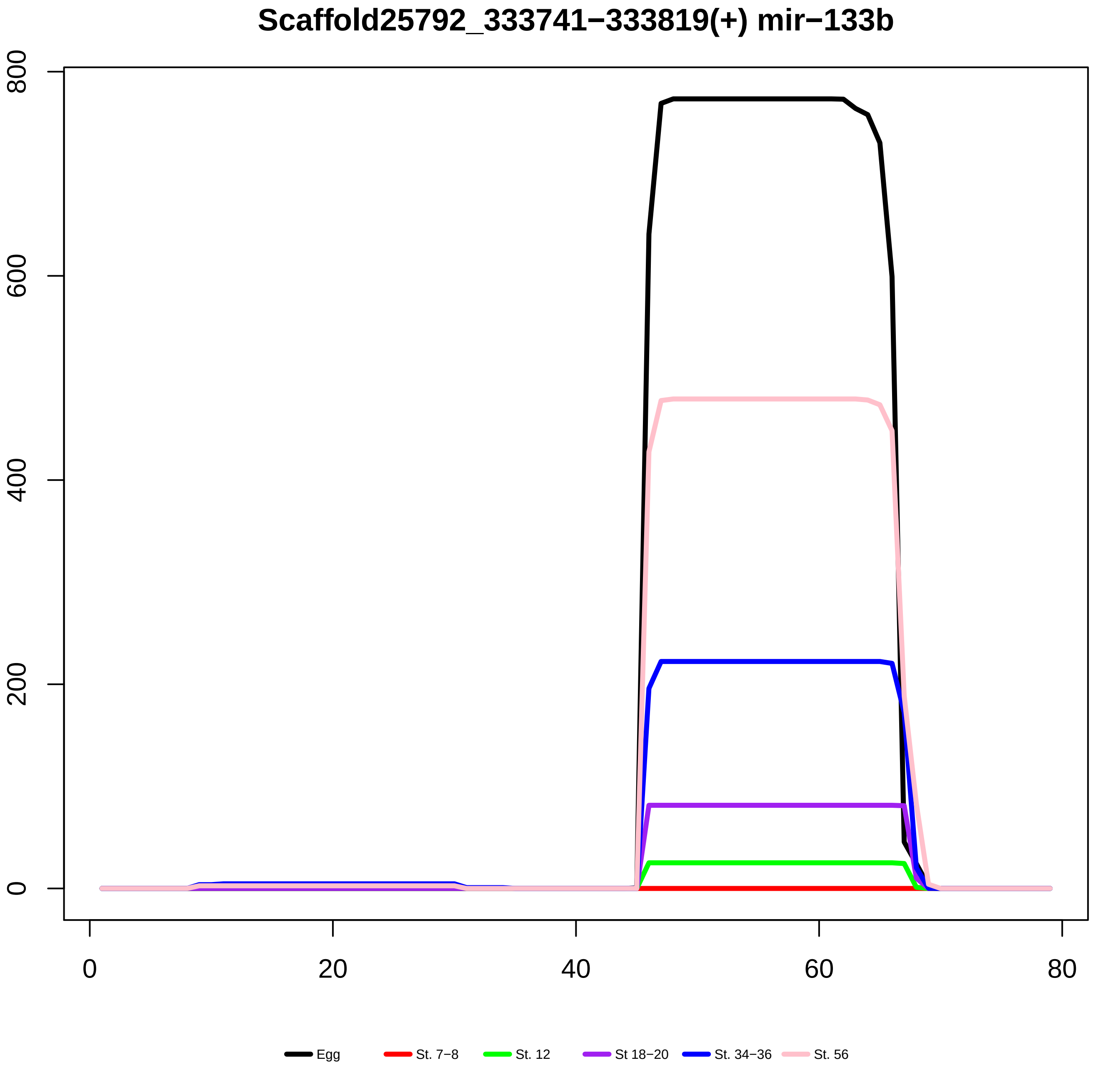

Scaffold25792_333741−333819(+) mir−133b
0
200
400
600
800
0
20
40
60
80
Egg
St. 7−8
St. 12
St 18−20
St. 34−36
St. 56

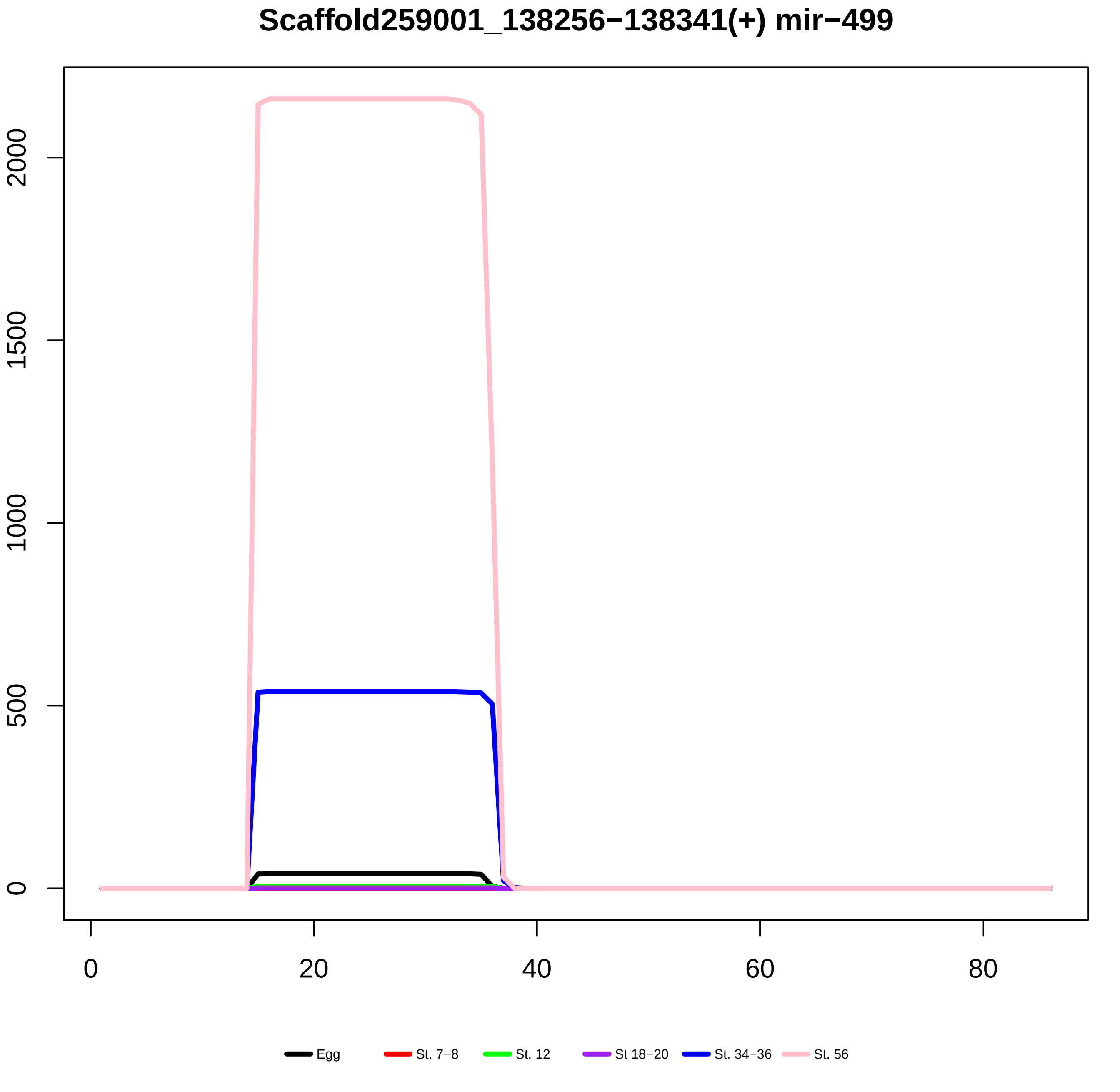

Scaffold259001_138256−138341(+) mir−499
0
500
1000
1500
2000
0
20
40
60
80
Egg
St. 7−8
St. 12
St 18−20
St. 34−36
St. 56

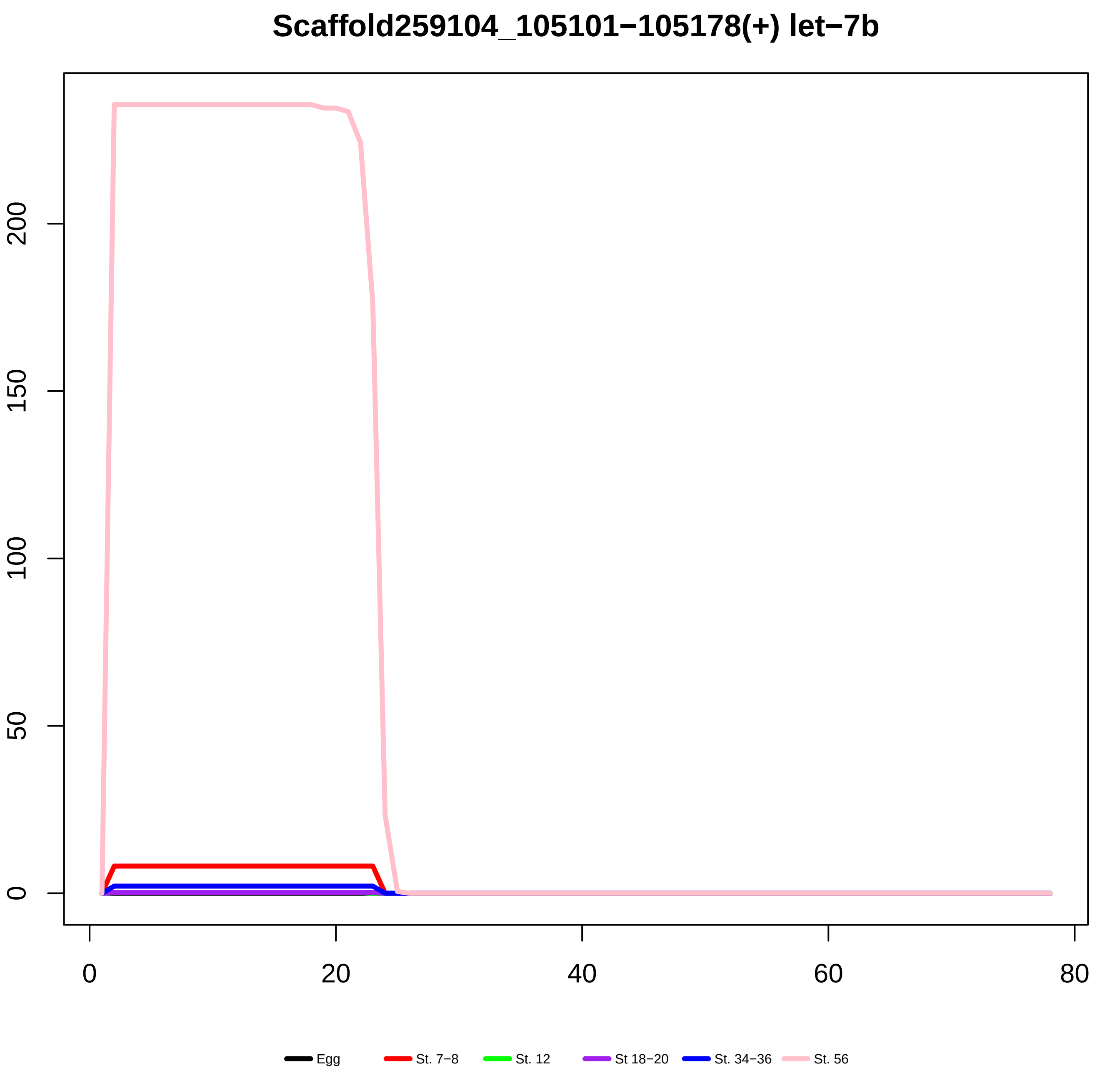

Scaffold259104_105101−105178(+) let−7b
0
50
100
150
200
0
20
40
60
80
Egg
St. 7−8
St. 12
St 18−20
St. 34−36
St. 56

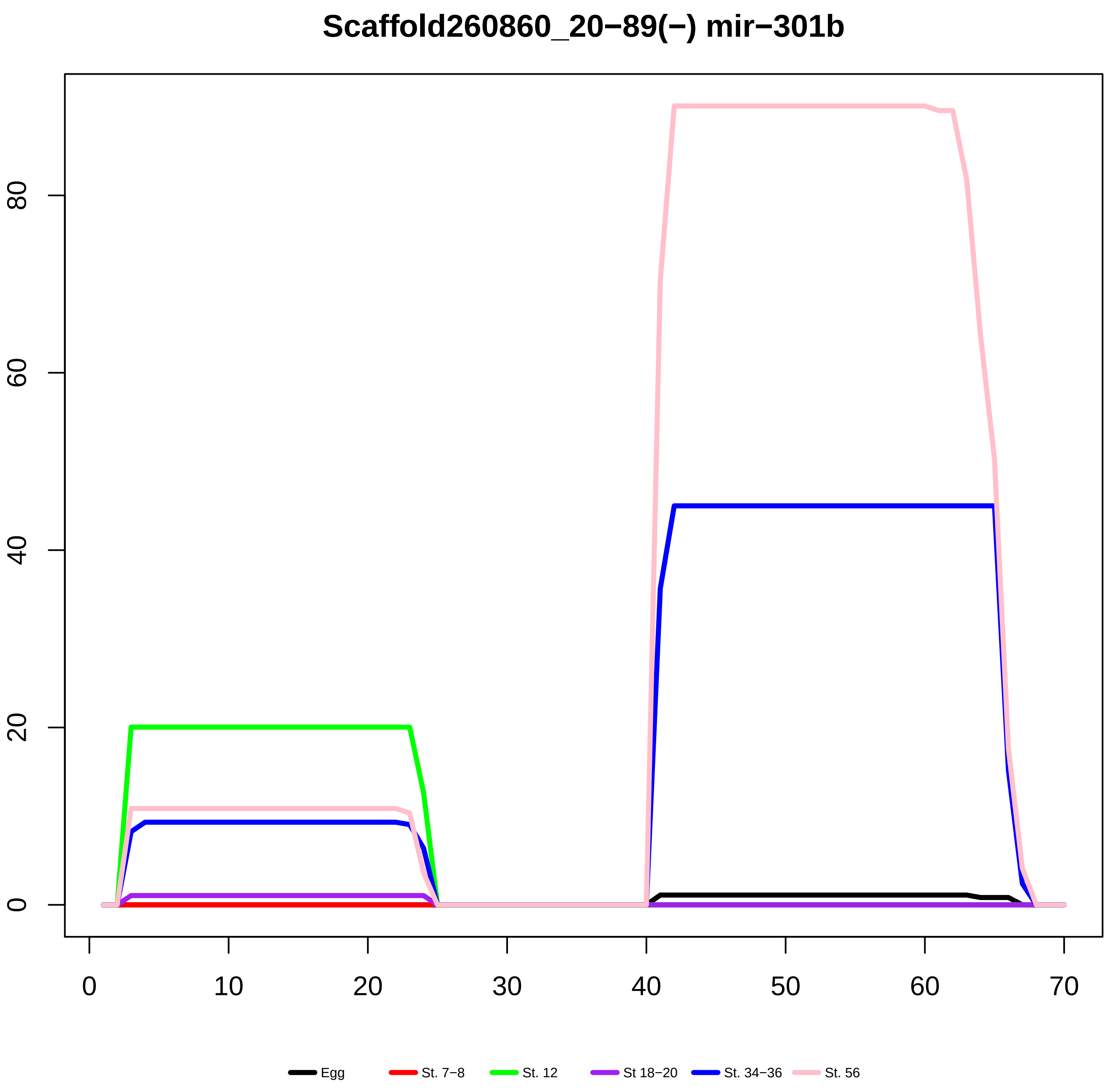

Scaffold260860_20−89(−) mir−301b
0
20
40
60
80
0
10
20
30
40
50
60
70
Egg
St. 7−8
St. 12
St 18−20
St. 34−36
St. 56

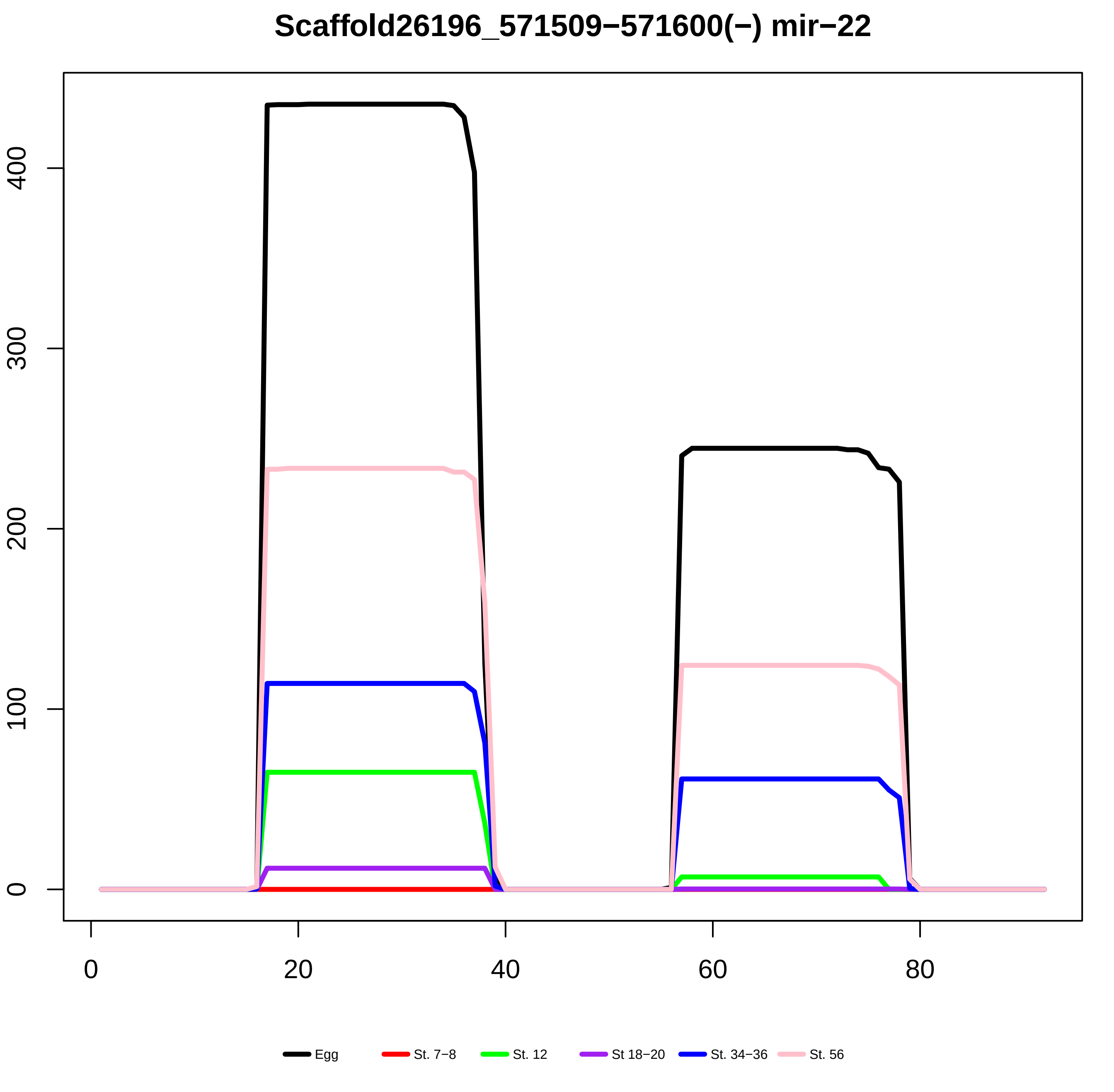

Scaffold26196_571509−571600(−) mir−22
0
100
200
300
400
0
20
40
60
80
Egg
St. 7−8
St. 12
St 18−20
St. 34−36
St. 56

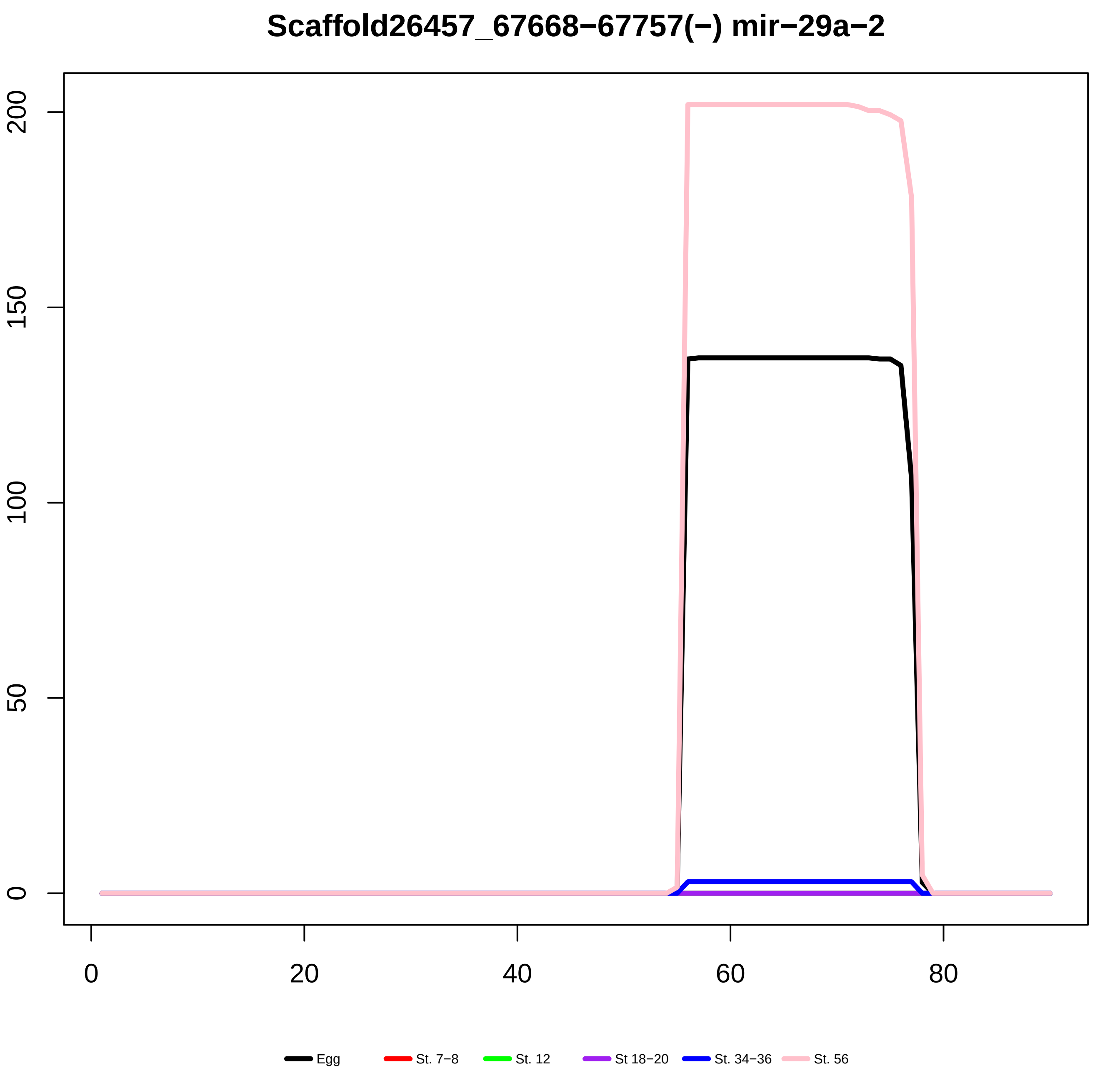

Scaffold26457_67668−67757(−) mir−29a−2
0
50
100
150
200
0
20
40
60
80
Egg
St. 7−8
St. 12
St 18−20
St. 34−36
St. 56

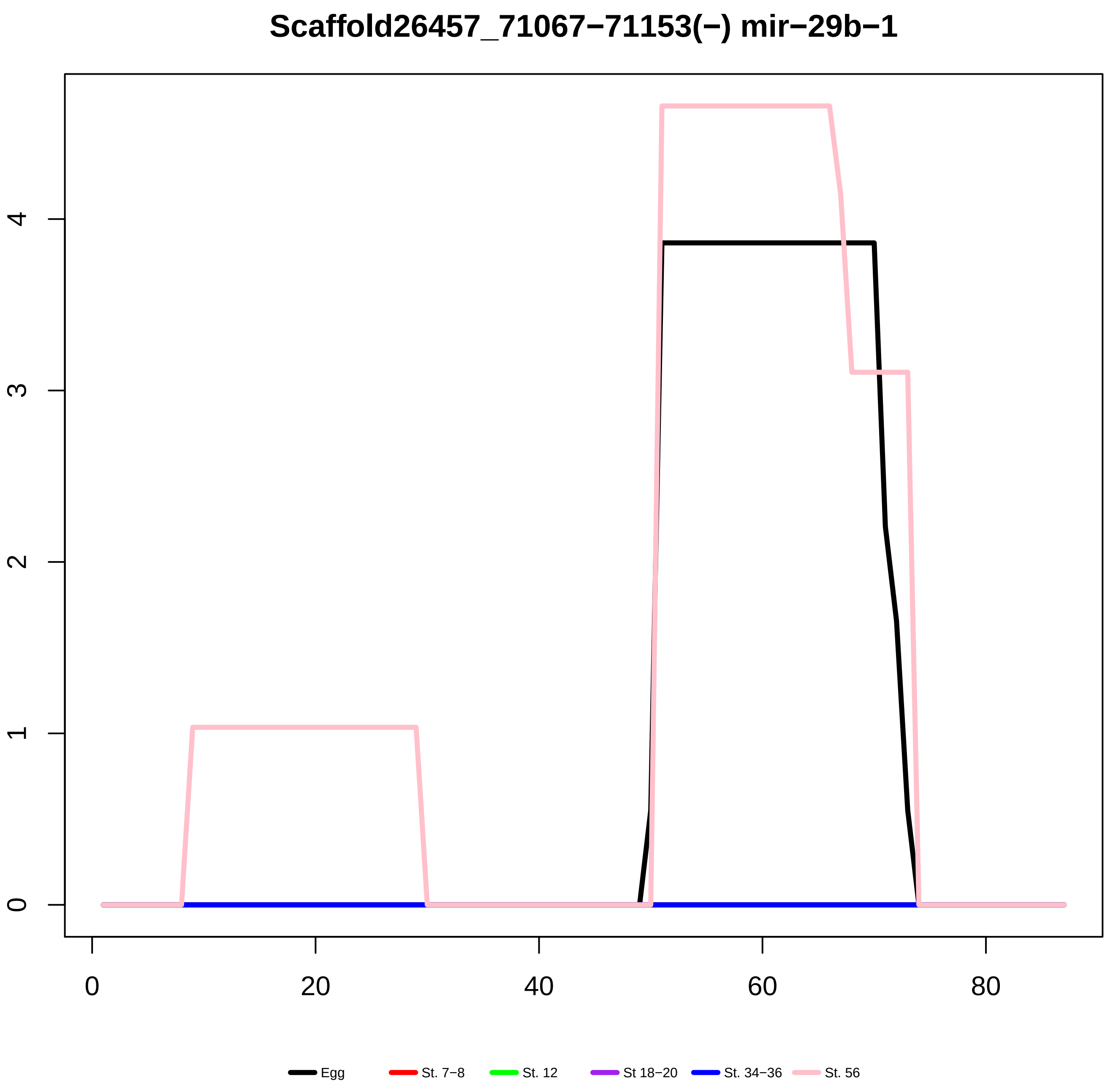
Scaffold26457_71067−71153(−) mir−29b−1
0
1
2
3
4
0
20
40
60
80
Egg
St. 7−8
St. 12
St 18−20
St. 34−36
St. 56

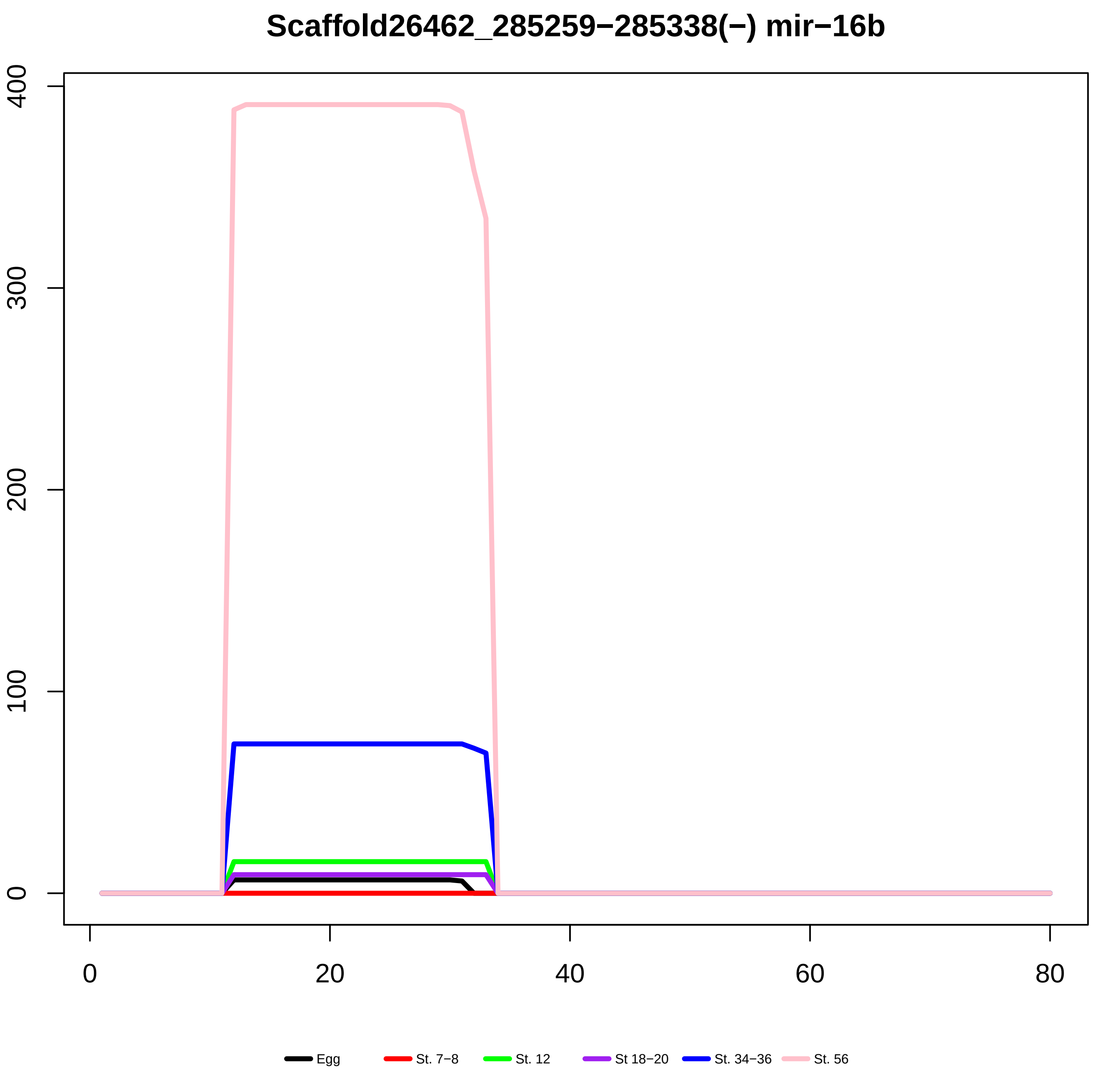

Scaffold26462_285259−285338(−) mir−16b
0
100
200
300
400
0
20
40
60
80
Egg
St. 7−8
St. 12
St 18−20
St. 34−36
St. 56

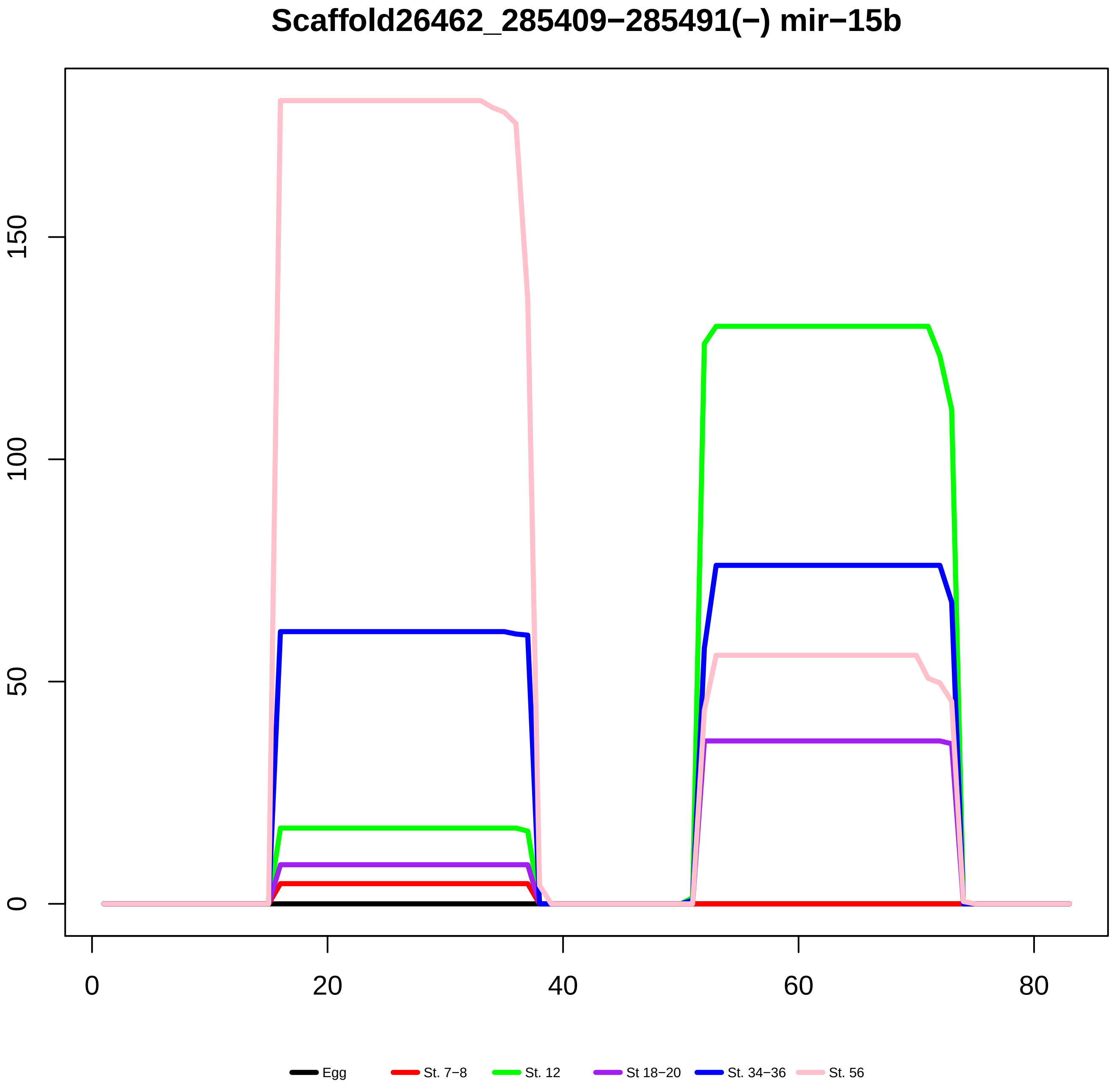
Scaffold26462_285409−285491(−) mir−15b
0
50
100
150
0
20
40
60
80
Egg
St. 7−8
St. 12
St 18−20
St. 34−36
St. 56

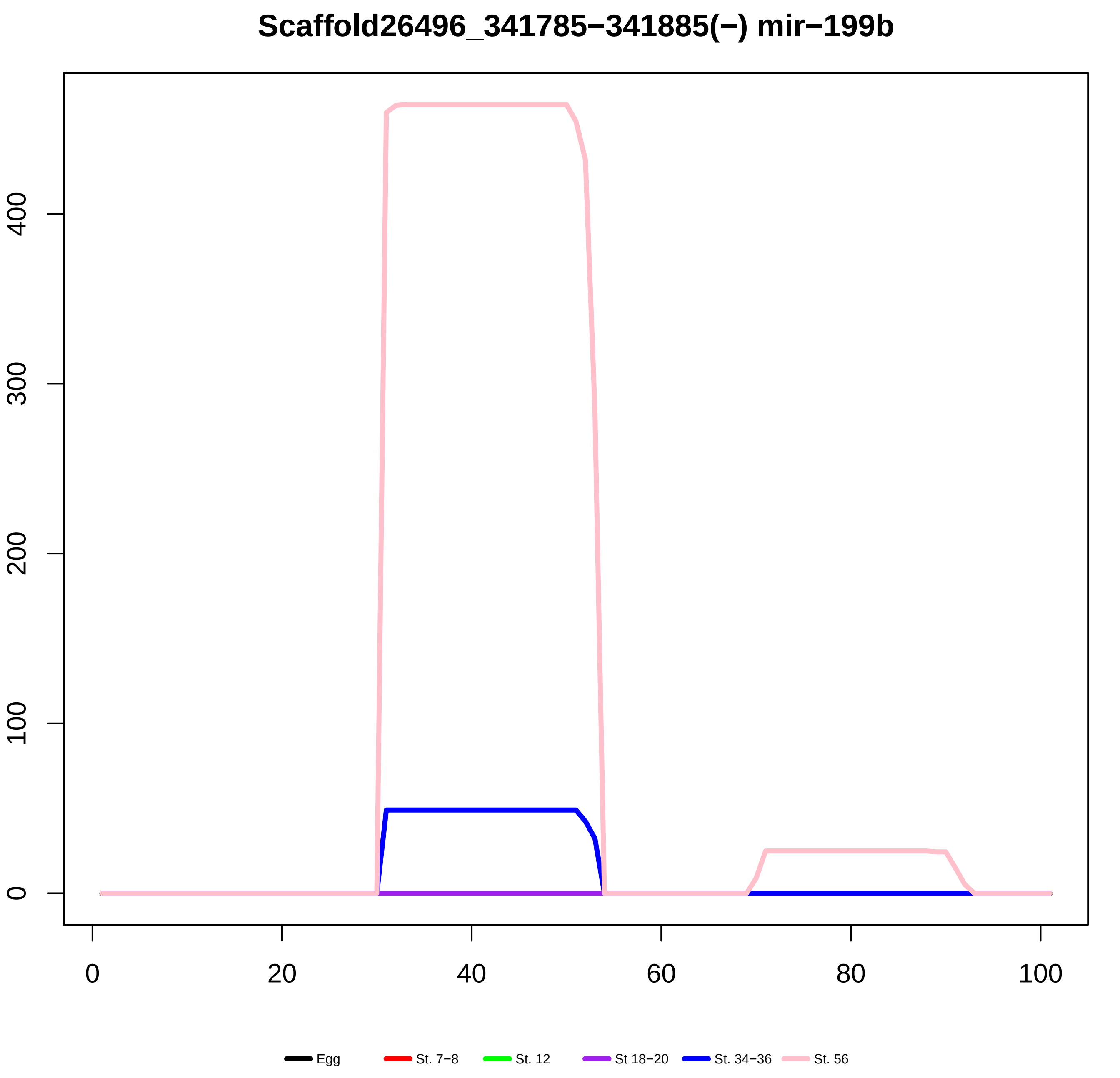
Scaffold26496_341785−341885(−) mir−199b
0
100
200
300
400
0
20
40
60
80
100
Egg
St. 7−8
St. 12
St 18−20
St. 34−36
St. 56

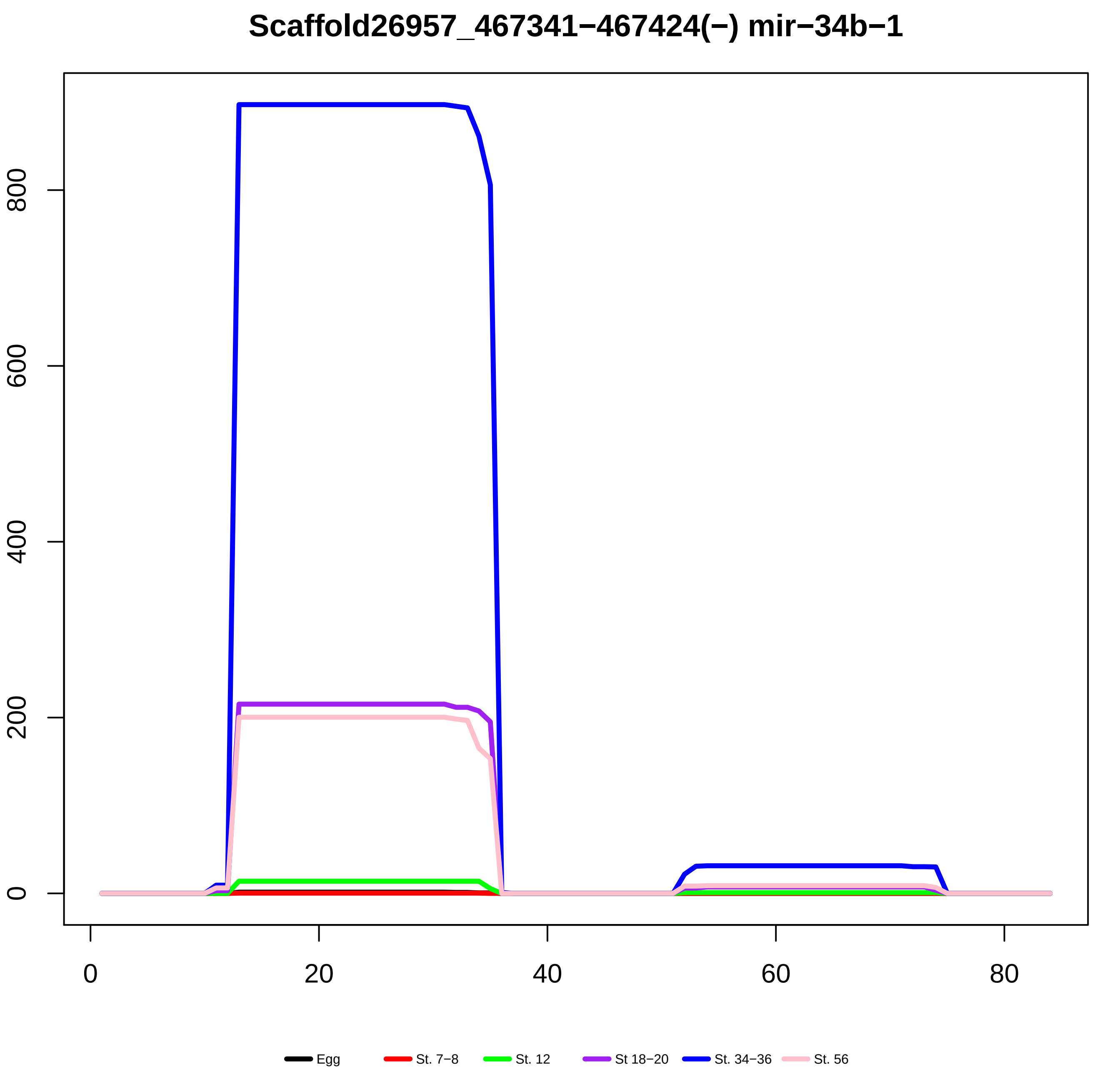
Scaffold26957_467341−467424(−) mir−34b−1
0
200
400
600
800
0
20
40
60
80
Egg
St. 7−8
St. 12
St 18−20
St. 34−36
St. 56

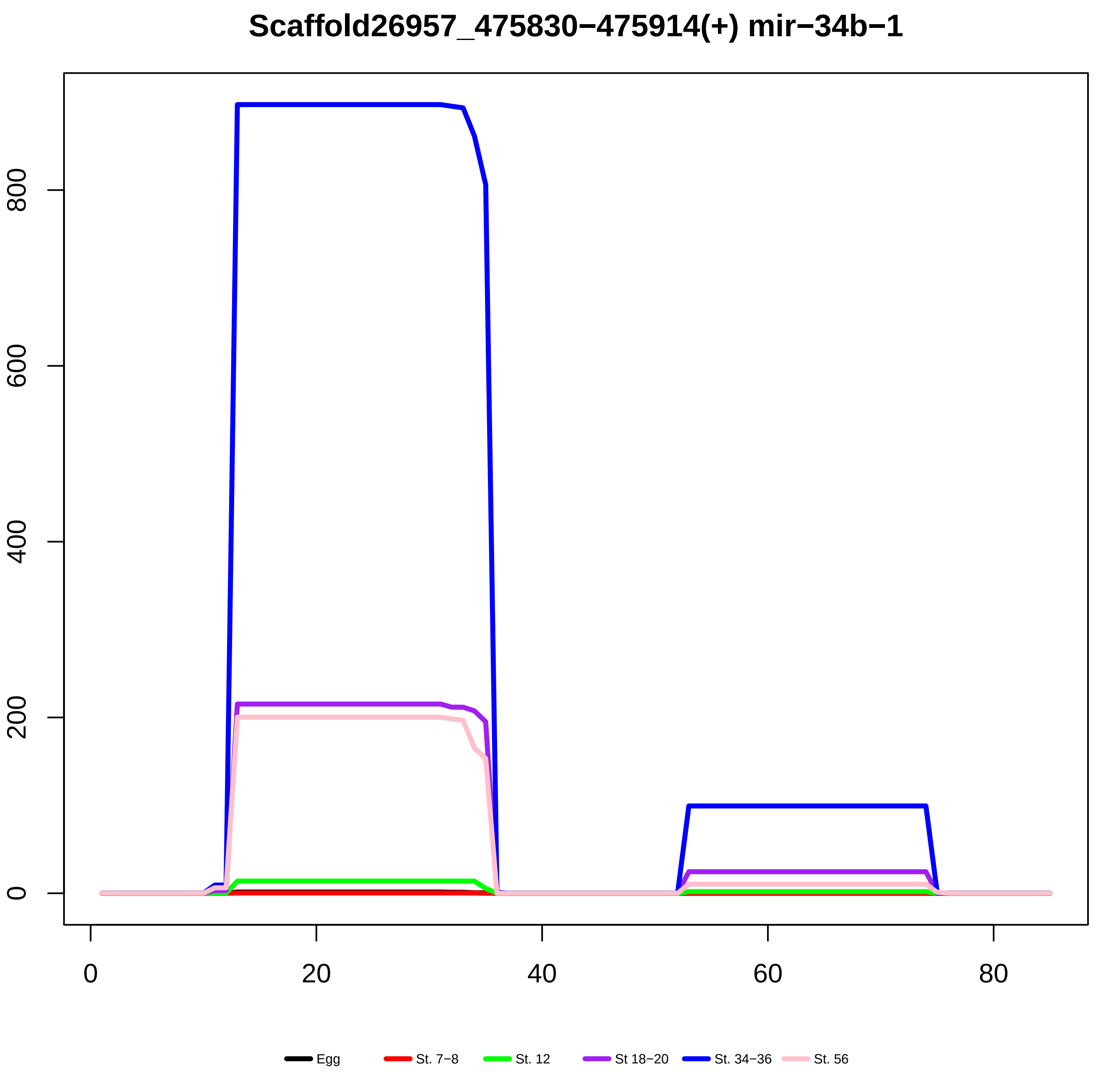

Scaffold26957_475830−475914(+) mir−34b−1
0
200
400
600
800
0
20
40
60
80
Egg
St. 7−8
St. 12
St 18−20
St. 34−36
St. 56

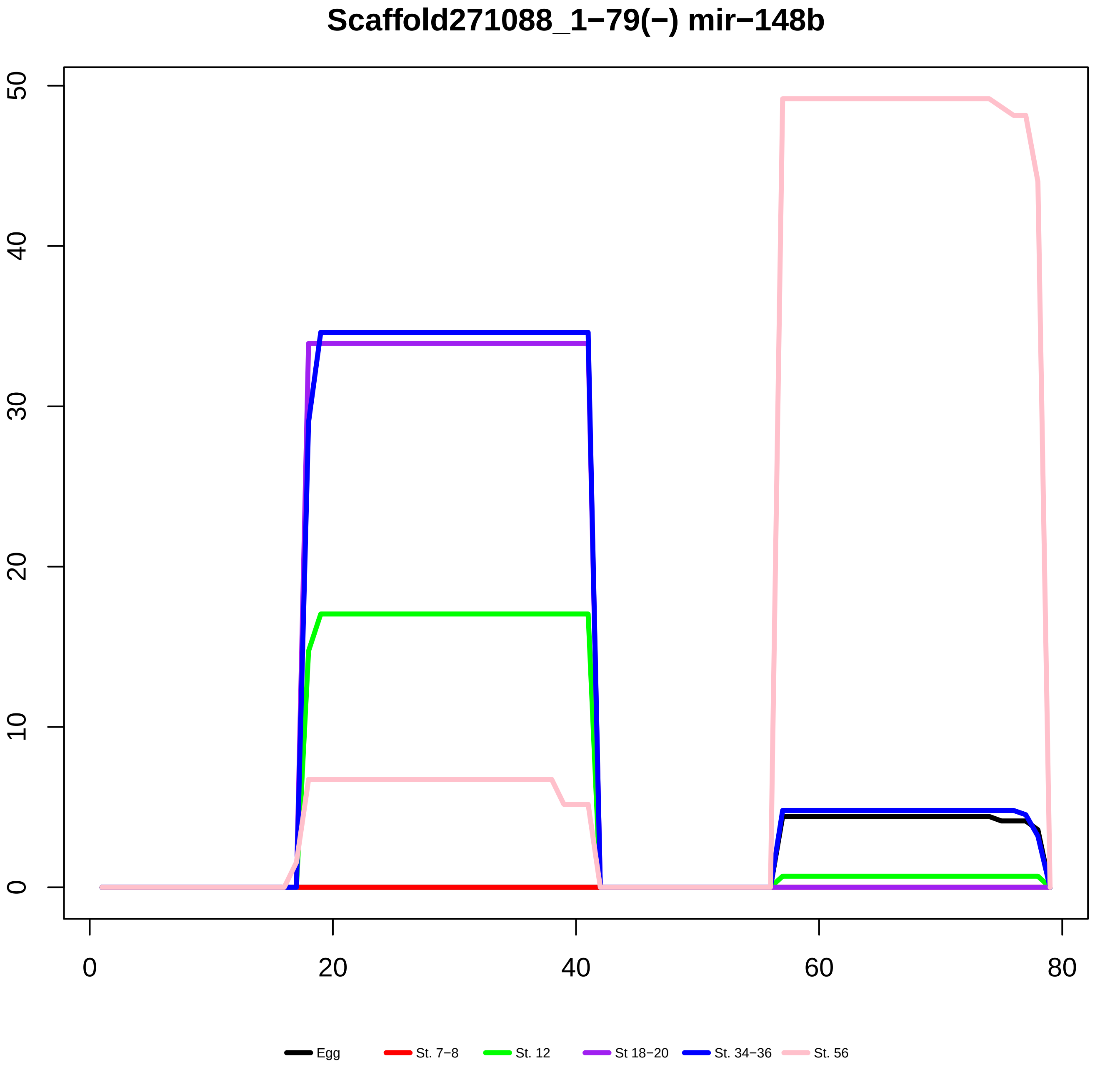

Scaffold271088_1−79(−) mir−148b
0
10
20
30
40
50
0
20
40
60
80
Egg
St. 7−8
St. 12
St 18−20
St. 34−36
St. 56

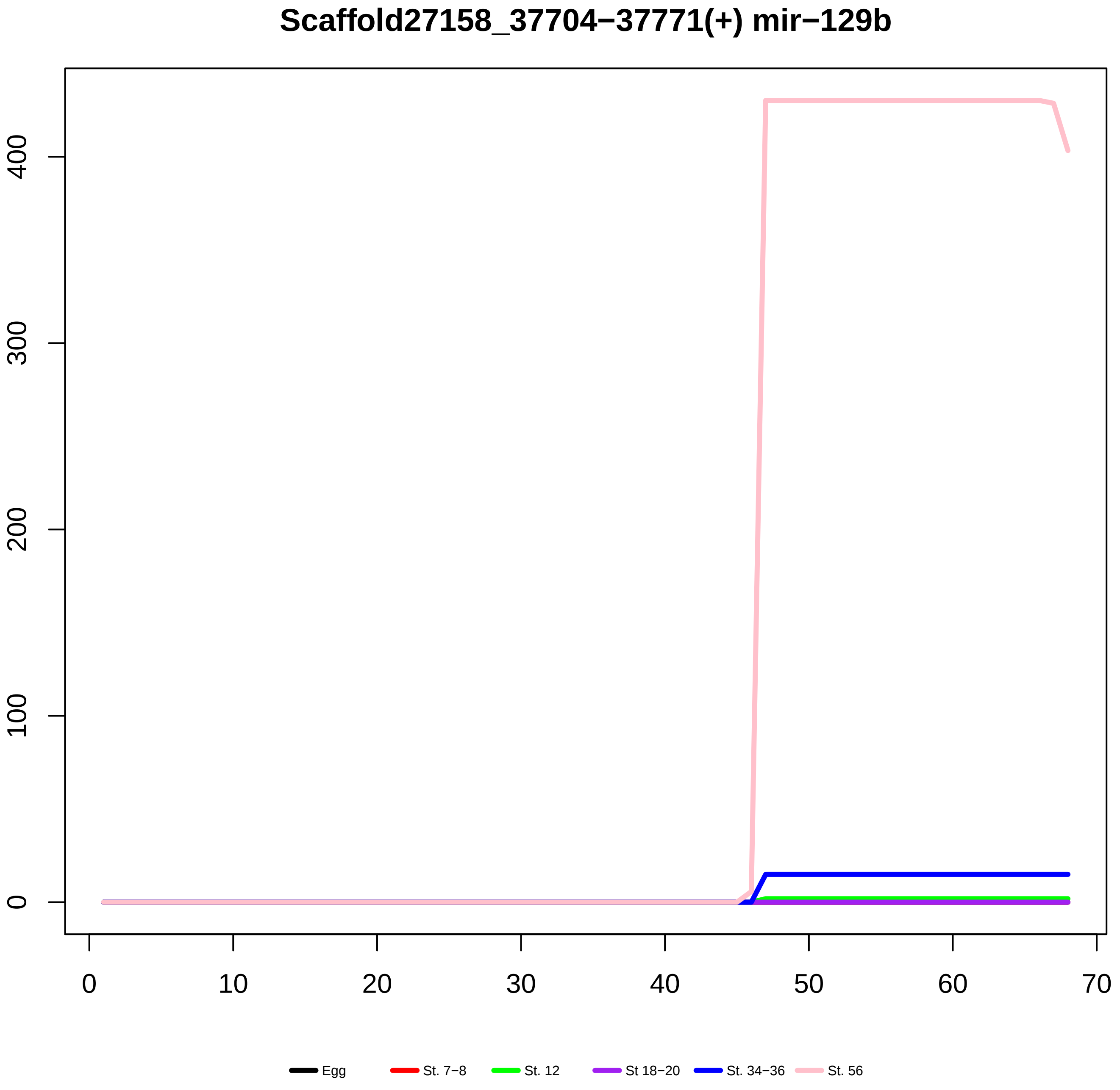

Scaffold27158_37704−37771(+) mir−129b
0
100
200
300
400
0
10
20
30
40
50
60
70
Egg
St. 7−8
St. 12
St 18−20
St. 34−36
St. 56

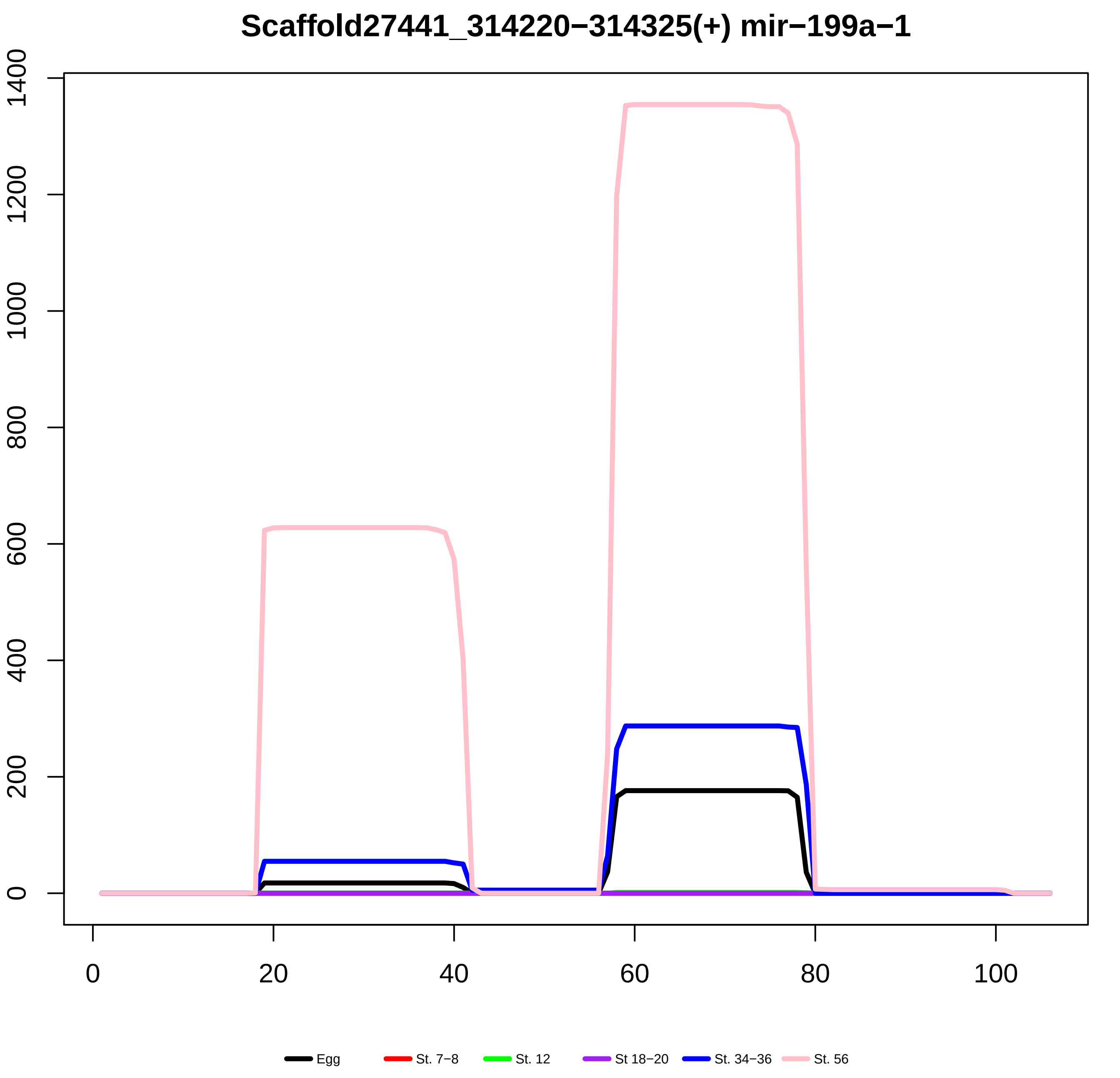

Scaffold27441_314220−314325(+) mir−199a−1
0
200
400
600
800
1000
1200
1400
0
20
40
60
80
100
Egg
St. 7−8
St. 12
St 18−20
St. 34−36
St. 56

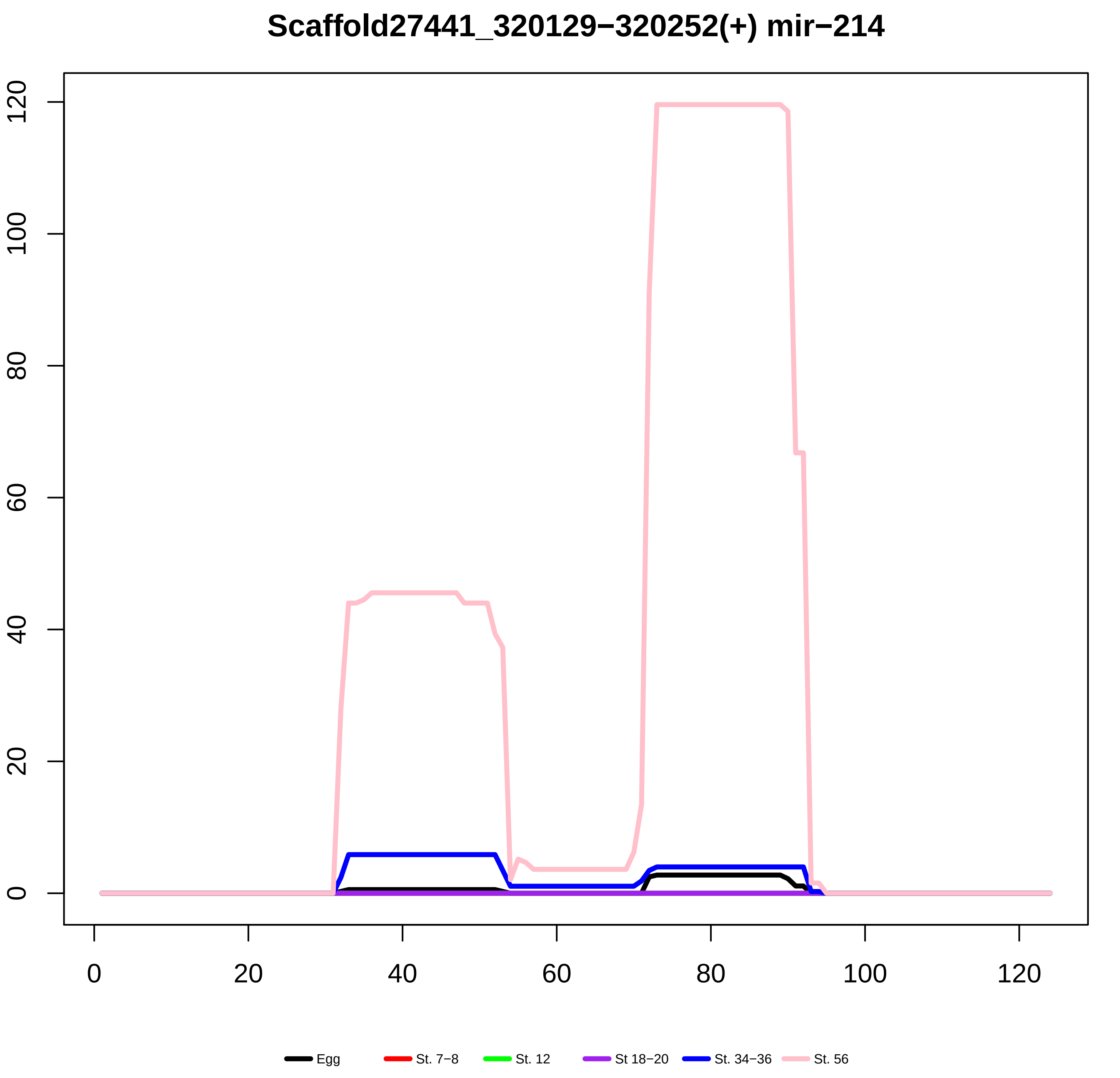

Scaffold27441_320129−320252(+) mir−214
0
20
40
60
80
100
120
0
20
40
60
80
100
120
Egg
St. 7−8
St. 12
St 18−20
St. 34−36
St. 56

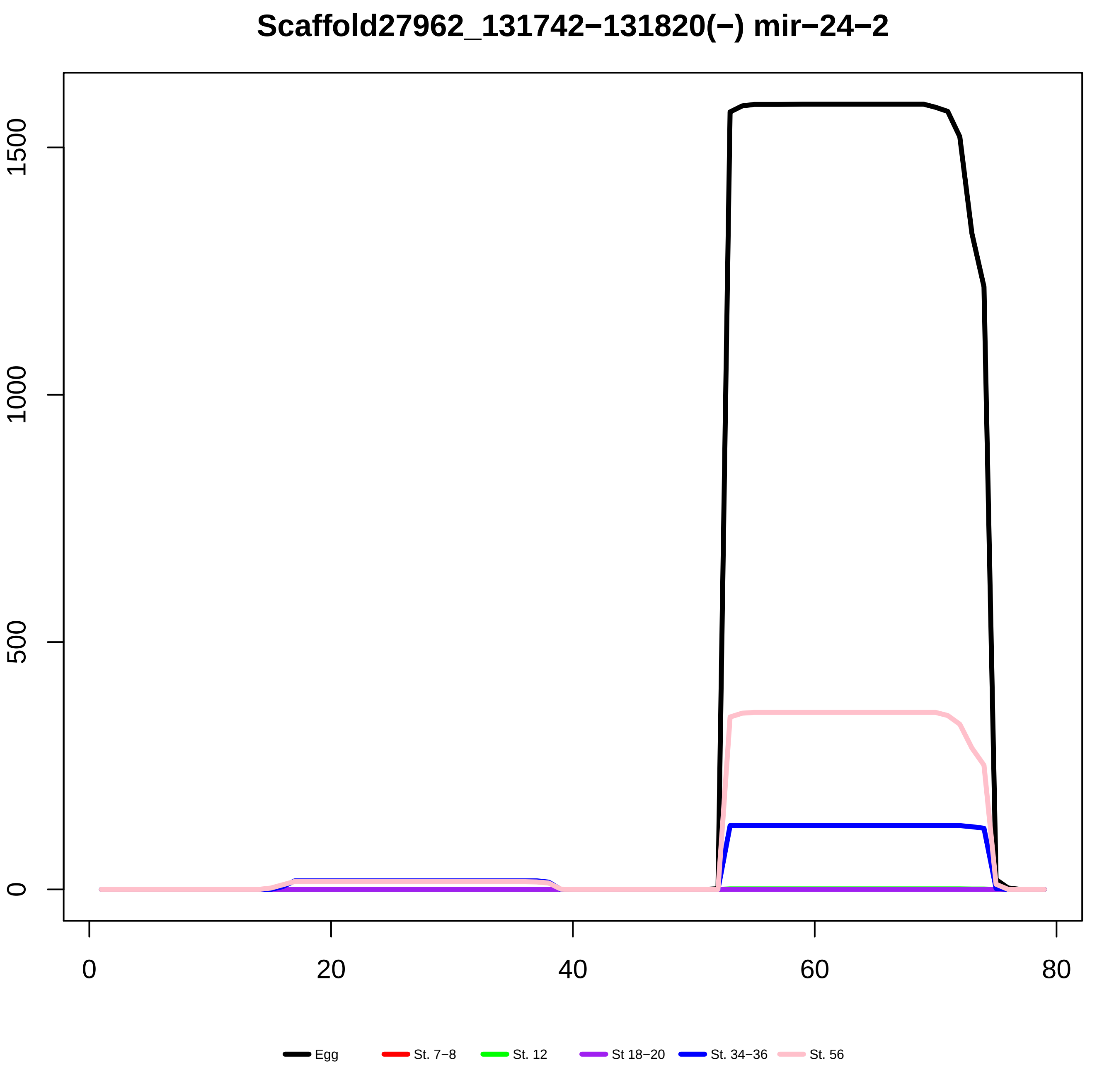

Scaffold27962_131742−131820(−) mir−24−2
0
500
1000
1500
0
20
40
60
80
Egg
St. 7−8
St. 12
St 18−20
St. 34−36
St. 56

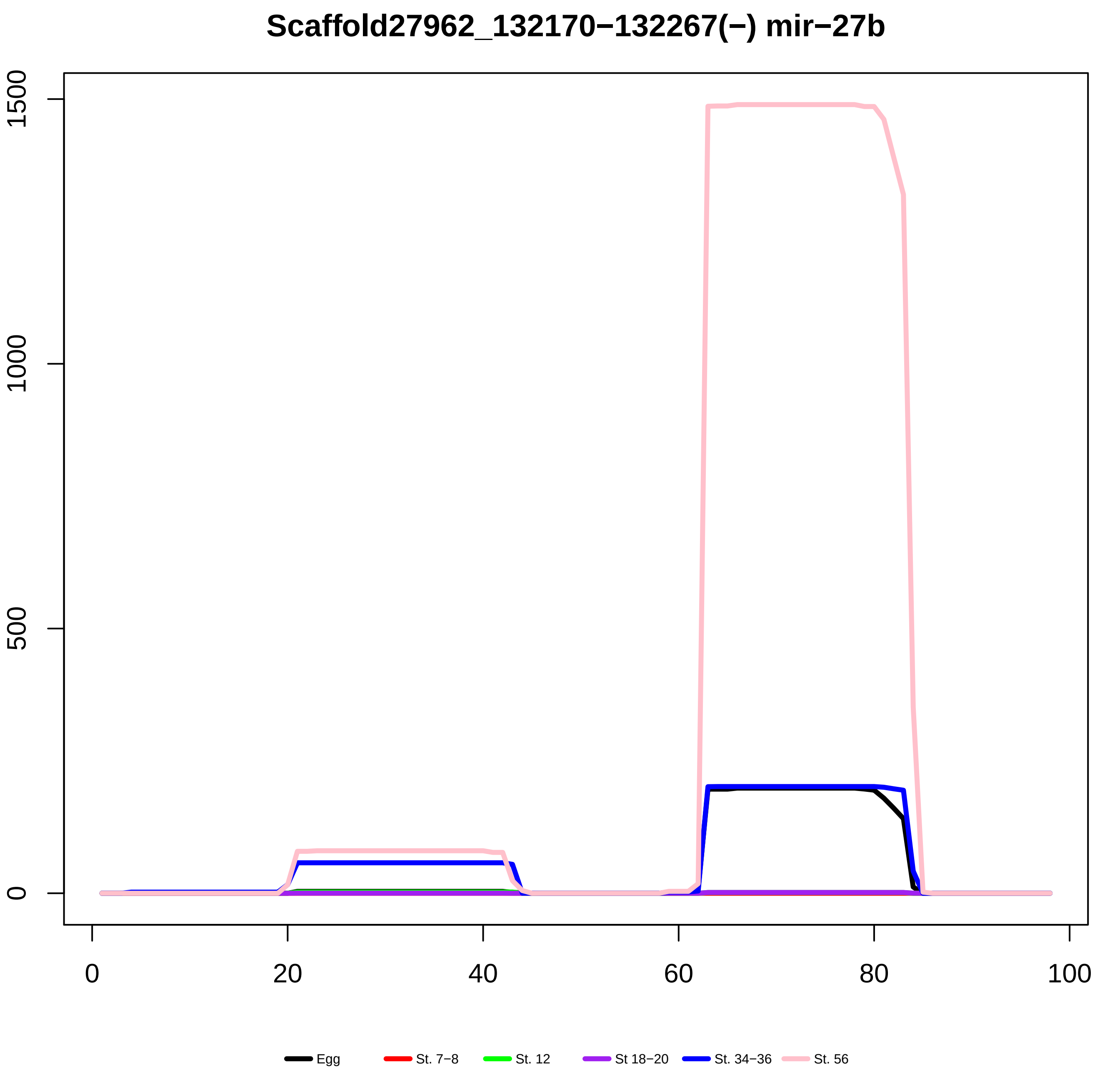

Scaffold27962_132170−132267(−) mir−27b
0
500
1000
1500
0
20
40
60
80
100
Egg
St. 7−8
St. 12
St 18−20
St. 34−36
St. 56

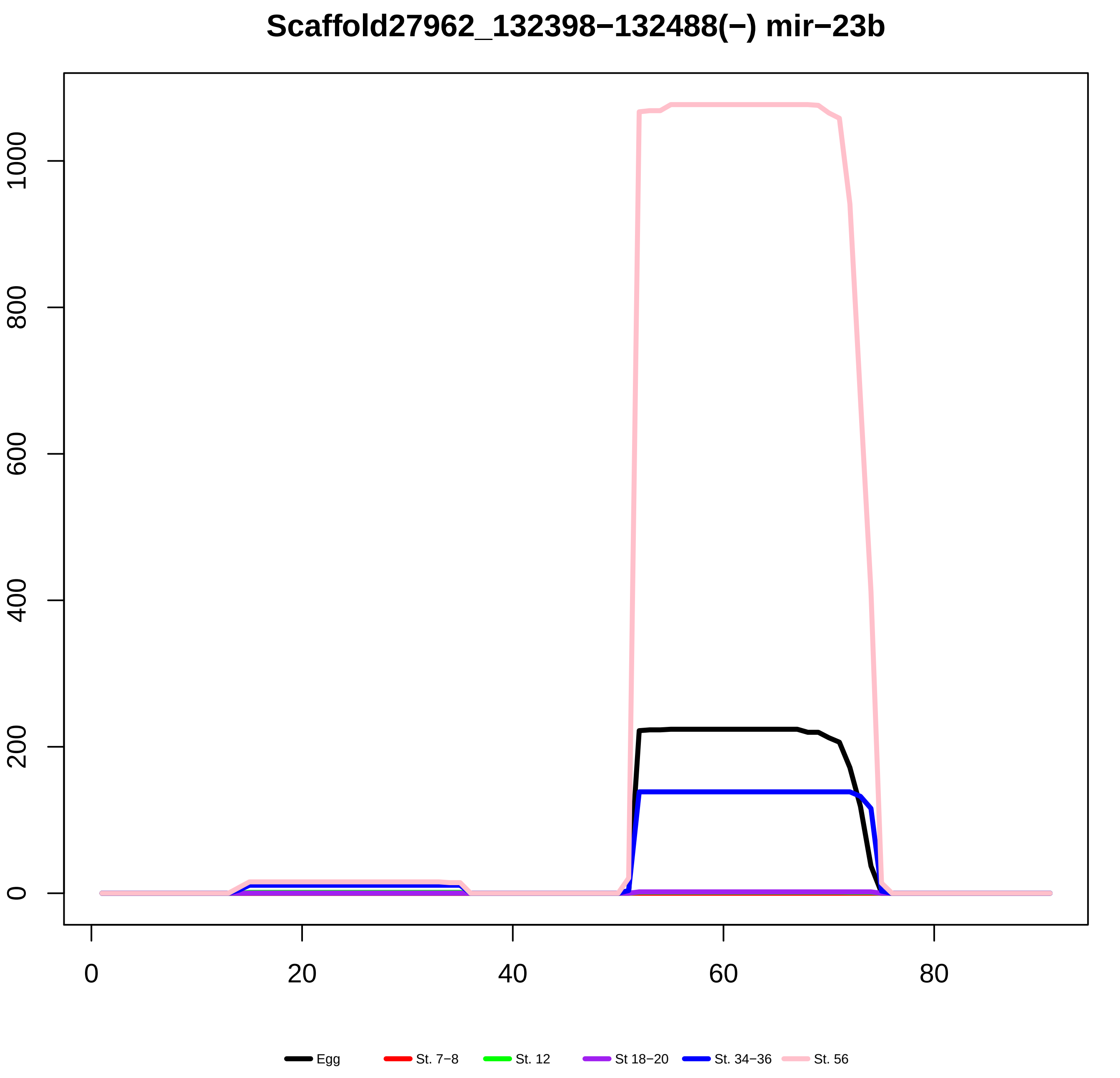
Scaffold27962_132398−132488(−) mir−23b
0
200
400
600
800
1000
0
20
40
60
80
Egg
St. 7−8
St. 12
St 18−20
St. 34−36
St. 56

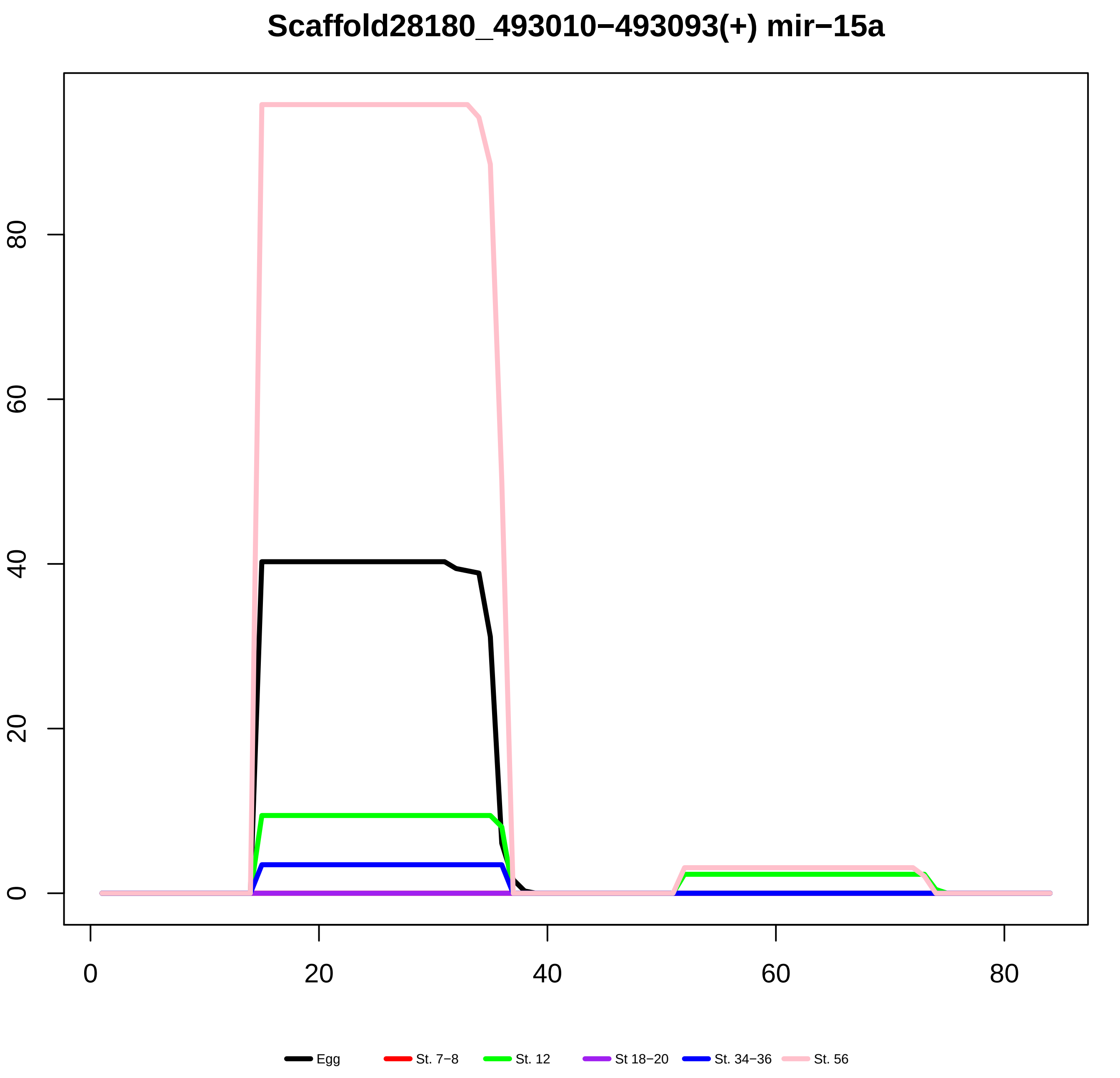

Scaffold28180_493010−493093(+) mir−15a
0
20
40
60
80
0
20
40
60
80
Egg
St. 7−8
St. 12
St 18−20
St. 34−36
St. 56

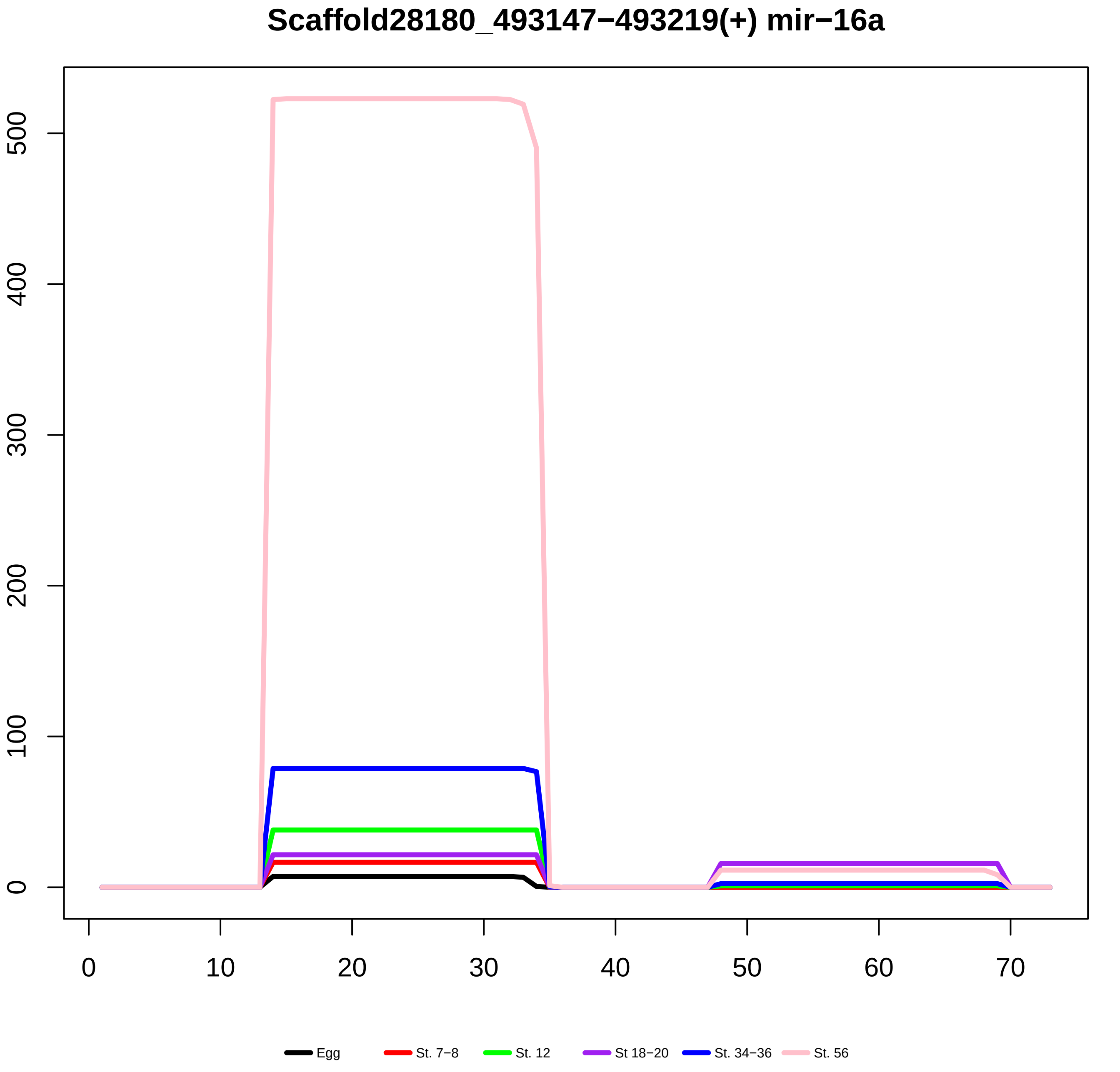

Scaffold28180_493147−493219(+) mir−16a
0
100
200
300
400
500
0
10
20
30
40
50
60
70
Egg
St. 7−8
St. 12
St 18−20
St. 34−36
St. 56

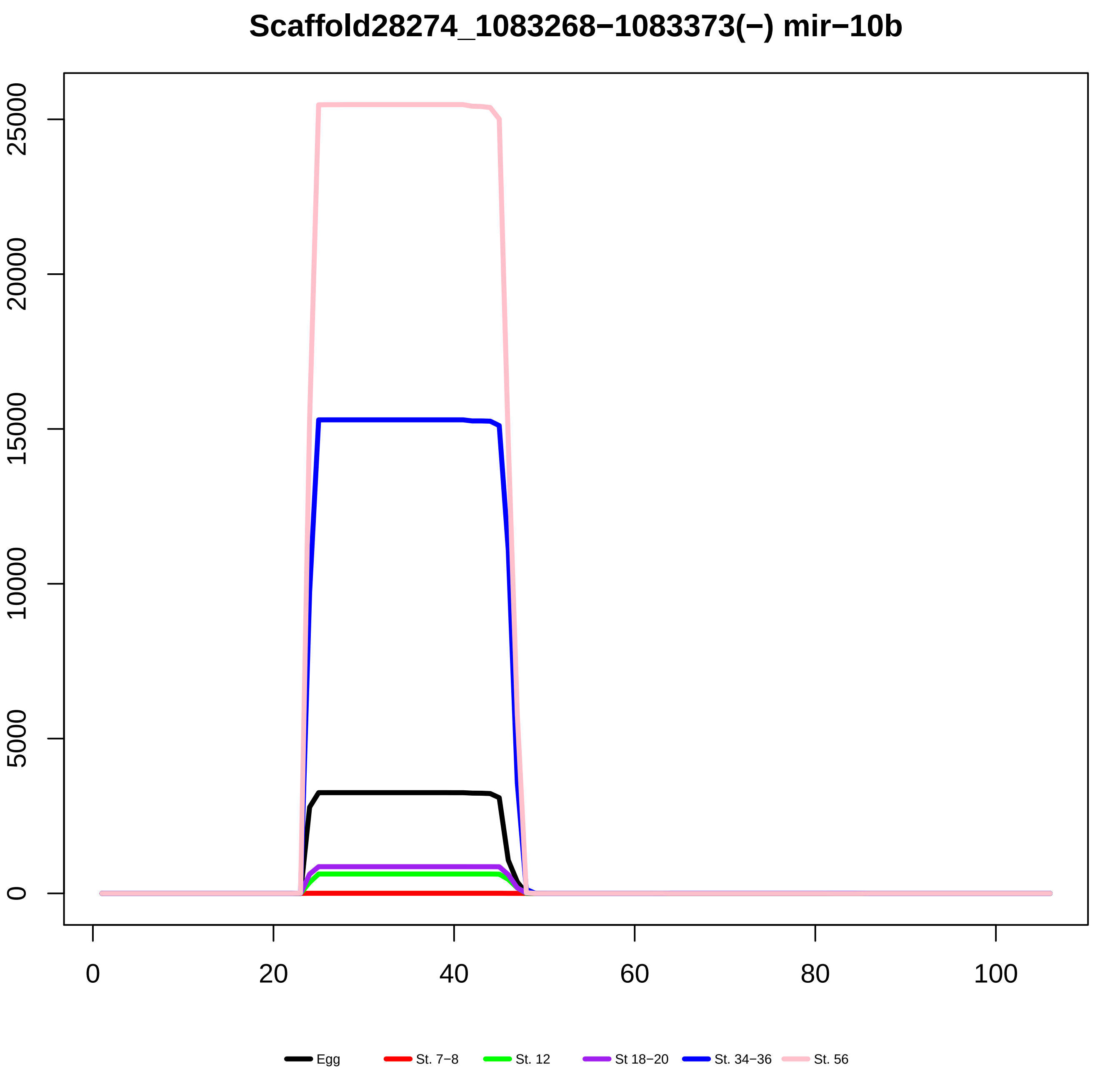

Scaffold28274_1083268−1083373(−) mir−10b
0
5000
10000
15000
20000
25000
0
20
40
60
80
100
Egg
St. 7−8
St. 12
St 18−20
St. 34−36
St. 56

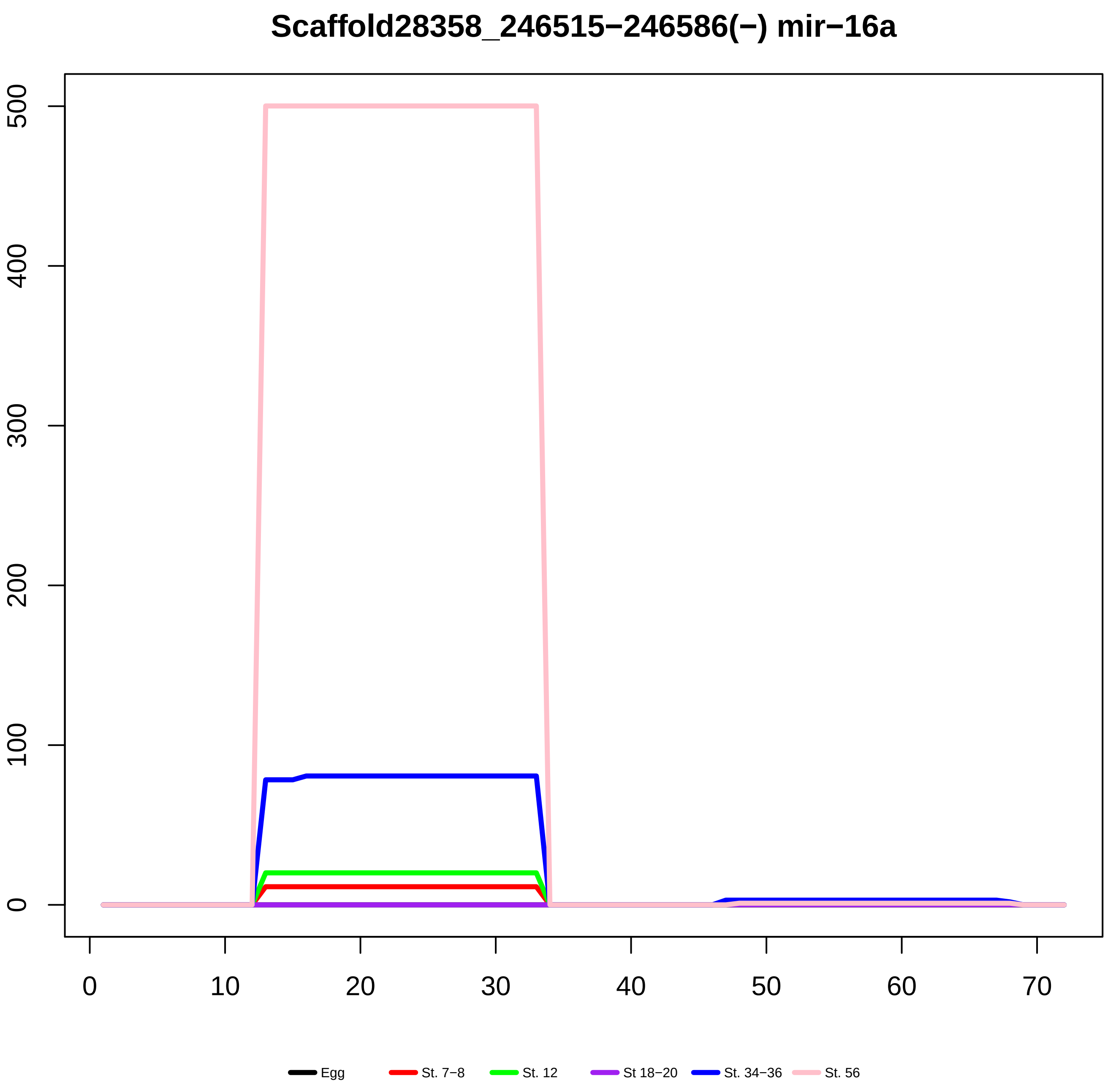

Scaffold28358_246515−246586(−) mir−16a
0
100
200
300
400
500
0
10
20
30
40
50
60
70
Egg
St. 7−8
St. 12
St 18−20
St. 34−36
St. 56

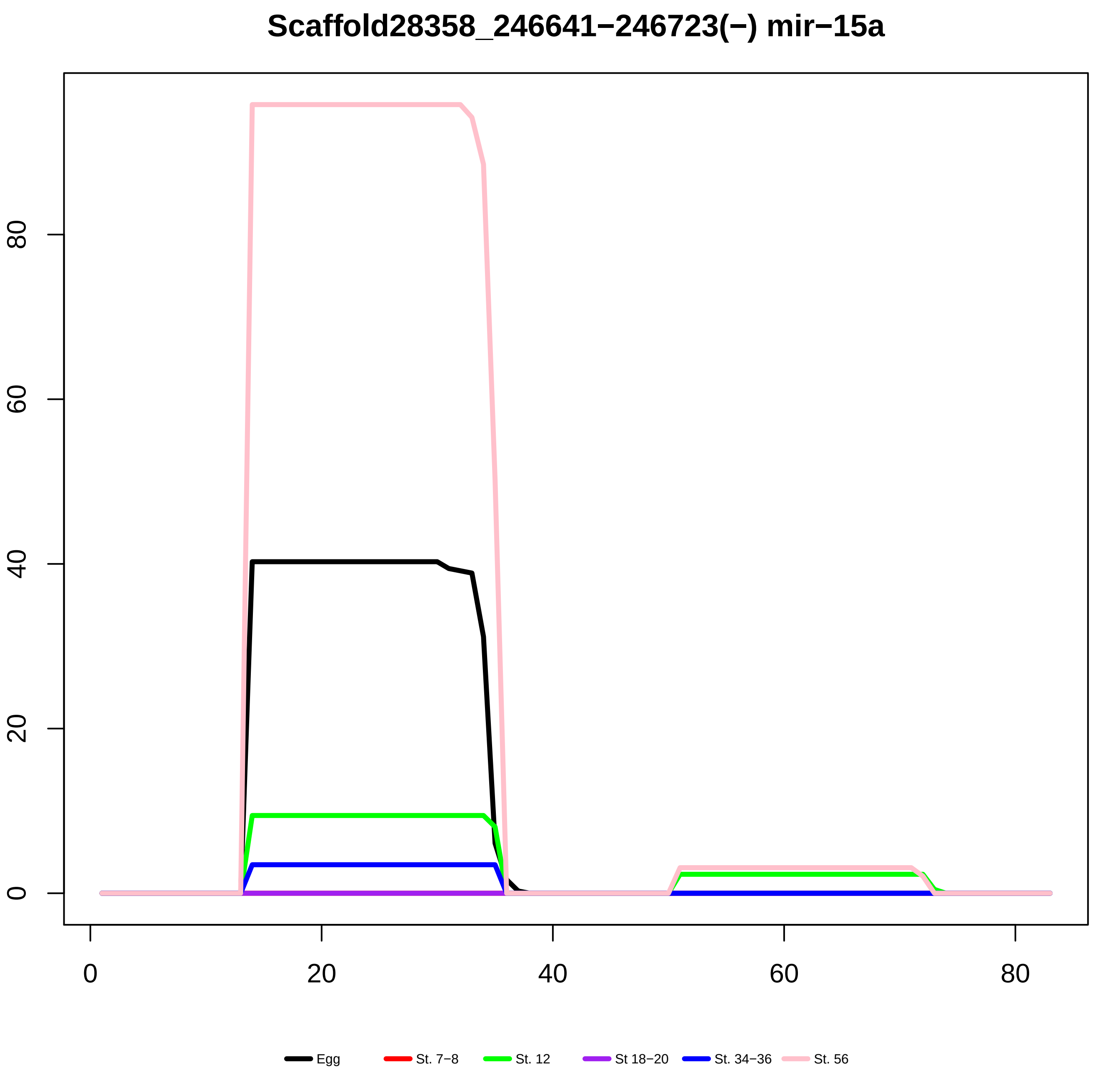

Scaffold28358_246641−246723(−) mir−15a
0
20
40
60
80
0
20
40
60
80
Egg
St. 7−8
St. 12
St 18−20
St. 34−36
St. 56

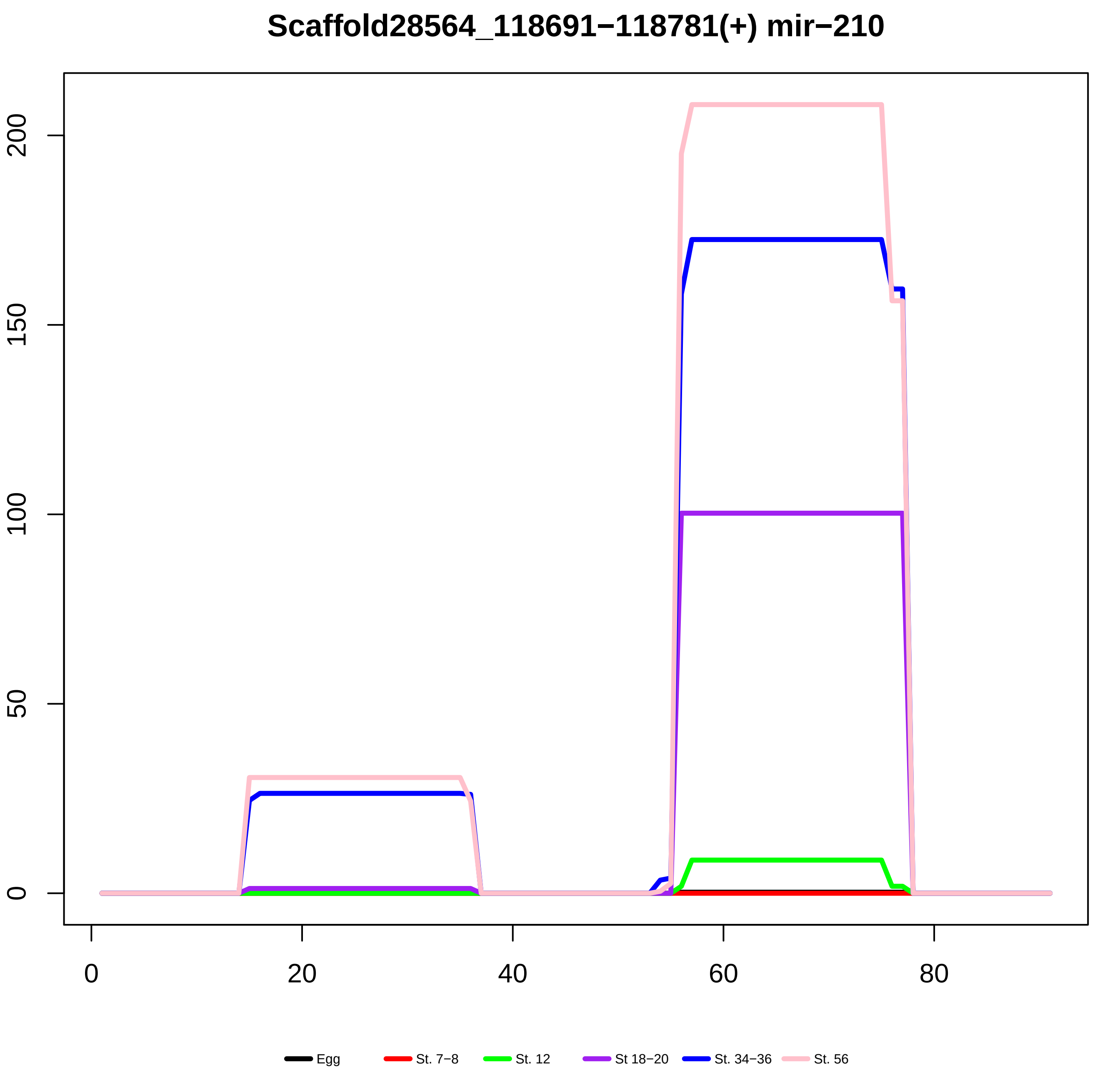
Scaffold28564_118691−118781(+) mir−210
0
50
100
150
200
0
20
40
60
80
Egg
St. 7−8
St. 12
St 18−20
St. 34−36
St. 56

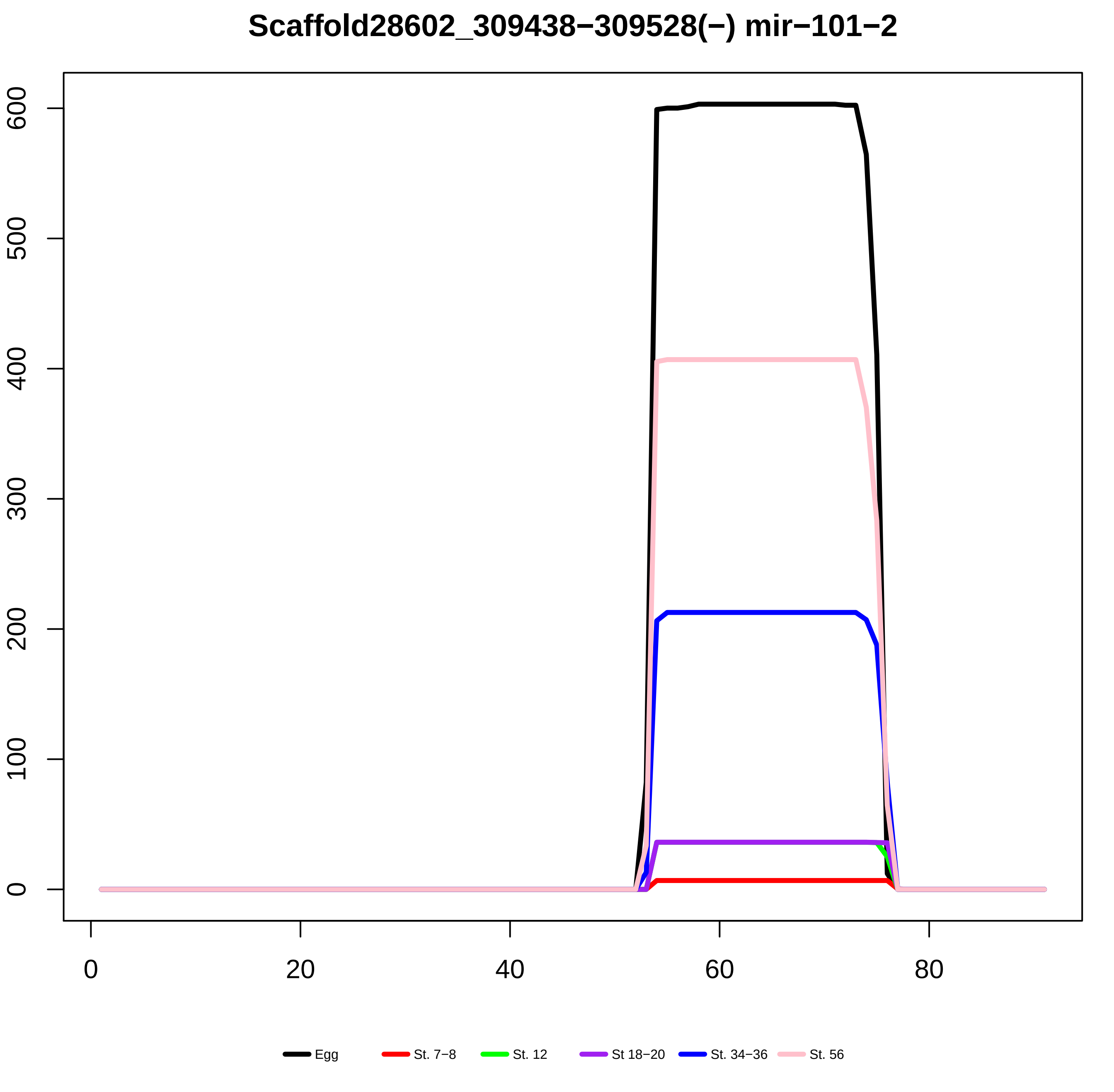

Scaffold28602_309438−309528(−) mir−101−2
0
100
200
300
400
500
600
0
20
40
60
80
Egg
St. 7−8
St. 12
St 18−20
St. 34−36
St. 56

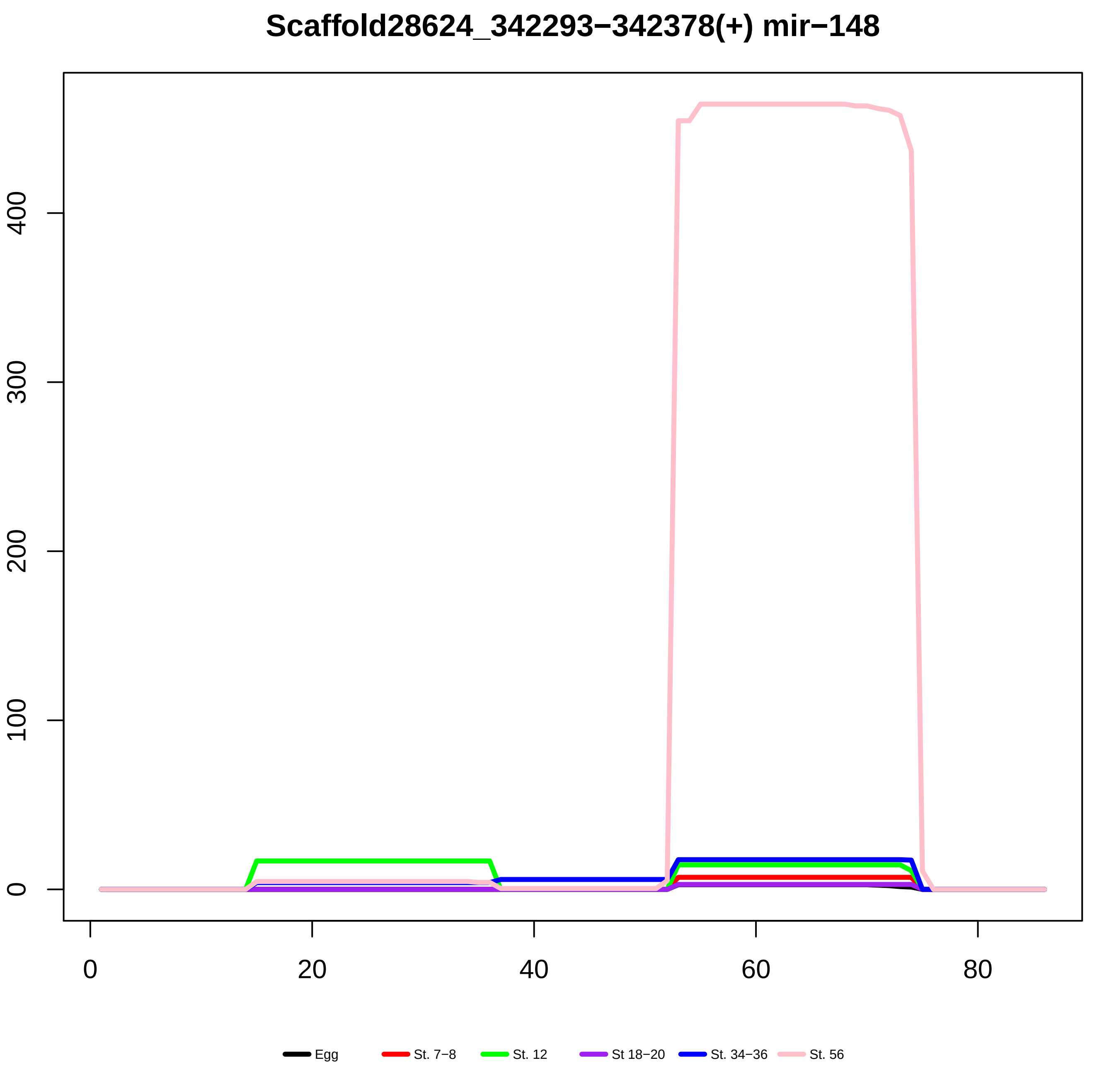

Scaffold28624_342293−342378(+) mir−148
0
100
200
300
400
0
20
40
60
80
Egg
St. 7−8
St. 12
St 18−20
St. 34−36
St. 56

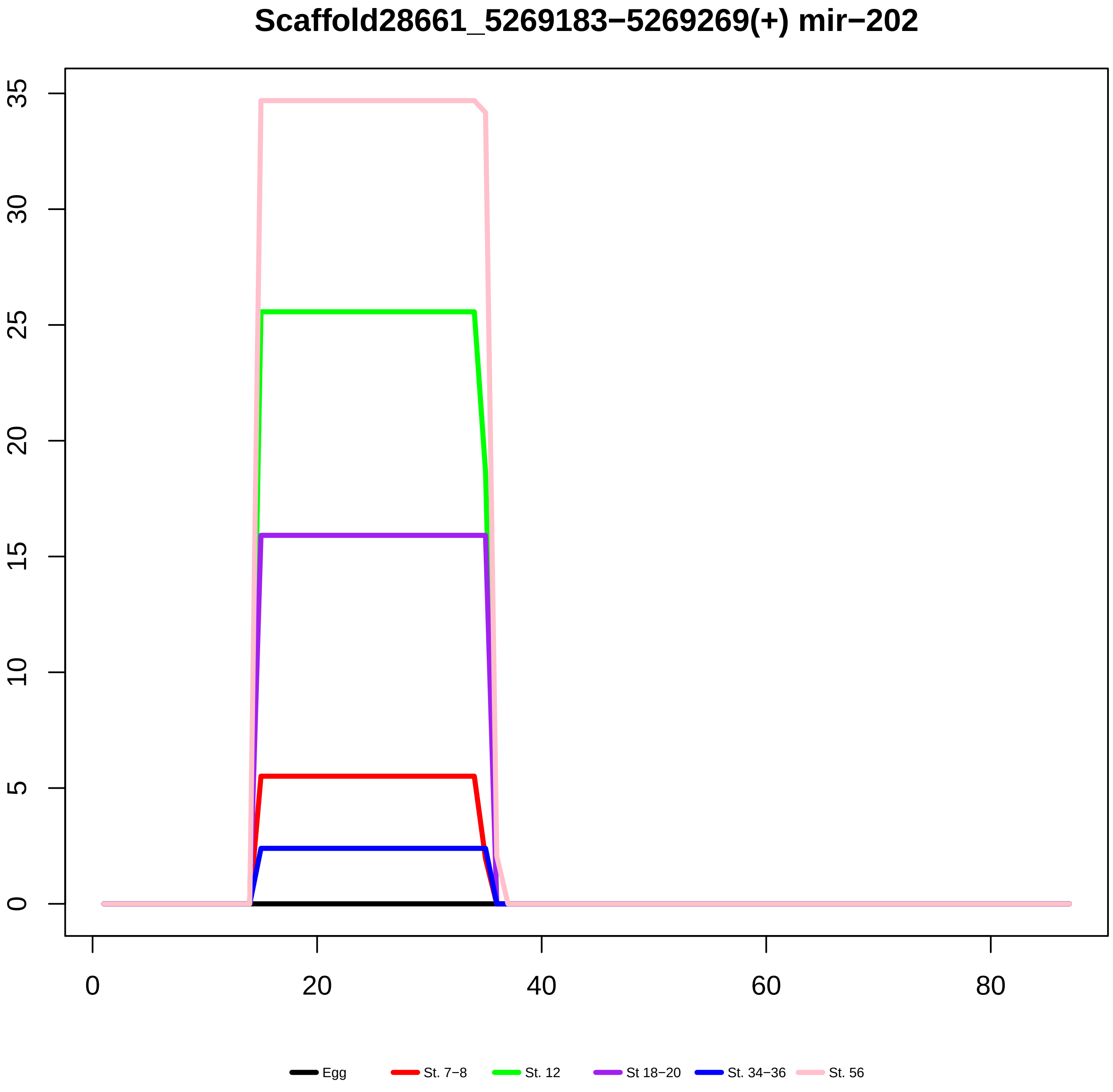

Scaffold28661_5269183−5269269(+) mir−202
0
5
10
15
20
25
30
35
0
20
40
60
80
Egg
St. 7−8
St. 12
St 18−20
St. 34−36
St. 56

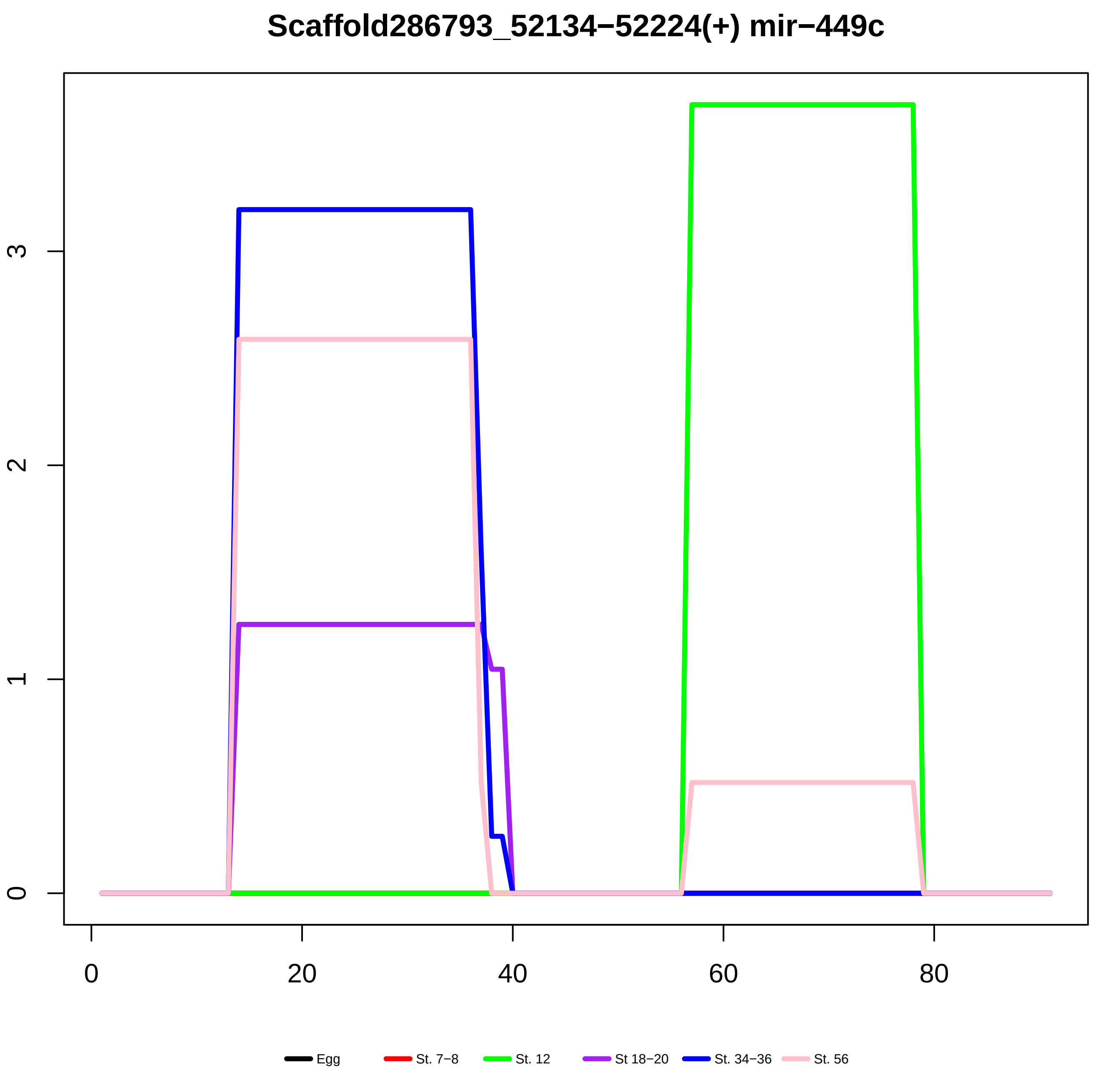
Scaffold286793_52134−52224(+) mir−449c
0
1
2
3
0
20
40
60
80
Egg
St. 7−8
St. 12
St 18−20
St. 34−36
St. 56

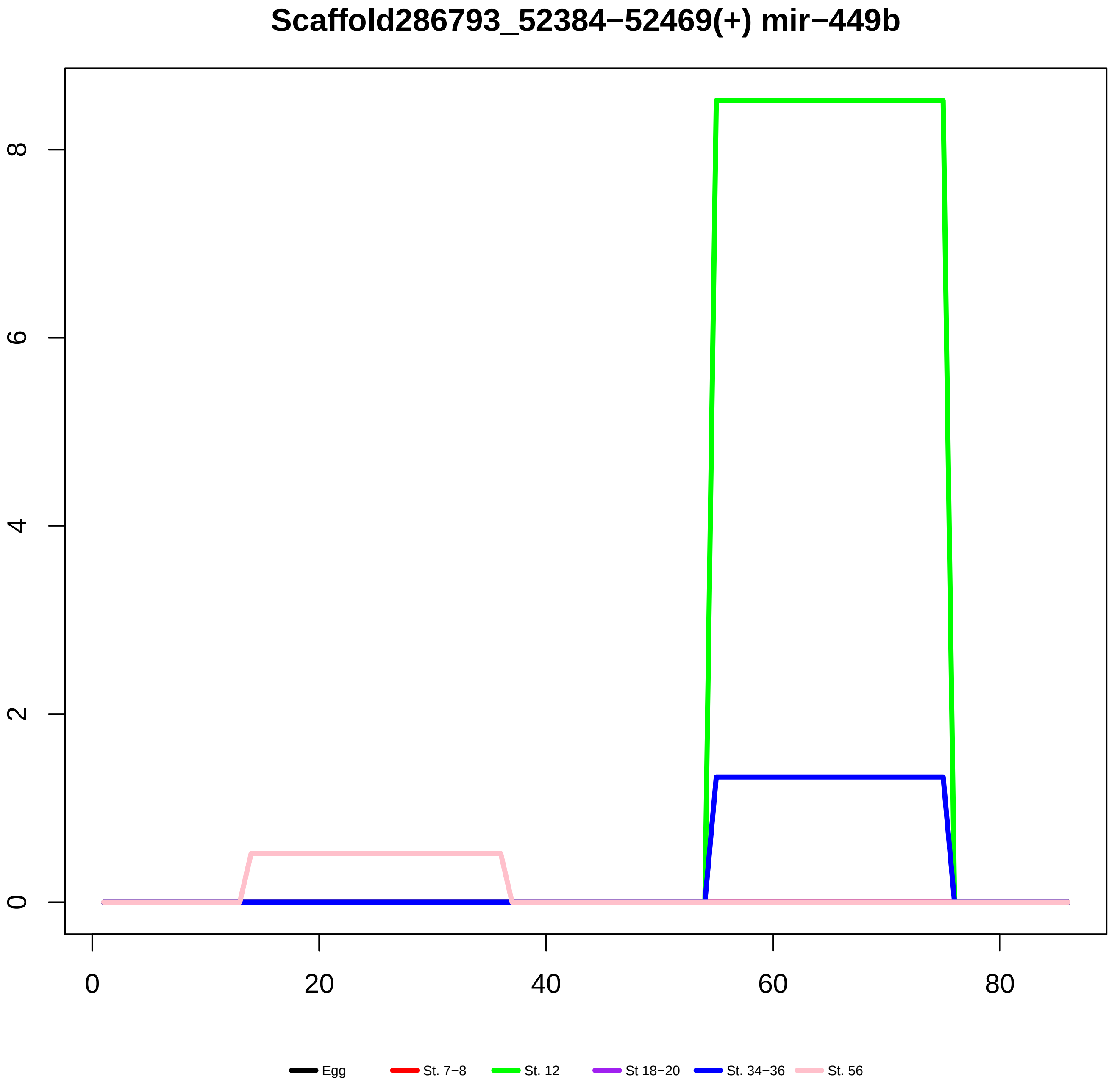

Scaffold286793_52384−52469(+) mir−449b
0
2
4
6
8
0
20
40
60
80
Egg
St. 7−8
St. 12
St 18−20
St. 34−36
St. 56

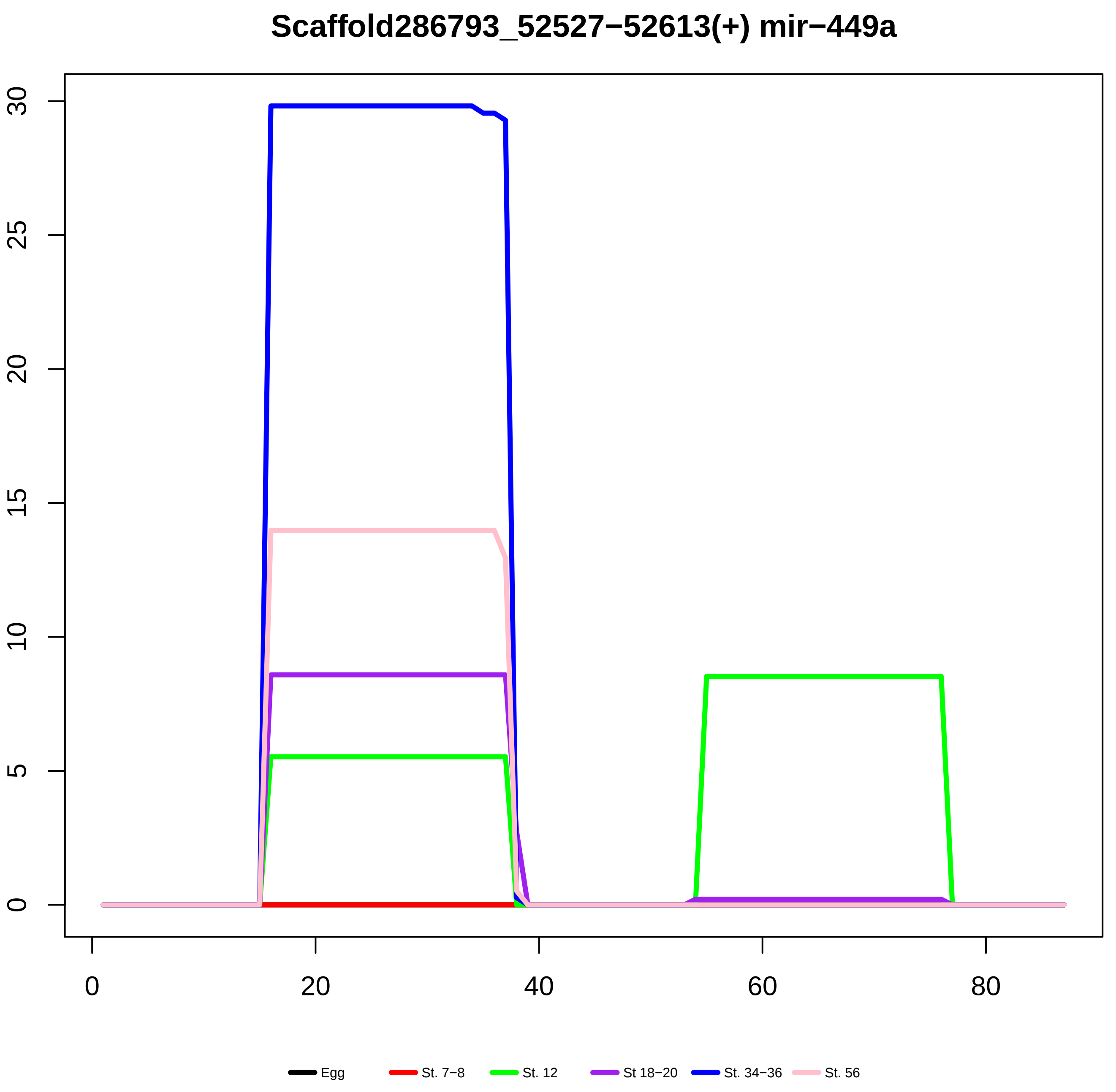

Scaffold286793_52527−52613(+) mir−449a
0
5
10
15
20
25
30
0
20
40
60
80
Egg
St. 7−8
St. 12
St 18−20
St. 34−36
St. 56

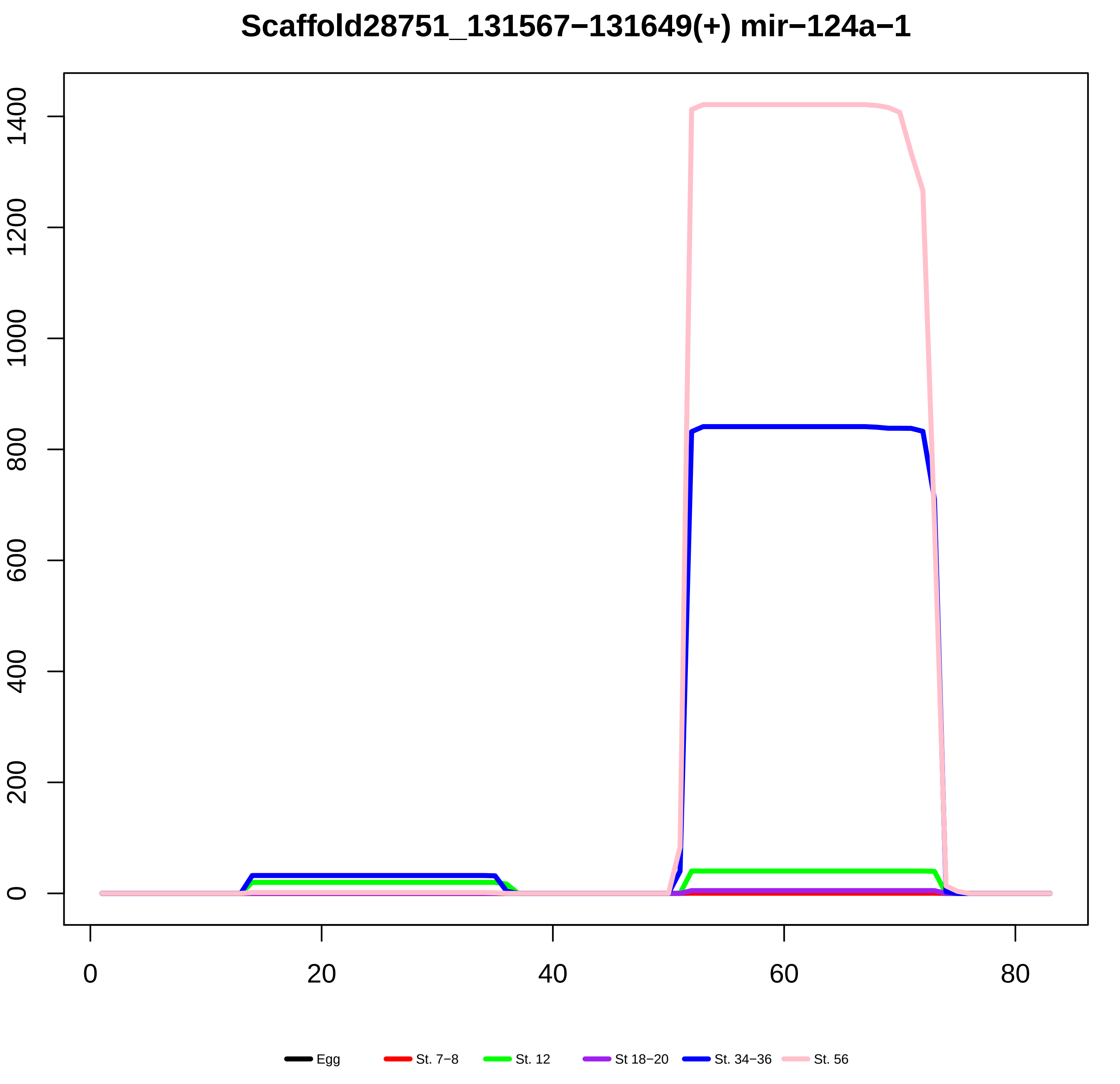

Scaffold28751_131567−131649(+) mir−124a−1
0
200
400
600
800
1000
1200
1400
0
20
40
60
80
Egg
St. 7−8
St. 12
St 18−20
St. 34−36
St. 56

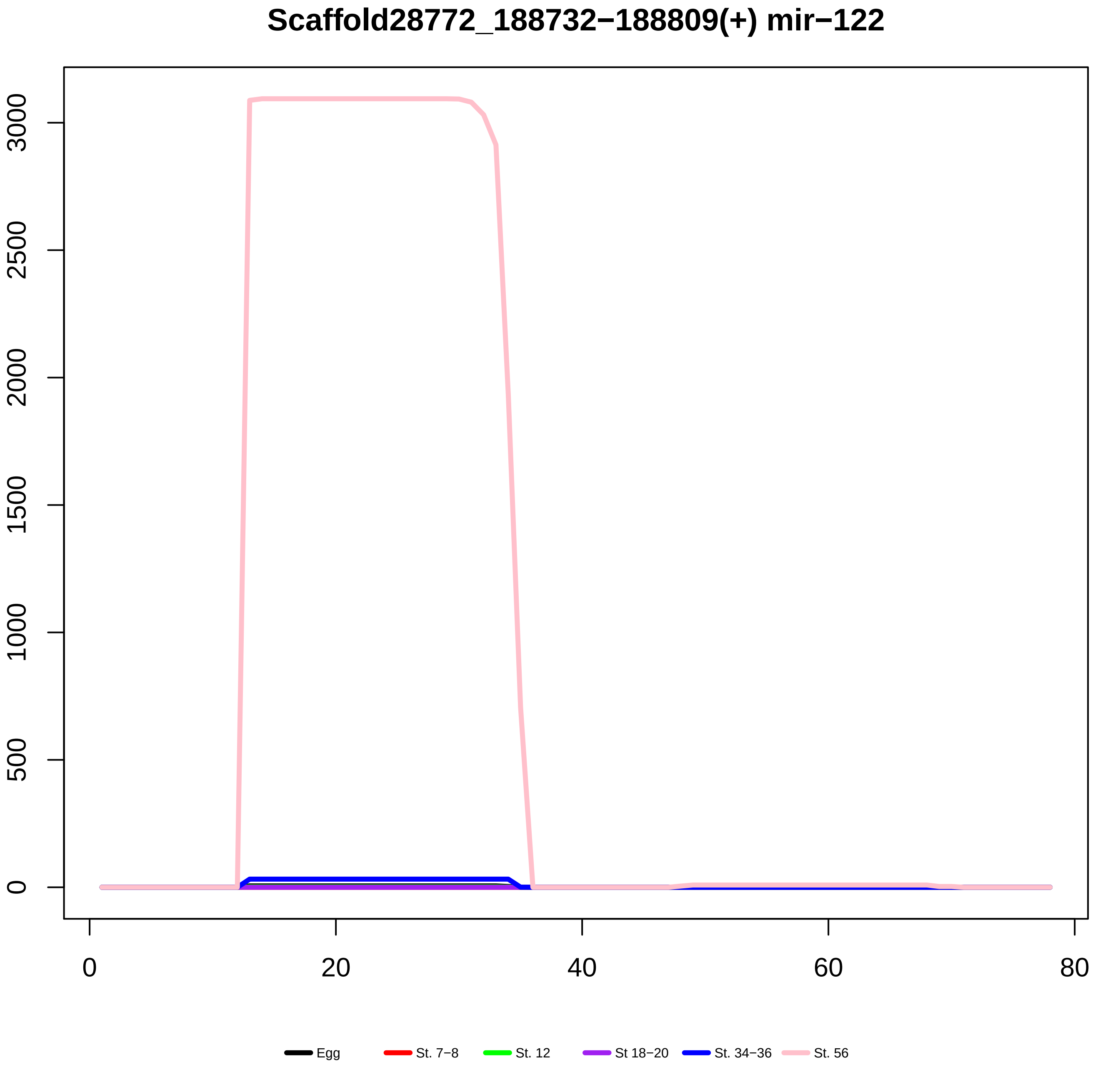
Scaffold28772_188732−188809(+) mir−122
0
500
1000
1500
2000
2500
3000
0
20
40
60
80
Egg
St. 7−8
St. 12
St 18−20
St. 34−36
St. 56

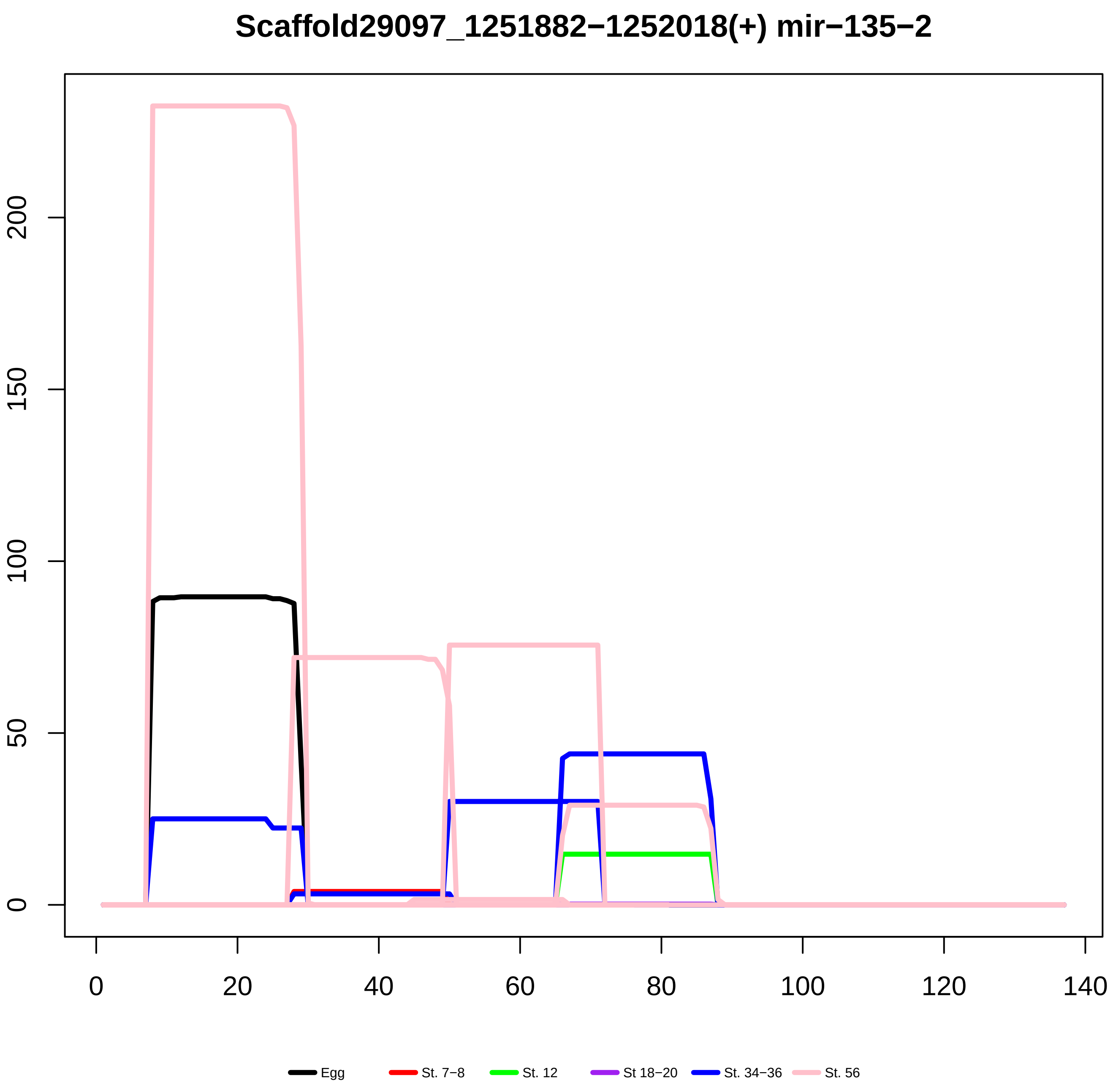

Scaffold29097_1251882−1252018(+) mir−135−2
0
50
100
150
200
0
20
40
60
80
100
120
140
Egg
St. 7−8
St. 12
St 18−20
St. 34−36
St. 56

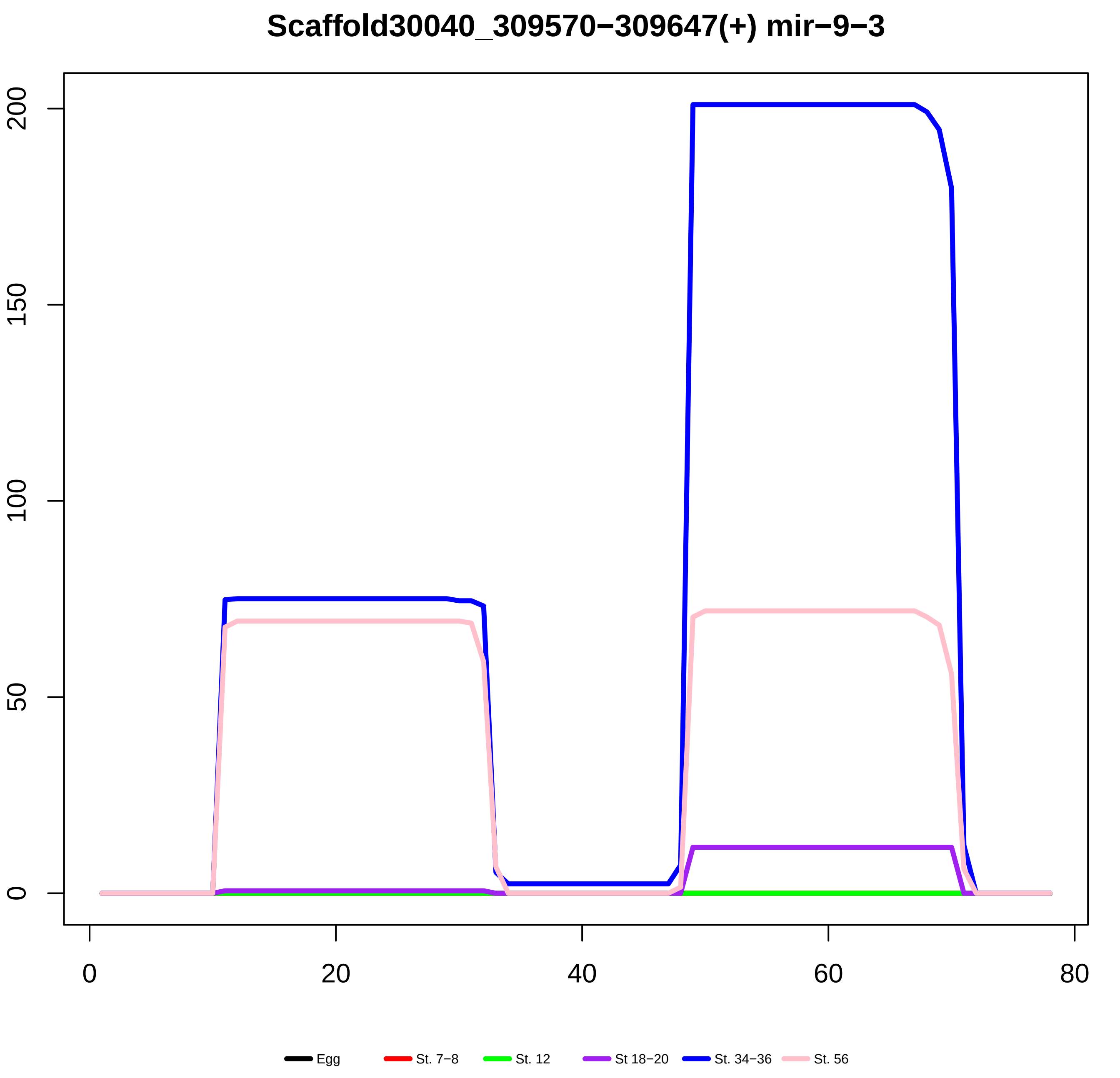

Scaffold30040_309570−309647(+) mir−9−3
0
50
100
150
200
0
20
40
60
80
Egg
St. 7−8
St. 12
St 18−20
St. 34−36
St. 56

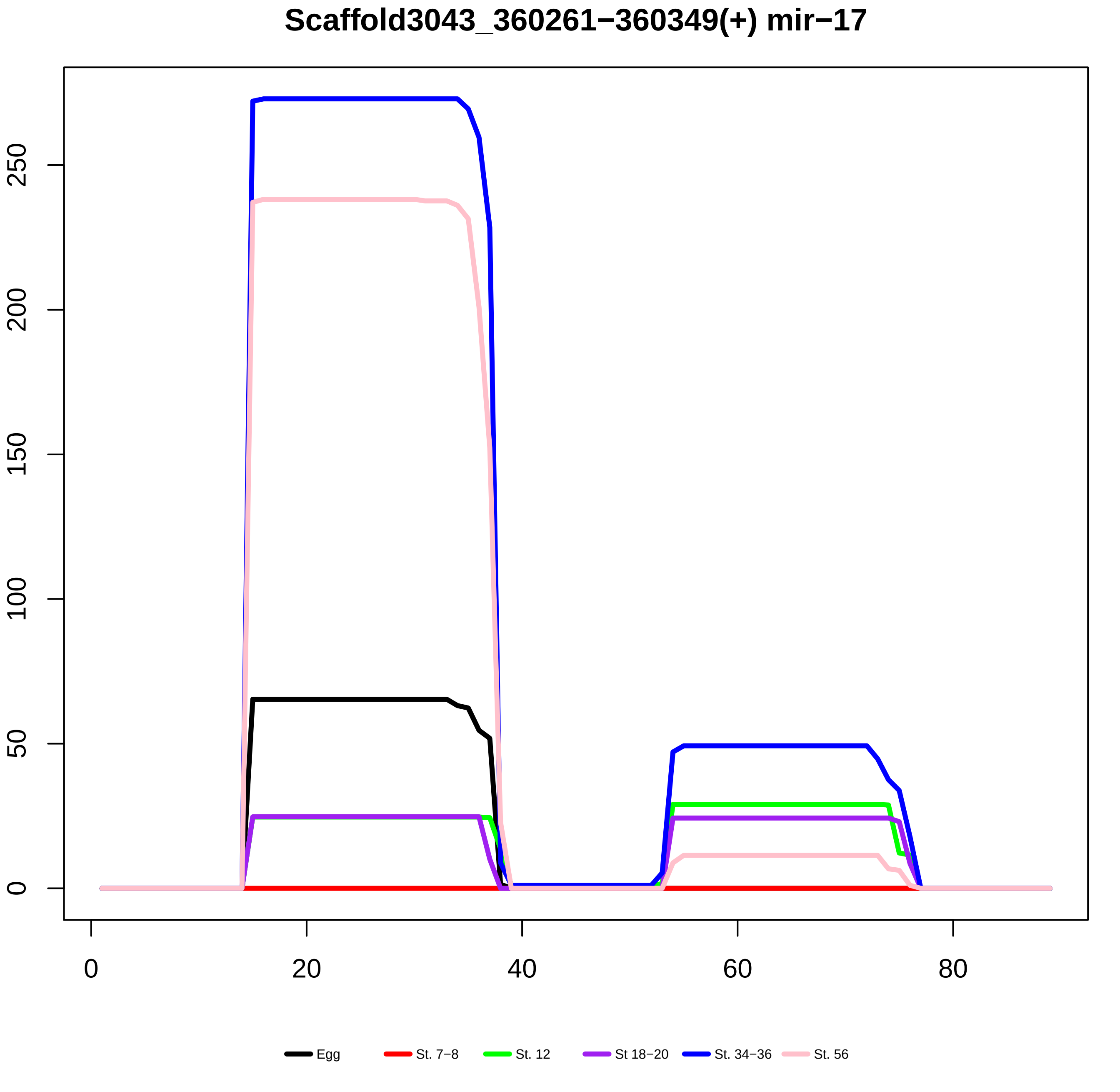
Scaffold3043_360261−360349(+) mir−17
0
50
100
150
200
250
0
20
40
60
80
Egg
St. 7−8
St. 12
St 18−20
St. 34−36
St. 56

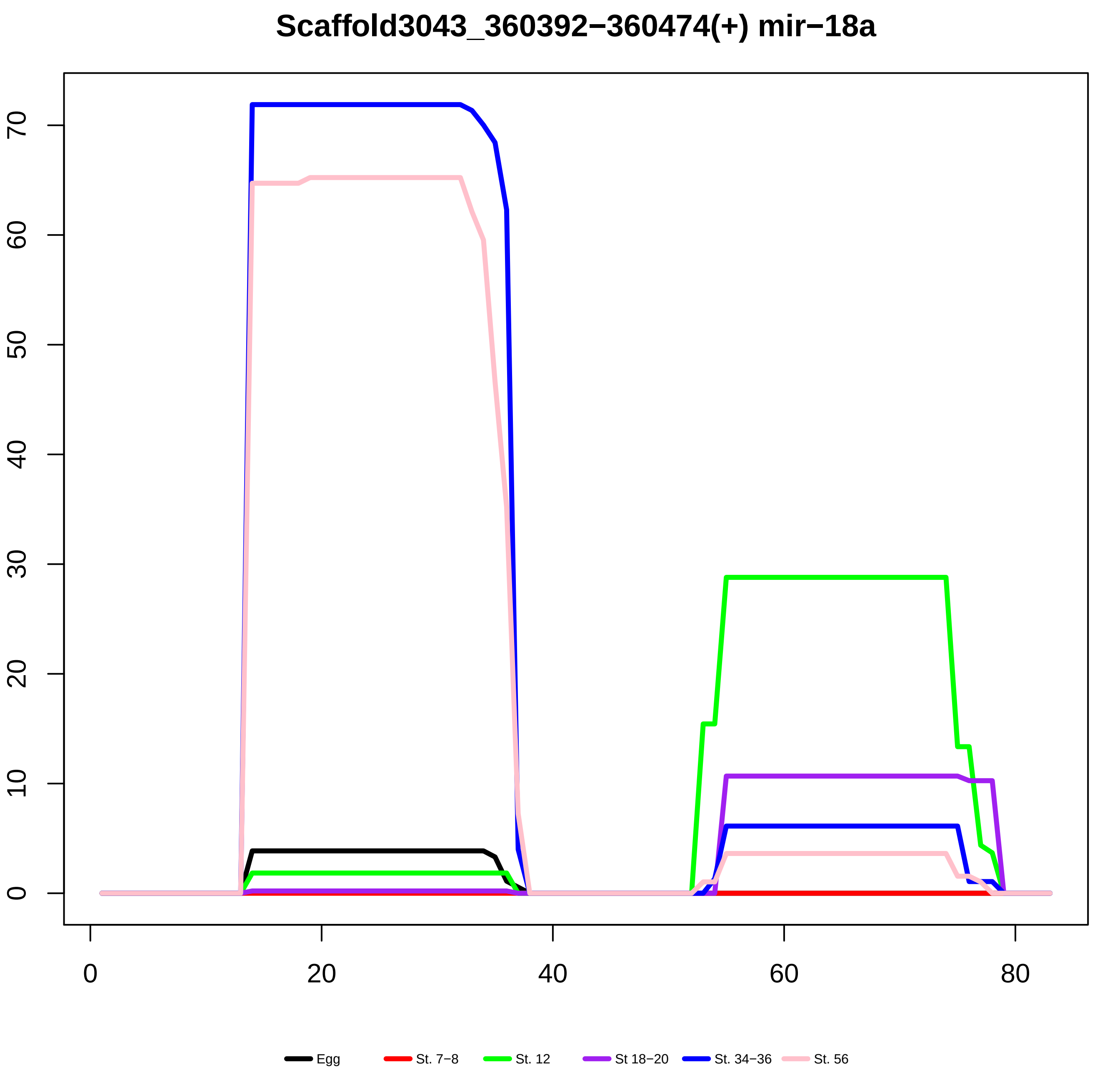

Scaffold3043_360392−360474(+) mir−18a
0
10
20
30
40
50
60
70
0
20
40
60
80
Egg
St. 7−8
St. 12
St 18−20
St. 34−36
St. 56

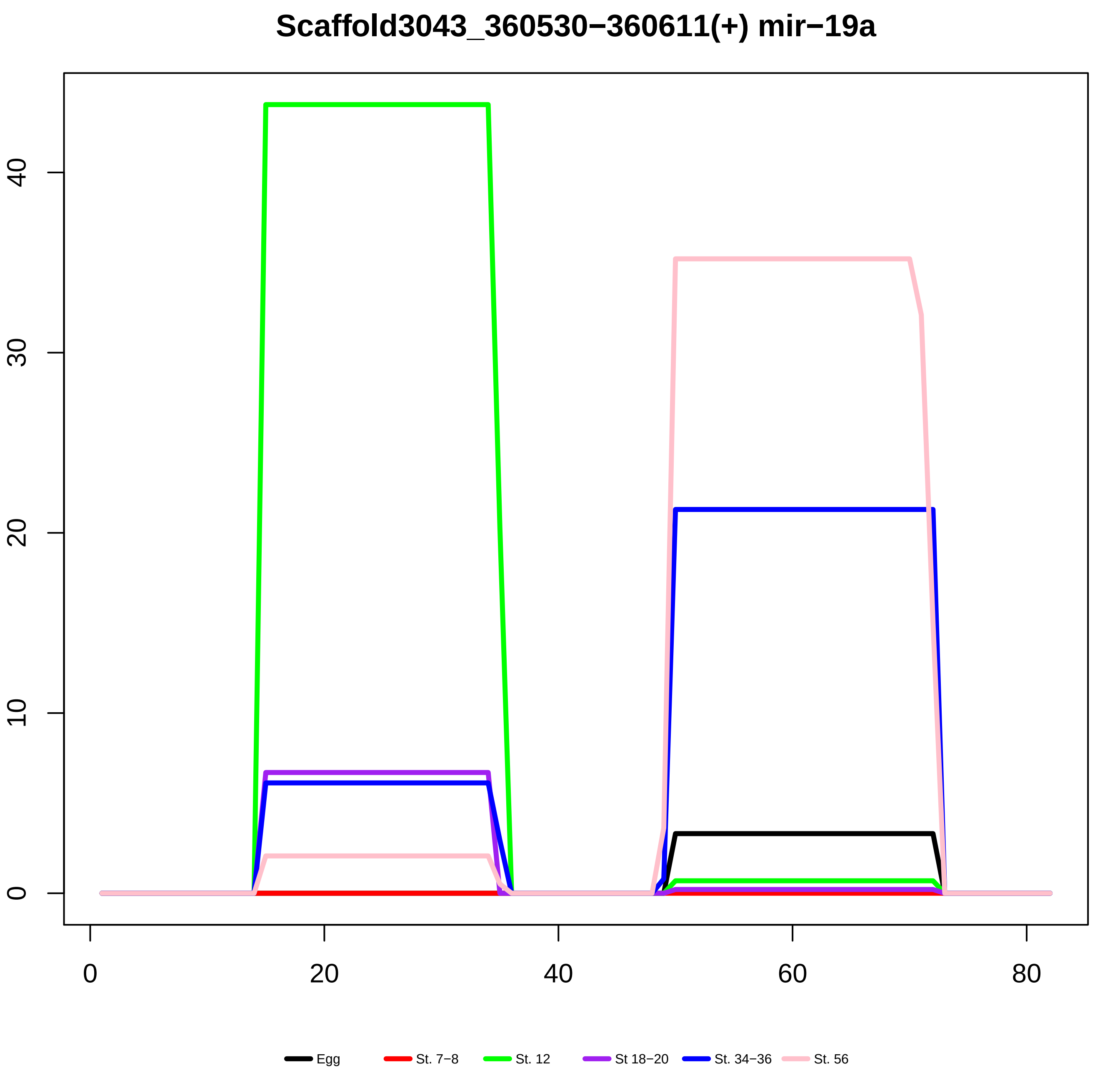

Scaffold3043_360530−360611(+) mir−19a
0
10
20
30
40
0
20
40
60
80
Egg
St. 7−8
St. 12
St 18−20
St. 34−36
St. 56

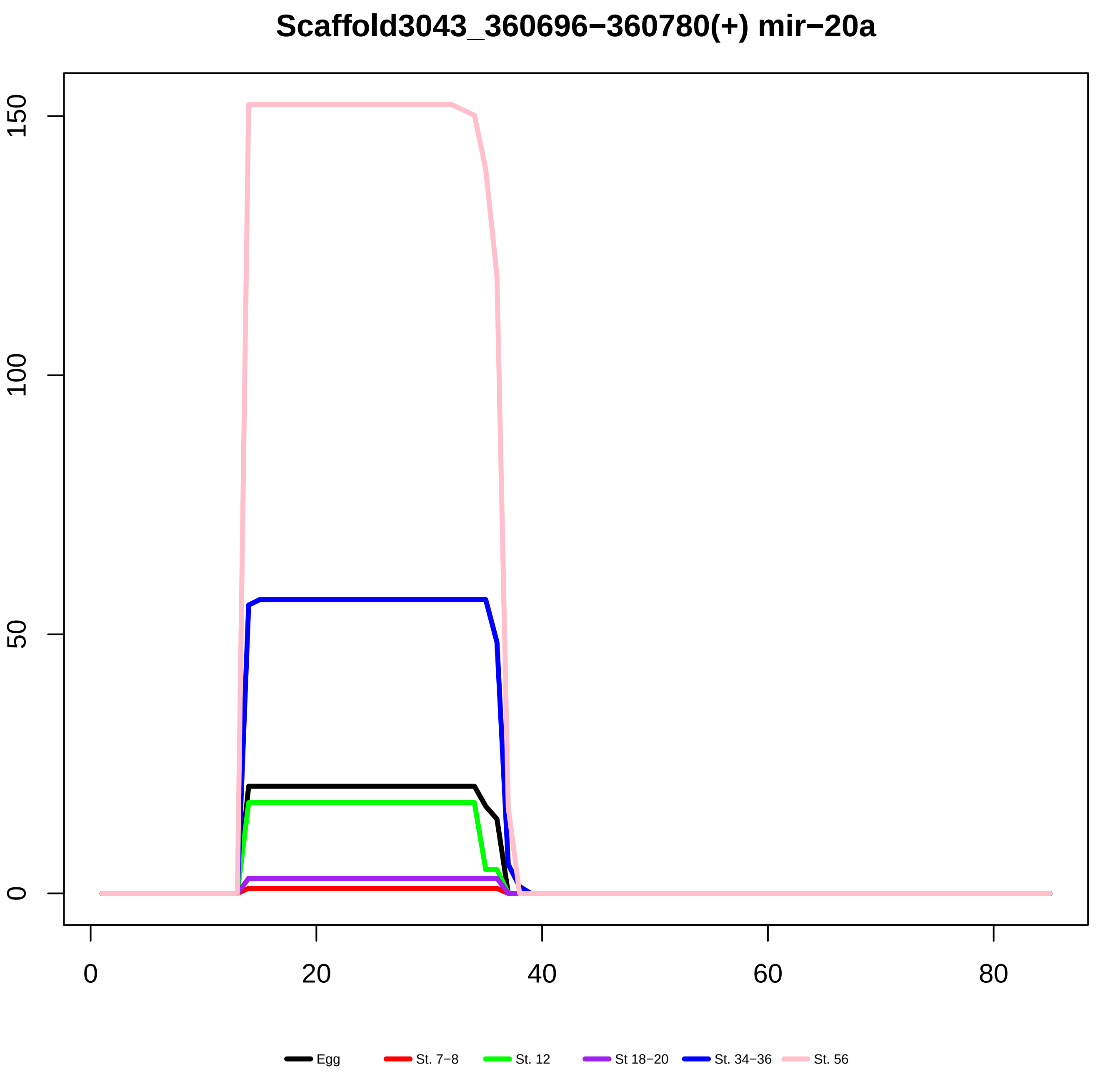
Scaffold3043_360696−360780(+) mir−20a
0
50
100
150
0
20
40
60
80
Egg
St. 7−8
St. 12
St 18−20
St. 34−36
St. 56

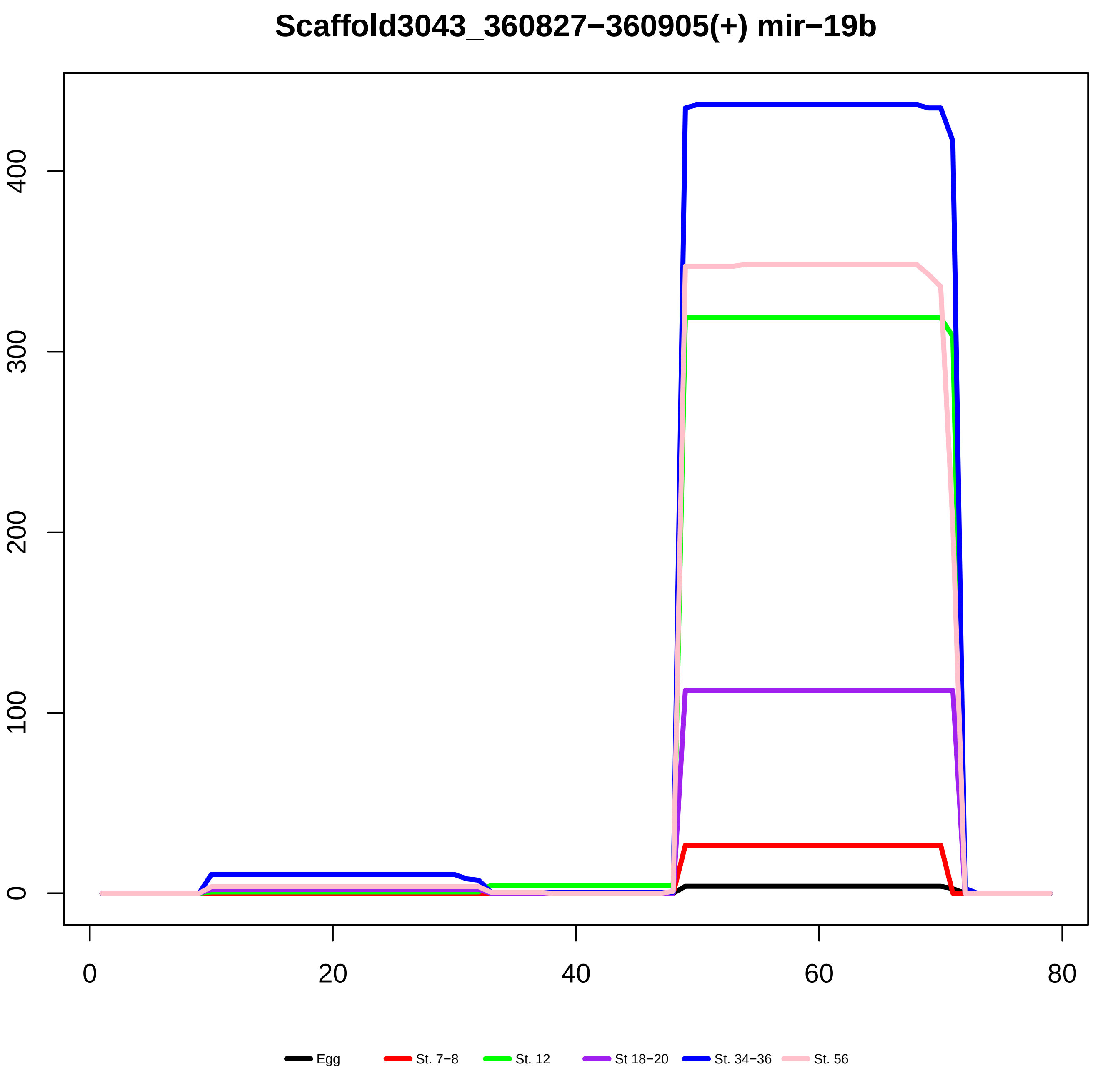
Scaffold3043_360827−360905(+) mir−19b
0
100
200
300
400
0
20
40
60
80
Egg
St. 7−8
St. 12
St 18−20
St. 34−36
St. 56

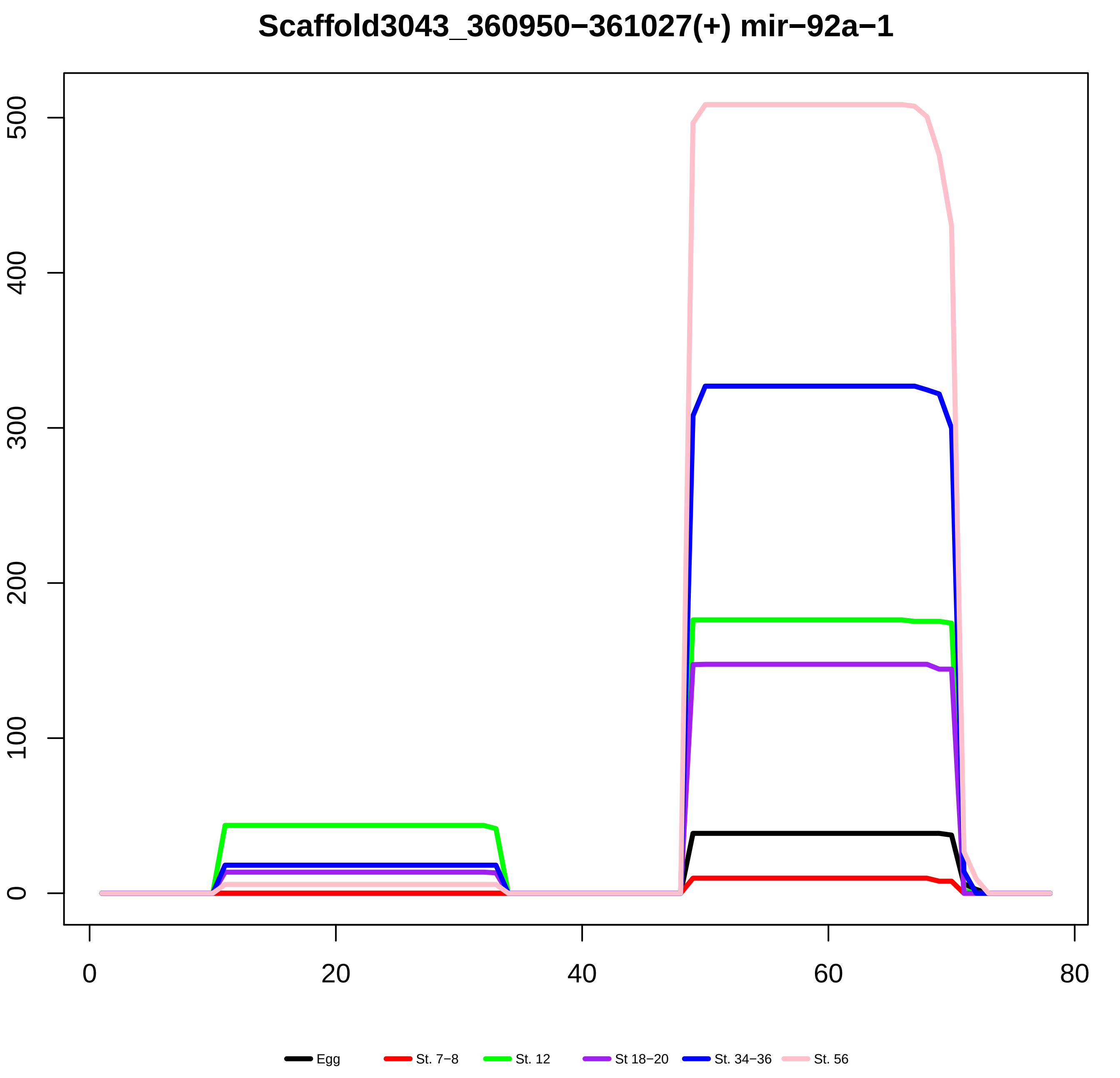
Scaffold3043_360950−361027(+) mir−92a−1
0
100
200
300
400
500
0
20
40
60
80
Egg
St. 7−8
St. 12
St 18−20
St. 34−36
St. 56

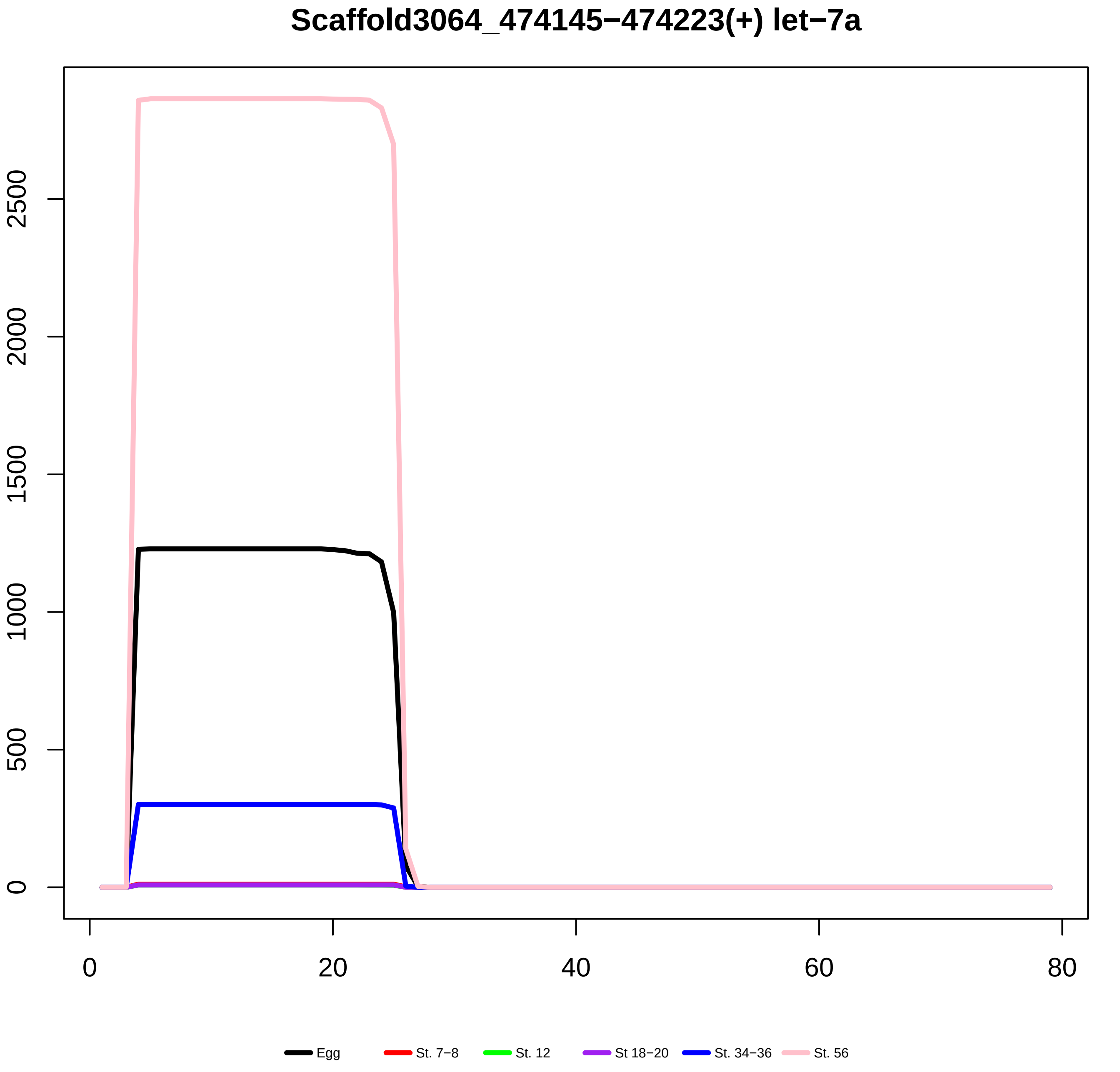
Scaffold3064_474145−474223(+) let−7a
0
500
1000
1500
2000
2500
0
20
40
60
80
Egg
St. 7−8
St. 12
St 18−20
St. 34−36
St. 56

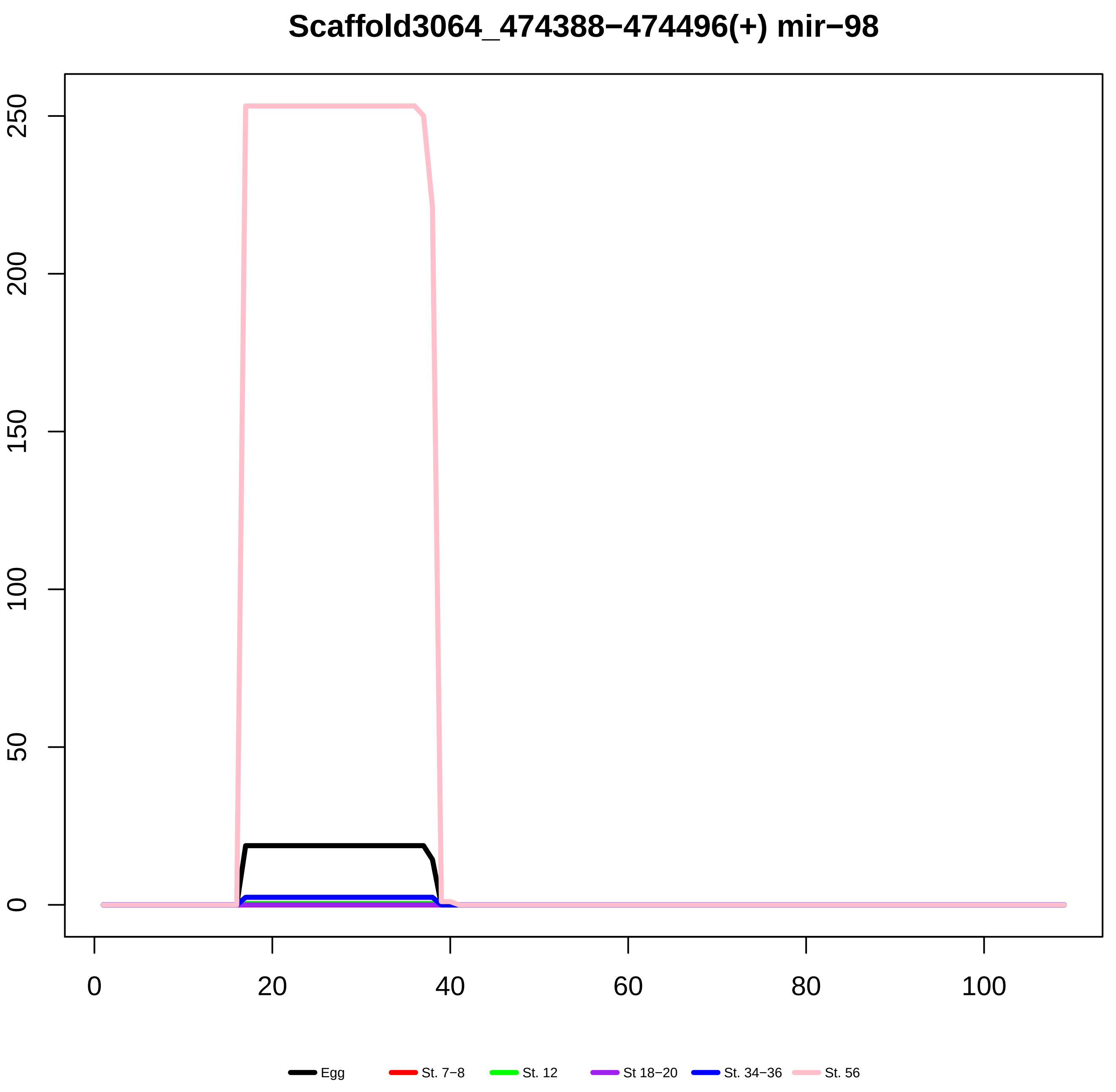

Scaffold3064_474388−474496(+) mir−98
0
50
100
150
200
250
0
20
40
60
80
100
Egg
St. 7−8
St. 12
St 18−20
St. 34−36
St. 56

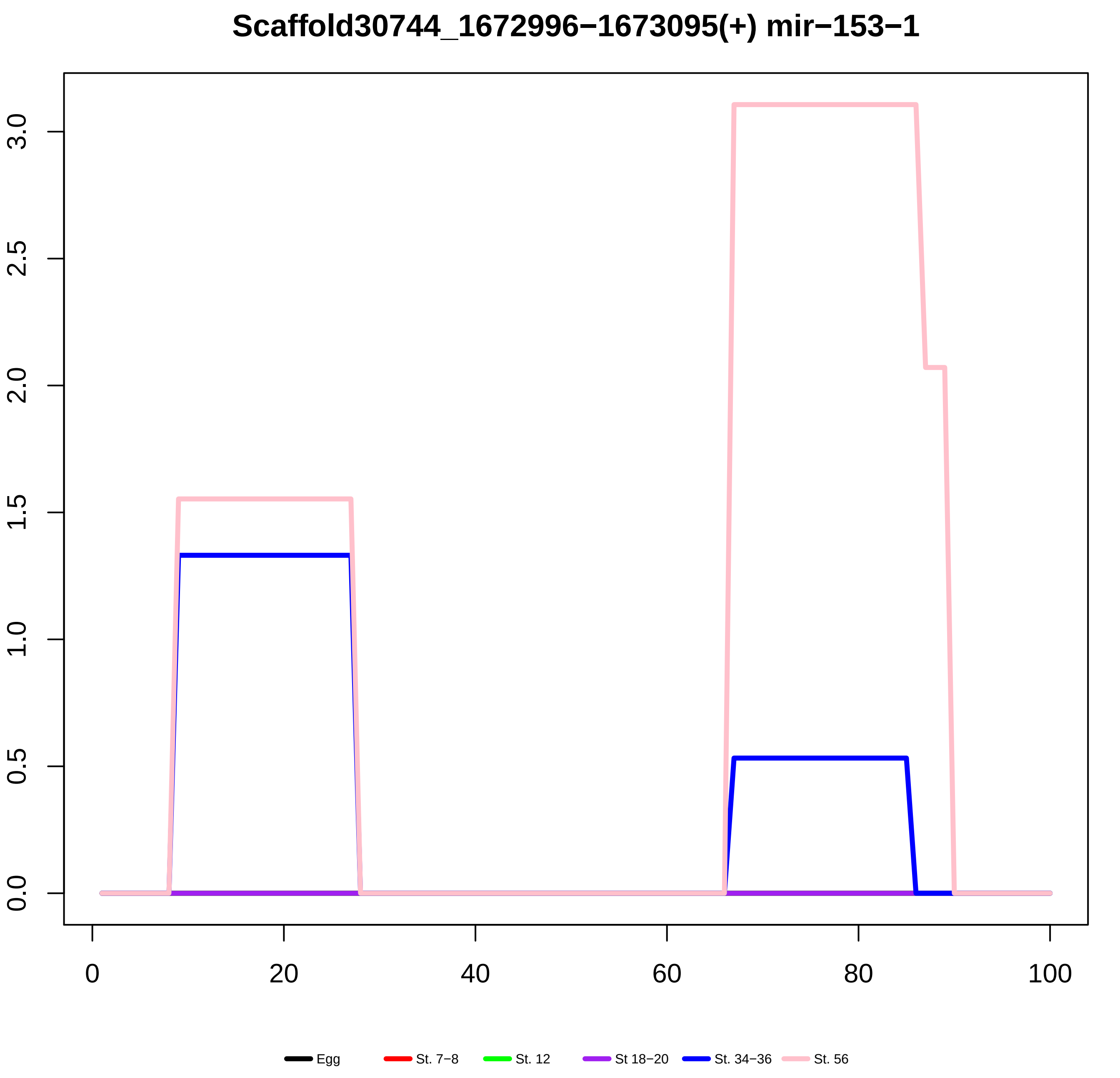

Scaffold30744_1672996−1673095(+) mir−153−1
0.0
0.5
1.0
1.5
2.0
2.5
3.0
0
20
40
60
80
100
Egg
St. 7−8
St. 12
St 18−20
St. 34−36
St. 56

Scaffold30976_647037−647139(−) mir−125b−2

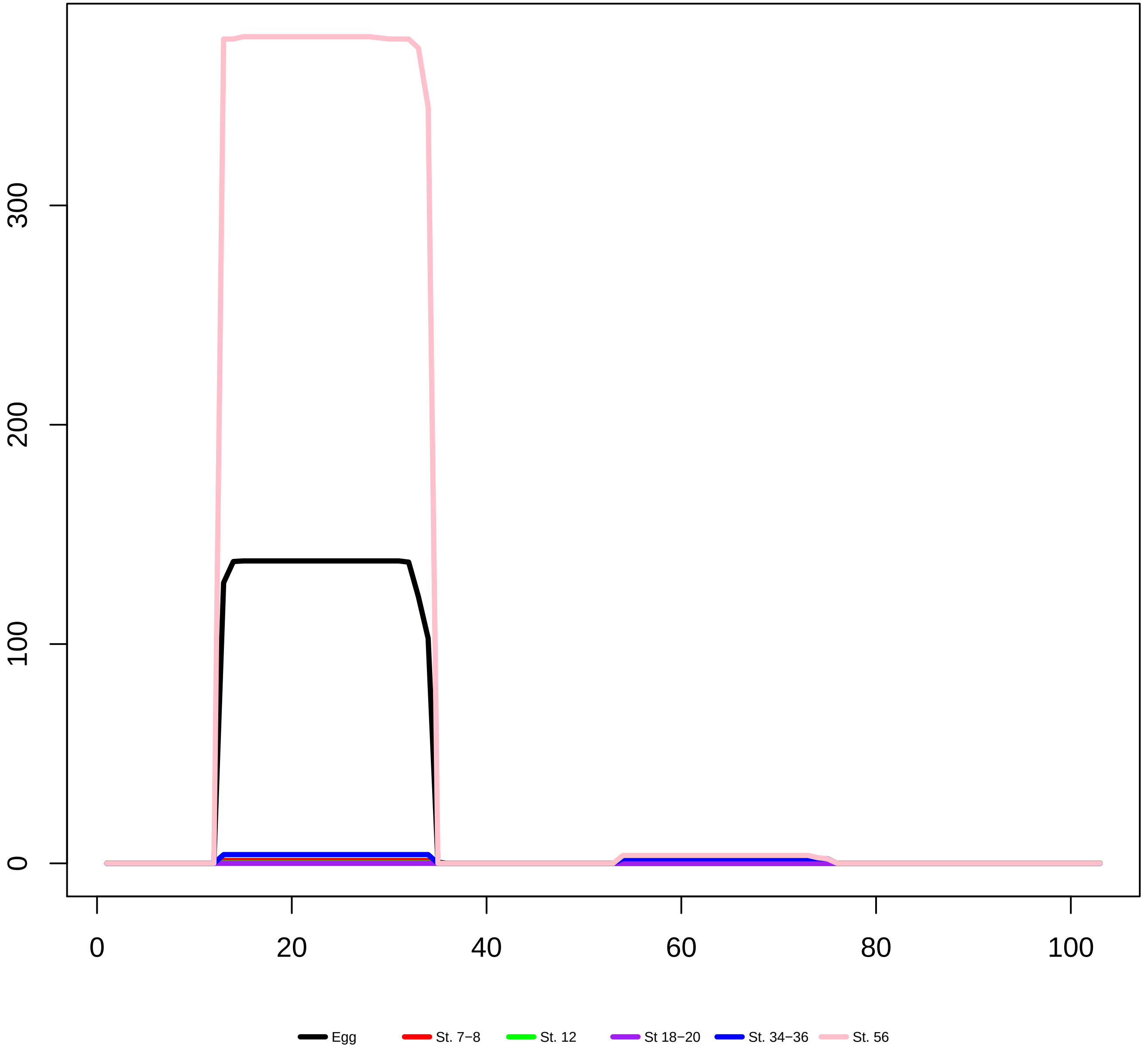

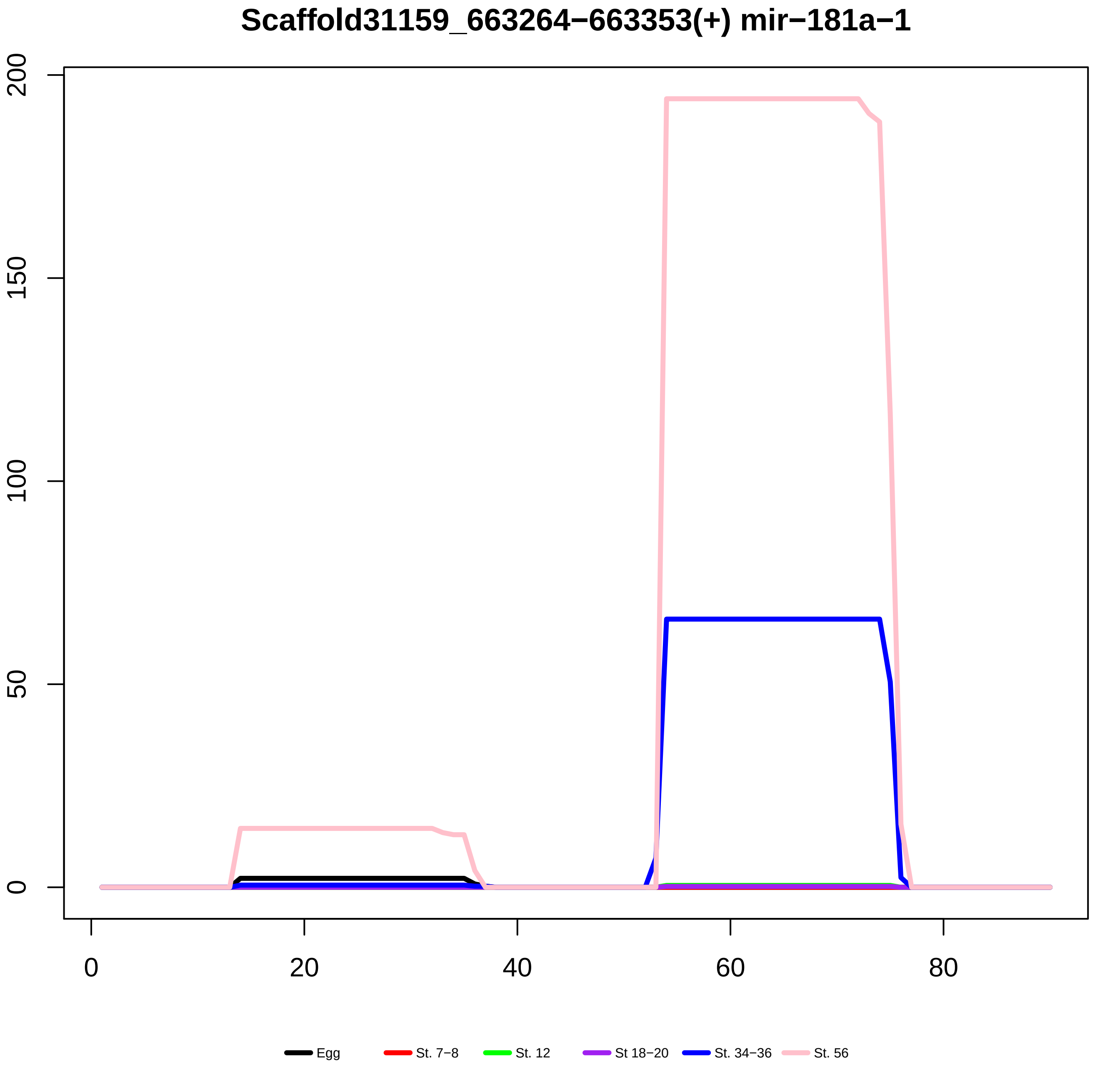
Scaffold31159_663264−663353(+) mir−181a−1
0
50
100
150
200
0
20
40
60
80
Egg
St. 7−8
St. 12
St 18−20
St. 34−36
St. 56

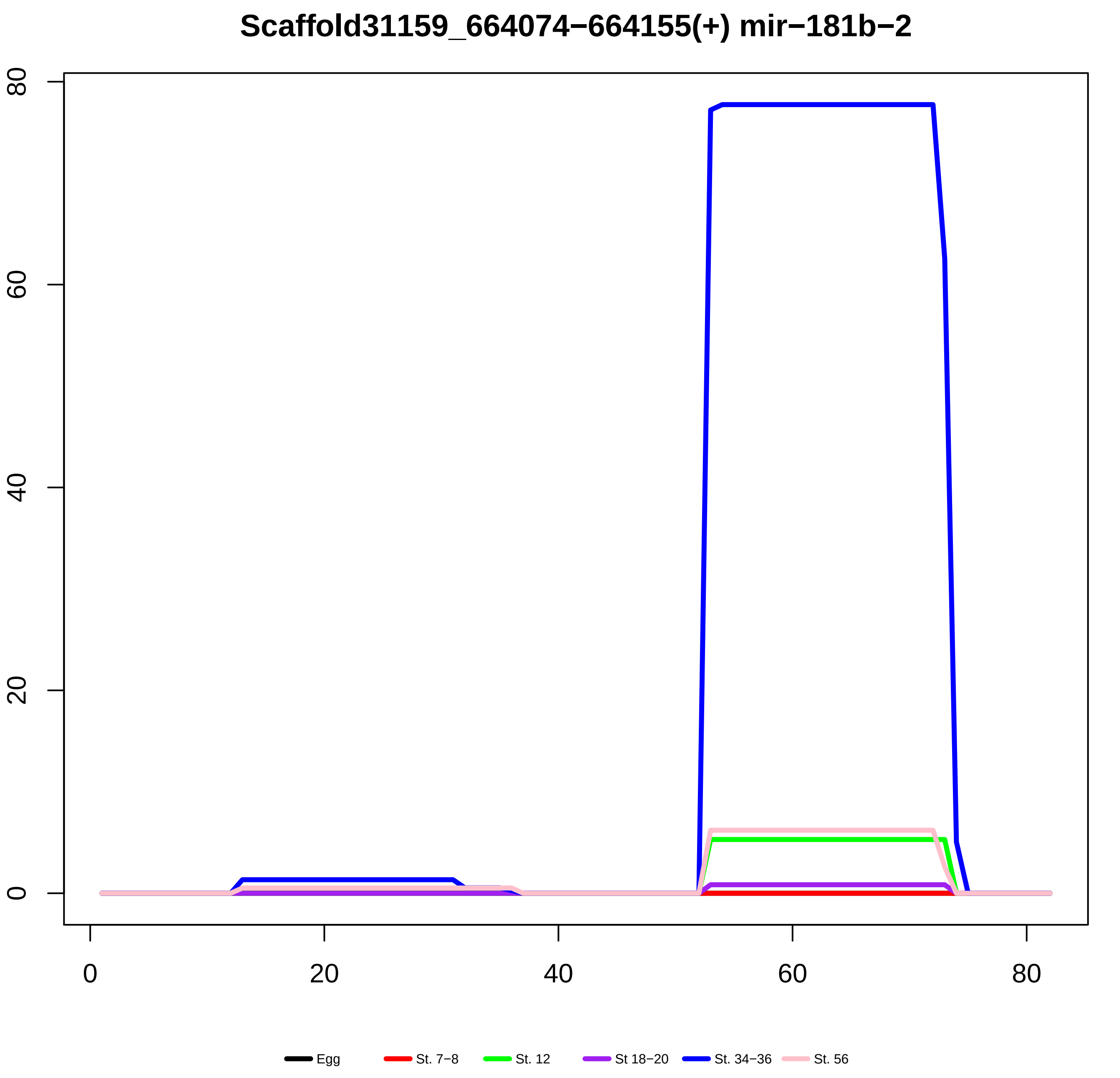

Scaffold31159_664074−664155(+) mir−181b−2
0
20
40
60
80
0
20
40
60
80
Egg
St. 7−8
St. 12
St 18−20
St. 34−36
St. 56

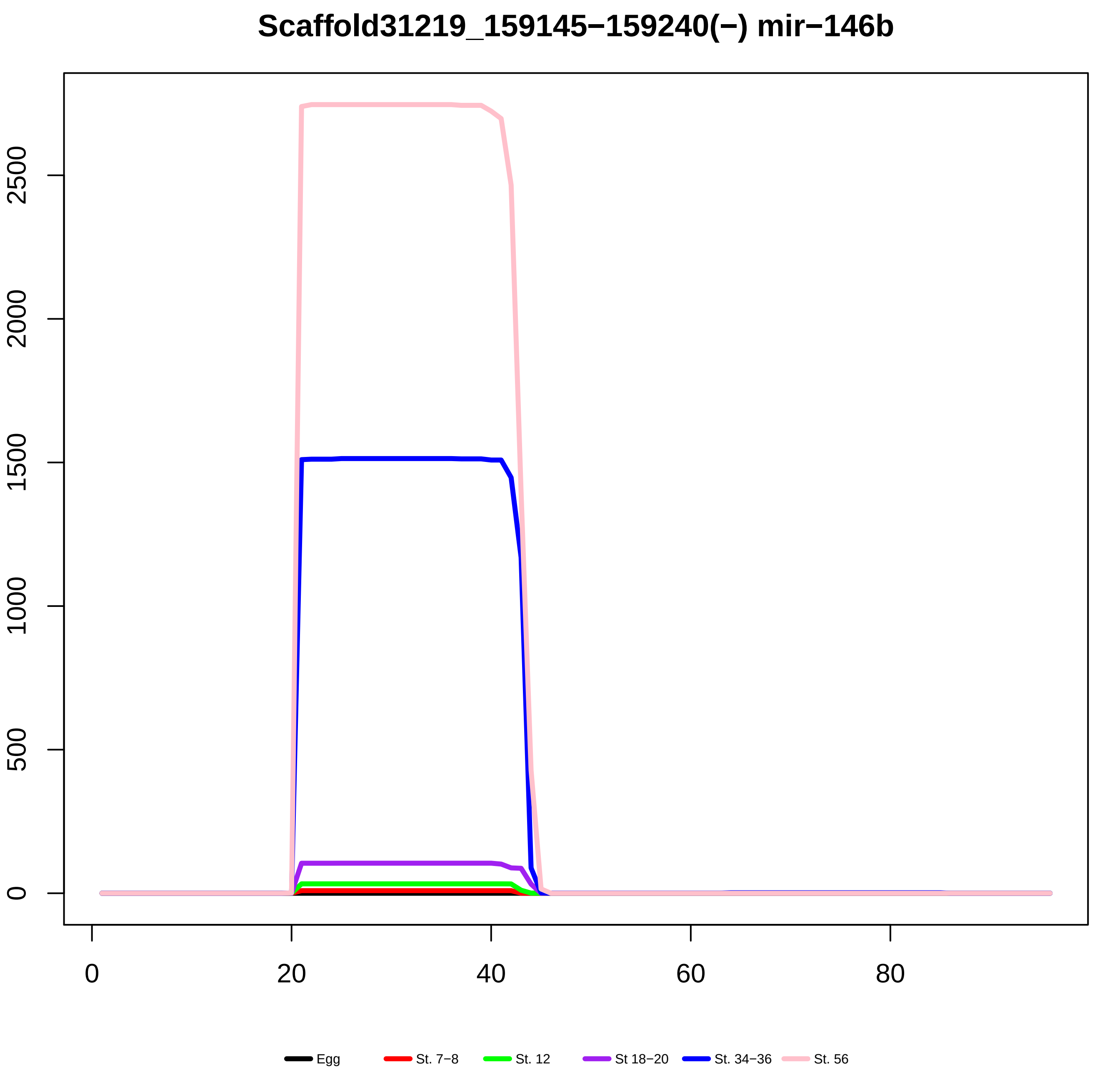

Scaffold31219_159145−159240(−) mir−146b
0
500
1000
1500
2000
2500
0
20
40
60
80
Egg
St. 7−8
St. 12
St 18−20
St. 34−36
St. 56

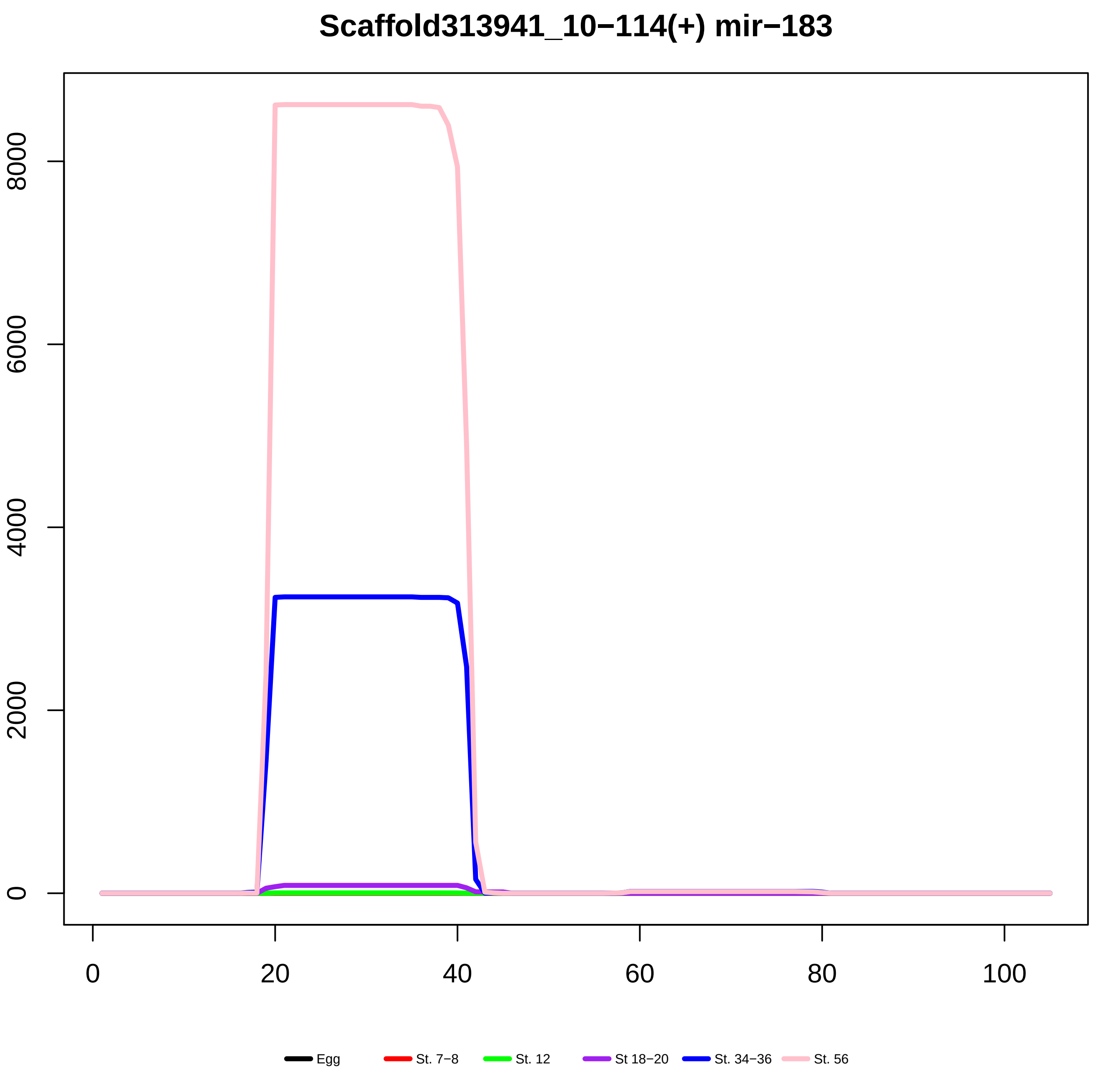

Scaffold313941_10−114(+) mir−183
0
2000
4000
6000
8000
0
20
40
60
80
100
Egg
St. 7−8
St. 12
St 18−20
St. 34−36
St. 56

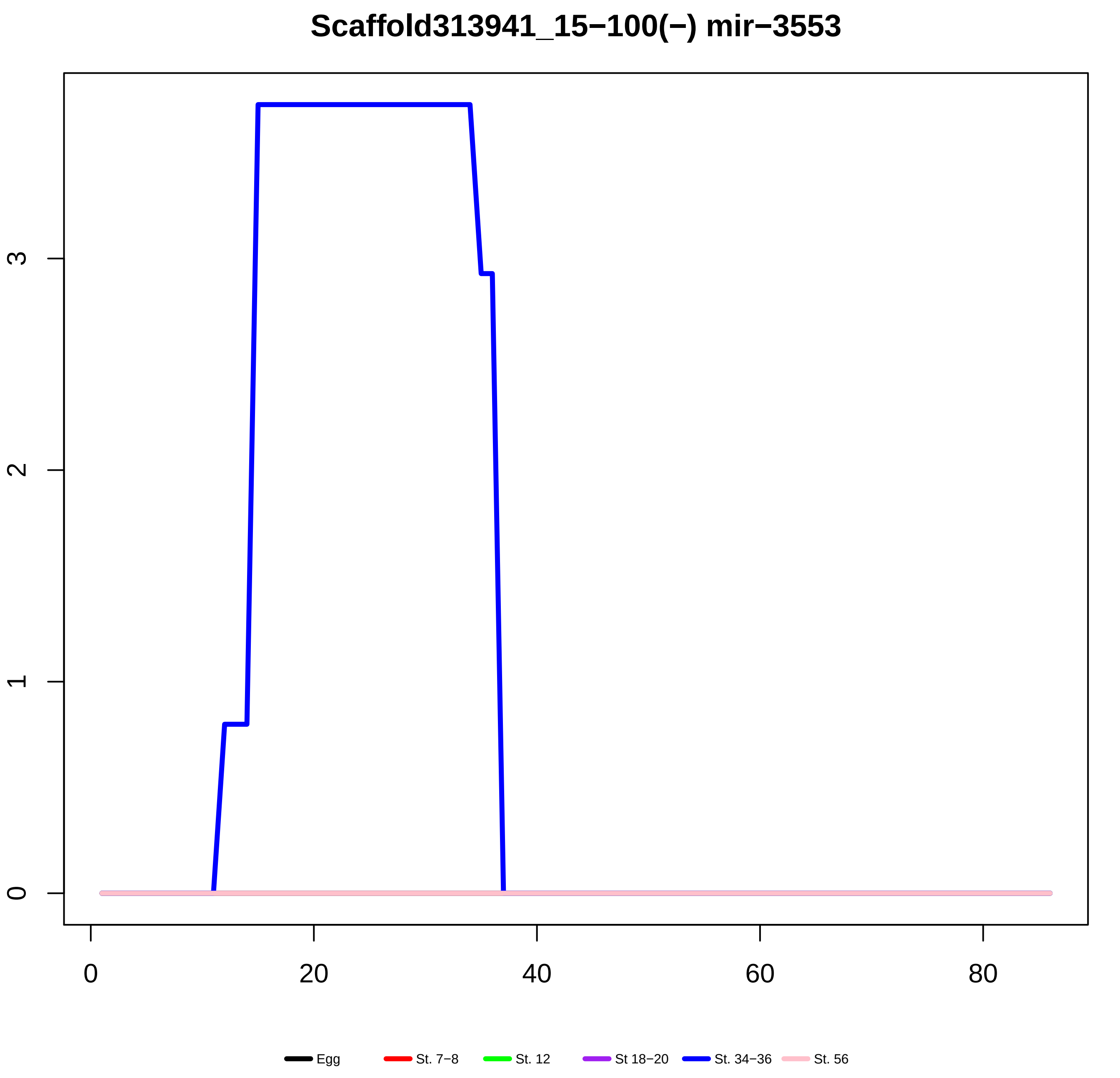
Scaffold313941_15−100(−) mir−3553
0
1
2
3
0
20
40
60
80
Egg
St. 7−8
St. 12
St 18−20
St. 34−36
St. 56

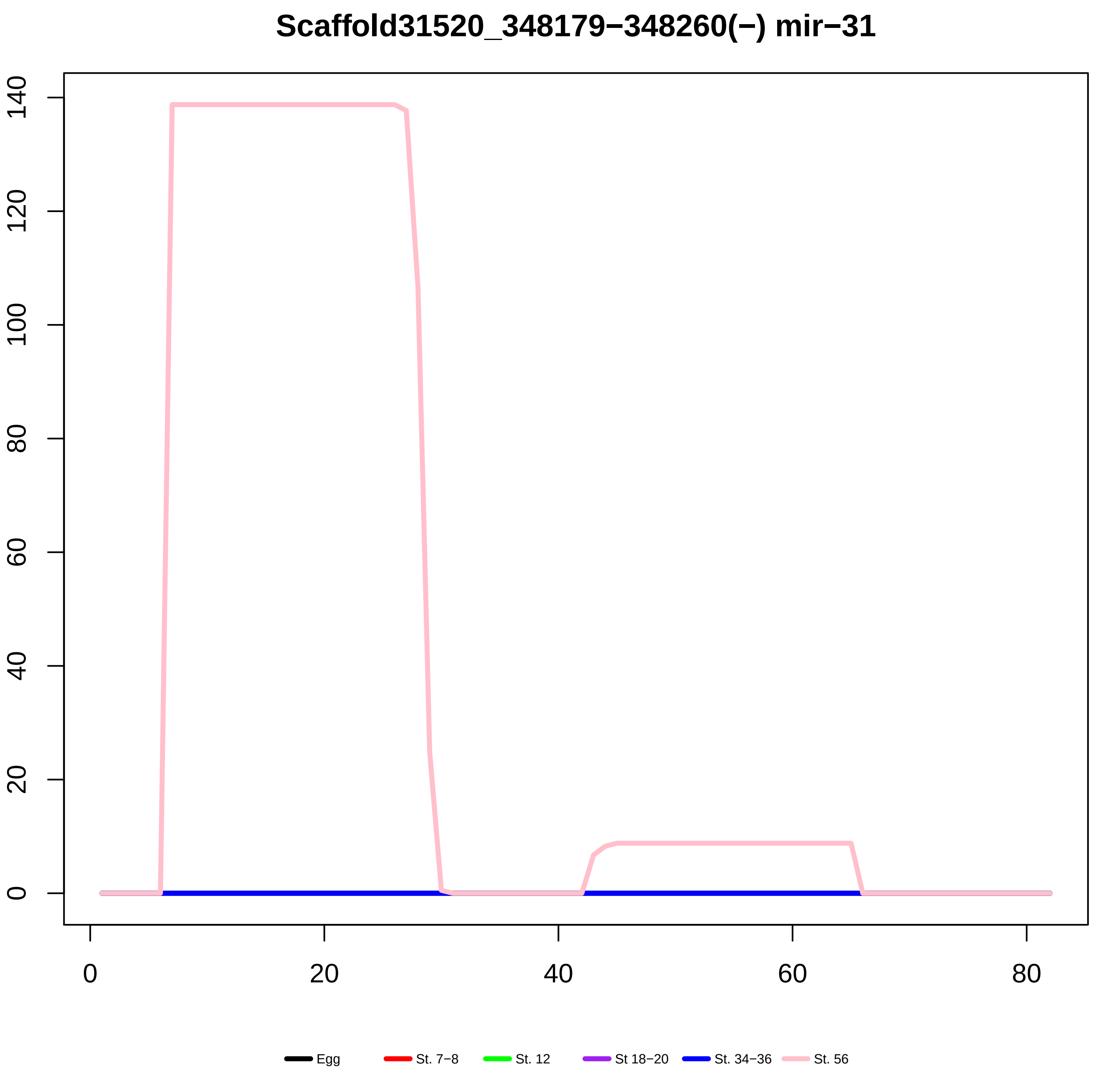
Scaffold31520_348179−348260(−) mir−31
0
20
40
60
80
100
120
140
0
20
40
60
80
Egg
St. 7−8
St. 12
St 18−20
St. 34−36
St. 56

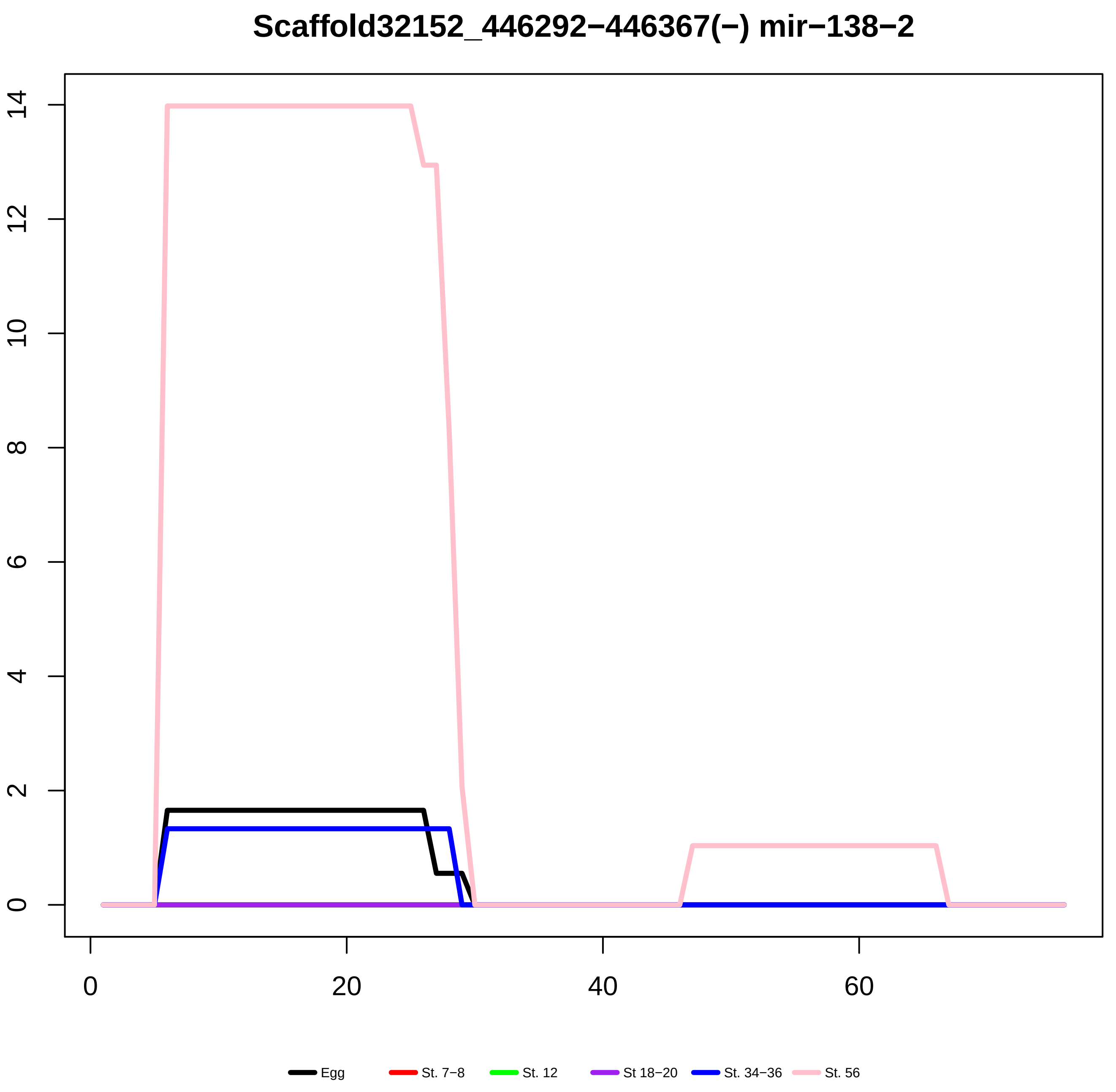
Scaffold32152_446292−446367(−) mir−138−2
0
2
4
6
8
10
12
14
0
20
40
60
Egg
St. 7−8
St. 12
St 18−20
St. 34−36
St. 56

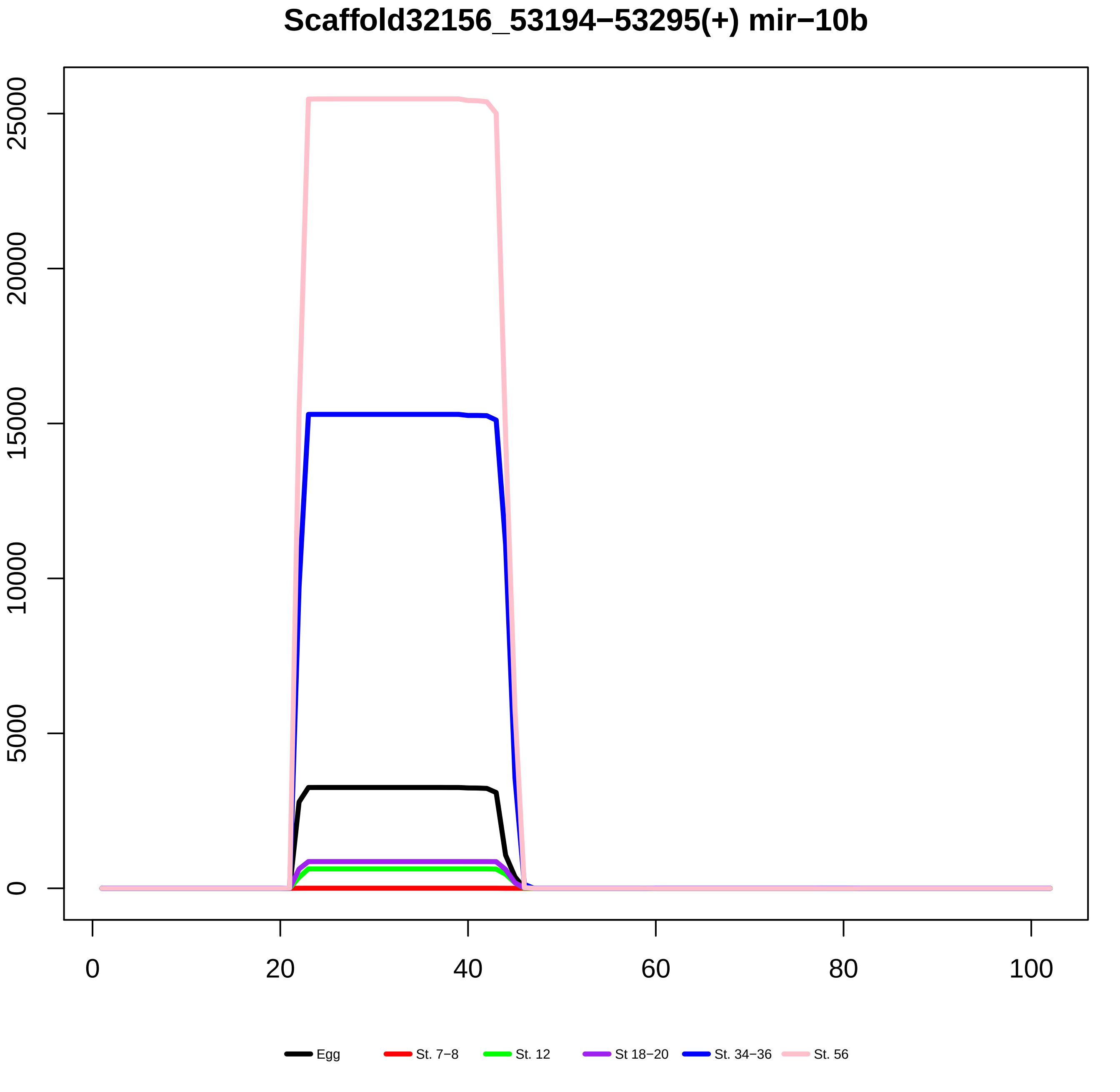

Scaffold32156_53194−53295(+) mir−10b
0
5000
10000
15000
20000
25000
0
20
40
60
80
100
Egg
St. 7−8
St. 12
St 18−20
St. 34−36
St. 56

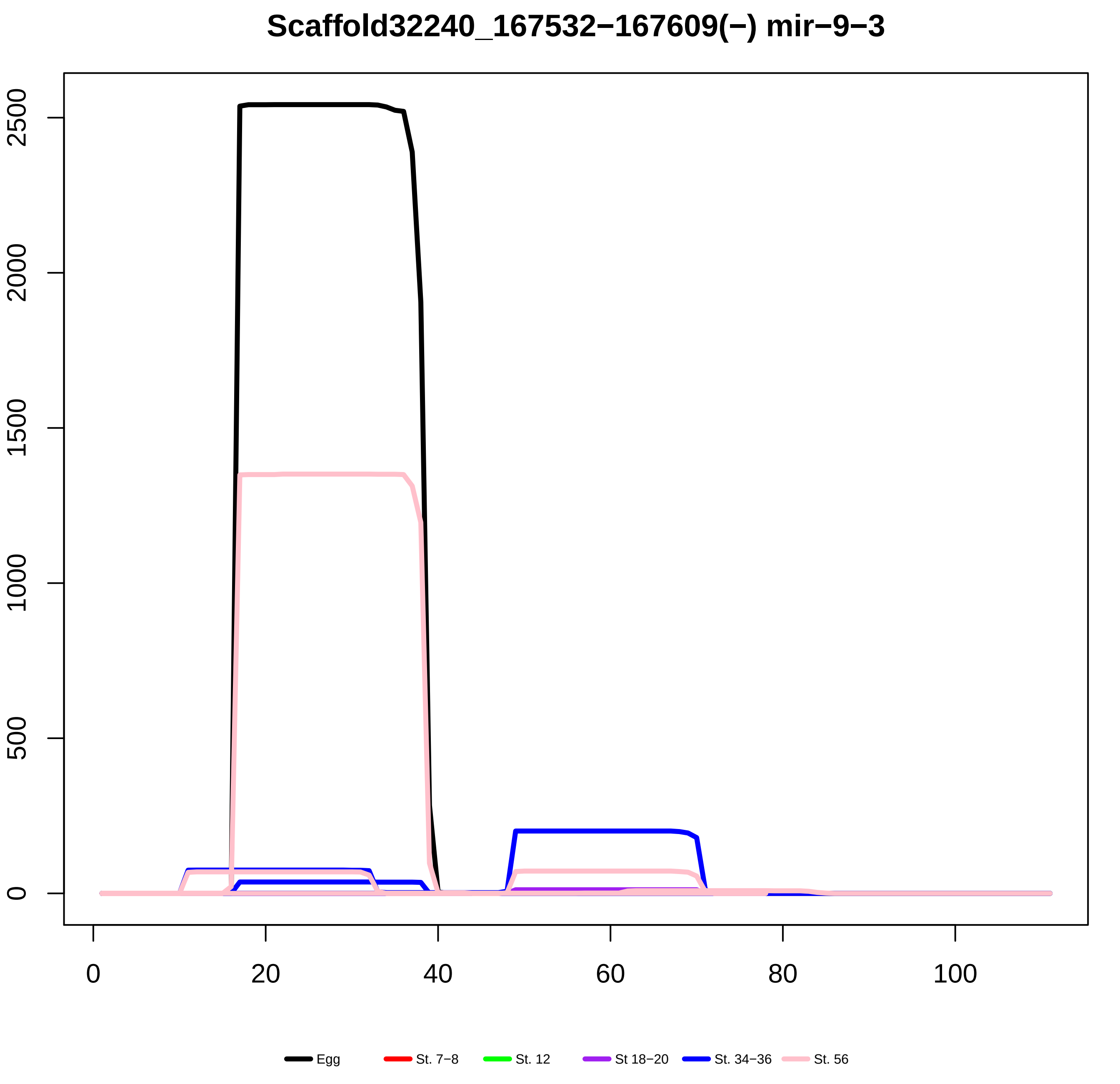

Scaffold32240_167532−167609(−) mir−9−3
0
500
1000
1500
2000
2500
0
20
40
60
80
100
Egg
St. 7−8
St. 12
St 18−20
St. 34−36
St. 56

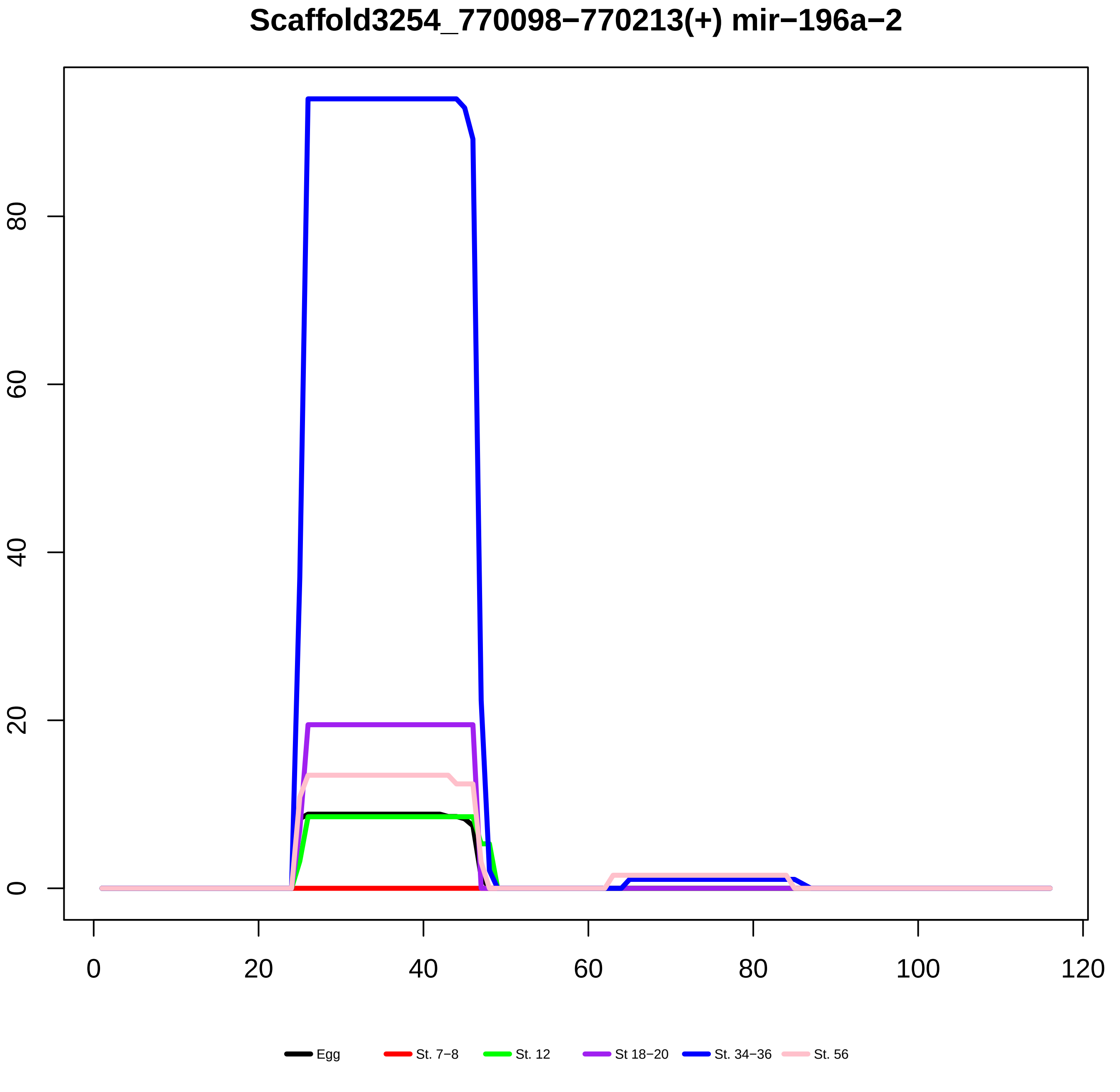

Scaffold3254_770098−770213(+) mir−196a−2
0
20
40
60
80
0
20
40
60
80
100
120
Egg
St. 7−8
St. 12
St 18−20
St. 34−36
St. 56

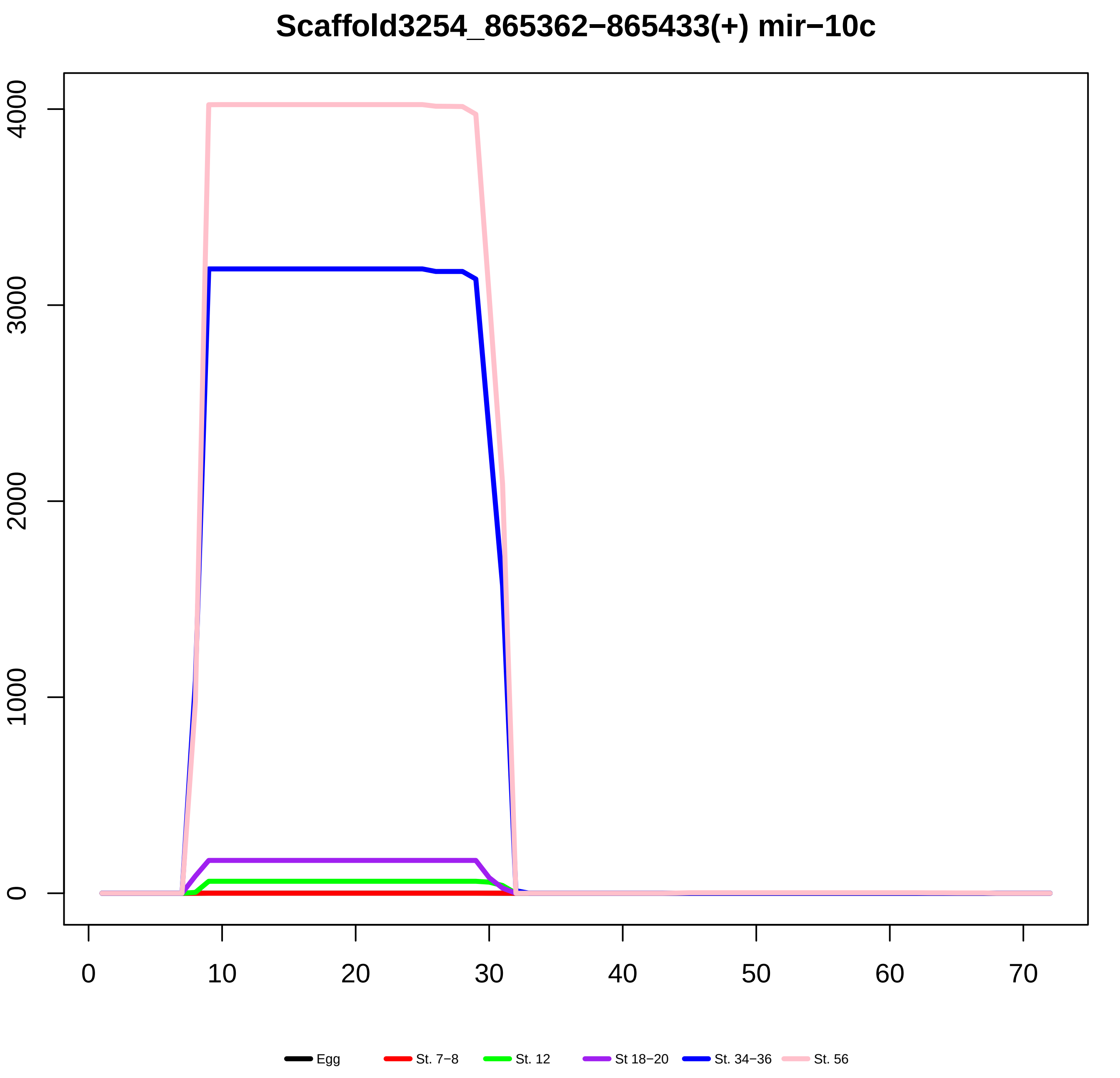
Scaffold3254_865362−865433(+) mir−10c
0
1000
2000
3000
4000
0
10
20
30
40
50
60
70
Egg
St. 7−8
St. 12
St 18−20
St. 34−36
St. 56

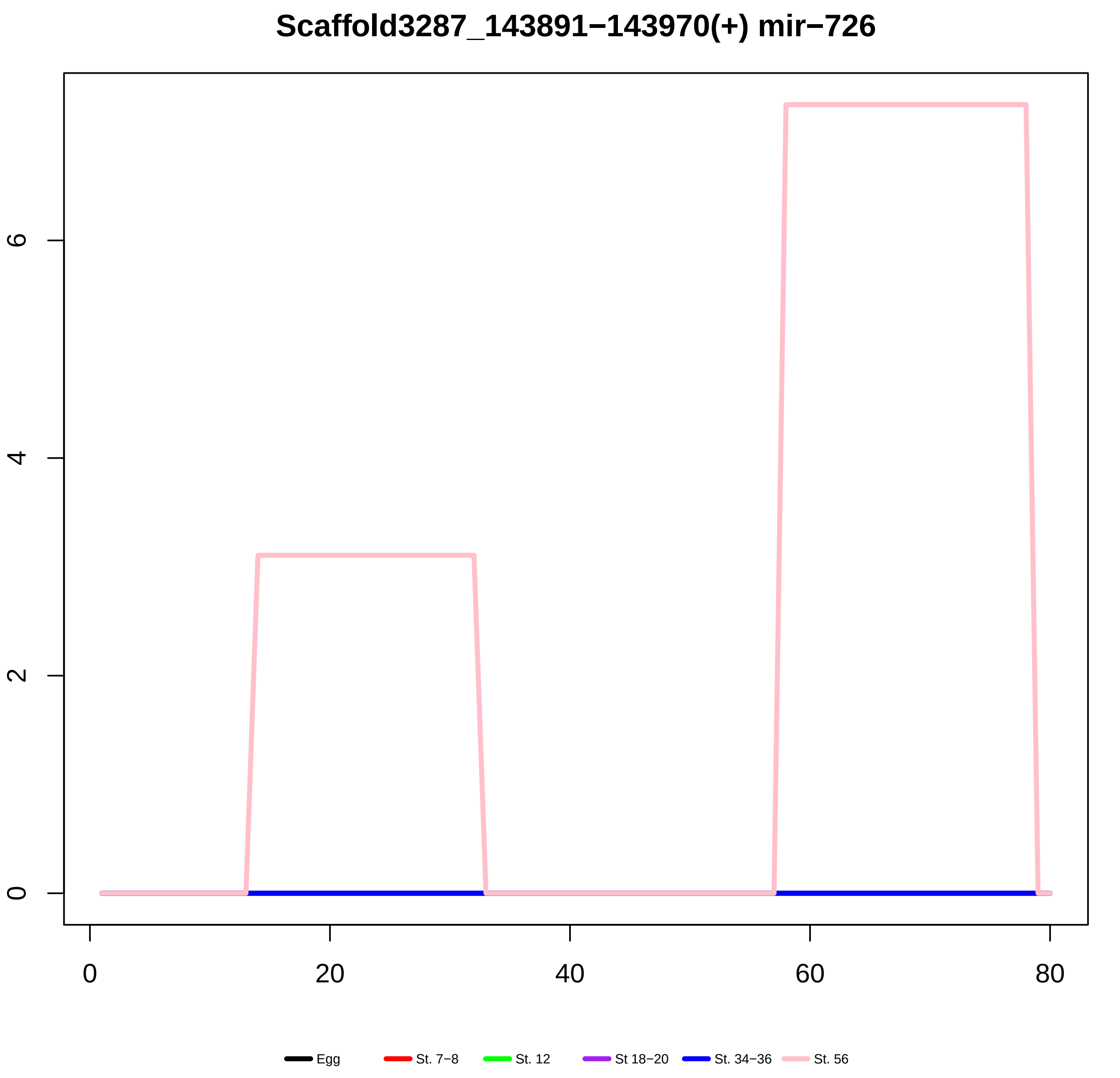

Scaffold3287_143891−143970(+) mir−726
0
2
4
6
0
20
40
60
80
Egg
St. 7−8
St. 12
St 18−20
St. 34−36
St. 56

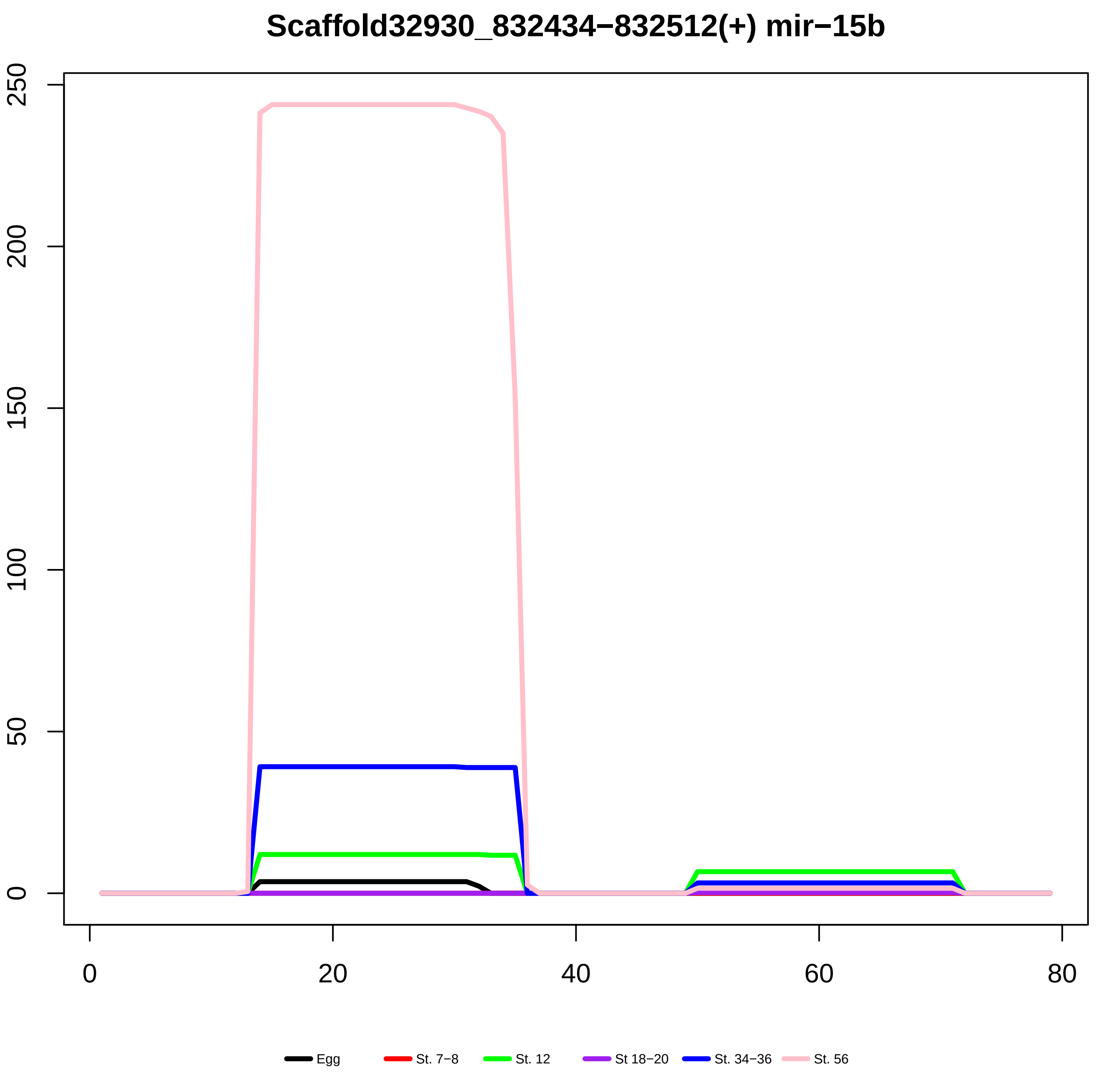

Scaffold32930_832434−832512(+) mir−15b
0
50
100
150
200
250
0
20
40
60
80
Egg
St. 7−8
St. 12
St 18−20
St. 34−36
St. 56

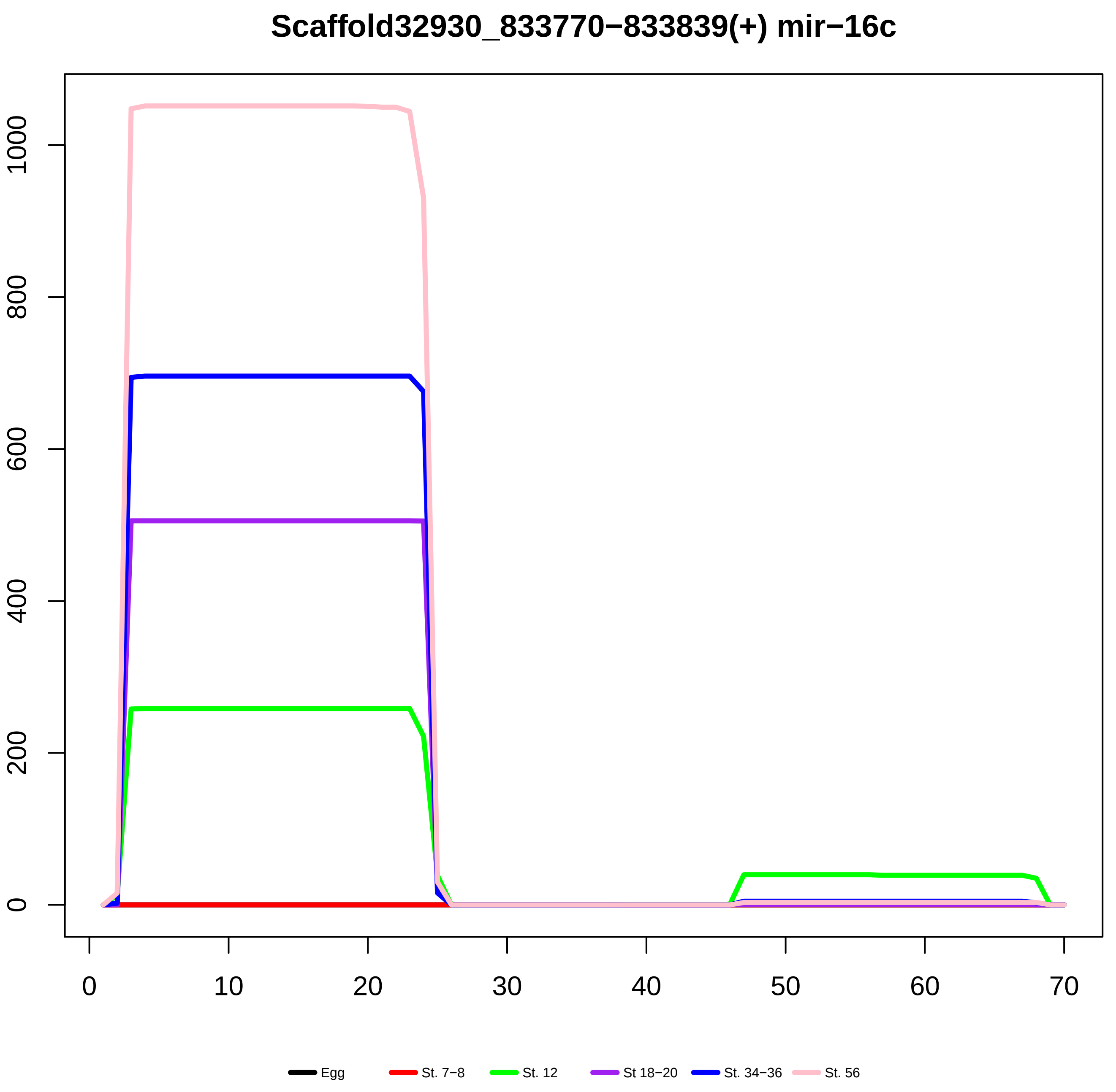

Scaffold32930_833770−833839(+) mir−16c
0
200
400
600
800
1000
0
10
20
30
40
50
60
70
Egg
St. 7−8
St. 12
St 18−20
St. 34−36
St. 56

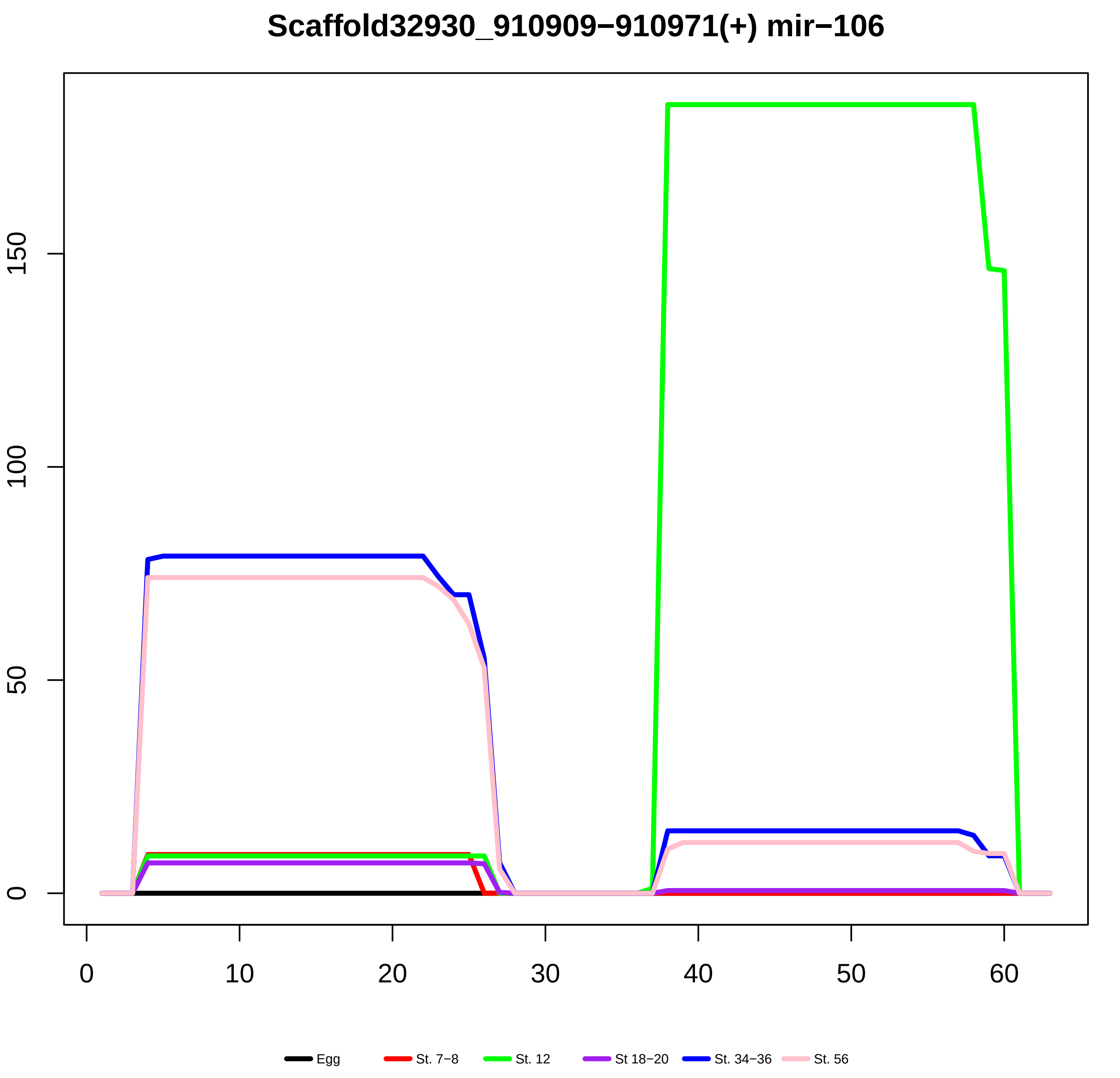

Scaffold32930_910909−910971(+) mir−106
0
50
100
150
0
10
20
30
40
50
60
Egg
St. 7−8
St. 12
St 18−20
St. 34−36
St. 56

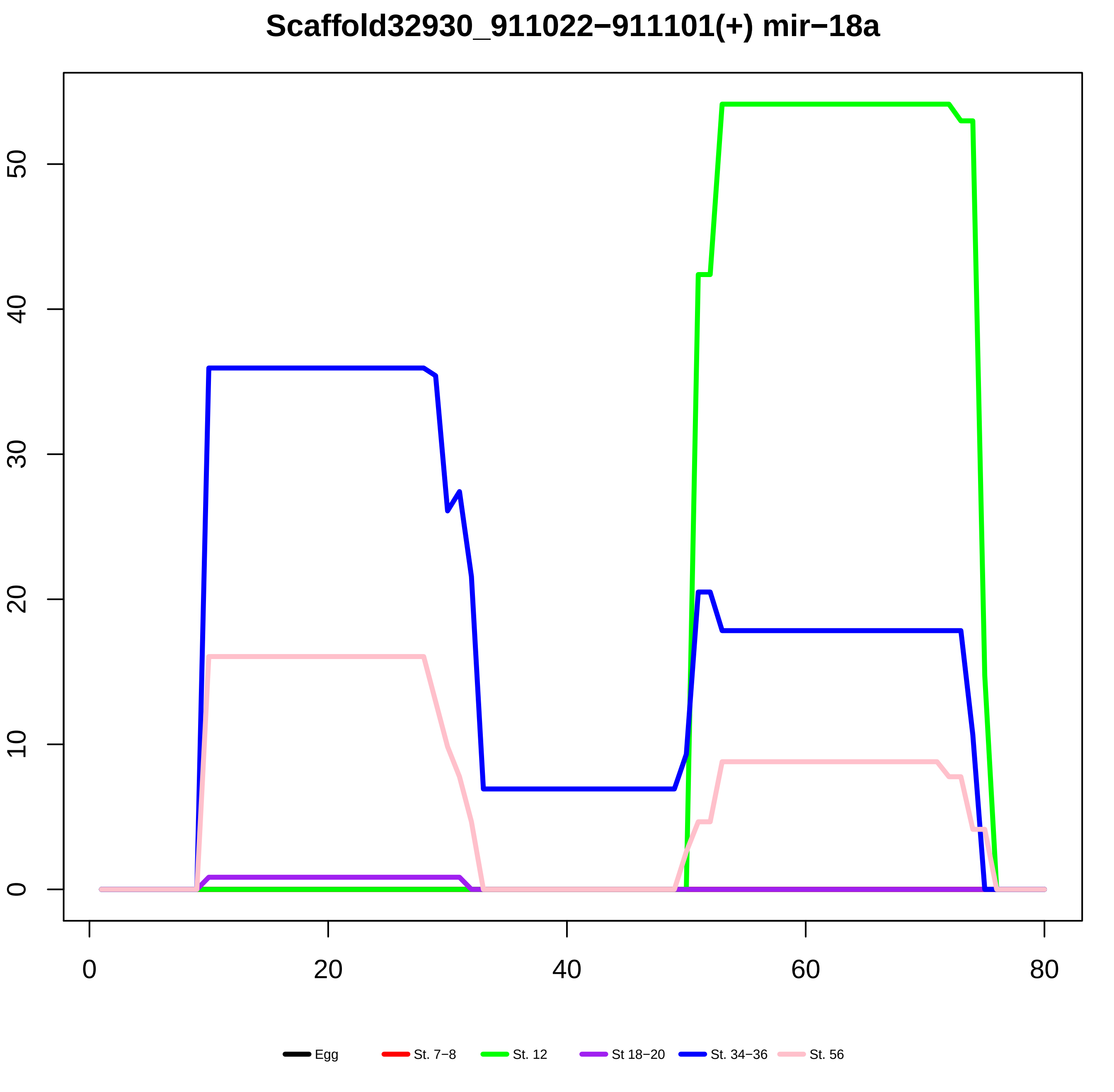

Scaffold32930_911022−911101(+) mir−18a
0
10
20
30
40
50
0
20
40
60
80
Egg
St. 7−8
St. 12
St 18−20
St. 34−36
St. 56

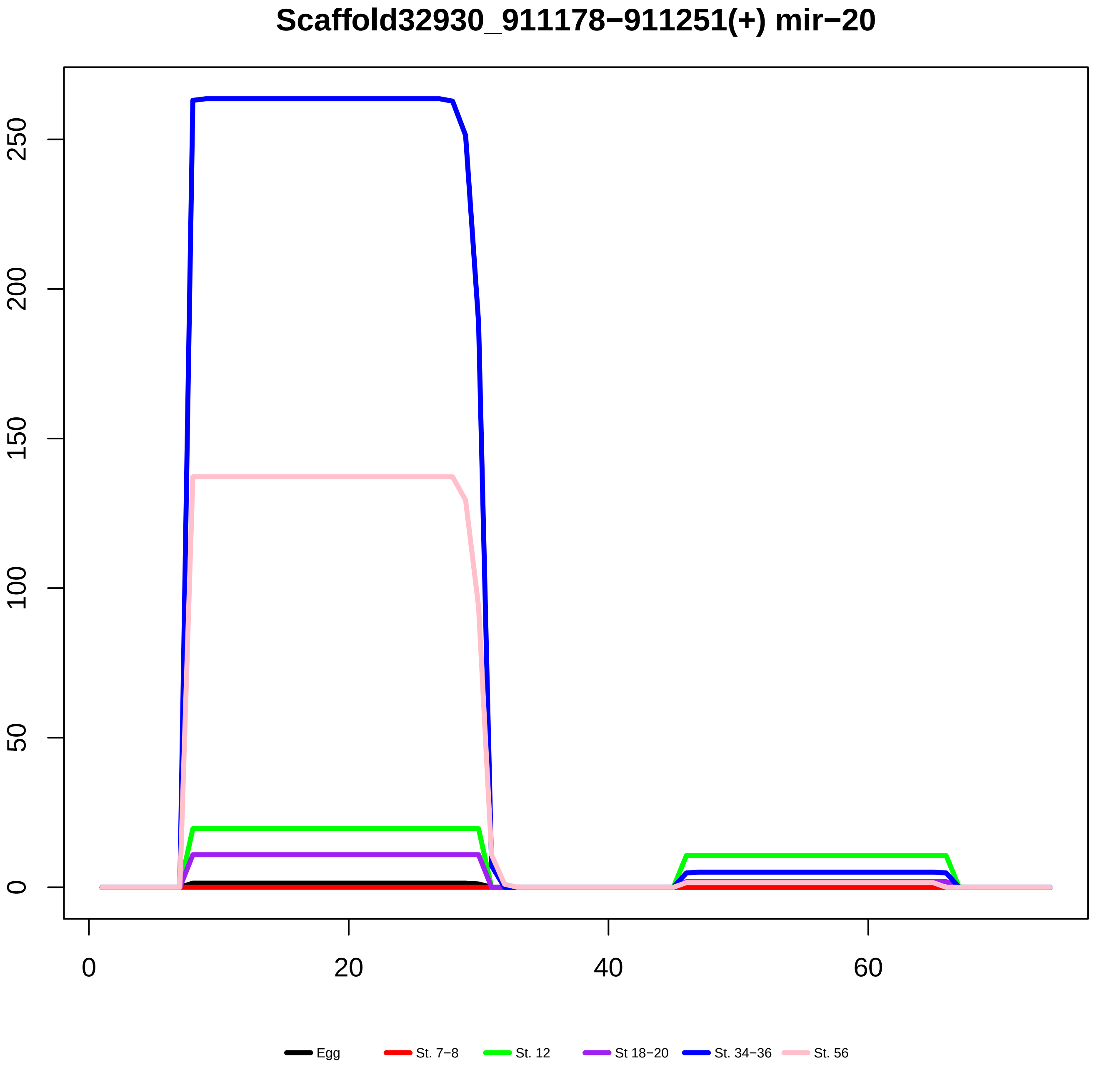

Scaffold32930_911178−911251(+) mir−20
0
50
100
150
200
250
0
20
40
60
Egg
St. 7−8
St. 12
St 18−20
St. 34−36
St. 56

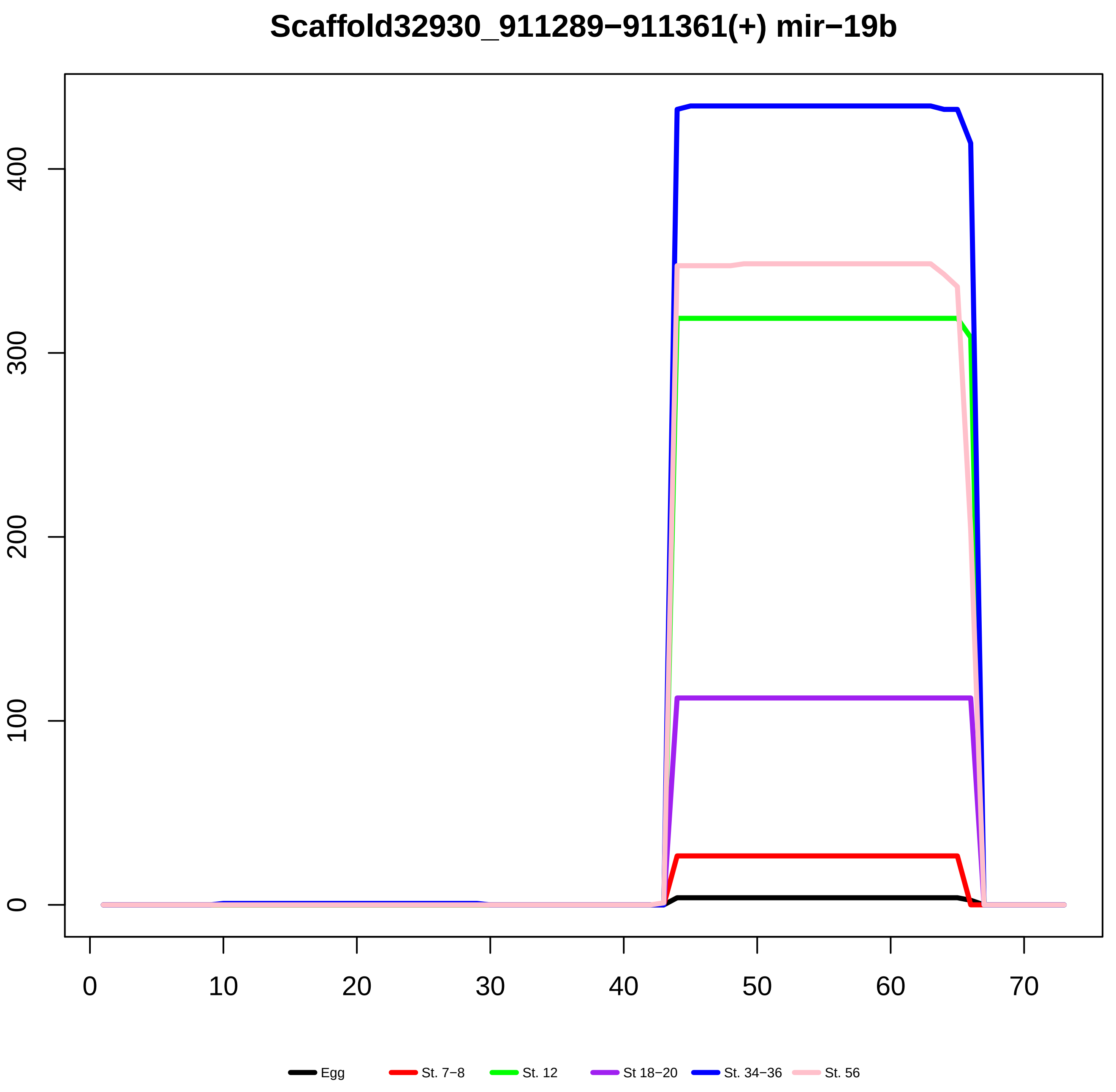

Scaffold32930_911289−911361(+) mir−19b
0
100
200
300
400
0
10
20
30
40
50
60
70
Egg
St. 7−8
St. 12
St 18−20
St. 34−36
St. 56

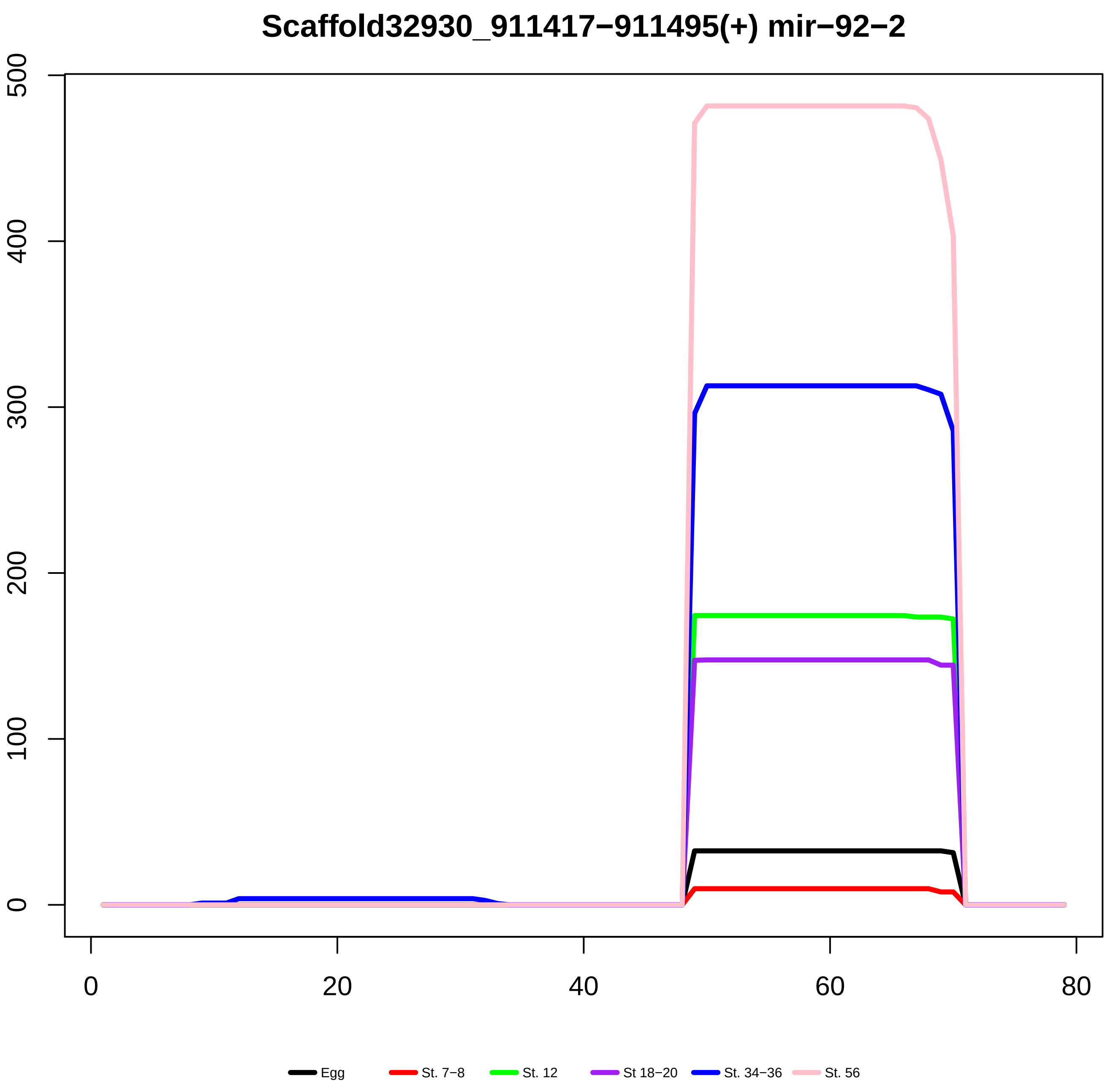

Scaffold32930_911417−911495(+) mir−92−2
0
100
200
300
400
500
0
20
40
60
80
Egg
St. 7−8
St. 12
St 18−20
St. 34−36
St. 56

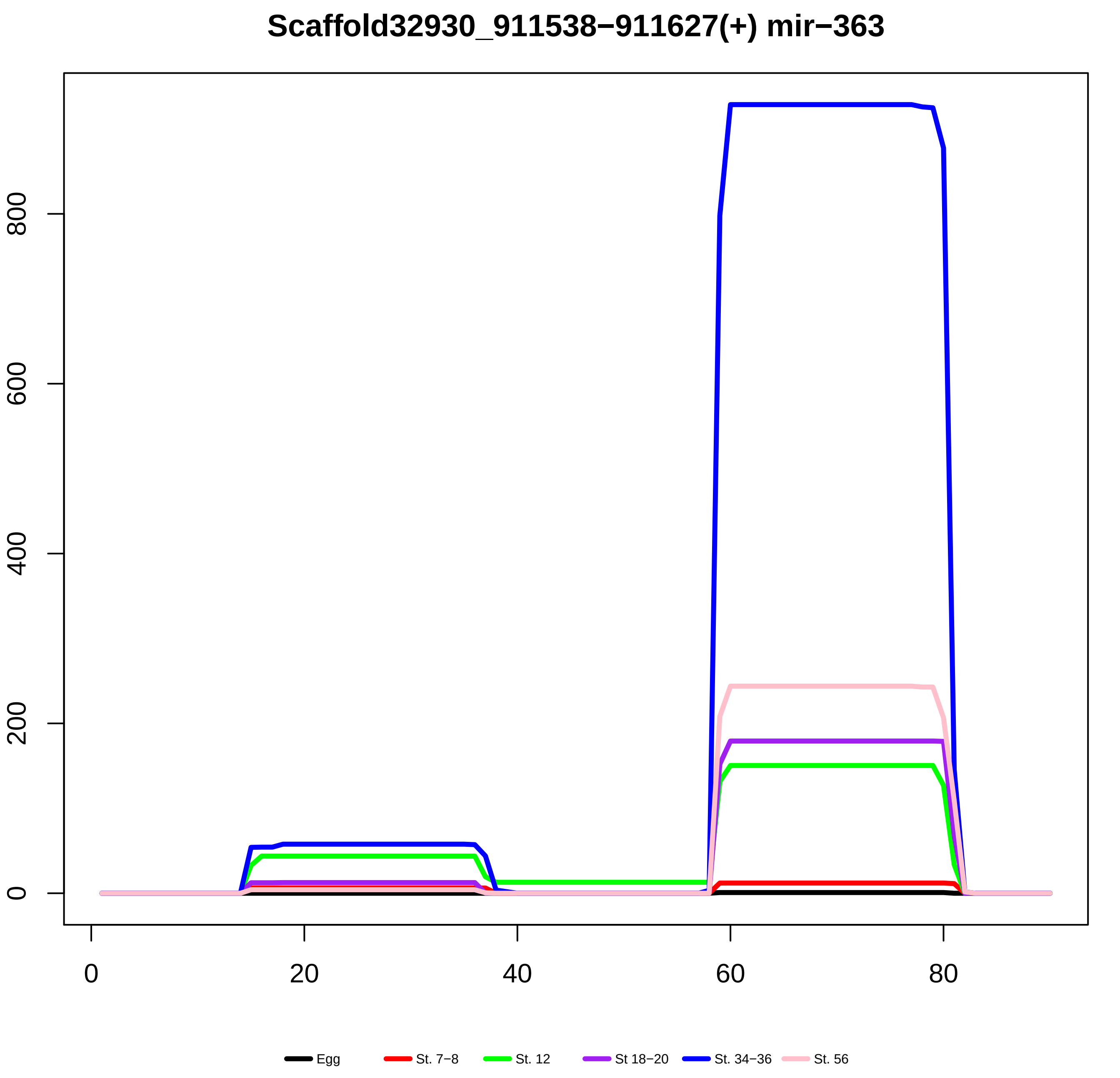

Scaffold32930_911538−911627(+) mir−363
0
200
400
600
800
0
20
40
60
80
Egg
St. 7−8
St. 12
St 18−20
St. 34−36
St. 56

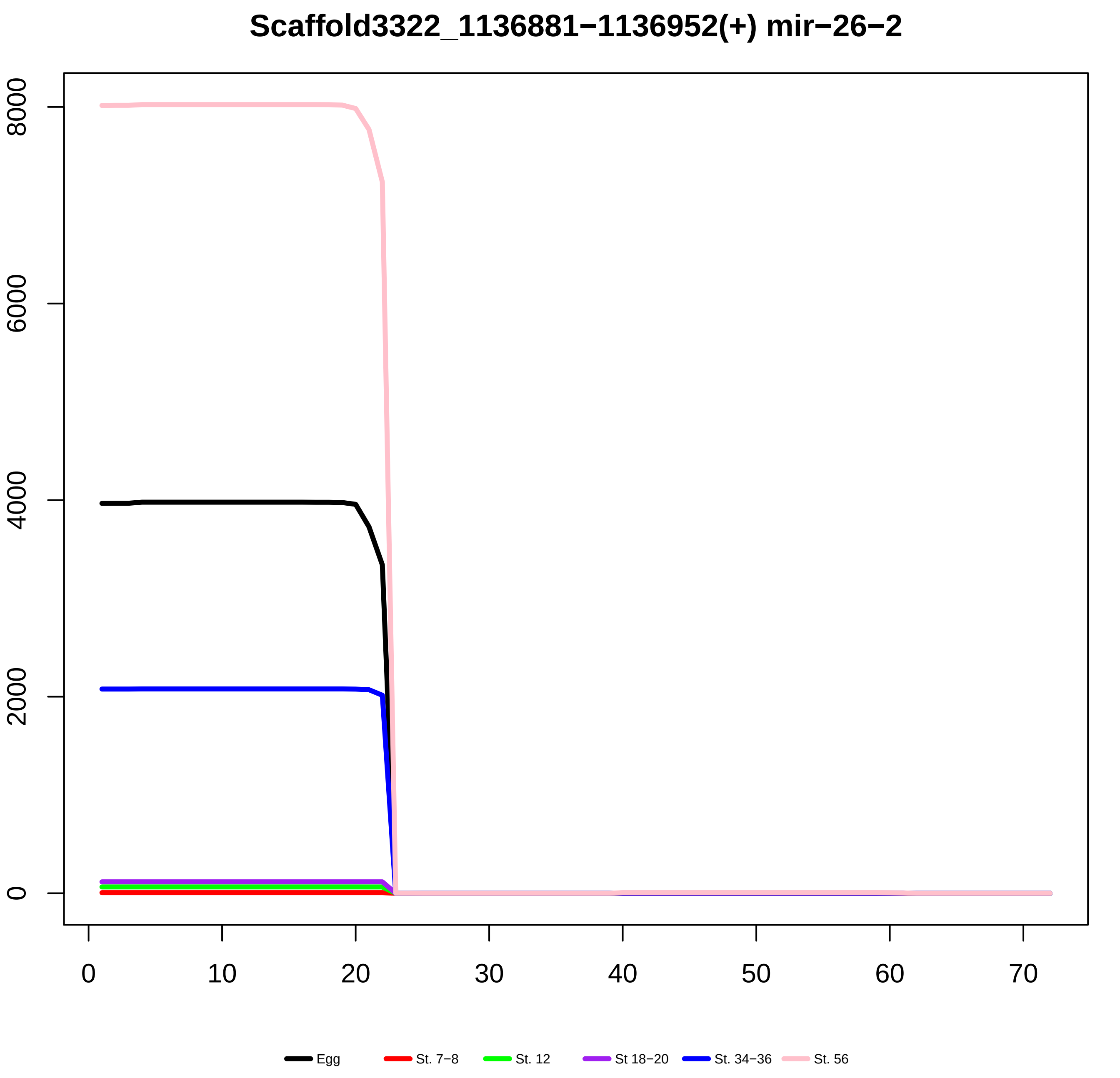
Scaffold3322_1136881−1136952(+) mir−26−2
0
2000
4000
6000
8000
0
10
20
30
40
50
60
70
Egg
St. 7−8
St. 12
St 18−20
St. 34−36
St. 56

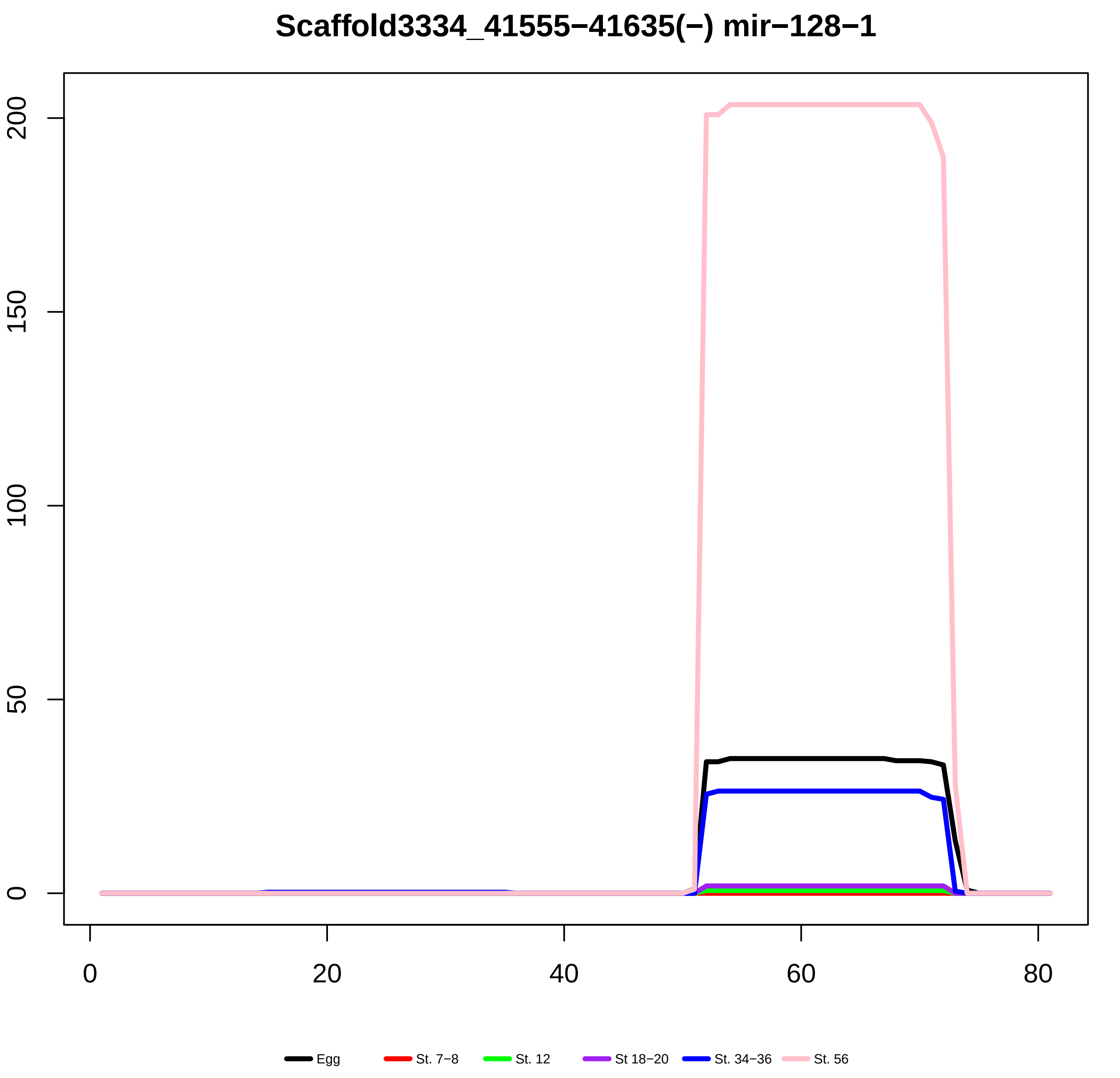

Scaffold3334_41555−41635(−) mir−128−1
0
50
100
150
200
0
20
40
60
80
Egg
St. 7−8
St. 12
St 18−20
St. 34−36
St. 56

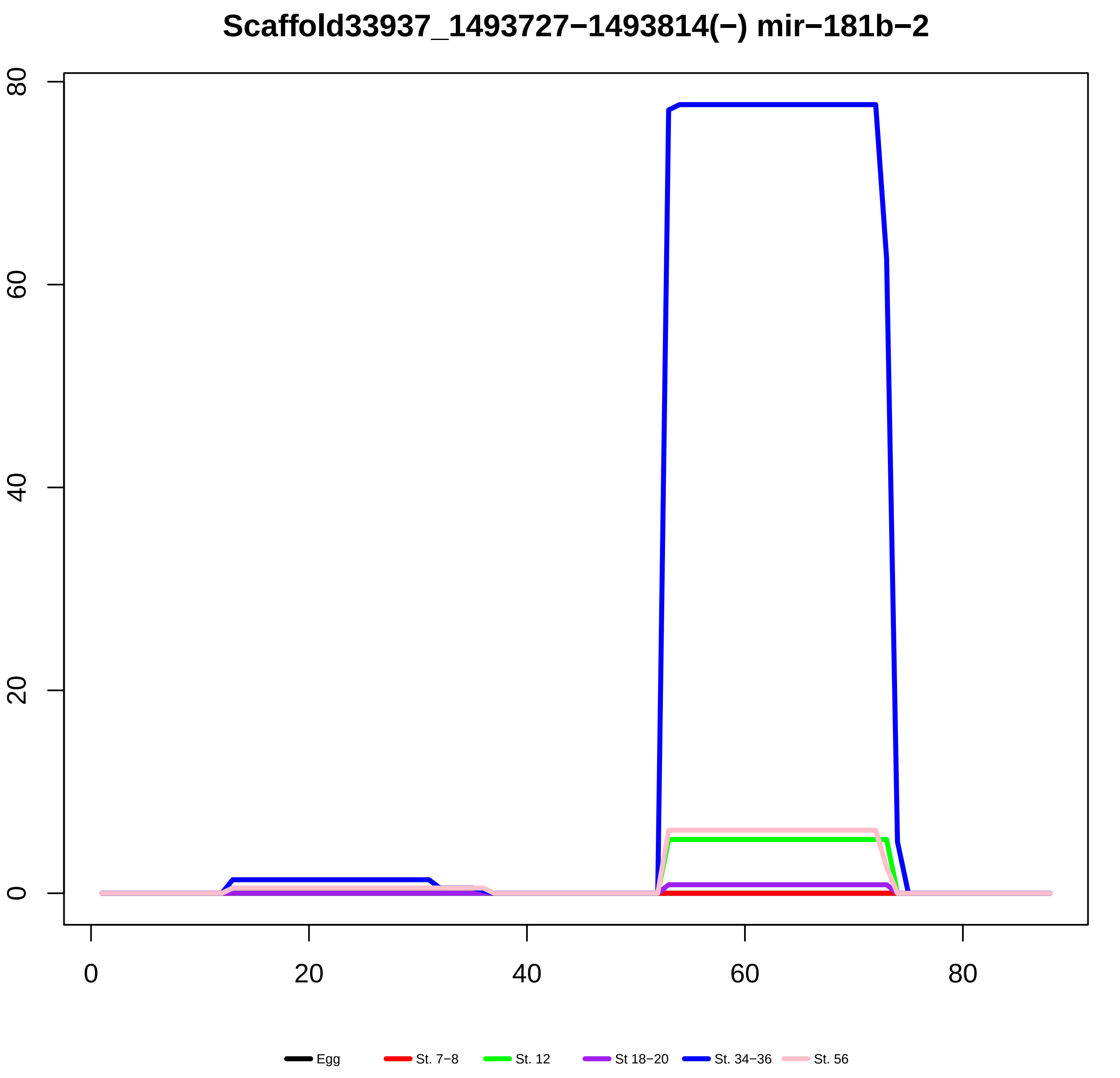

Scaffold33937_1493727−1493814(−) mir−181b−2
0
20
40
60
80
0
20
40
60
80
Egg
St. 7−8
St. 12
St 18−20
St. 34−36
St. 56

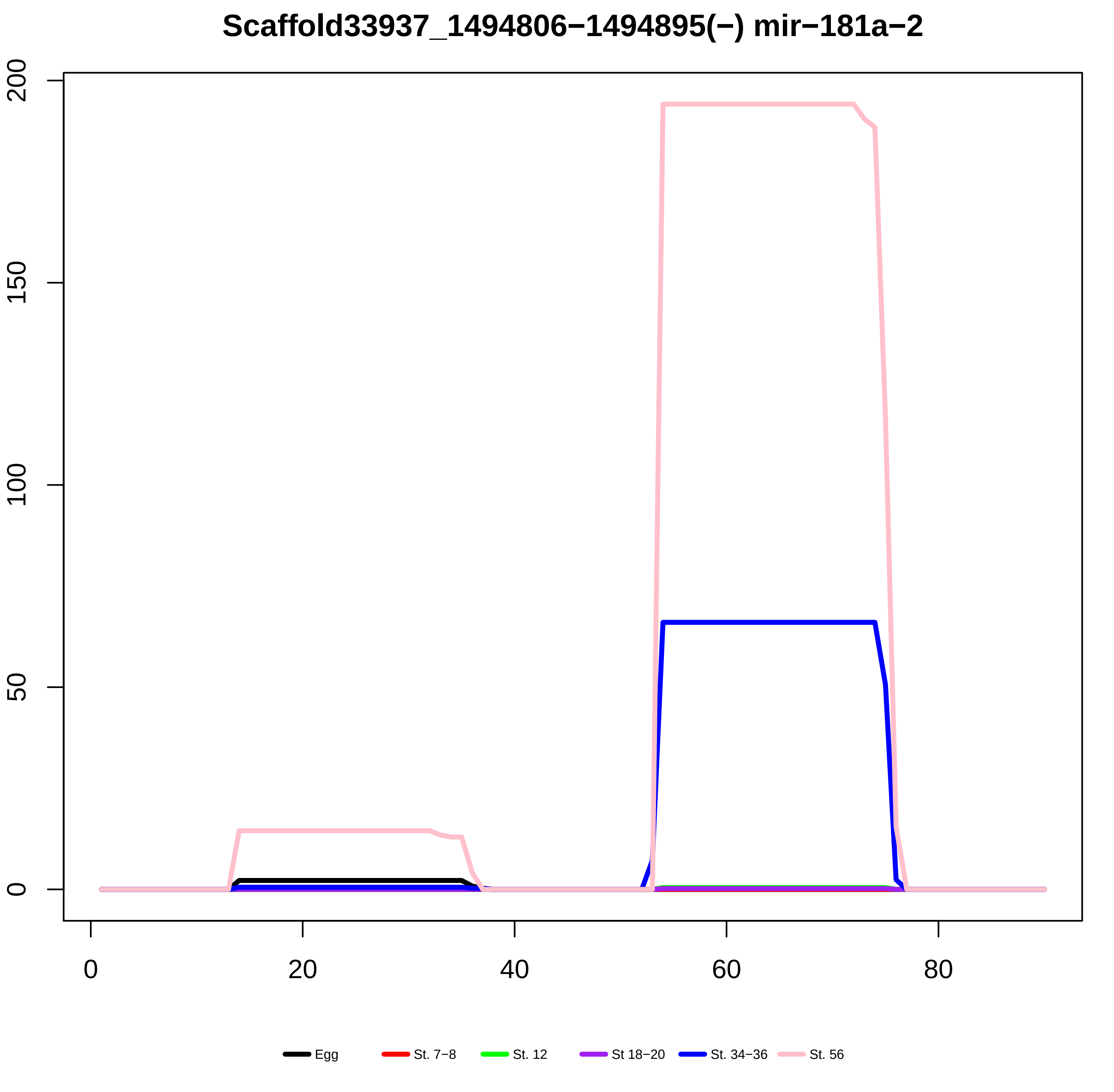

Scaffold33937_1494806−1494895(−) mir−181a−2
0
50
100
150
200
0
20
40
60
80
Egg
St. 7−8
St. 12
St 18−20
St. 34−36
St. 56

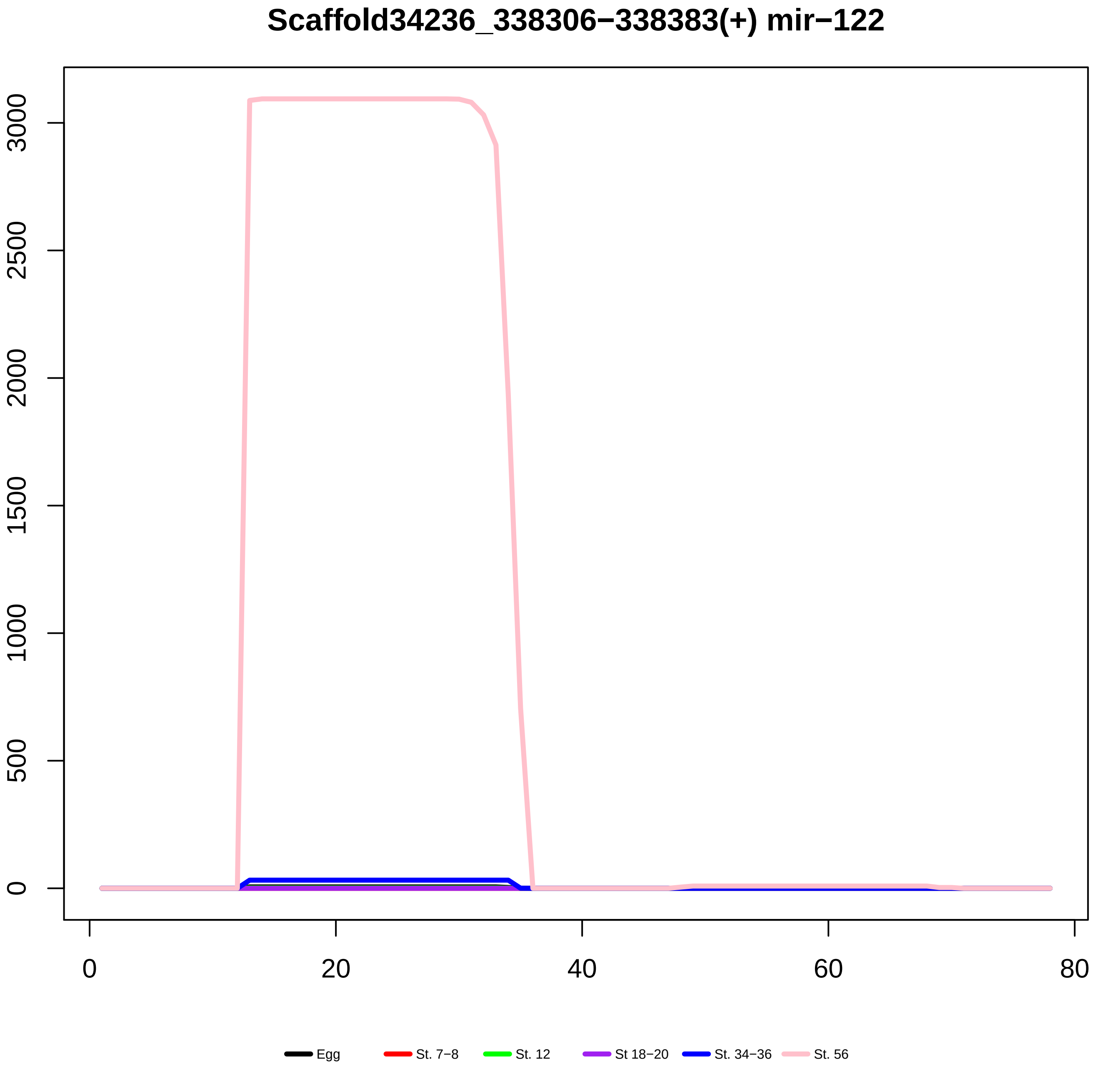
Scaffold34236_338306−338383(+) mir−122
0
500
1000
1500
2000
2500
3000
0
20
40
60
80
Egg
St. 7−8
St. 12
St 18−20
St. 34−36
St. 56

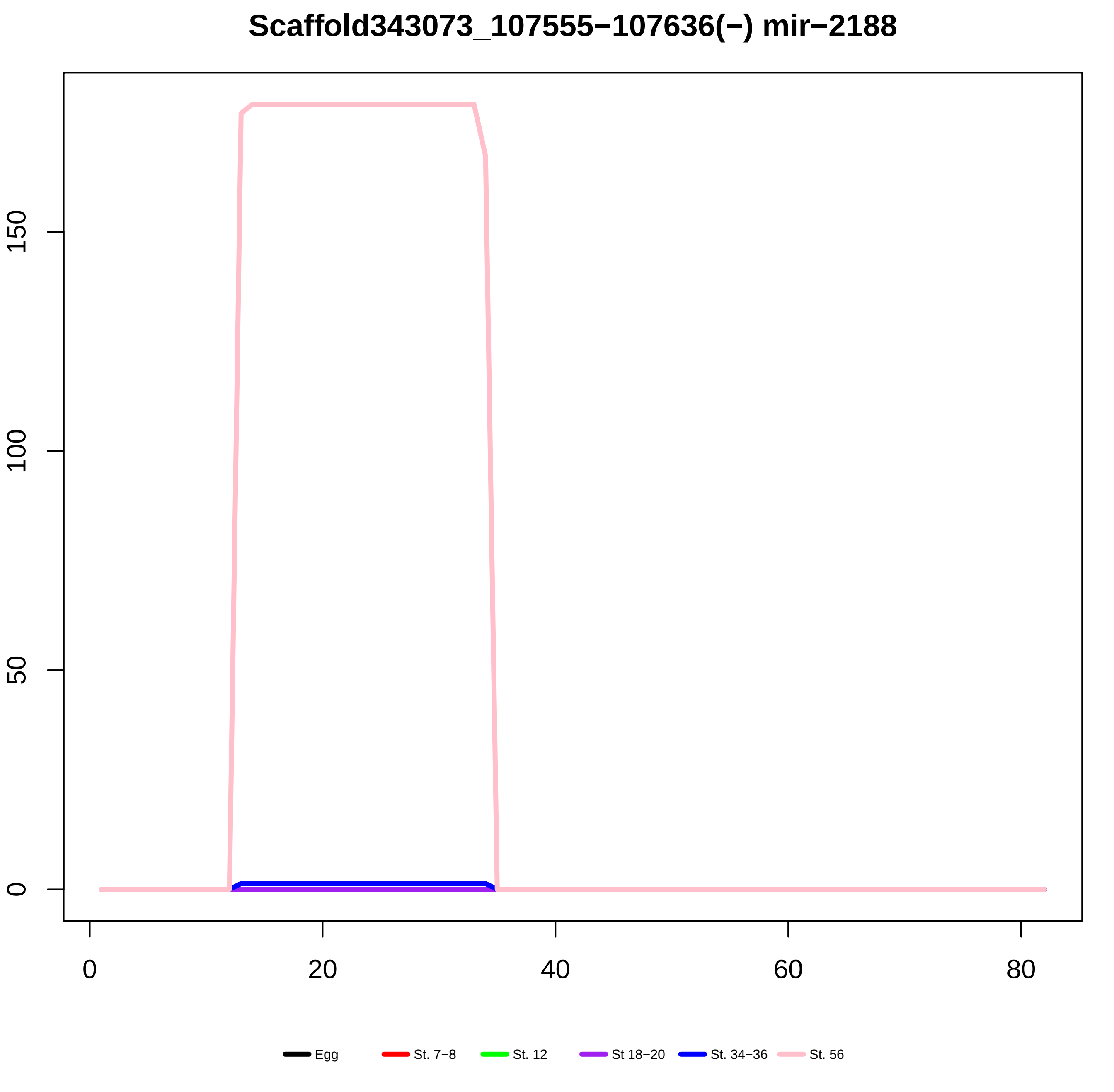

Scaffold343073_107555−107636(−) mir−2188
0
50
100
150
0
20
40
60
80
Egg
St. 7−8
St. 12
St 18−20
St. 34−36
St. 56

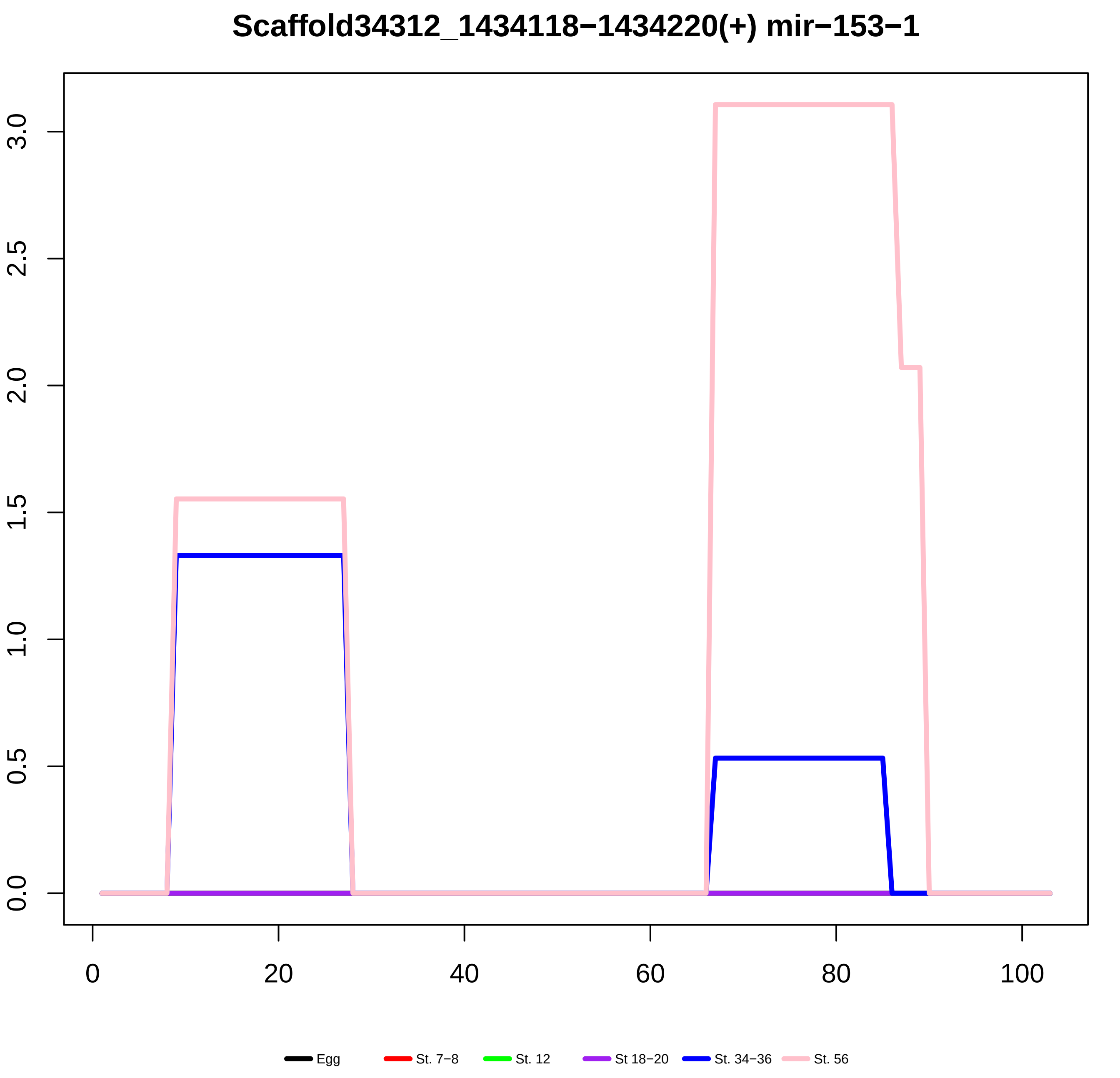

Scaffold34312_1434118−1434220(+) mir−153−1
0.0
0.5
1.0
1.5
2.0
2.5
3.0
0
20
40
60
80
100
Egg
St. 7−8
St. 12
St 18−20
St. 34−36
St. 56

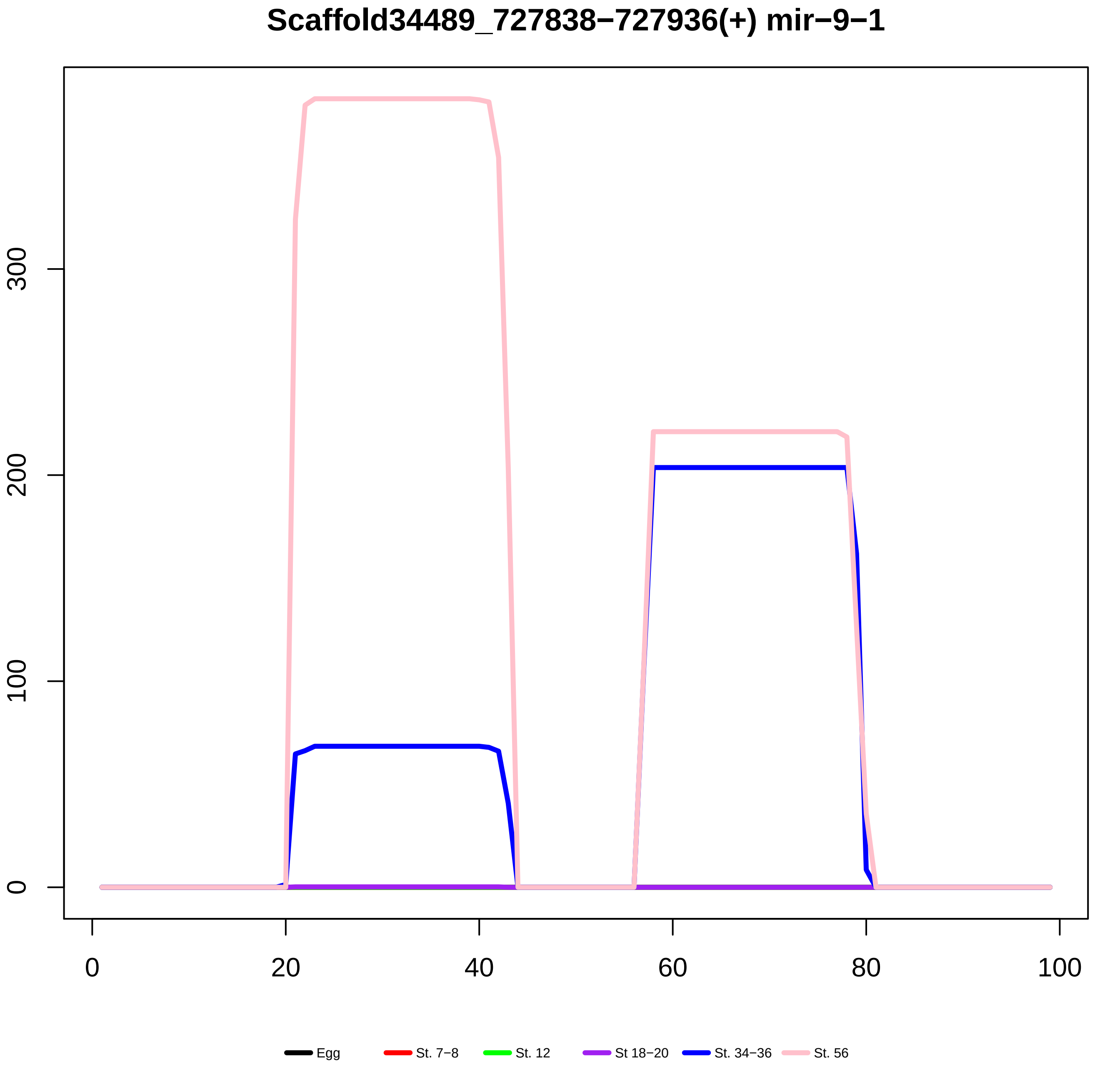

Scaffold34489_727838−727936(+) mir−9−1
0
100
200
300
0
20
40
60
80
100
Egg
St. 7−8
St. 12
St 18−20
St. 34−36
St. 56

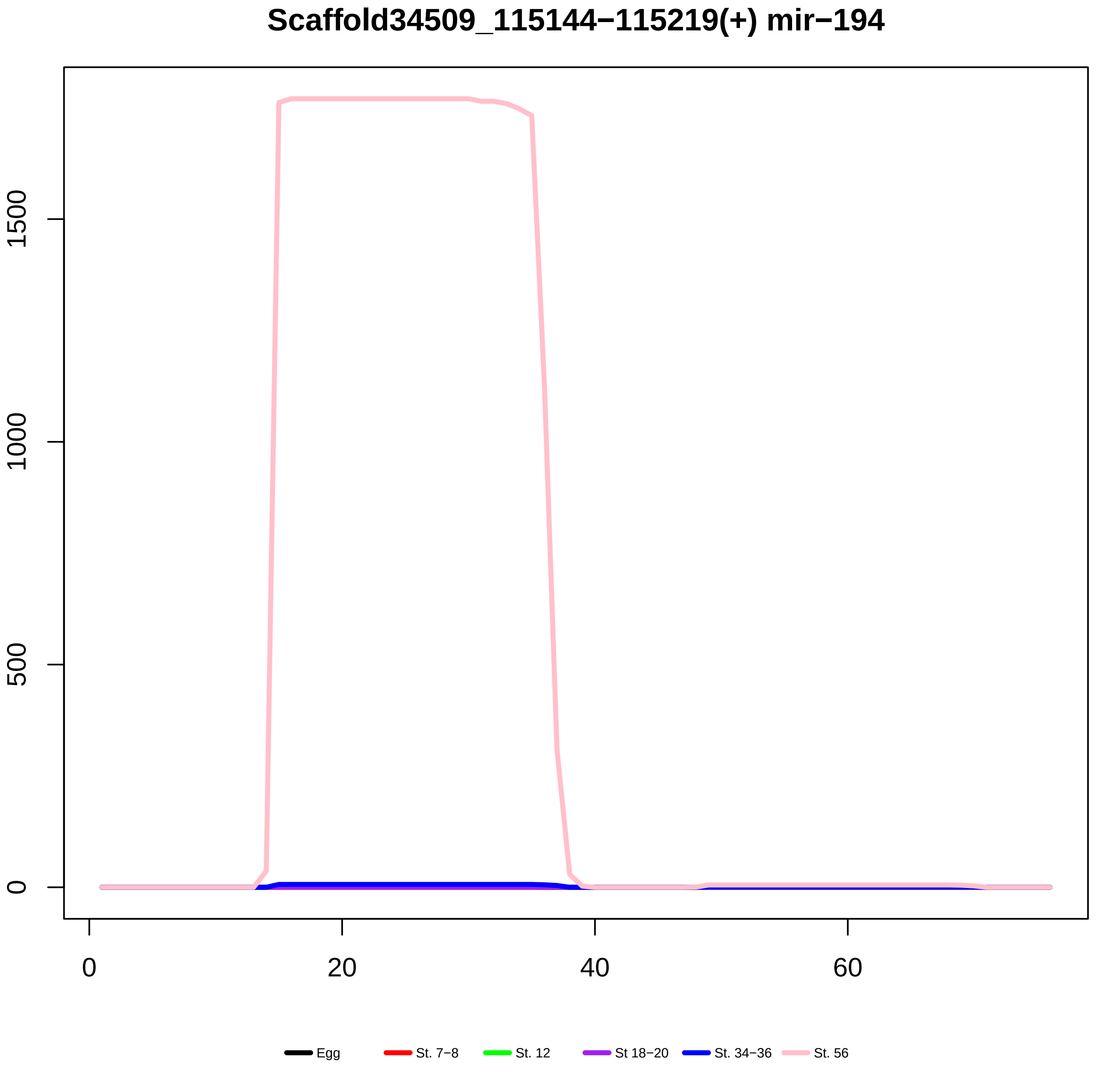

Scaffold34509_115144−115219(+) mir−194
0
500
1000
1500
0
20
40
60
Egg
St. 7−8
St. 12
St 18−20
St. 34−36
St. 56

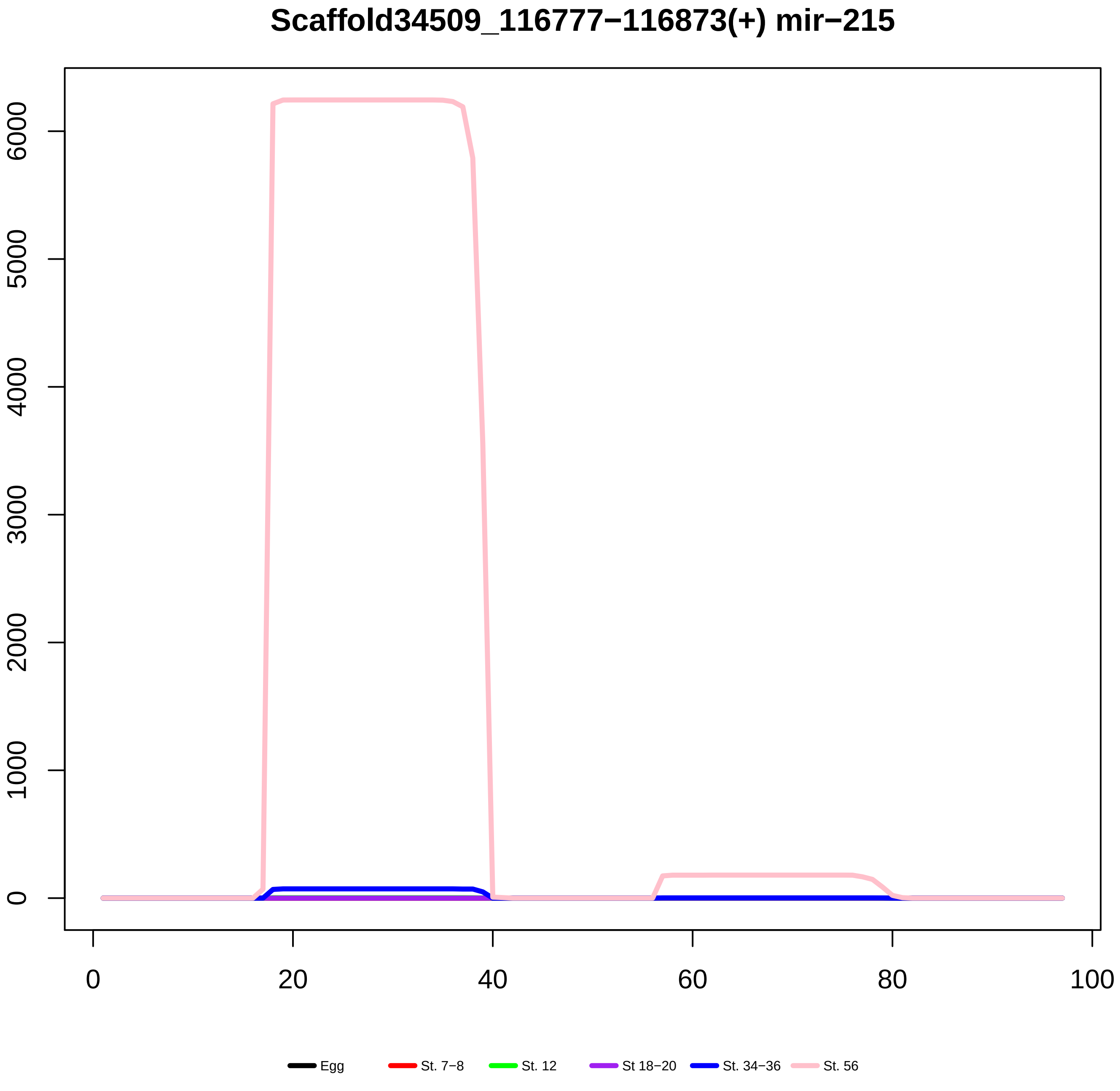

Scaffold34509_116777−116873(+) mir−215
0
1000
2000
3000
4000
5000
6000
0
20
40
60
80
100
Egg
St. 7−8
St. 12
St 18−20
St. 34−36
St. 56

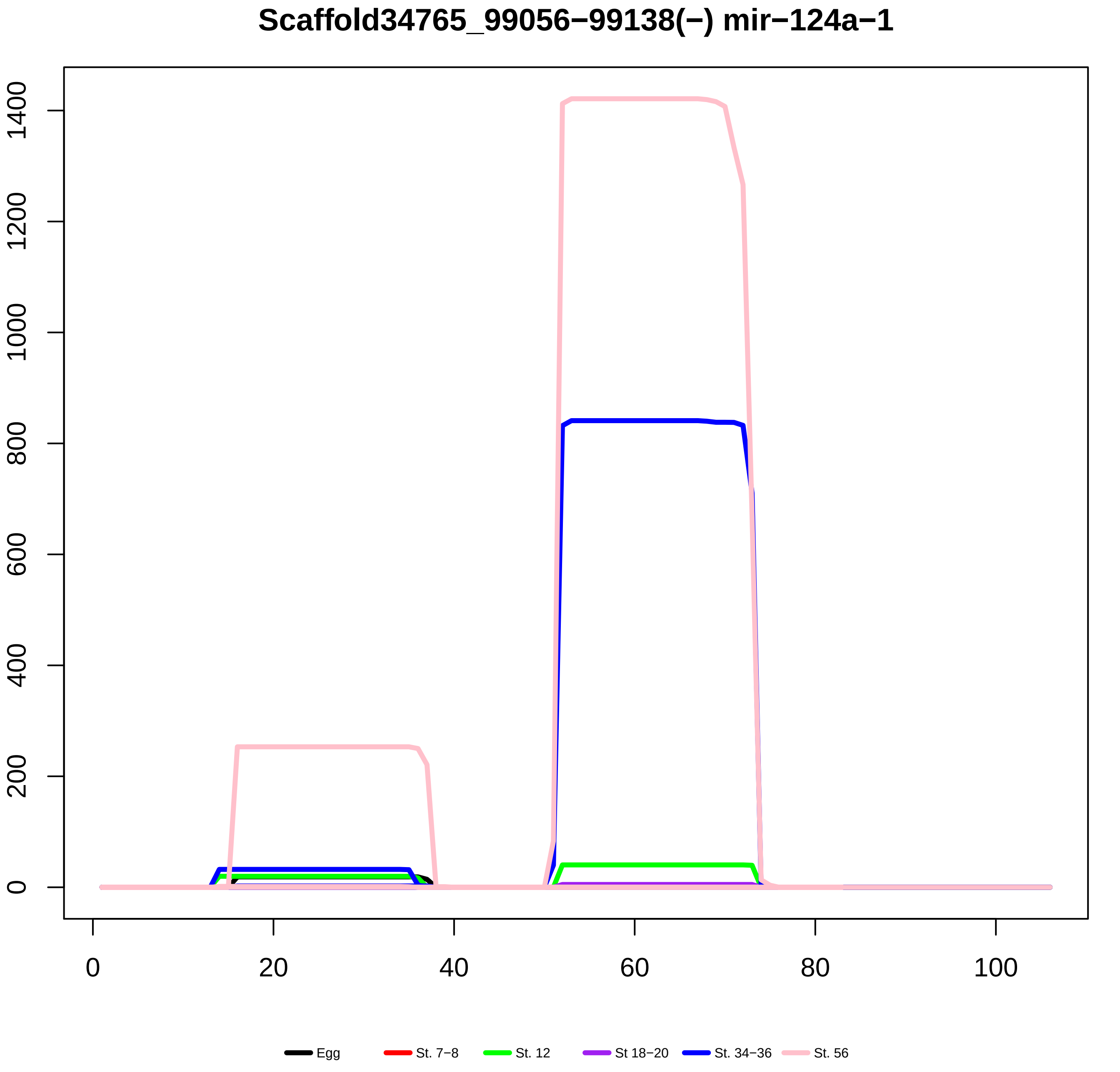

Scaffold34765_99056−99138(−) mir−124a−1
0
200
400
600
800
1000
1200
1400
0
20
40
60
80
100
Egg
St. 7−8
St. 12
St 18−20
St. 34−36
St. 56

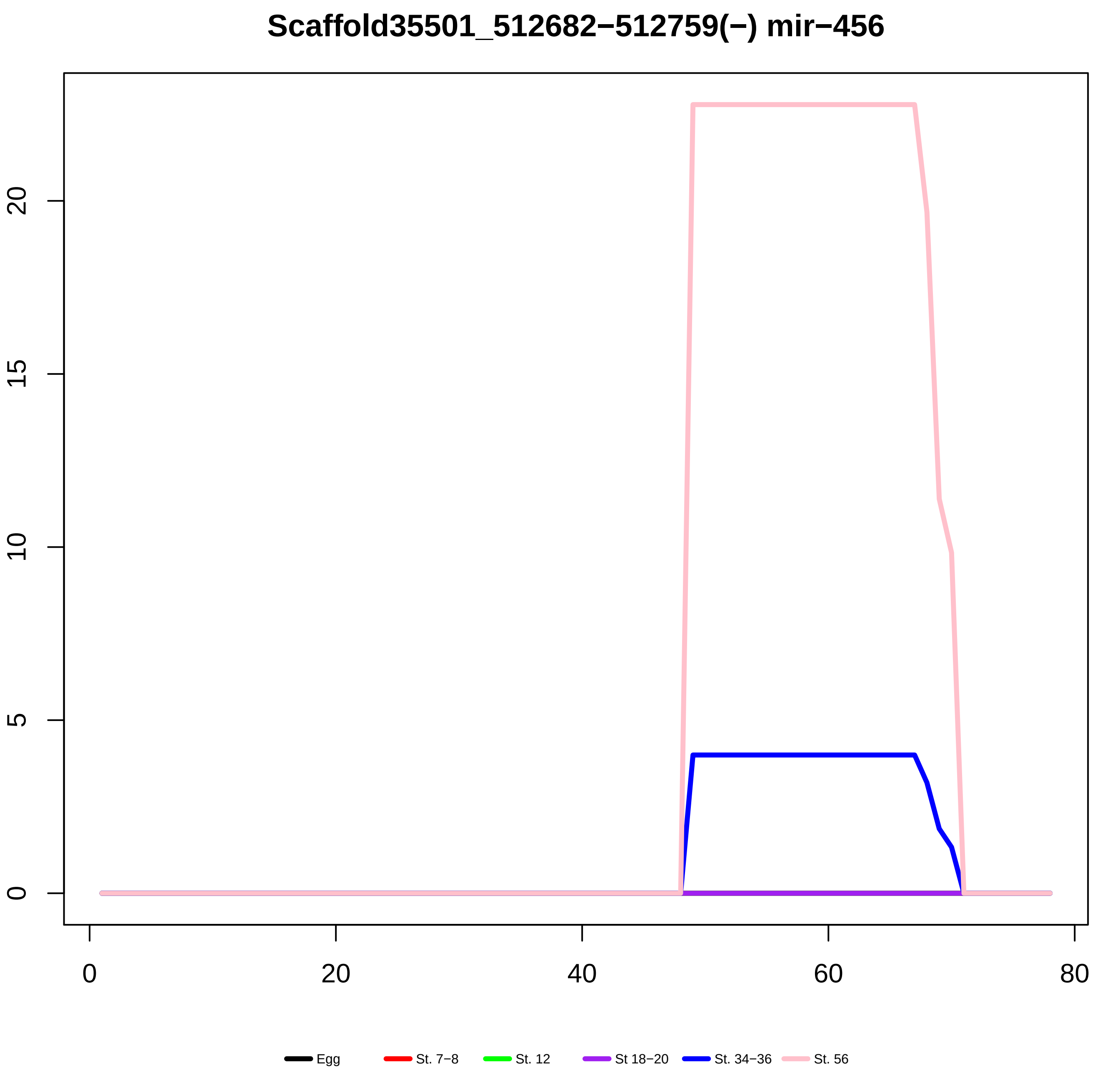

Scaffold35501_512682−512759(−) mir−456
0
5
10
15
20
0
20
40
60
80
Egg
St. 7−8
St. 12
St 18−20
St. 34−36
St. 56

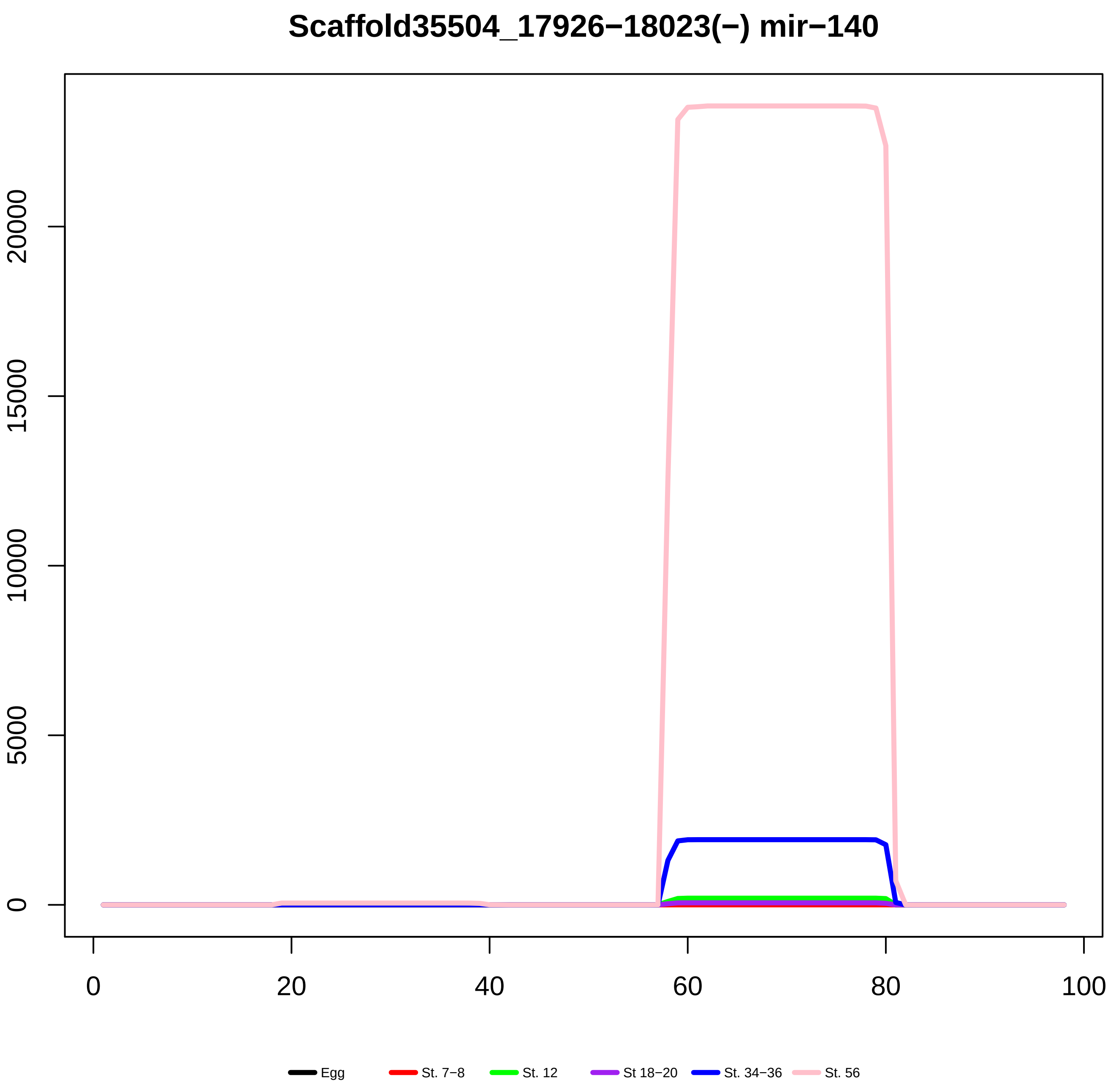

Scaffold35504_17926−18023(−) mir−140
0
5000
10000
15000
20000
0
20
40
60
80
100
Egg
St. 7−8
St. 12
St 18−20
St. 34−36
St. 56

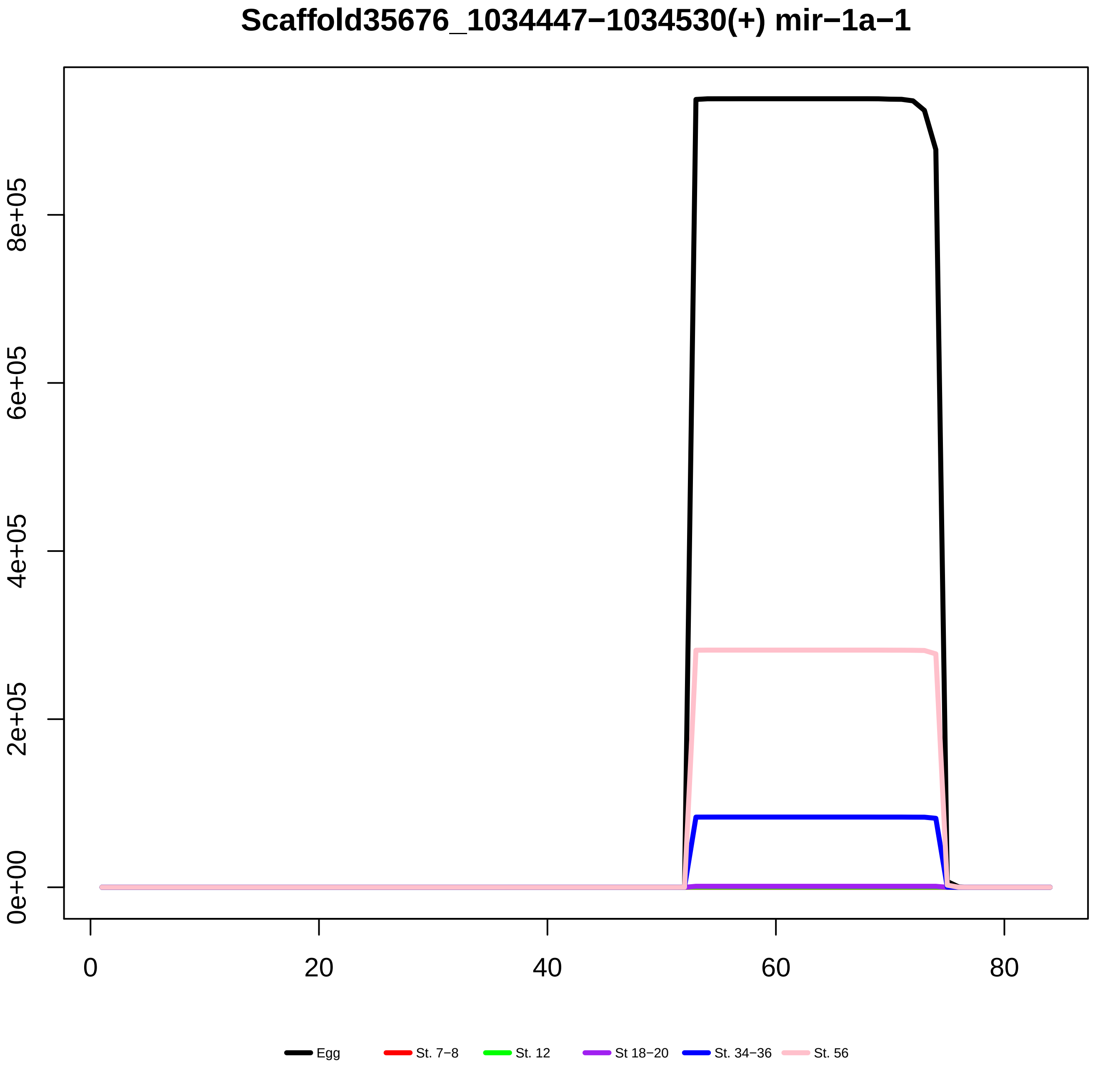

Scaffold35676_1034447−1034530(+) mir−1a−1
0e+00
2e+05
4e+05
6e+05
8e+05
0
20
40
60
80
Egg
St. 7−8
St. 12
St 18−20
St. 34−36
St. 56

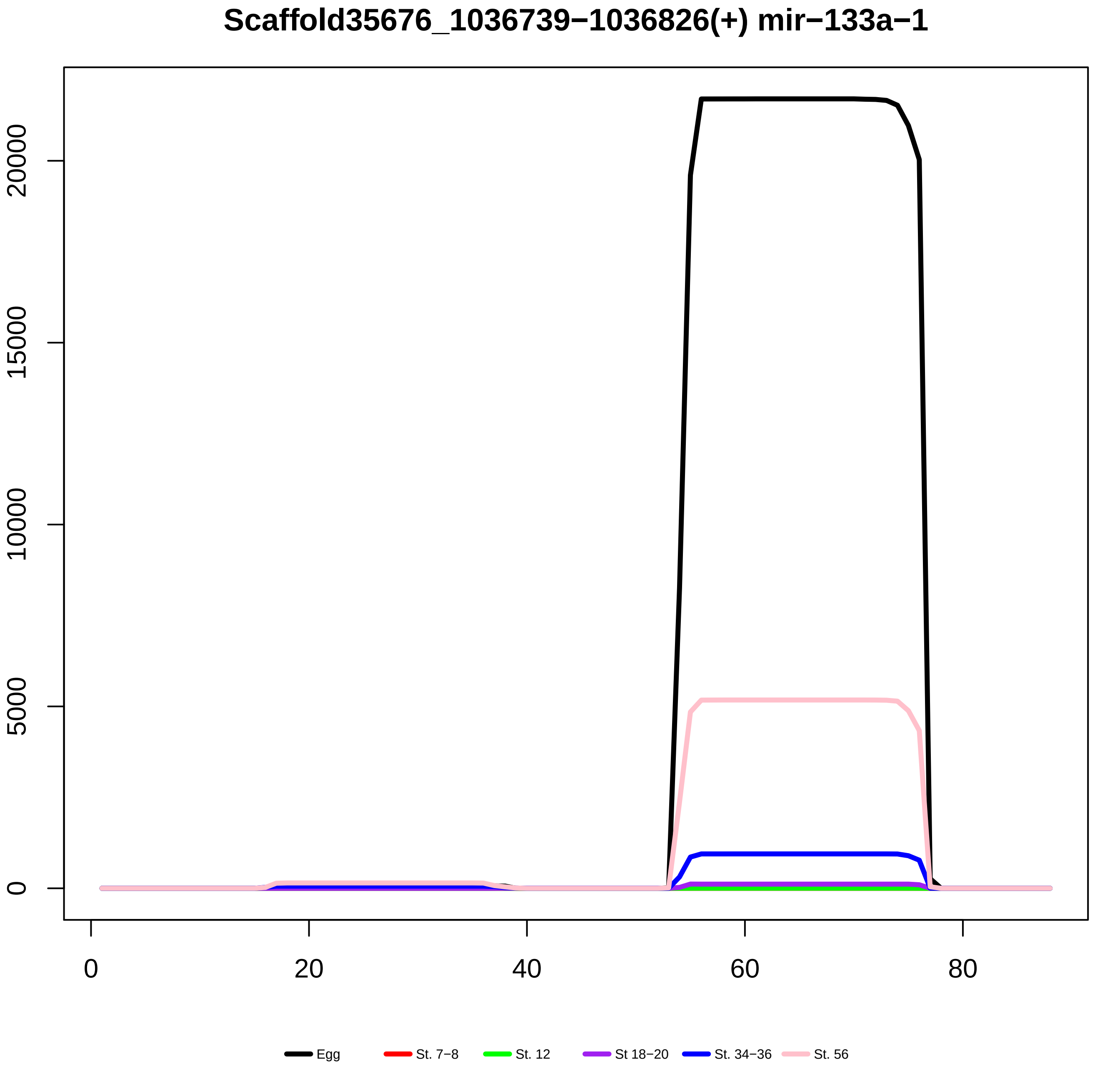

Scaffold35676_1036739−1036826(+) mir−133a−1
0
5000
10000
15000
20000
0
20
40
60
80
Egg
St. 7−8
St. 12
St 18−20
St. 34−36
St. 56

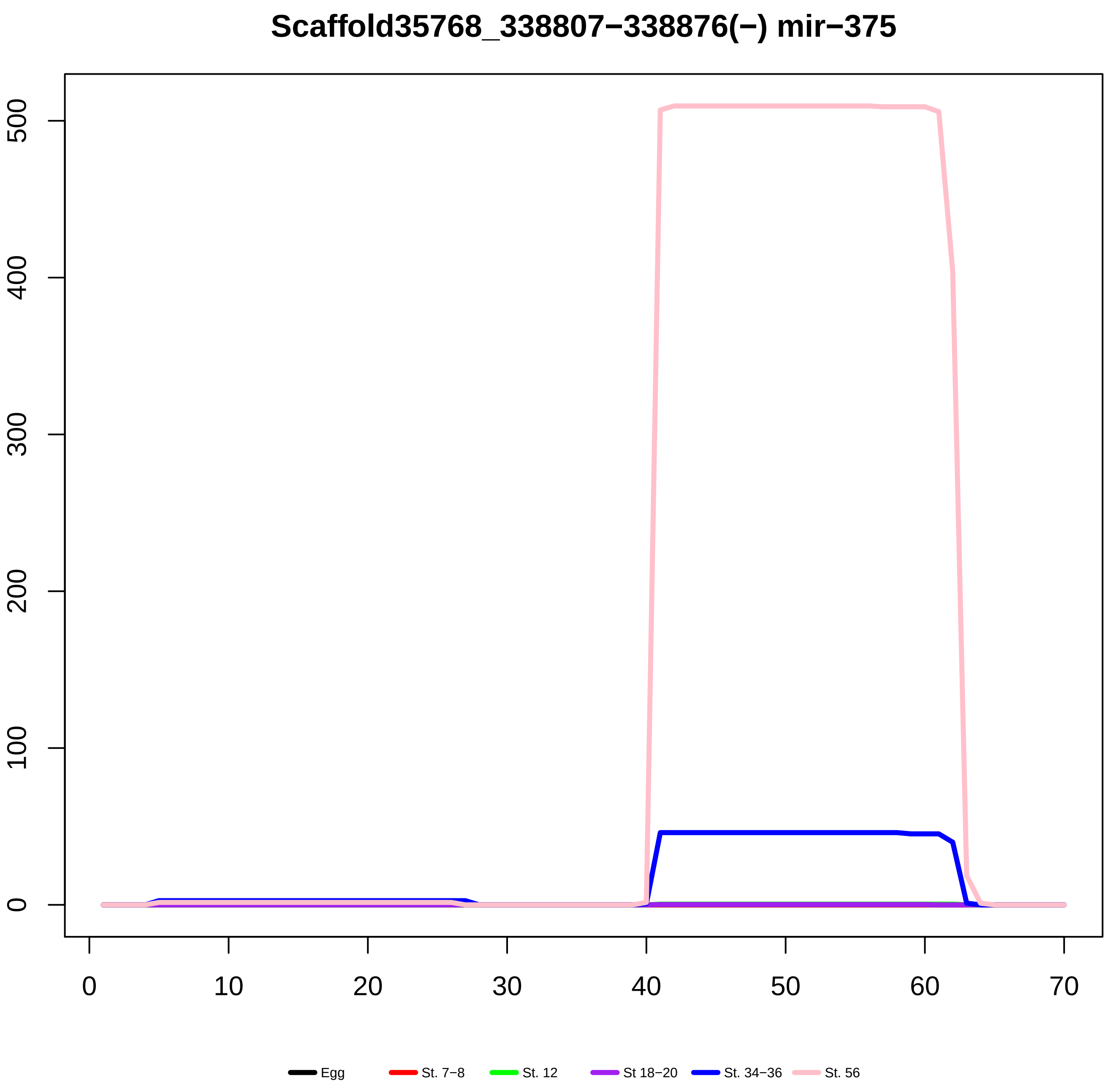
Scaffold35768_338807−338876(−) mir−375
0
100
200
300
400
500
0
10
20
30
40
50
60
70
Egg
St. 7−8
St. 12
St 18−20
St. 34−36
St. 56

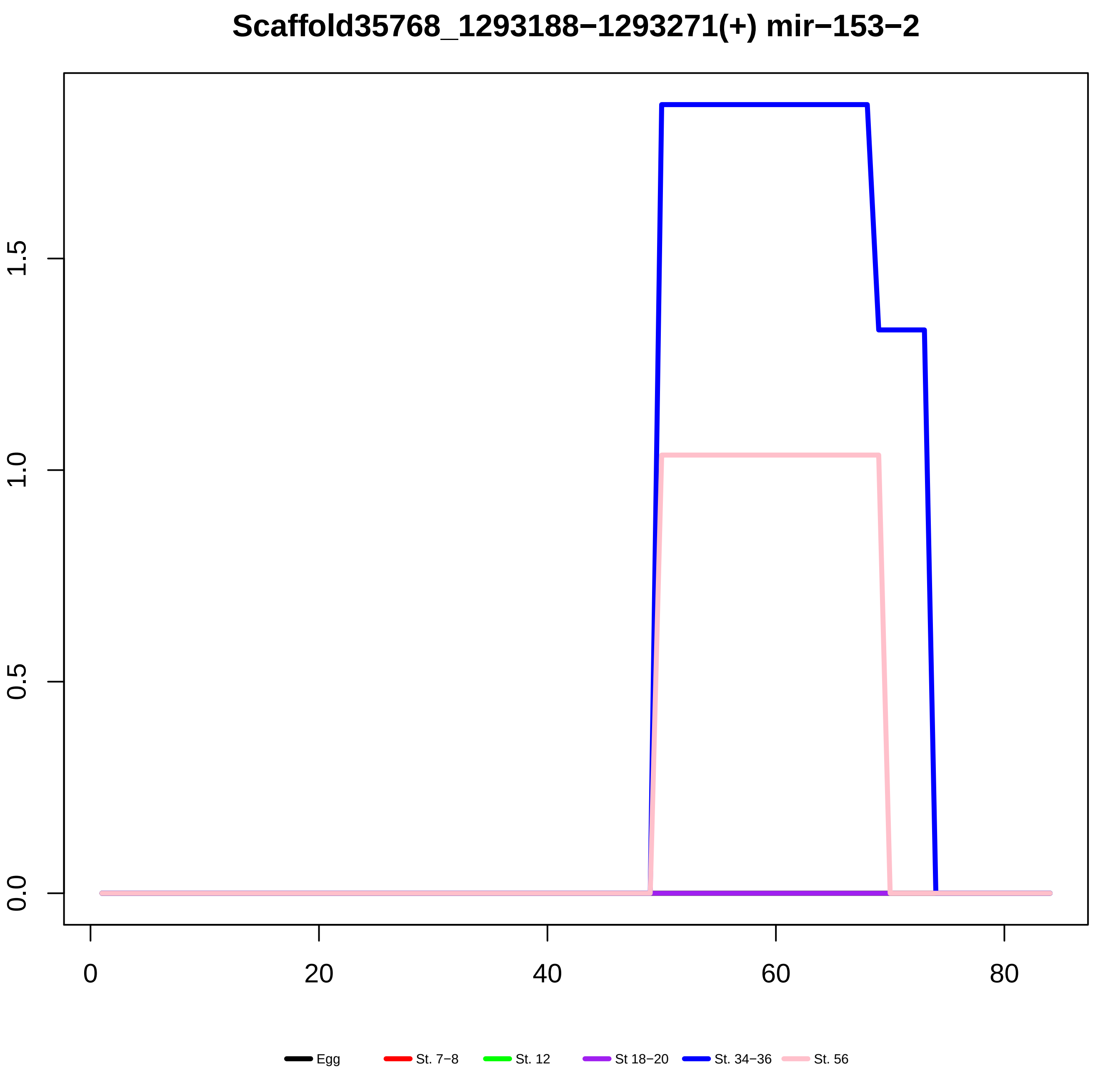
Scaffold35768_1293188−1293271(+) mir−153−2
0.0
0.5
1.0
1.5
0
20
40
60
80
Egg
St. 7−8
St. 12
St 18−20
St. 34−36
St. 56

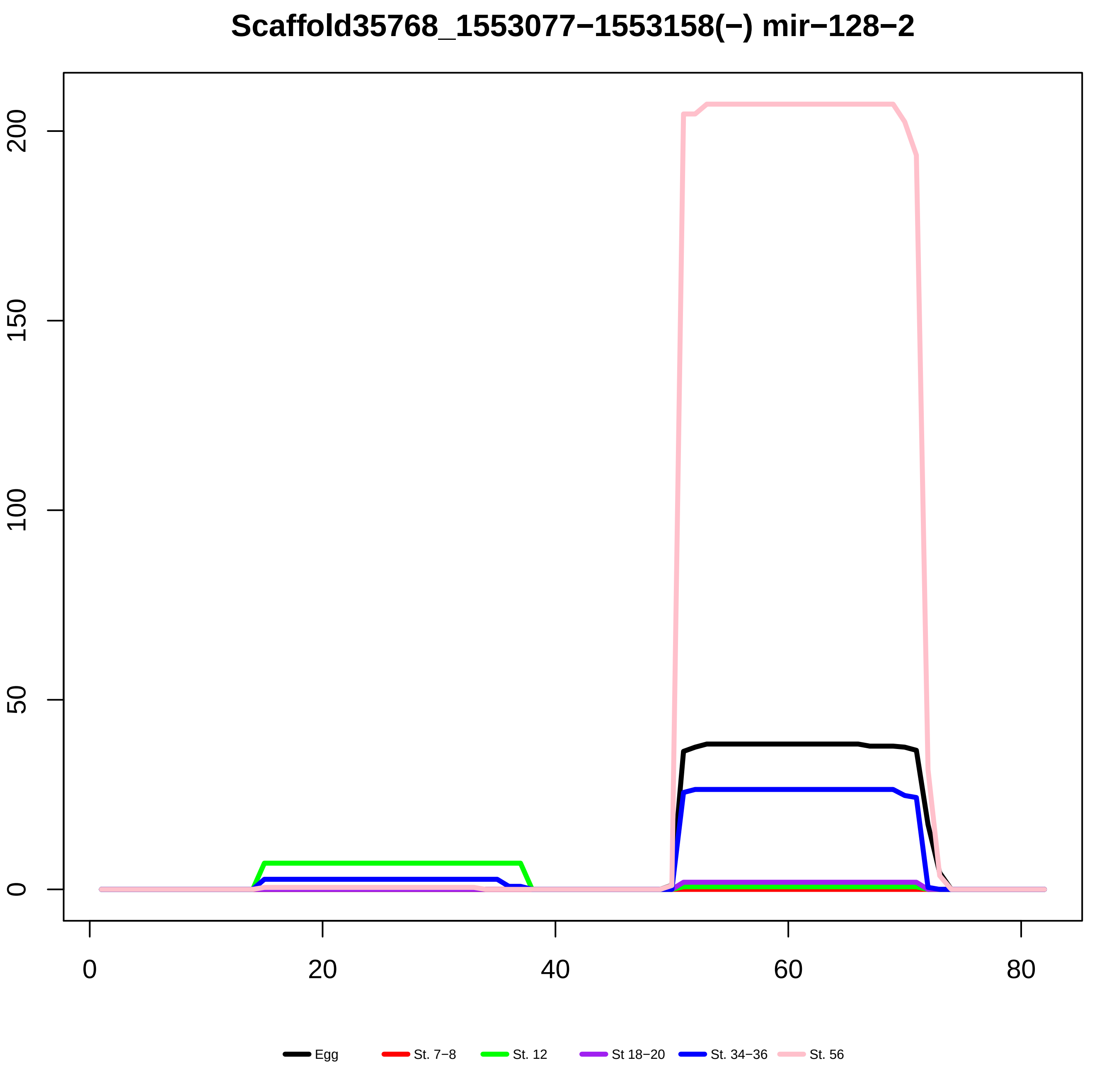

Scaffold35768_1553077−1553158(−) mir−128−2
0
50
100
150
200
0
20
40
60
80
Egg
St. 7−8
St. 12
St 18−20
St. 34−36
St. 56

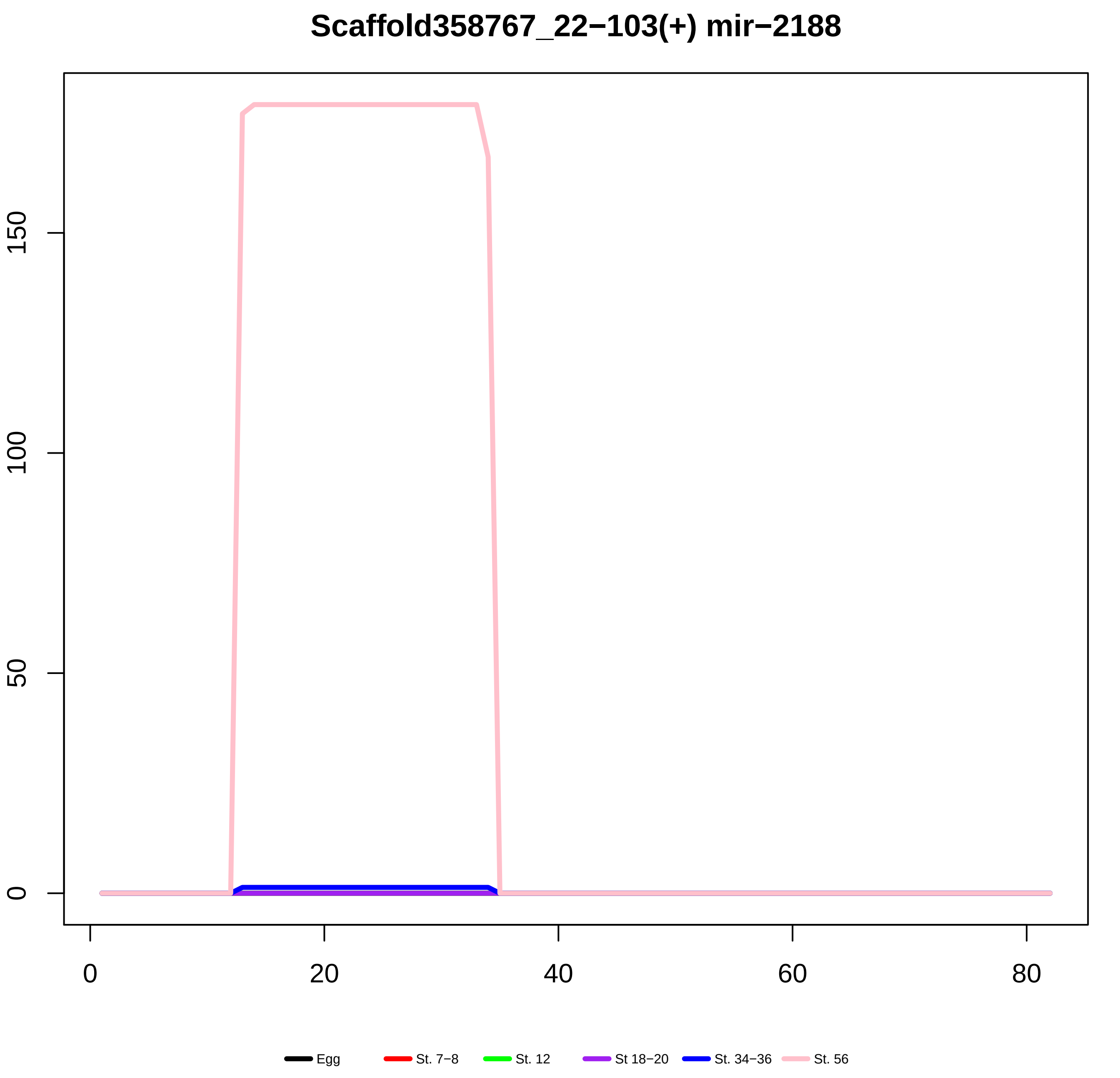

Scaffold358767_22−103(+) mir−2188
0
50
100
150
0
20
40
60
80
Egg
St. 7−8
St. 12
St 18−20
St. 34−36
St. 56

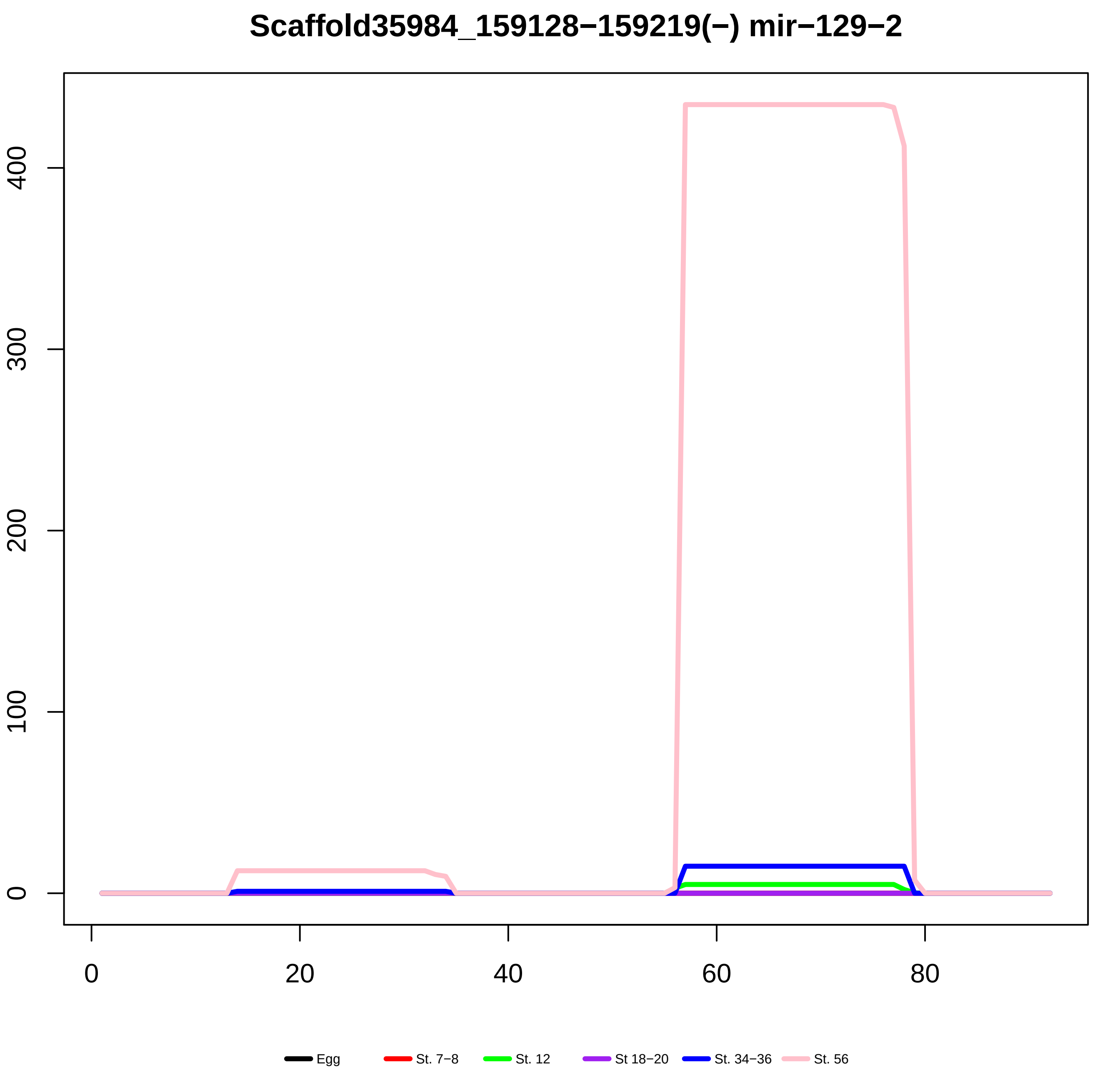

Scaffold35984_159128−159219(−) mir−129−2
0
100
200
300
400
0
20
40
60
80
Egg
St. 7−8
St. 12
St 18−20
St. 34−36
St. 56

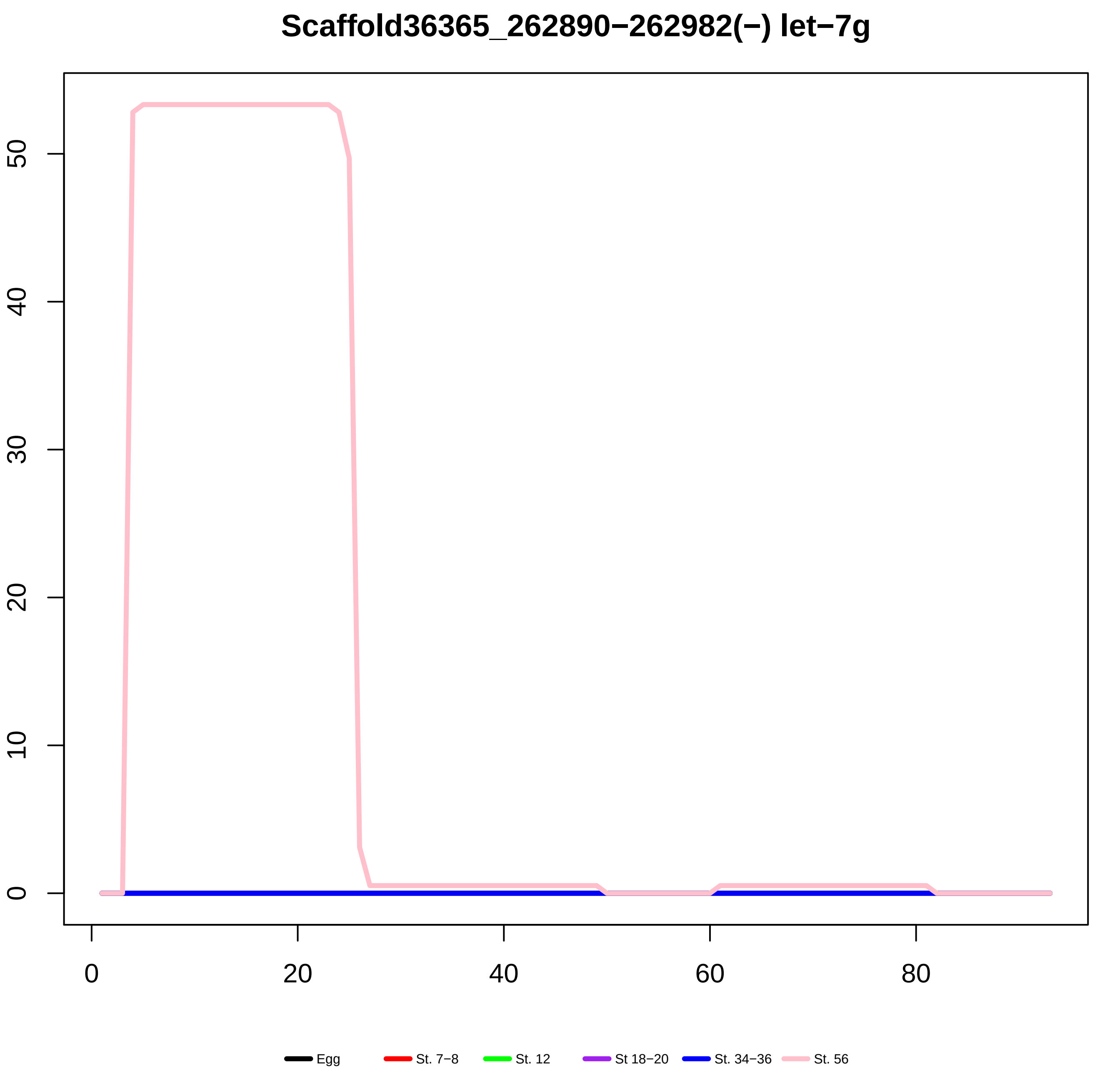

Scaffold36365_262890−262982(−) let−7g
0
10
20
30
40
50
0
20
40
60
80
Egg
St. 7−8
St. 12
St 18−20
St. 34−36
St. 56

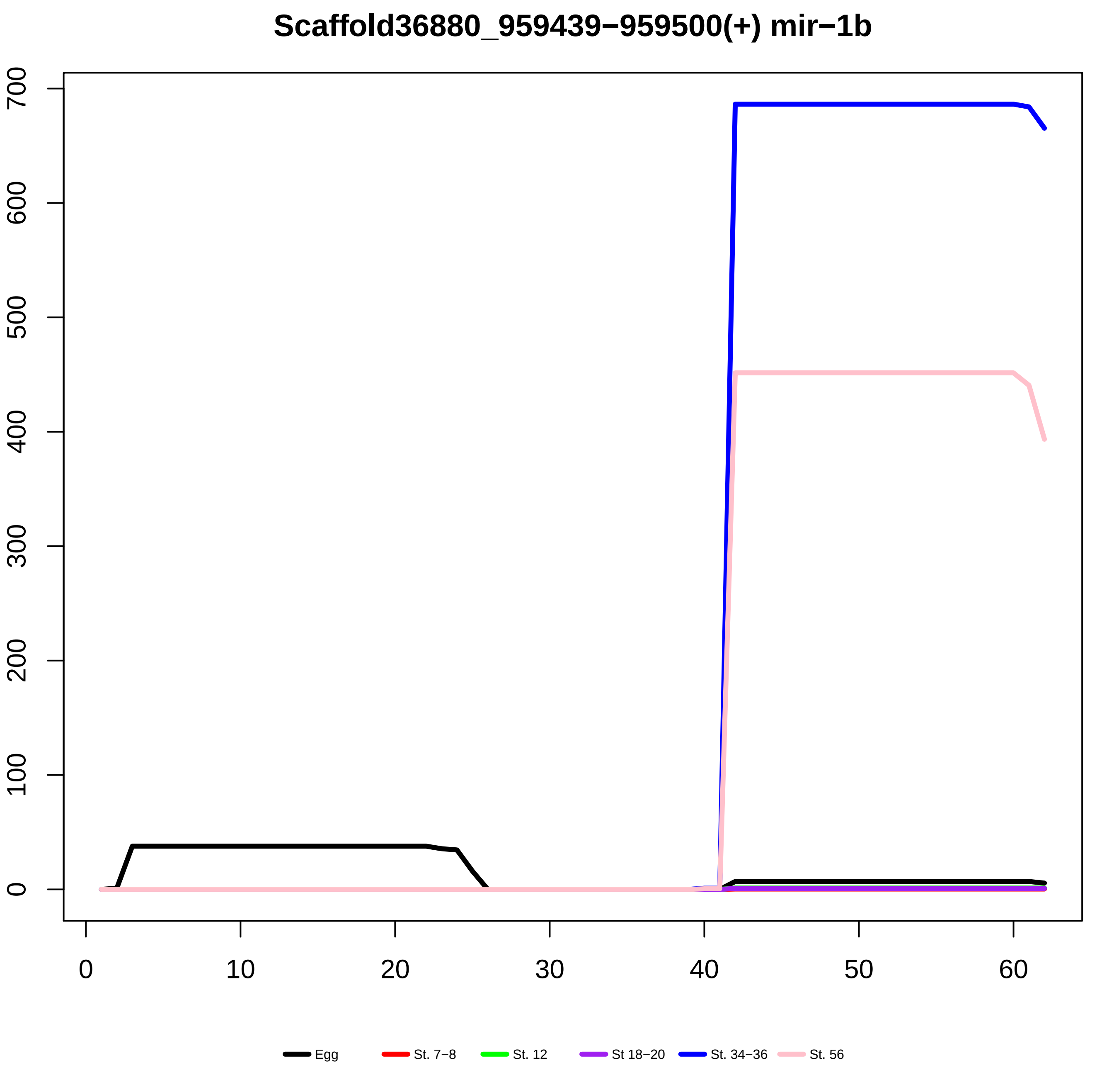

Scaffold36880_959439−959500(+) mir−1b
0
100
200
300
400
500
600
700
0
10
20
30
40
50
60
Egg
St. 7−8
St. 12
St 18−20
St. 34−36
St. 56

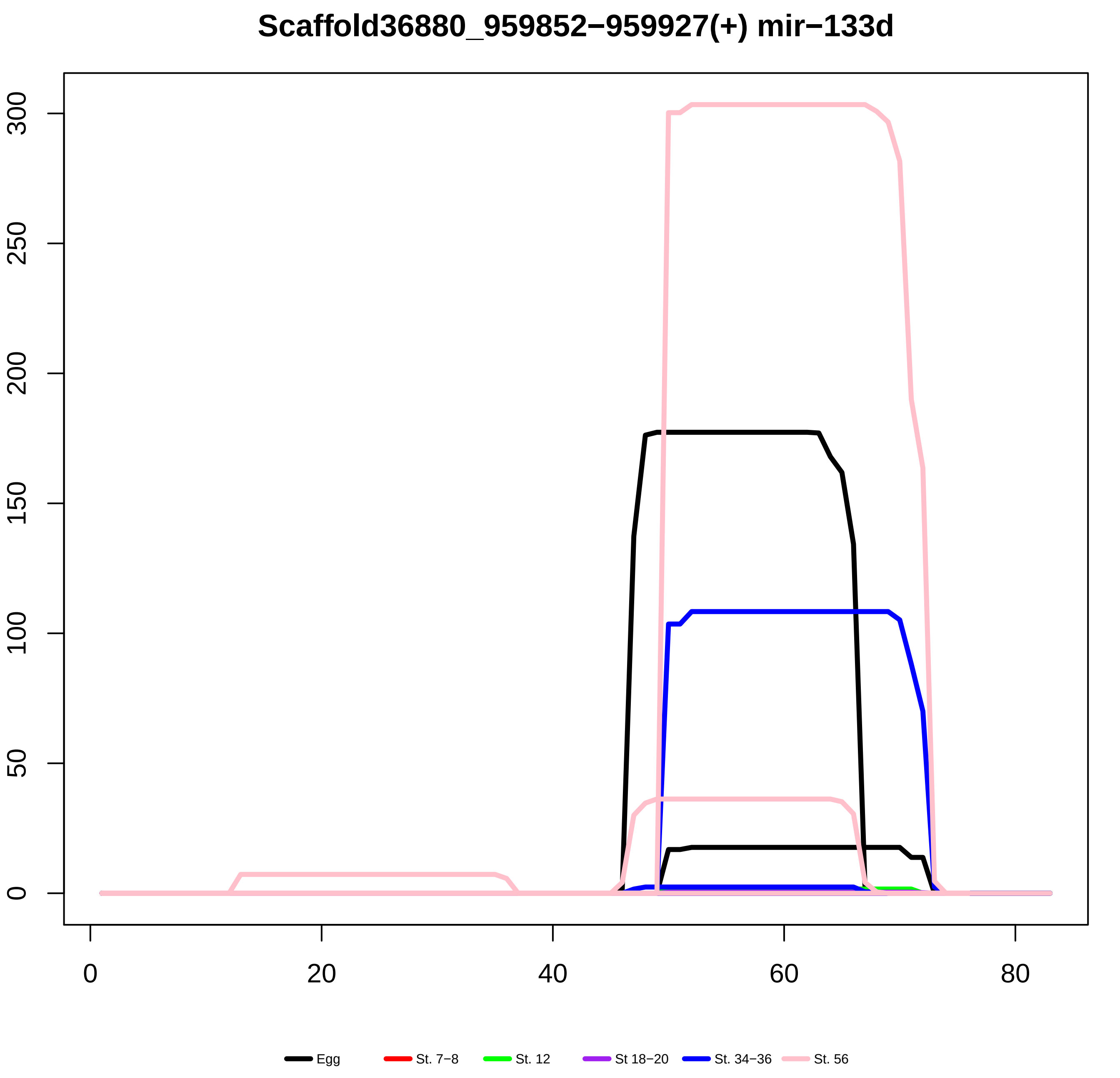
Scaffold36880_959852−959927(+) mir−133d
300
250
200
150
100
50
0
0
20
40
60
80
Egg
St. 7−8
St. 12
St 18−20
St. 34−36
St. 56

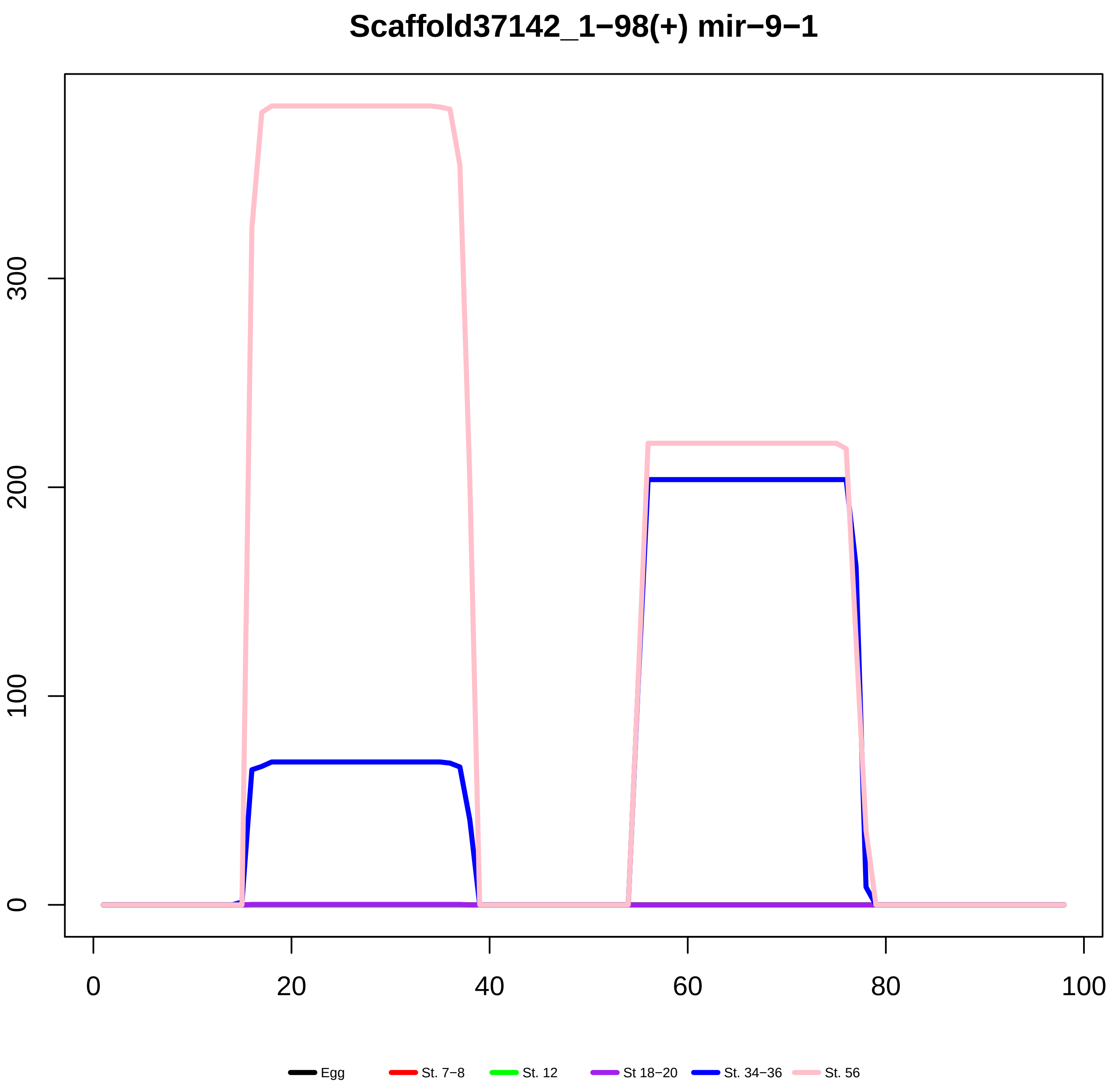

Scaffold37142_1−98(+) mir−9−1
0
100
200
300
0
20
40
60
80
100
Egg
St. 7−8
St. 12
St 18−20
St. 34−36
St. 56

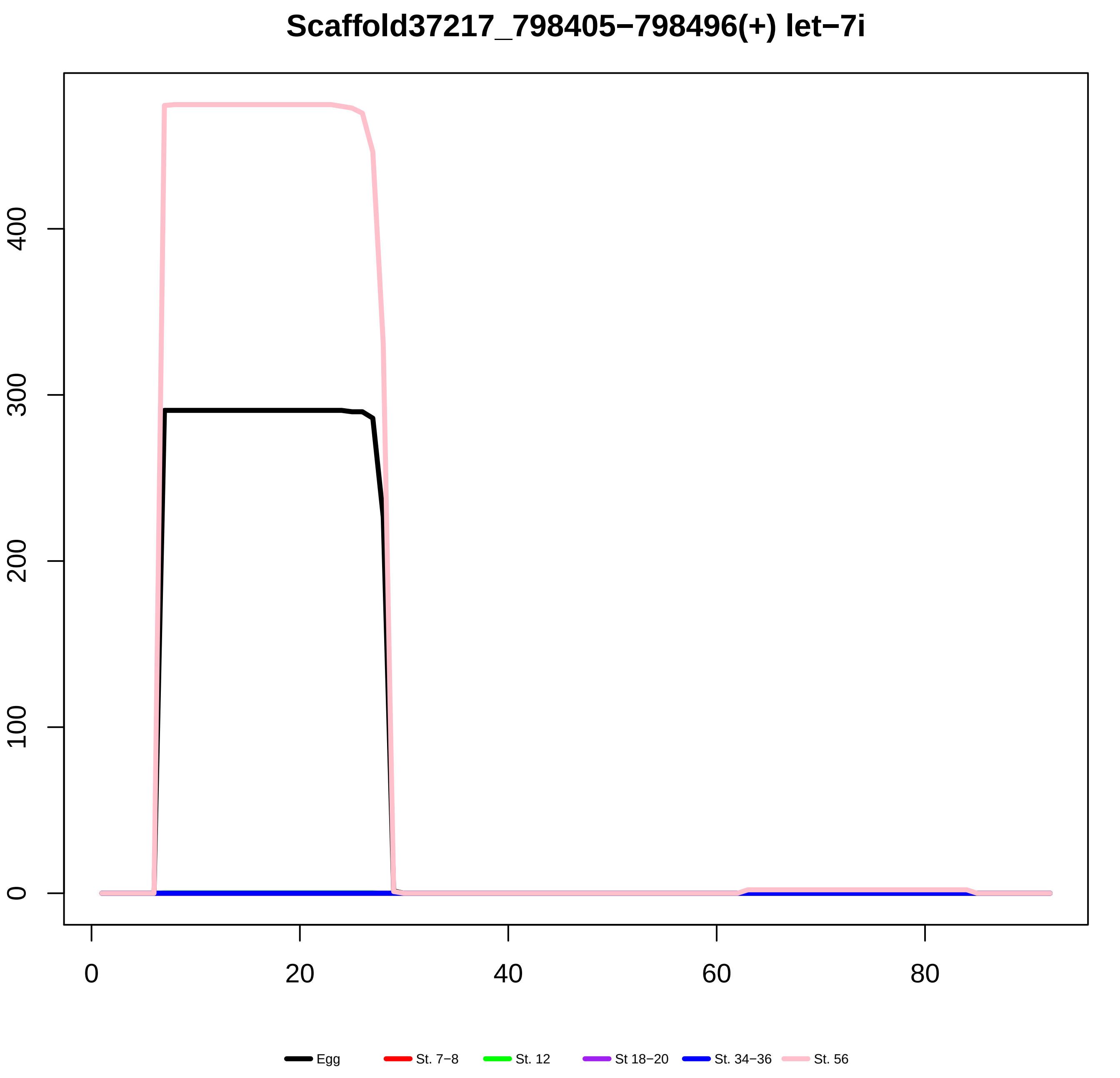
Scaffold37217_798405−798496(+) let−7i
0
100
200
300
400
0
20
40
60
80
Egg
St. 7−8
St. 12
St 18−20
St. 34−36
St. 56

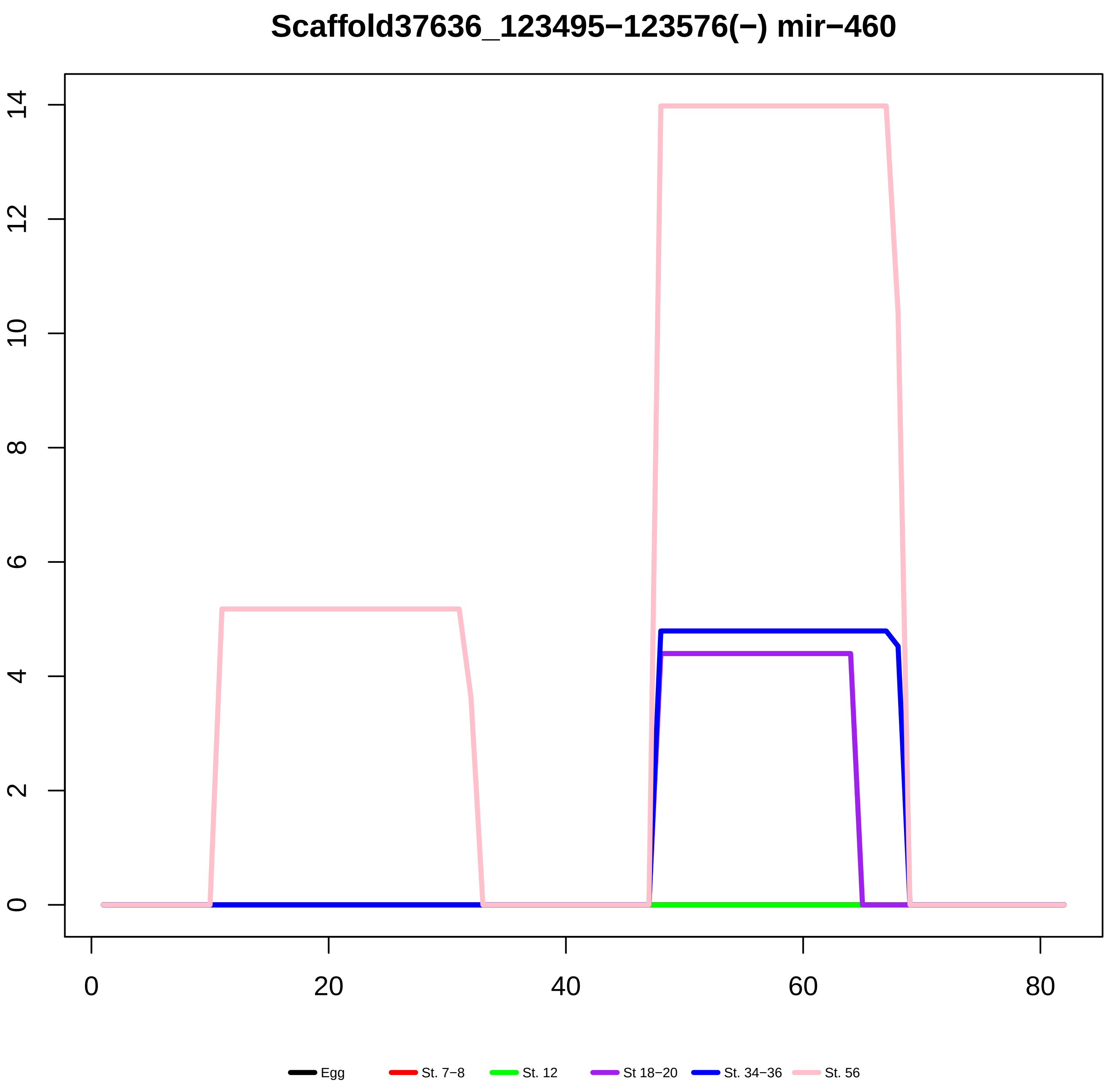

Scaffold37636_123495−123576(−) mir−460
0
2
4
6
8
10
12
14
0
20
40
60
80
Egg
St. 7−8
St. 12
St 18−20
St. 34−36
St. 56

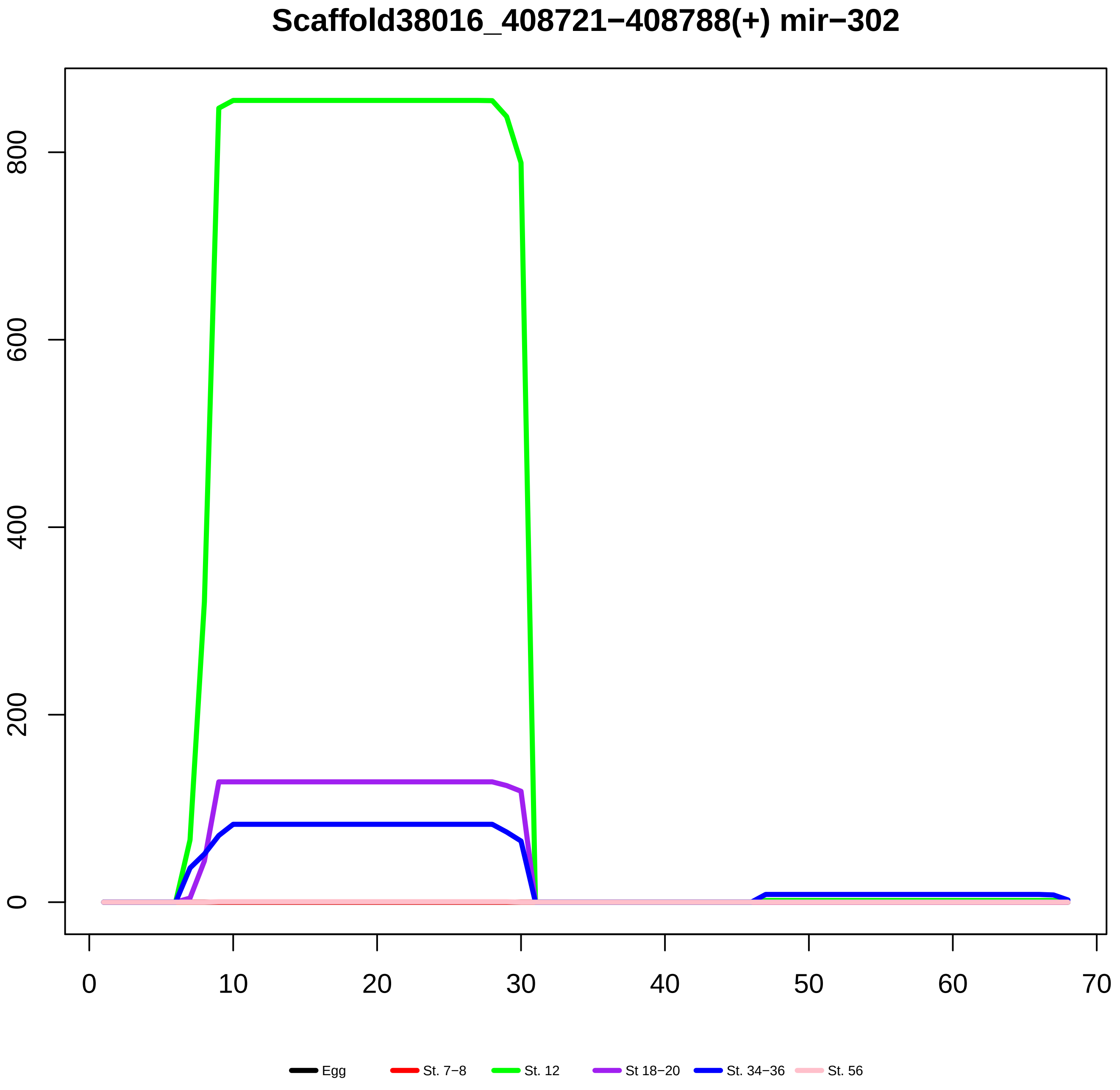

Scaffold38016_408721−408788(+) mir−302
0
200
400
600
800
0
10
20
30
40
50
60
70
Egg
St. 7−8
St. 12
St 18−20
St. 34−36
St. 56

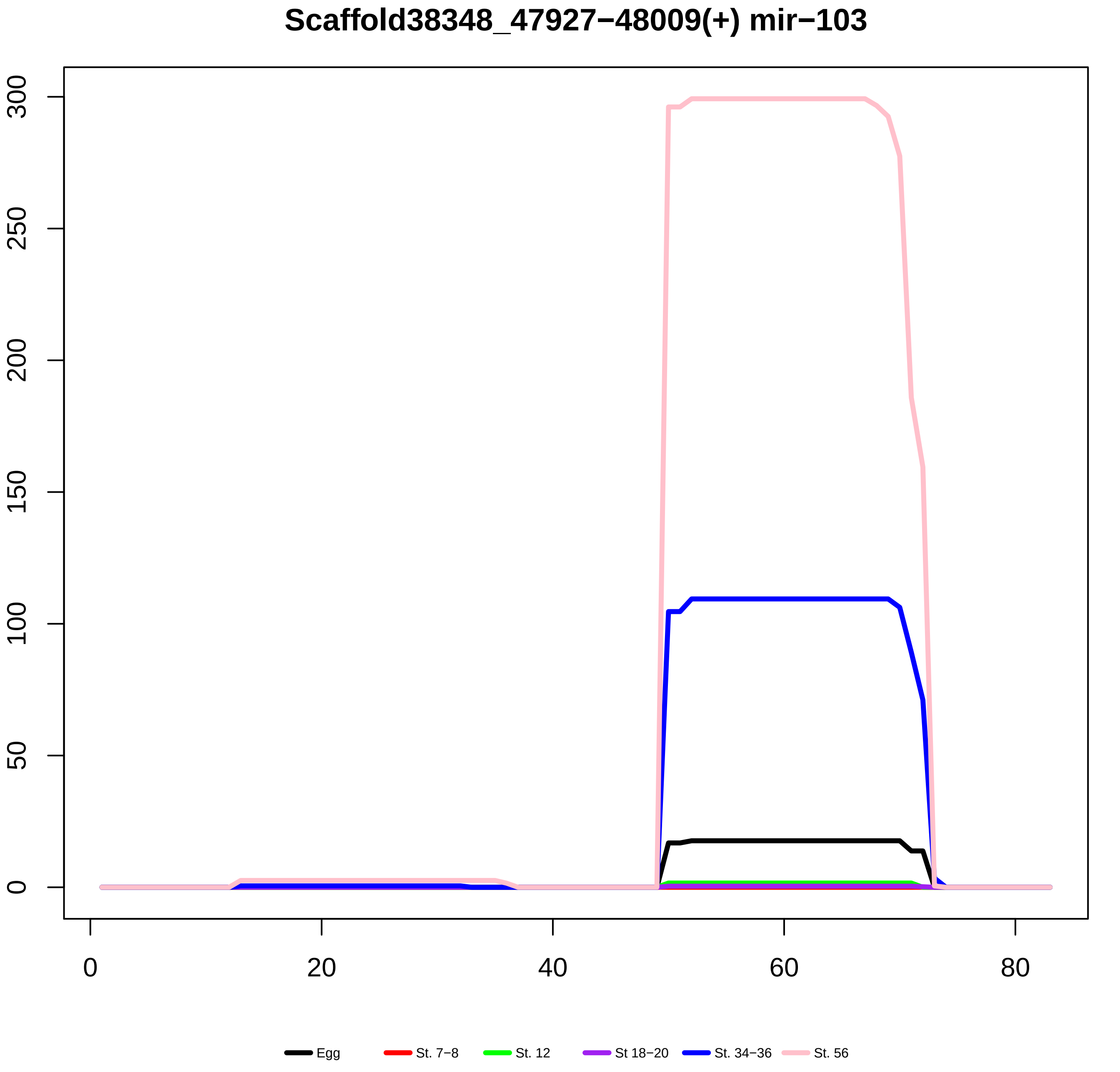
Scaffold38348_47927−48009(+) mir−103
0
50
100
150
200
250
300
0
20
40
60
80
Egg
St. 7−8
St. 12
St 18−20
St. 34−36
St. 56

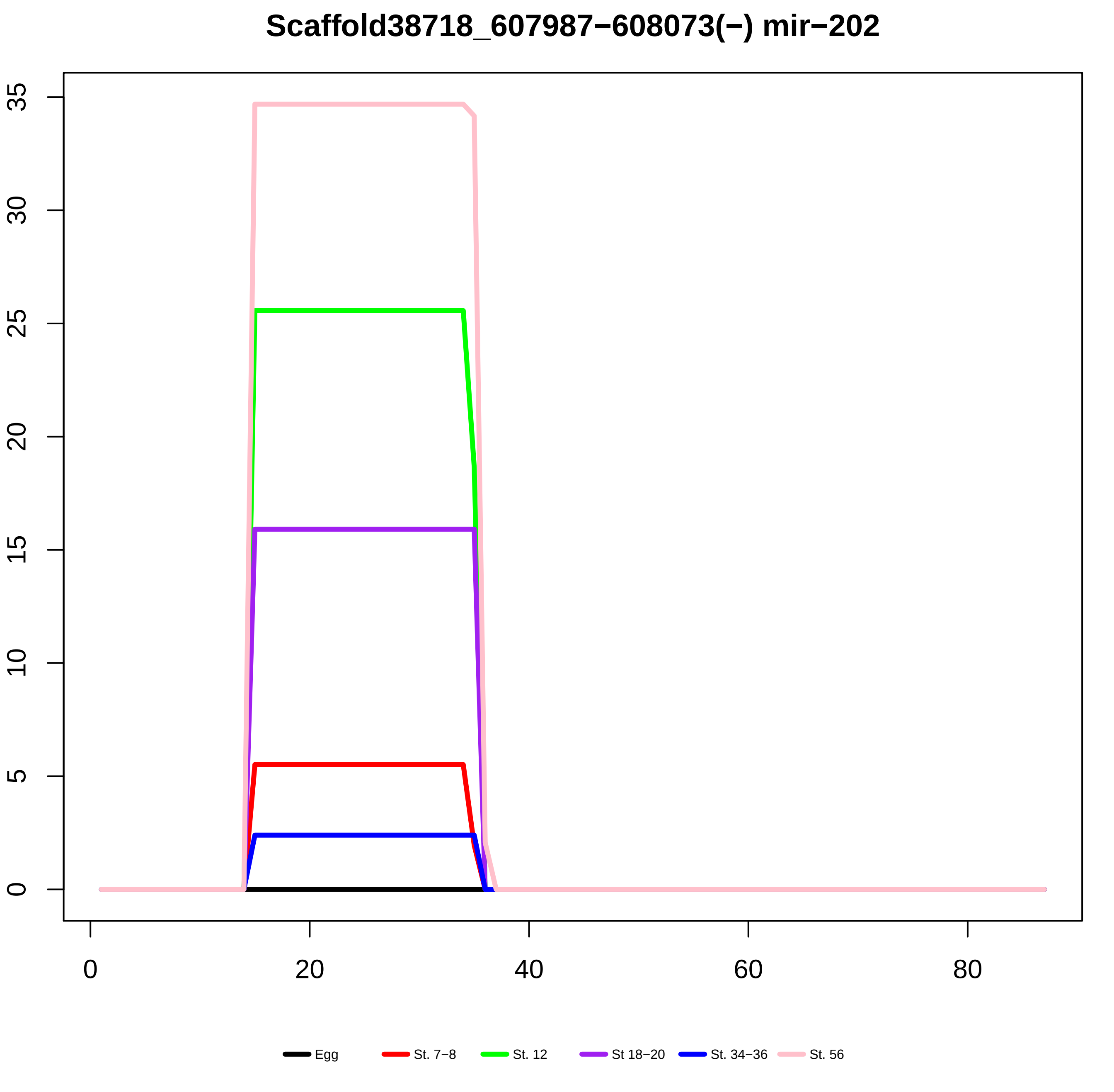

Scaffold38718_607987−608073(−) mir−202
0
5
10
15
20
25
30
35
0
20
40
60
80
Egg
St. 7−8
St. 12
St 18−20
St. 34−36
St. 56

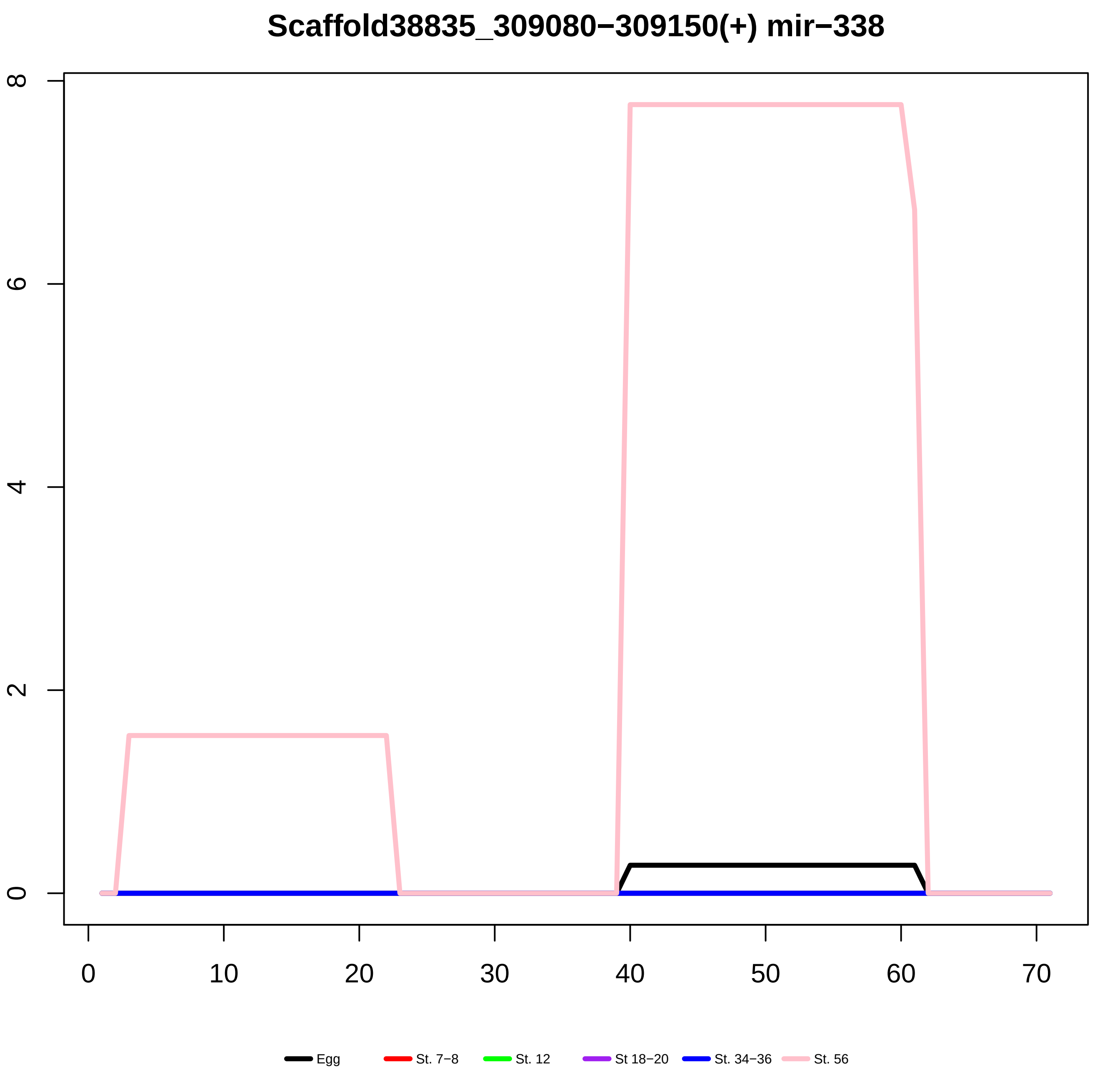

Scaffold38835_309080−309150(+) mir−338
0
2
4
6
8
0
10
20
30
40
50
60
70
Egg
St. 7−8
St. 12
St 18−20
St. 34−36
St. 56

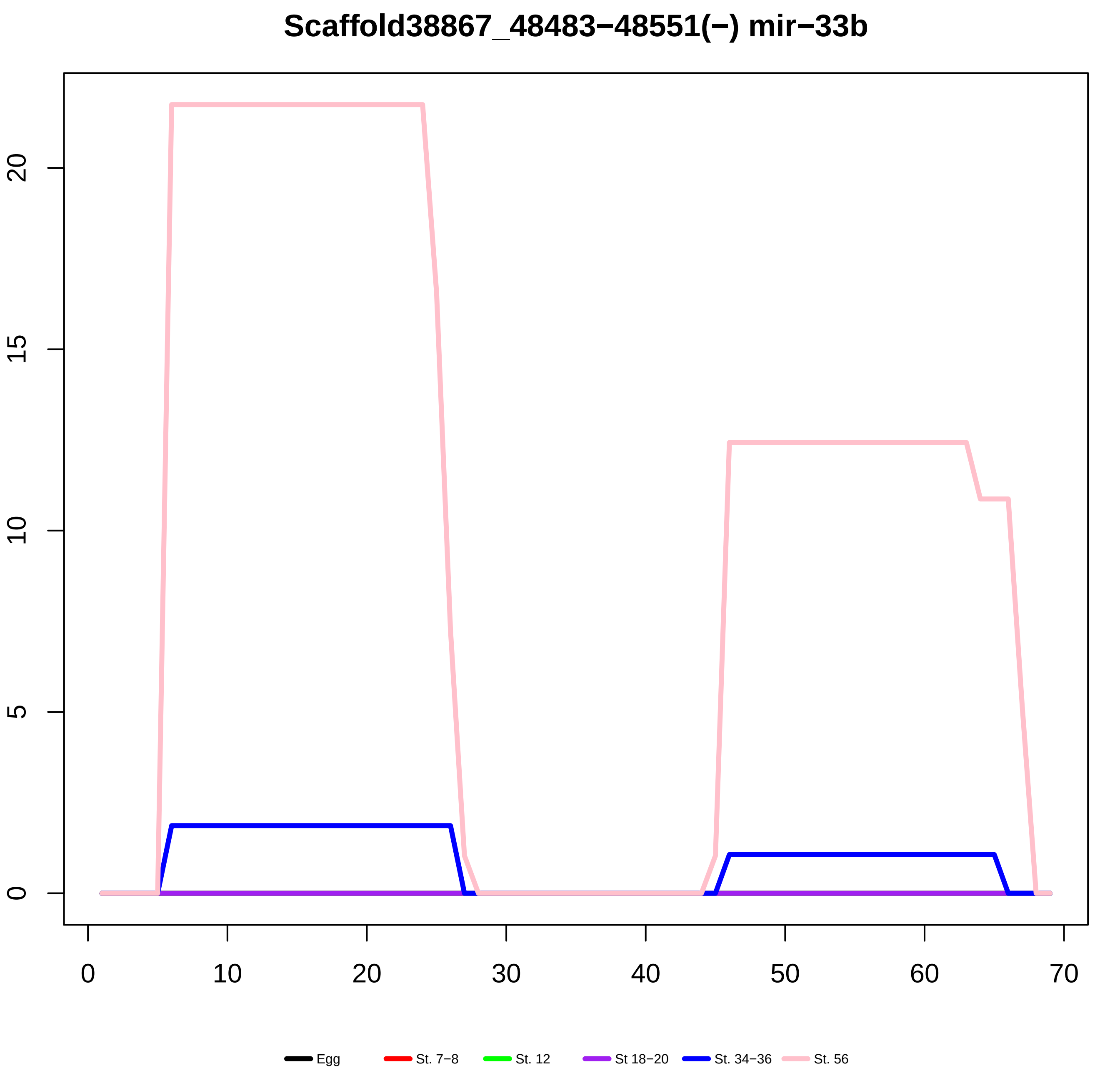
Scaffold38867_48483−48551(−) mir−33b
0
5
10
15
20
0
10
20
30
40
50
60
70
Egg
St. 7−8
St. 12
St 18−20
St. 34−36
St. 56

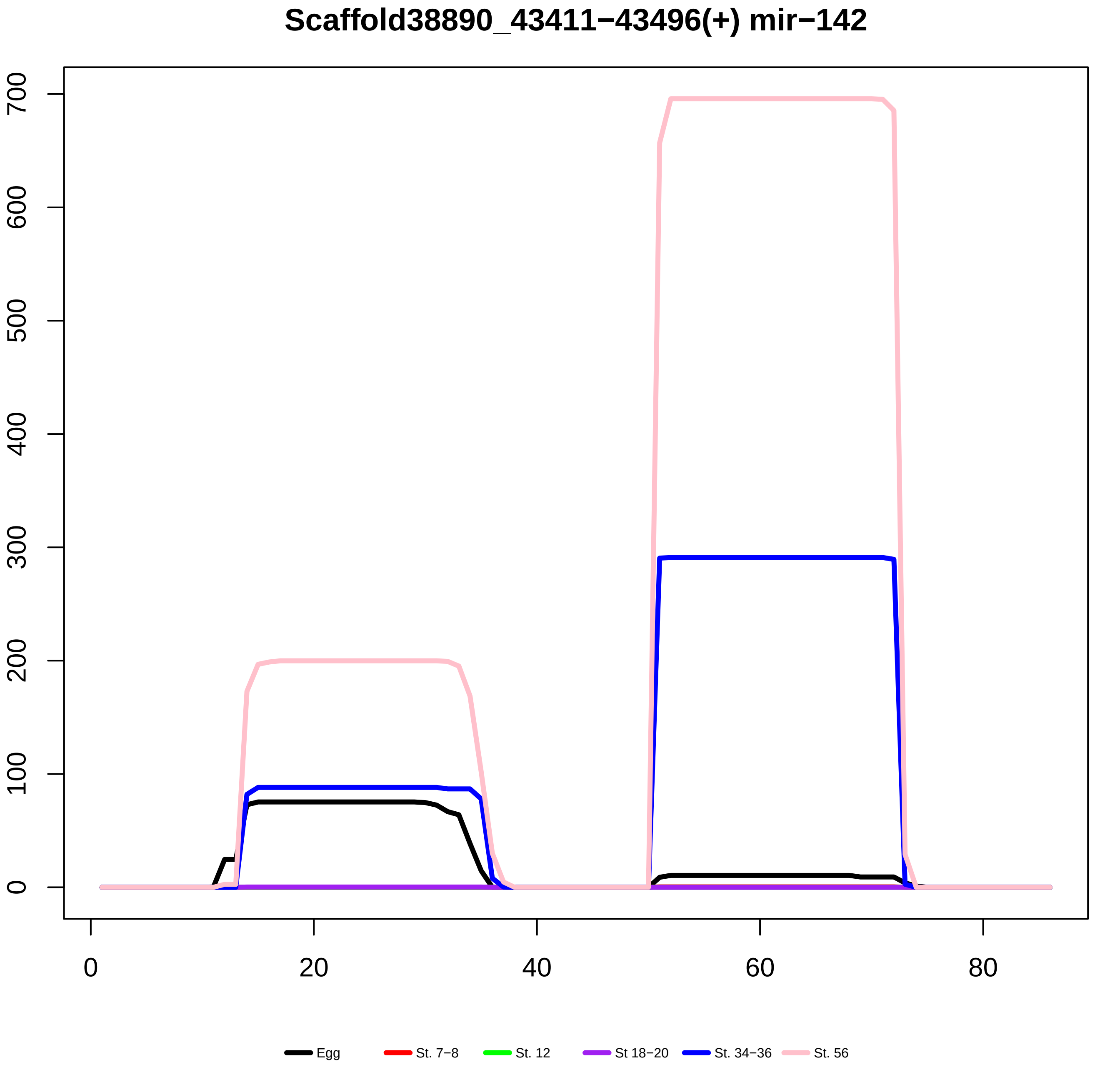

Scaffold38890_43411−43496(+) mir−142
0
100
200
300
400
500
600
700
0
20
40
60
80
Egg
St. 7−8
St. 12
St 18−20
St. 34−36
St. 56

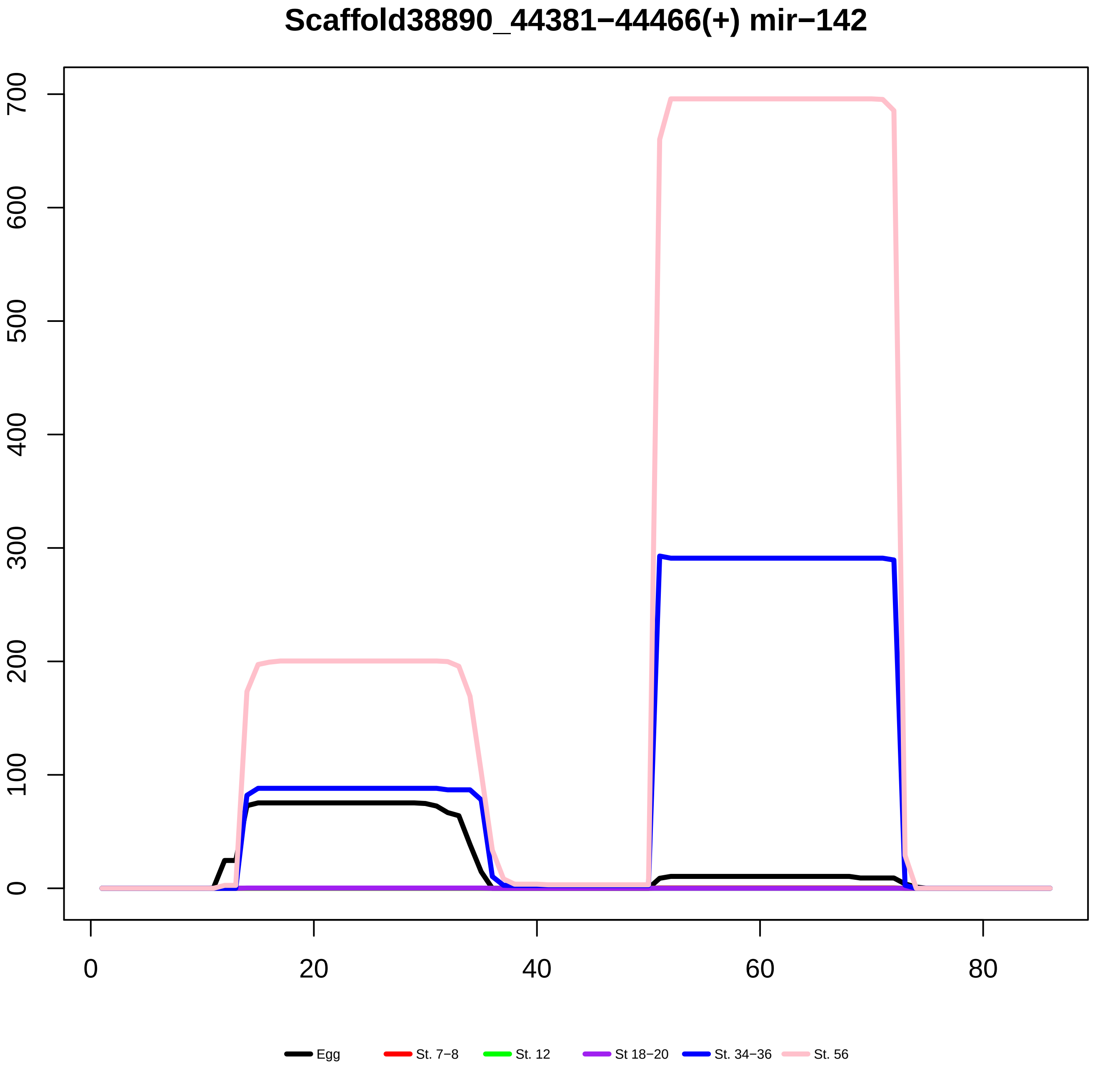
Scaffold38890_44381−44466(+) mir−142
0
100
200
300
400
500
600
700
0
20
40
60
80
Egg
St. 7−8
St. 12
St 18−20
St. 34−36
St. 56

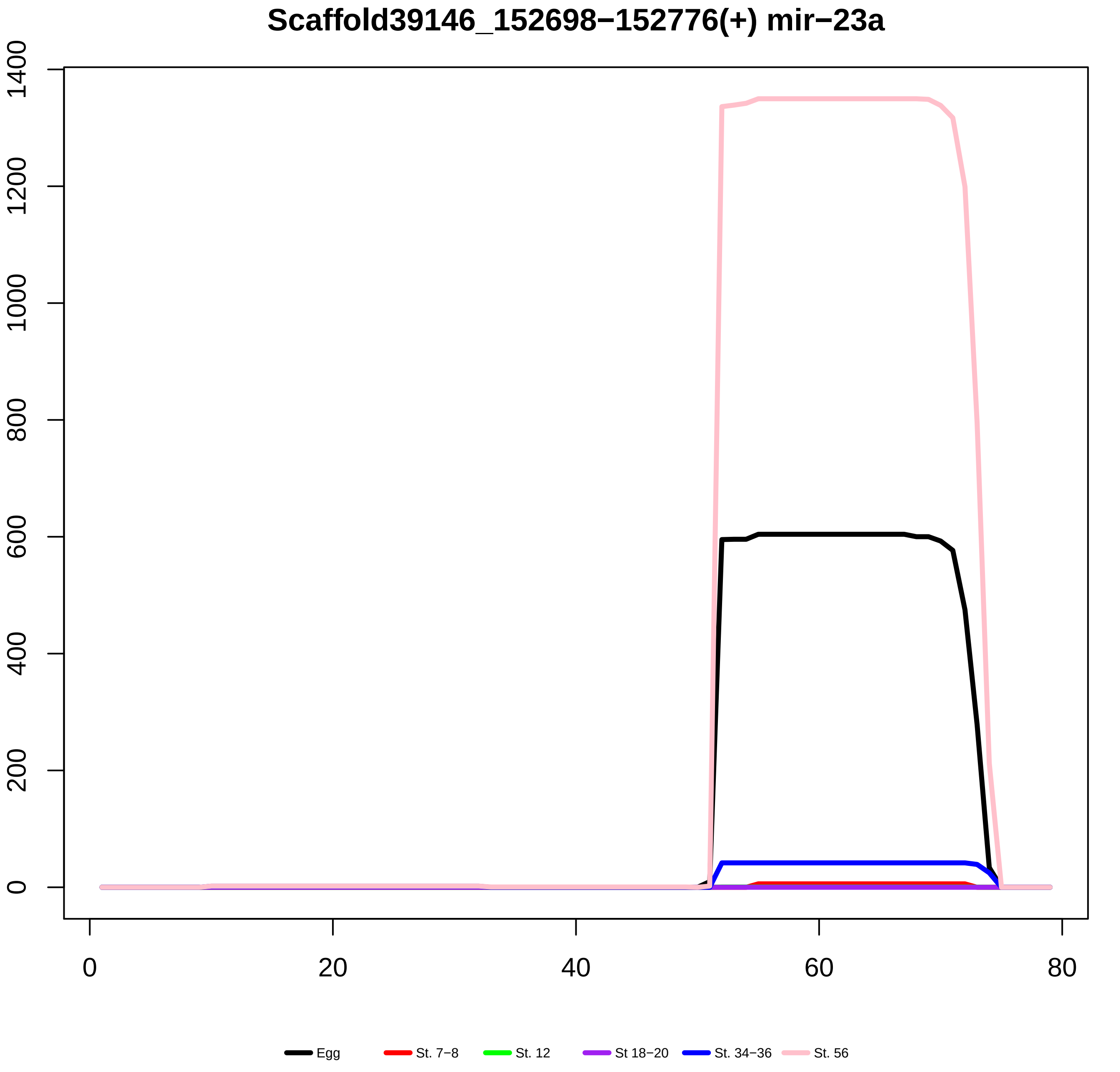

Scaffold39146_152698−152776(+) mir−23a
0
200
400
600
800
1000
1200
1400
0
20
40
60
80
Egg
St. 7−8
St. 12
St 18−20
St. 34−36
St. 56

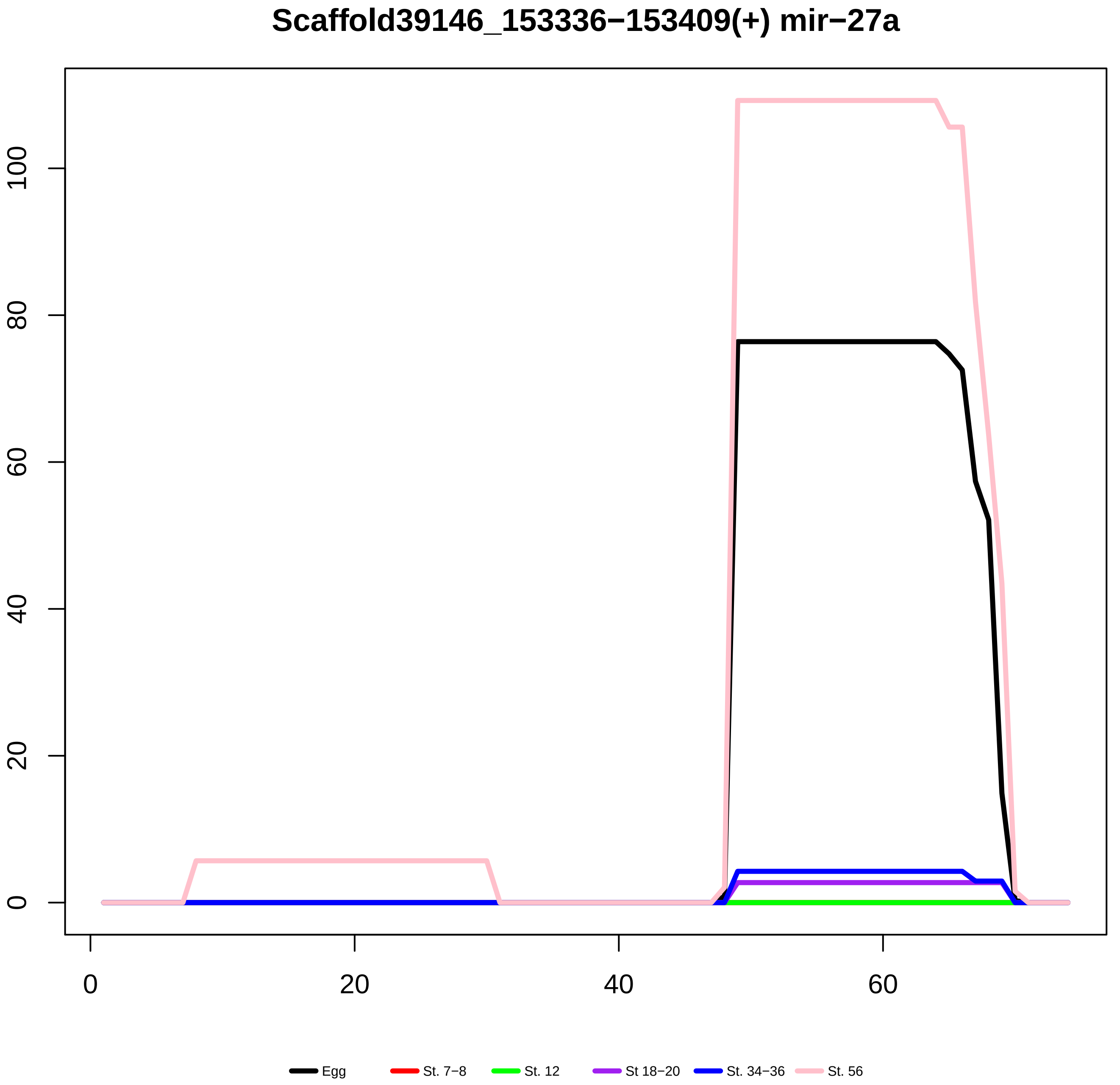

Scaffold39146_153336−153409(+) mir−27a
0
20
40
60
80
100
0
20
40
60
Egg
St. 7−8
St. 12
St 18−20
St. 34−36
St. 56

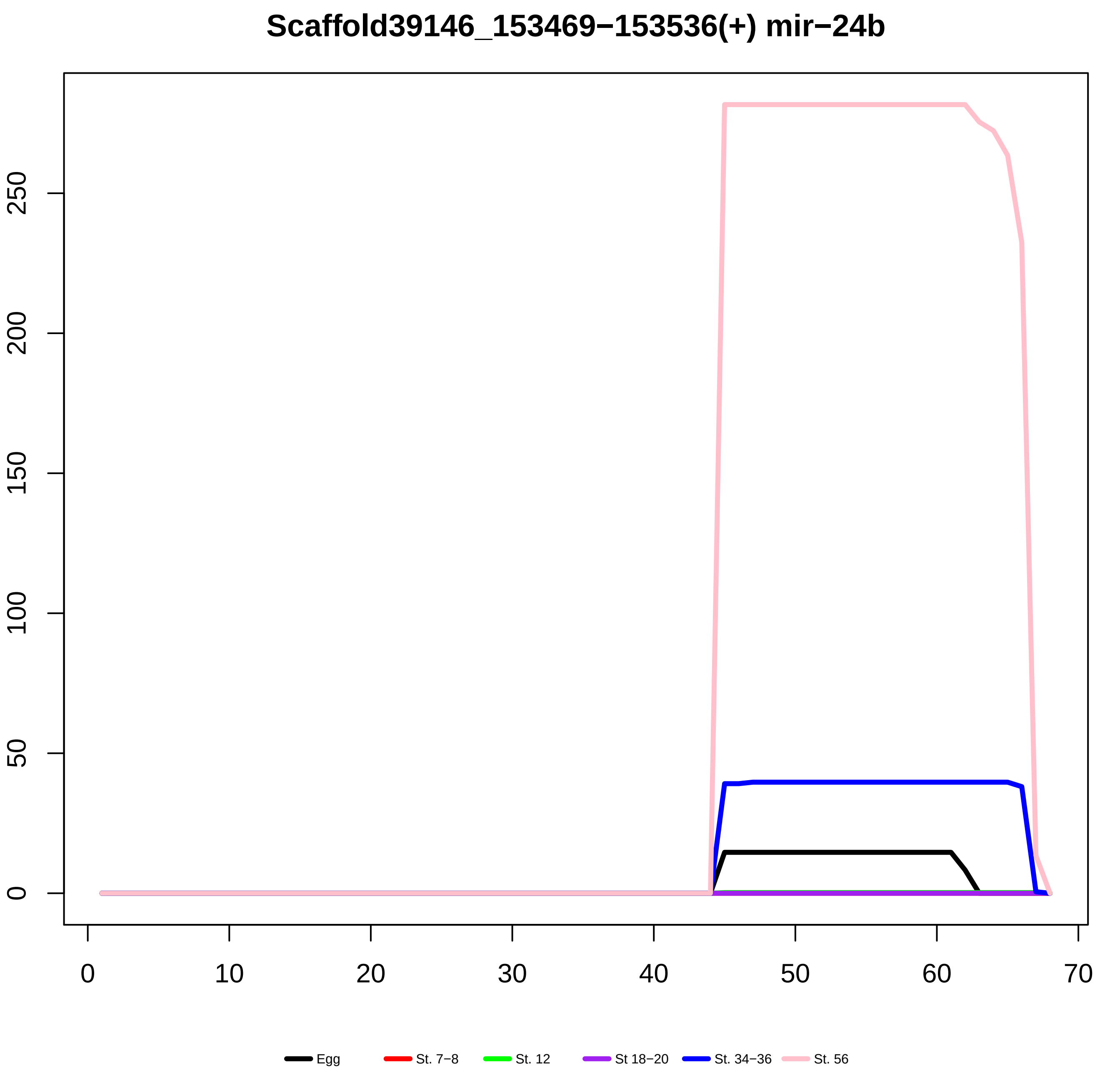
Scaffold39146_153469−153536(+) mir−24b
0
50
100
150
200
250
0
10
20
30
40
50
60
70
Egg
St. 7−8
St. 12
St 18−20
St. 34−36
St. 56

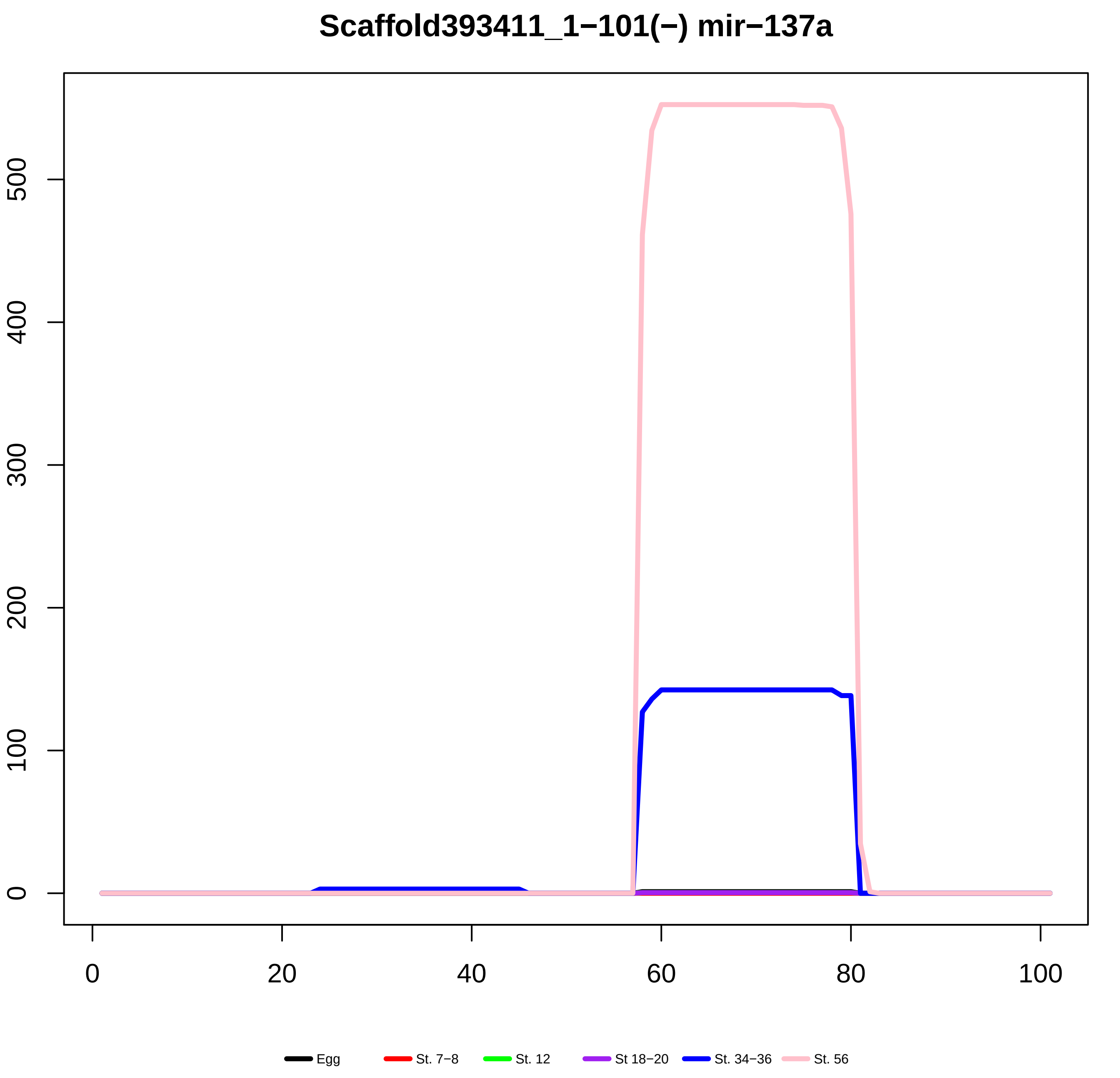

Scaffold393411_1−101(−) mir−137a
0
100
200
300
400
500
0
20
40
60
80
100
Egg
St. 7−8
St. 12
St 18−20
St. 34−36
St. 56

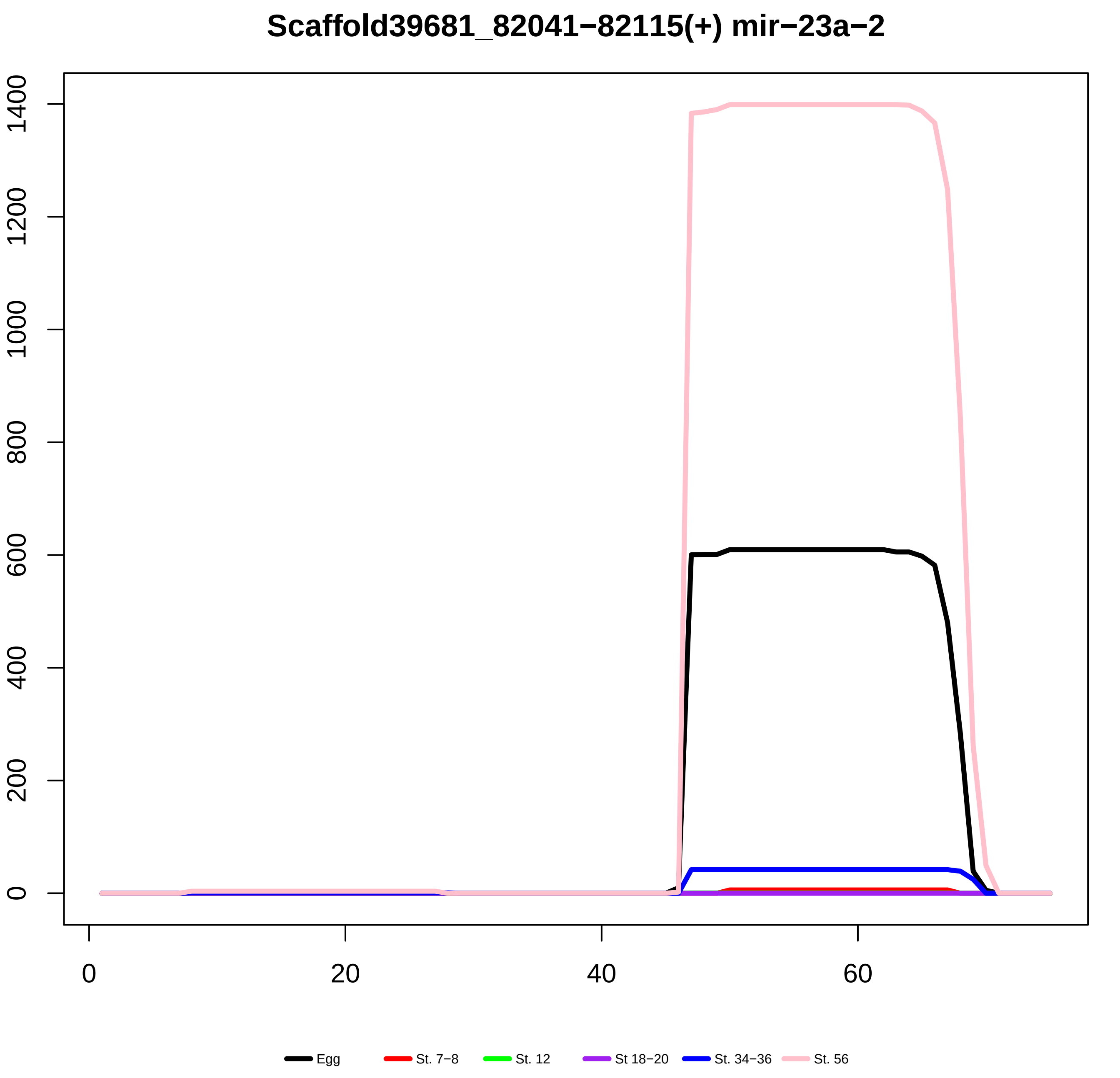

Scaffold39681_82041−82115(+) mir−23a−2
0
200
400
600
800
1000
1200
1400
0
20
40
60
Egg
St. 7−8
St. 12
St 18−20
St. 34−36
St. 56

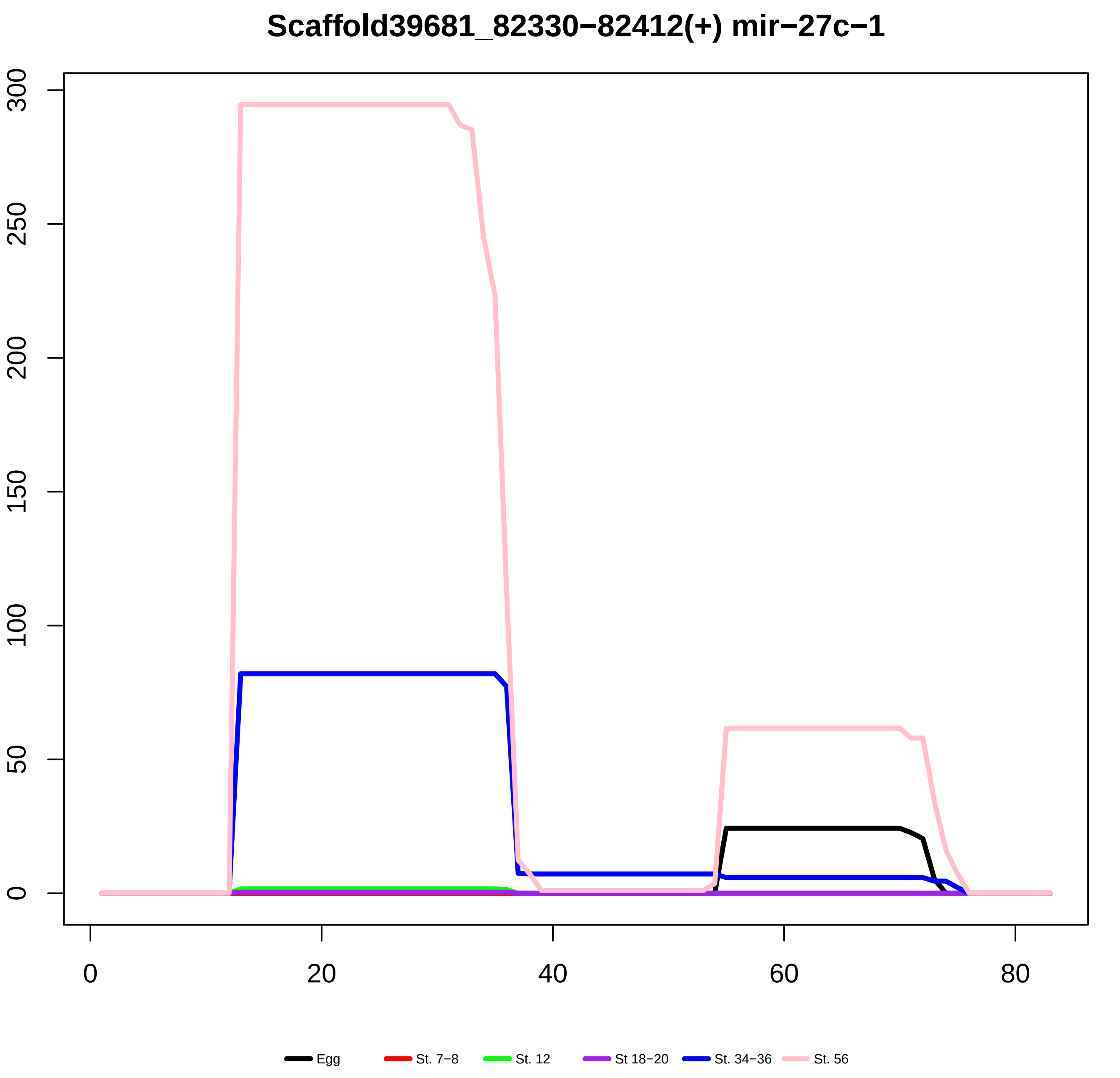

Scaffold39681_82330−82412(+) mir−27c−1
0
50
100
150
200
250
300
0
20
40
60
80
Egg
St. 7−8
St. 12
St 18−20
St. 34−36
St. 56

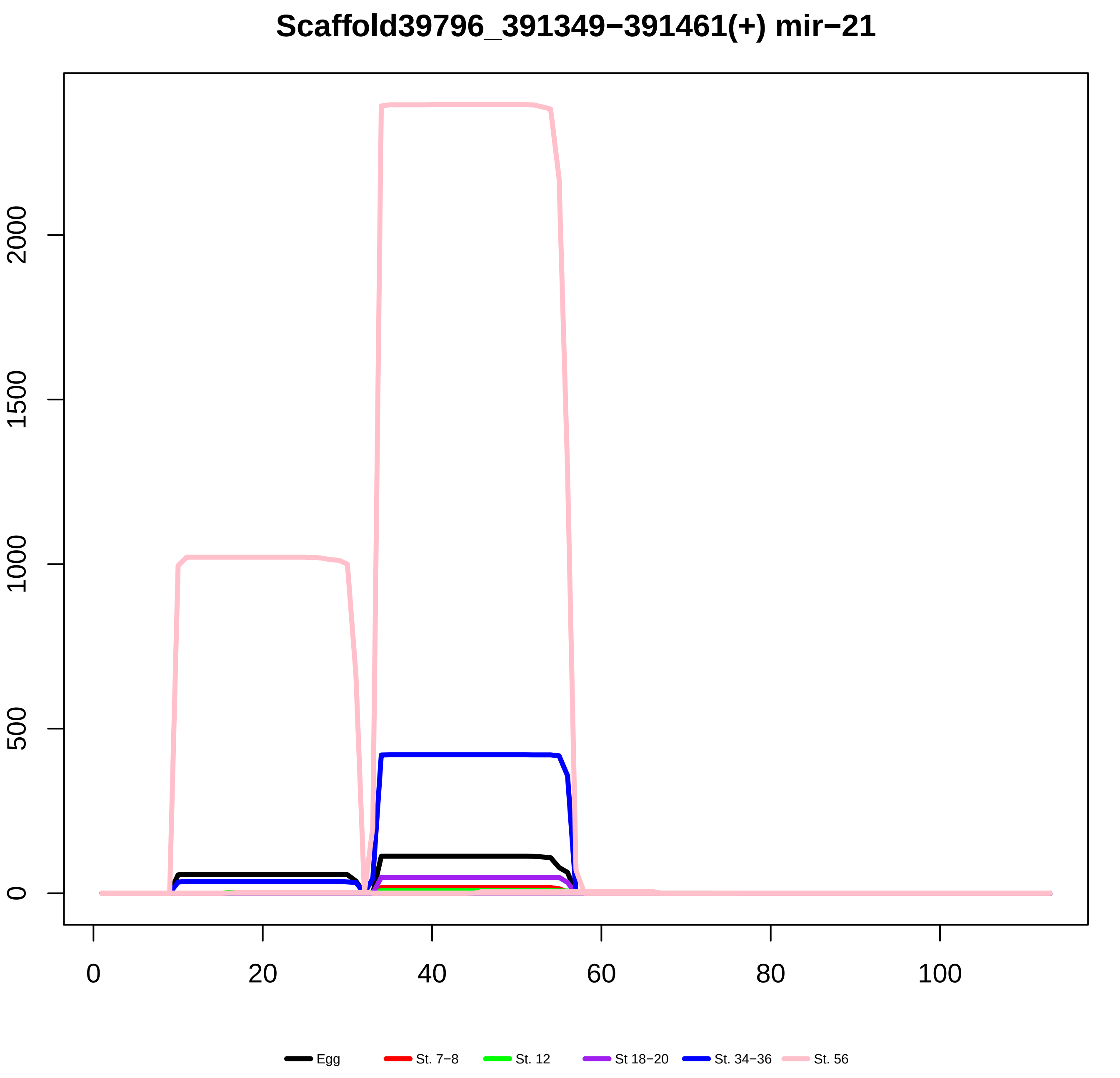
Scaffold39796_391349−391461(+) mir−21
0
500
1000
1500
2000
0
20
40
60
80
100
Egg
St. 7−8
St. 12
St 18−20
St. 34−36
St. 56

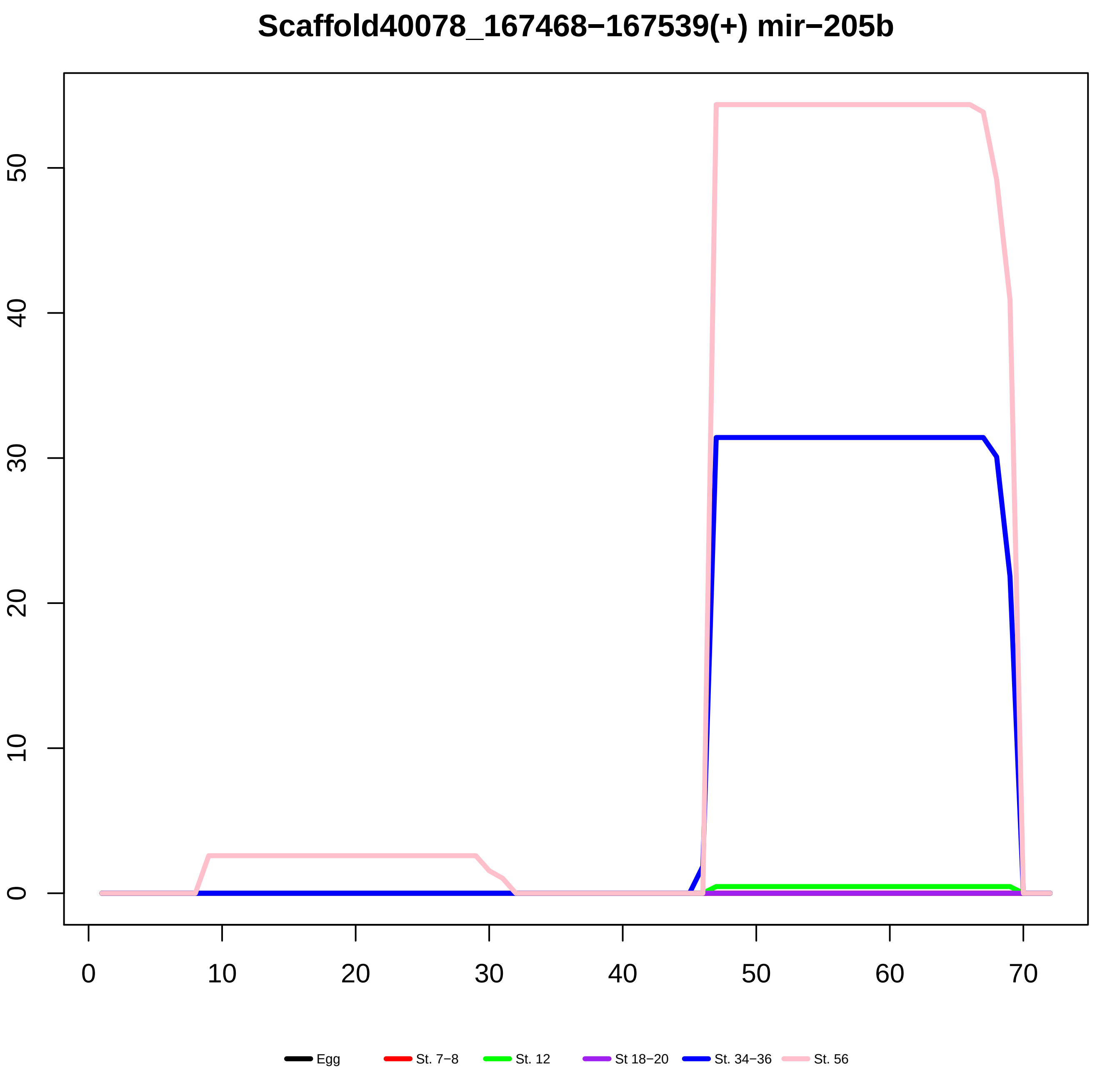
Scaffold40078_167468−167539(+) mir−205b
0
10
20
30
40
50
0
10
20
30
40
50
60
70
Egg
St. 7−8
St. 12
St 18−20
St. 34−36
St. 56

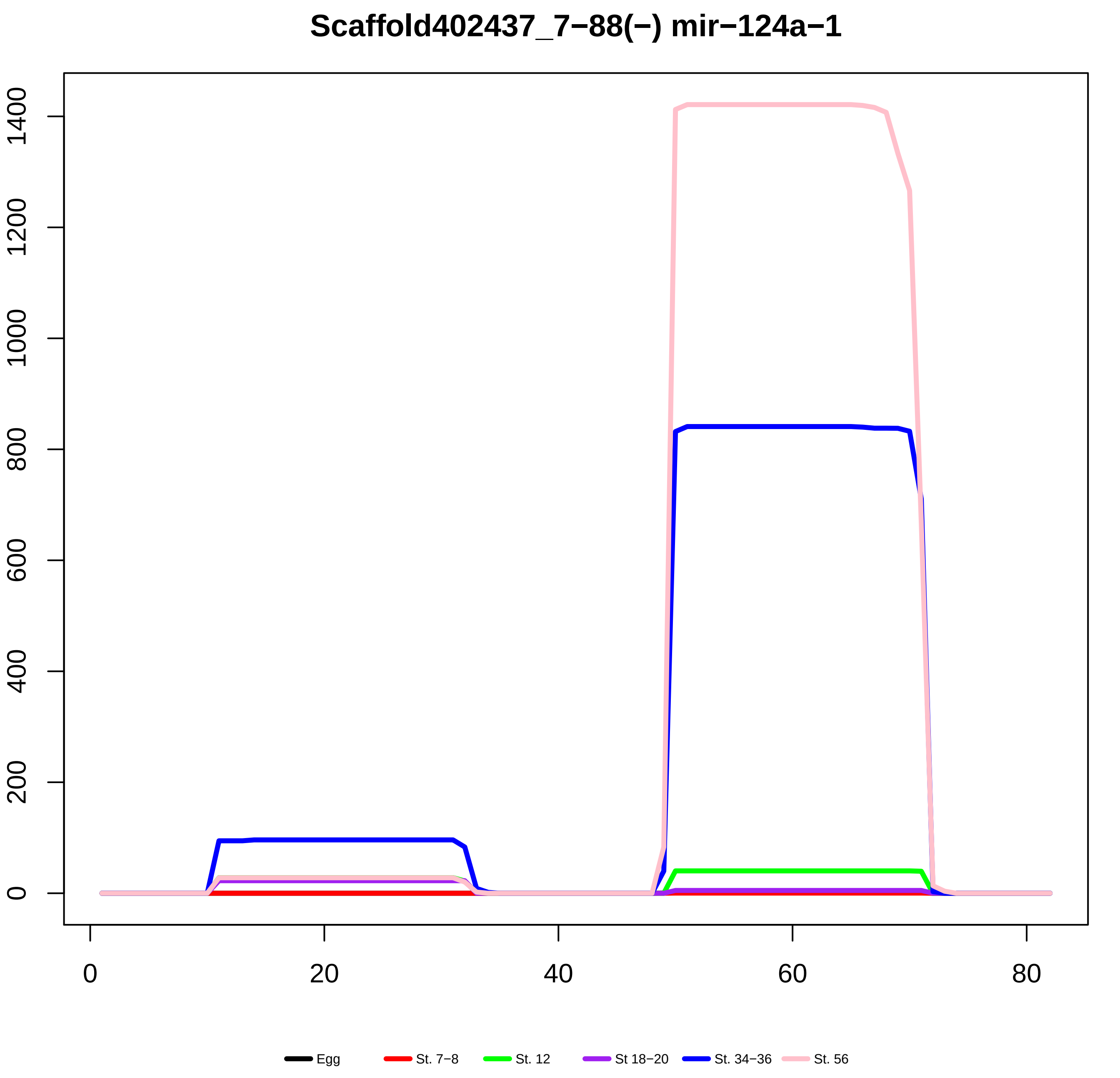

Scaffold402437_7−88(−) mir−124a−1
0
200
400
600
800
1000
1200
1400
0
20
40
60
80
Egg
St. 7−8
St. 12
St 18−20
St. 34−36
St. 56

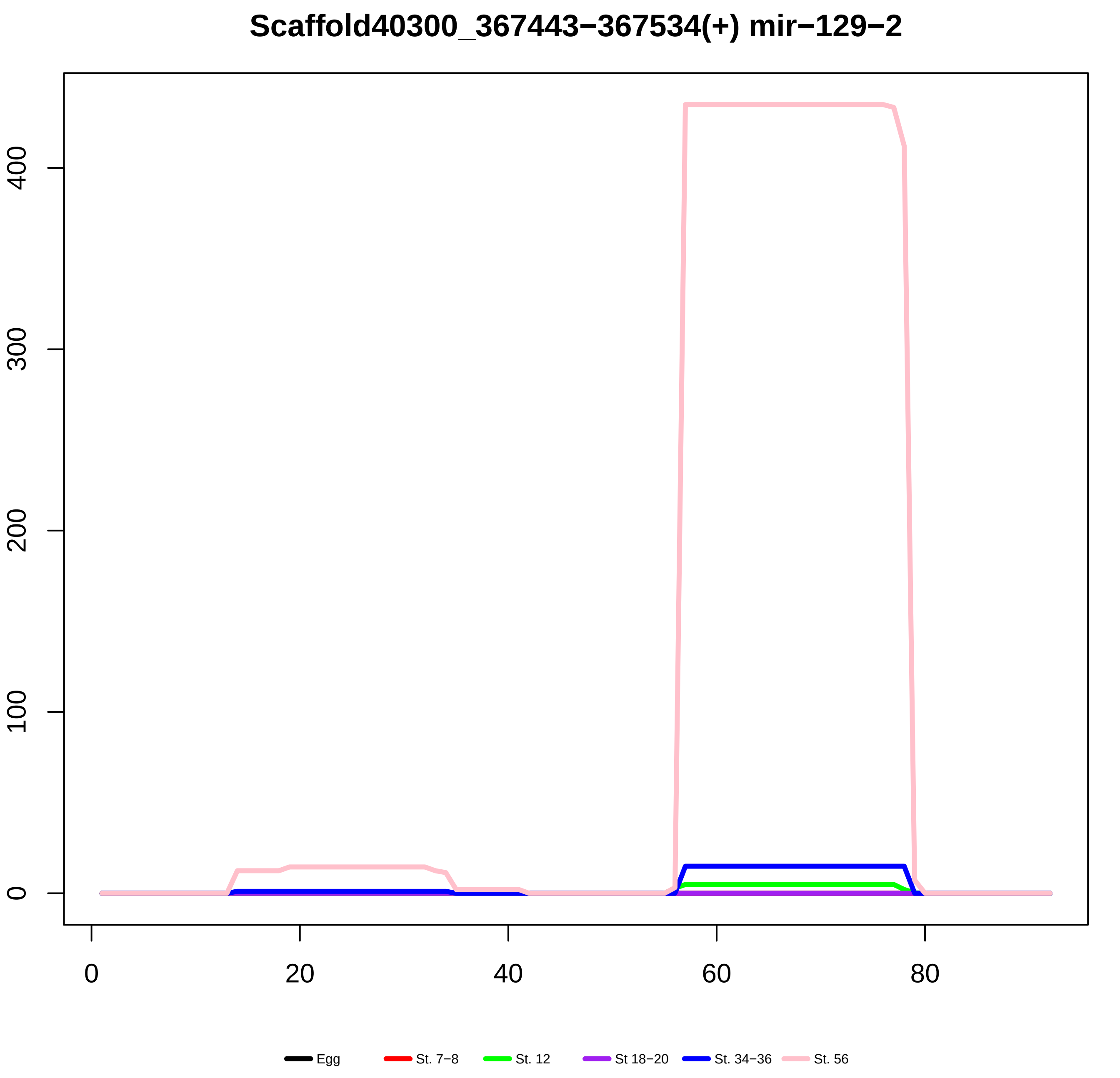

Scaffold40300_367443−367534(+) mir−129−2
0
100
200
300
400
0
20
40
60
80
Egg
St. 7−8
St. 12
St 18−20
St. 34−36
St. 56

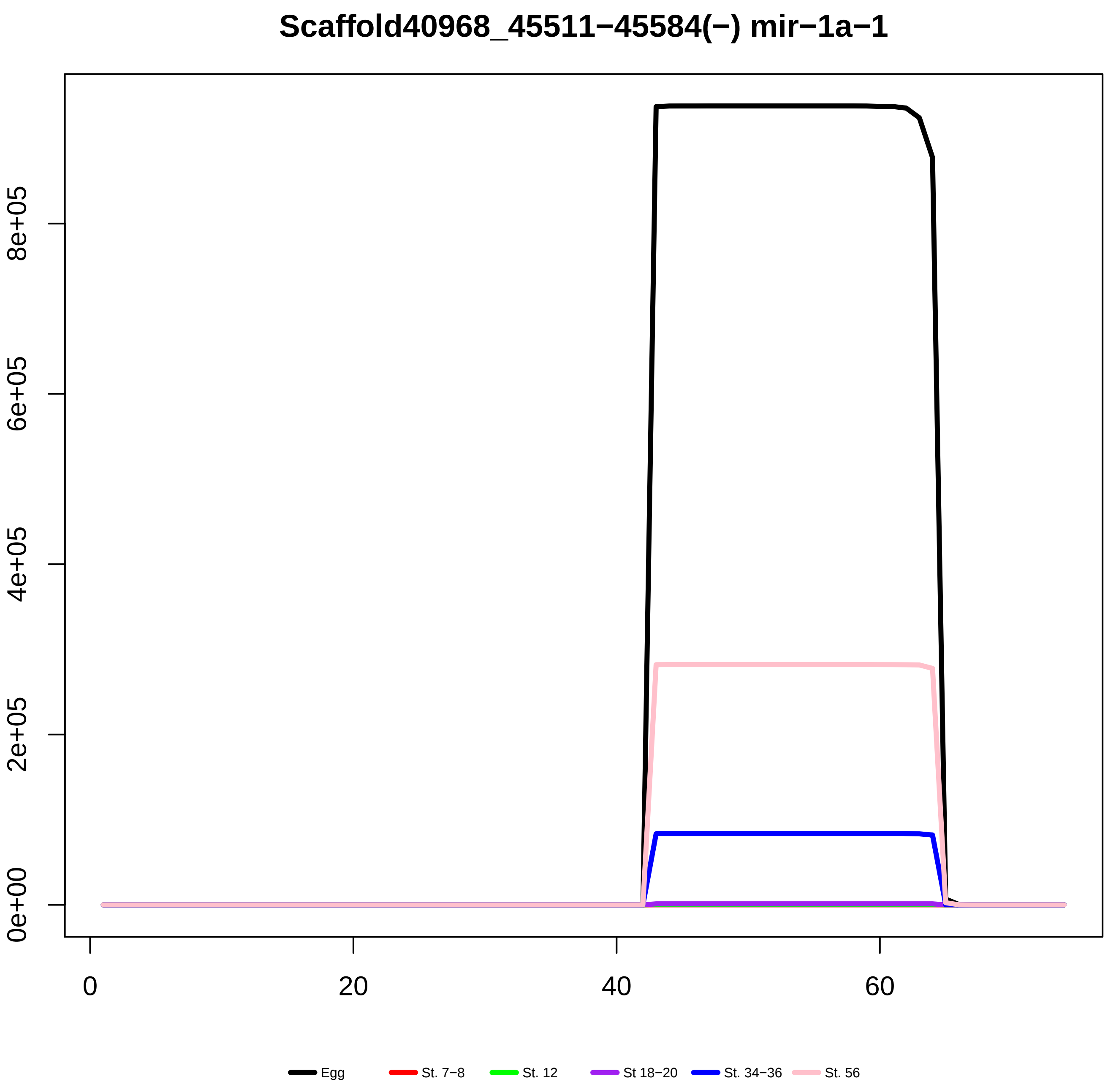

Scaffold40968_45511−45584(−) mir−1a−1
0e+00
2e+05
4e+05
6e+05
8e+05
0
20
40
60
Egg
St. 7−8
St. 12
St 18−20
St. 34−36
St. 56

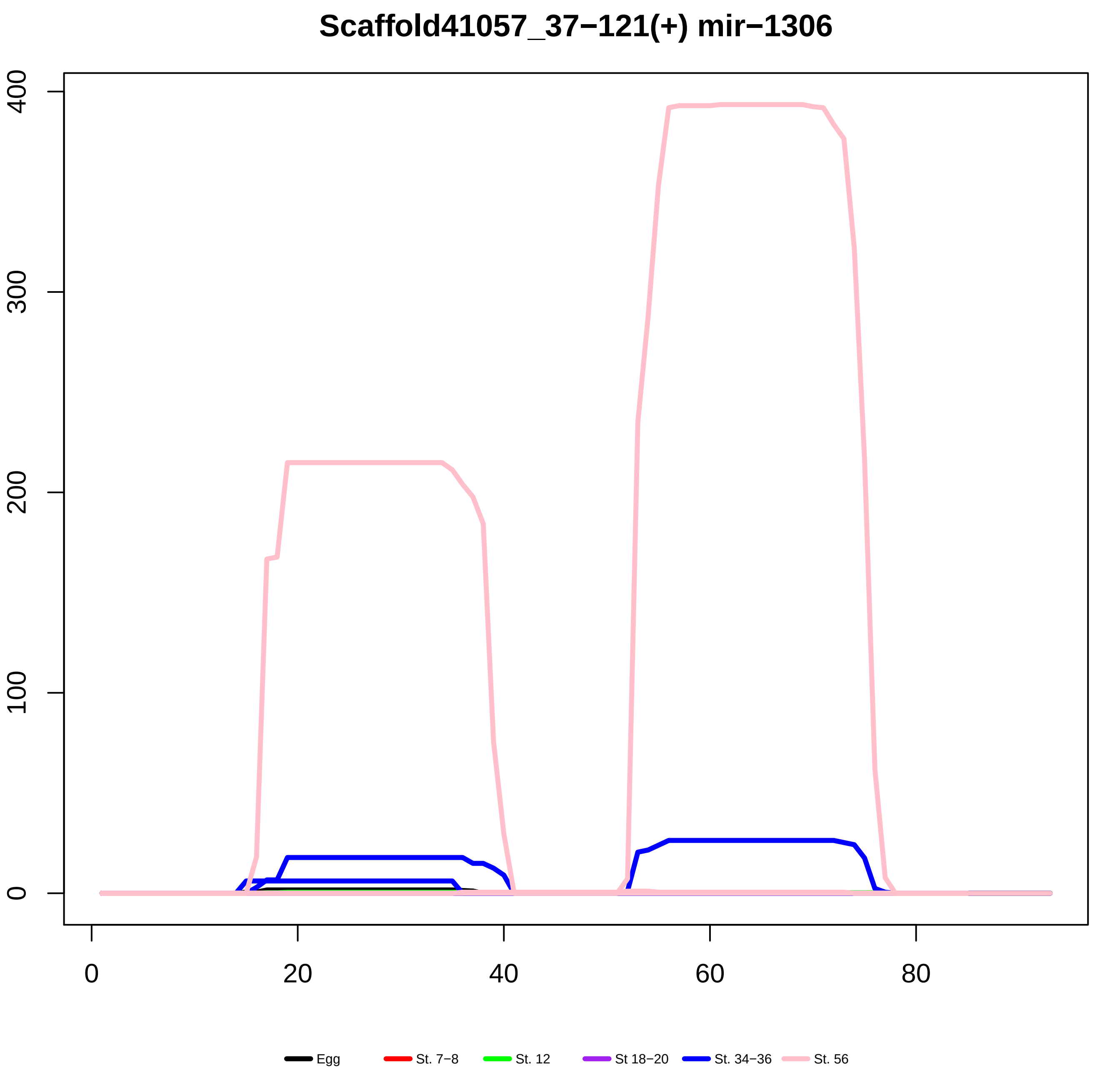

Scaffold41057_37−121(+) mir−1306
0
100
200
300
400
0
20
40
60
80
Egg
St. 7−8
St. 12
St 18−20
St. 34−36
St. 56

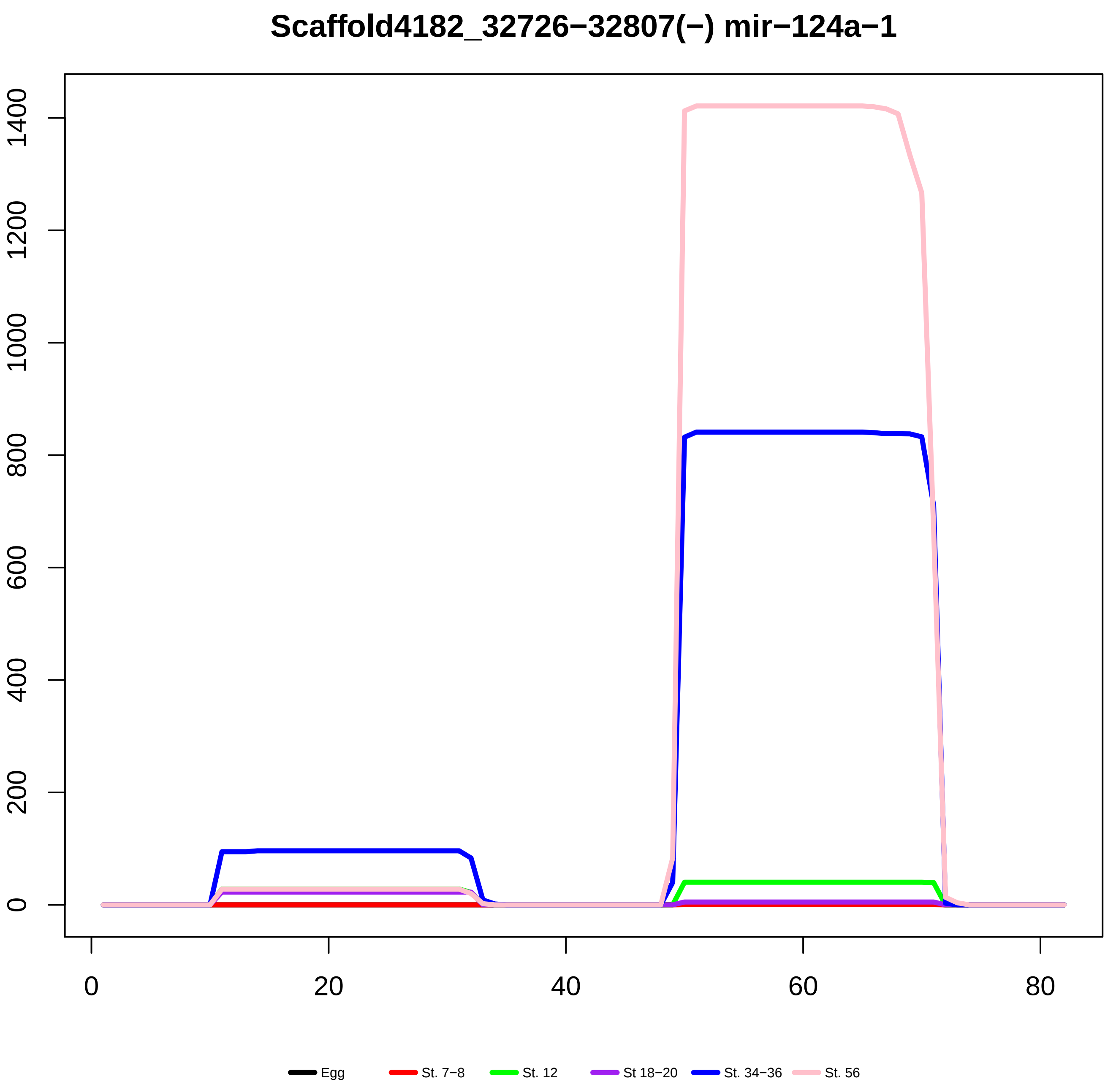

Scaffold4182_32726−32807(−) mir−124a−1
0
200
400
600
800
1000
1200
1400
0
20
40
60
80
Egg
St. 7−8
St. 12
St 18−20
St. 34−36
St. 56

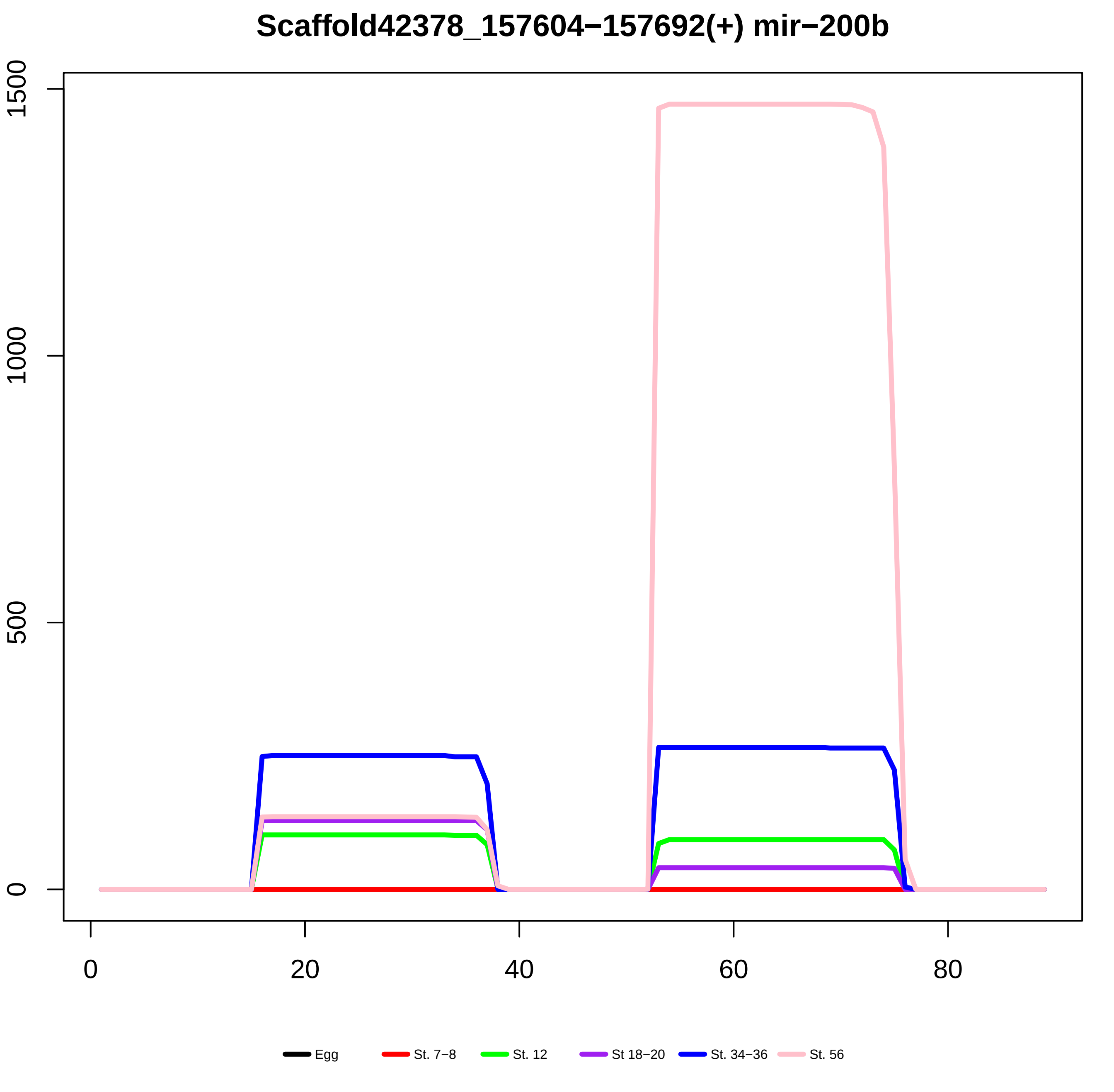

Scaffold42378_157604−157692(+) mir−200b
0
500
1000
1500
0
20
40
60
80
Egg
St. 7−8
St. 12
St 18−20
St. 34−36
St. 56

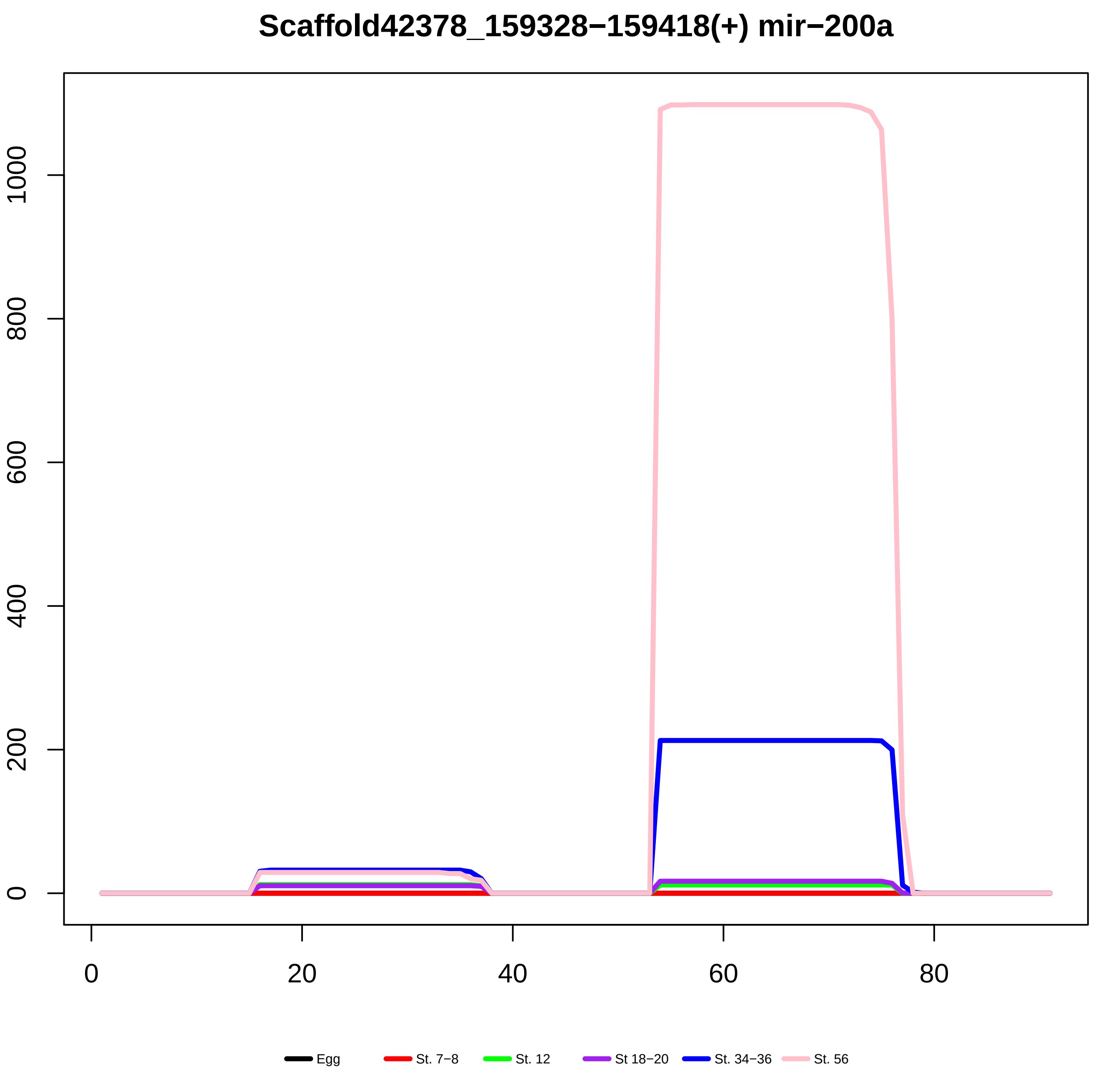
Scaffold42378_159328−159418(+) mir−200a
0
200
400
600
800
1000
0
20
40
60
80
Egg
St. 7−8
St. 12
St 18−20
St. 34−36
St. 56

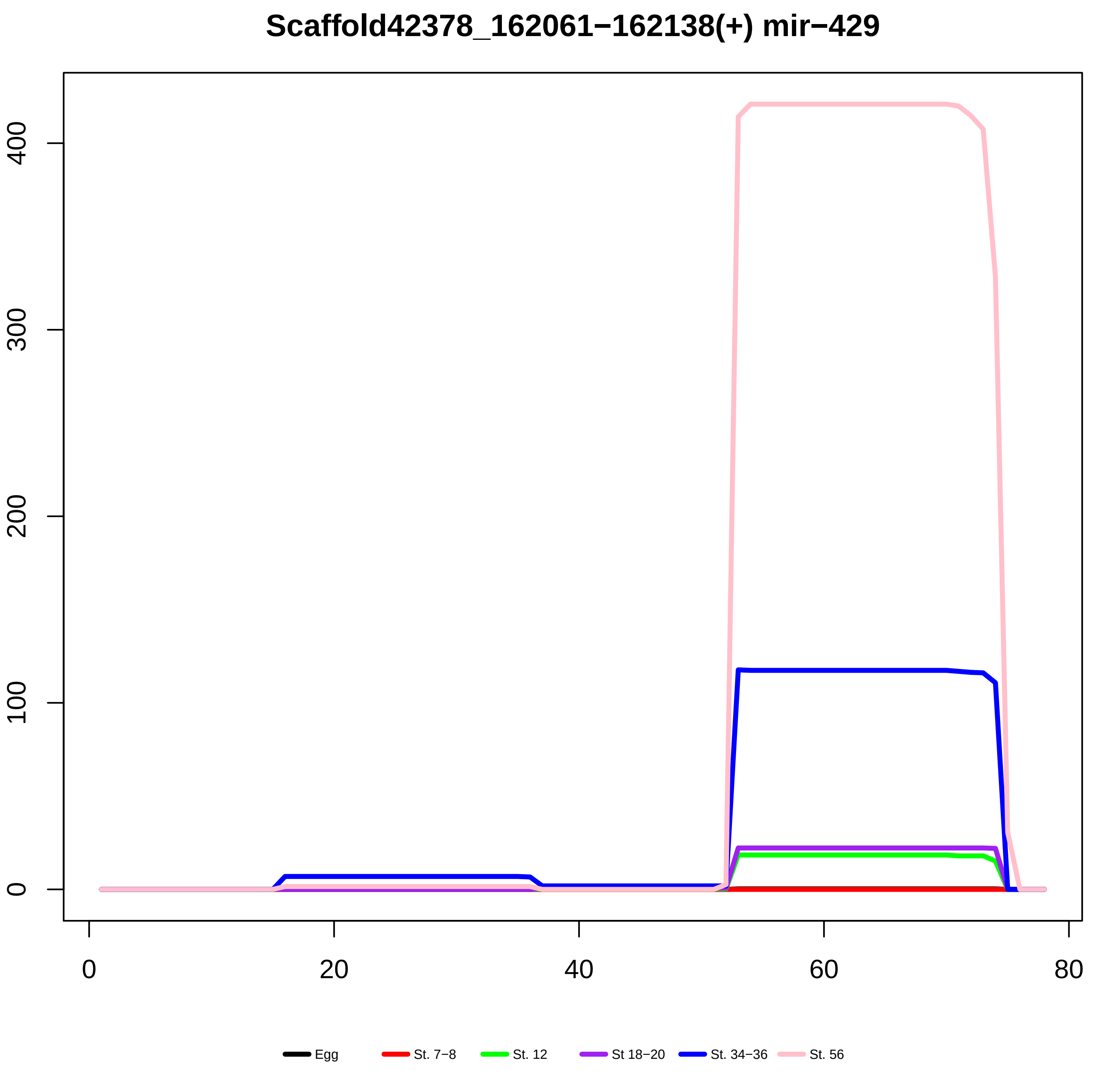

Scaffold42378_162061−162138(+) mir−429
0
100
200
300
400
0
20
40
60
80
Egg
St. 7−8
St. 12
St 18−20
St. 34−36
St. 56

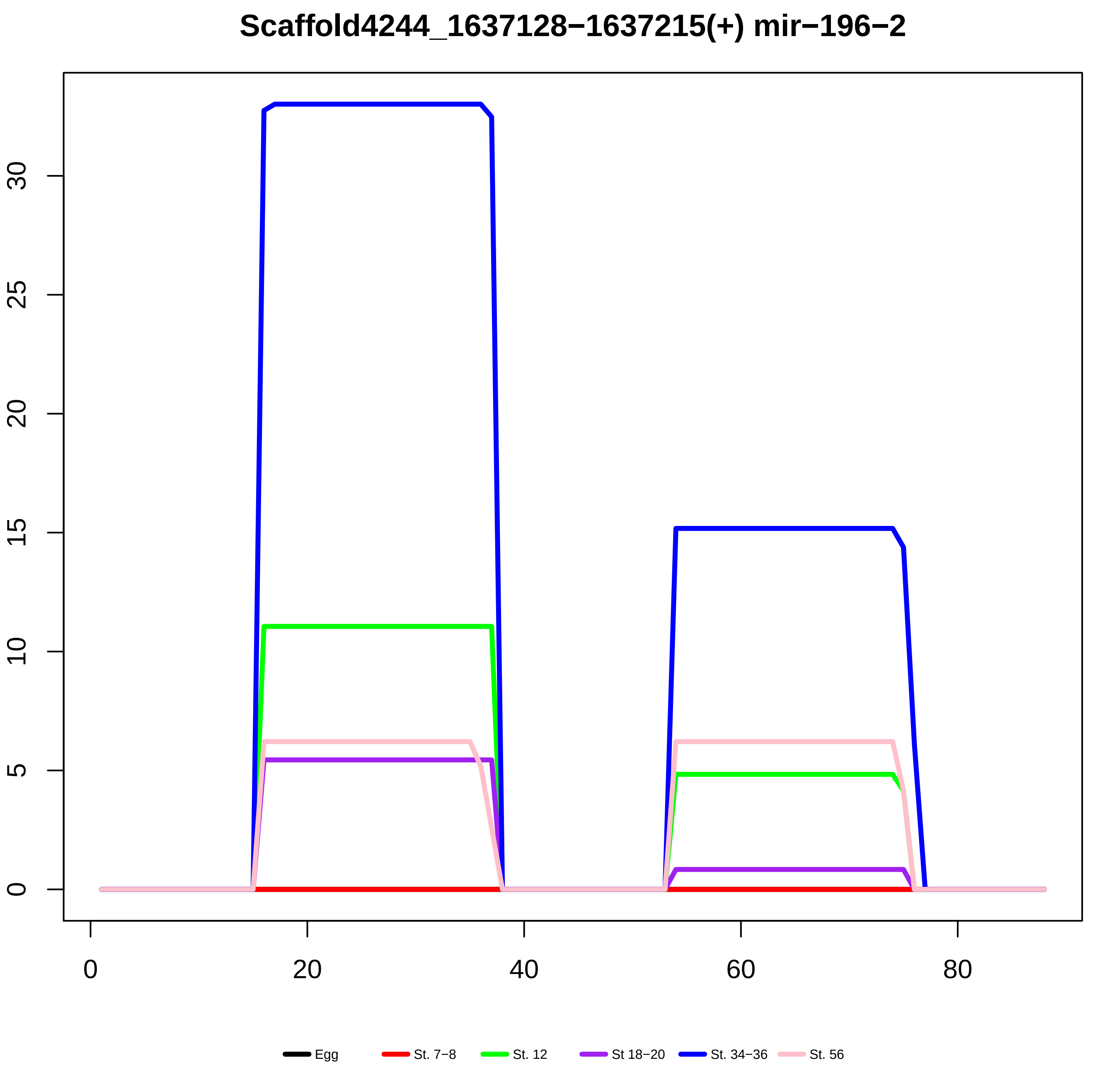

Scaffold4244_1637128−1637215(+) mir−196−2
0
5
10
15
20
25
30
0
20
40
60
80
Egg
St. 7−8
St. 12
St 18−20
St. 34−36
St. 56

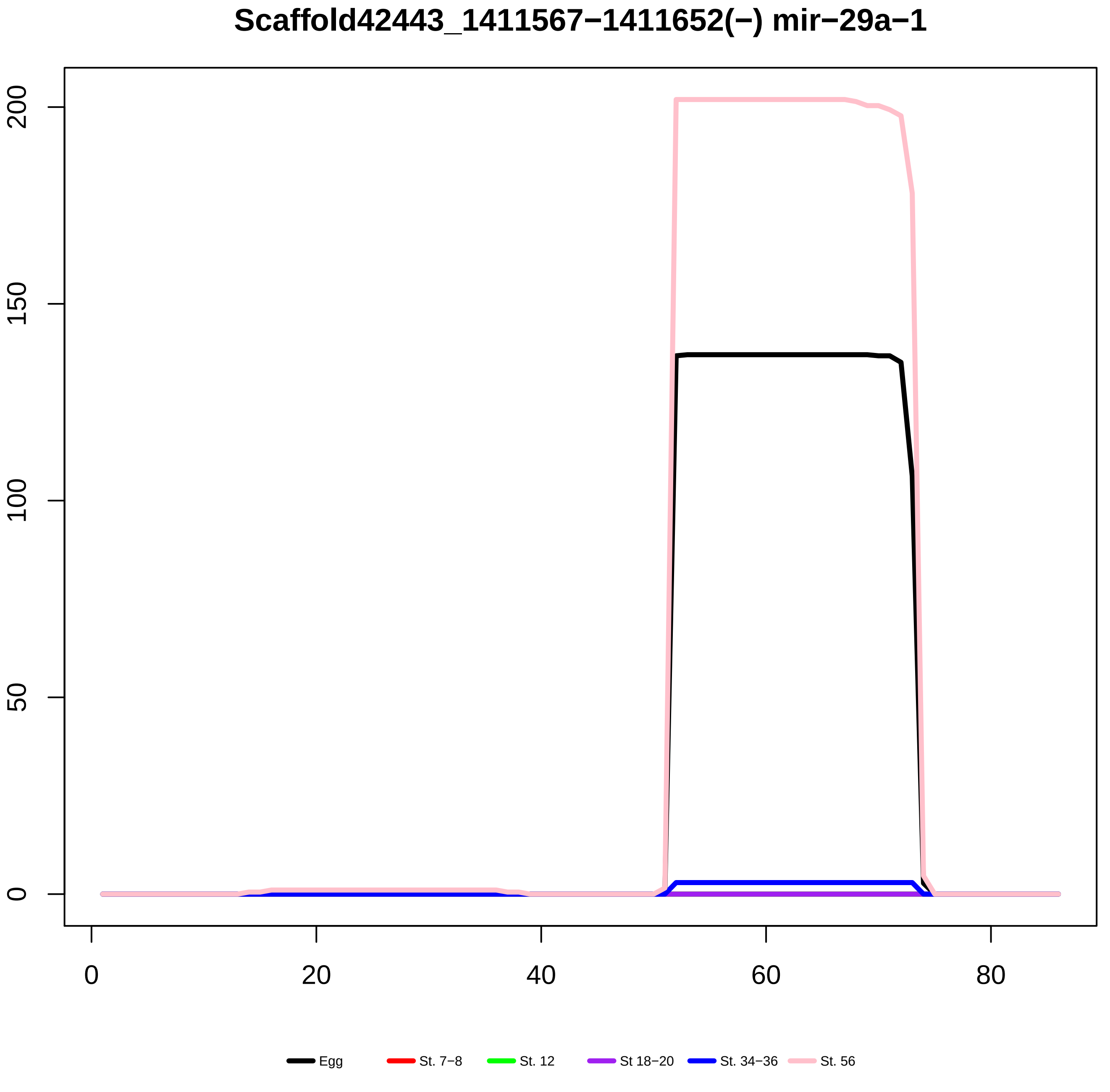
Scaffold42443_1411567−1411652(−) mir−29a−1
0
50
100
150
200
0
20
40
60
80
Egg
St. 7−8
St. 12
St 18−20
St. 34−36
St. 56

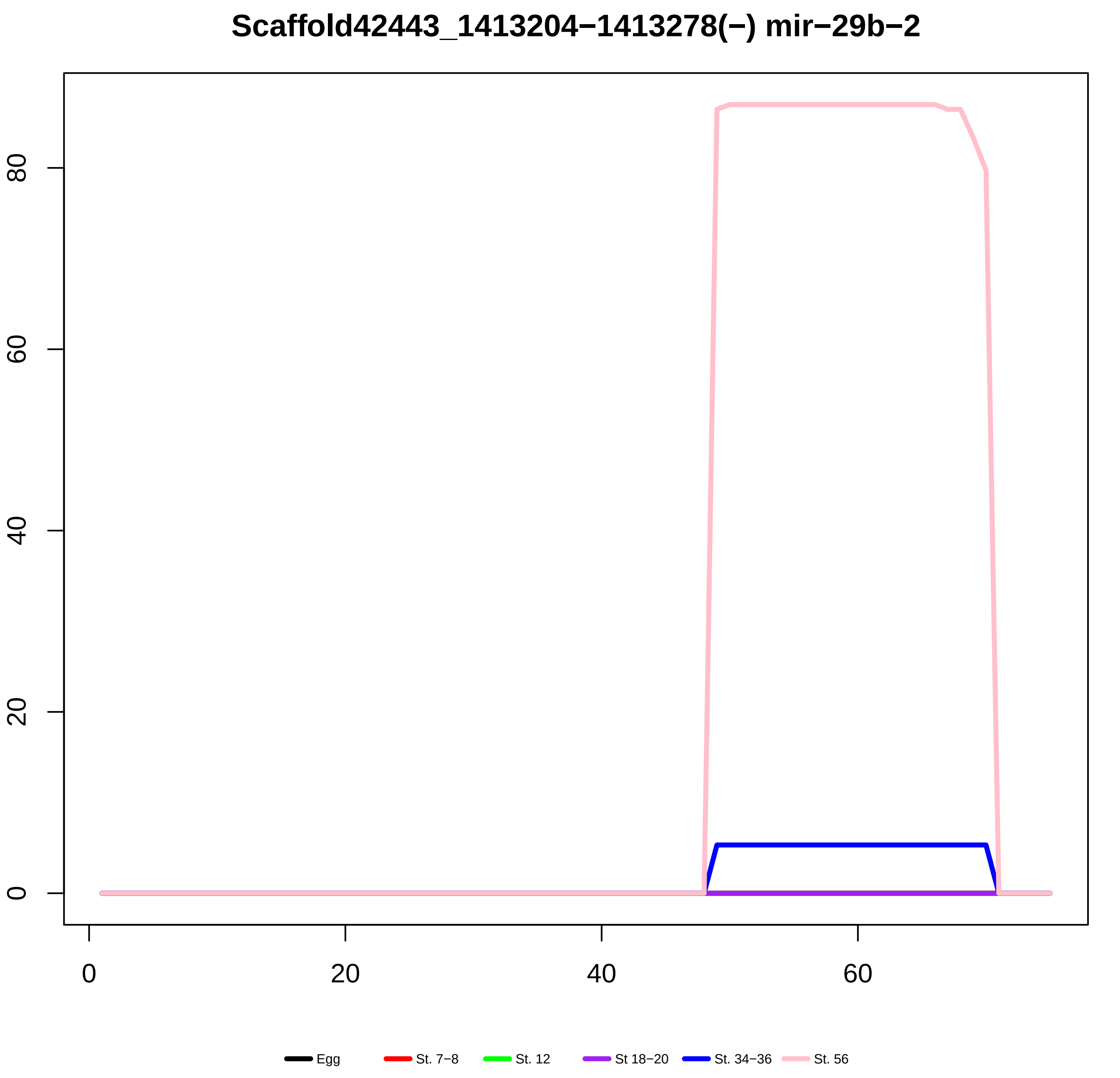
Scaffold42443_1413204−1413278(−) mir−29b−2
0
20
40
60
80
0
20
40
60
Egg
St. 7−8
St. 12
St 18−20
St. 34−36
St. 56

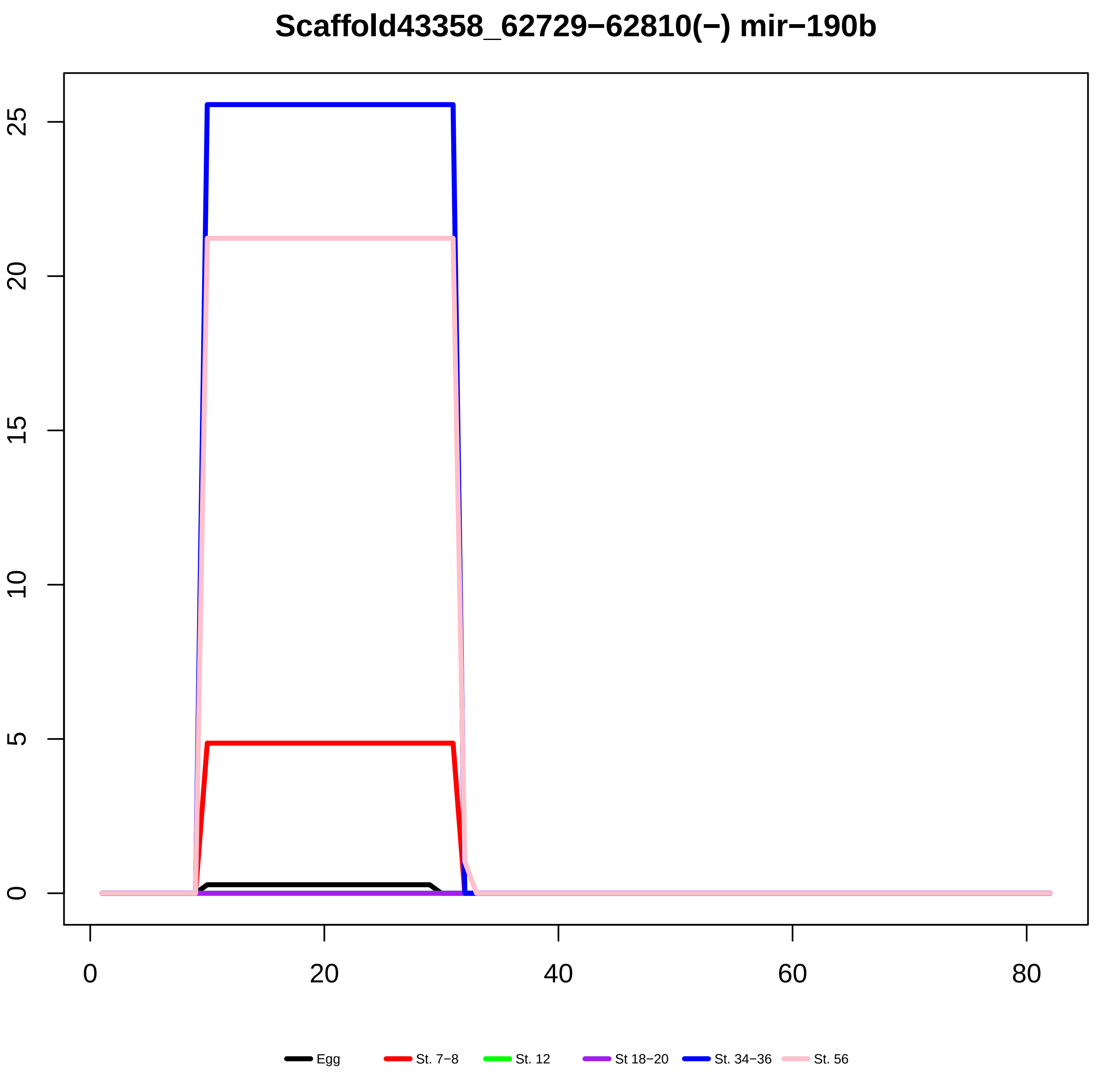

Scaffold43358_62729−62810(−) mir−190b
0
5
10
15
20
25
0
20
40
60
80
Egg
St. 7−8
St. 12
St 18−20
St. 34−36
St. 56

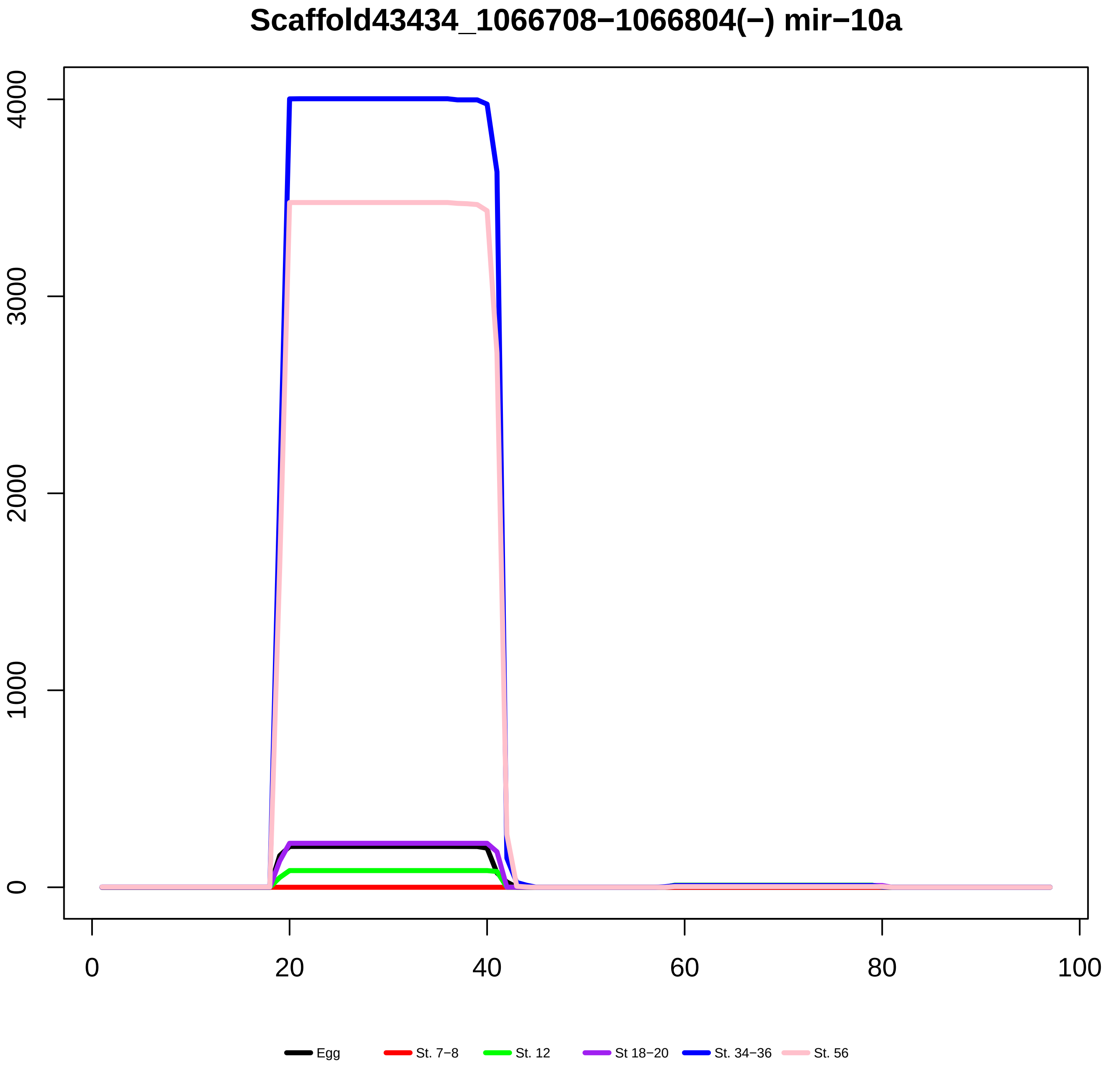

Scaffold43434_1066708−1066804(−) mir−10a
0
1000
2000
3000
4000
0
20
40
60
80
100
Egg
St. 7−8
St. 12
St 18−20
St. 34−36
St. 56

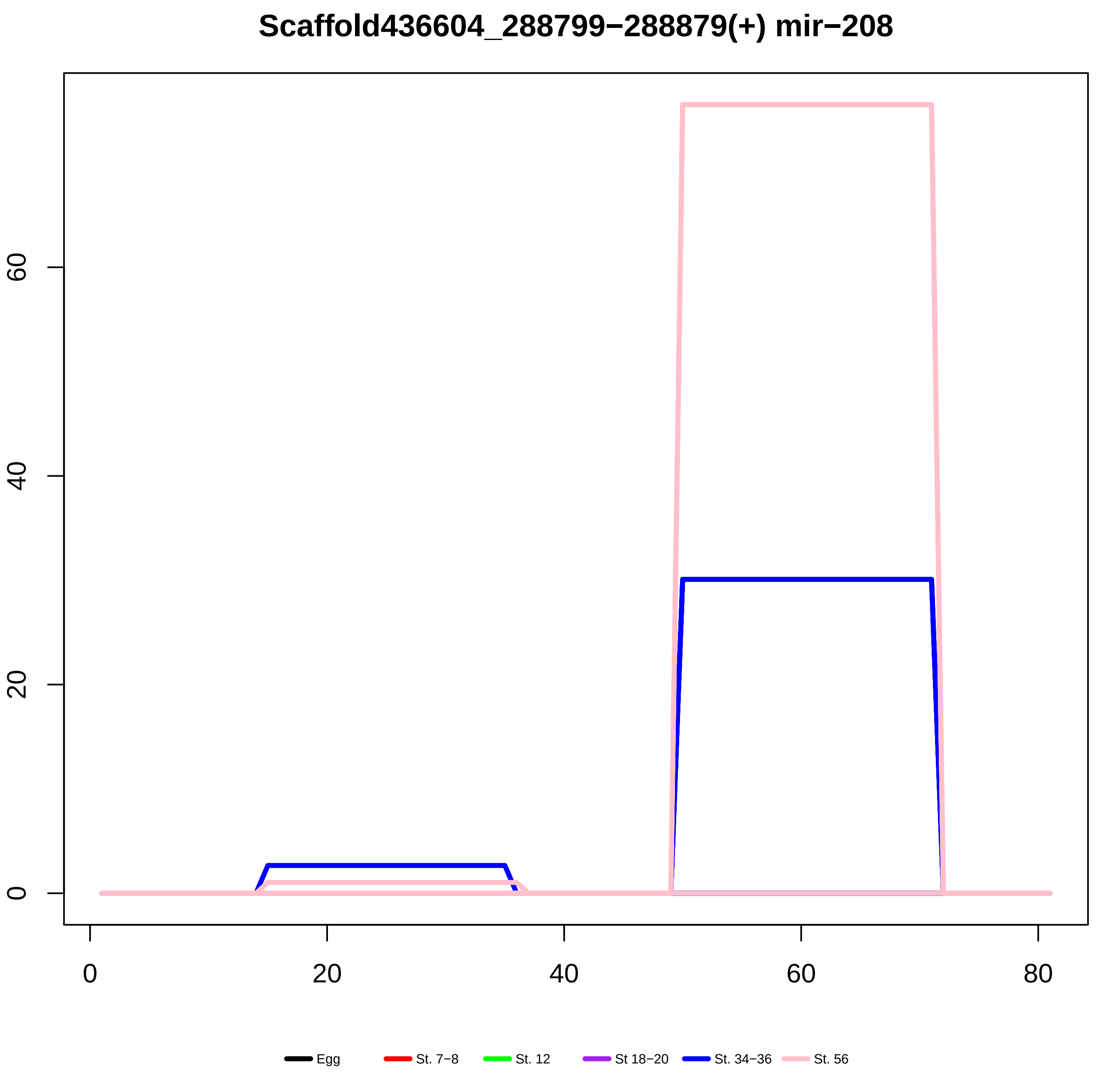
Scaffold436604_288799−288879(+) mir−208
0
20
40
60
0
20
40
60
80
Egg
St. 7−8
St. 12
St 18−20
St. 34−36
St. 56

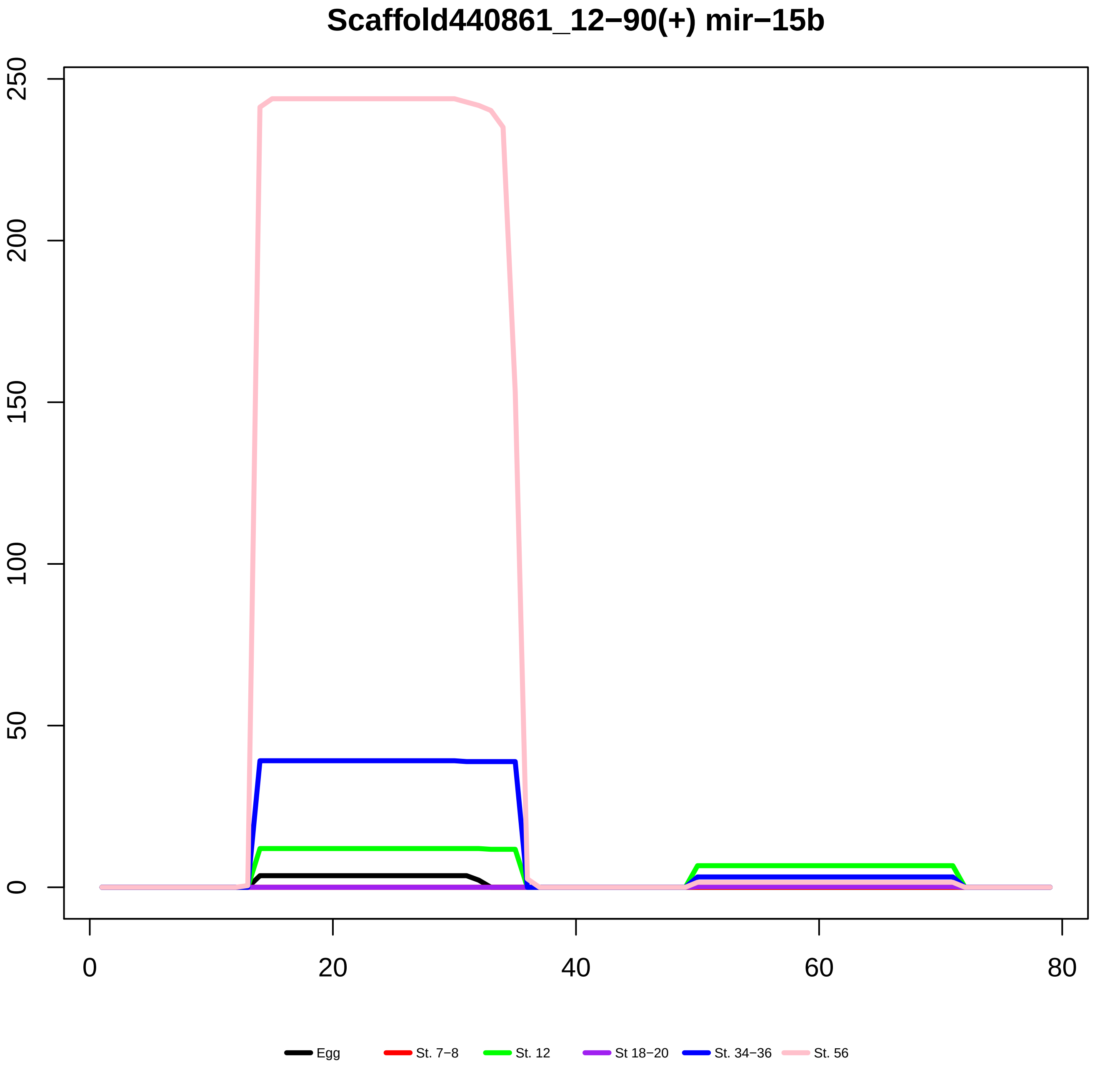
Scaffold440861_12−90(+) mir−15b
250
200
150
100
50
0
0
20
40
60
80
Egg
St. 7−8
St. 12
St 18−20
St. 34−36
St. 56

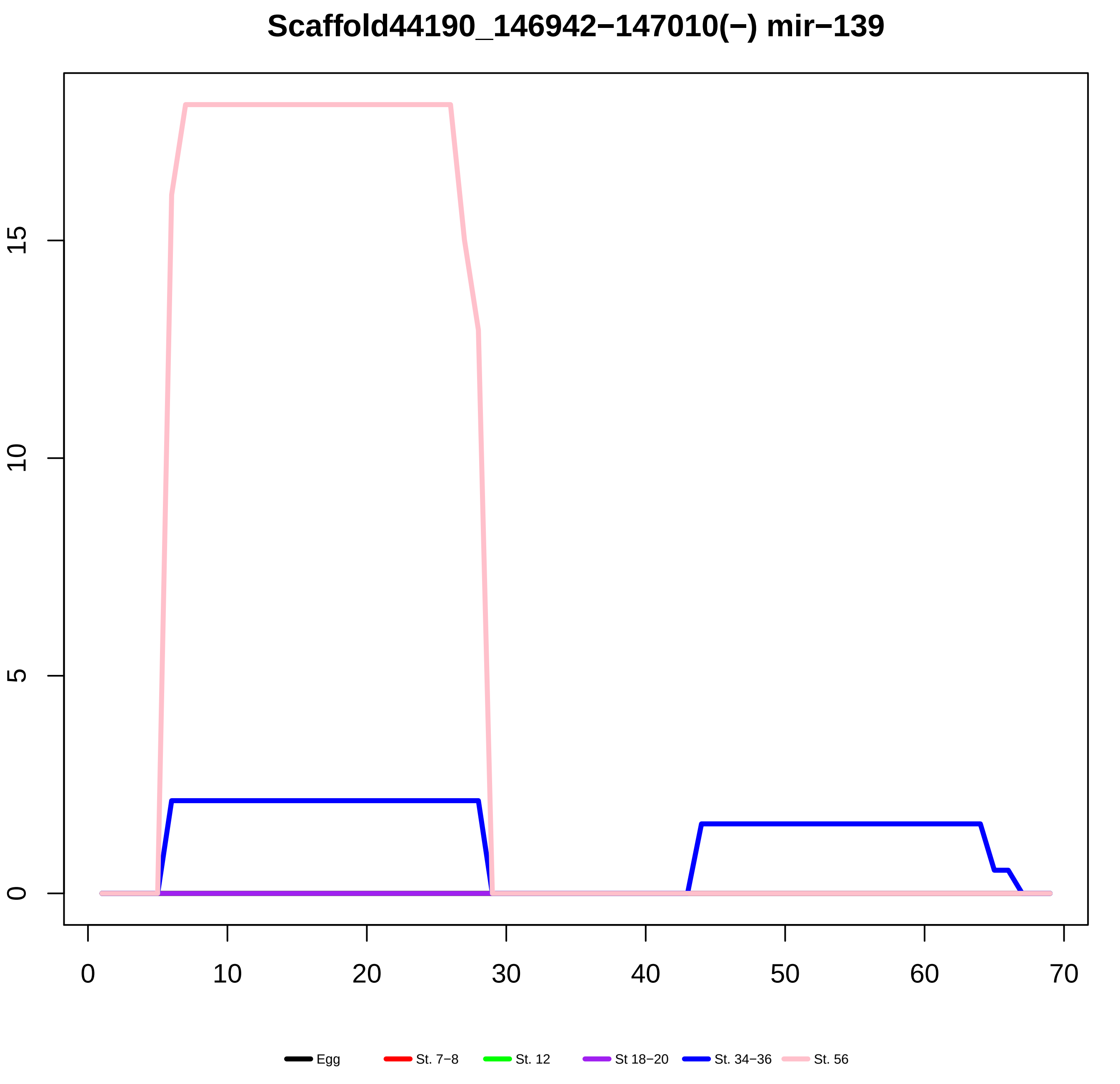
Scaffold44190_146942−147010(−) mir−139
0
5
10
15
0
10
20
30
40
50
60
70
Egg
St. 7−8
St. 12
St 18−20
St. 34−36
St. 56

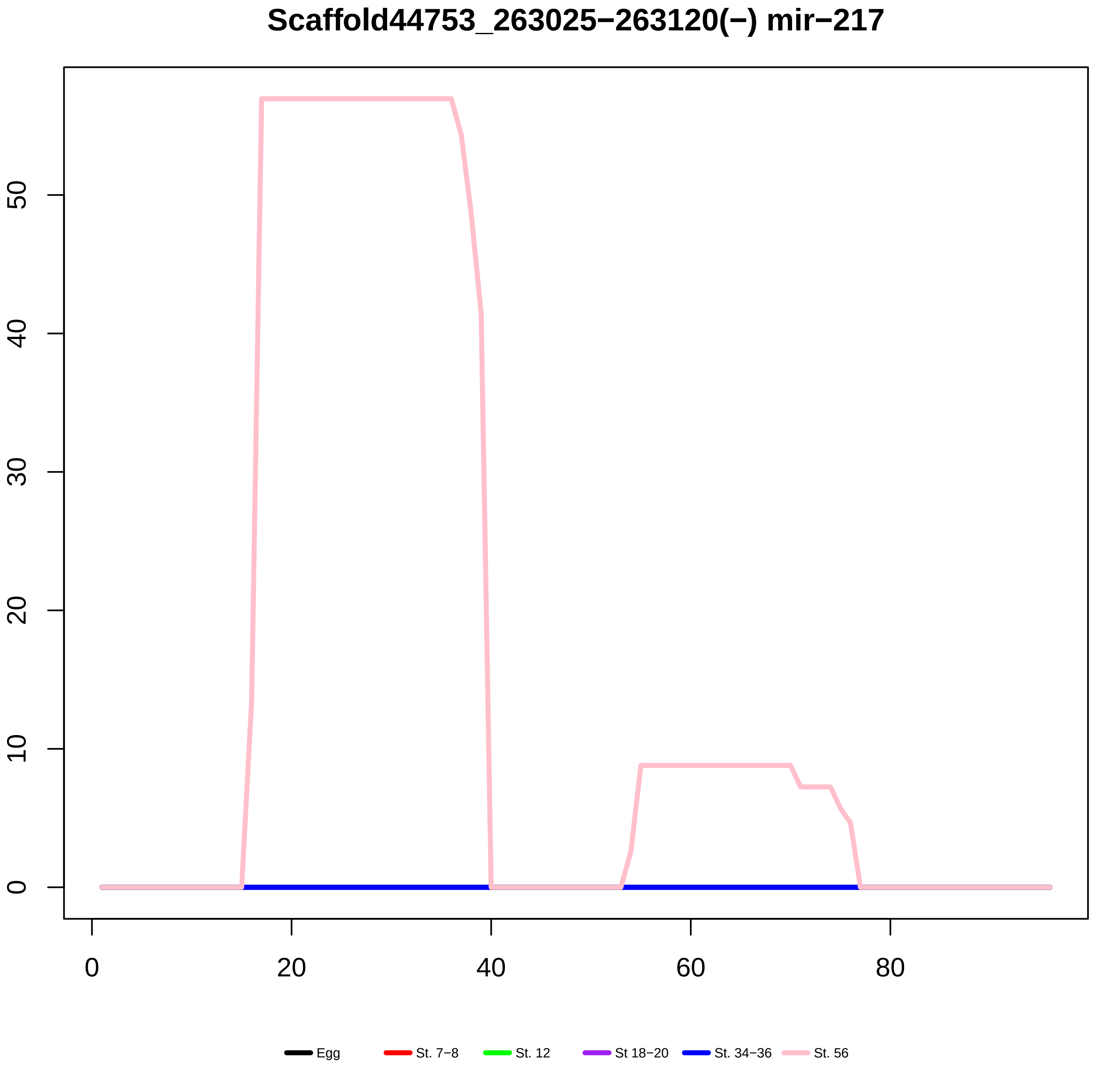
Scaffold44753_263025−263120(−) mir−217
0
10
20
30
40
50
0
20
40
60
80
Egg
St. 7−8
St. 12
St 18−20
St. 34−36
St. 56

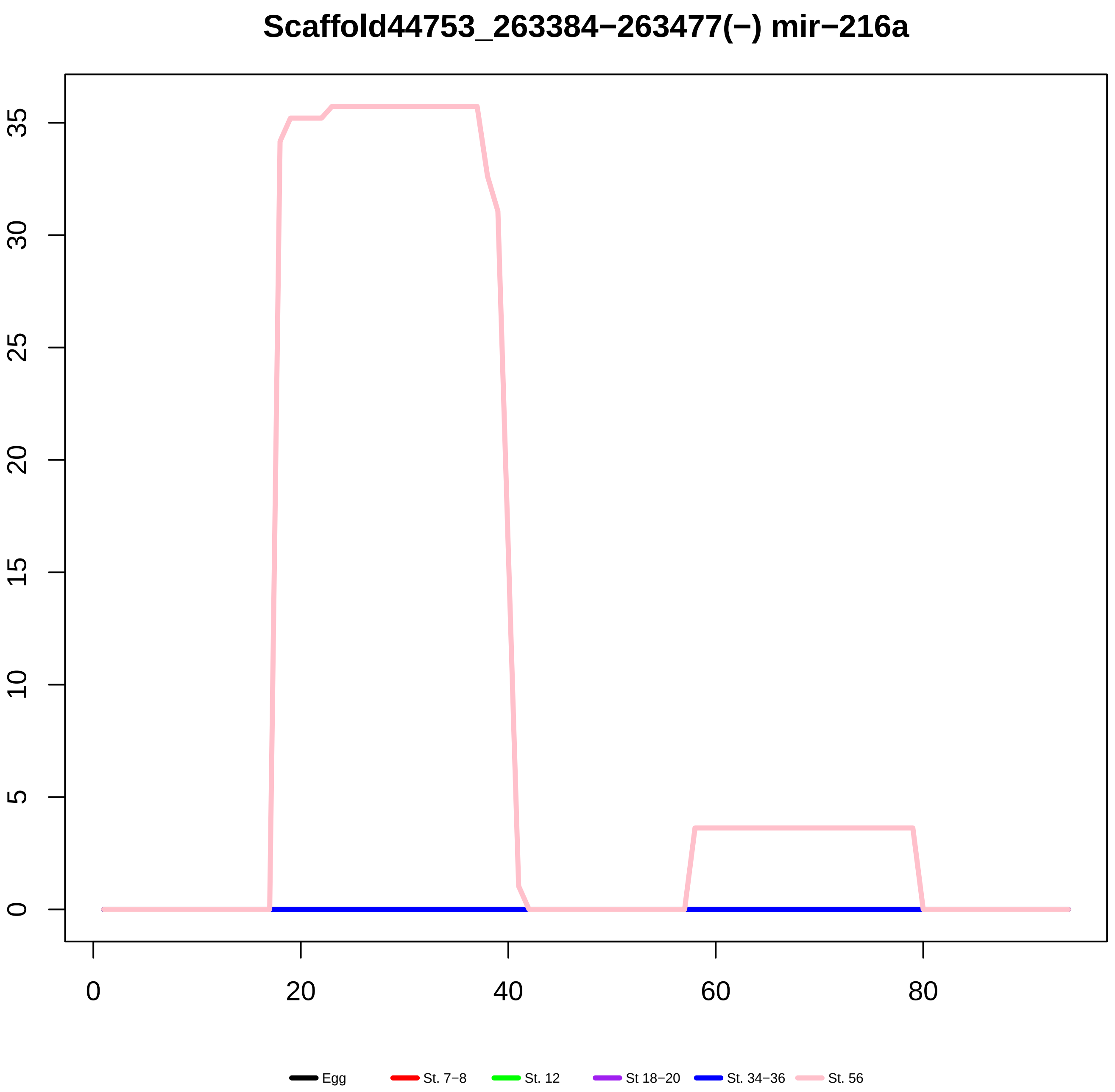

Scaffold44753_263384−263477(−) mir−216a
0
5
10
15
20
25
30
35
0
20
40
60
80
Egg
St. 7−8
St. 12
St 18−20
St. 34−36
St. 56

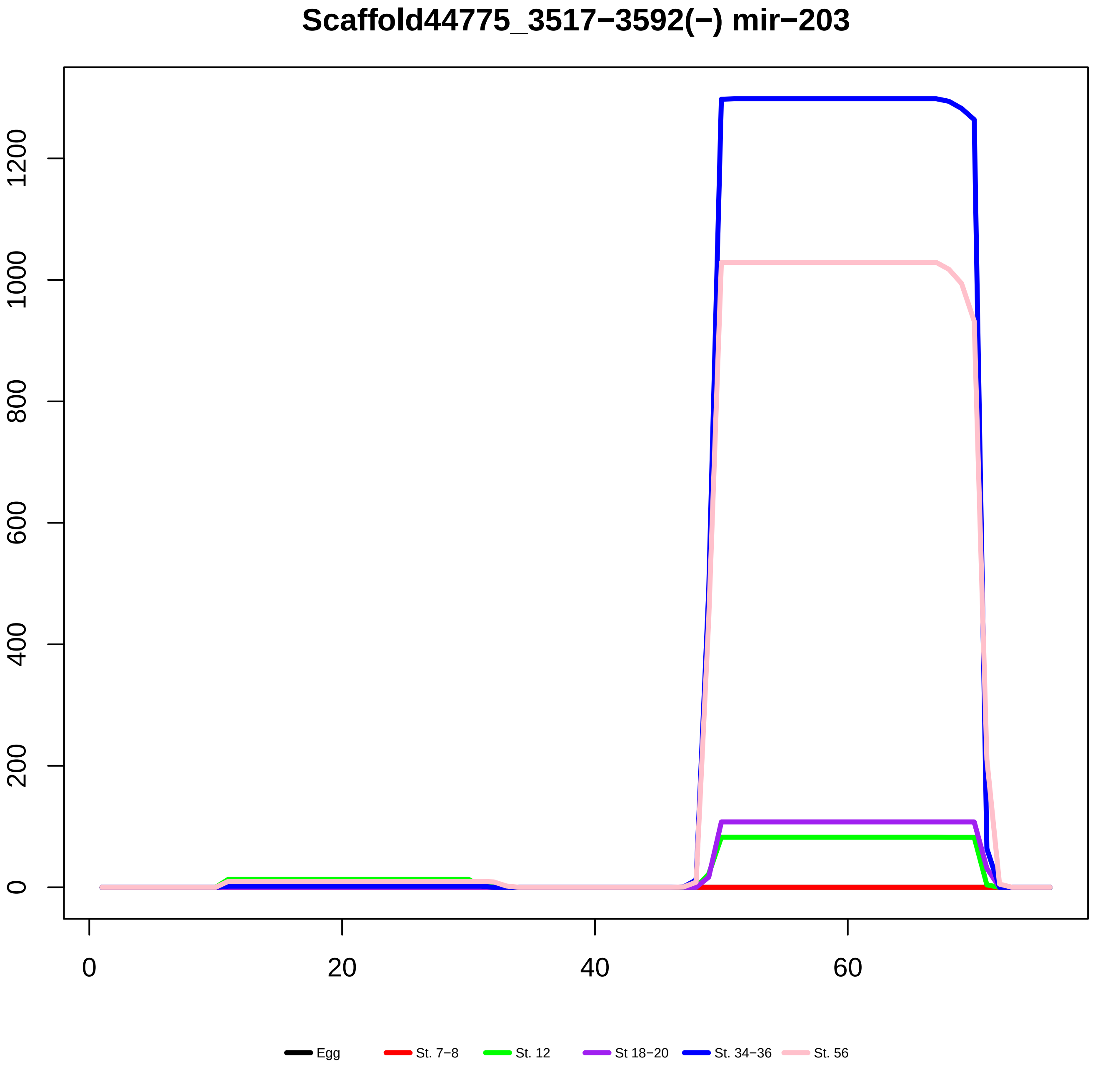
Scaffold44775_3517−3592(−) mir−203
0
200
400
600
800
1000
1200
0
20
40
60
Egg
St. 7−8
St. 12
St 18−20
St. 34−36
St. 56

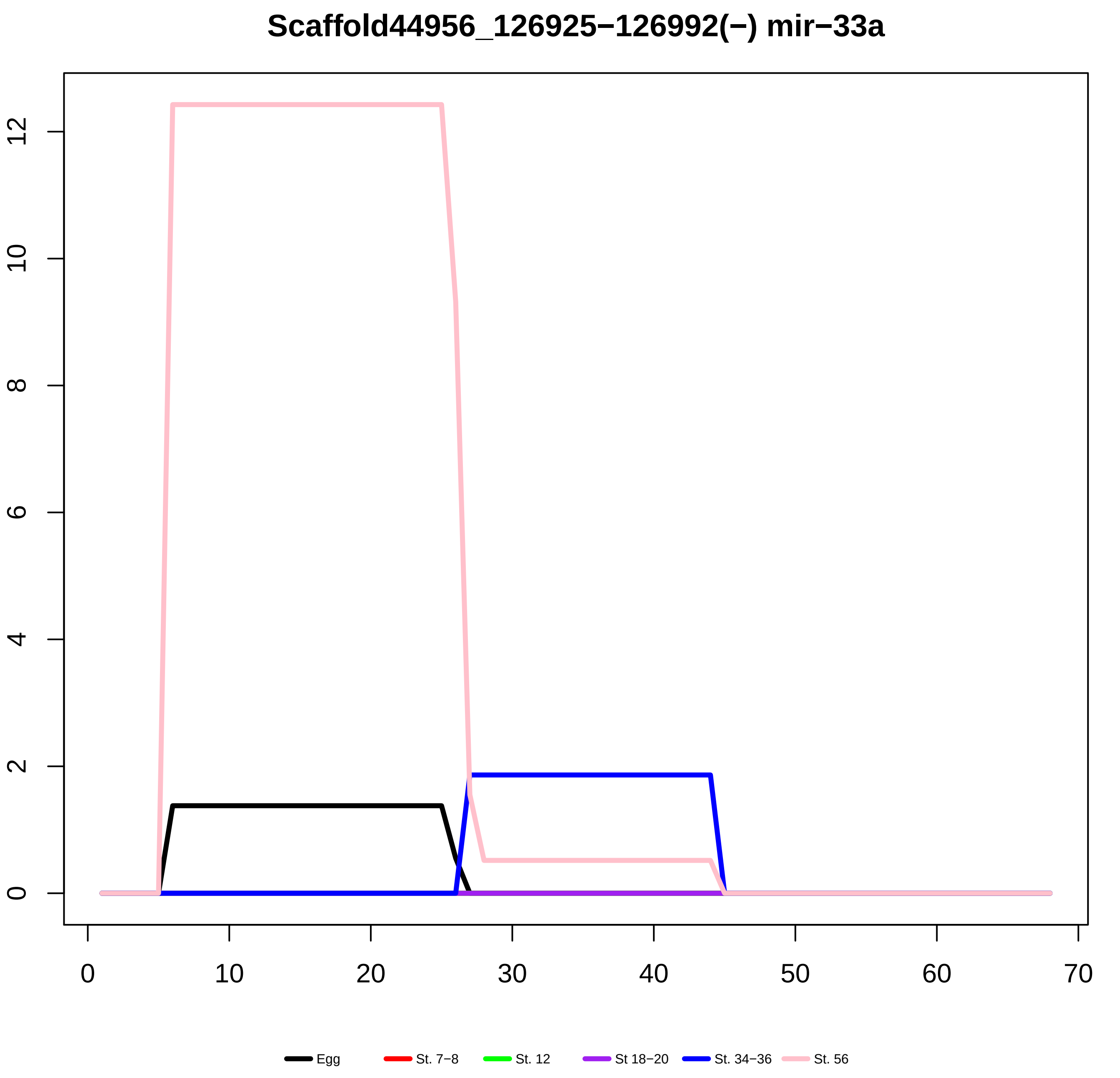

Scaffold44956_126925−126992(−) mir−33a
0
2
4
6
8
10
12
0
10
20
30
40
50
60
70
Egg
St. 7−8
St. 12
St 18−20
St. 34−36
St. 56

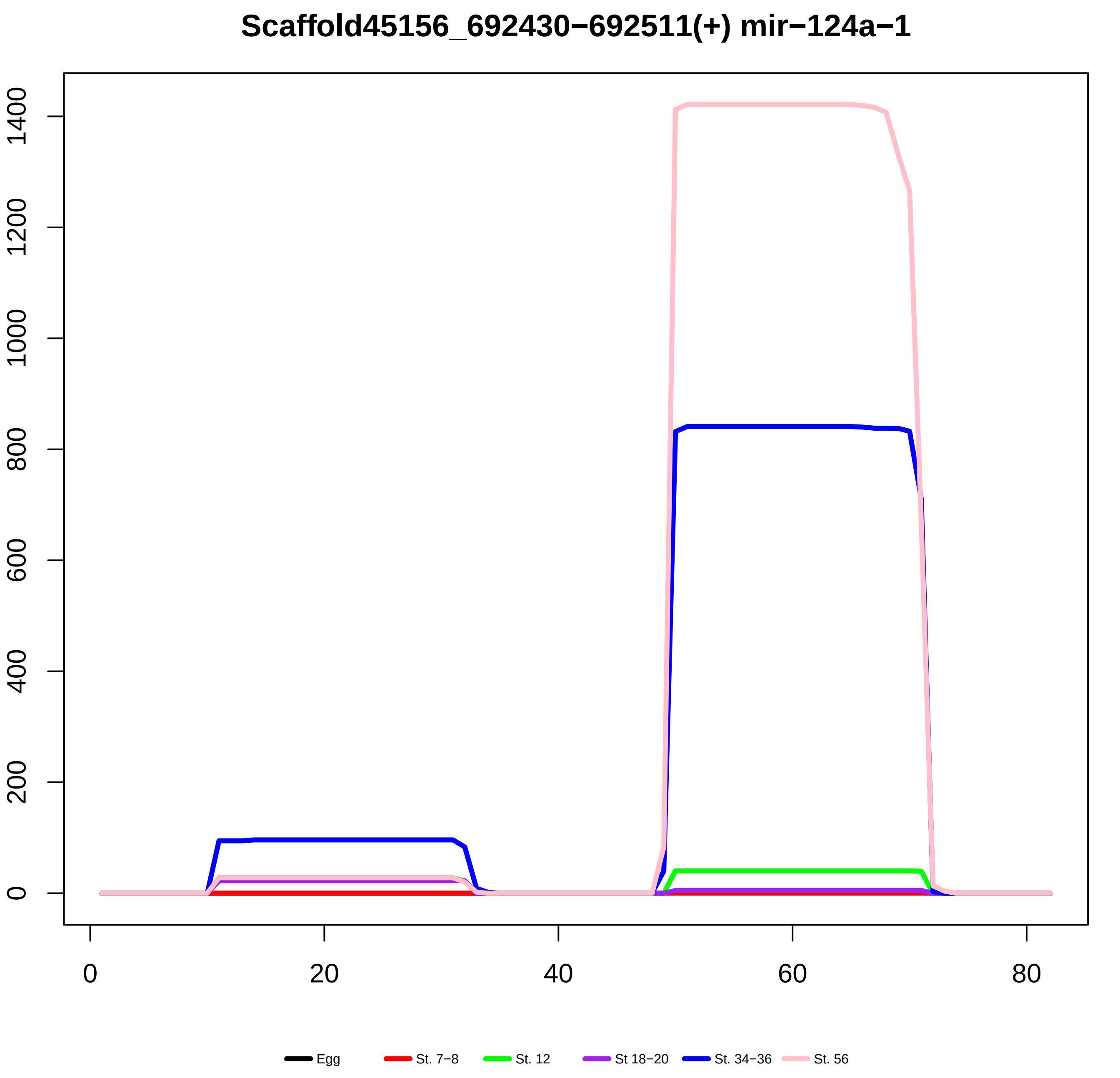
Scaffold45156_692430−692511(+) mir−124a−1
0
200
400
600
800
1000
1200
1400
0
20
40
60
80
Egg
St. 7−8
St. 12
St 18−20
St. 34−36
St. 56

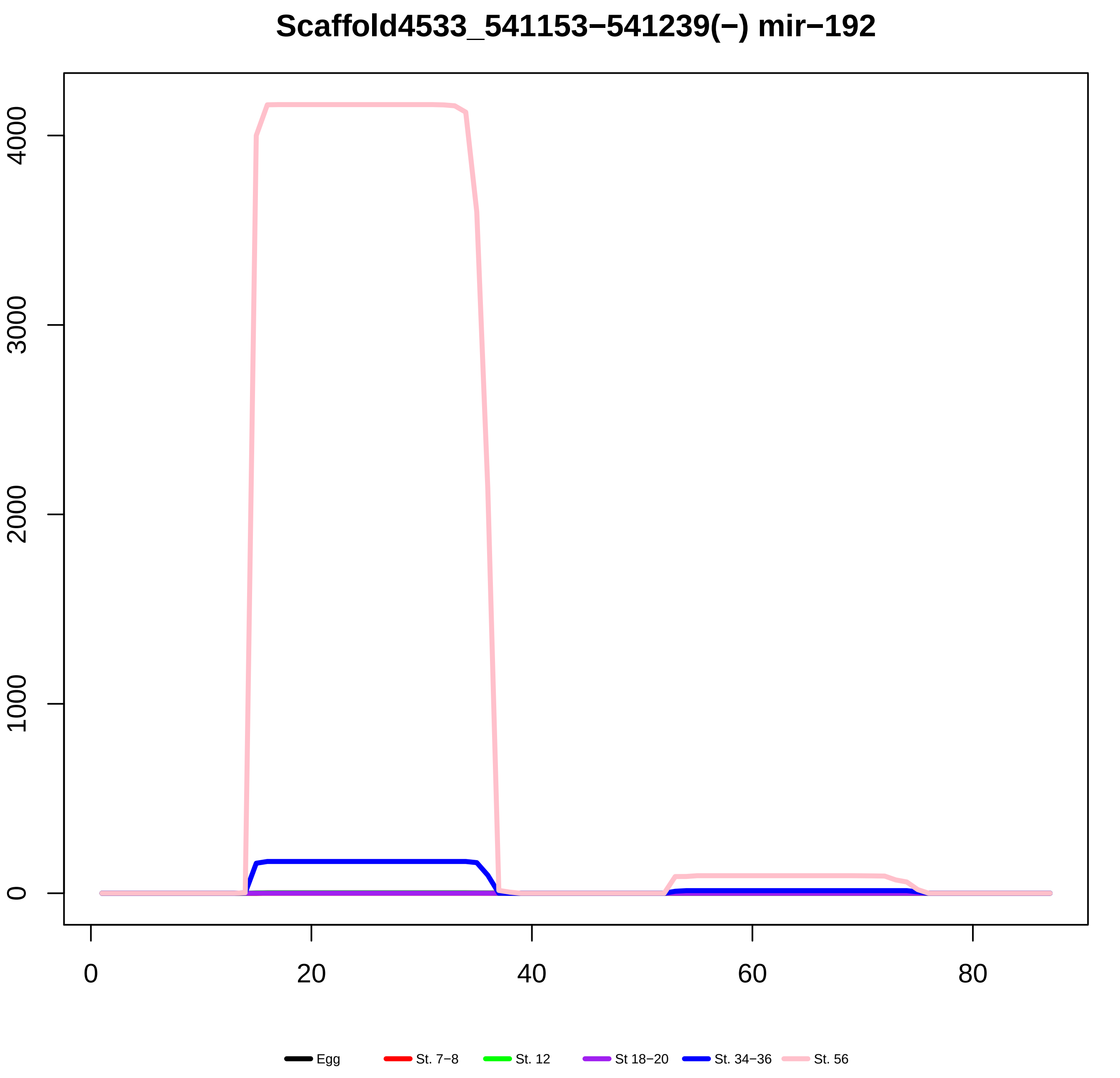

Scaffold4533_541153−541239(−) mir−192
0
1000
2000
3000
4000
0
20
40
60
80
Egg
St. 7−8
St. 12
St 18−20
St. 34−36
St. 56

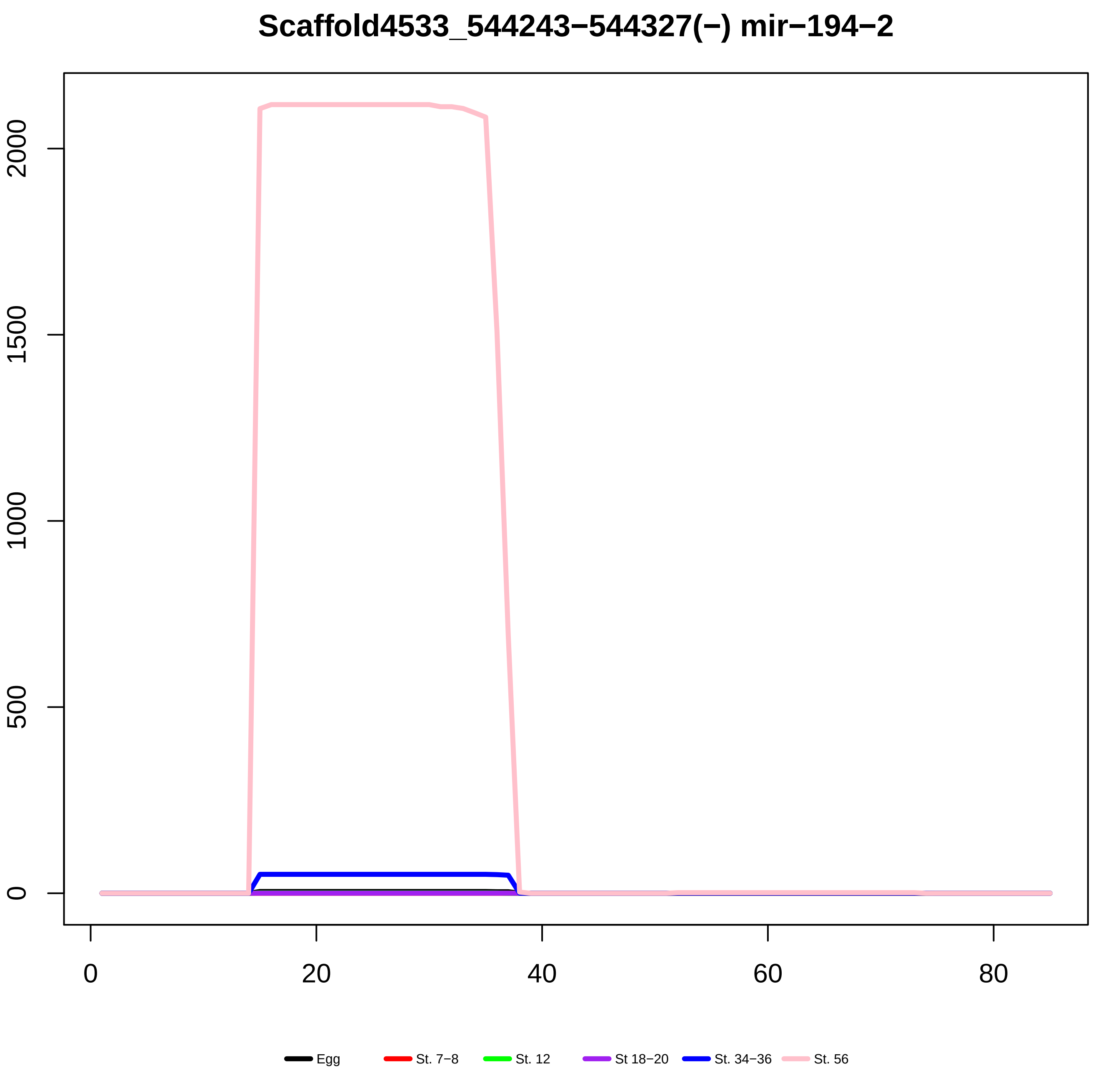

Scaffold4533_544243−544327(−) mir−194−2
0
500
1000
1500
2000
0
20
40
60
80
Egg
St. 7−8
St. 12
St 18−20
St. 34−36
St. 56

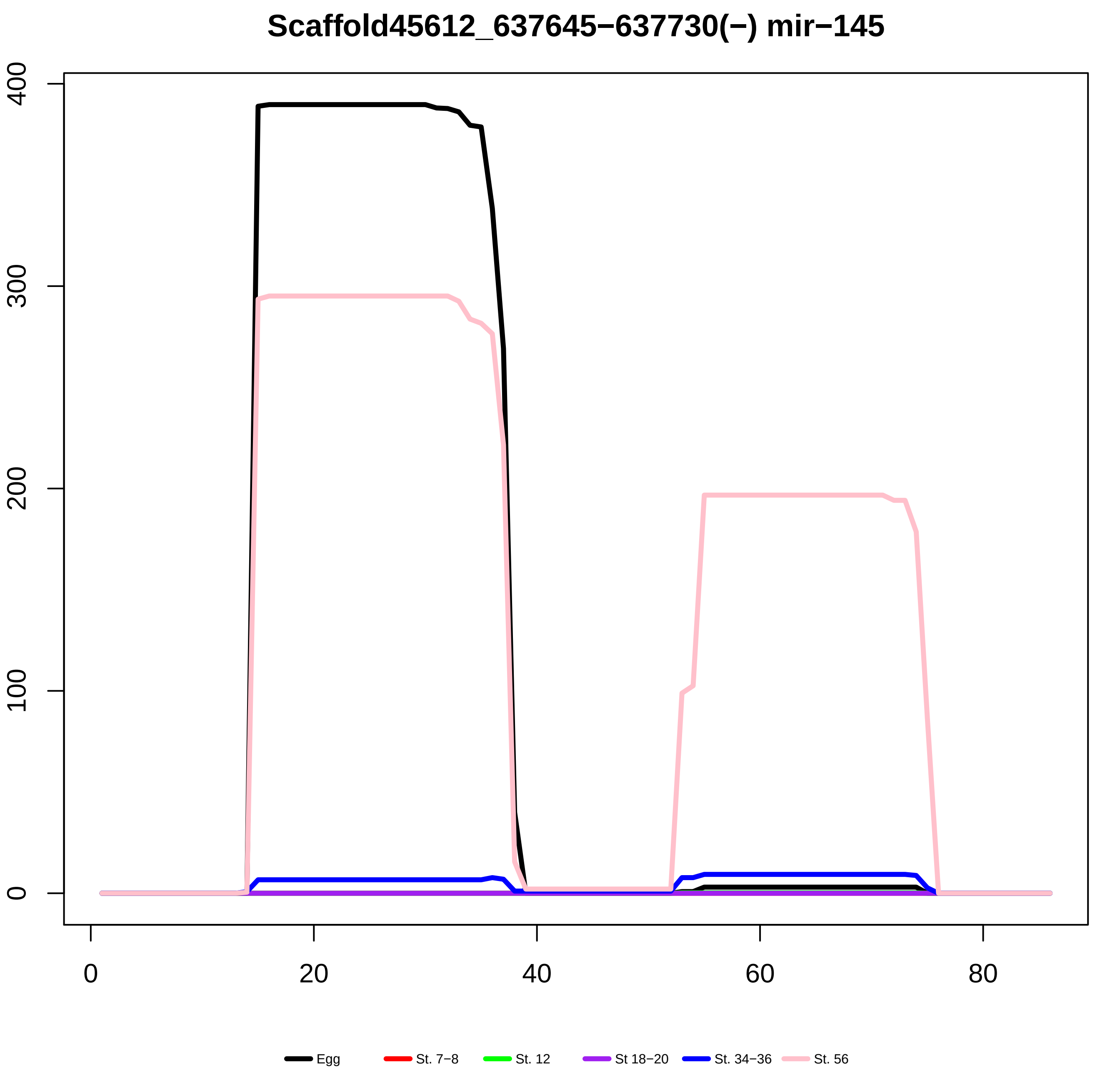

Scaffold45612_637645−637730(−) mir−145
400
300
200
100
0
0
20
40
60
80
Egg
St. 7−8
St. 12
St 18−20
St. 34−36
St. 56

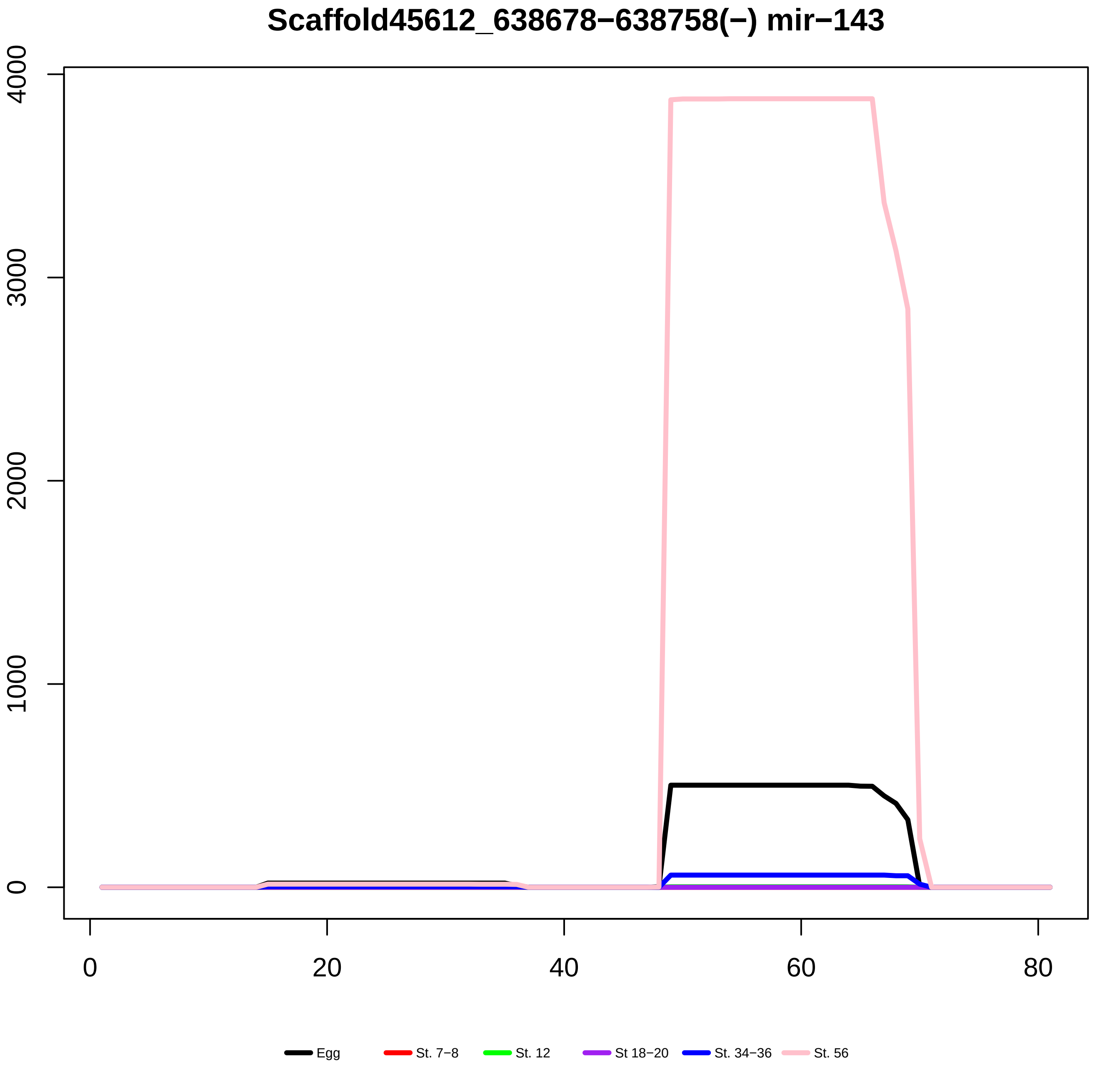
Scaffold45612_638678−638758(−) mir−143
4000
3000
2000
1000
0
0
20
40
60
80
Egg
St. 7−8
St. 12
St 18−20
St. 34−36
St. 56

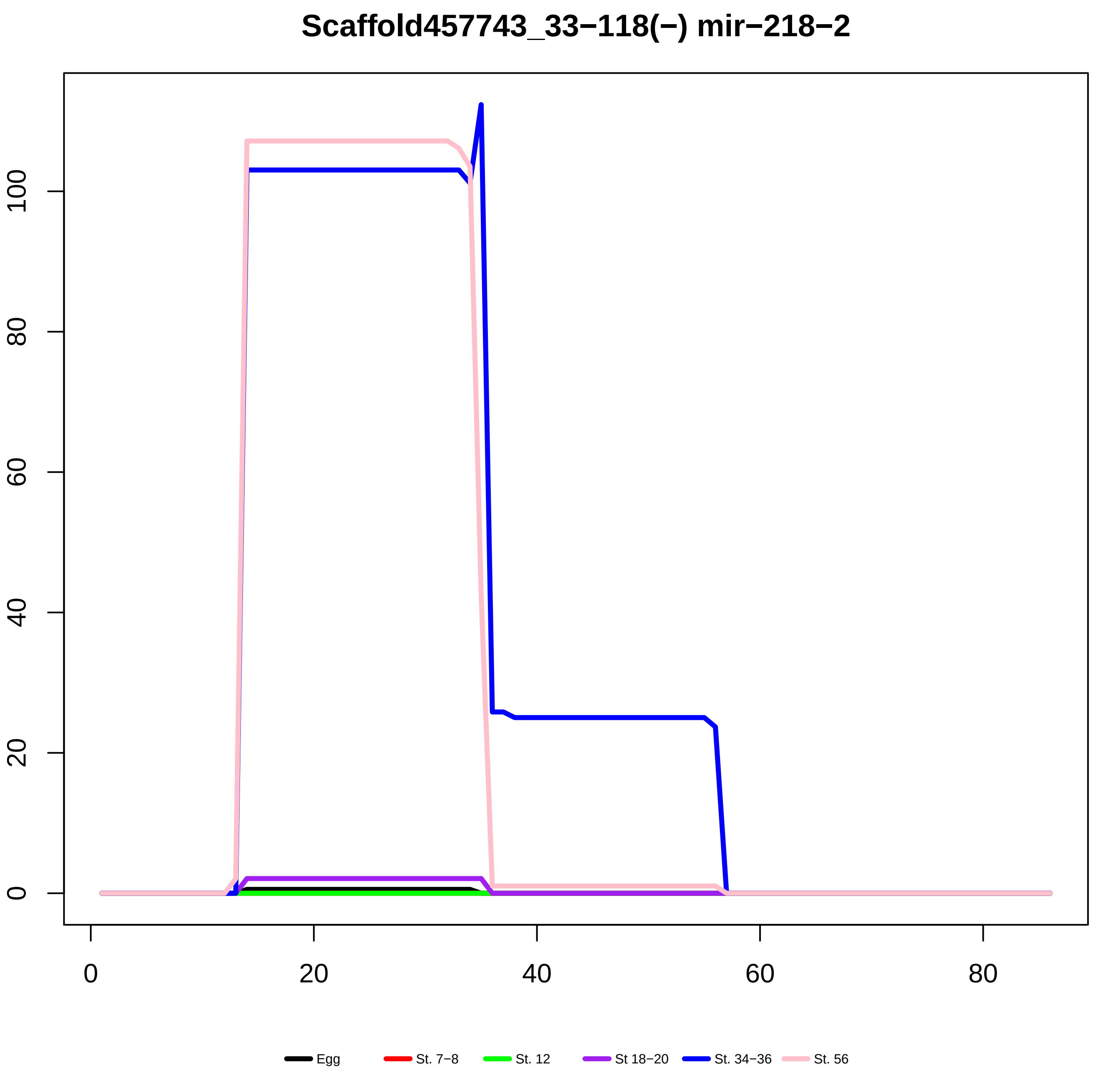
Scaffold457743_33−118(−) mir−218−2
0
20
40
60
80
100
0
20
40
60
80
Egg
St. 7−8
St. 12
St 18−20
St. 34−36
St. 56

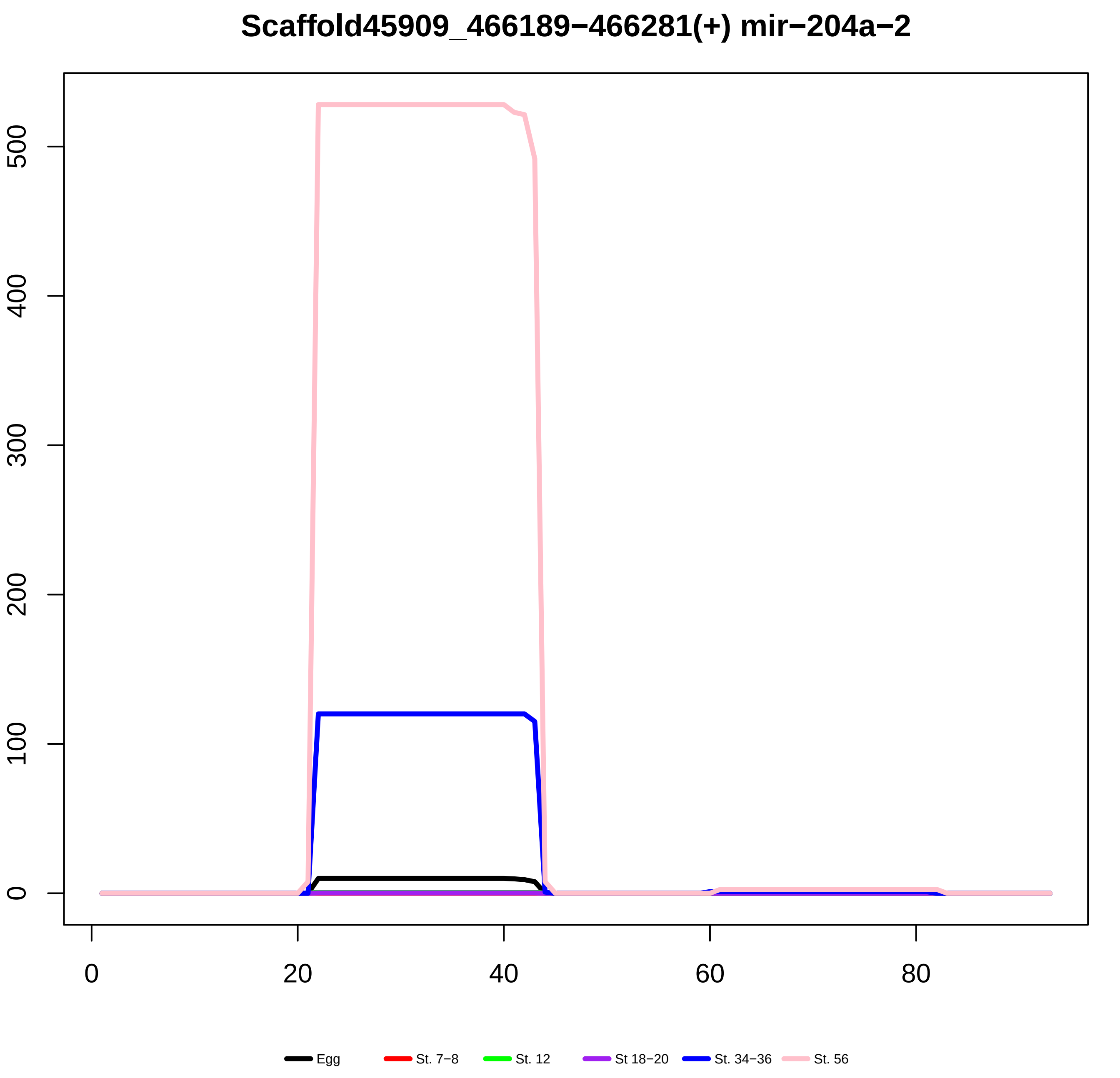

Scaffold45909_466189−466281(+) mir−204a−2
0
100
200
300
400
500
0
20
40
60
80
Egg
St. 7−8
St. 12
St 18−20
St. 34−36
St. 56

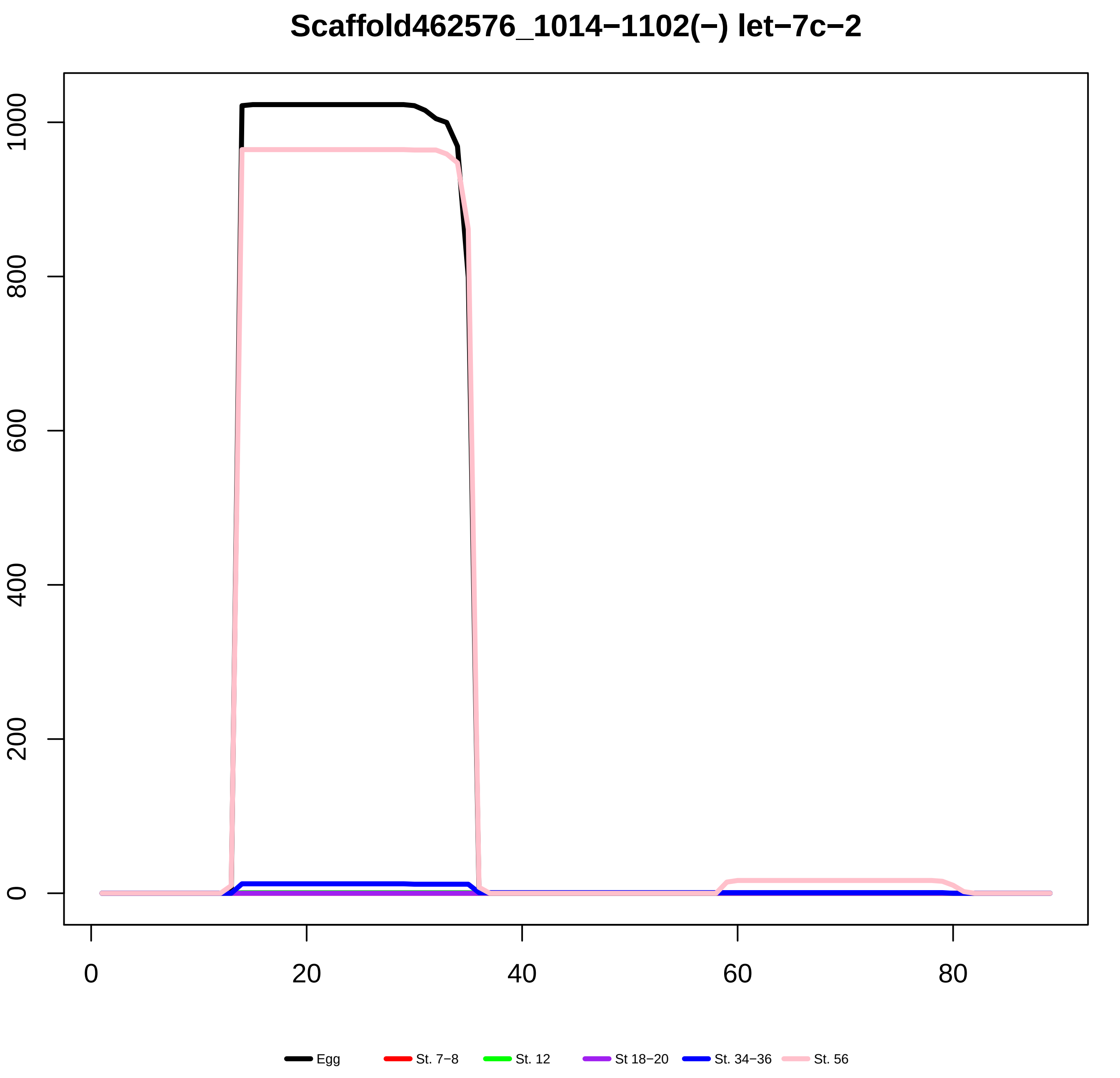
Scaffold462576_1014−1102(−) let−7c−2
0
200
400
600
800
1000
0
20
40
60
80
Egg
St. 7−8
St. 12
St 18−20
St. 34−36
St. 56

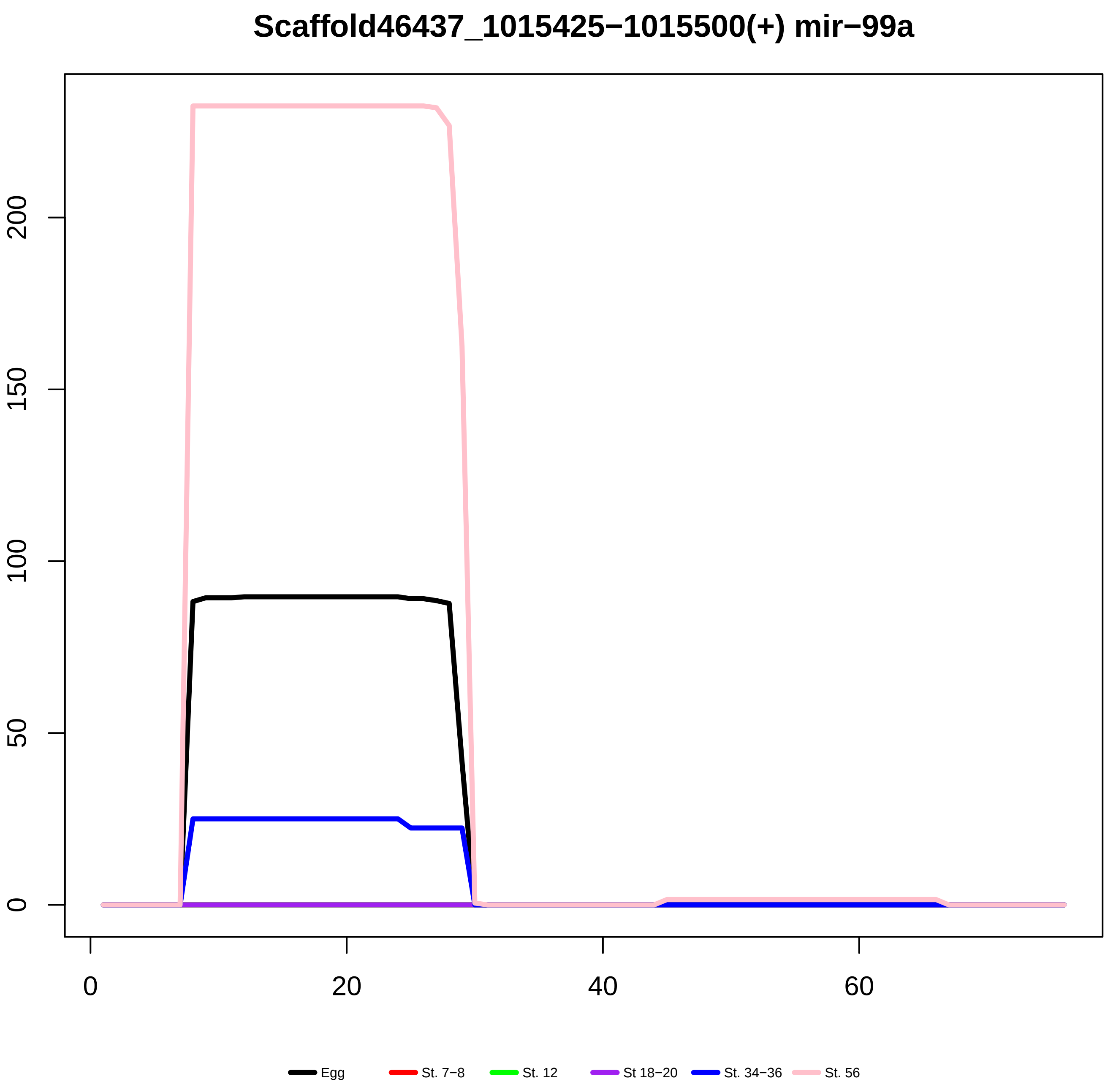

Scaffold46437_1015425−1015500(+) mir−99a
0
50
100
150
200
0
20
40
60
Egg
St. 7−8
St. 12
St 18−20
St. 34−36
St. 56

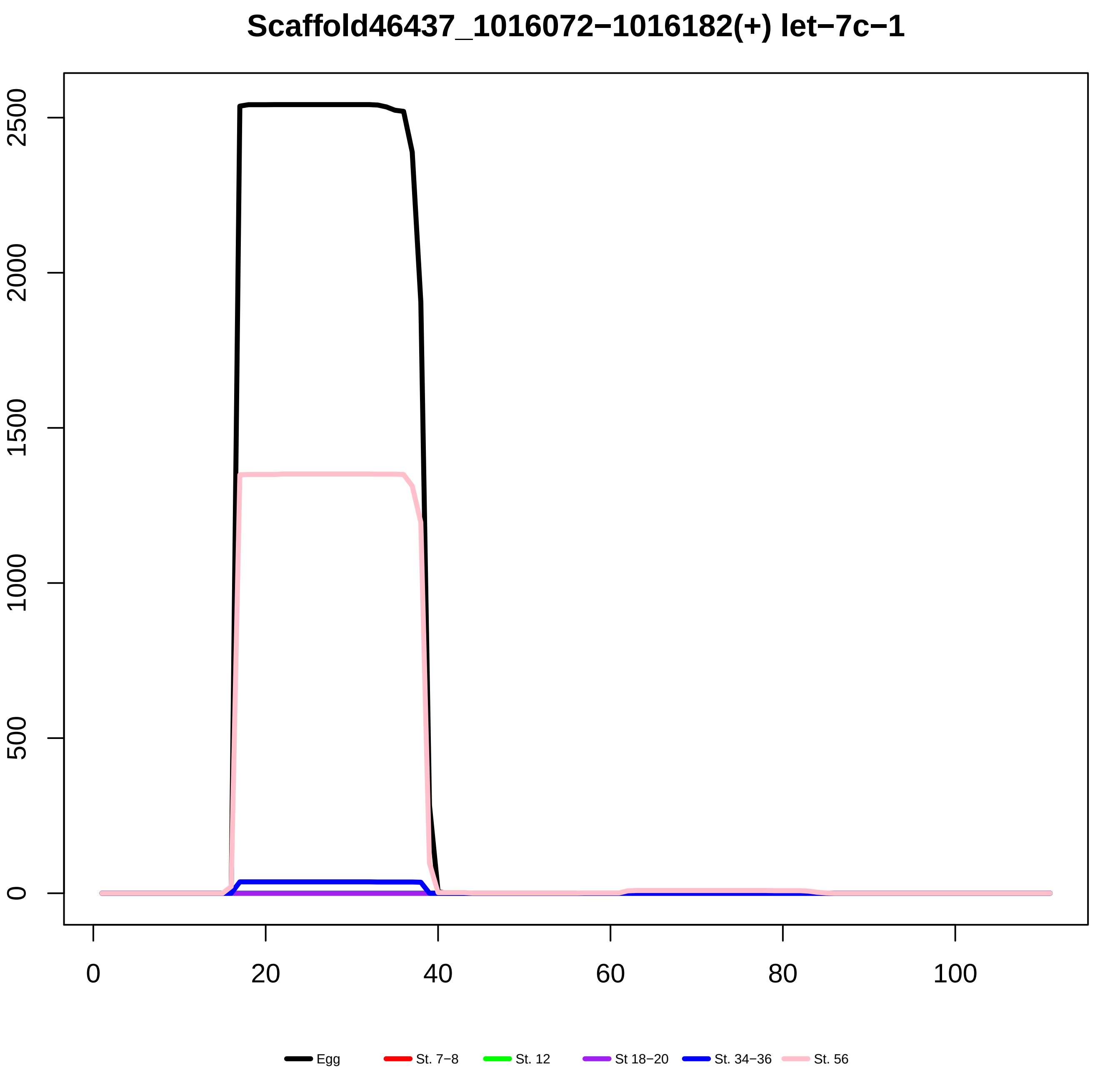

Scaffold46437_1016072−1016182(+) let−7c−1
0
500
1000
1500
2000
2500
0
20
40
60
80
100
Egg
St. 7−8
St. 12
St 18−20
St. 34−36
St. 56

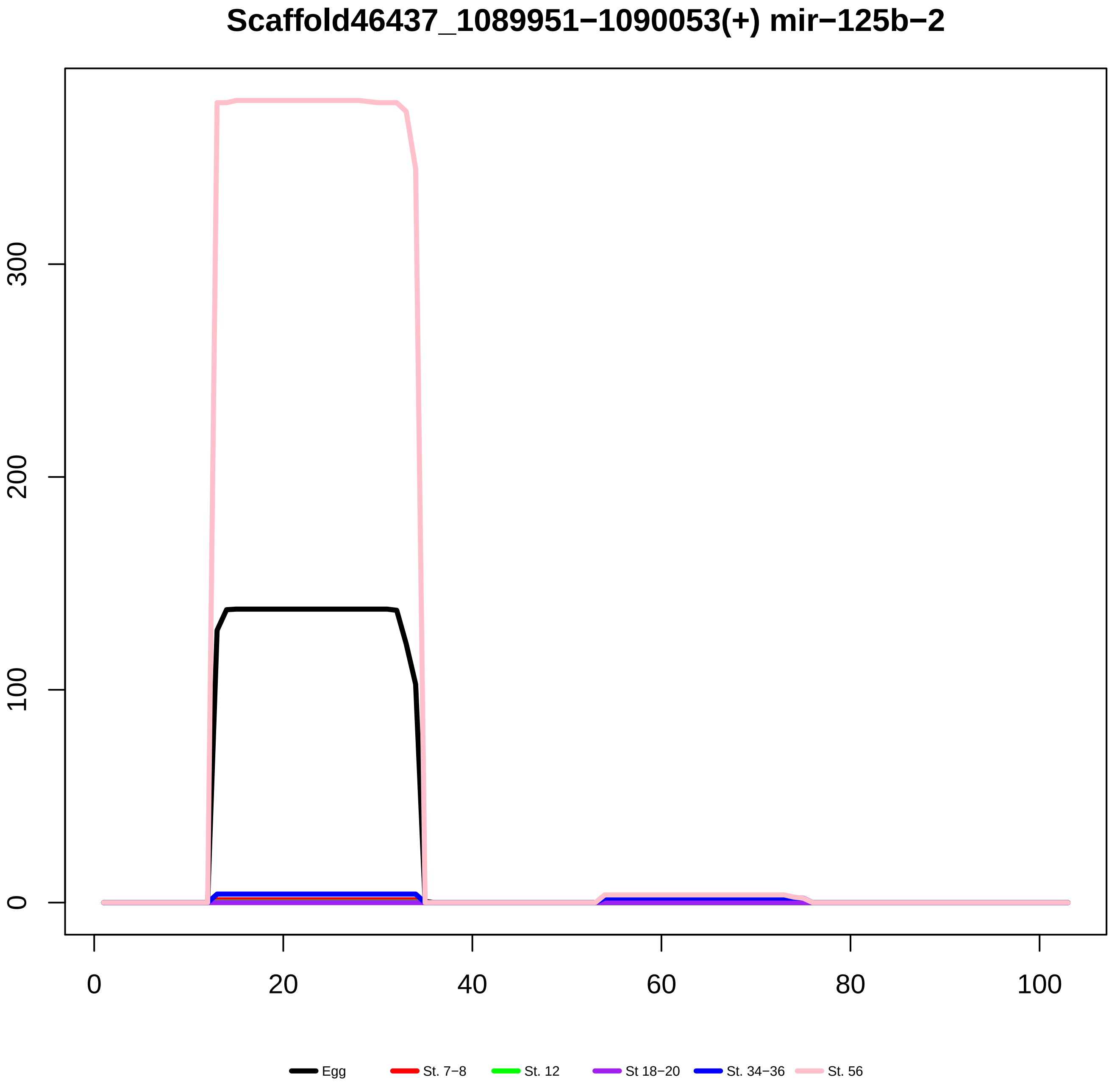

Scaffold46437_1089951−1090053(+) mir−125b−2
0
100
200
300
0
20
40
60
80
100
Egg
St. 7−8
St. 12
St 18−20
St. 34−36
St. 56

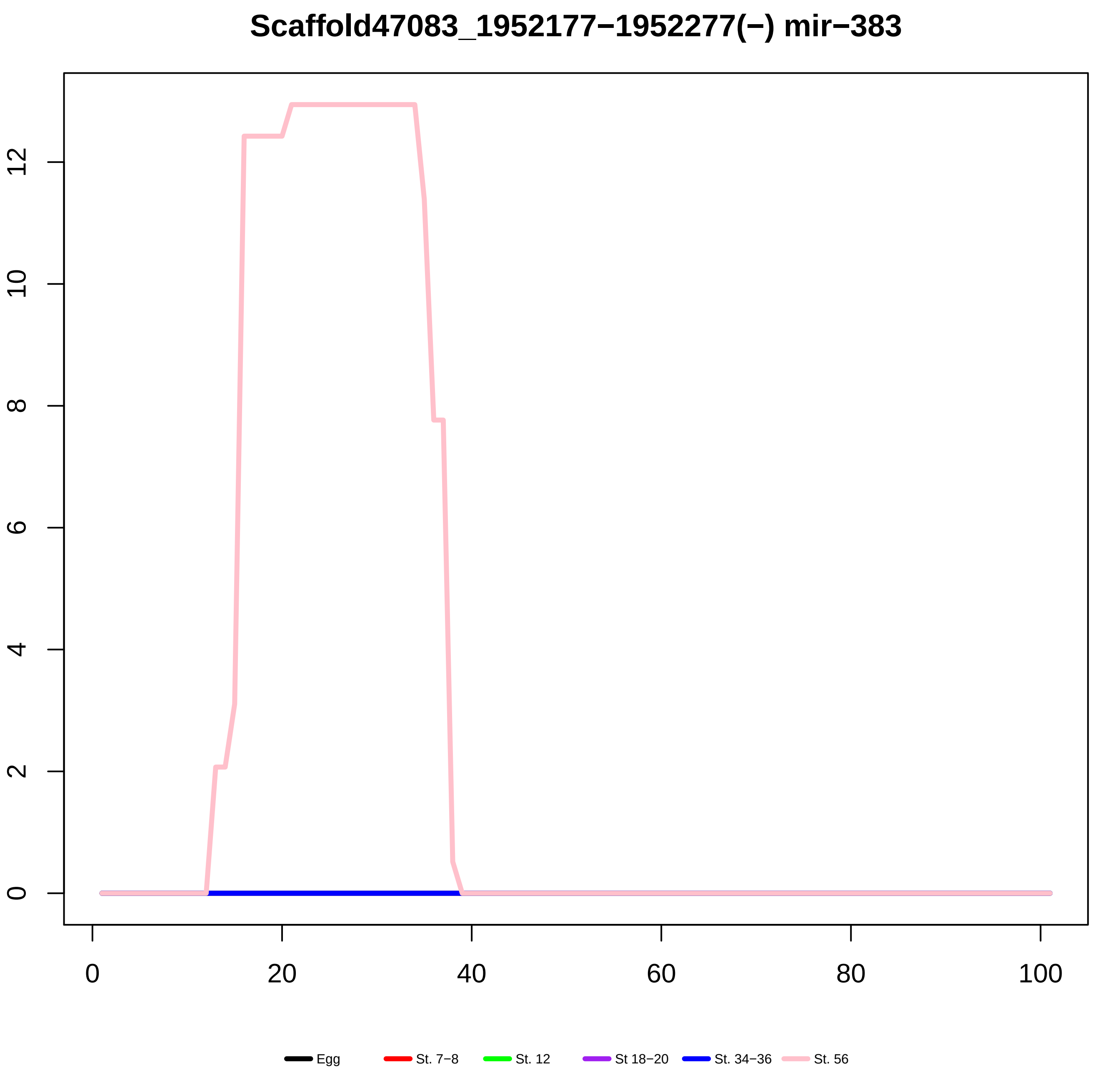
Scaffold47083_1952177−1952277(−) mir−383
0
2
4
6
8
10
12
0
20
40
60
80
100
Egg
St. 7−8
St. 12
St 18−20
St. 34−36
St. 56

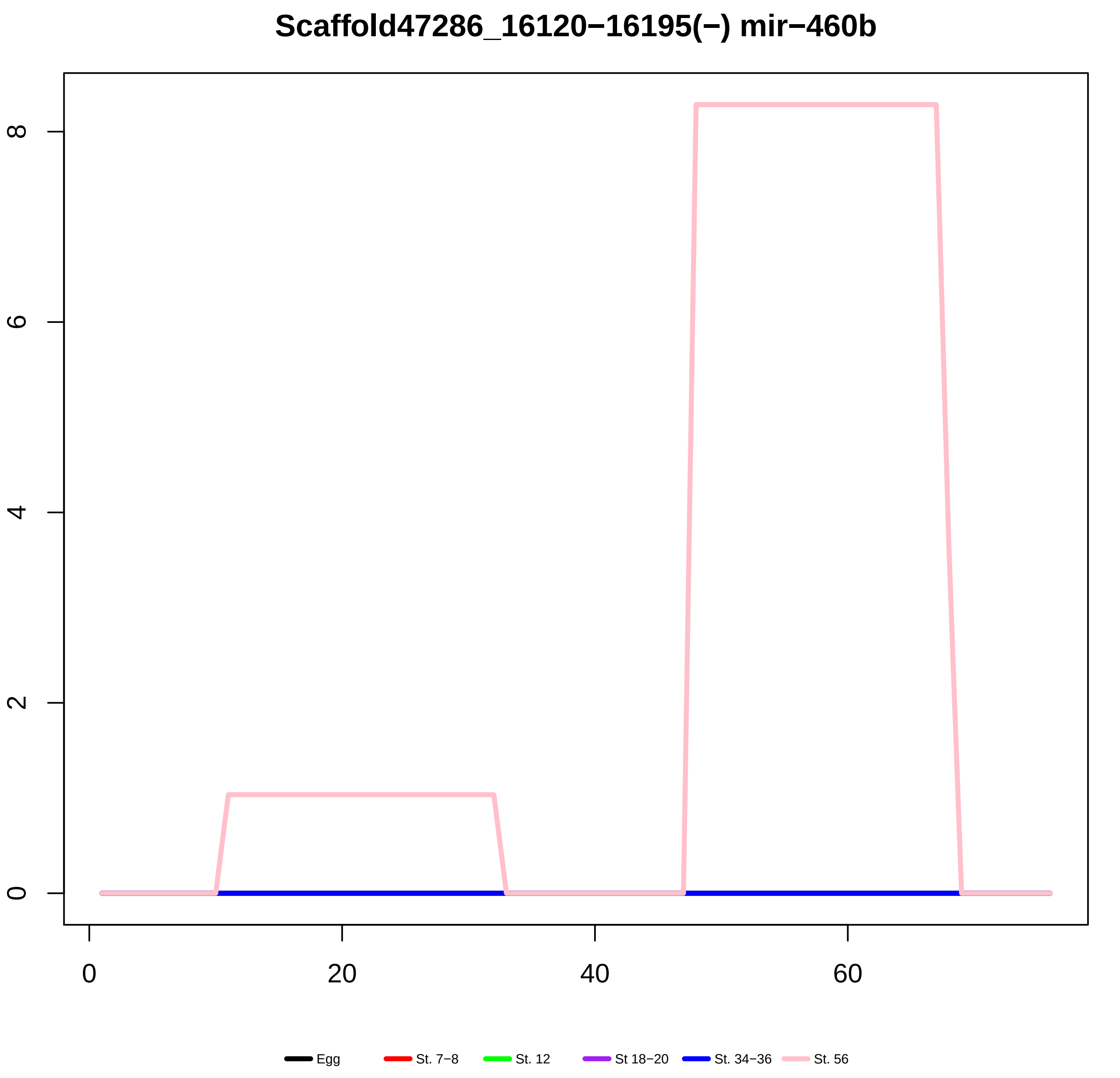

Scaffold47286_16120−16195(−) mir−460b
0
2
4
6
8
0
20
40
60
Egg
St. 7−8
St. 12
St 18−20
St. 34−36
St. 56

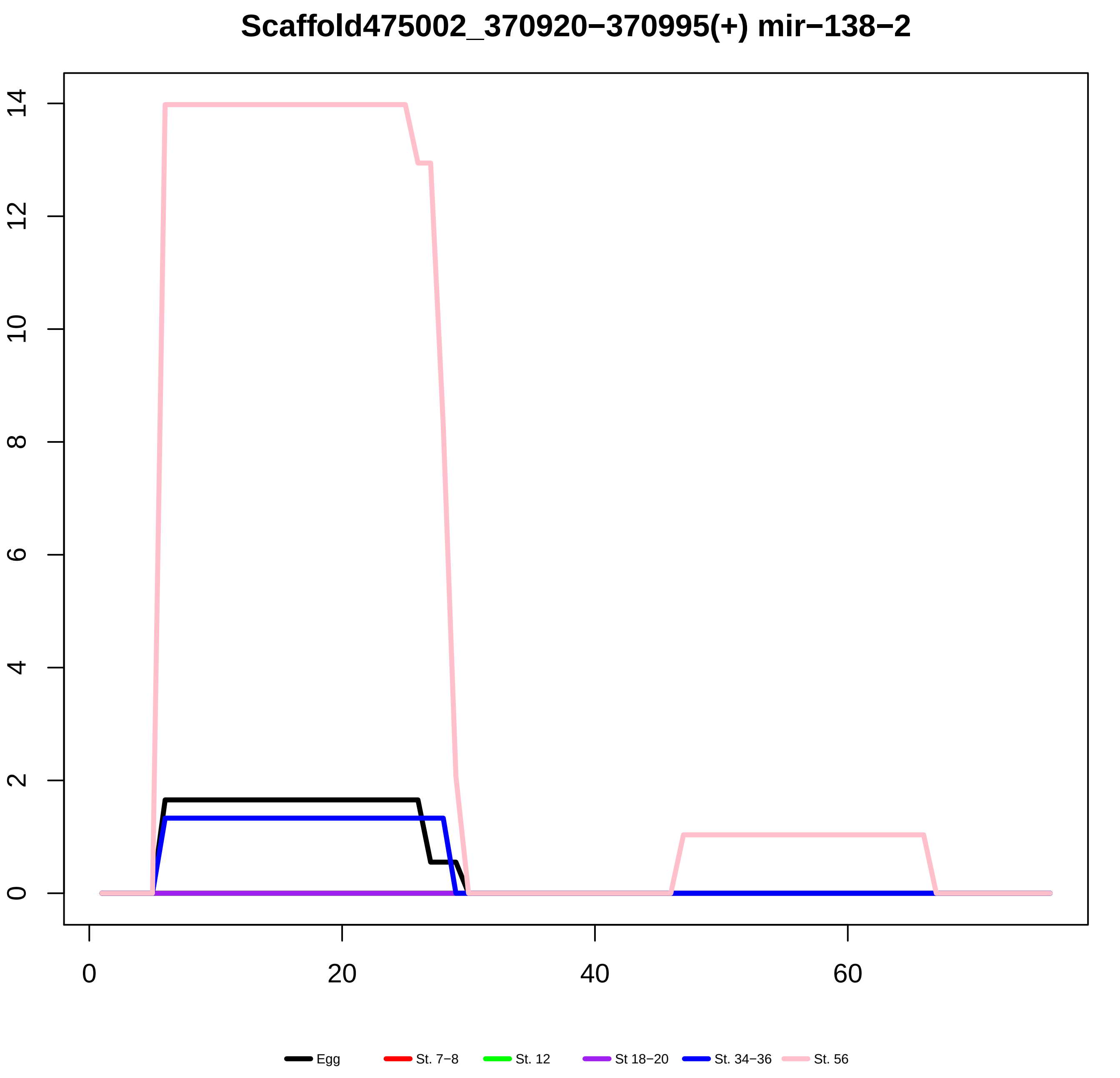
Scaffold475002_370920−370995(+) mir−138−2
0
2
4
6
8
10
12
14
0
20
40
60
Egg
St. 7−8
St. 12
St 18−20
St. 34−36
St. 56

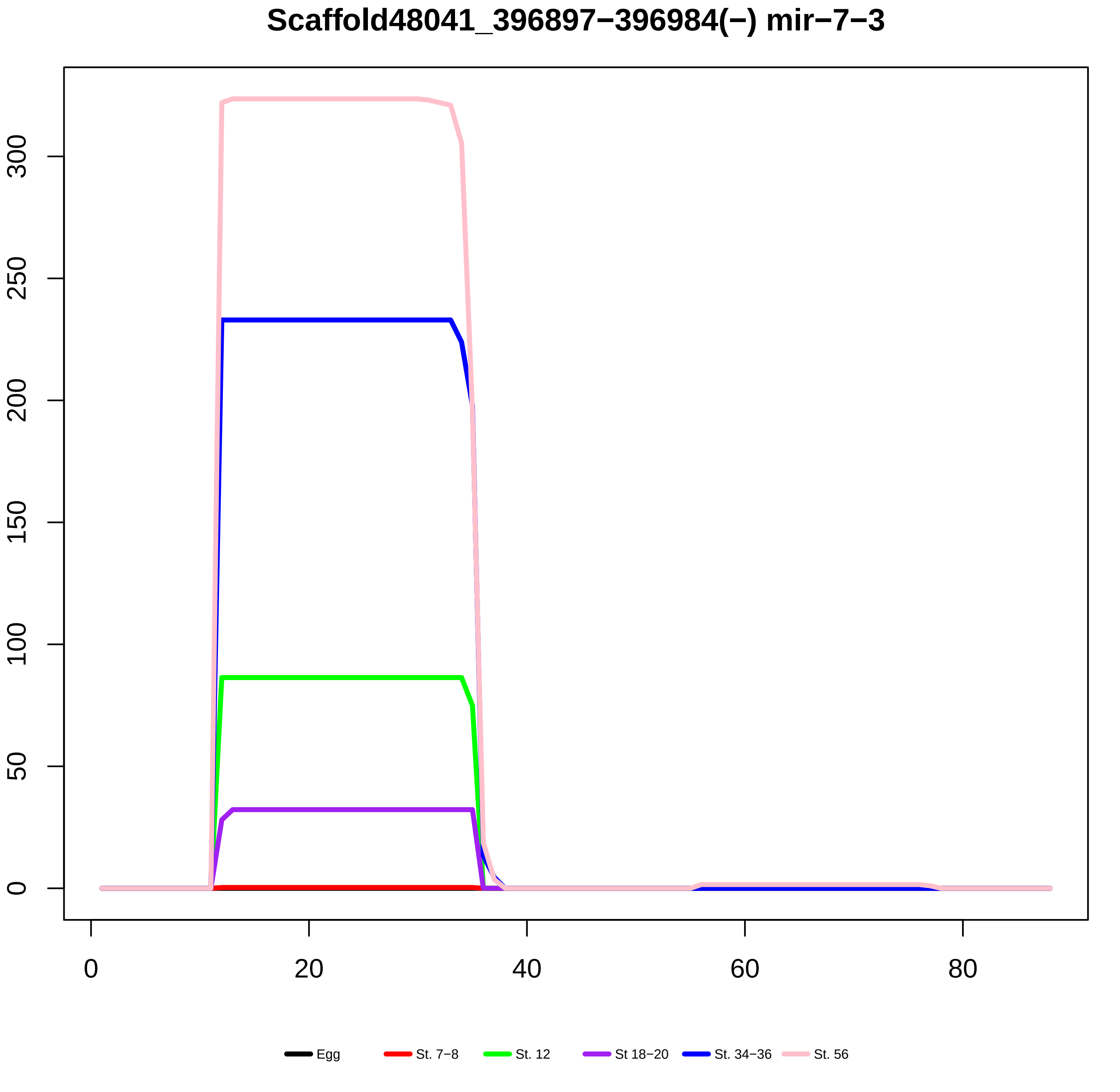

Scaffold48041_396897−396984(−) mir−7−3
0
50
100
150
200
250
300
0
20
40
60
80
Egg
St. 7−8
St. 12
St 18−20
St. 34−36
St. 56

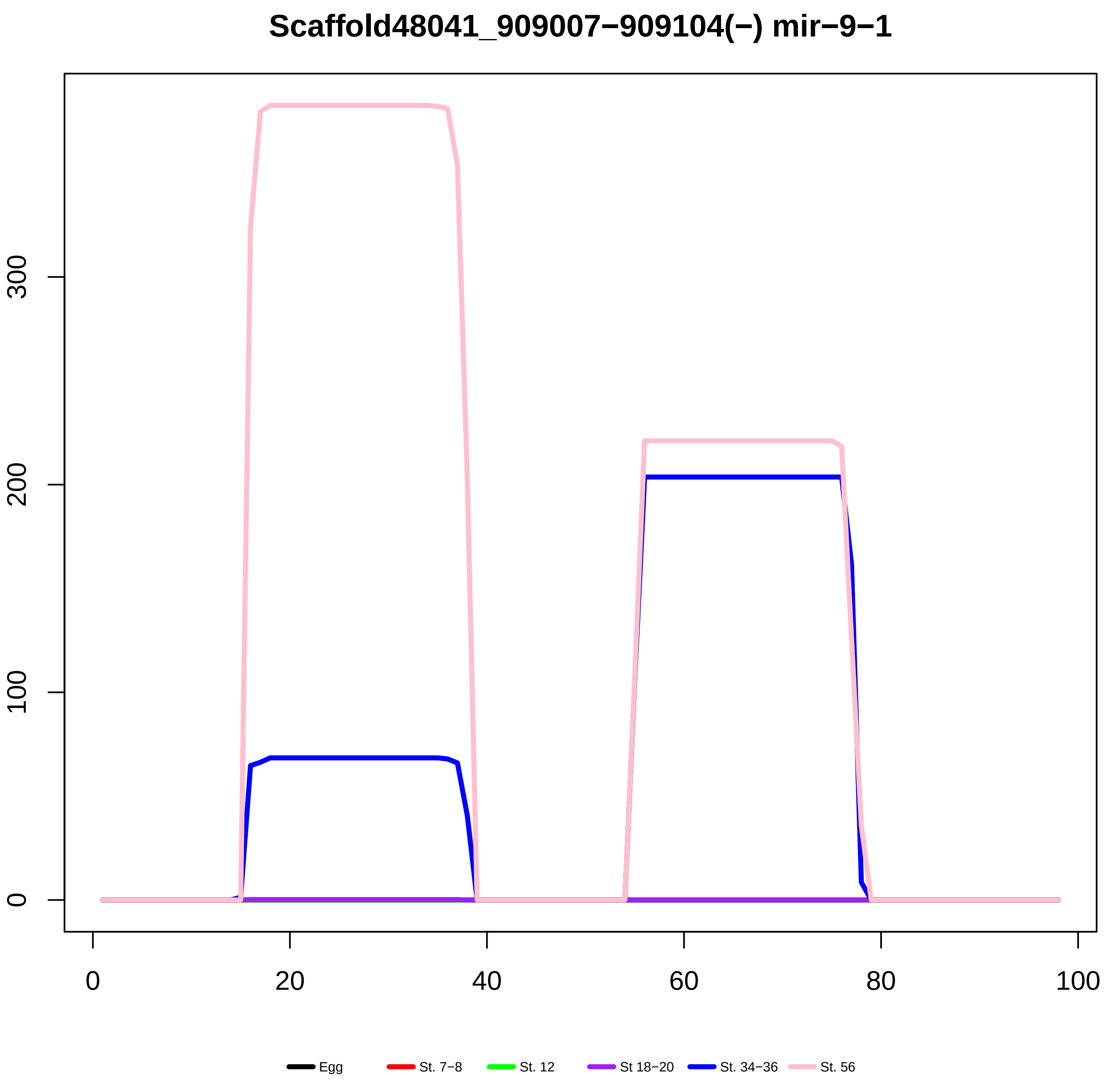
Scaffold48041_909007−909104(−) mir−9−1
0
100
200
300
0
20
40
60
80
100
Egg
St. 7−8
St. 12
St 18−20
St. 34−36
St. 56

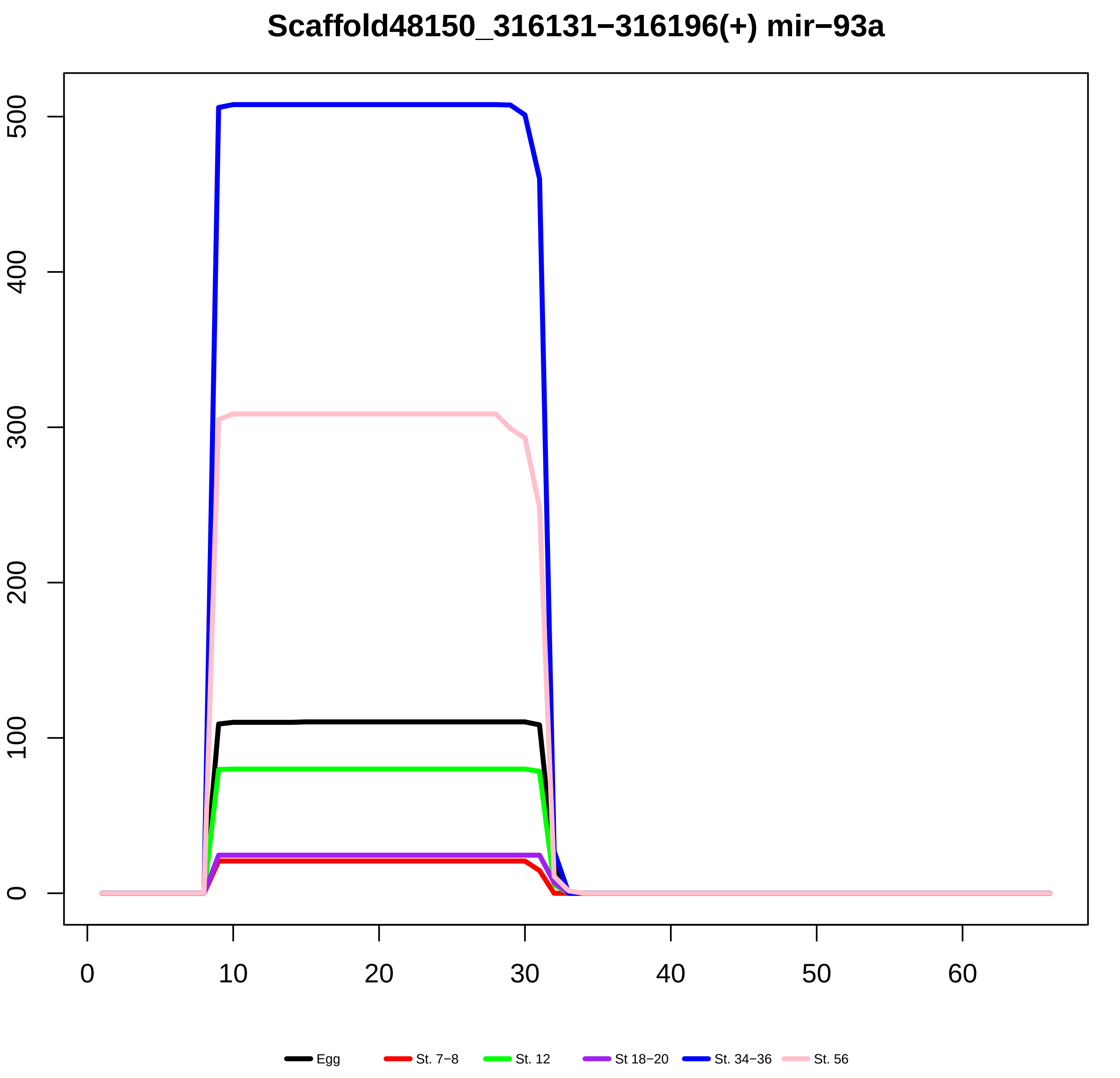

Scaffold48150_316131−316196(+) mir−93a
0
100
200
300
400
500
0
10
20
30
40
50
60
Egg
St. 7−8
St. 12
St 18−20
St. 34−36
St. 56

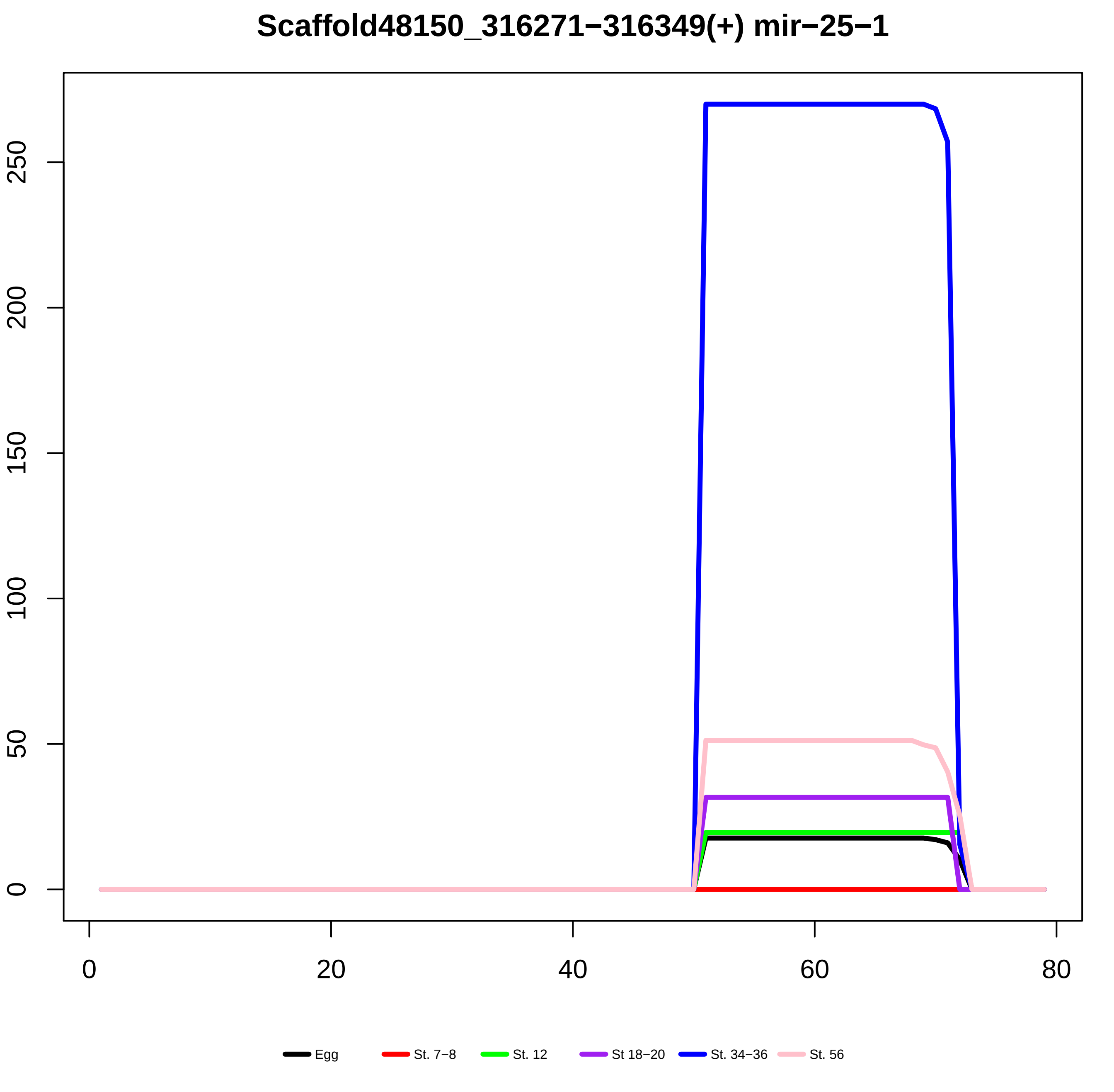
Scaffold48150_316271−316349(+) mir−25−1
0
50
100
150
200
250
0
20
40
60
80
Egg
St. 7−8
St. 12
St 18−20
St. 34−36
St. 56

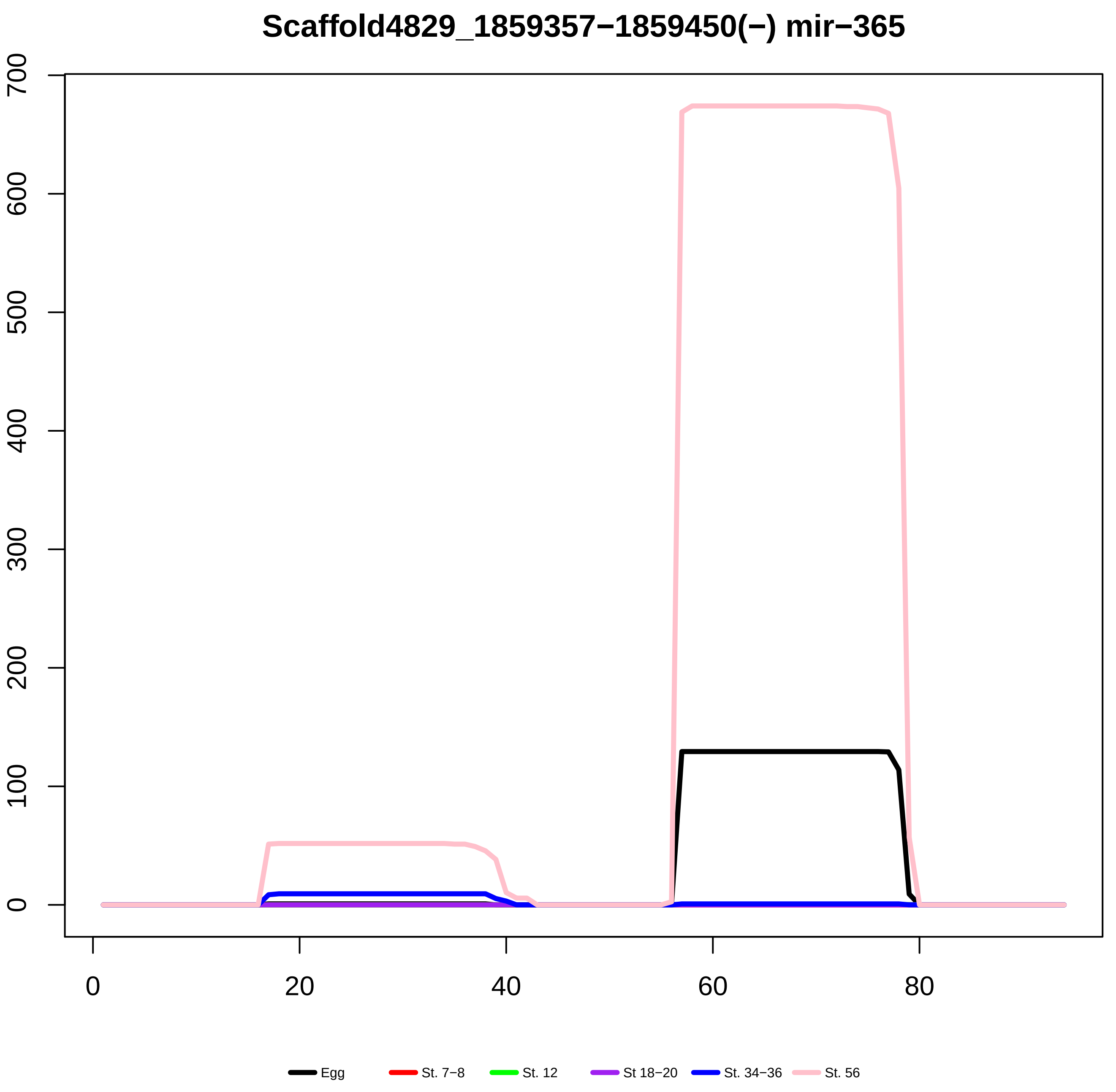

Scaffold4829_1859357−1859450(−) mir−365
0
100
200
300
400
500
600
700
0
20
40
60
80
Egg
St. 7−8
St. 12
St 18−20
St. 34−36
St. 56

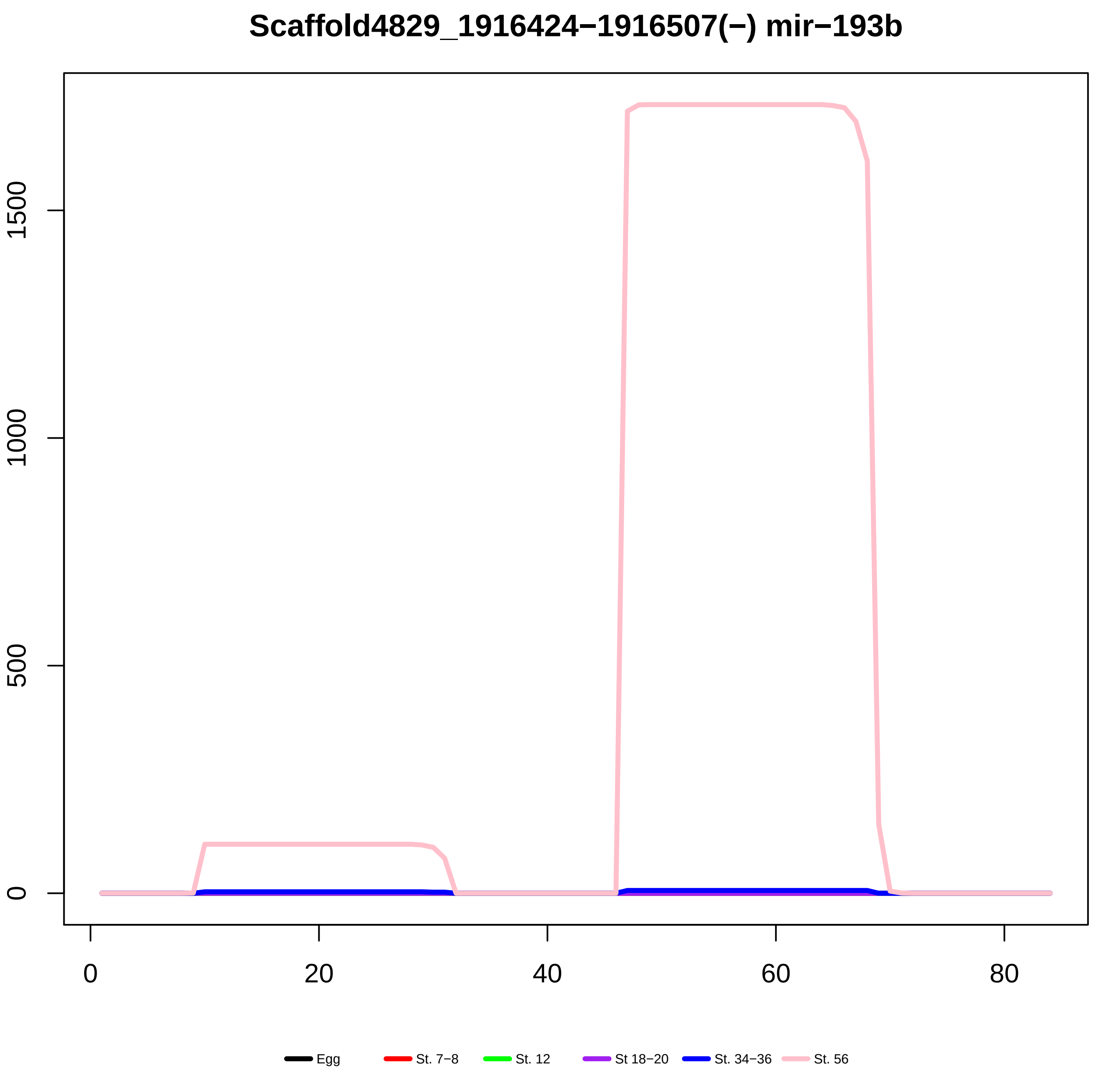

Scaffold4829_1916424−1916507(−) mir−193b
0
500
1000
1500
0
20
40
60
80
Egg
St. 7−8
St. 12
St 18−20
St. 34−36
St. 56

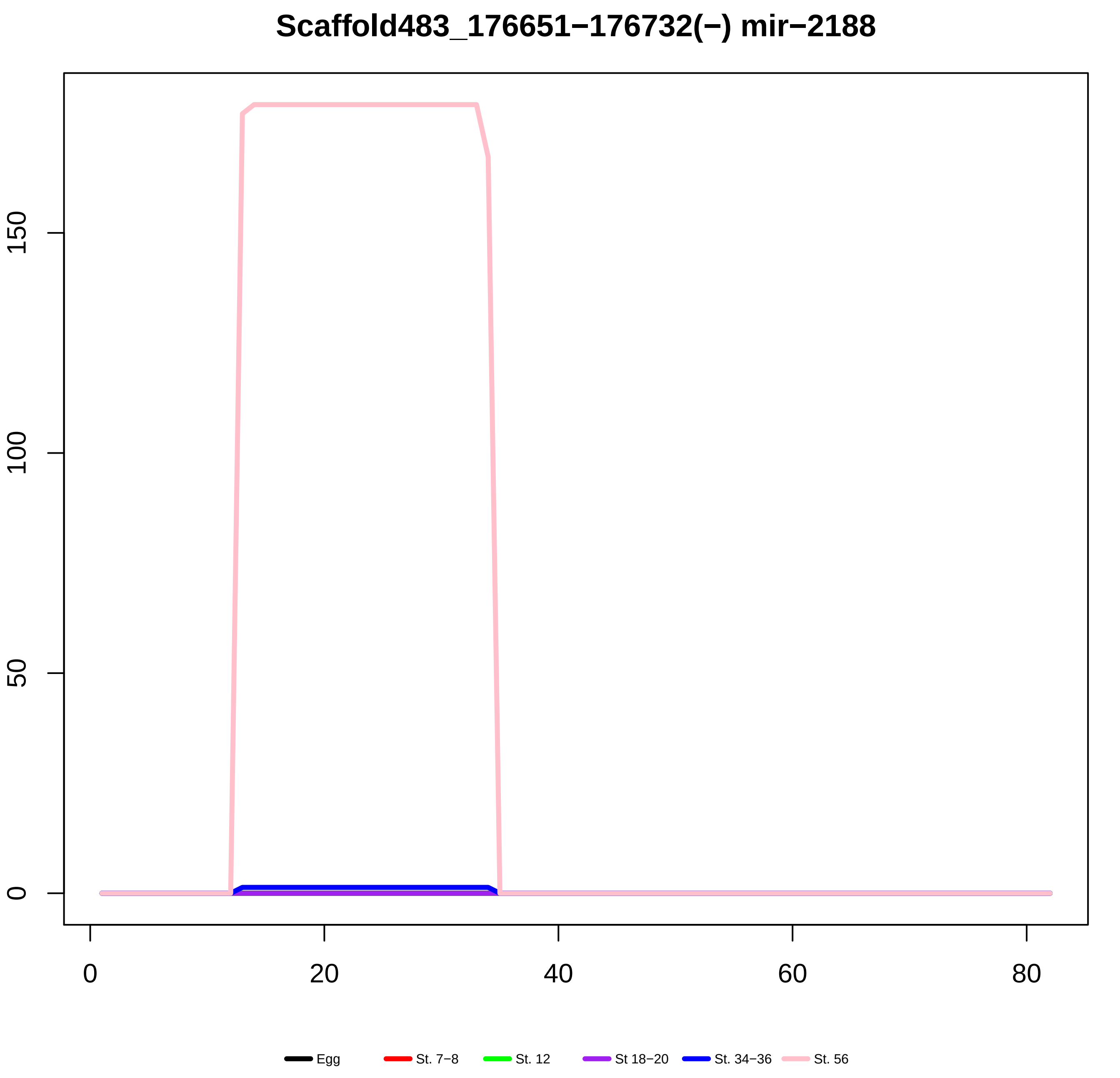

Scaffold483_176651−176732(−) mir−2188
0
50
100
150
0
20
40
60
80
Egg
St. 7−8
St. 12
St 18−20
St. 34−36
St. 56

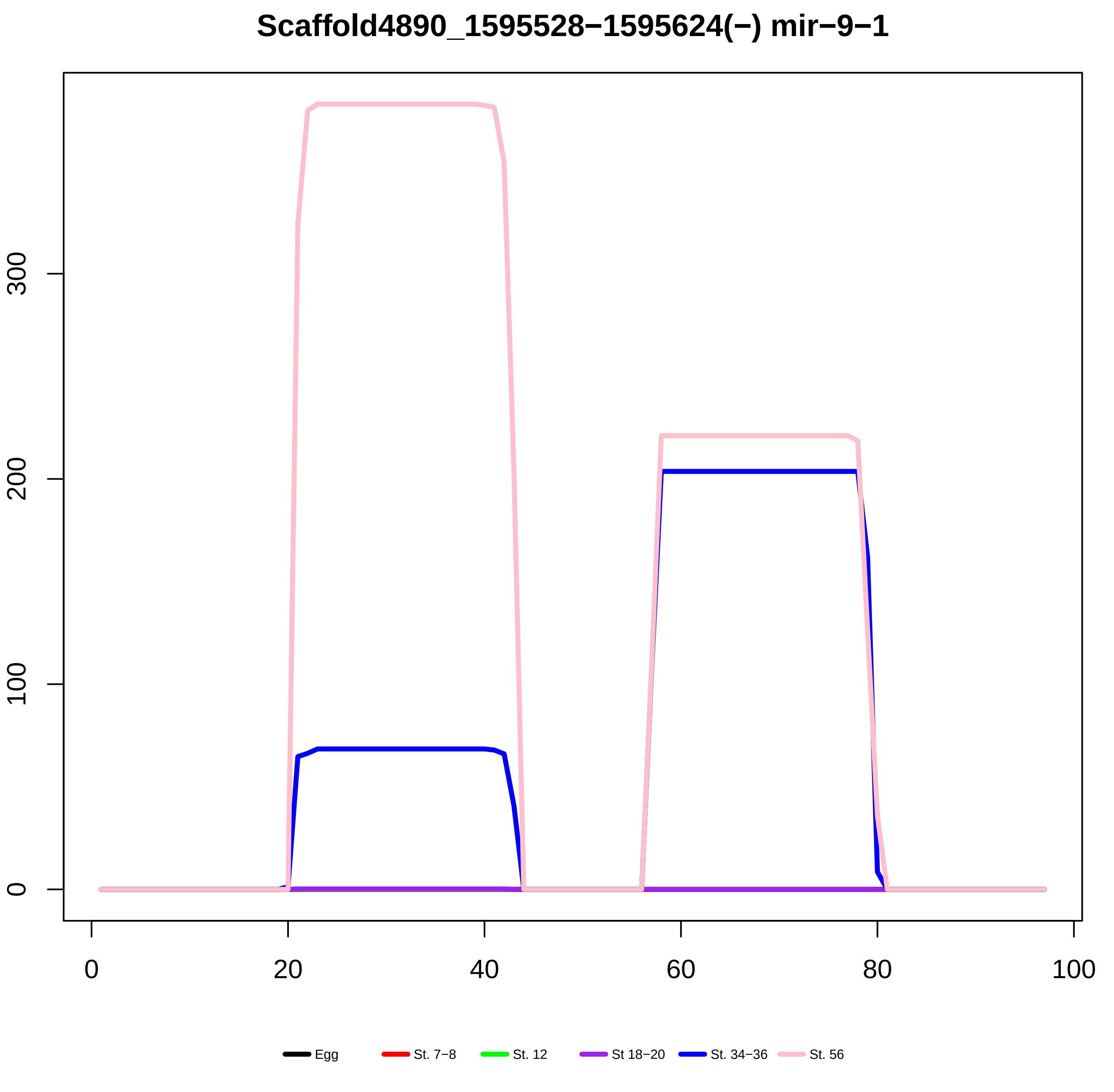

Scaffold4890_1595528−1595624(−) mir−9−1
0
100
200
300
0
20
40
60
80
100
Egg
St. 7−8
St. 12
St 18−20
St. 34−36
St. 56

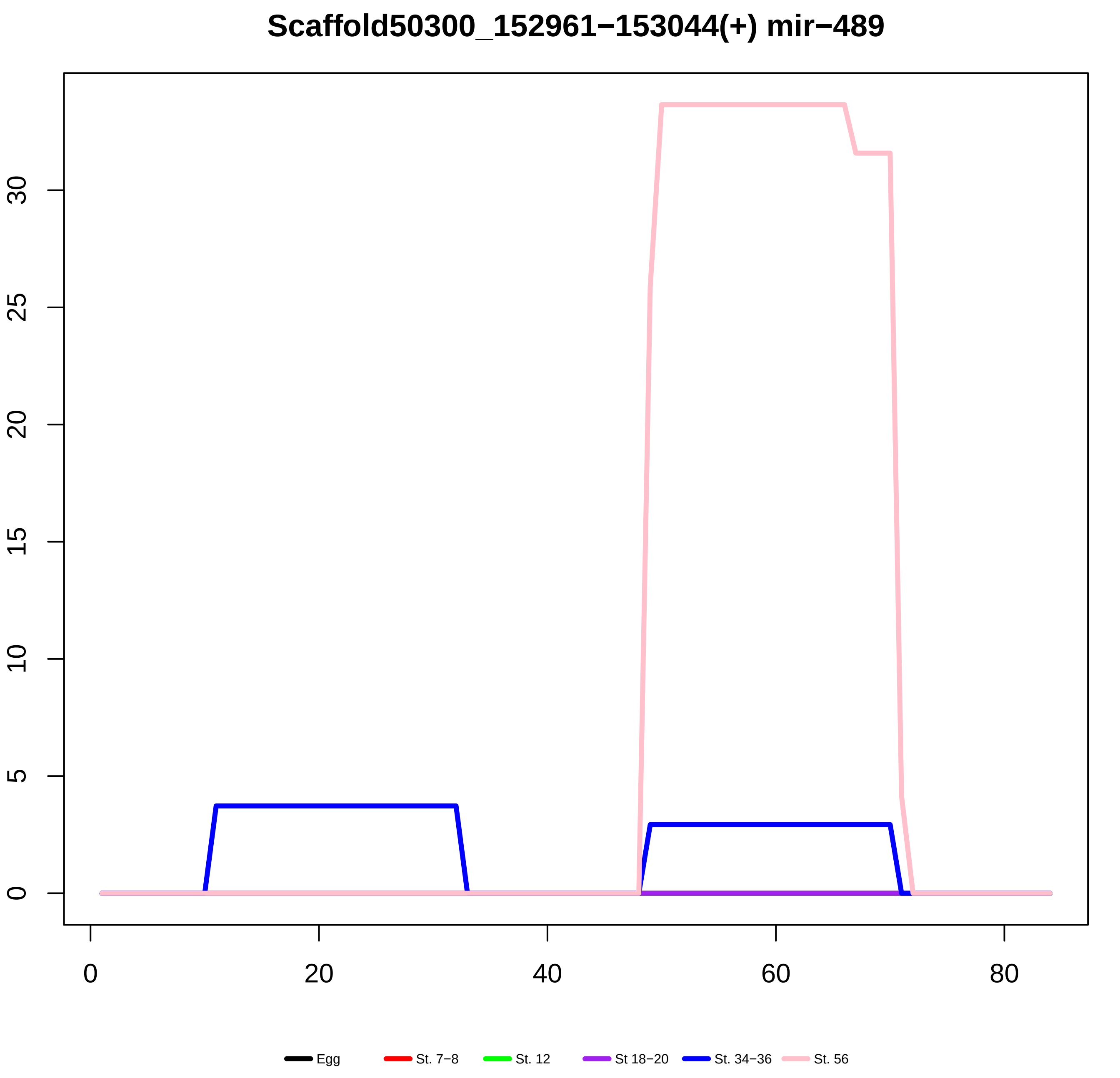

Scaffold50300_152961−153044(+) mir−489
0
5
10
15
20
25
30
0
20
40
60
80
Egg
St. 7−8
St. 12
St 18−20
St. 34−36
St. 56

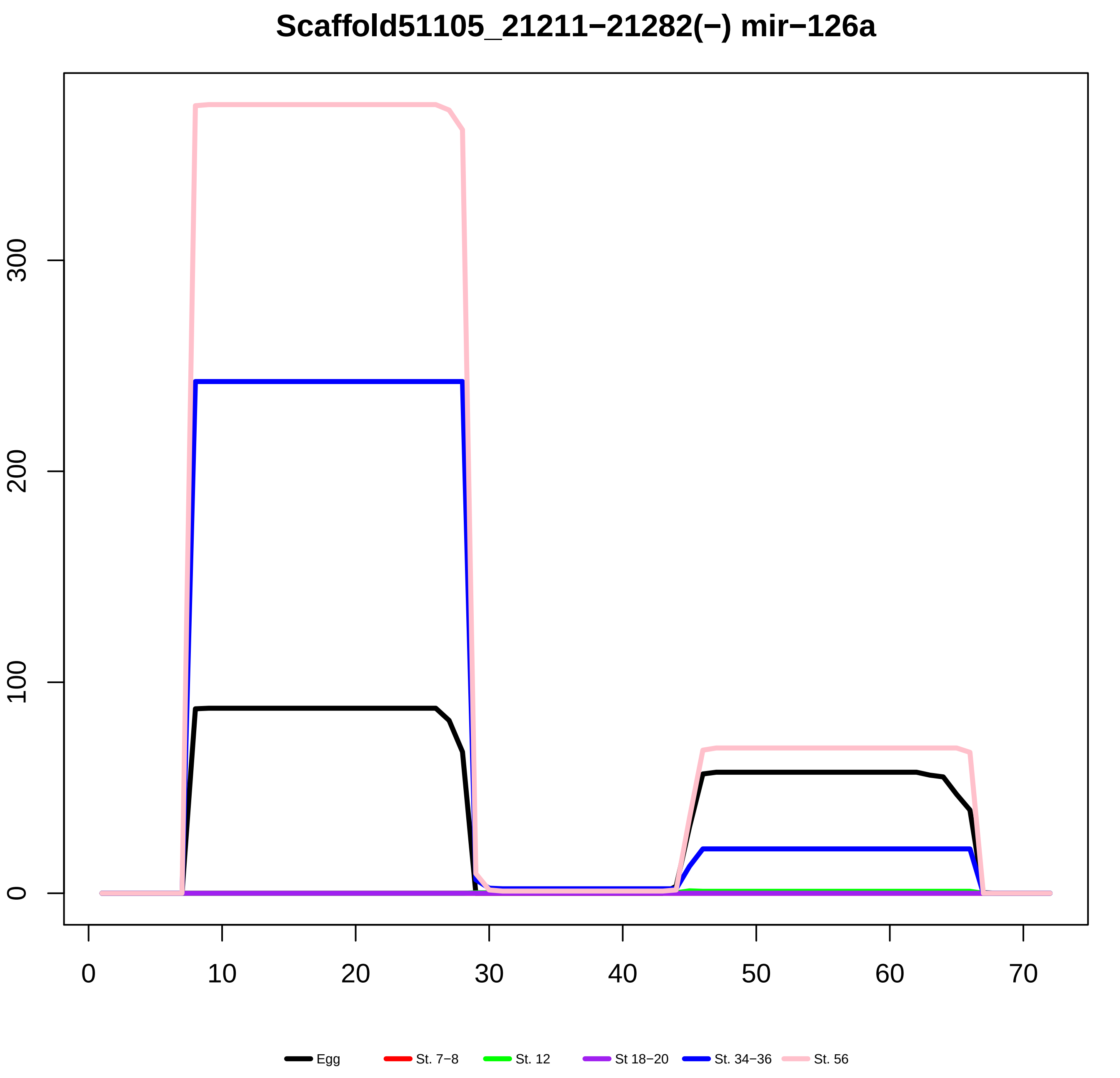

Scaffold51105_21211−21282(−) mir−126a
0
100
200
300
0
10
20
30
40
50
60
70
Egg
St. 7−8
St. 12
St 18−20
St. 34−36
St. 56

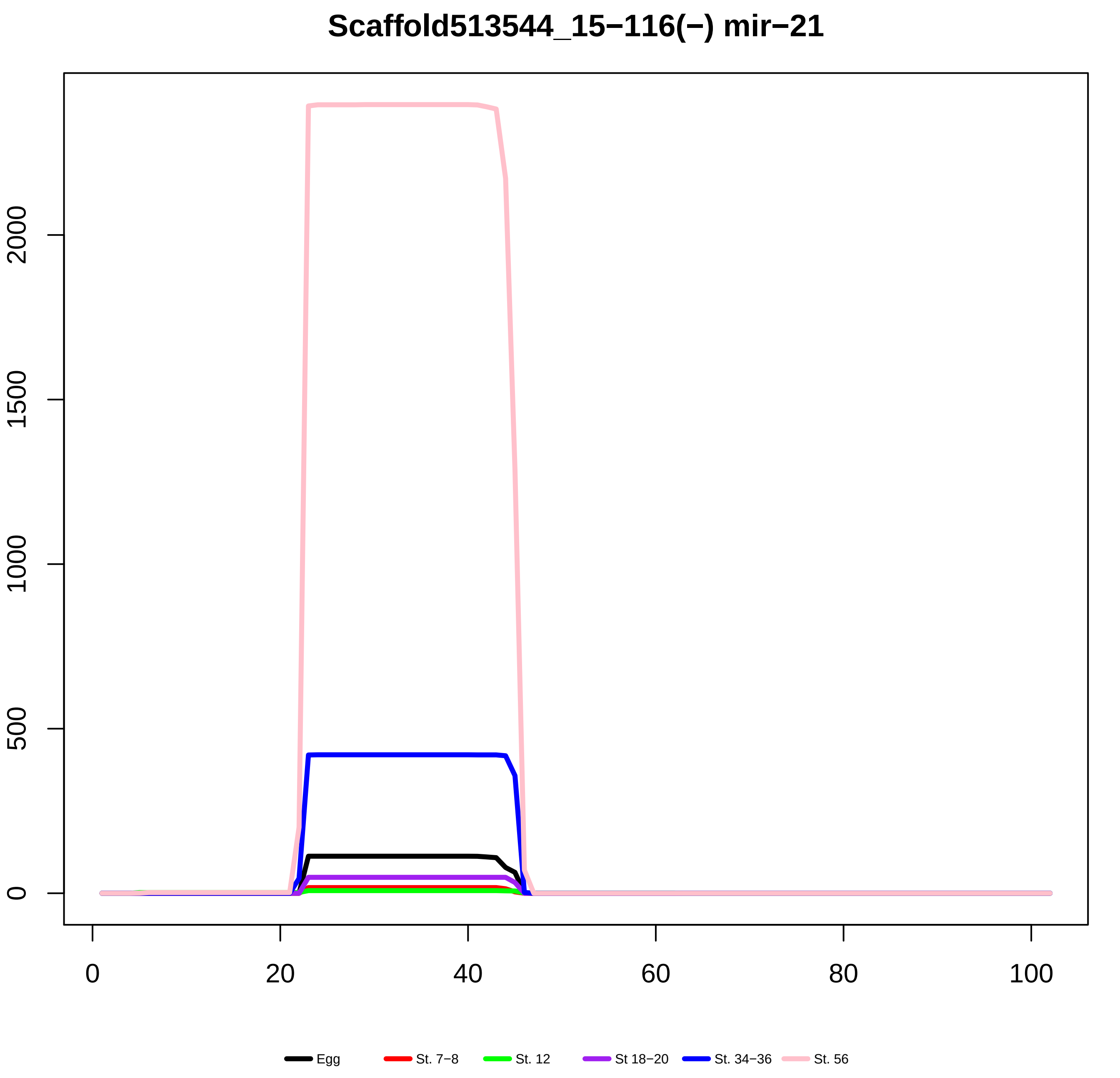
Scaffold513544_15−116(−) mir−21
0
500
1000
1500
2000
0
20
40
60
80
100
Egg
St. 7−8
St. 12
St 18−20
St. 34−36
St. 56

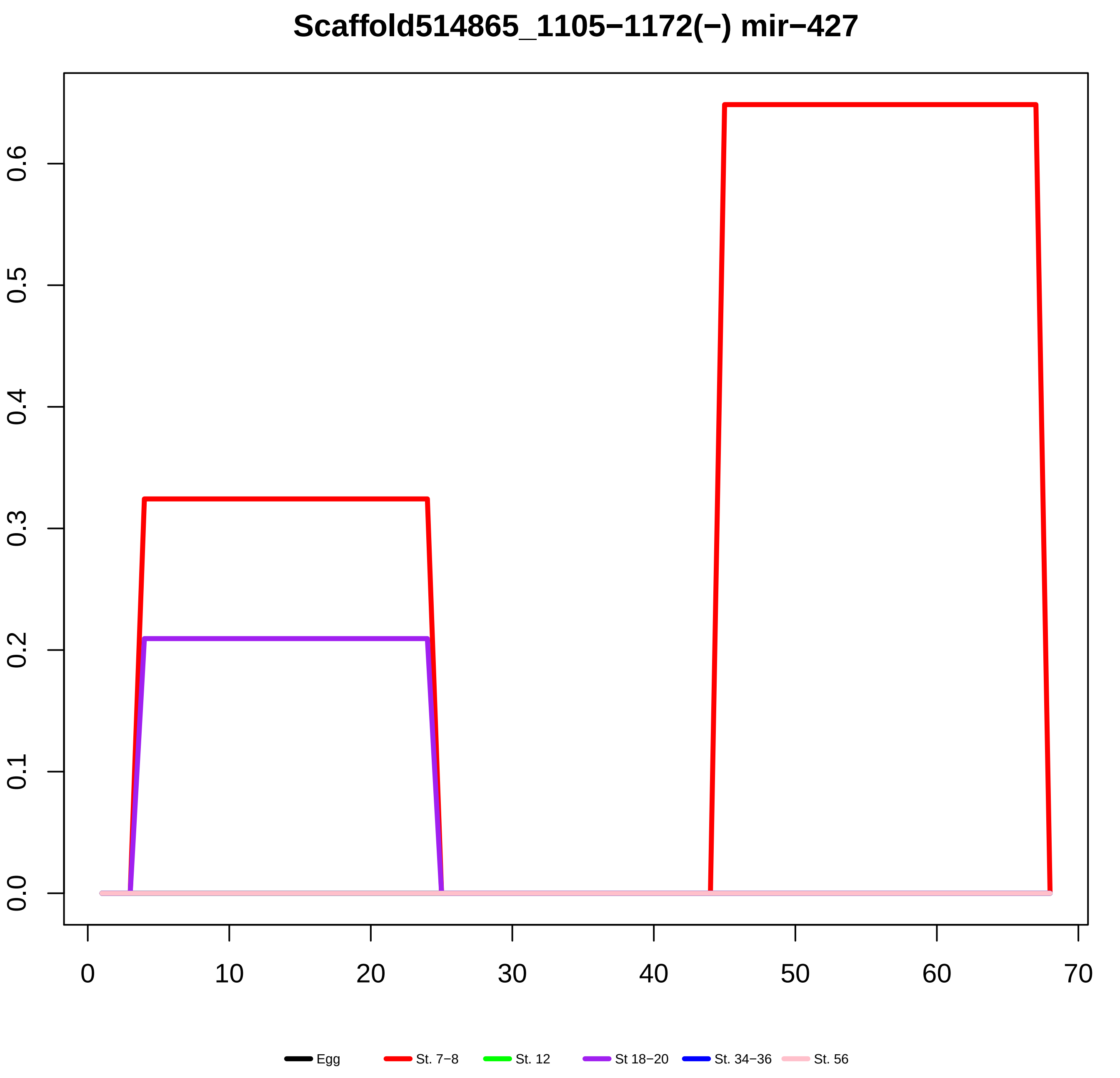

Scaffold514865_1105−1172(−) mir−427
0.0
0.1
0.2
0.3
0.4
0.5
0.6
0
10
20
30
40
50
60
70
Egg
St. 7−8
St. 12
St 18−20
St. 34−36
St. 56

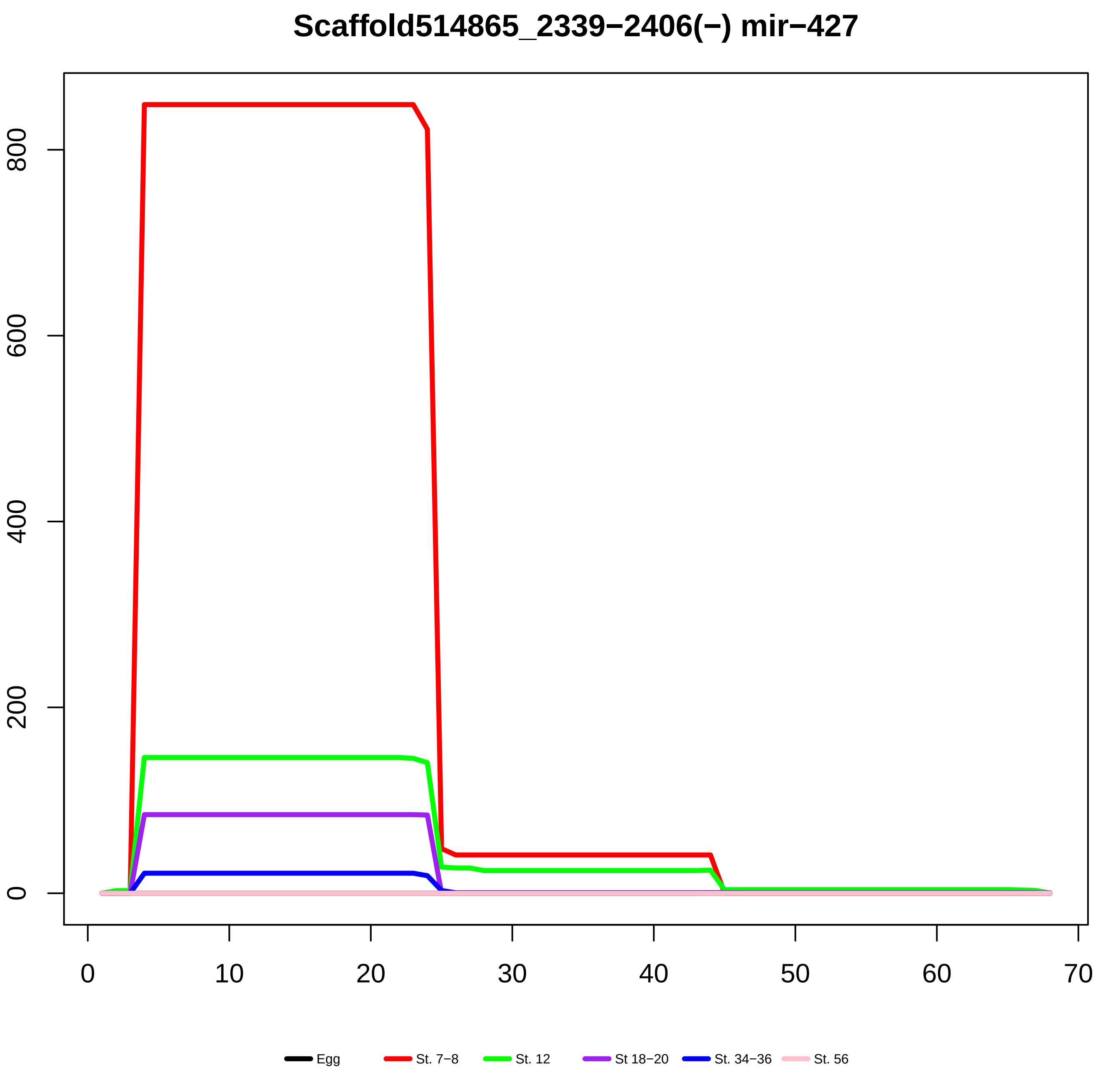

Scaffold514865_2339−2406(−) mir−427
0
200
400
600
800
0
10
20
30
40
50
60
70
Egg
St. 7−8
St. 12
St 18−20
St. 34−36
St. 56

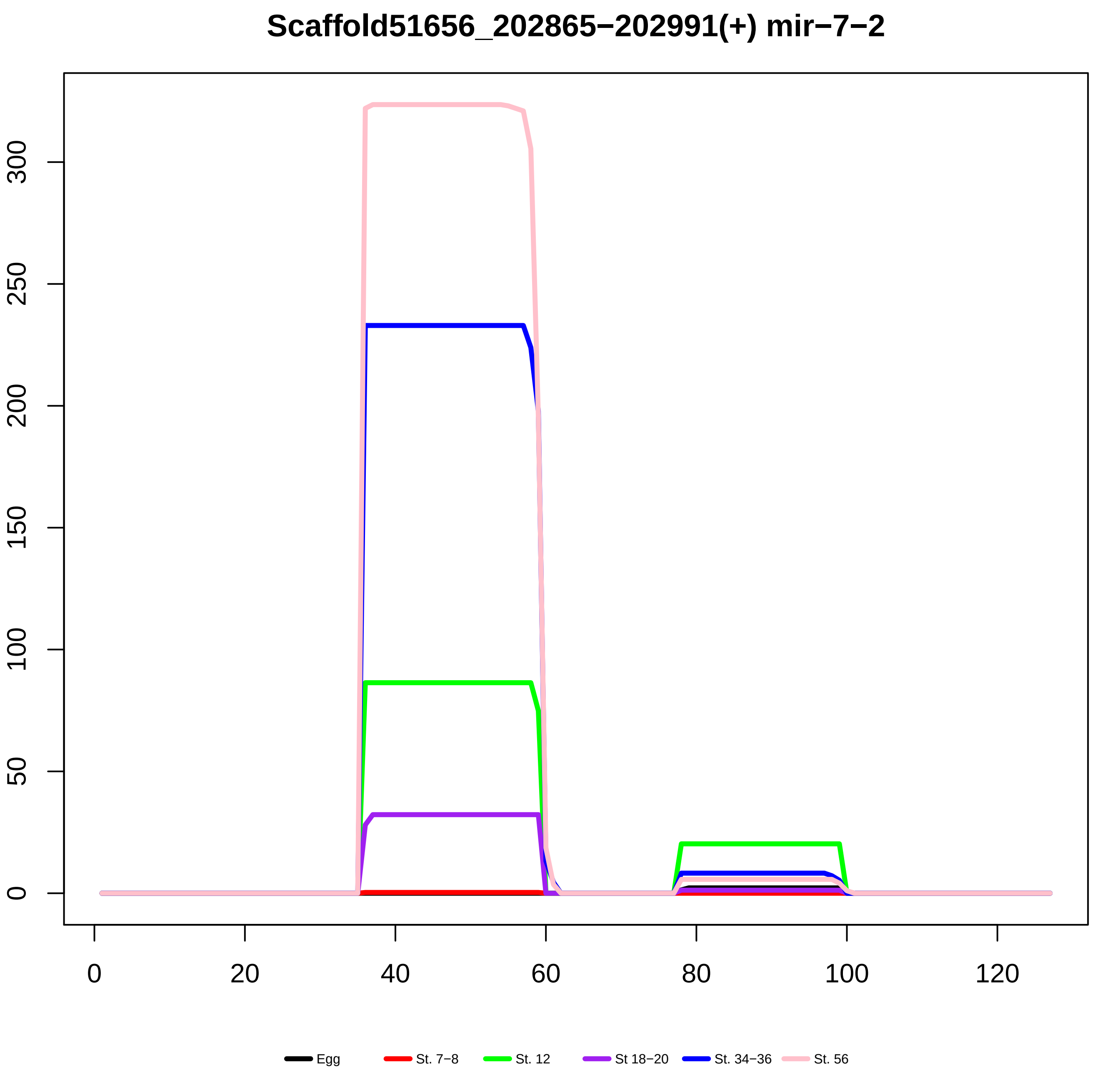
Scaffold51656_202865−202991(+) mir−7−2
0
50
100
150
200
250
300
0
20
40
60
80
100
120
Egg
St. 7−8
St. 12
St 18−20
St. 34−36
St. 56

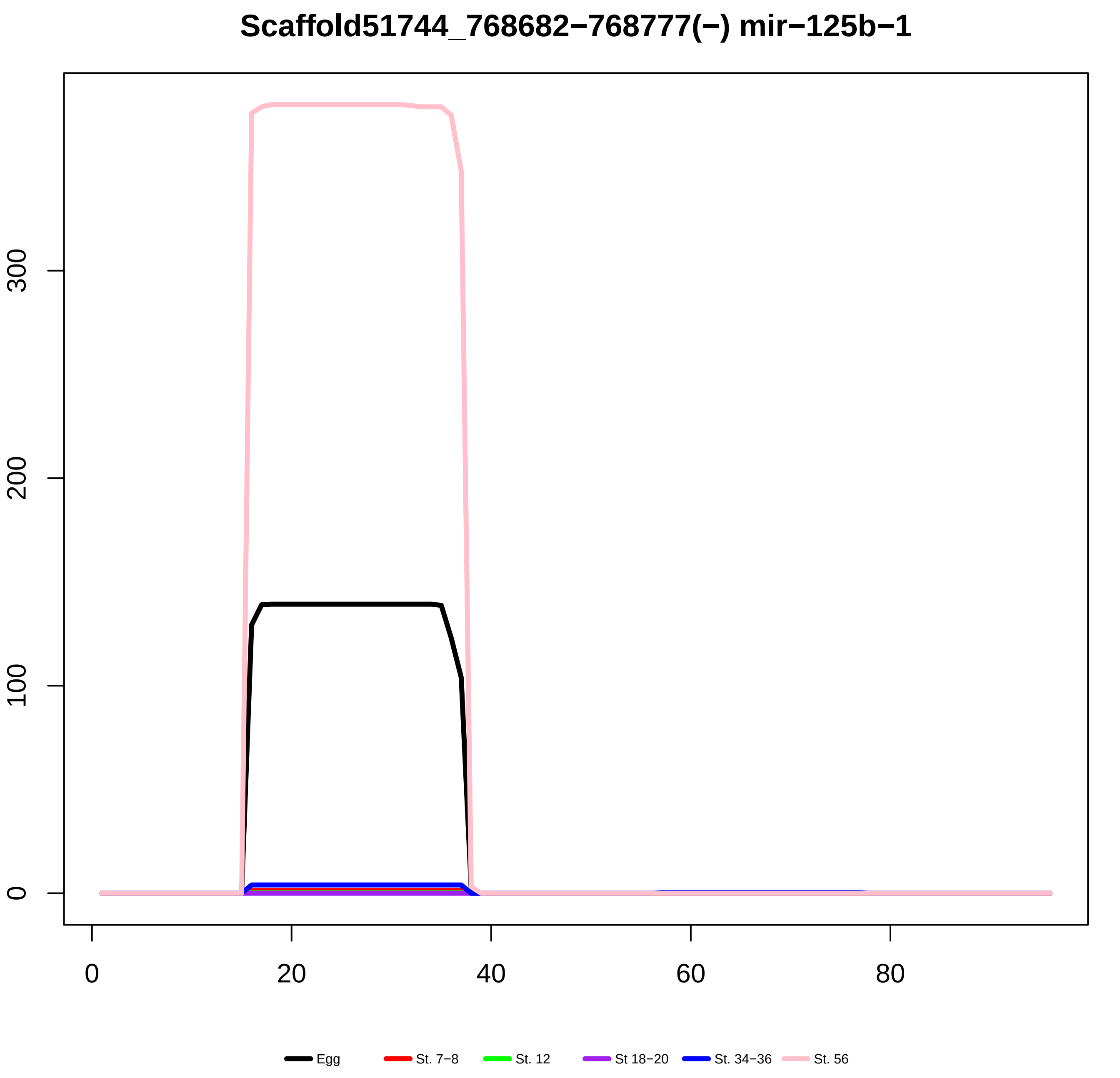

Scaffold51744_768682−768777(−) mir−125b−1
0
100
200
300
0
20
40
60
80
Egg
St. 7−8
St. 12
St 18−20
St. 34−36
St. 56

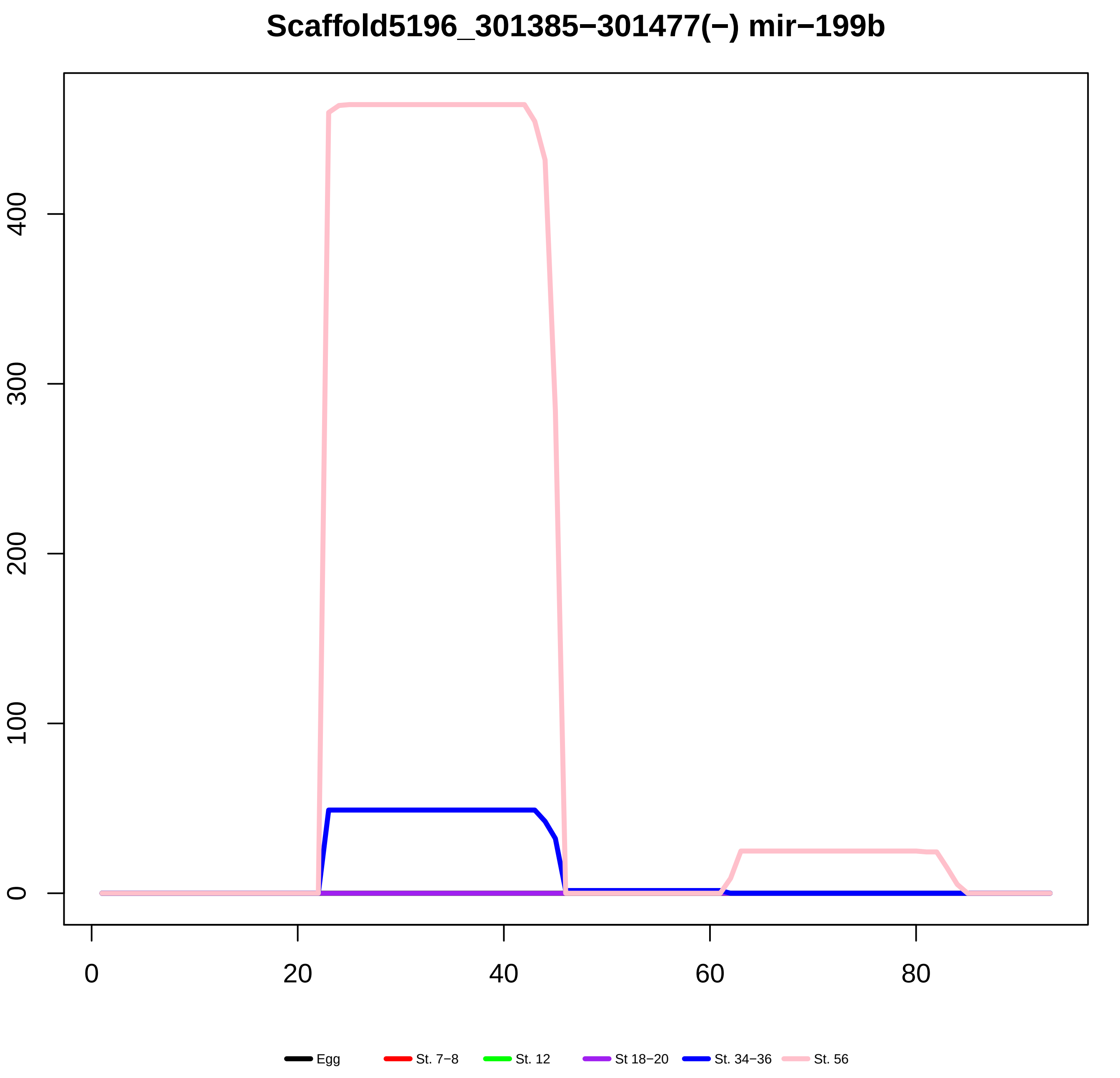
Scaffold5196_301385−301477(−) mir−199b
0
100
200
300
400
0
20
40
60
80
Egg
St. 7−8
St. 12
St 18−20
St. 34−36
St. 56

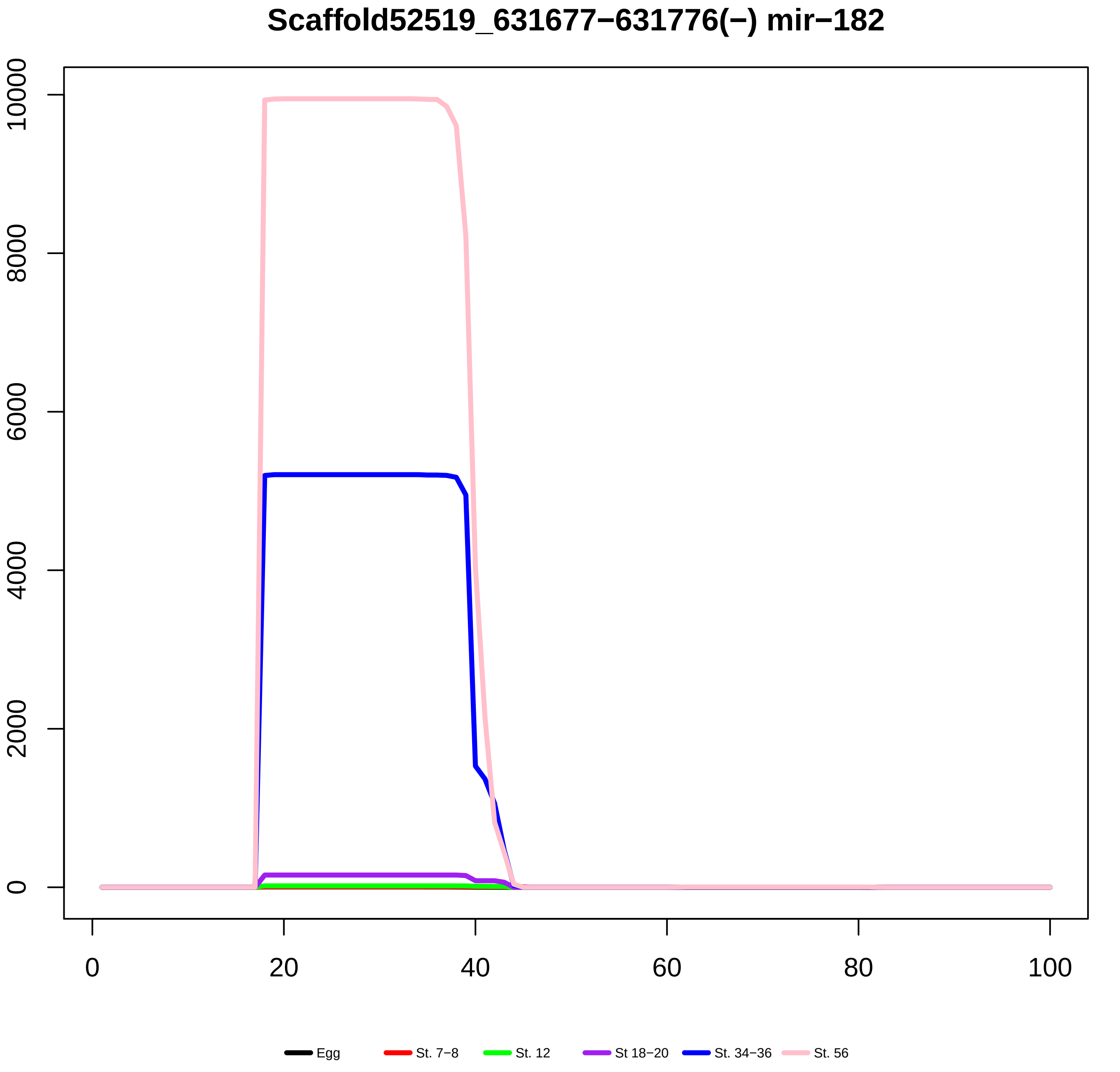

Scaffold52519_631677−631776(−) mir−182
10000
8000
6000
4000
2000
0
0
20
40
60
80
100
Egg
St. 7−8
St. 12
St 18−20
St. 34−36
St. 56

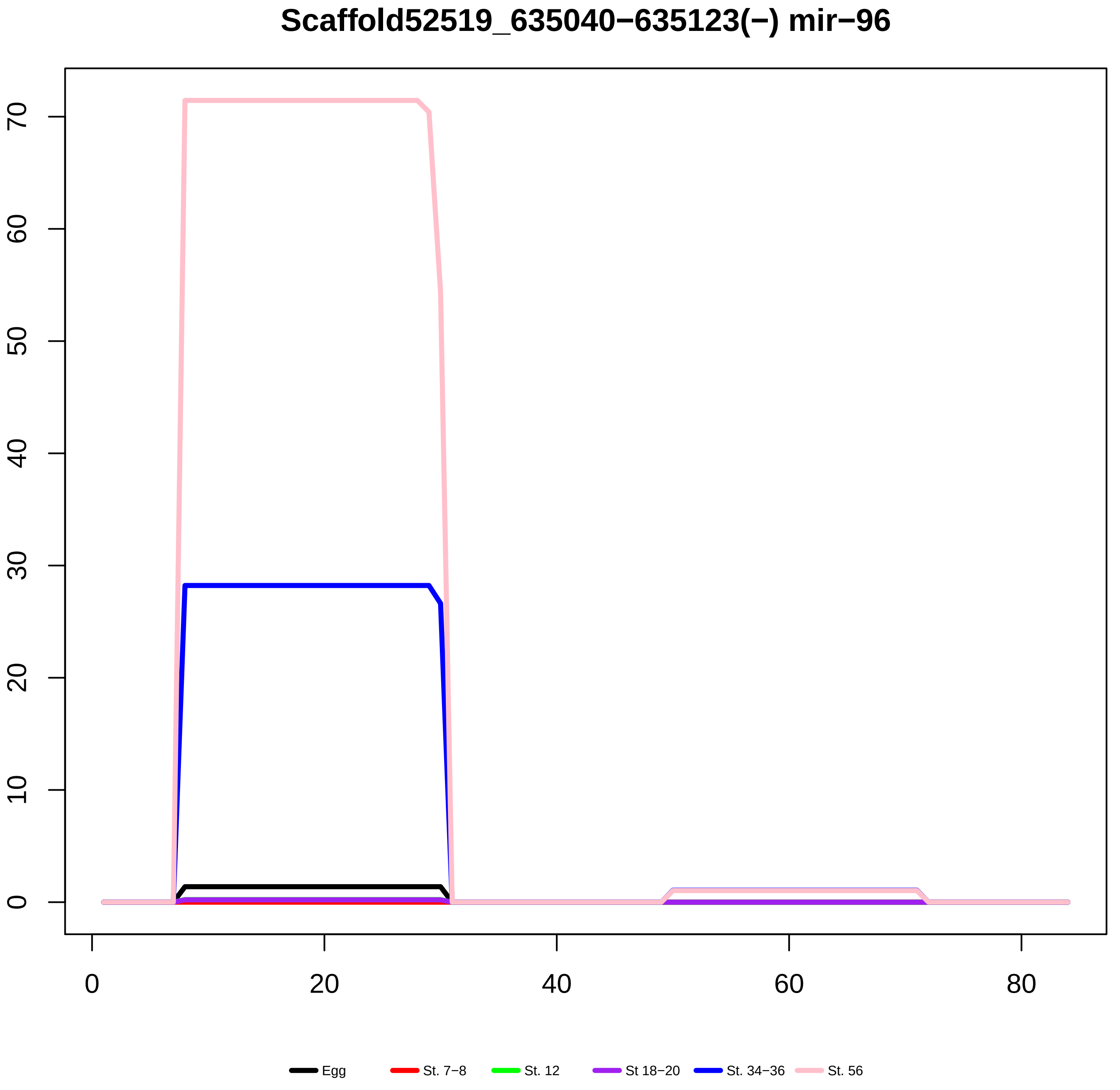

Scaffold52519_635040−635123(−) mir−96
0
10
20
30
40
50
60
70
0
20
40
60
80
Egg
St. 7−8
St. 12
St 18−20
St. 34−36
St. 56

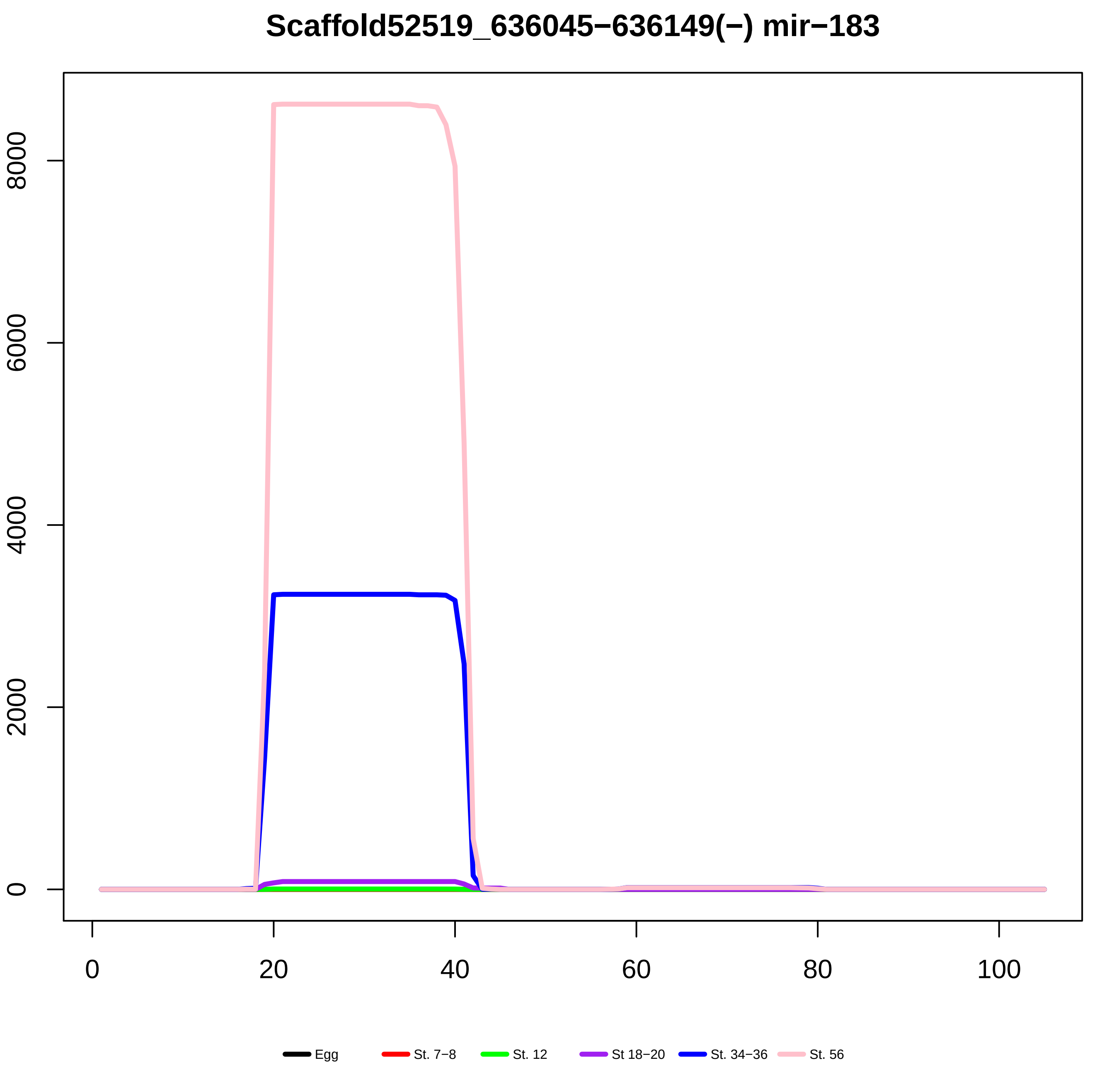
Scaffold52519_636045−636149(−) mir−183
0
2000
4000
6000
8000
0
20
40
60
80
100
Egg
St. 7−8
St. 12
St 18−20
St. 34−36
St. 56

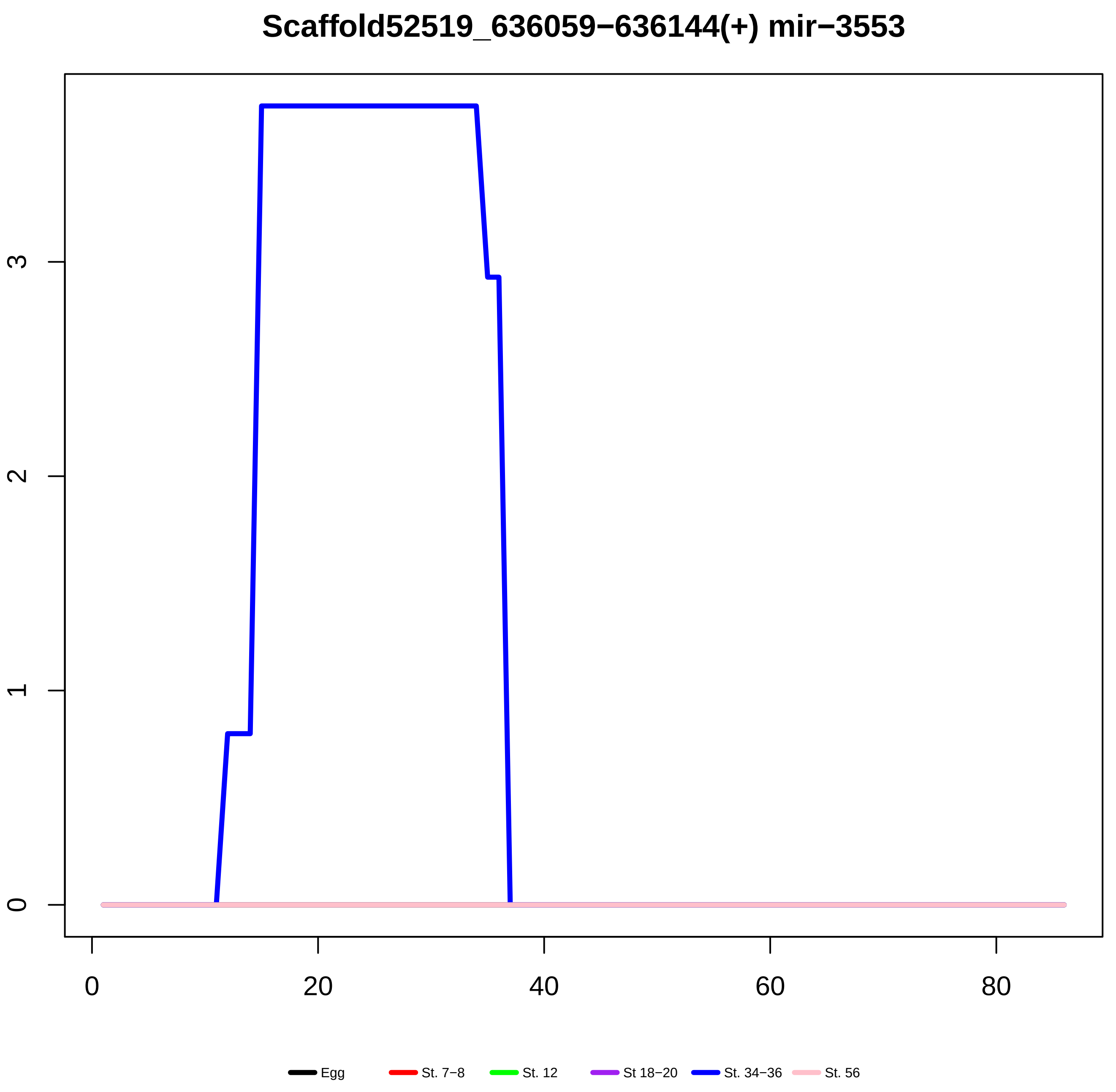

Scaffold52519_636059−636144(+) mir−3553
0
1
2
3
0
20
40
60
80
Egg
St. 7−8
St. 12
St 18−20
St. 34−36
St. 56

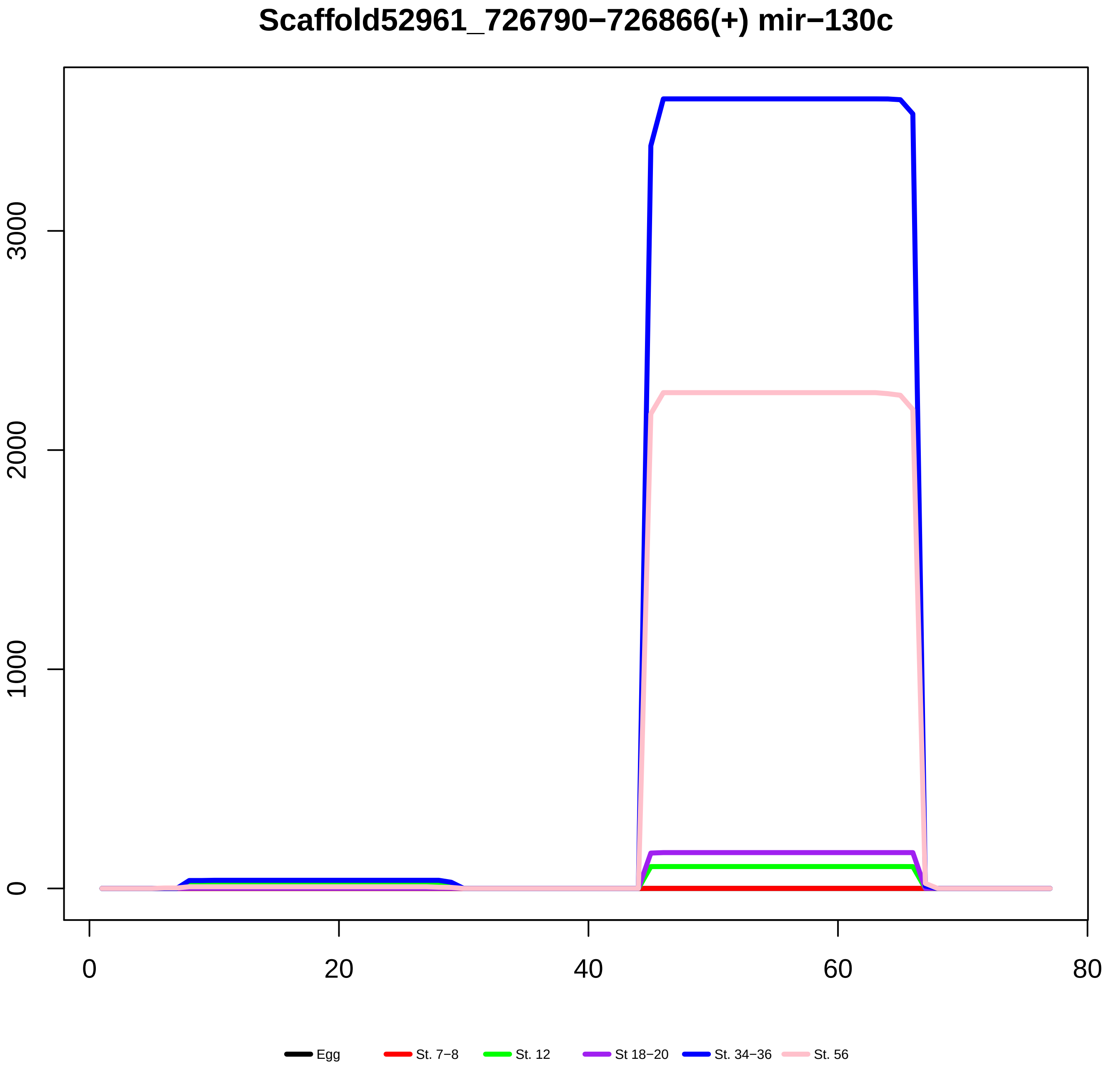
Scaffold52961_726790−726866(+) mir−130c
0
1000
2000
3000
0
20
40
60
80
Egg
St. 7−8
St. 12
St 18−20
St. 34−36
St. 56

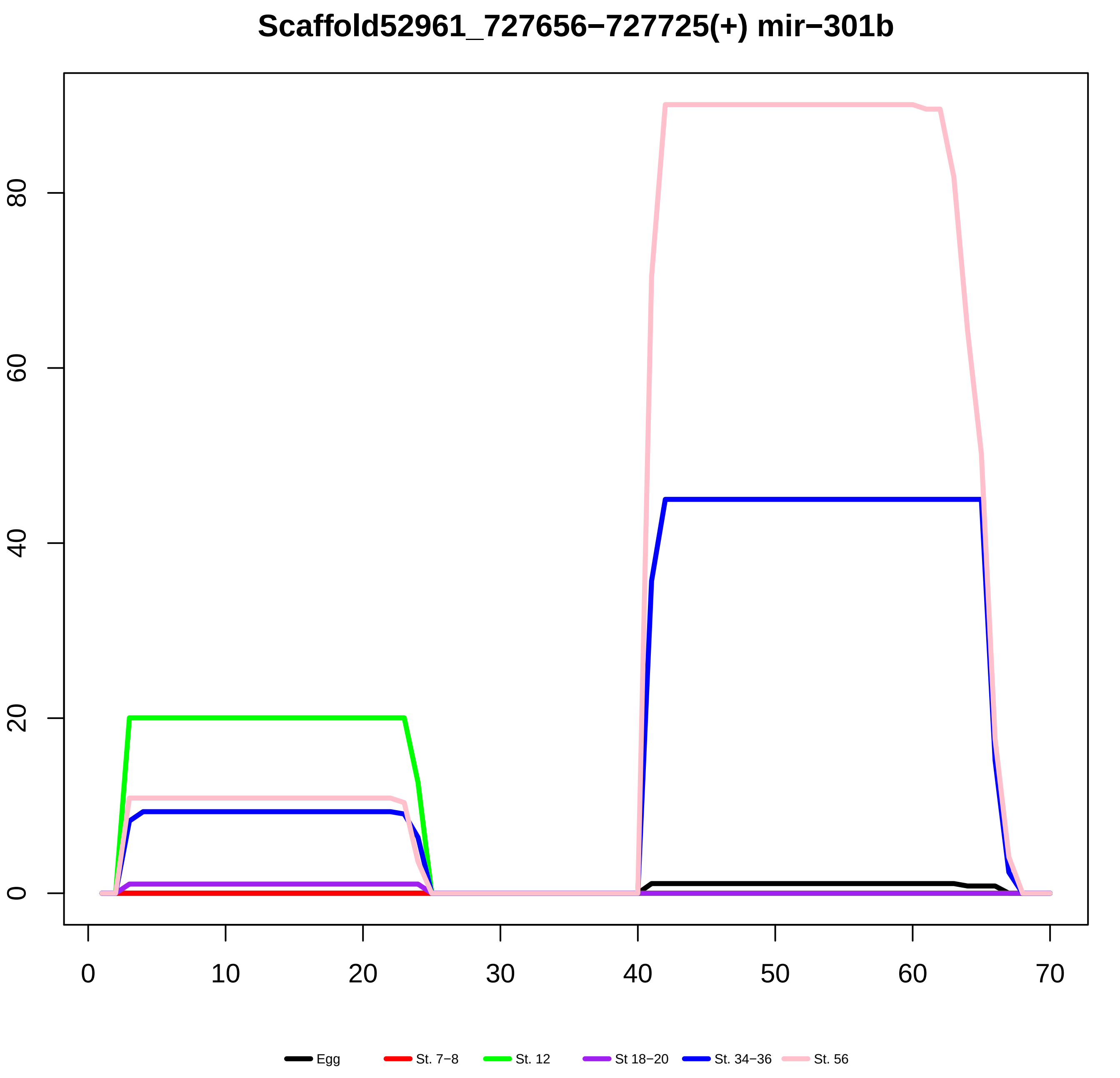

Scaffold52961_727656−727725(+) mir−301b
0
20
40
60
80
0
10
20
30
40
50
60
70
Egg
St. 7−8
St. 12
St 18−20
St. 34−36
St. 56

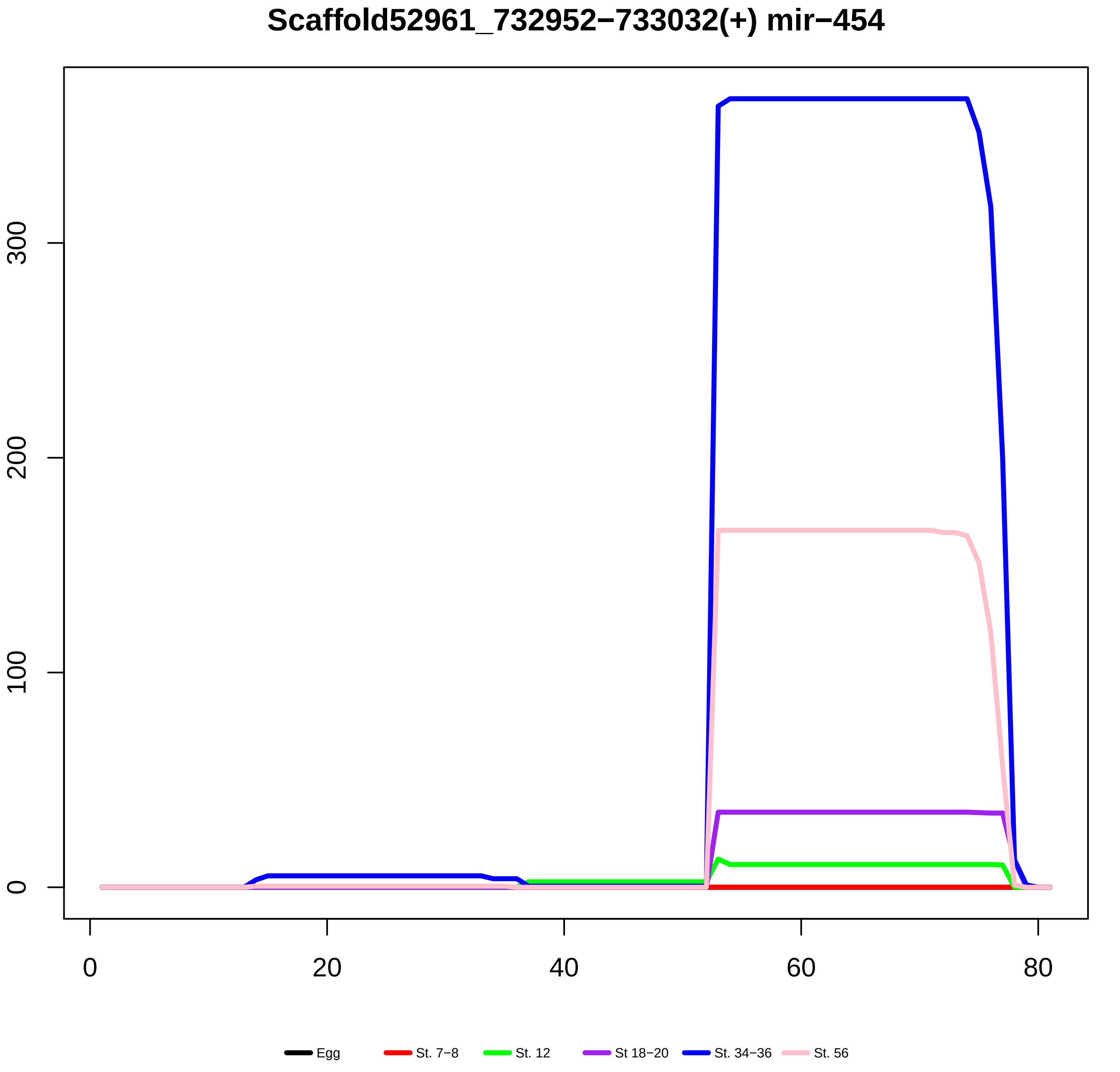

Scaffold52961_732952−733032(+) mir−454
0
100
200
300
0
20
40
60
80
Egg
St. 7−8
St. 12
St 18−20
St. 34−36
St. 56

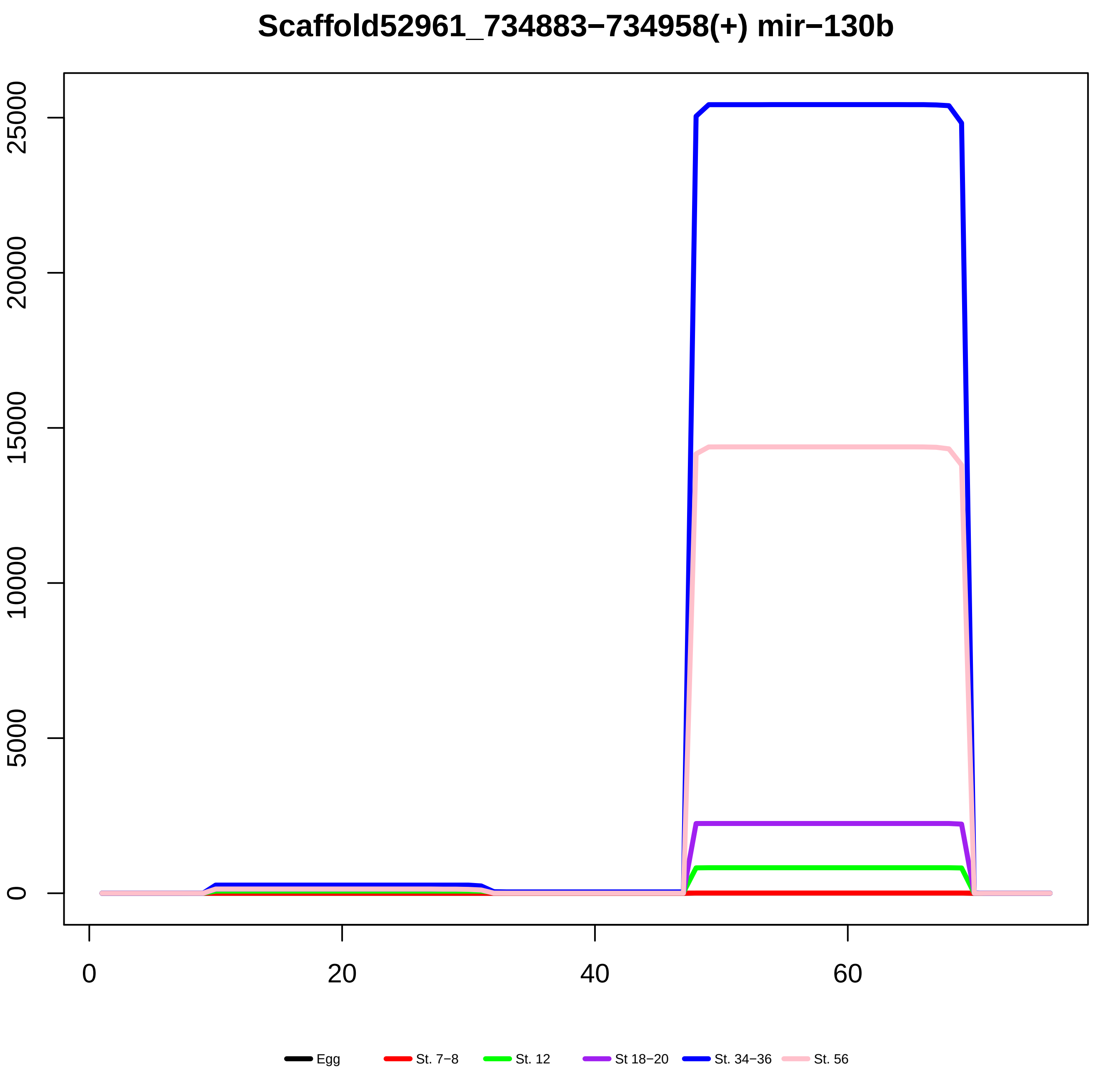

Scaffold52961_734883−734958(+) mir−130b
0
5000
10000
15000
20000
25000
0
20
40
60
Egg
St. 7−8
St. 12
St 18−20
St. 34−36
St. 56

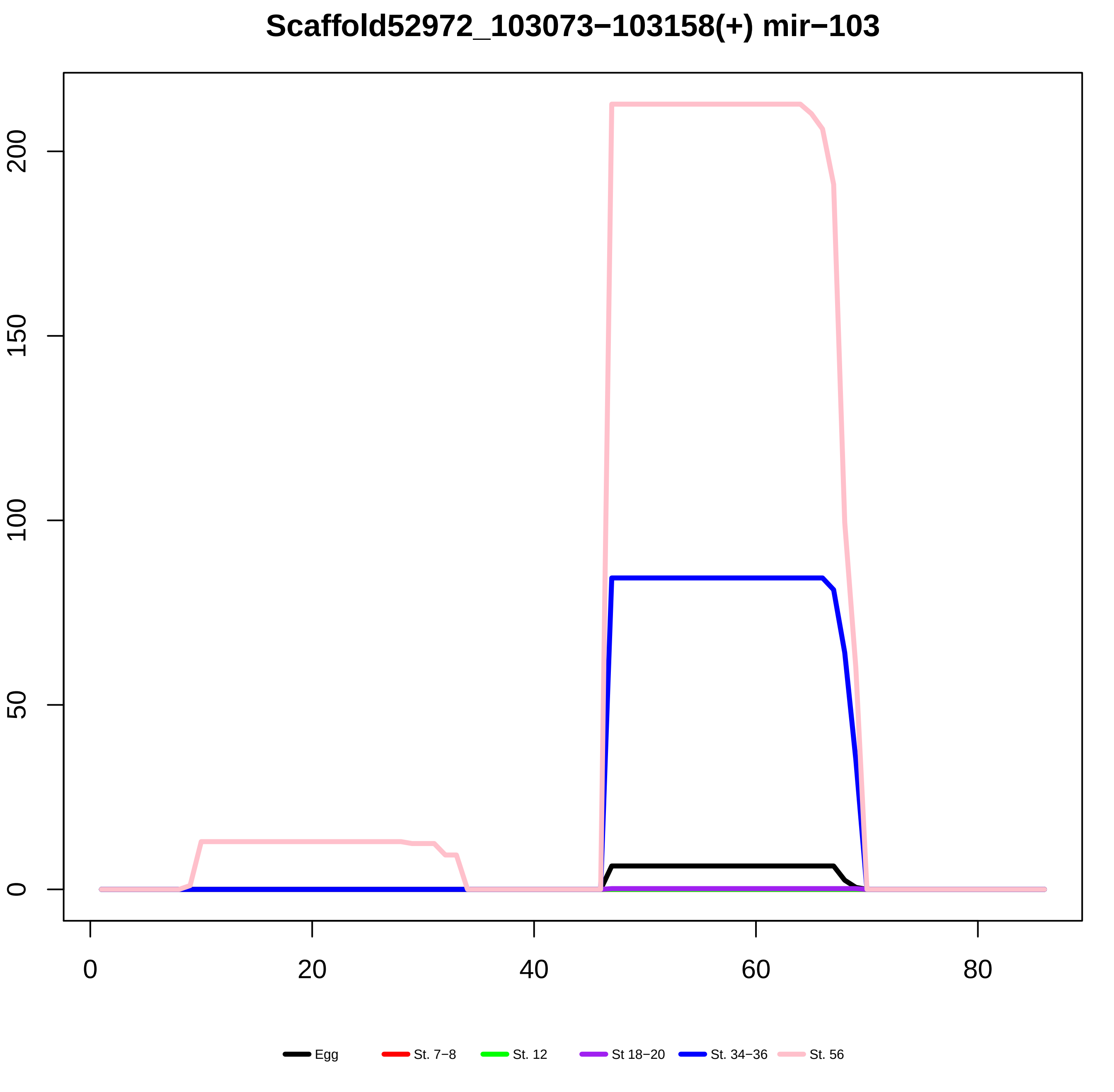

Scaffold52972_103073−103158(+) mir−103
0
50
100
150
200
0
20
40
60
80
Egg
St. 7−8
St. 12
St 18−20
St. 34−36
St. 56

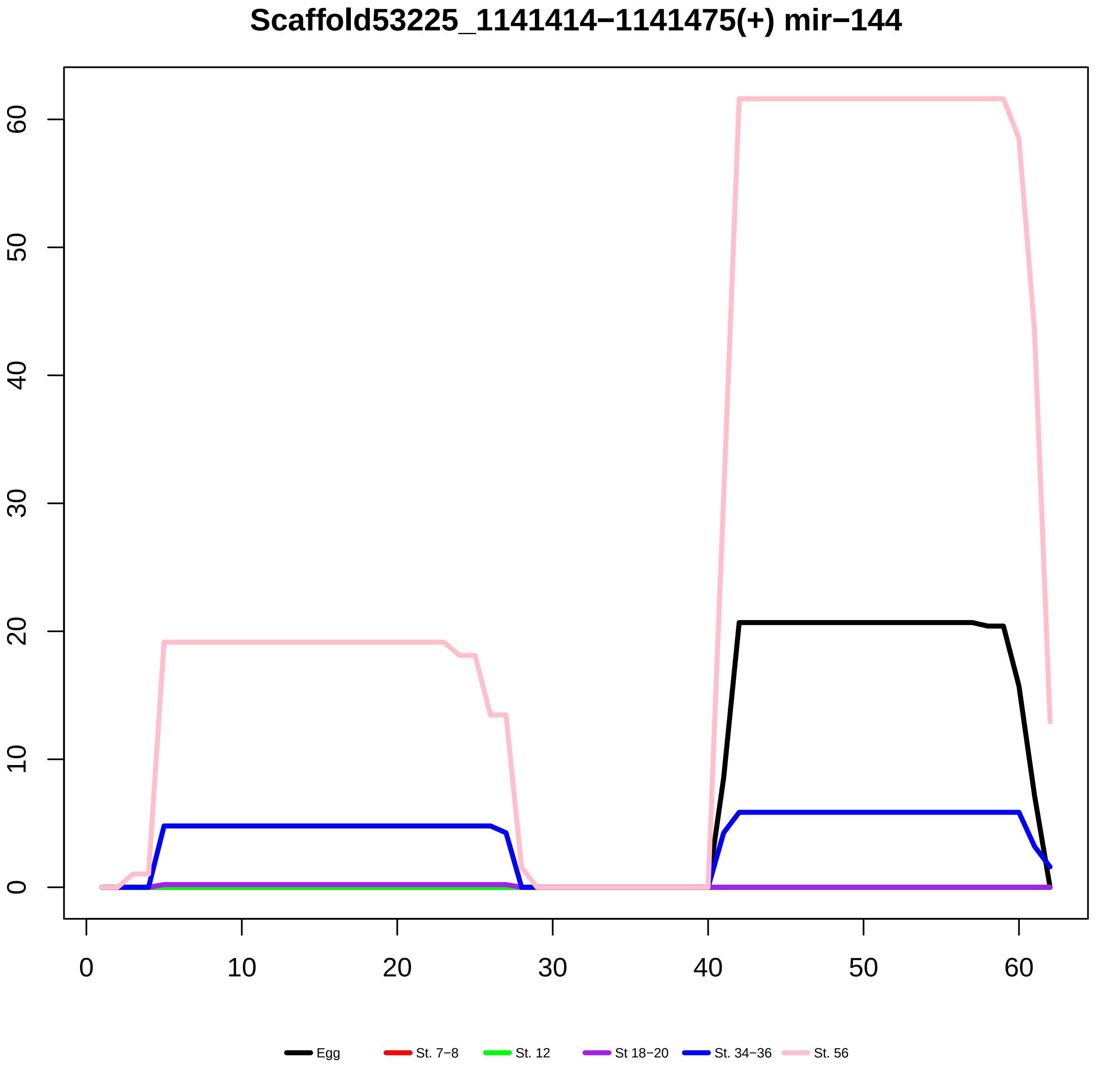

Scaffold53225_1141414−1141475(+) mir−144
0
10
20
30
40
50
60
0
10
20
30
40
50
60
Egg
St. 7−8
St. 12
St 18−20
St. 34−36
St. 56

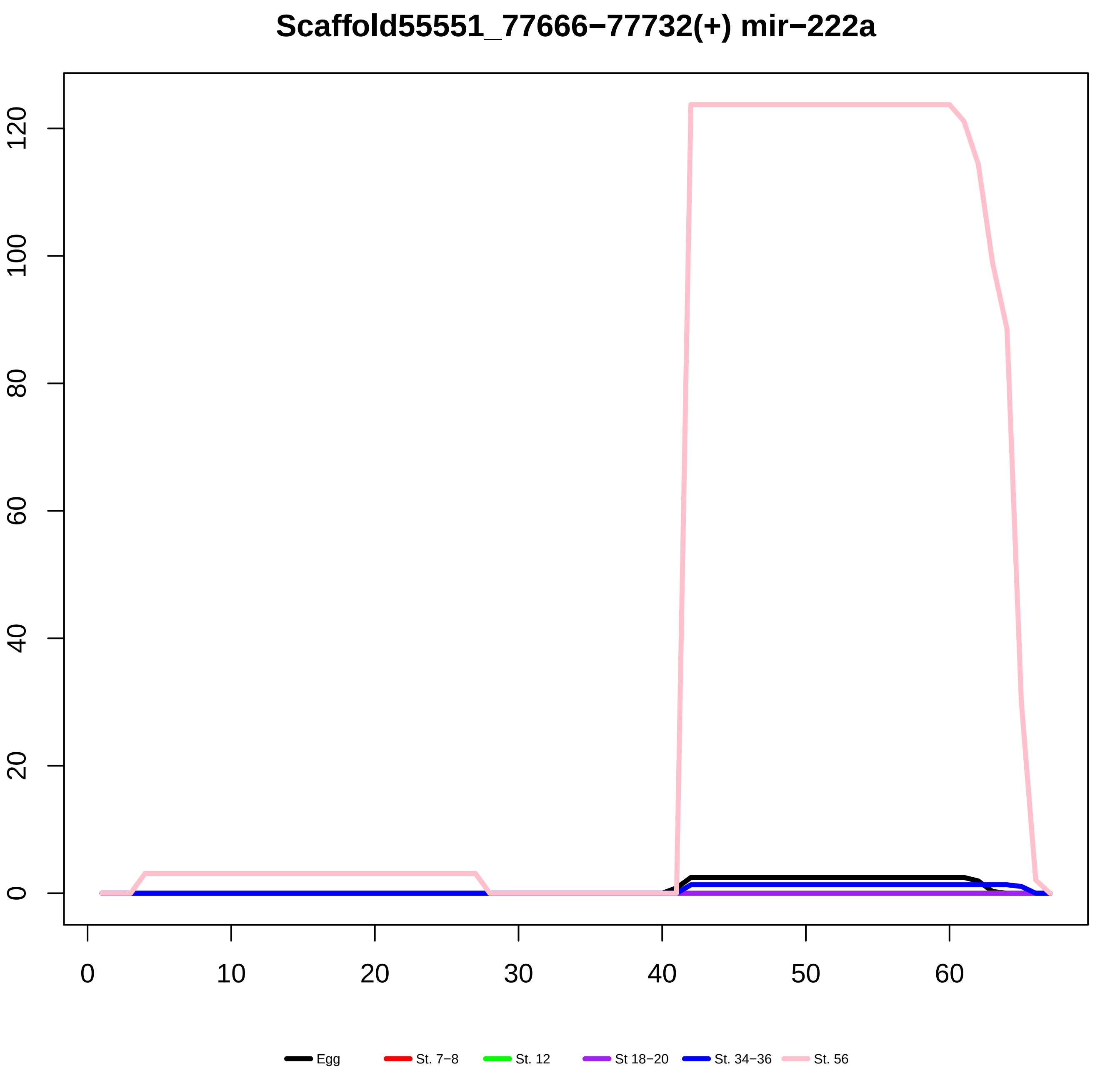
Scaffold55551_77666−77732(+) mir−222a
0
20
40
60
80
100
120
0
10
20
30
40
50
60
Egg
St. 7−8
St. 12
St 18−20
St. 34−36
St. 56

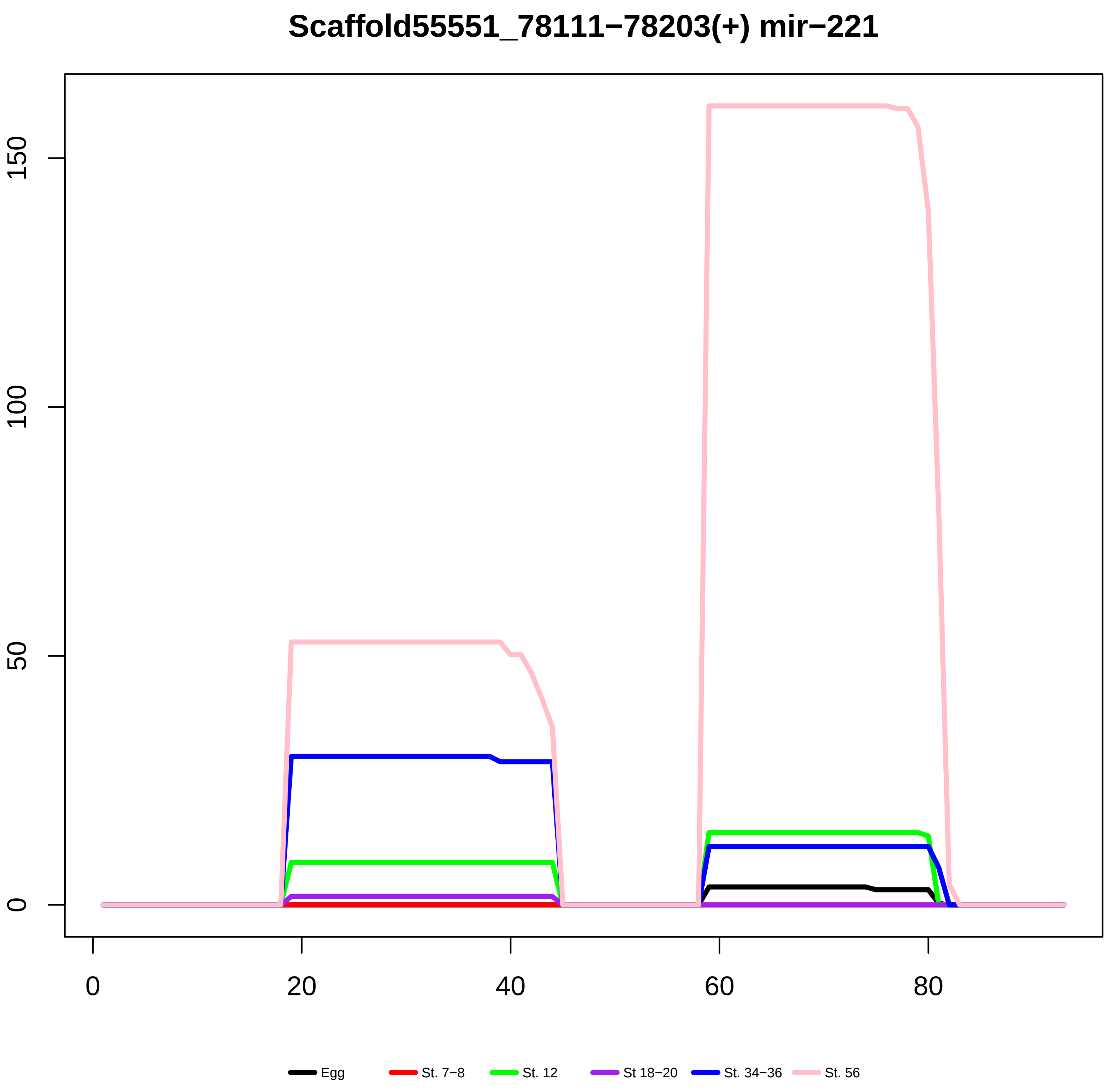

Scaffold55551_78111−78203(+) mir−221
0
50
100
150
0
20
40
60
80
Egg
St. 7−8
St. 12
St 18−20
St. 34−36
St. 56

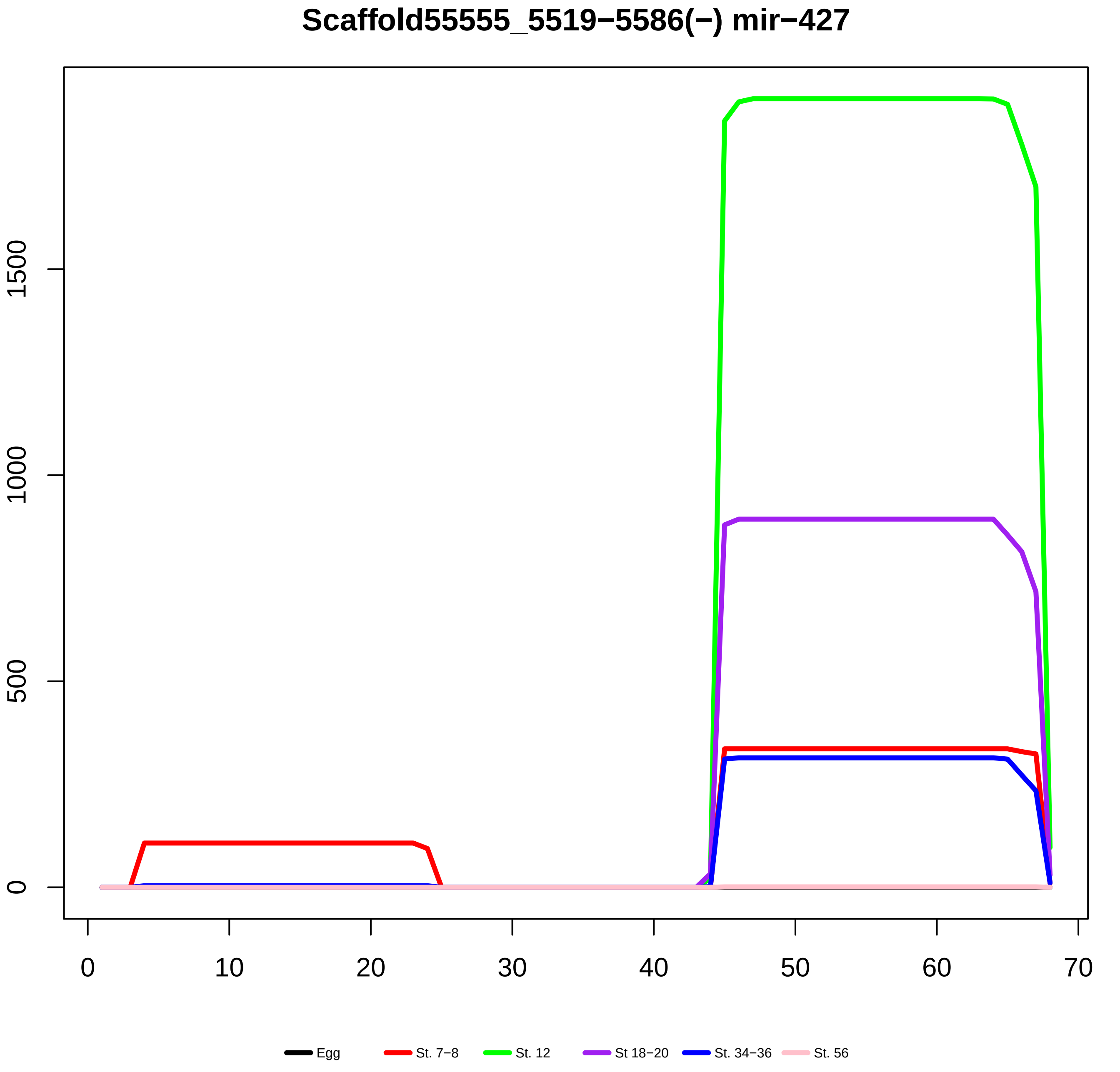

Scaffold55555_5519−5586(−) mir−427
0
500
1000
1500
0
10
20
30
40
50
60
70
Egg
St. 7−8
St. 12
St 18−20
St. 34−36
St. 56

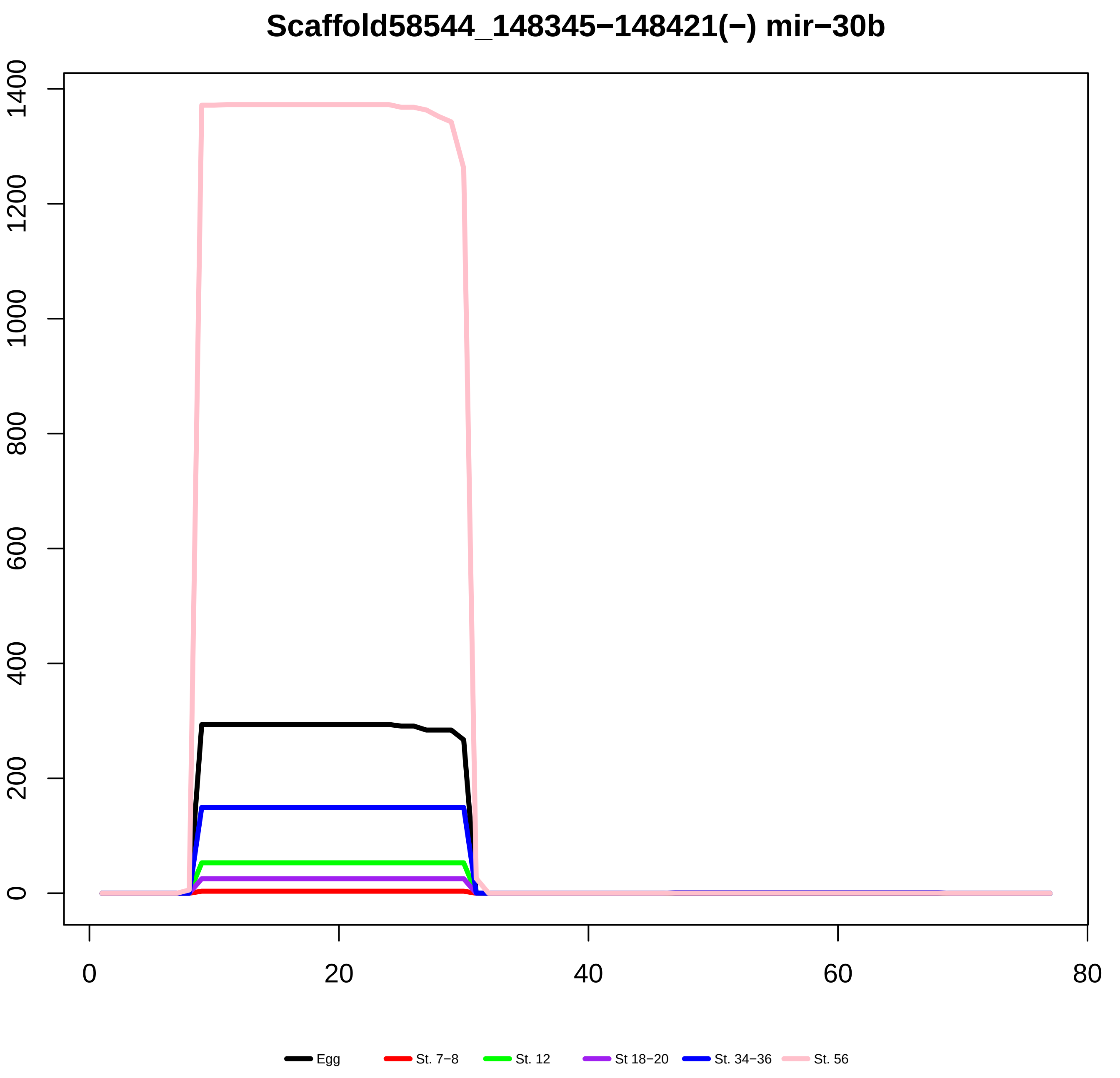
Scaffold58544_148345−148421(−) mir−30b
0
200
400
600
800
1000
1200
1400
0
20
40
60
80
Egg
St. 7−8
St. 12
St 18−20
St. 34−36
St. 56

Scaffold58544_151301−151381(−) mir−30d

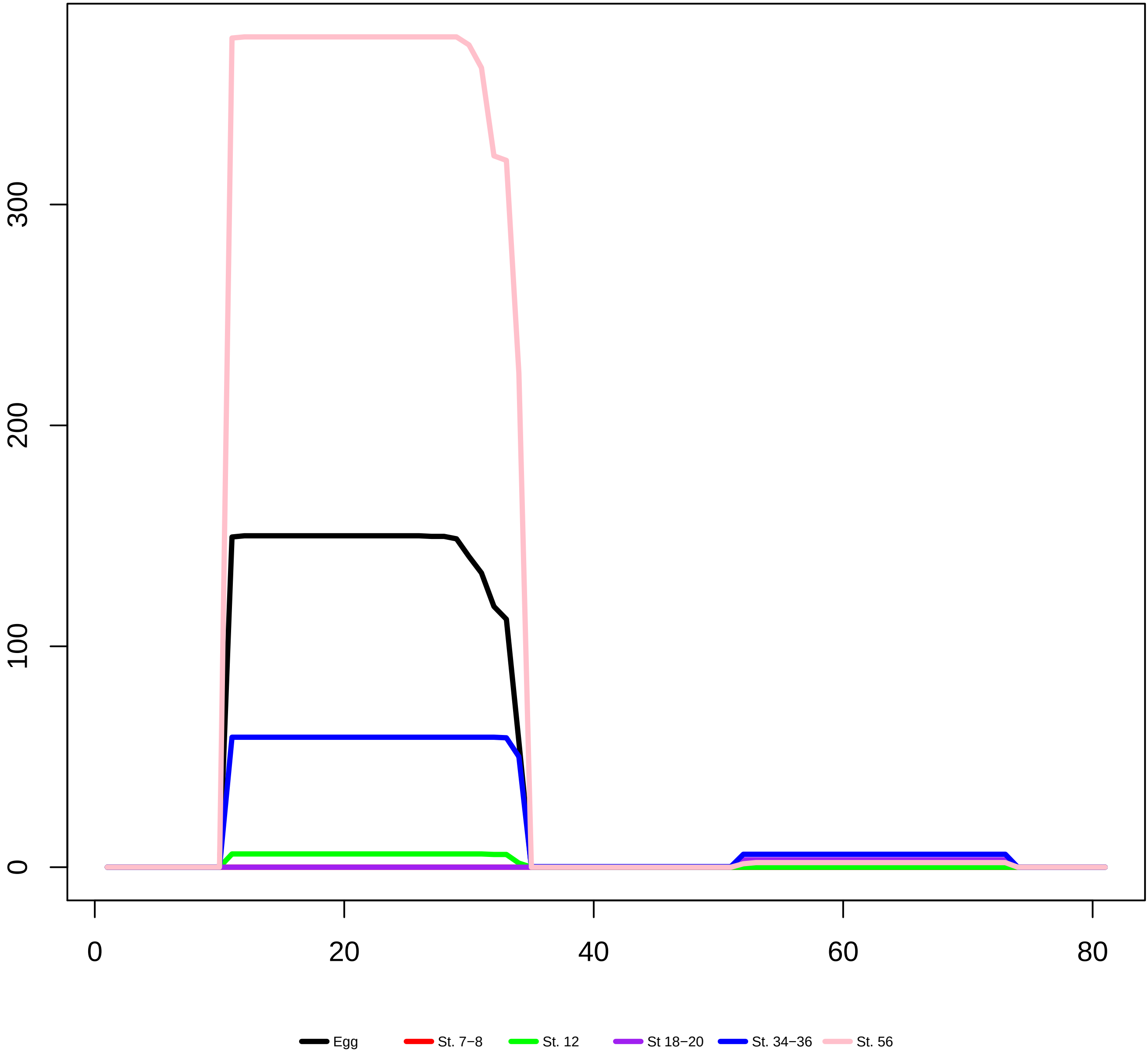

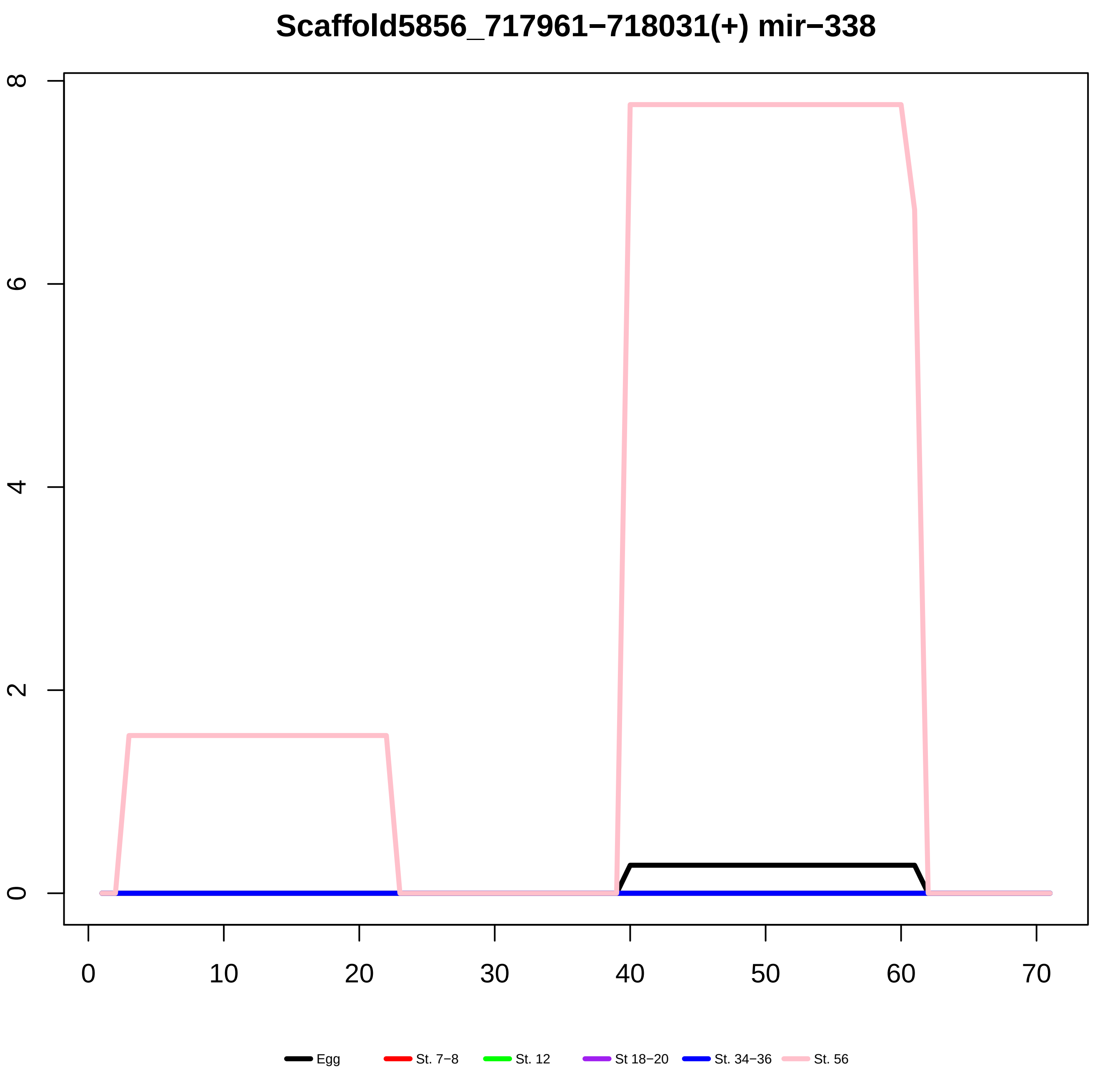
Scaffold5856_717961−718031(+) mir−338
0
2
4
6
8
0
10
20
30
40
50
60
70
Egg
St. 7−8
St. 12
St 18−20
St. 34−36
St. 56

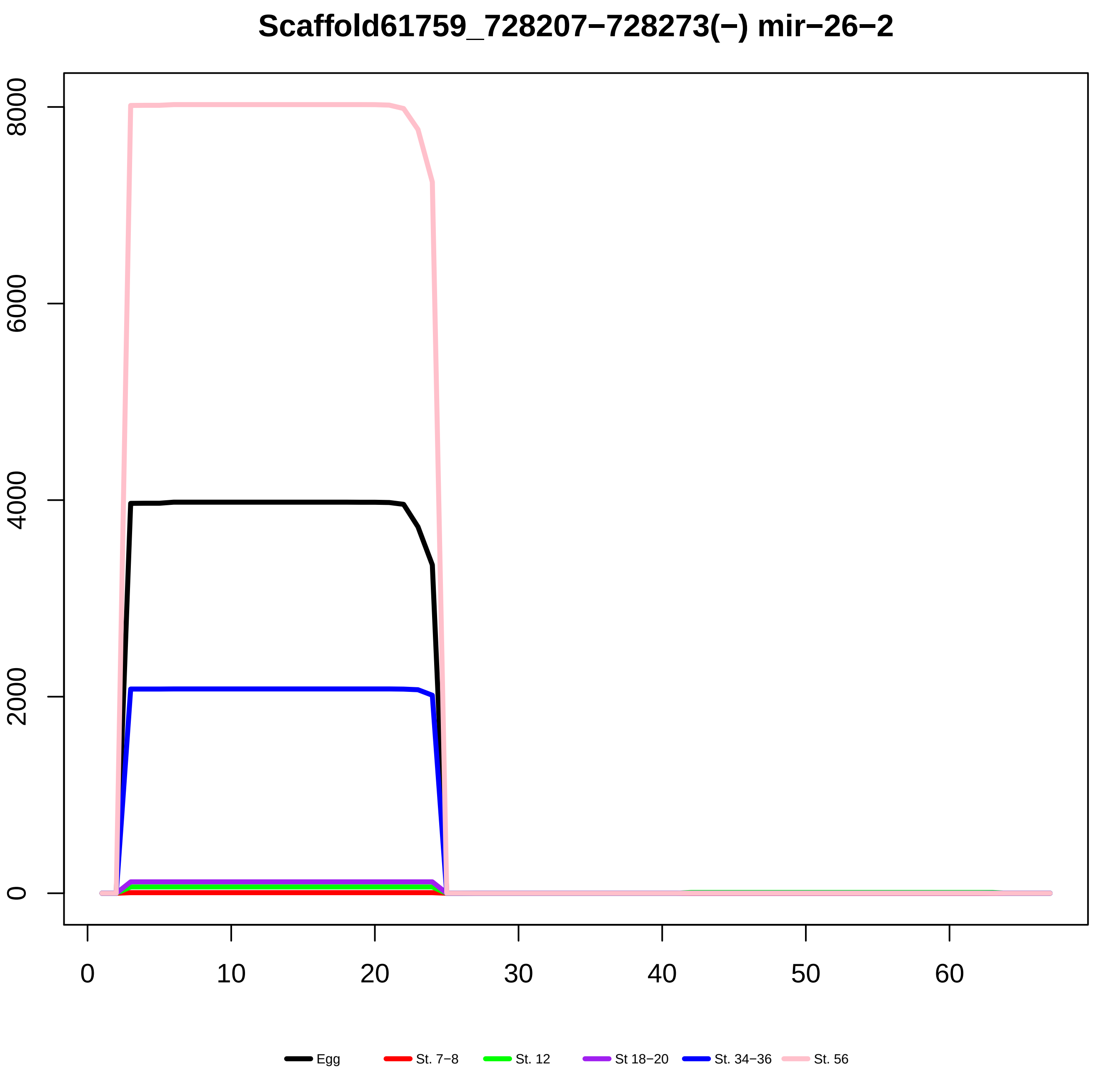

Scaffold61759_728207−728273(−) mir−26−2
0
2000
4000
6000
8000
0
10
20
30
40
50
60
Egg
St. 7−8
St. 12
St 18−20
St. 34−36
St. 56

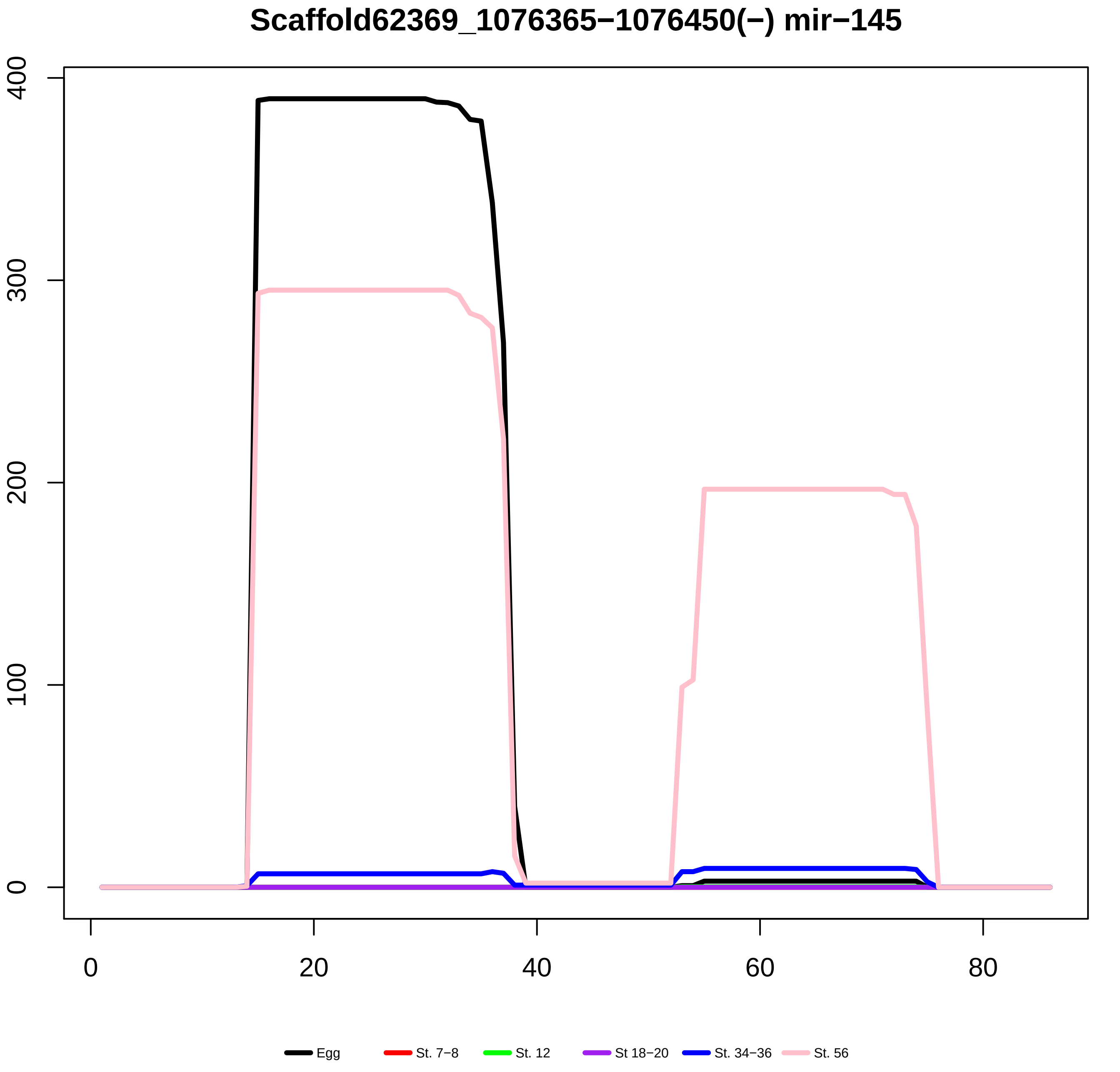

Scaffold62369_1076365−1076450(−) mir−145
0
100
200
300
400
0
20
40
60
80
Egg
St. 7−8
St. 12
St 18−20
St. 34−36
St. 56

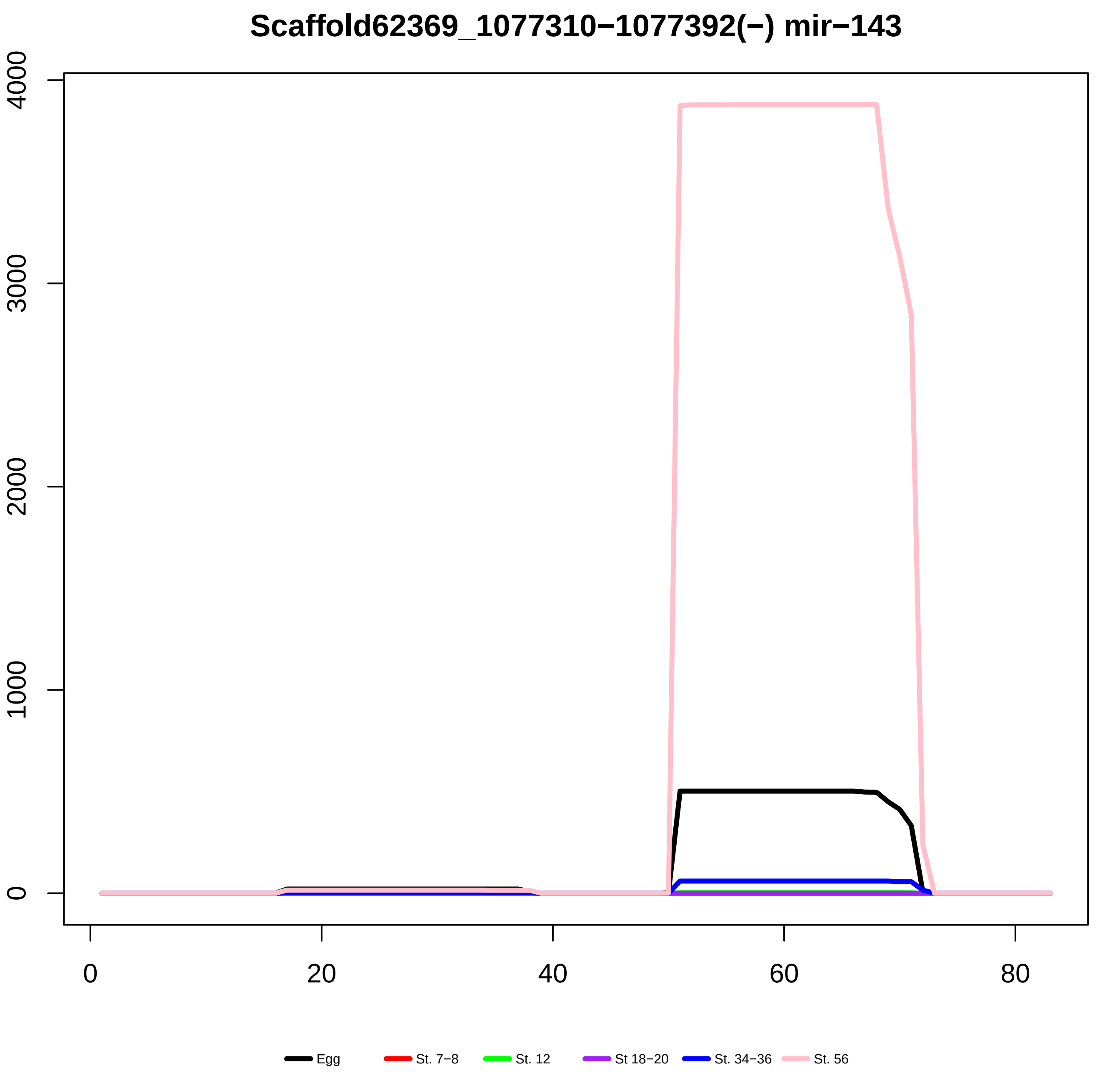
Scaffold62369_1077310−1077392(−) mir−143
0
1000
2000
3000
4000
0
20
40
60
80
Egg
St. 7−8
St. 12
St 18−20
St. 34−36
St. 56

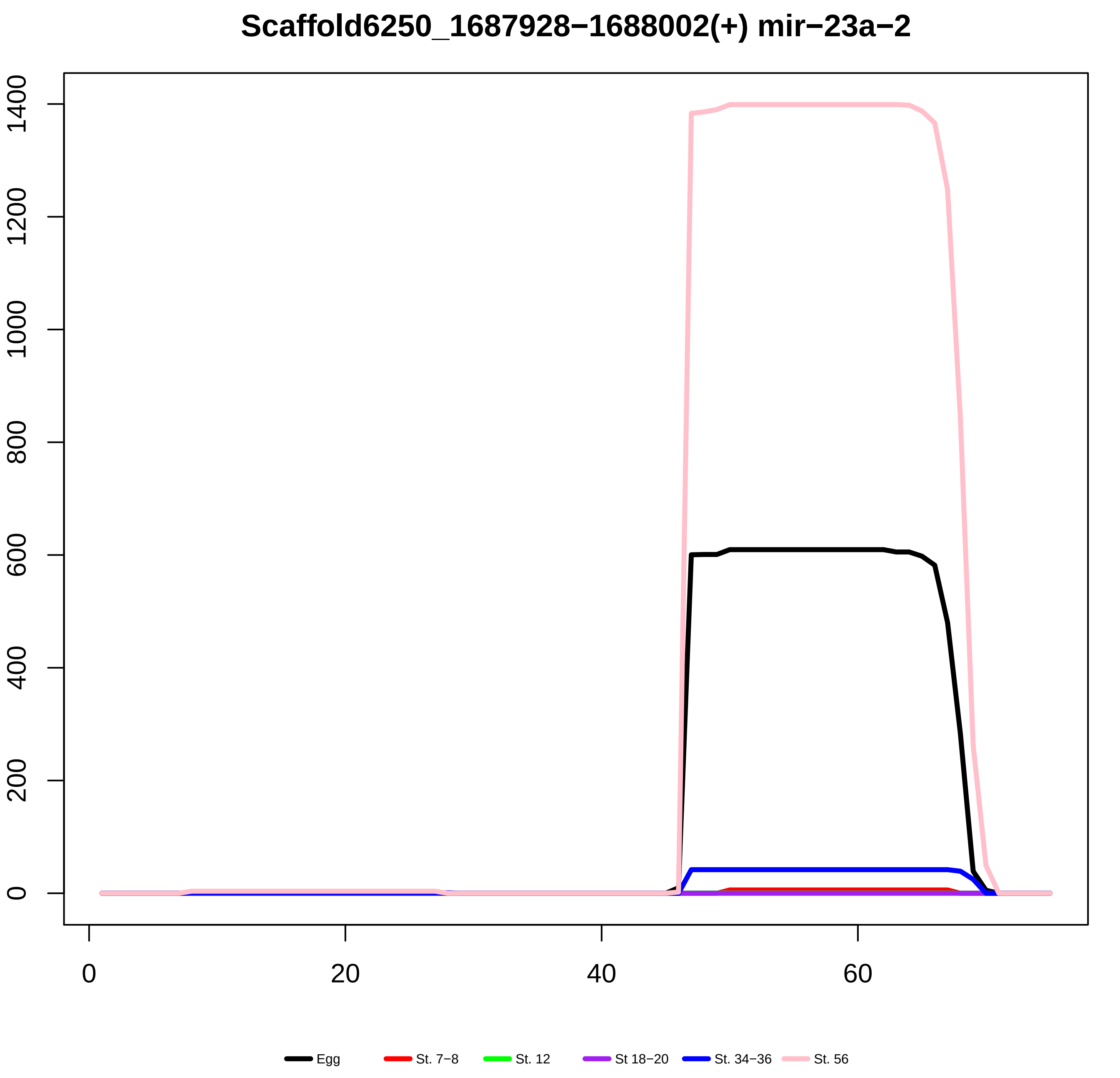

Scaffold6250_1687928−1688002(+) mir−23a−2
0
200
400
600
800
1000
1200
1400
0
20
40
60
Egg
St. 7−8
St. 12
St 18−20
St. 34−36
St. 56

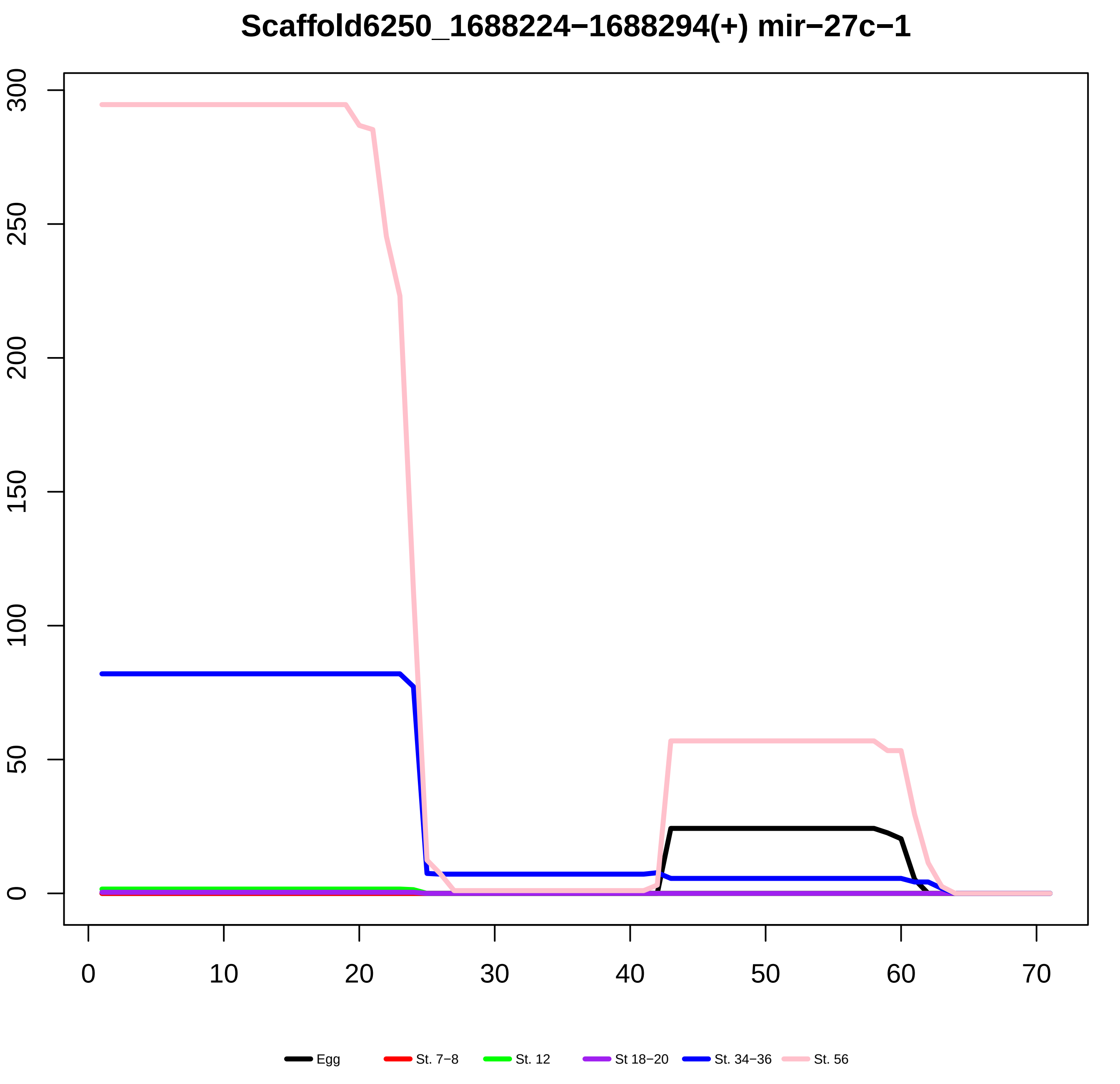

Scaffold6250_1688224−1688294(+) mir−27c−1
0
50
100
150
200
250
300
0
10
20
30
40
50
60
70
Egg
St. 7−8
St. 12
St 18−20
St. 34−36
St. 56

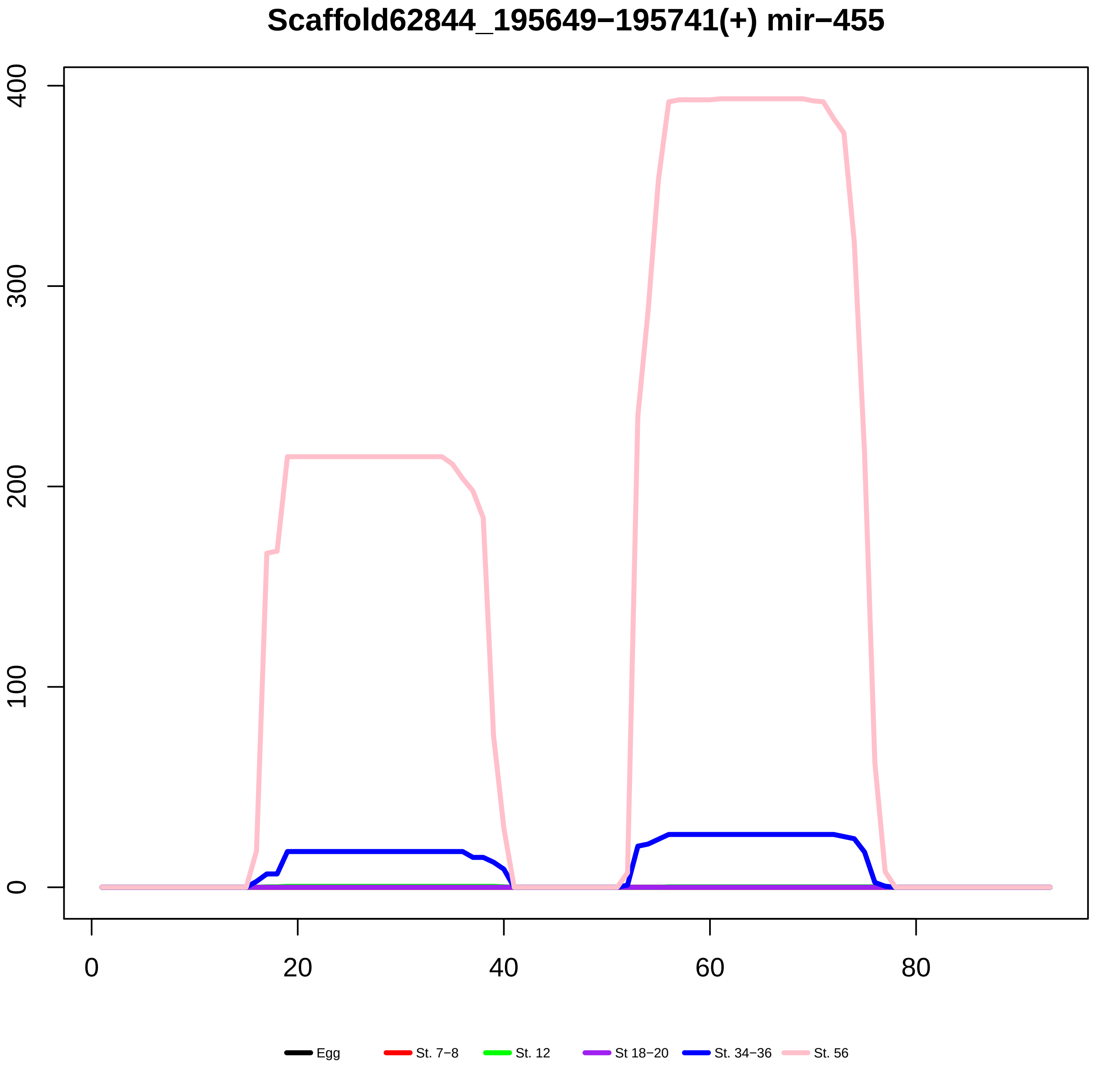

Scaffold62844_195649−195741(+) mir−455
0
100
200
300
400
0
20
40
60
80
Egg
St. 7−8
St. 12
St 18−20
St. 34−36
St. 56

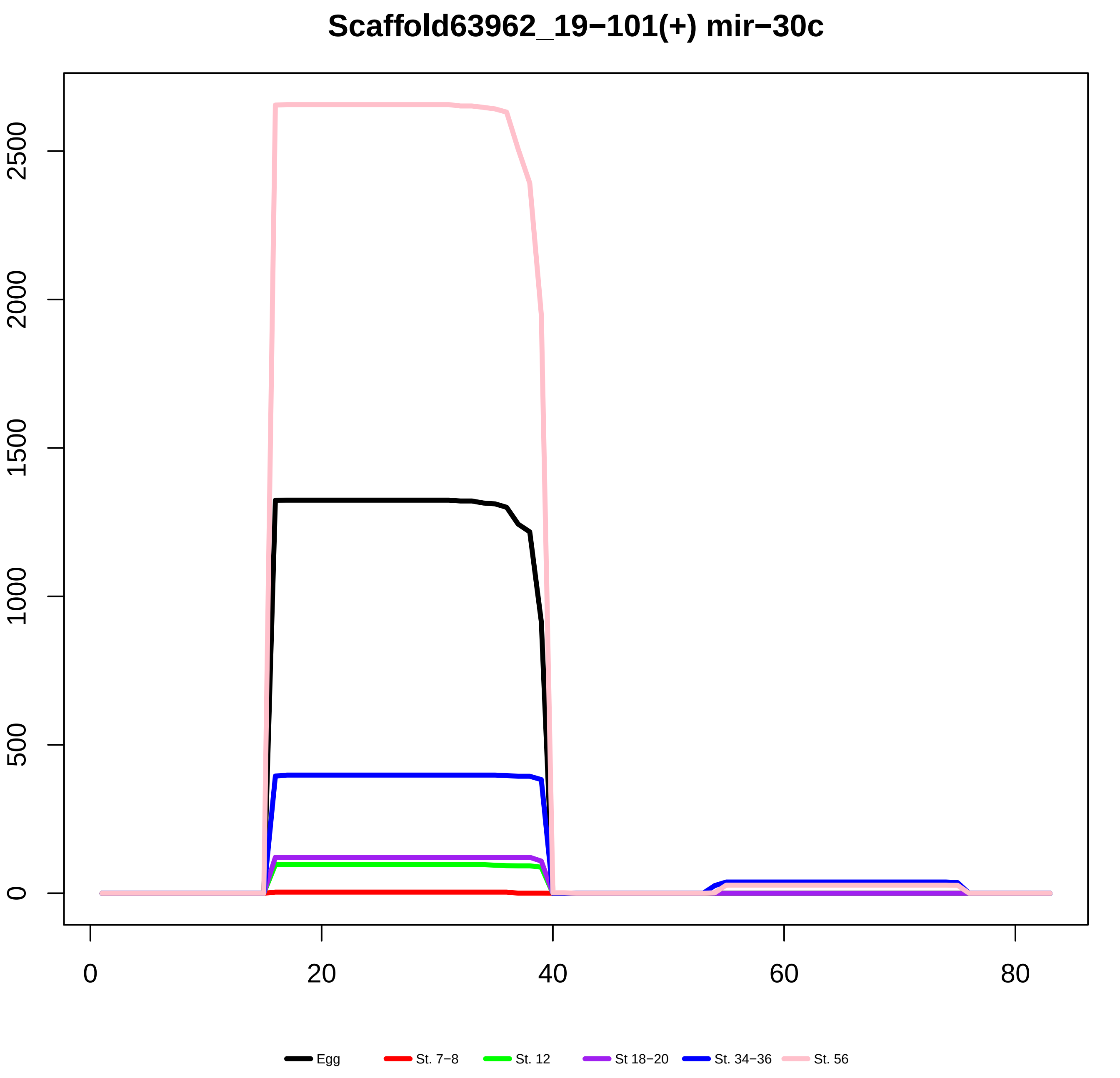
Scaffold63962_19−101(+) mir−30c
0
500
1000
1500
2000
2500
0
20
40
60
80
Egg
St. 7−8
St. 12
St 18−20
St. 34−36
St. 56

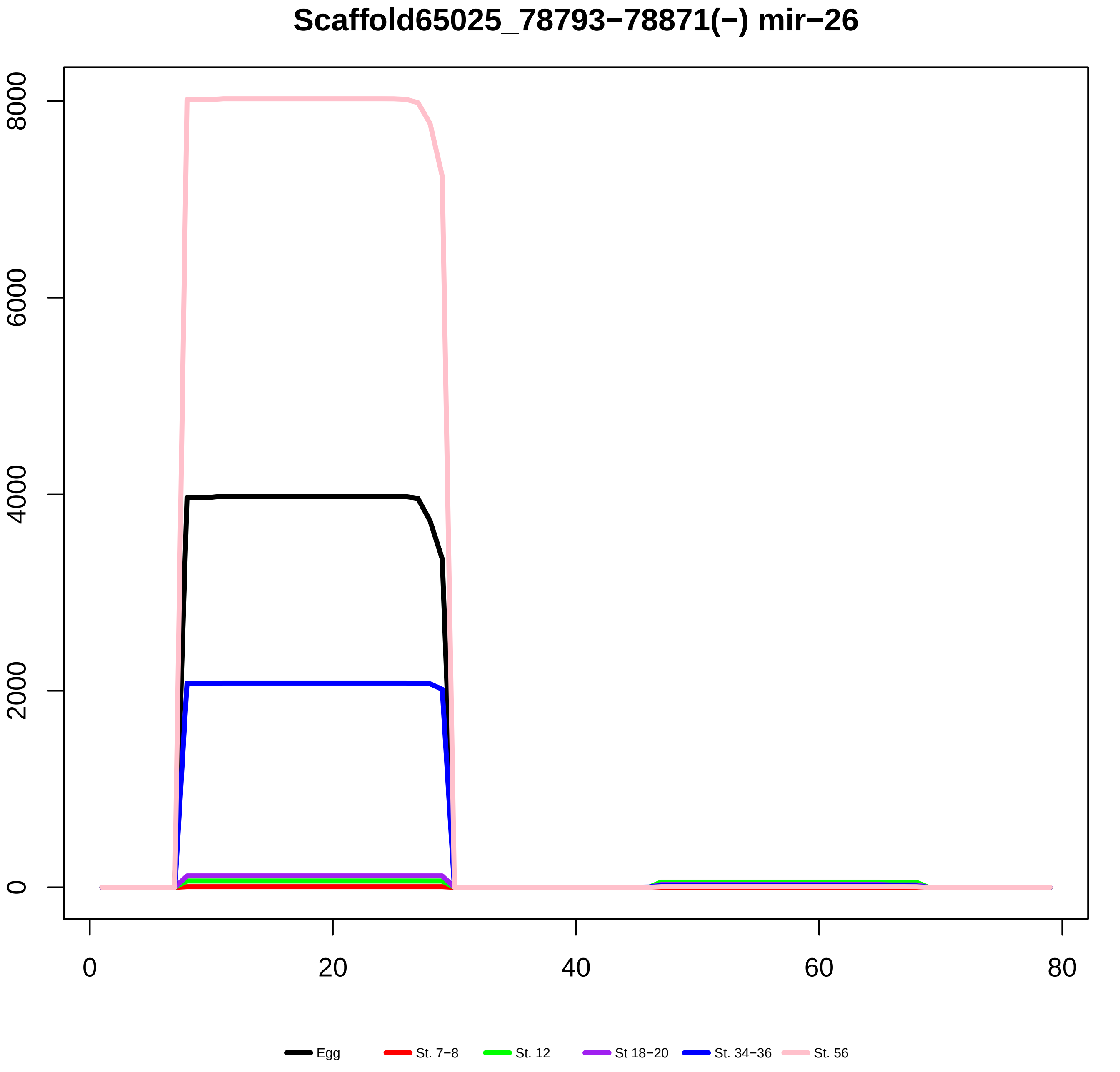
Scaffold65025_78793−78871(−) mir−26
8000
6000
4000
2000
0
0
20
40
60
80
Egg
St. 7−8
St. 12
St 18−20
St. 34−36
St. 56

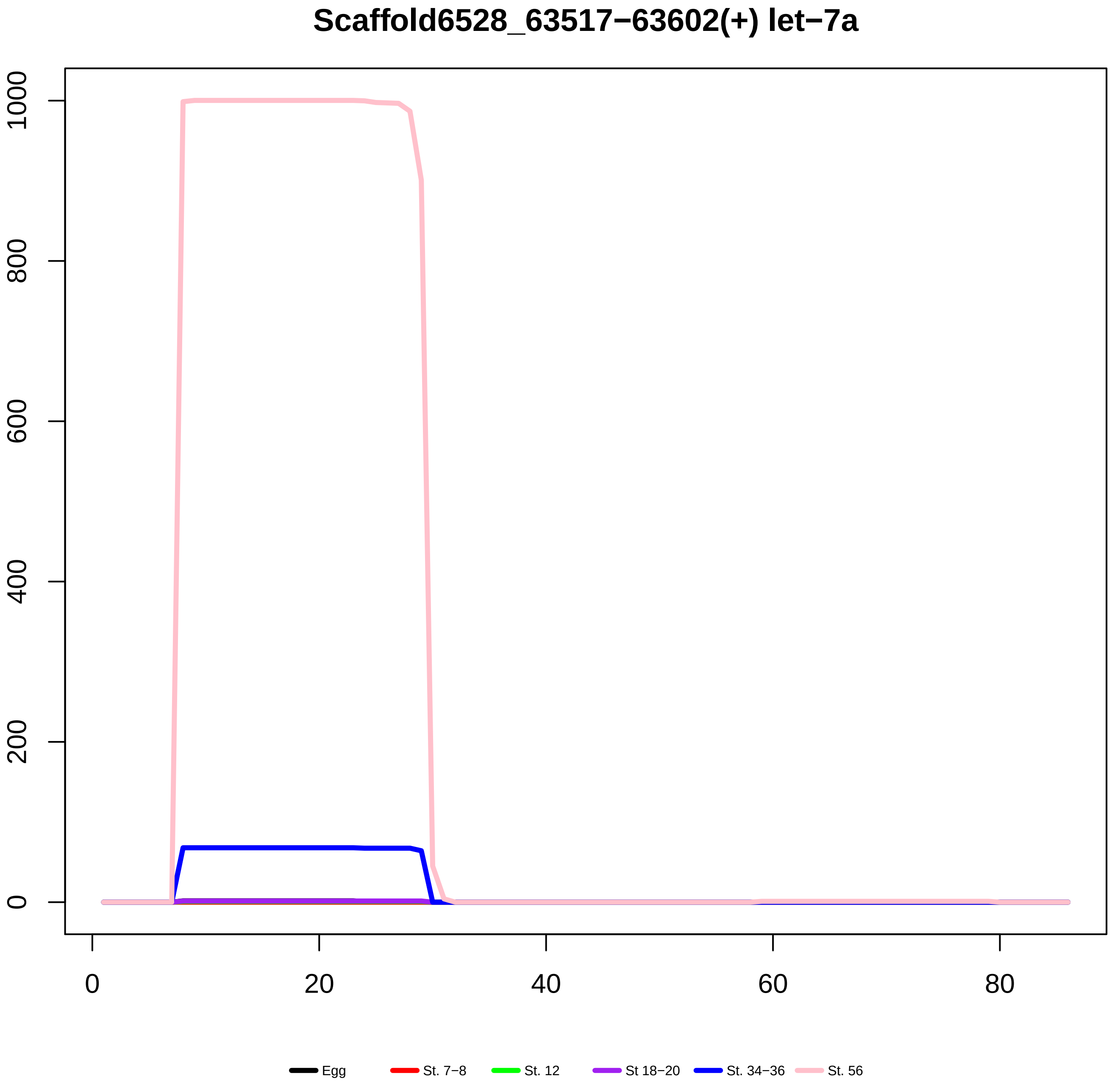
Scaffold6528_63517−63602(+) let−7a
0
200
400
600
800
1000
0
20
40
60
80
Egg
St. 7−8
St. 12
St 18−20
St. 34−36
St. 56

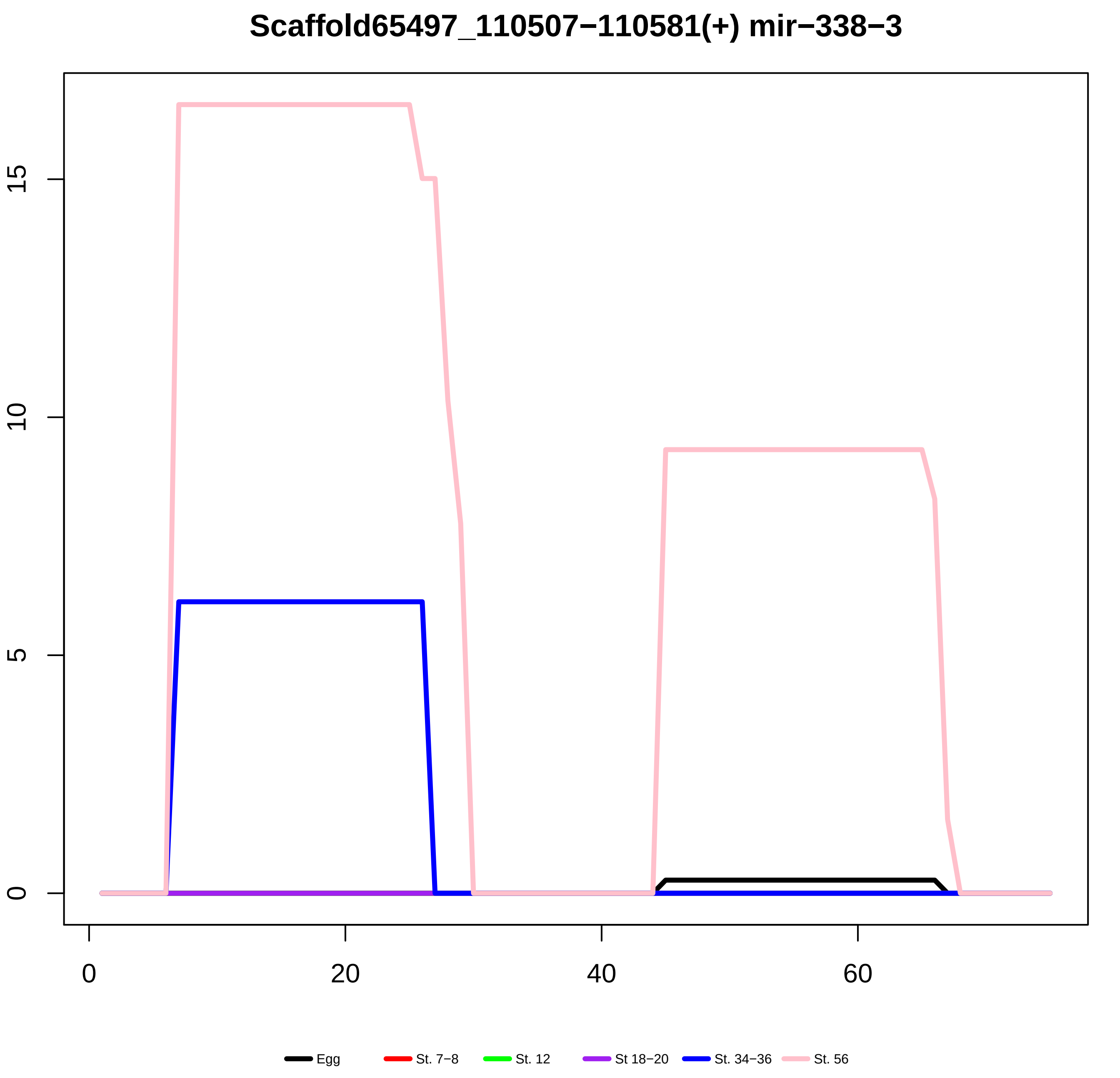
Scaffold65497_110507−110581(+) mir−338−3
0
5
10
15
0
20
40
60
Egg
St. 7−8
St. 12
St 18−20
St. 34−36
St. 56

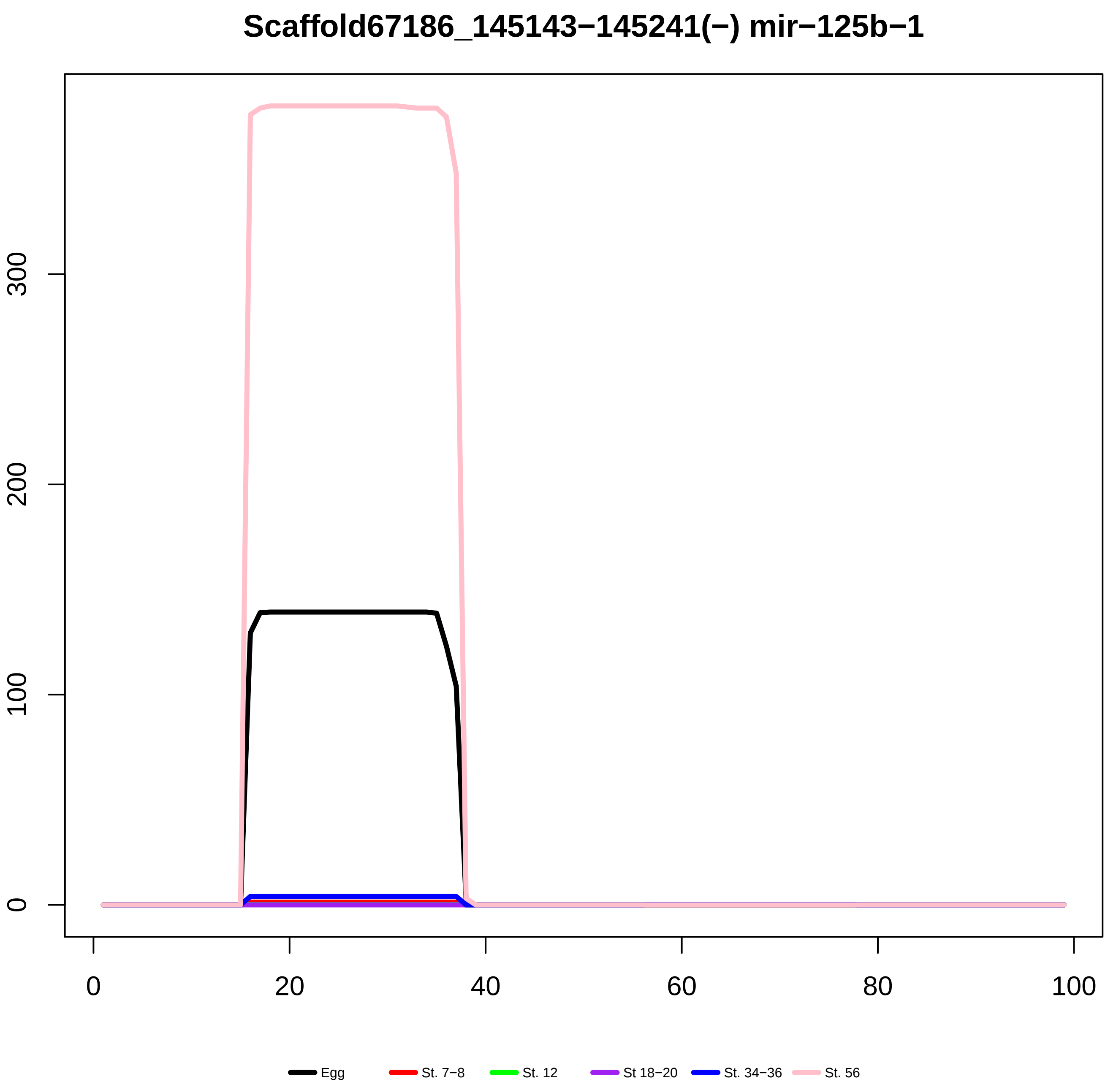

Scaffold67186_145143−145241(−) mir−125b−1
0
100
200
300
0
20
40
60
80
100
Egg
St. 7−8
St. 12
St 18−20
St. 34−36
St. 56

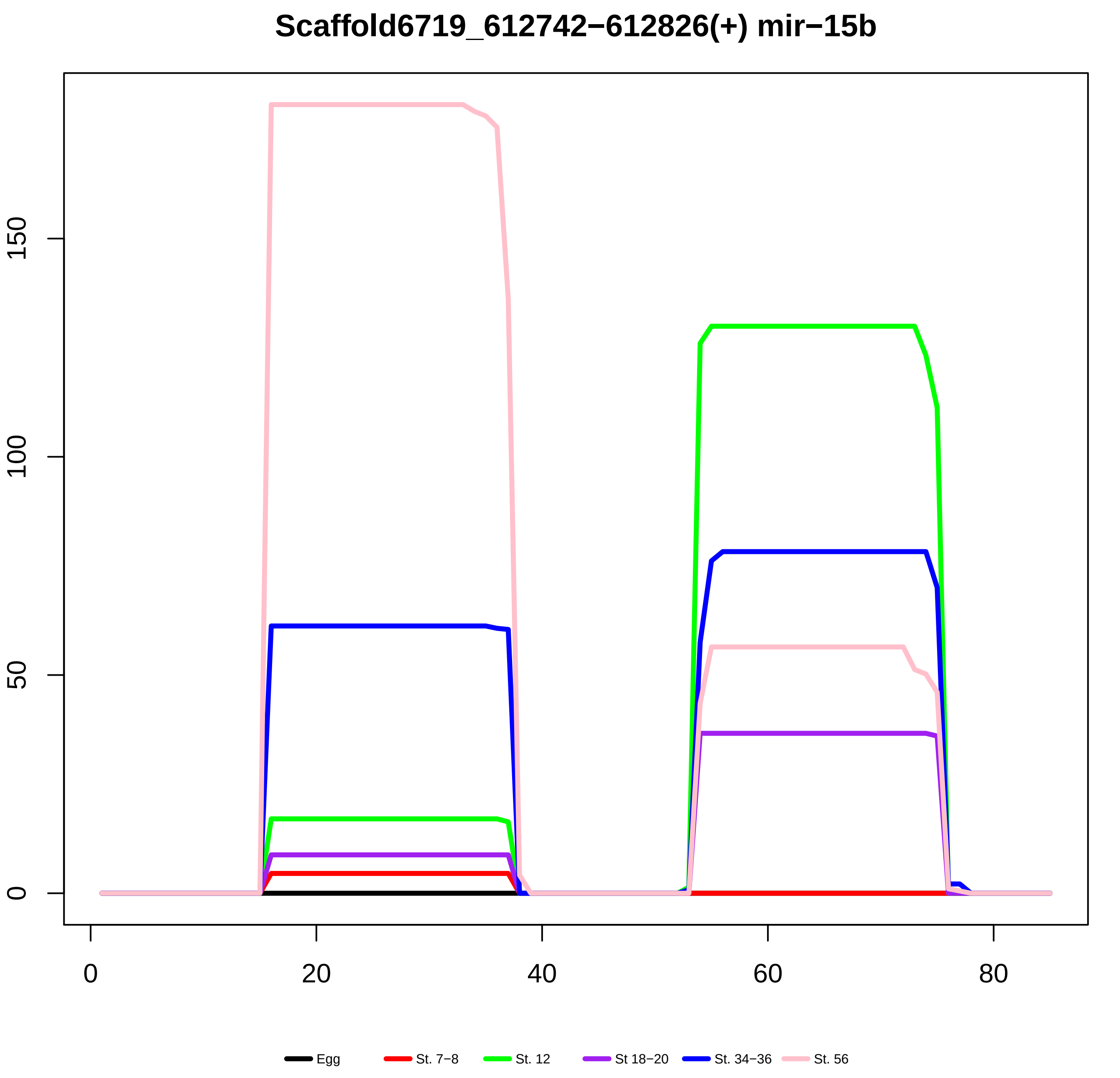
Scaffold6719_612742−612826(+) mir−15b
0
50
100
150
0
20
40
60
80
Egg
St. 7−8
St. 12
St 18−20
St. 34−36
St. 56

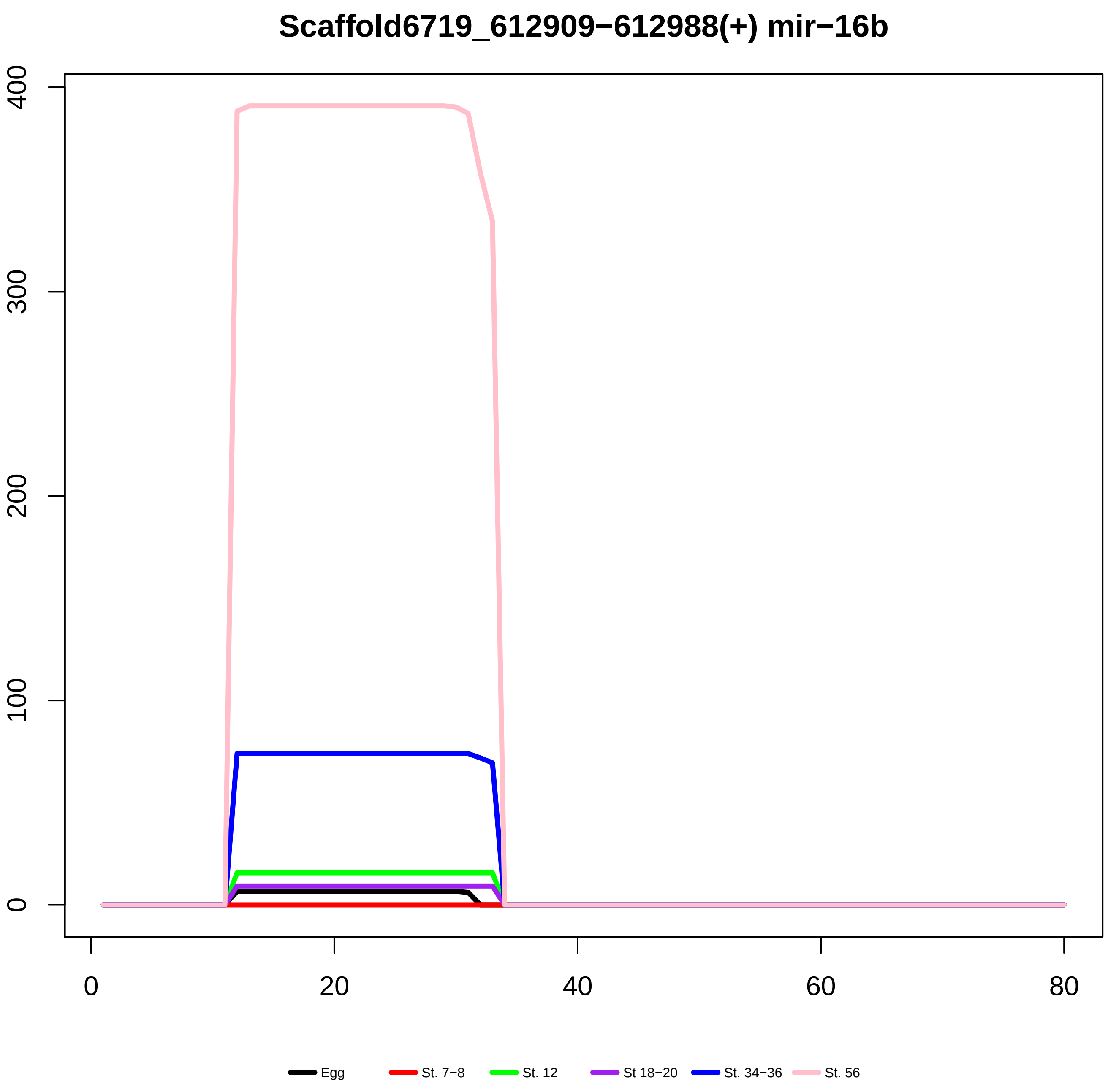
Scaffold6719_612909−612988(+) mir−16b
0
100
200
300
400
0
20
40
60
80
Egg
St. 7−8
St. 12
St 18−20
St. 34−36
St. 56

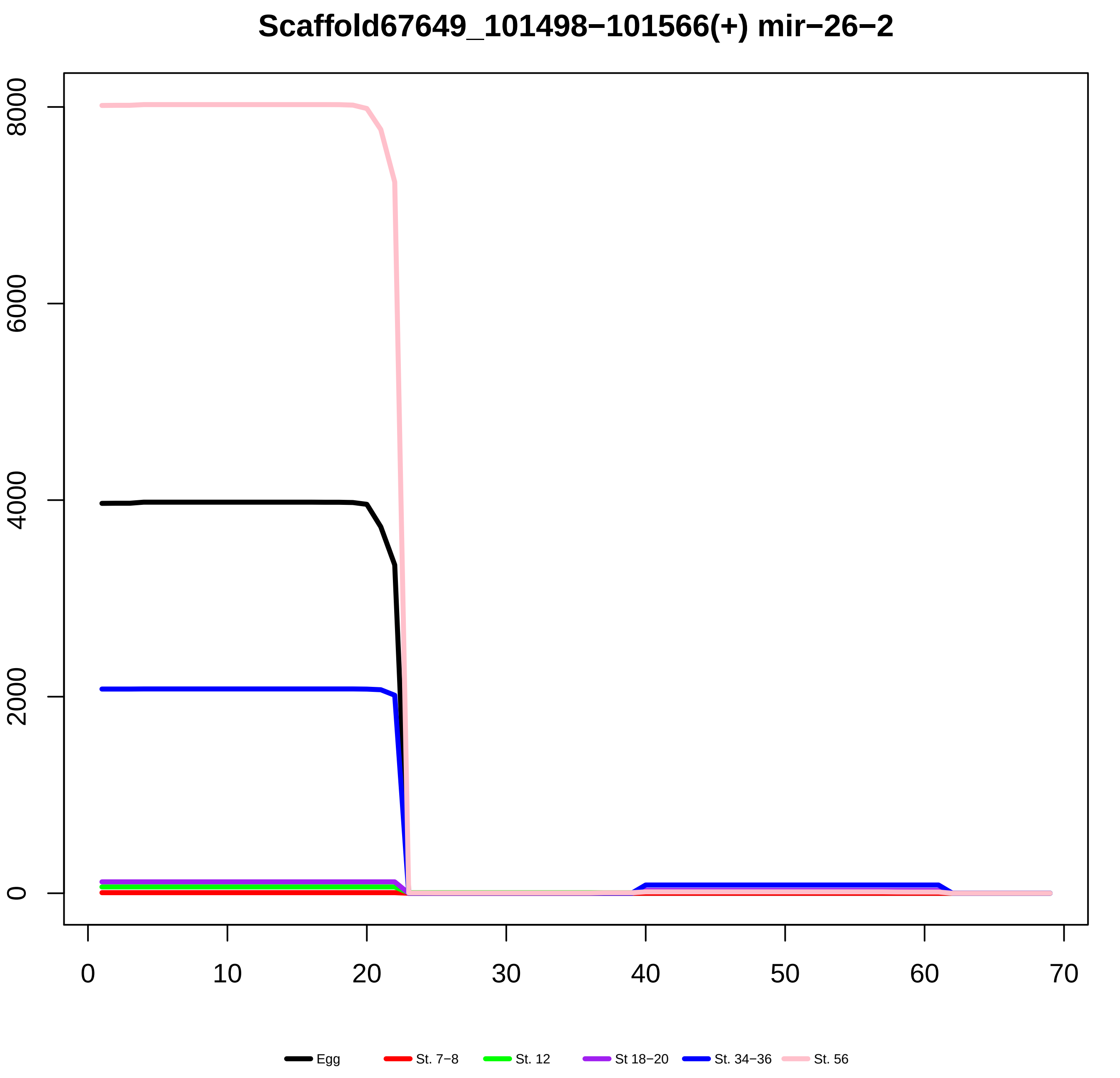

Scaffold67649_101498−101566(+) mir−26−2
0
2000
4000
6000
8000
0
10
20
30
40
50
60
70
Egg
St. 7−8
St. 12
St 18−20
St. 34−36
St. 56

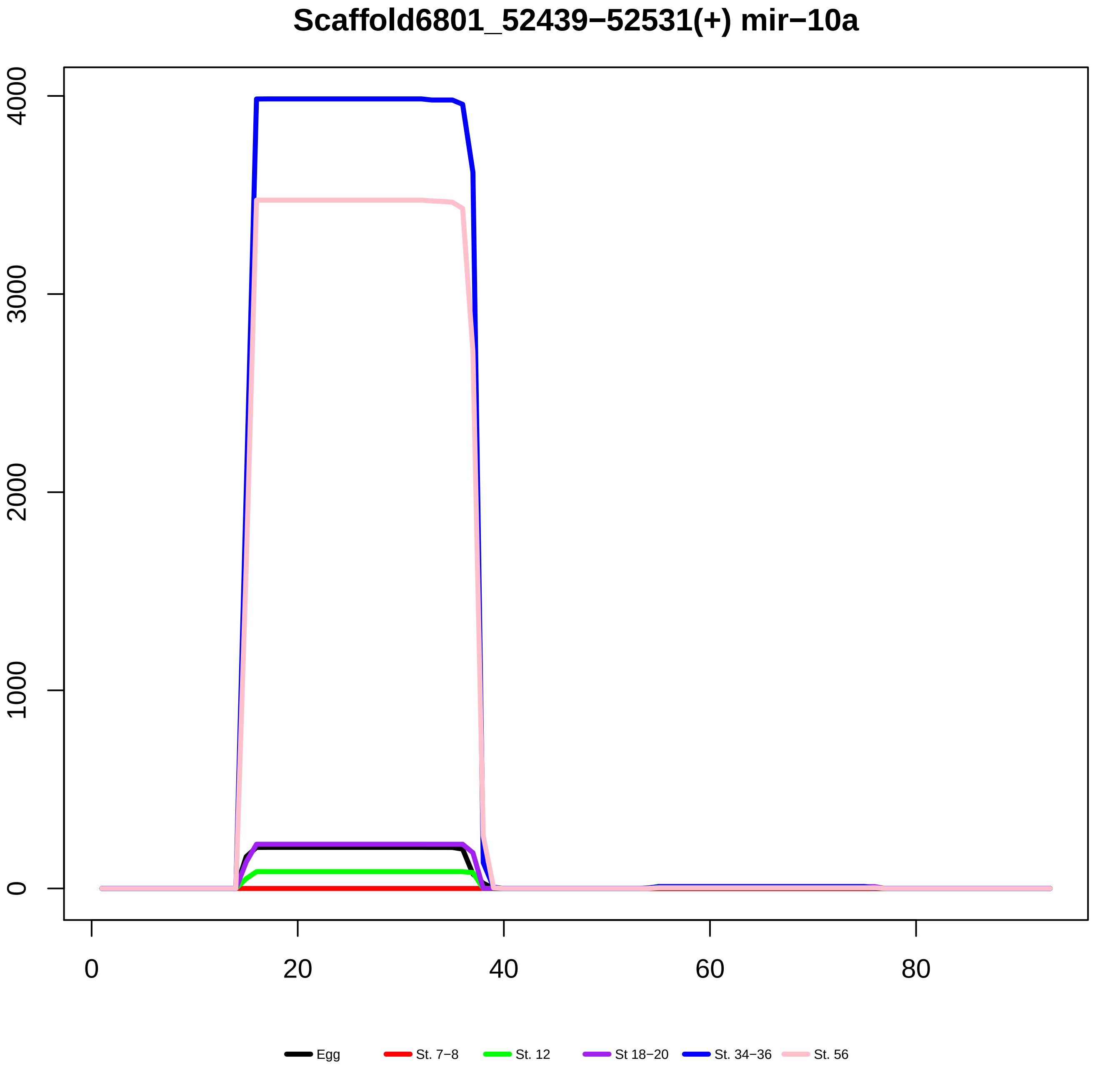

Scaffold6801_52439−52531(+) mir−10a
0
1000
2000
3000
4000
0
20
40
60
80
Egg
St. 7−8
St. 12
St 18−20
St. 34−36
St. 56

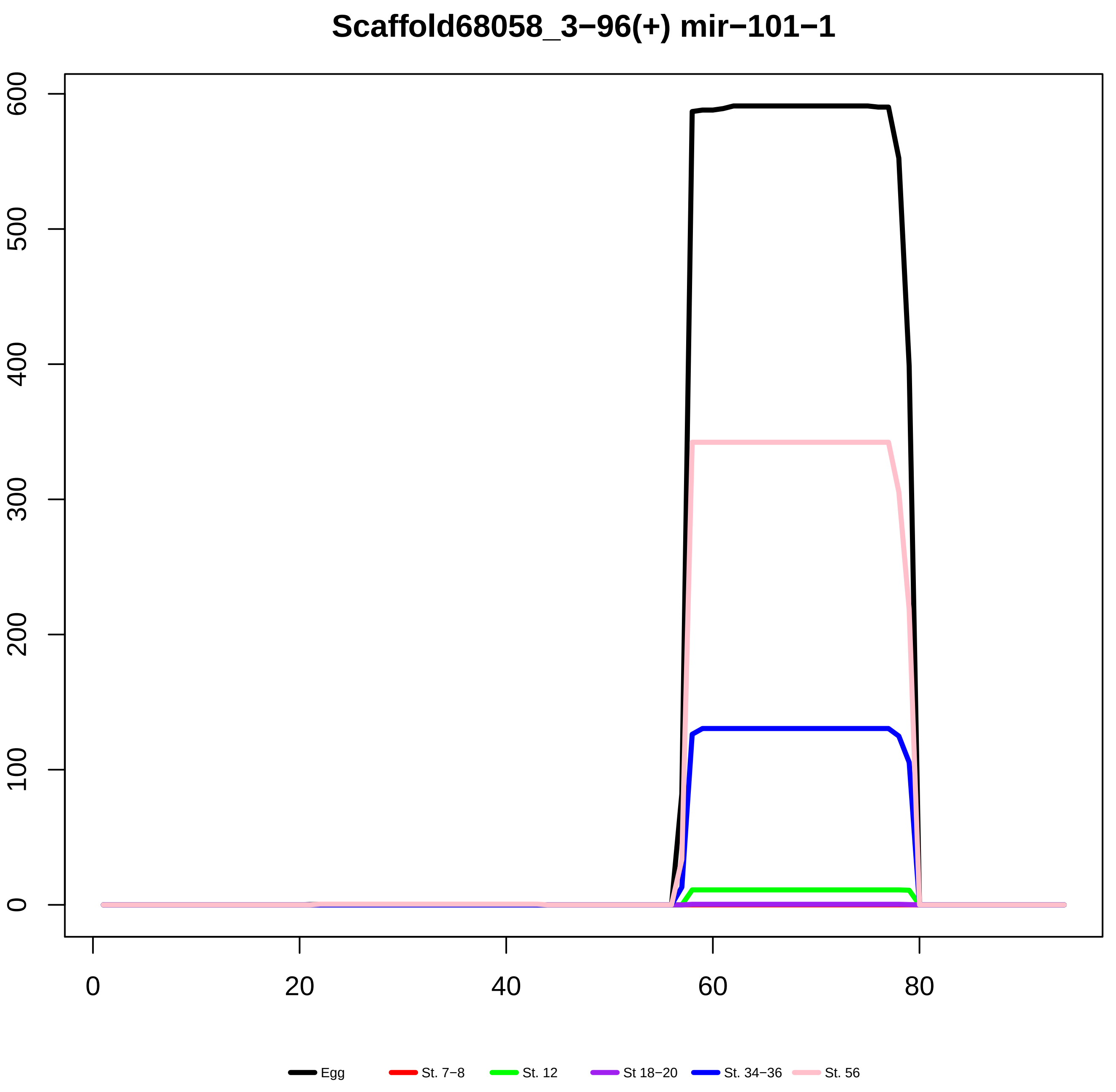
Scaffold68058_3−96(+) mir−101−1
600
500
400
300
200
100
0
0
20
40
60
80
Egg
St. 7−8
St. 12
St 18−20
St. 34−36
St. 56

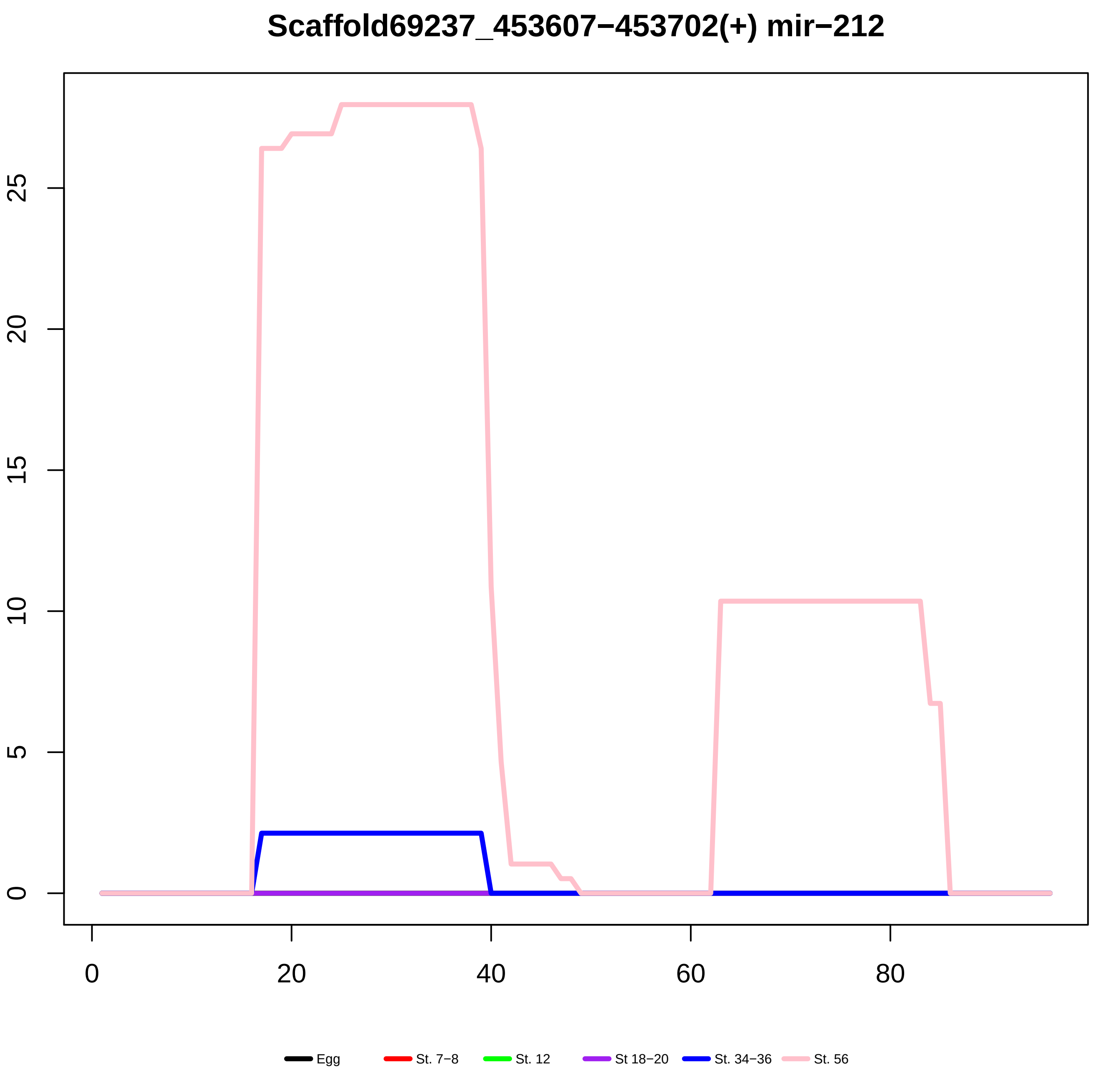

Scaffold69237_453607−453702(+) mir−212
0
5
10
15
20
25
0
20
40
60
80
Egg
St. 7−8
St. 12
St 18−20
St. 34−36
St. 56

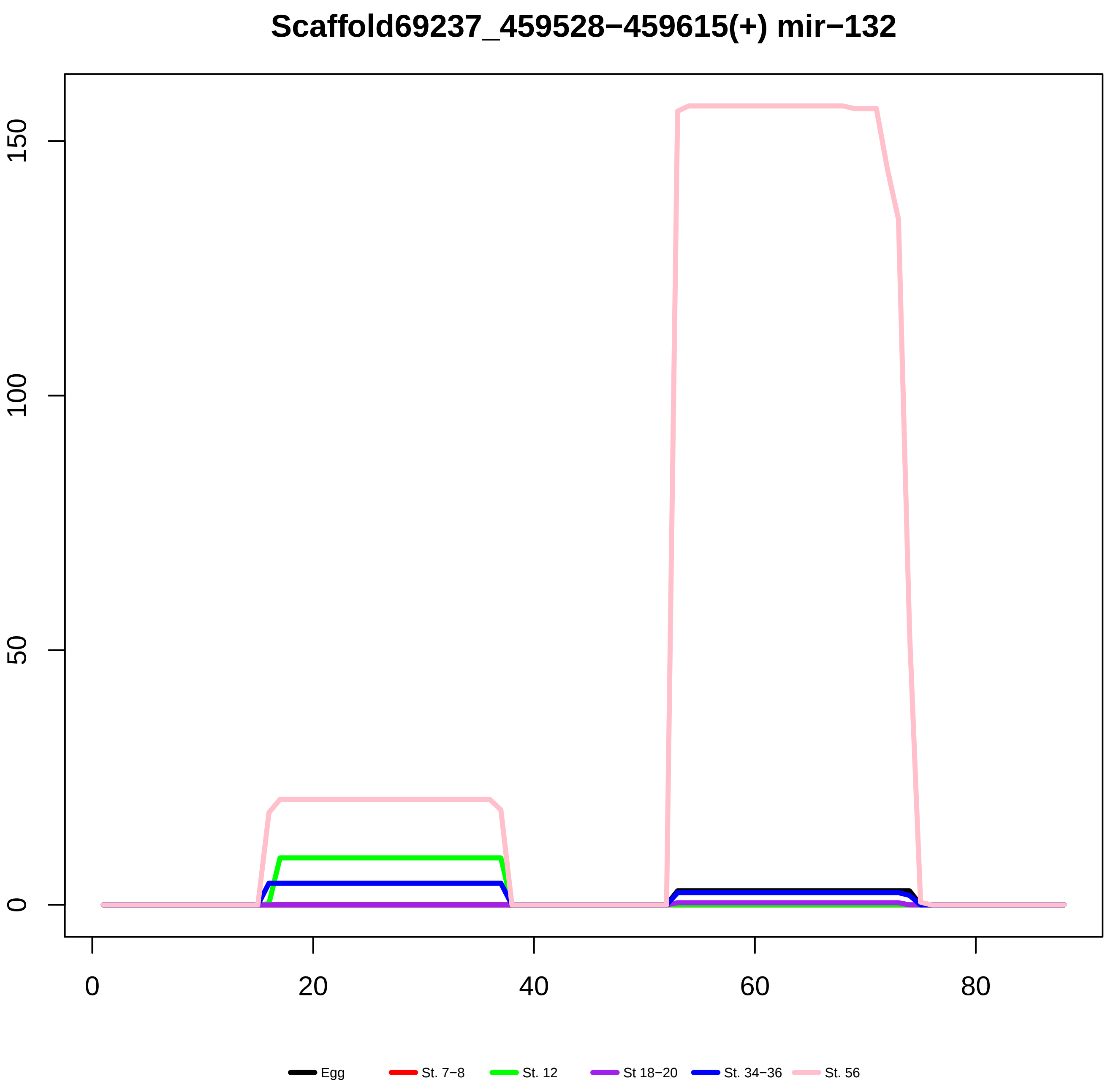

Scaffold69237_459528−459615(+) mir−132
0
50
100
150
0
20
40
60
80
Egg
St. 7−8
St. 12
St 18−20
St. 34−36
St. 56

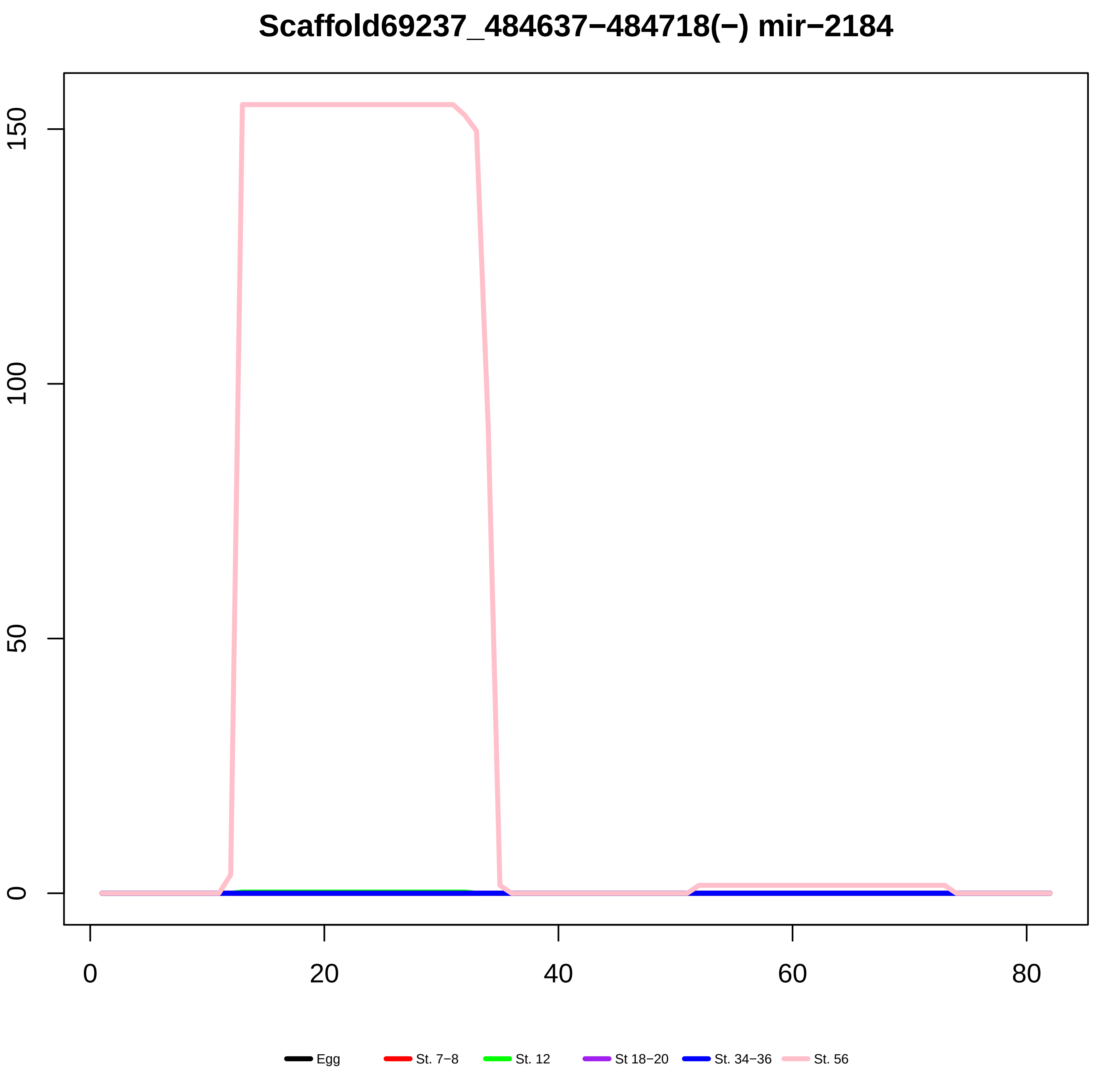

Scaffold69237_484637−484718(−) mir−2184
150
100
50
0
0
20
40
60
80
Egg
St. 7−8
St. 12
St 18−20
St. 34−36
St. 56

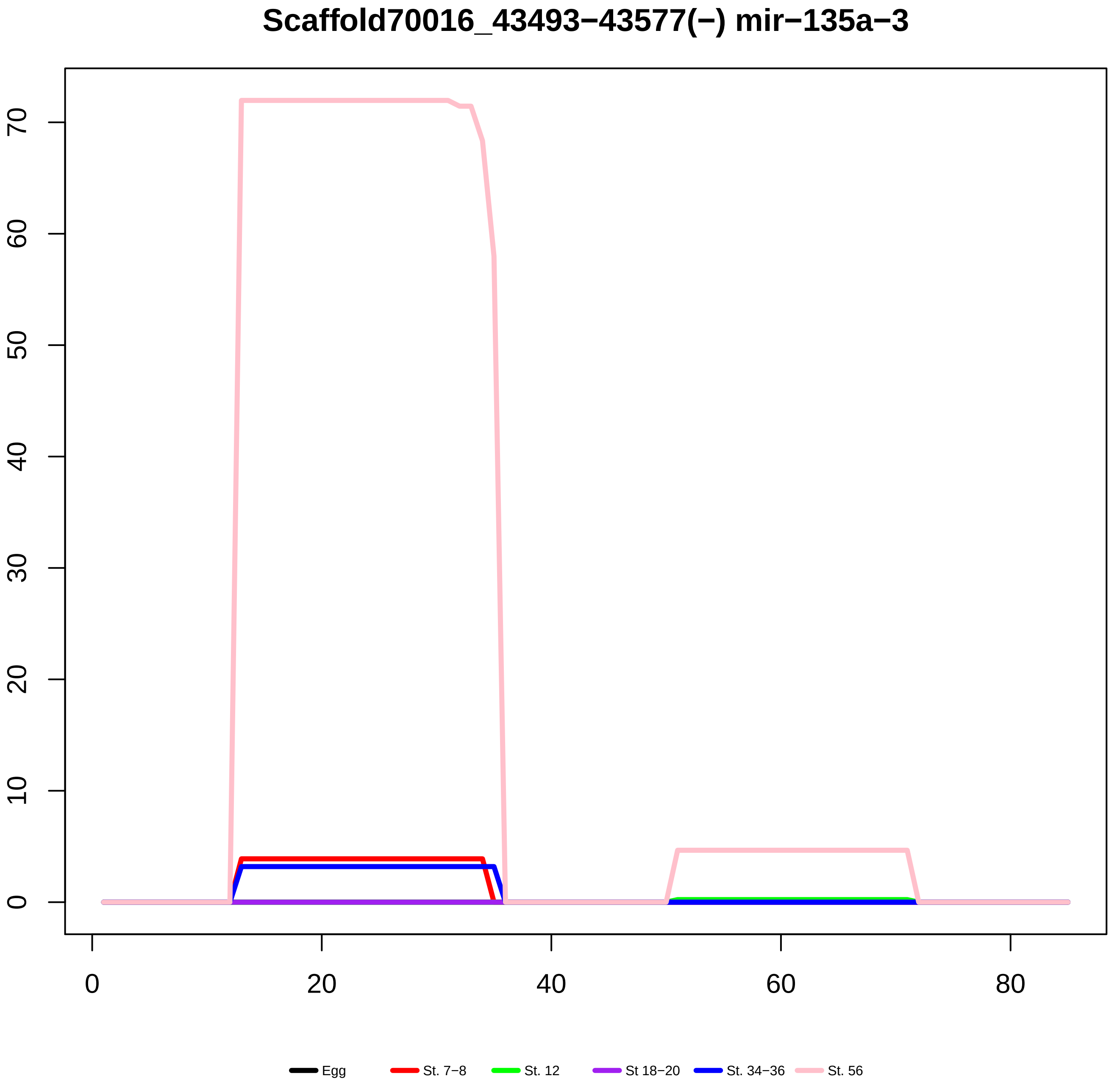
Scaffold70016_43493−43577(−) mir−135a−3
0
10
20
30
40
50
60
70
0
20
40
60
80
Egg
St. 7−8
St. 12
St 18−20
St. 34−36
St. 56

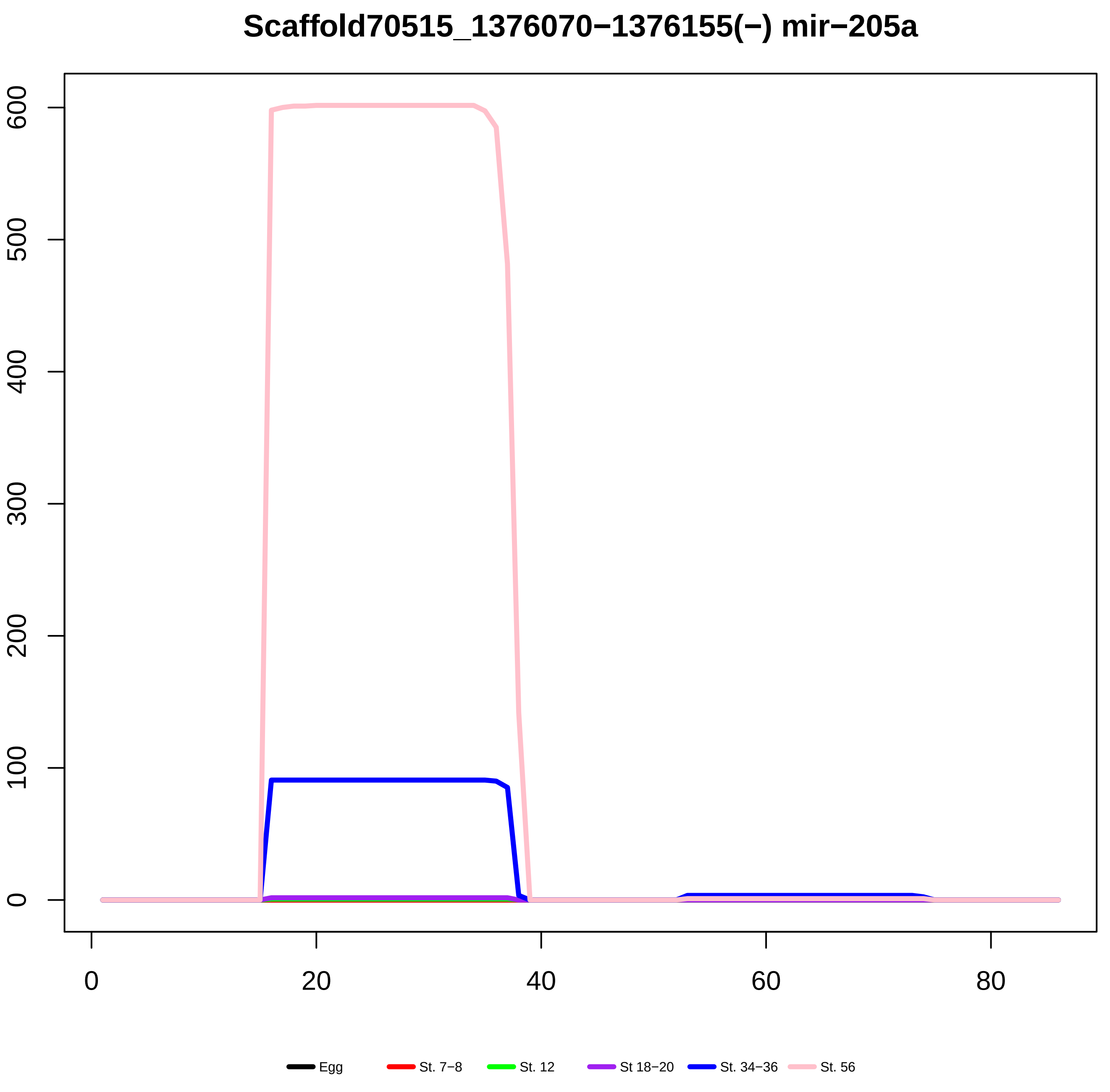

Scaffold70515_1376070−1376155(−) mir−205a
0
100
200
300
400
500
600
0
20
40
60
80
Egg
St. 7−8
St. 12
St 18−20
St. 34−36
St. 56

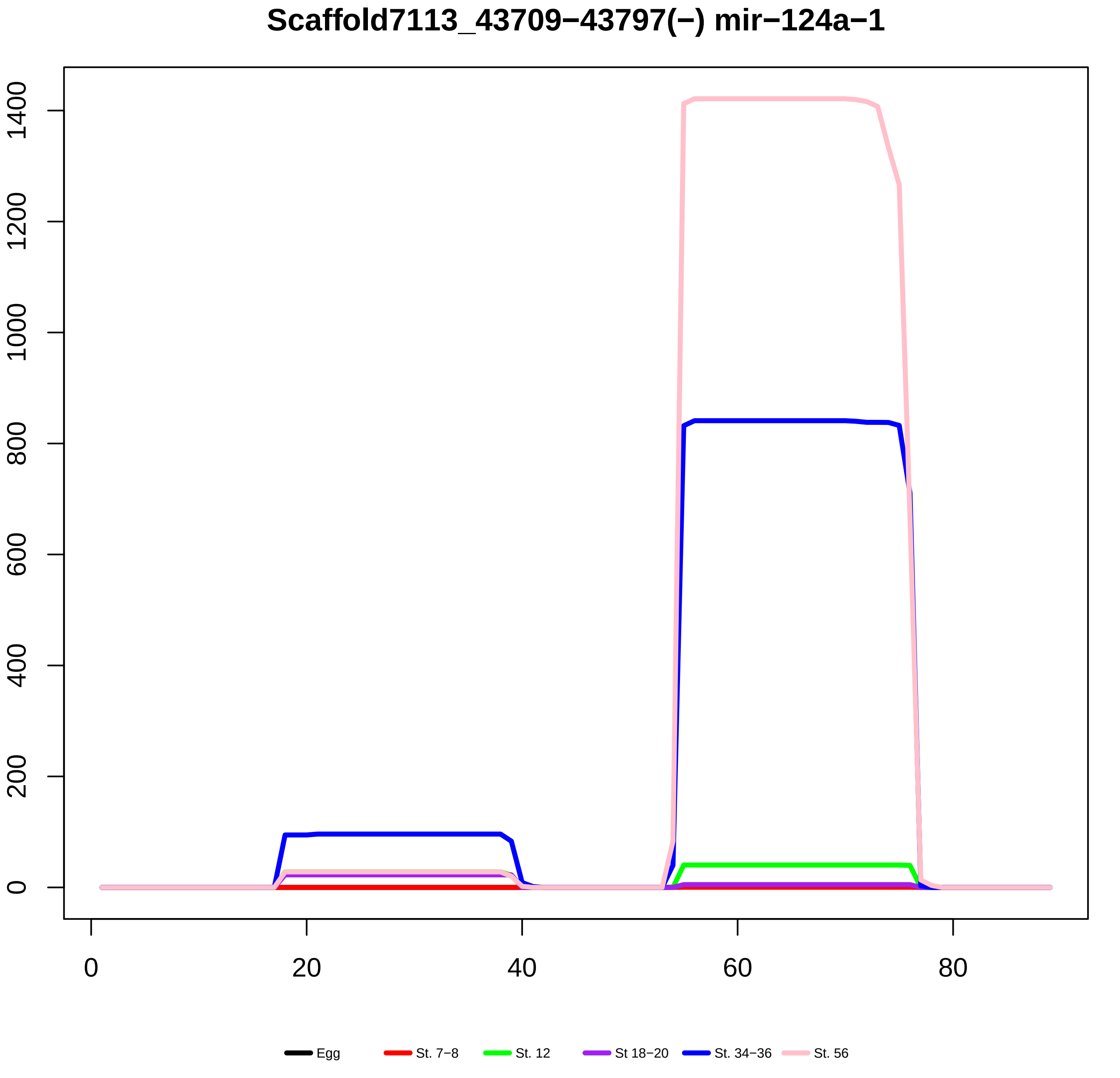

Scaffold7113_43709−43797(−) mir−124a−1
0
200
400
600
800
1000
1200
1400
0
20
40
60
80
Egg
St. 7−8
St. 12
St 18−20
St. 34−36
St. 56

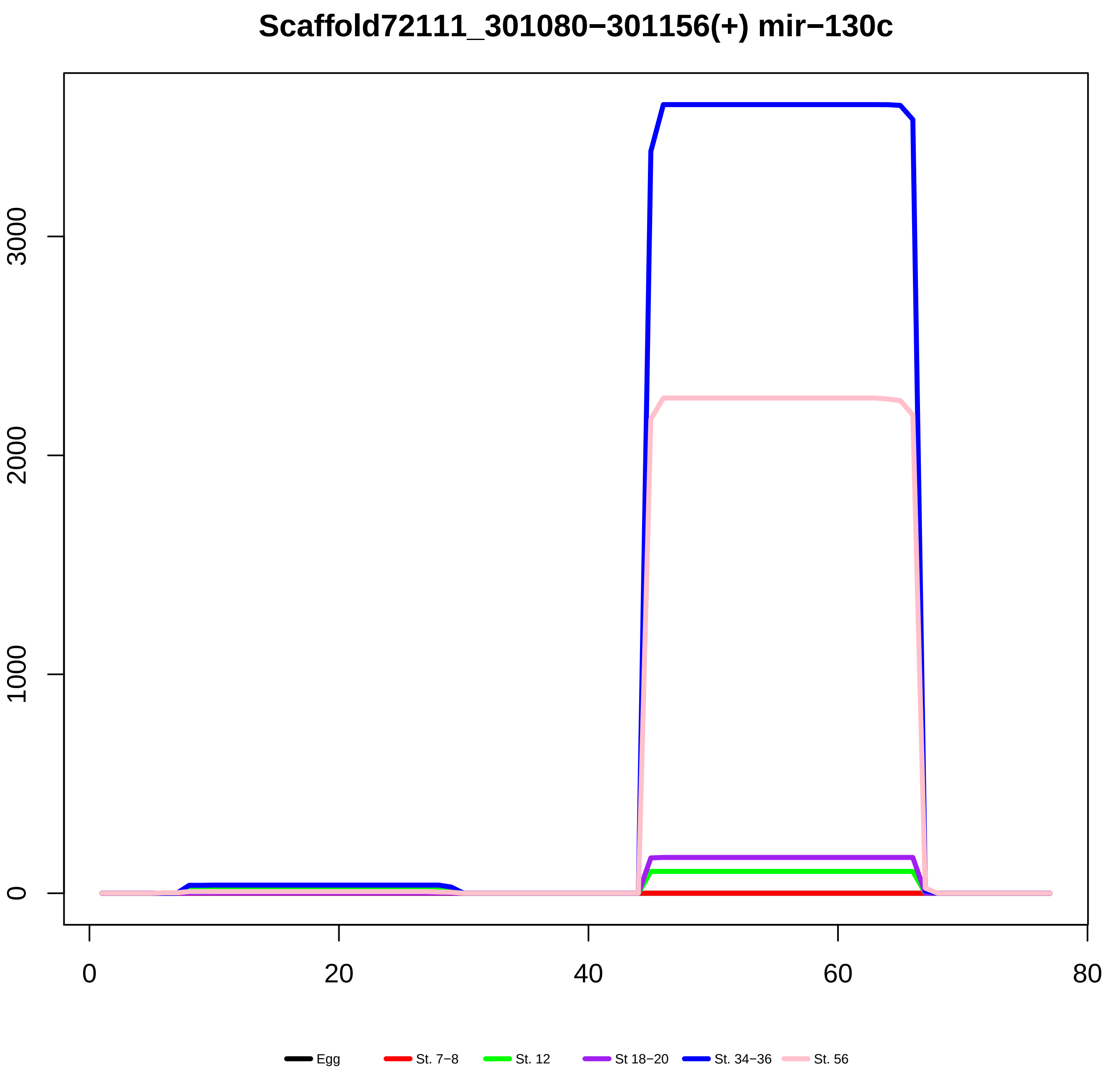

Scaffold72111_301080−301156(+) mir−130c
0
1000
2000
3000
0
20
40
60
80
Egg
St. 7−8
St. 12
St 18−20
St. 34−36
St. 56

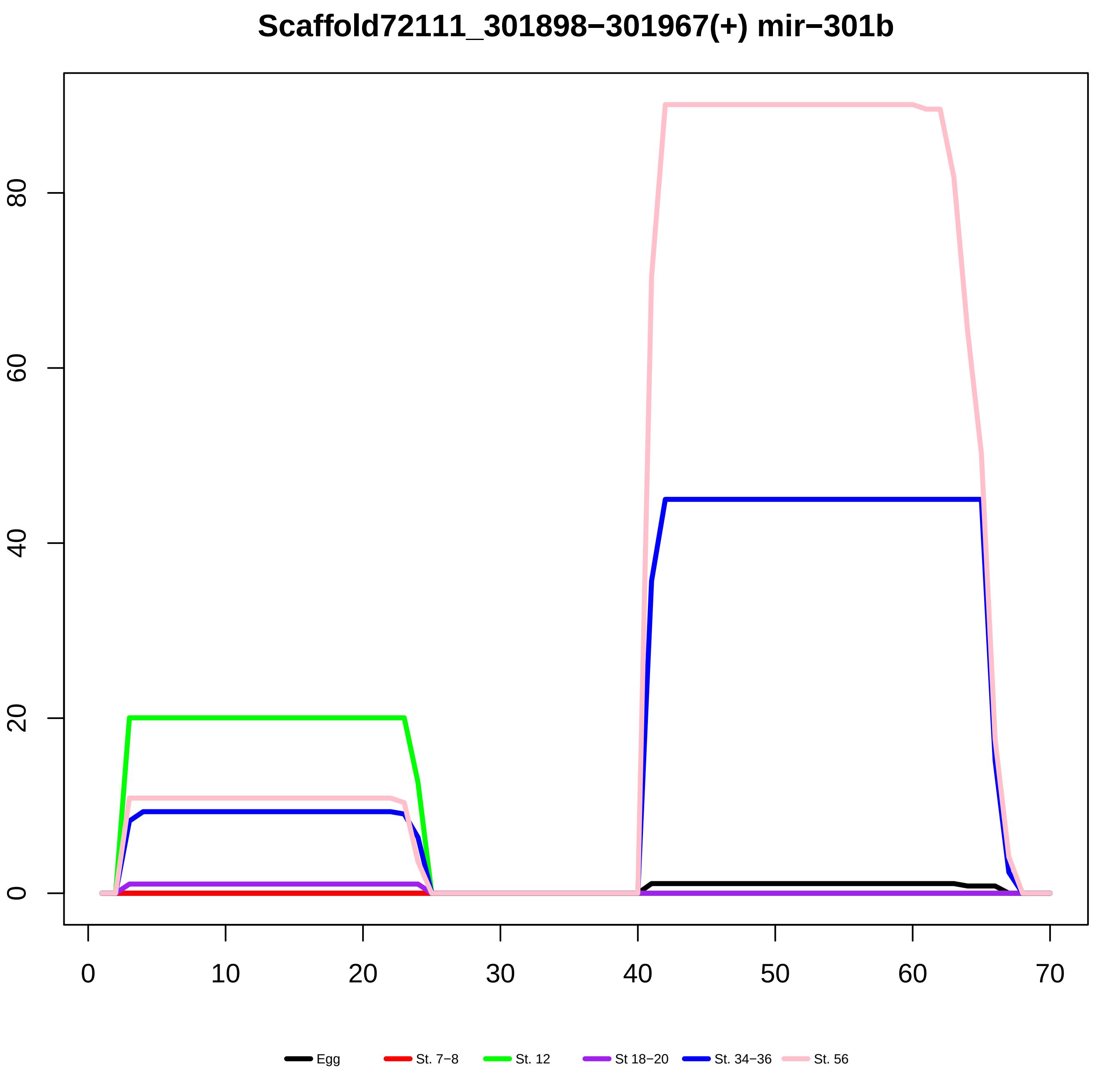
Scaffold72111_301898−301967(+) mir−301b
0
20
40
60
80
0
10
20
30
40
50
60
70
Egg
St. 7−8
St. 12
St 18−20
St. 34−36
St. 56

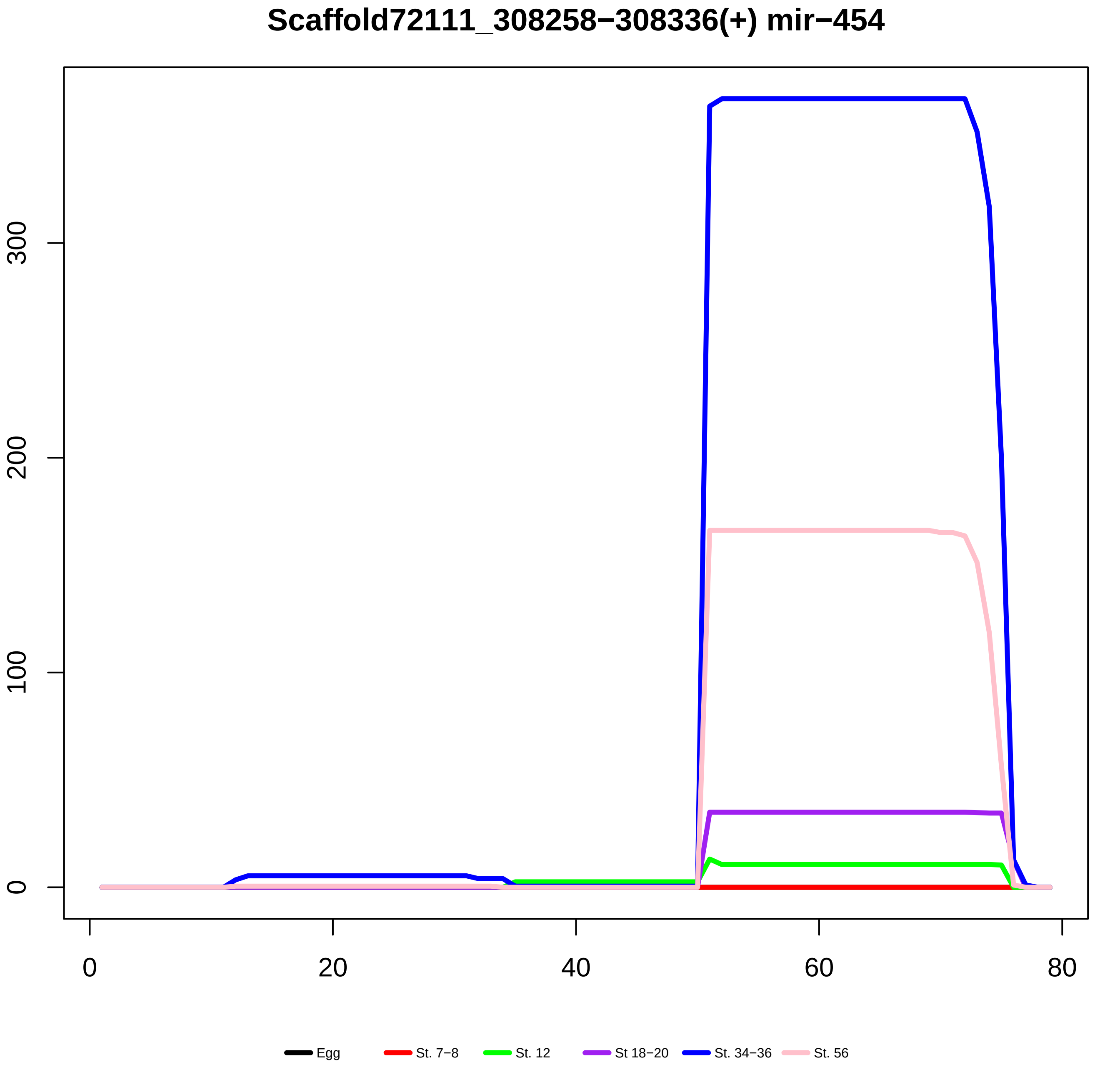

Scaffold72111_308258−308336(+) mir−454
0
100
200
300
0
20
40
60
80
Egg
St. 7−8
St. 12
St 18−20
St. 34−36
St. 56

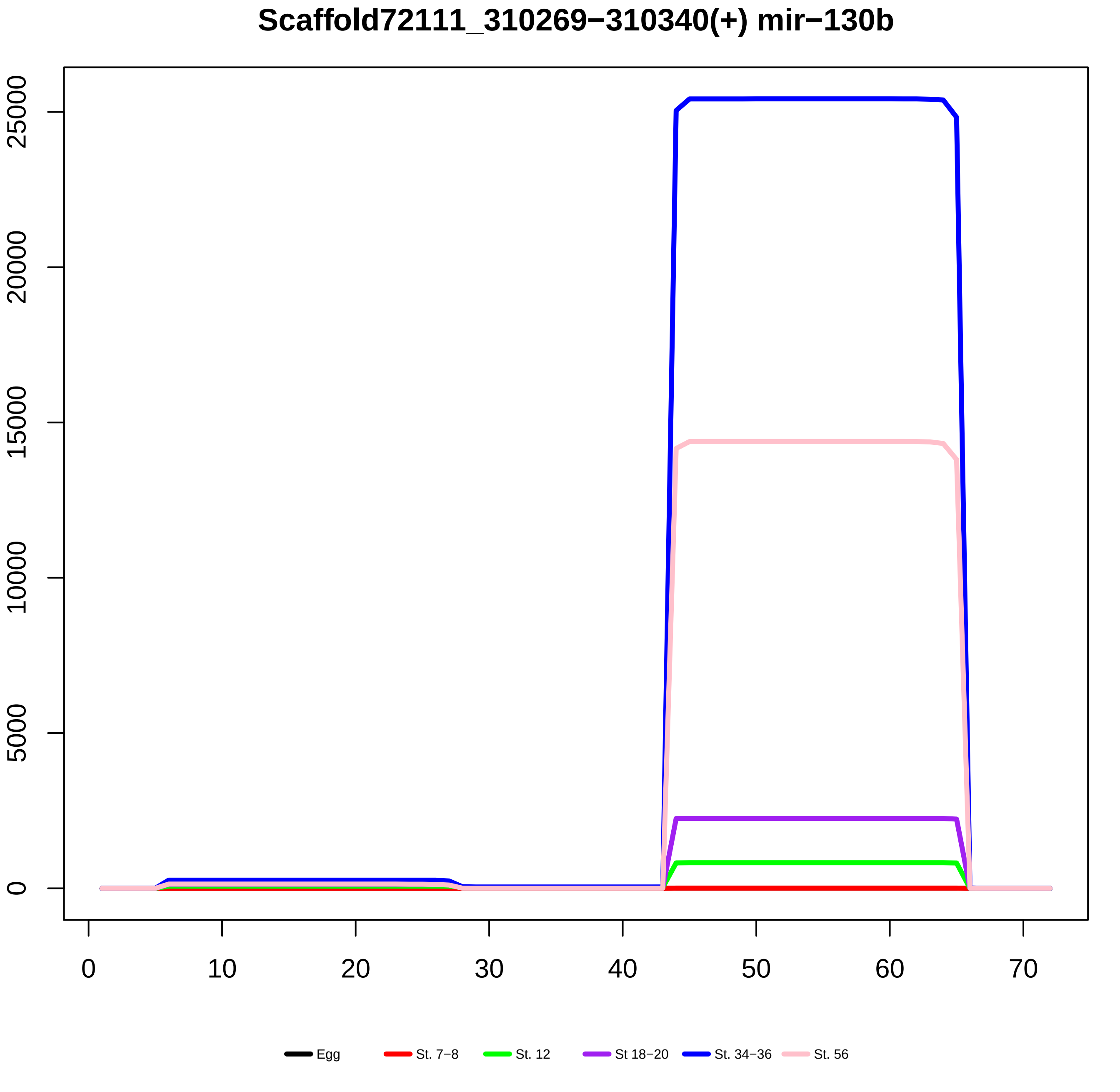
Scaffold72111_310269−310340(+) mir−130b
0
5000
10000
15000
20000
25000
0
10
20
30
40
50
60
70
Egg
St. 7−8
St. 12
St 18−20
St. 34−36
St. 56

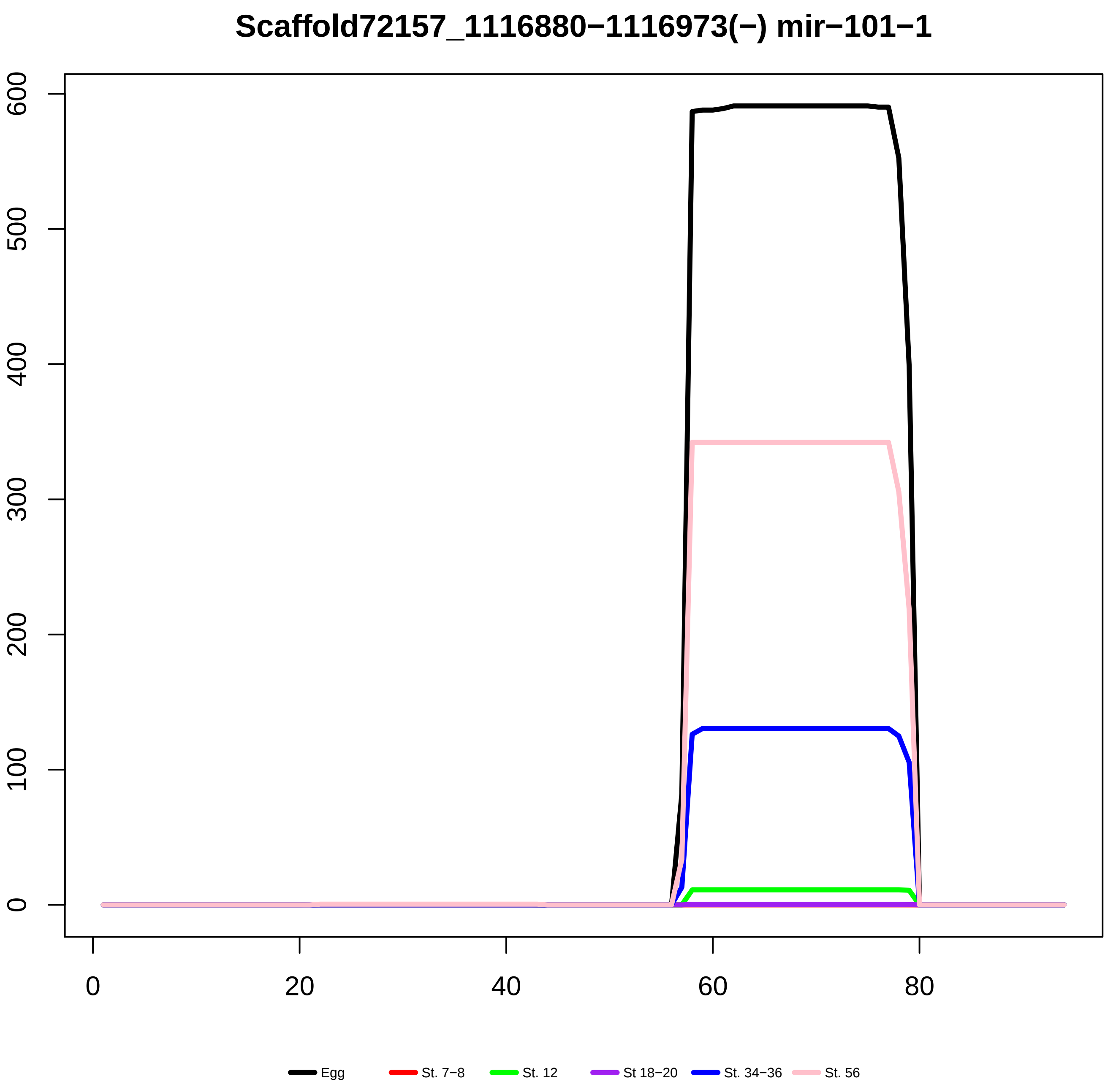

Scaffold72157_1116880−1116973(−) mir−101−1
0
100
200
300
400
500
600
0
20
40
60
80
Egg
St. 7−8
St. 12
St 18−20
St. 34−36
St. 56

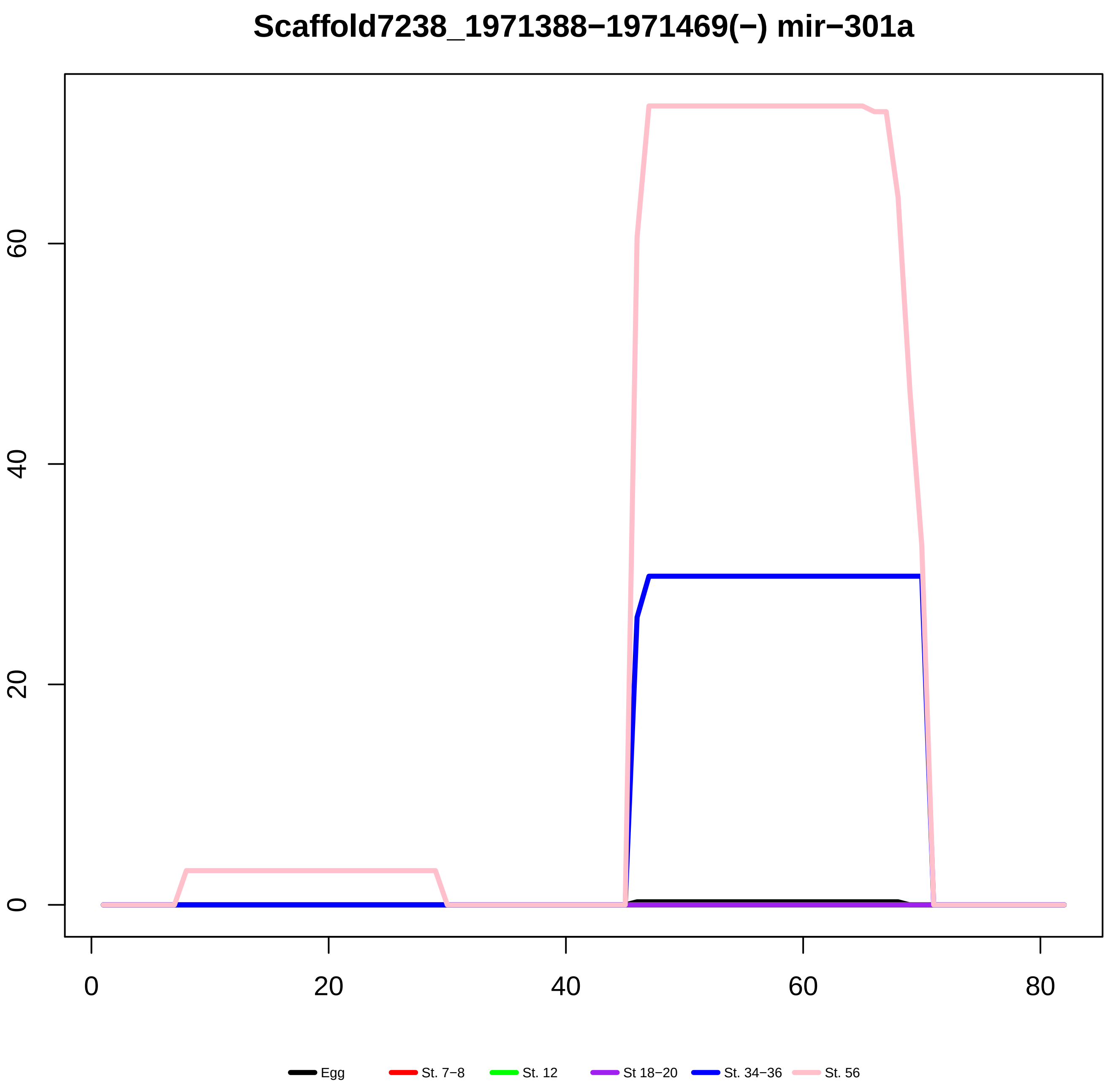
Scaffold7238_1971388−1971469(−) mir−301a
0
20
40
60
0
20
40
60
80
Egg
St. 7−8
St. 12
St 18−20
St. 34−36
St. 56

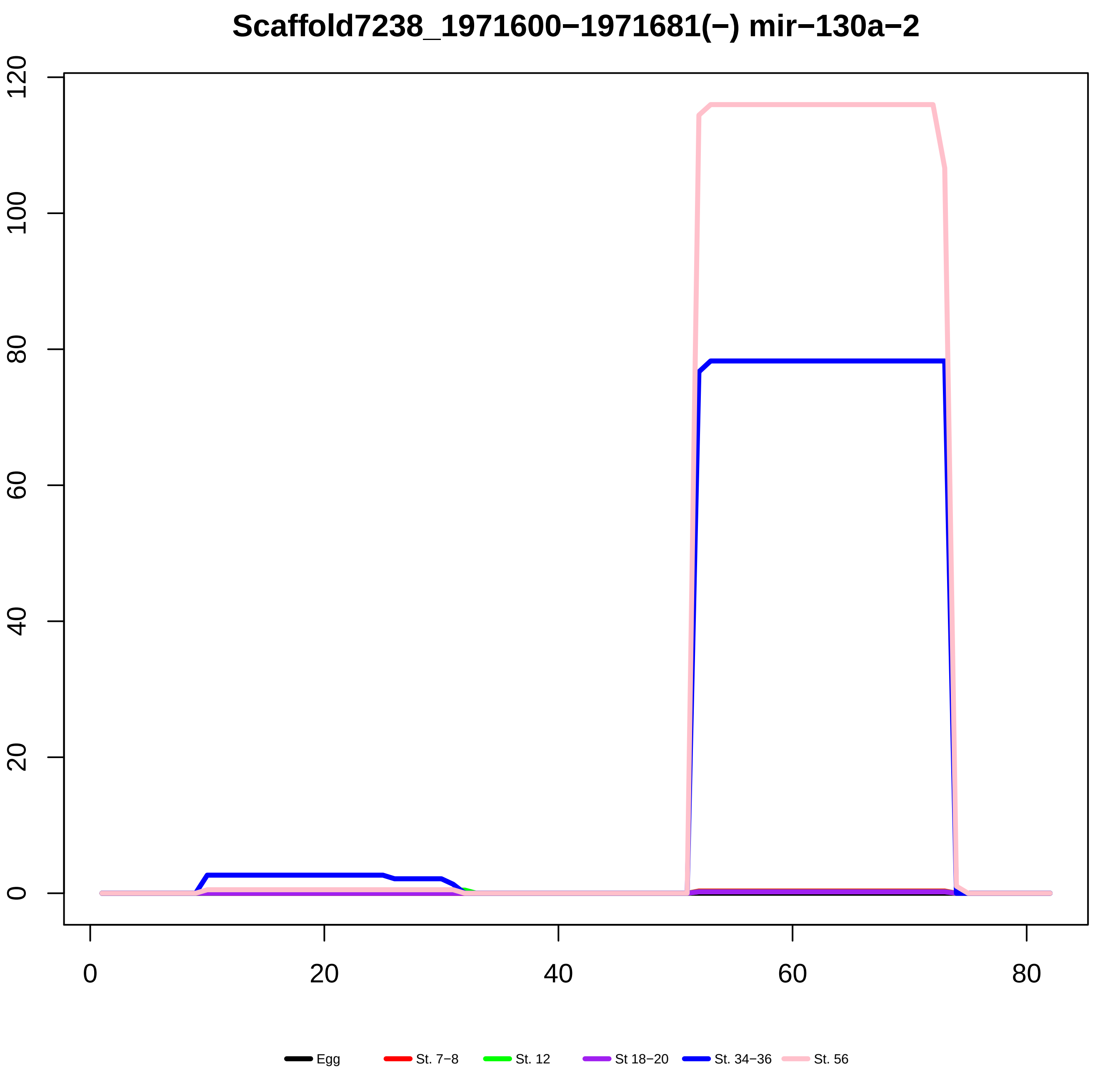

Scaffold7238_1971600−1971681(−) mir−130a−2
0
20
40
60
80
100
120
0
20
40
60
80
Egg
St. 7−8
St. 12
St 18−20
St. 34−36
St. 56

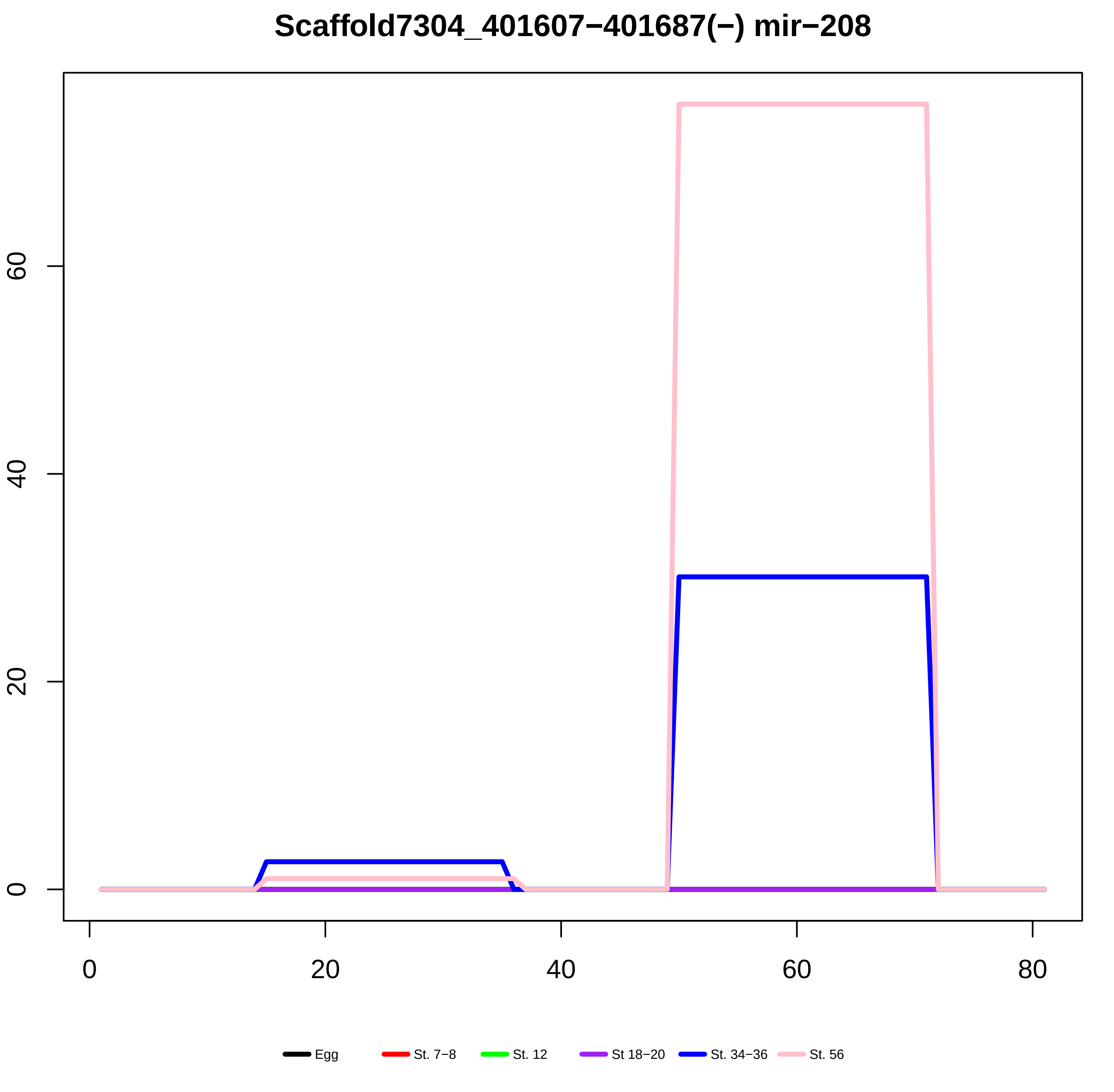

Scaffold7304_401607−401687(−) mir−208
0
20
40
60
0
20
40
60
80
Egg
St. 7−8
St. 12
St 18−20
St. 34−36
St. 56

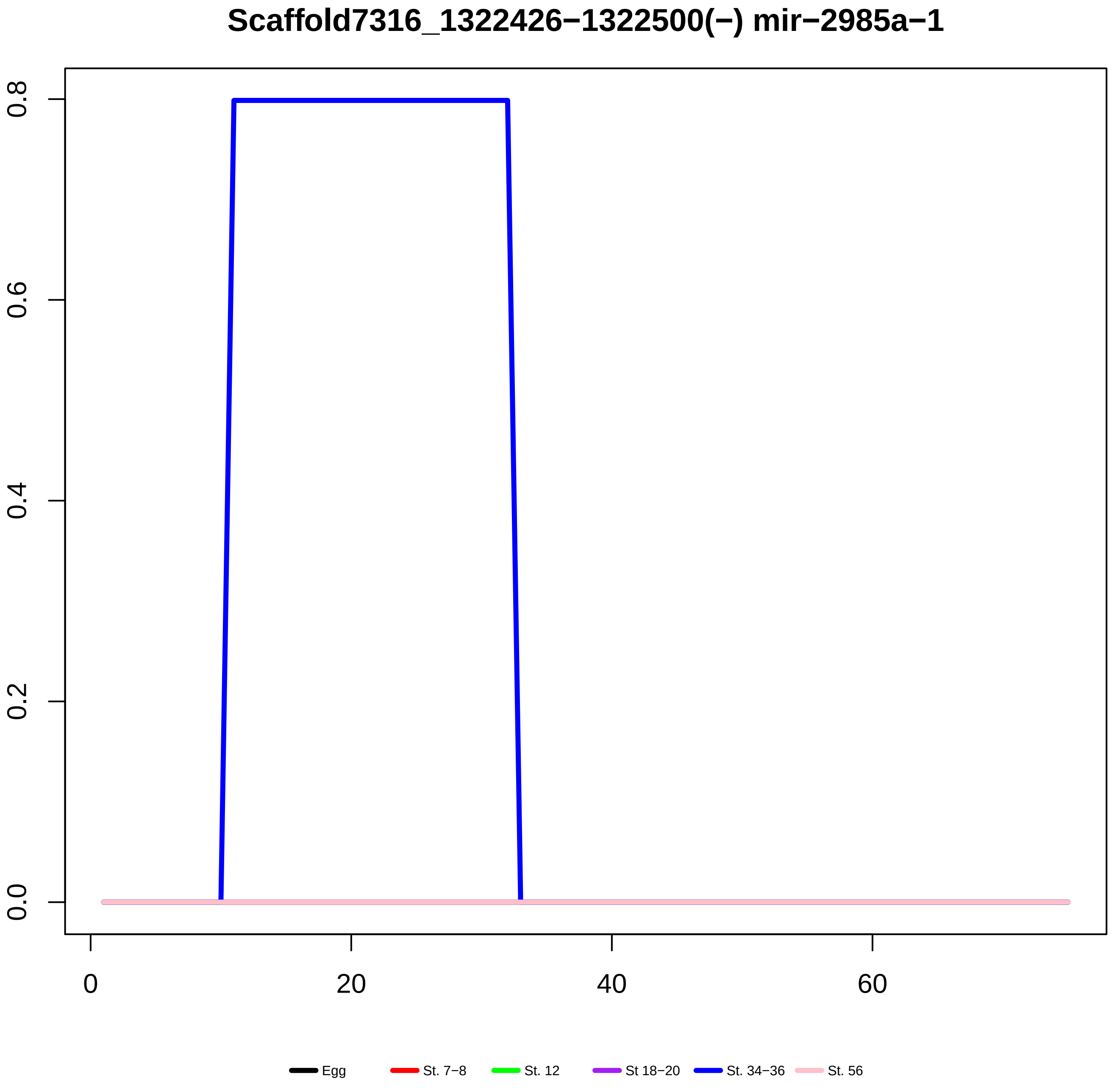
Scaffold7316_1322426−1322500(−) mir−2985a−1
0.0
0.2
0.4
0.6
0.8
0
20
40
60
Egg
St. 7−8
St. 12
St 18−20
St. 34−36
St. 56

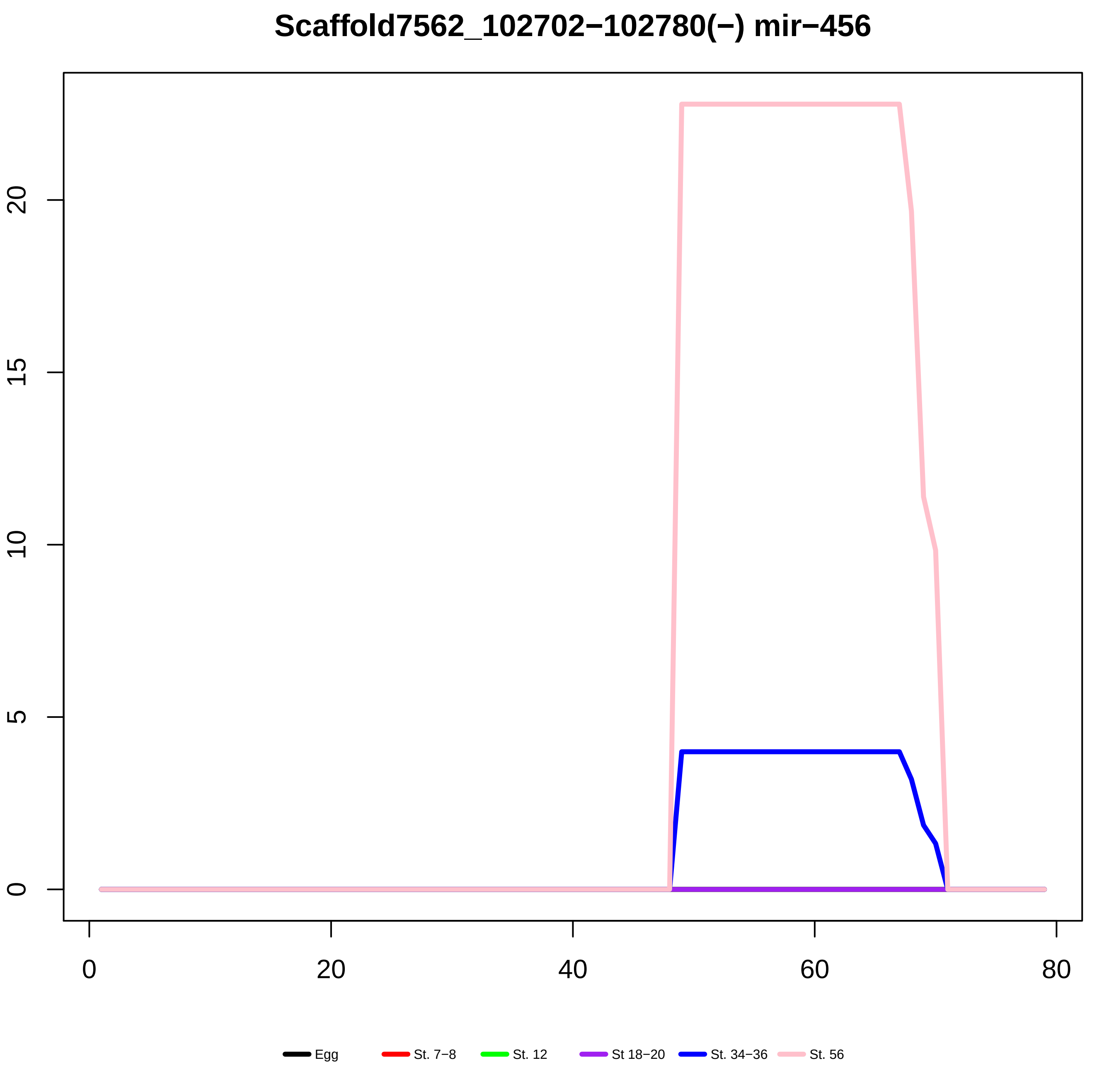
Scaffold7562_102702−102780(−) mir−456
0
5
10
15
20
0
20
40
60
80
Egg
St. 7−8
St. 12
St 18−20
St. 34−36
St. 56

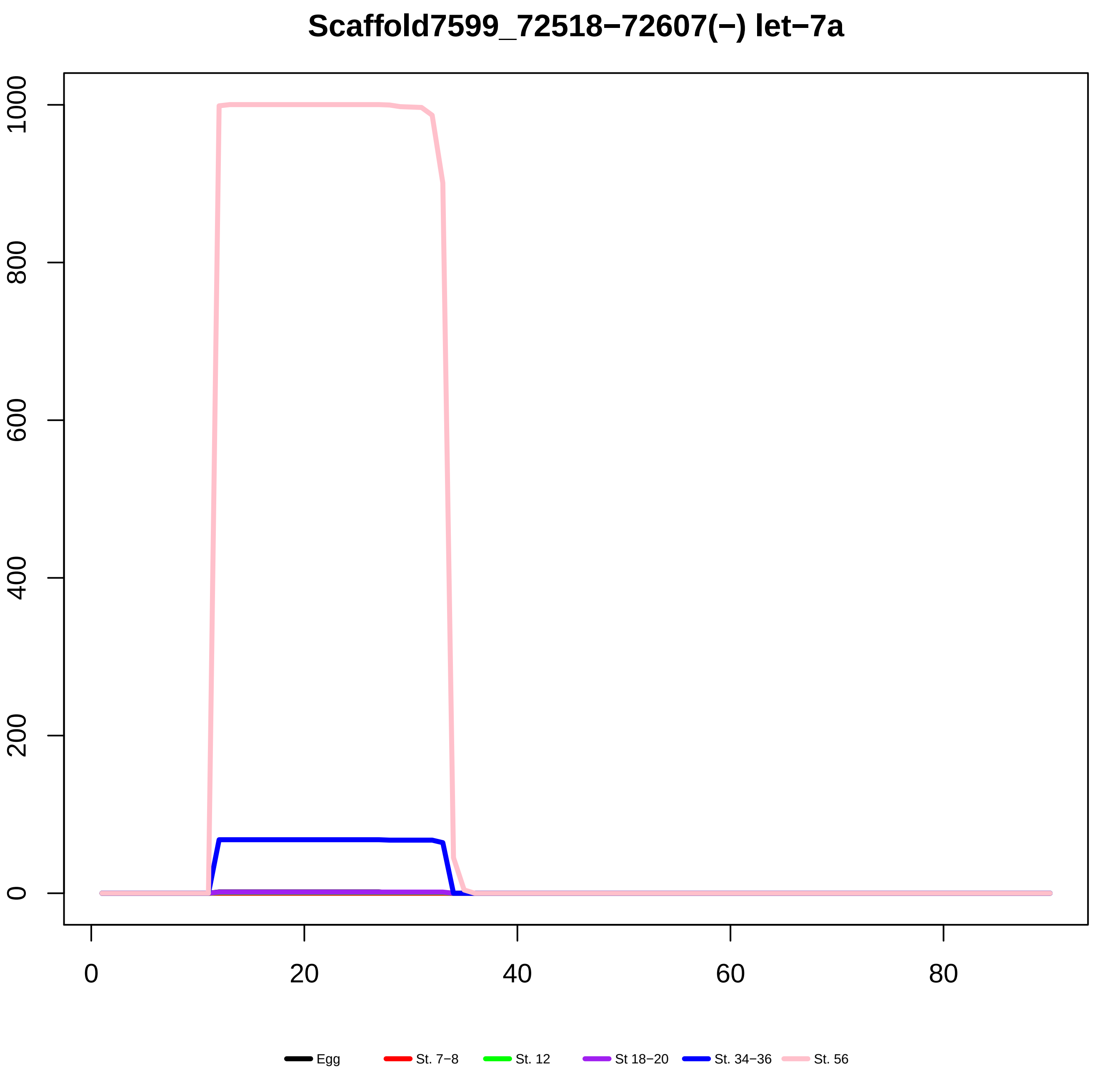
Scaffold7599_72518−72607(−) let−7a
0
200
400
600
800
1000
0
20
40
60
80
Egg
St. 7−8
St. 12
St 18−20
St. 34−36
St. 56

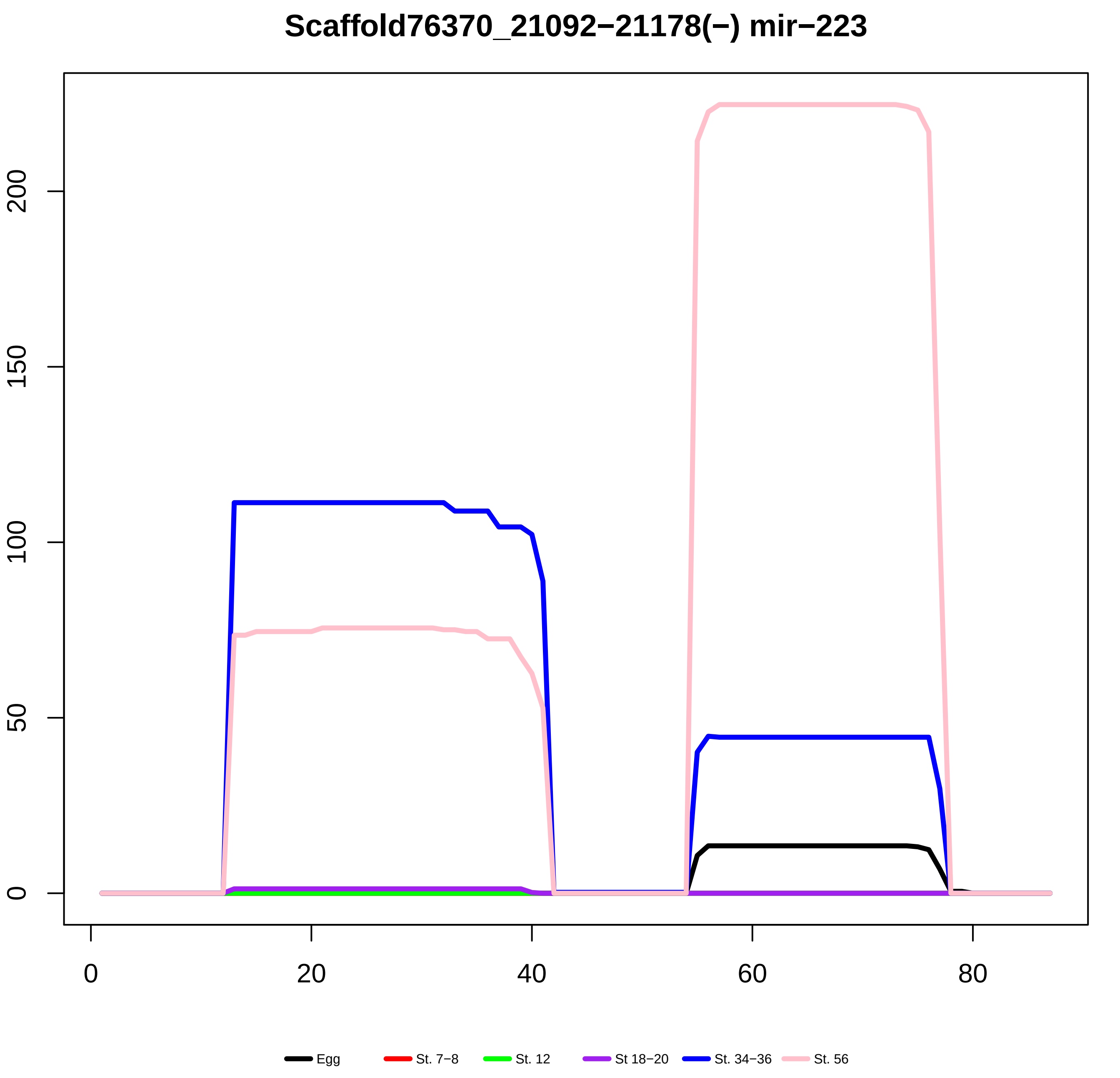
Scaffold76370_21092−21178(−) mir−223
0
50
100
150
200
0
20
40
60
80
Egg
St. 7−8
St. 12
St 18−20
St. 34−36
St. 56

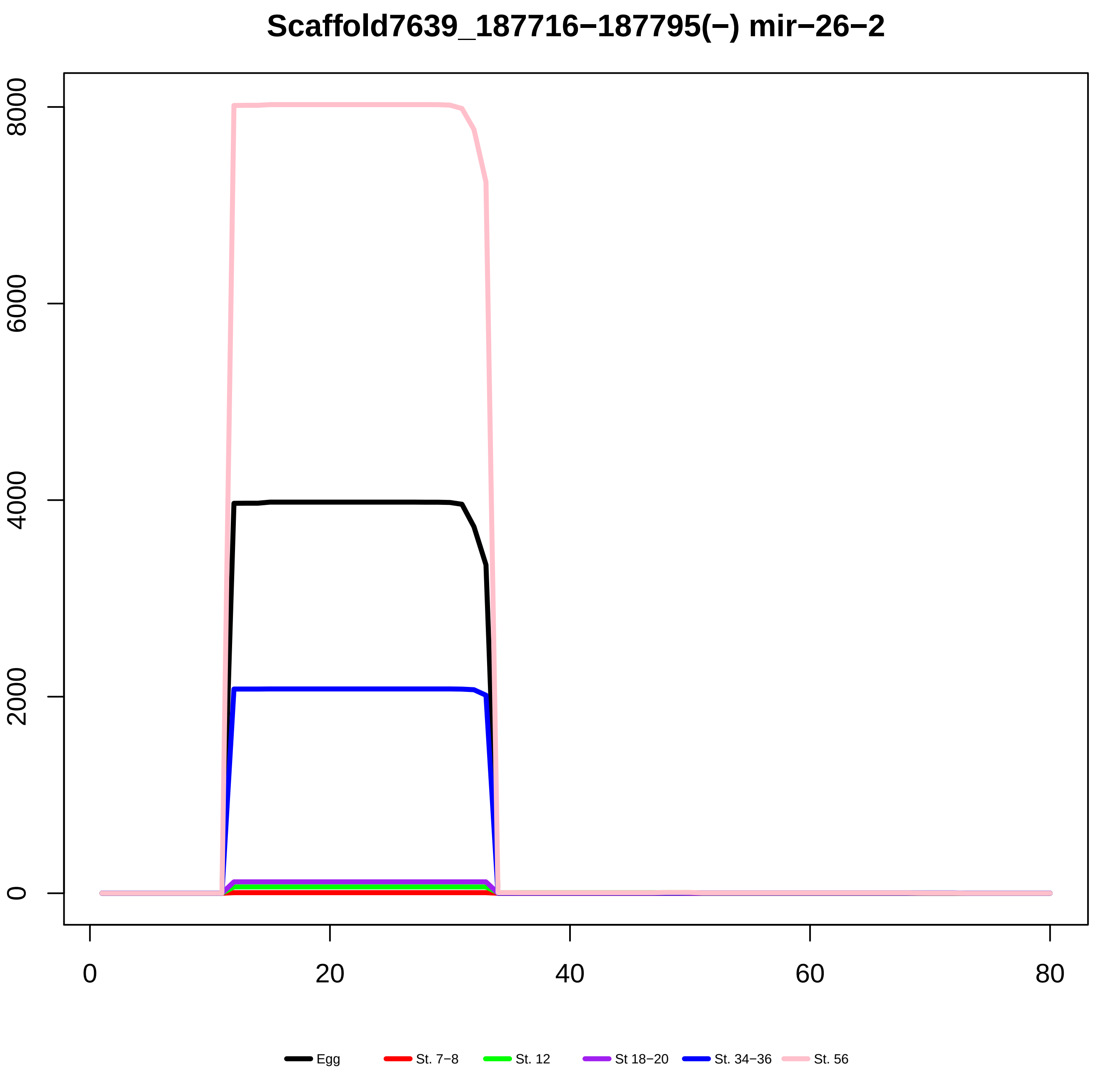
Scaffold7639_187716−187795(−) mir−26−2
0
2000
4000
6000
8000
0
20
40
60
80
Egg
St. 7−8
St. 12
St 18−20
St. 34−36
St. 56

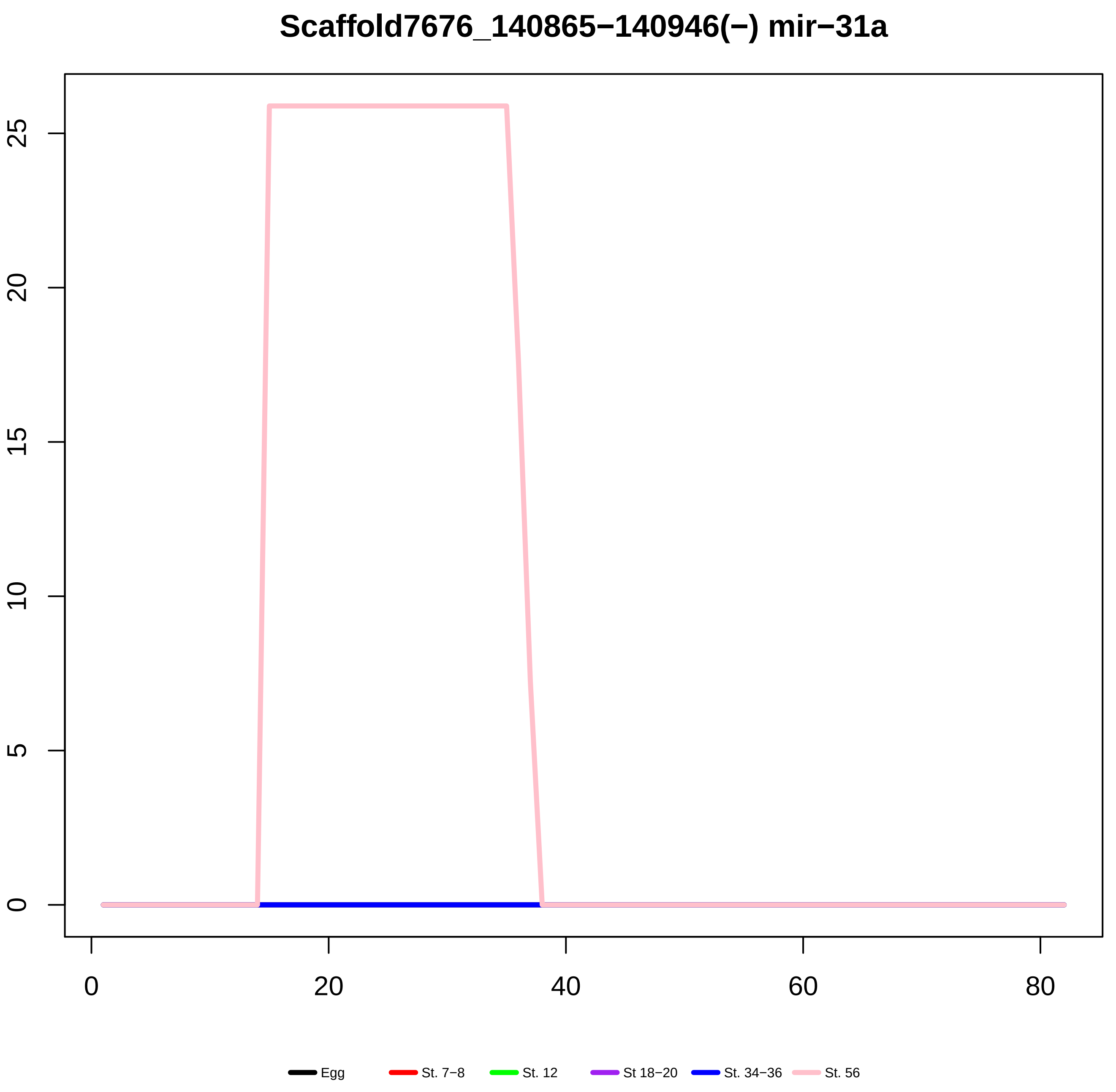

Scaffold7676_140865−140946(−) mir−31a
0
5
10
15
20
25
0
20
40
60
80
Egg
St. 7−8
St. 12
St 18−20
St. 34−36
St. 56

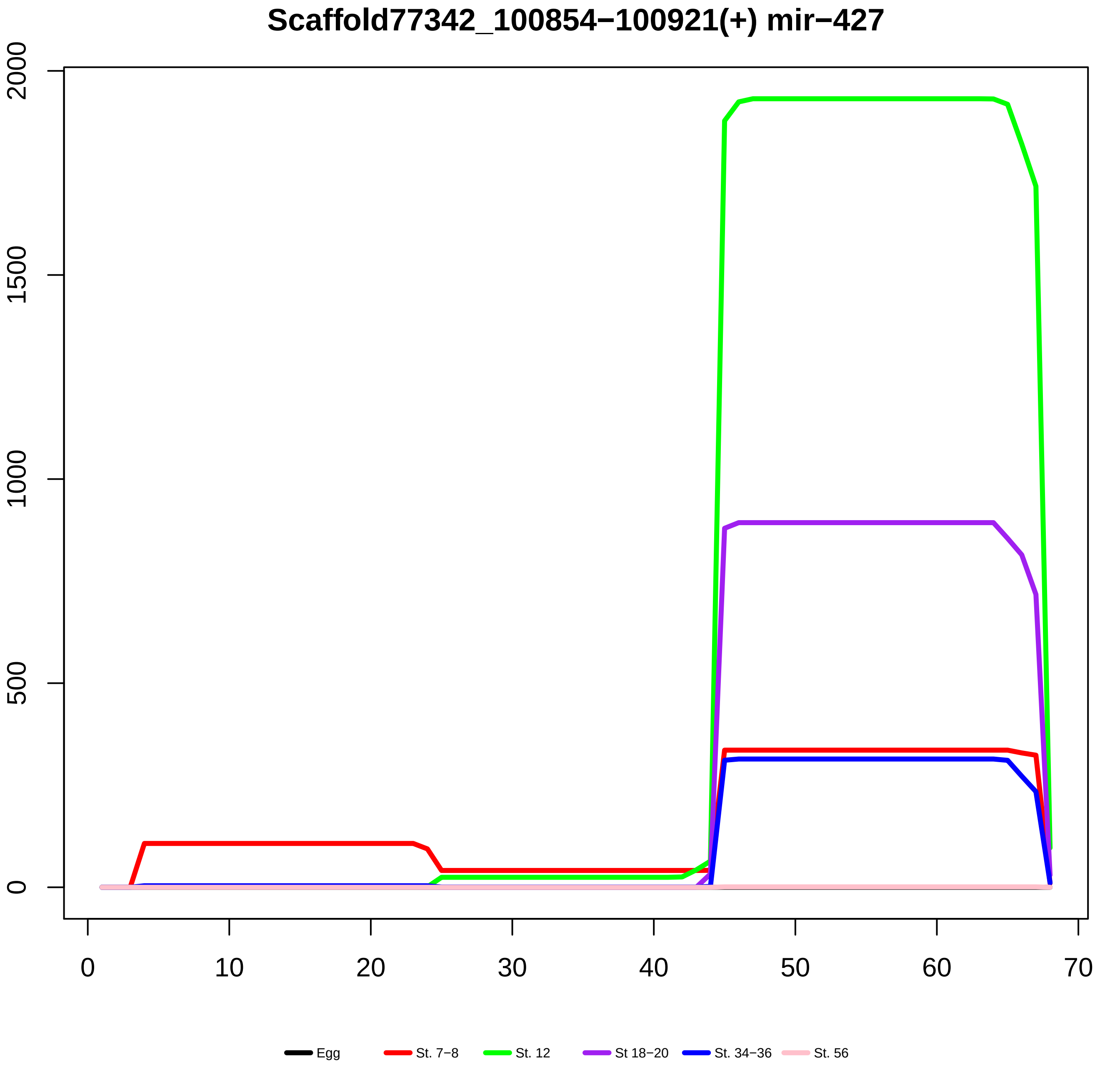

Scaffold77342_100854−100921(+) mir−427
0
500
1000
1500
2000
0
10
20
30
40
50
60
70
Egg
St. 7−8
St. 12
St 18−20
St. 34−36
St. 56

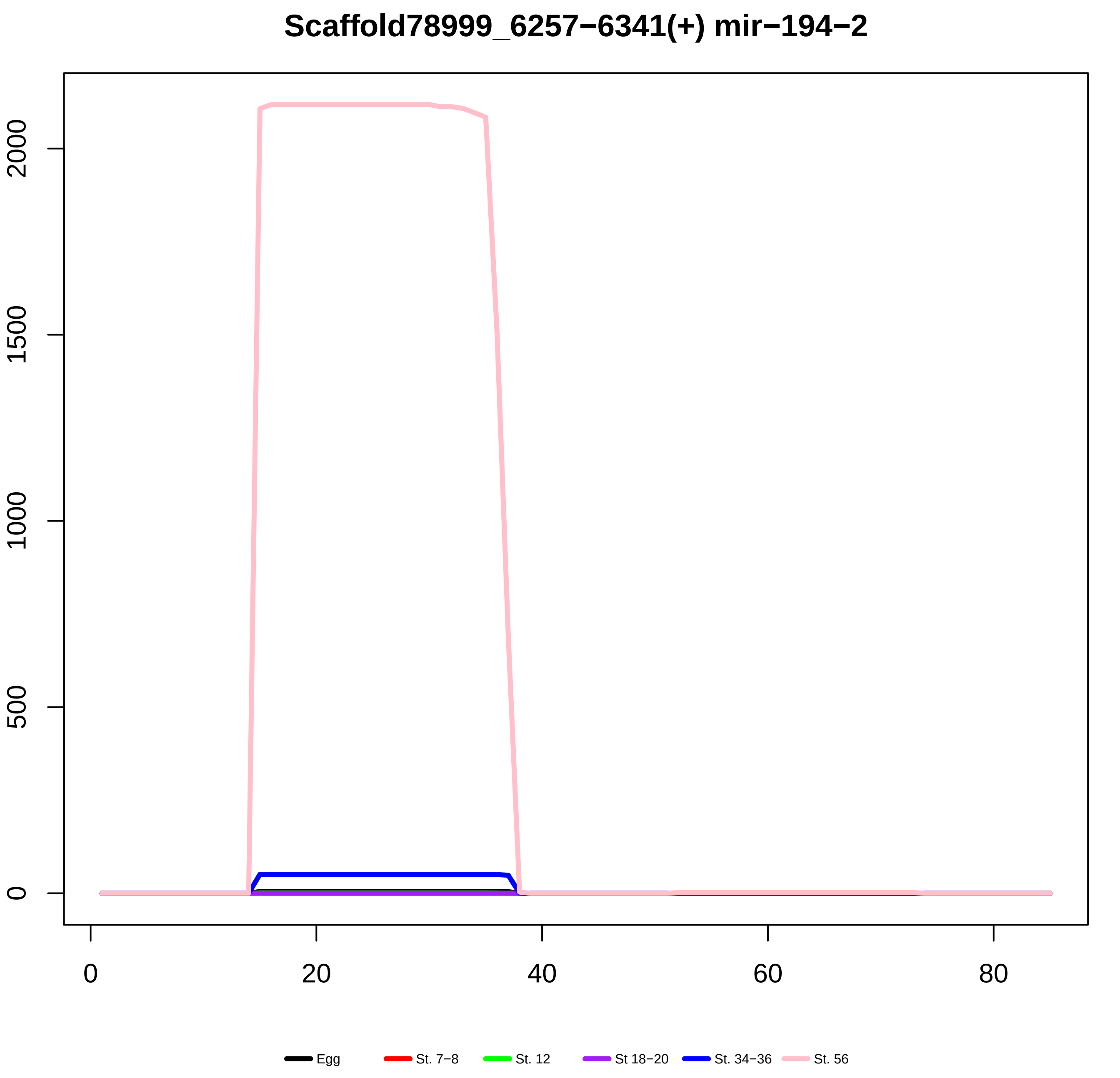

Scaffold78999_6257−6341(+) mir−194−2
0
500
1000
1500
2000
0
20
40
60
80
Egg
St. 7−8
St. 12
St 18−20
St. 34−36
St. 56

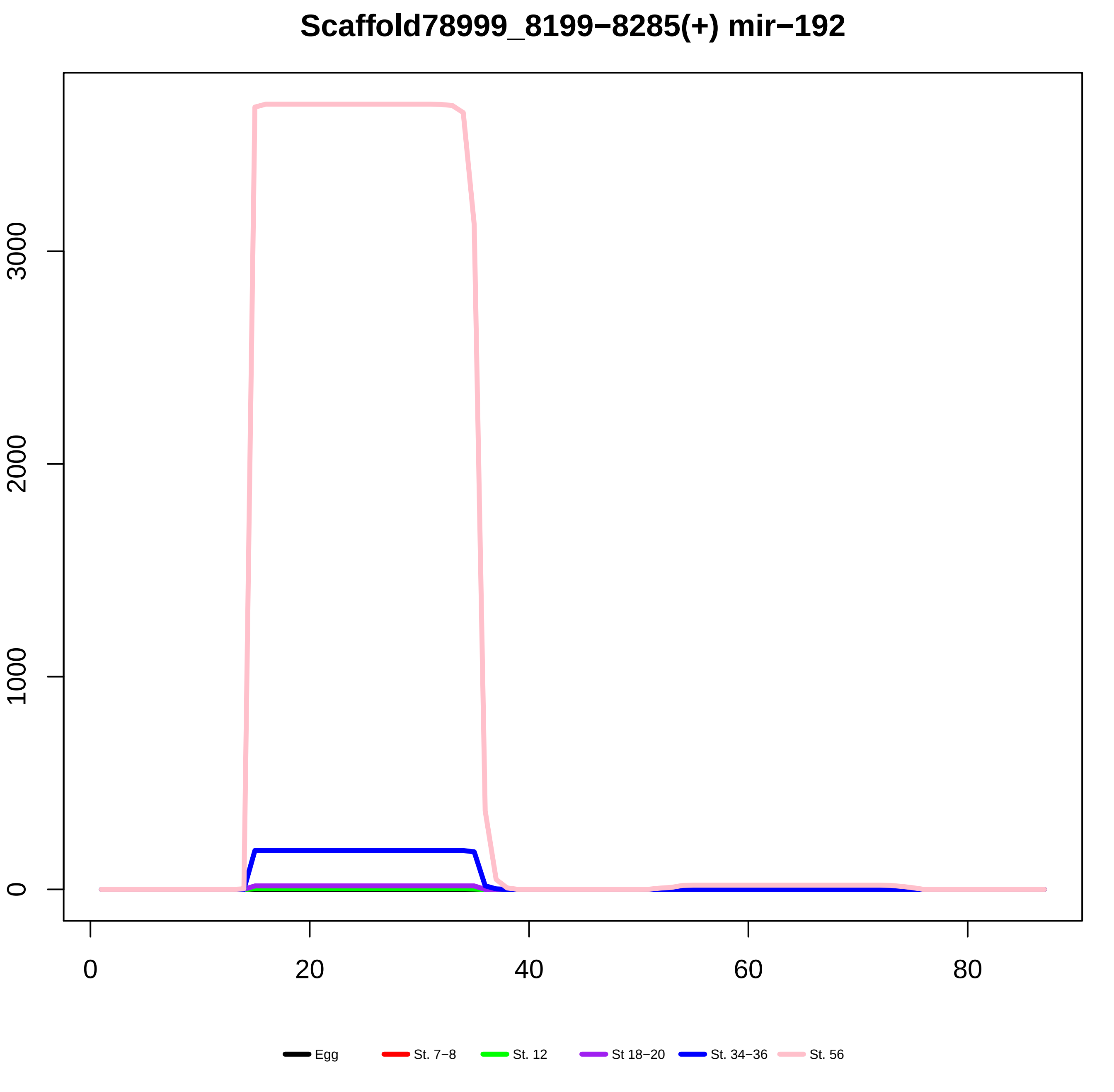

Scaffold78999_8199−8285(+) mir−192
0
1000
2000
3000
0
20
40
60
80
Egg
St. 7−8
St. 12
St 18−20
St. 34−36
St. 56

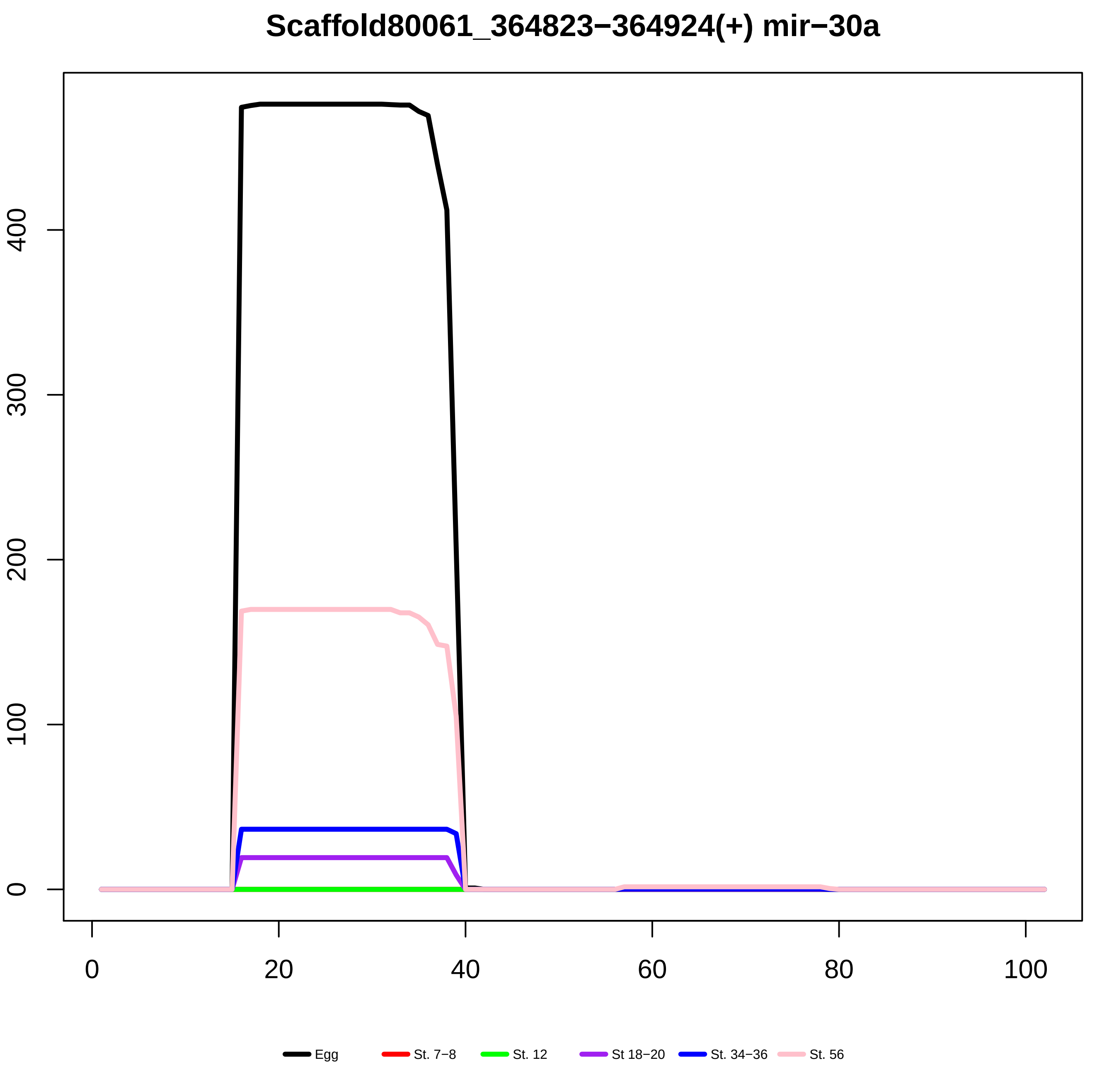

Scaffold80061_364823−364924(+) mir−30a
0
100
200
300
400
0
20
40
60
80
100
Egg
St. 7−8
St. 12
St 18−20
St. 34−36
St. 56

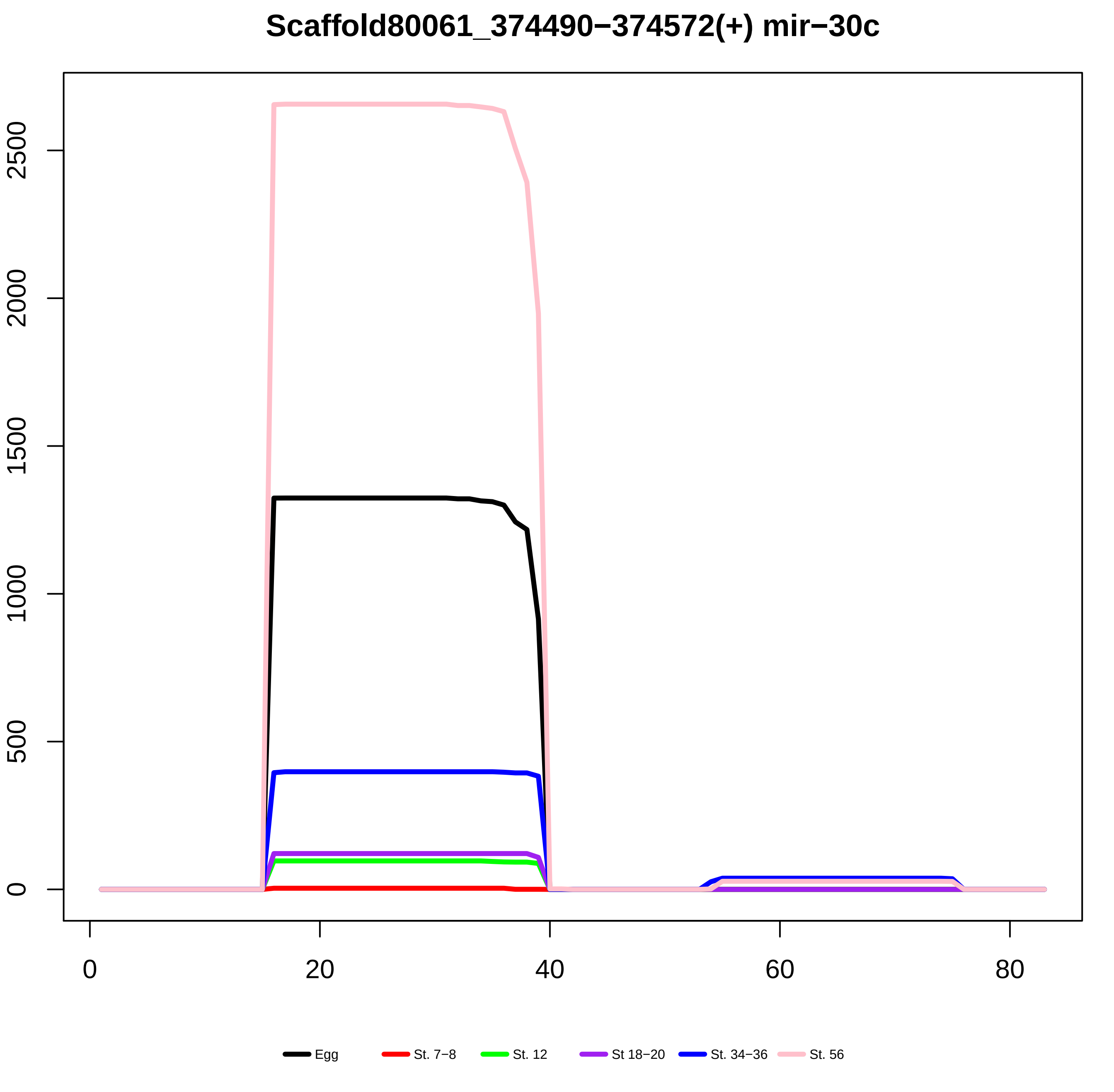

Scaffold80061_374490−374572(+) mir−30c
0
500
1000
1500
2000
2500
0
20
40
60
80
Egg
St. 7−8
St. 12
St 18−20
St. 34−36
St. 56

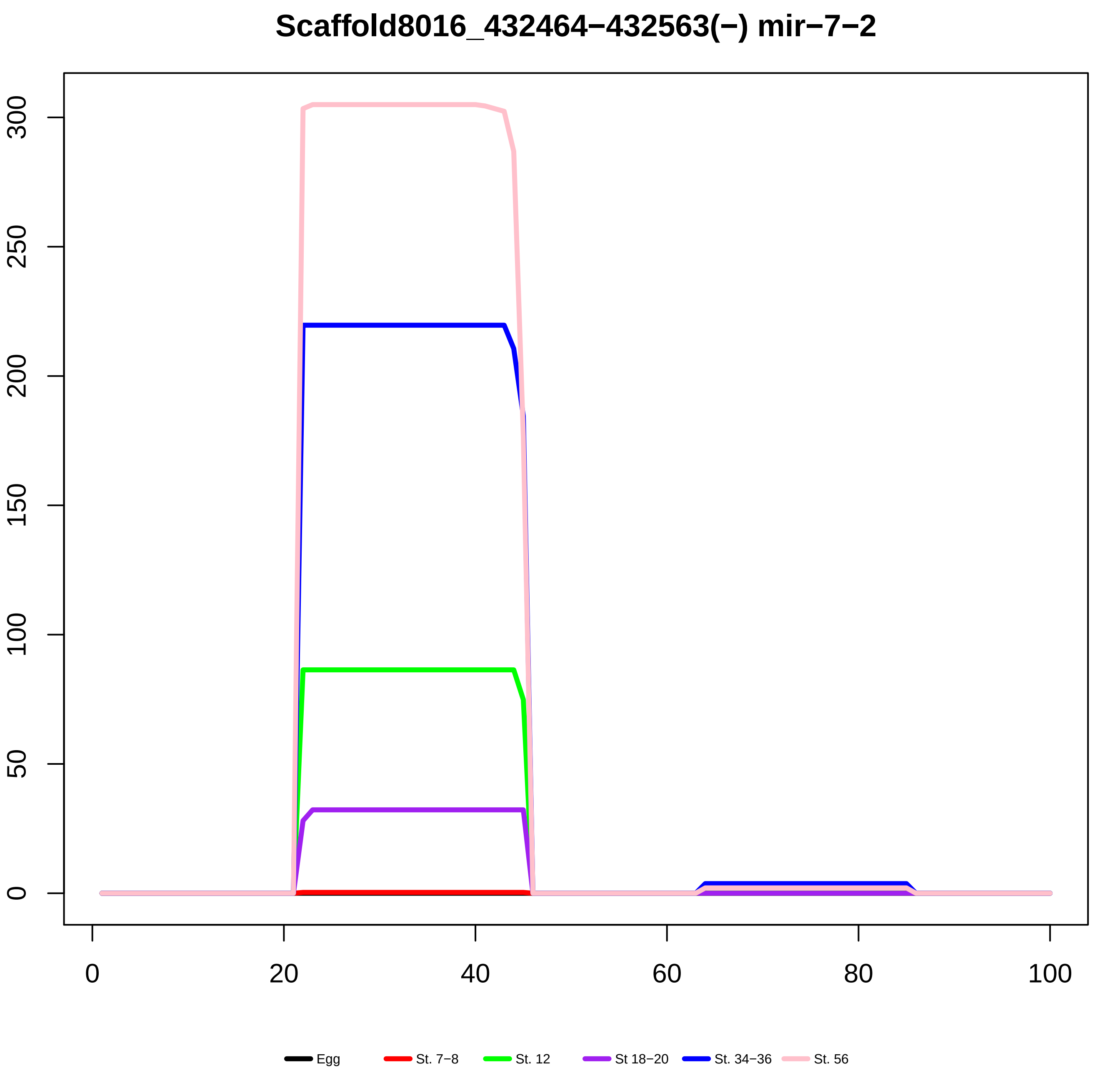

Scaffold8016_432464−432563(−) mir−7−2
300
250
200
150
100
50
0
0
20
40
60
80
100
Egg
St. 7−8
St. 12
St 18−20
St. 34−36
St. 56

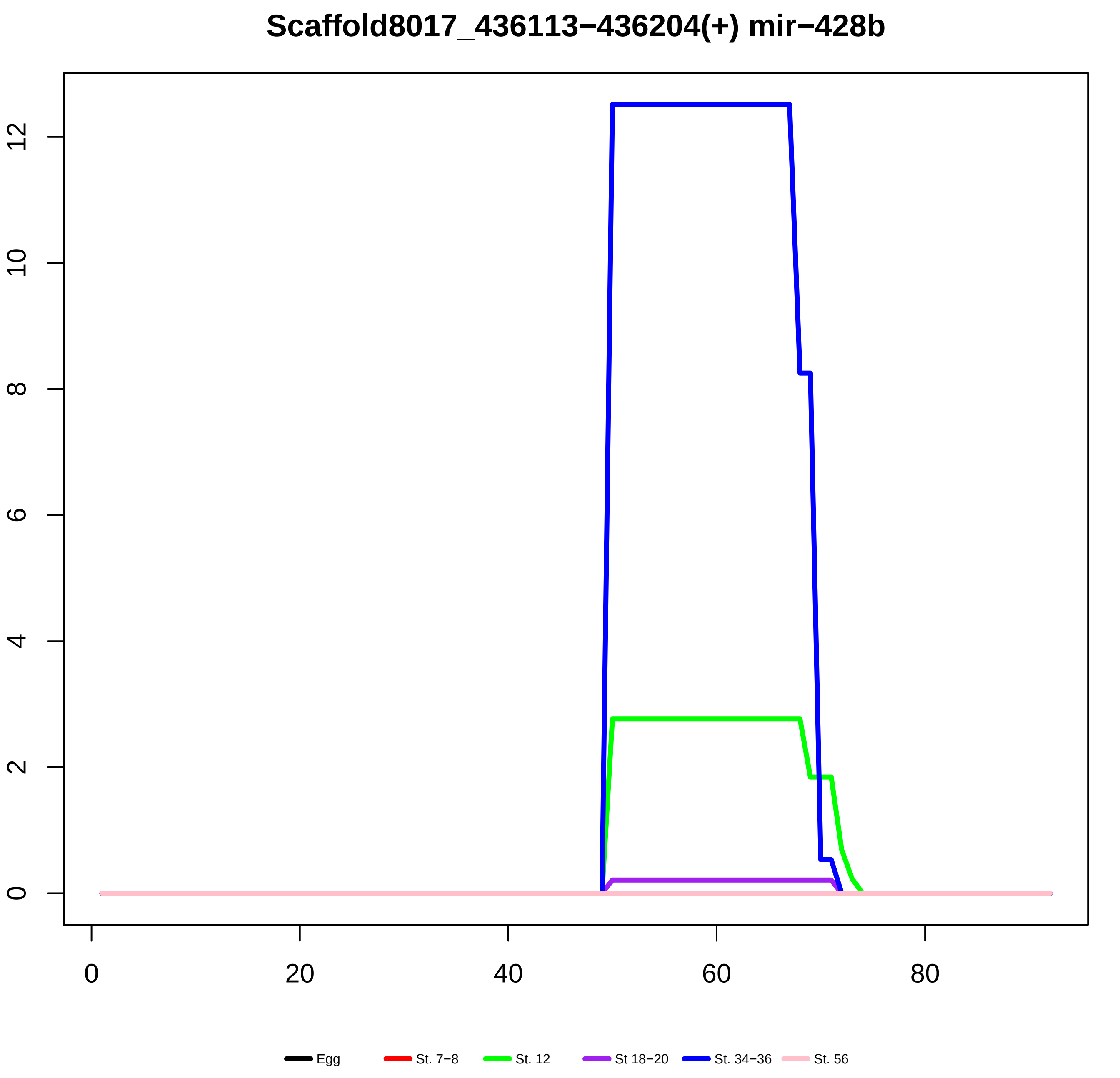

Scaffold8017_436113−436204(+) mir−428b
0
2
4
6
8
10
12
0
20
40
60
80
Egg
St. 7−8
St. 12
St 18−20
St. 34−36
St. 56

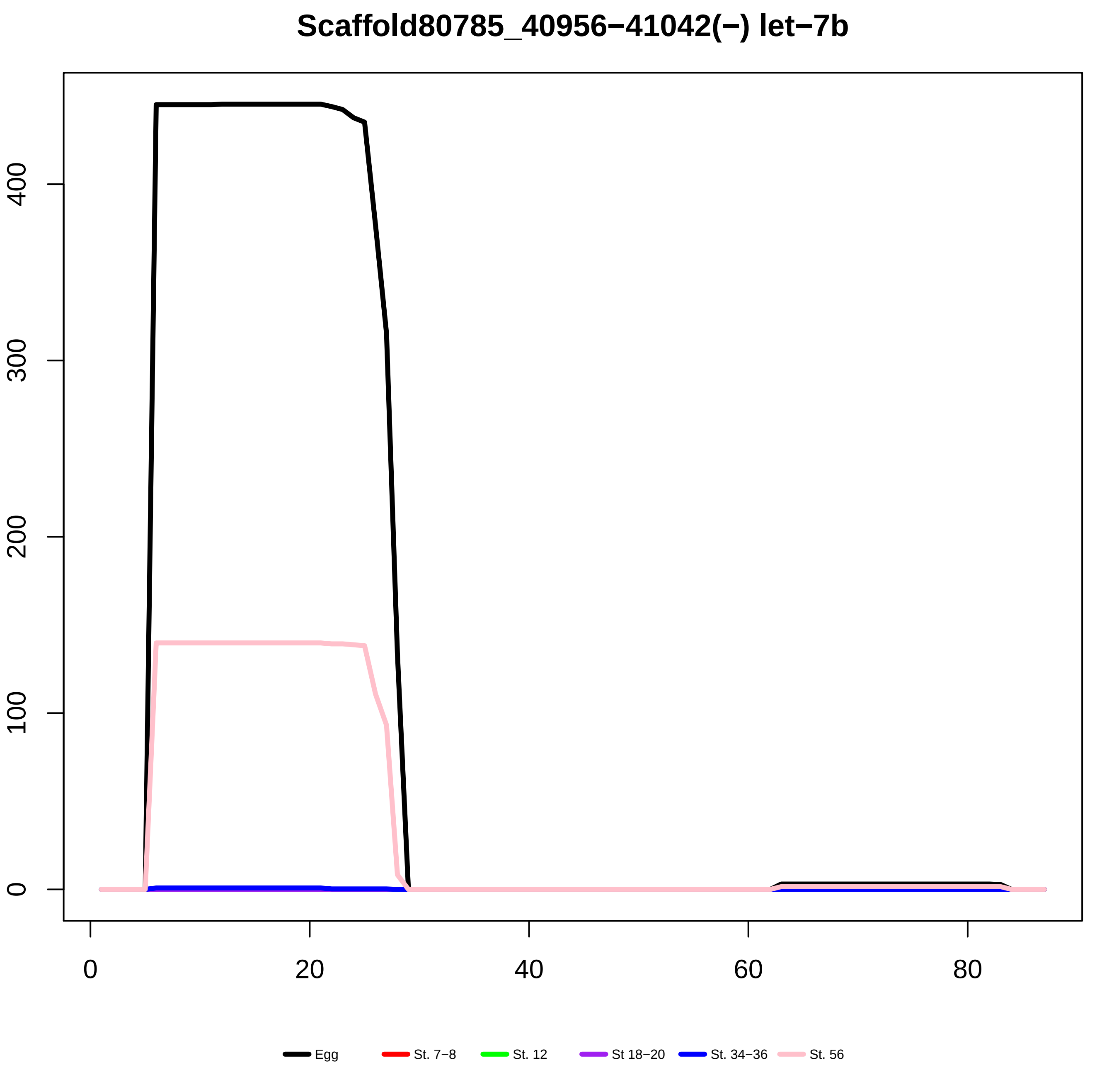

Scaffold80785_40956−41042(−) let−7b
0
100
200
300
400
0
20
40
60
80
Egg
St. 7−8
St. 12
St 18−20
St. 34−36
St. 56

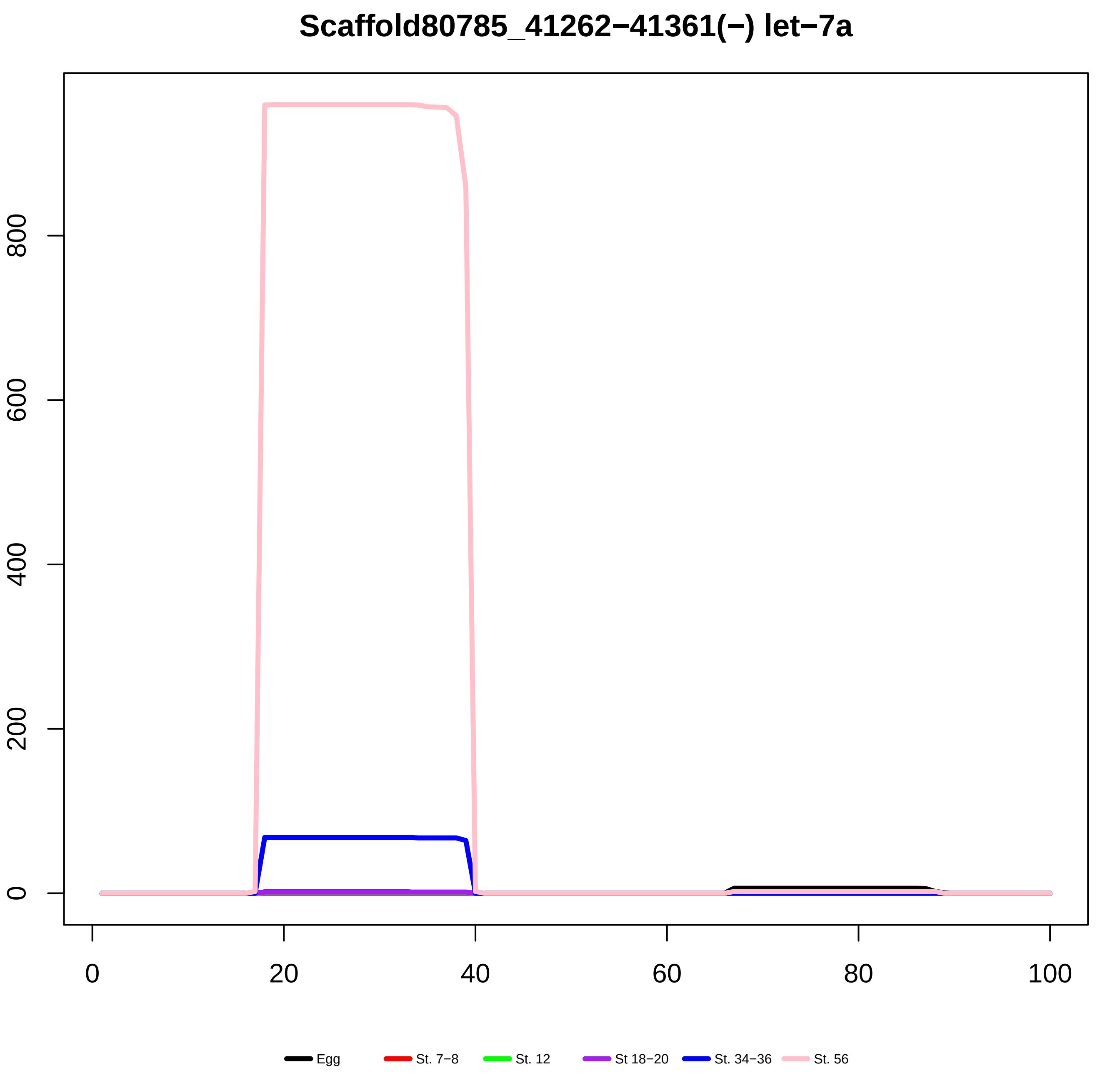
Scaffold80785_41262−41361(−) let−7a
0
200
400
600
800
0
20
40
60
80
100
Egg
St. 7−8
St. 12
St 18−20
St. 34−36
St. 56

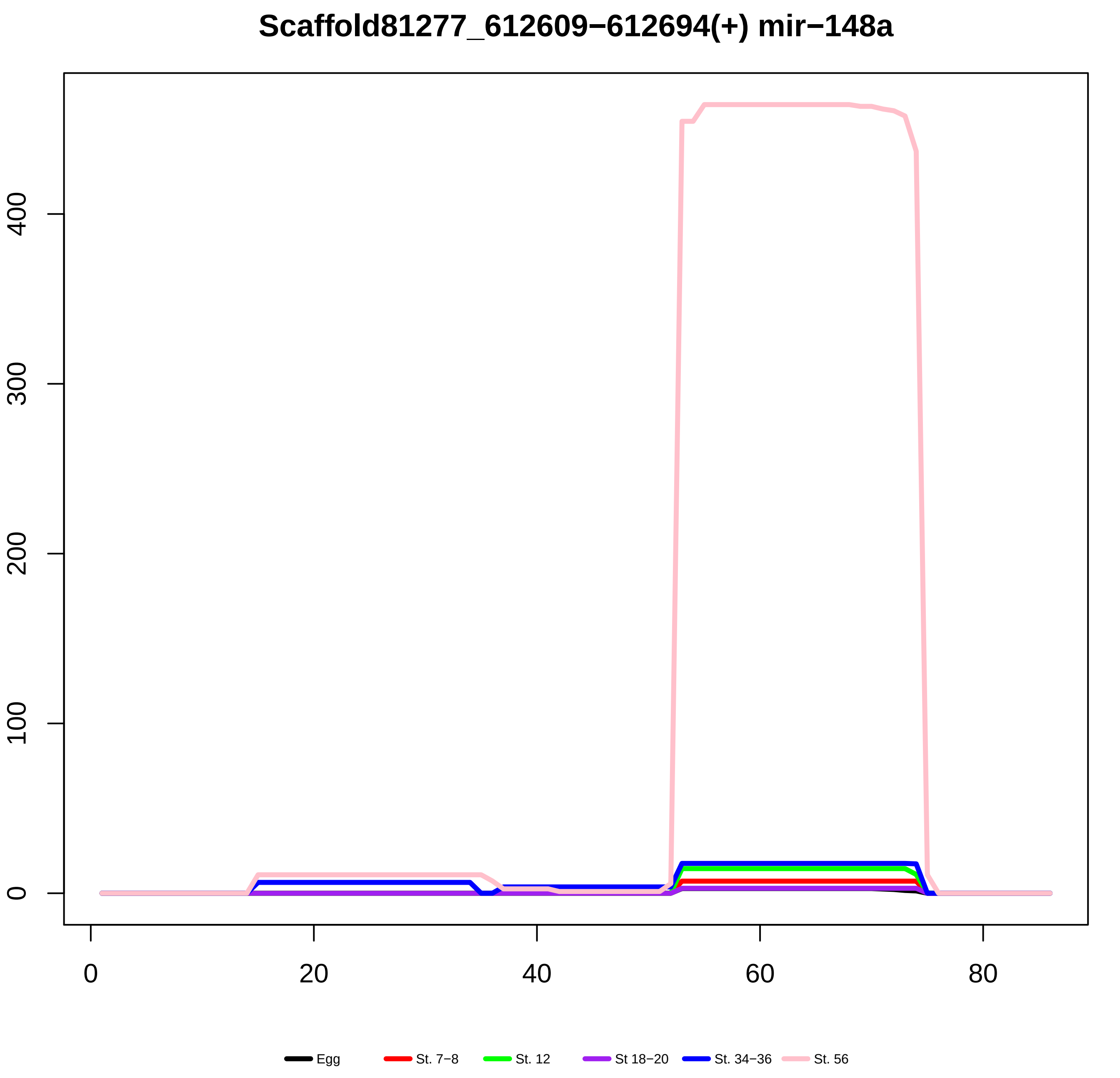
Scaffold81277_612609−612694(+) mir−148a
0
100
200
300
400
0
20
40
60
80
Egg
St. 7−8
St. 12
St 18−20
St. 34−36
St. 56

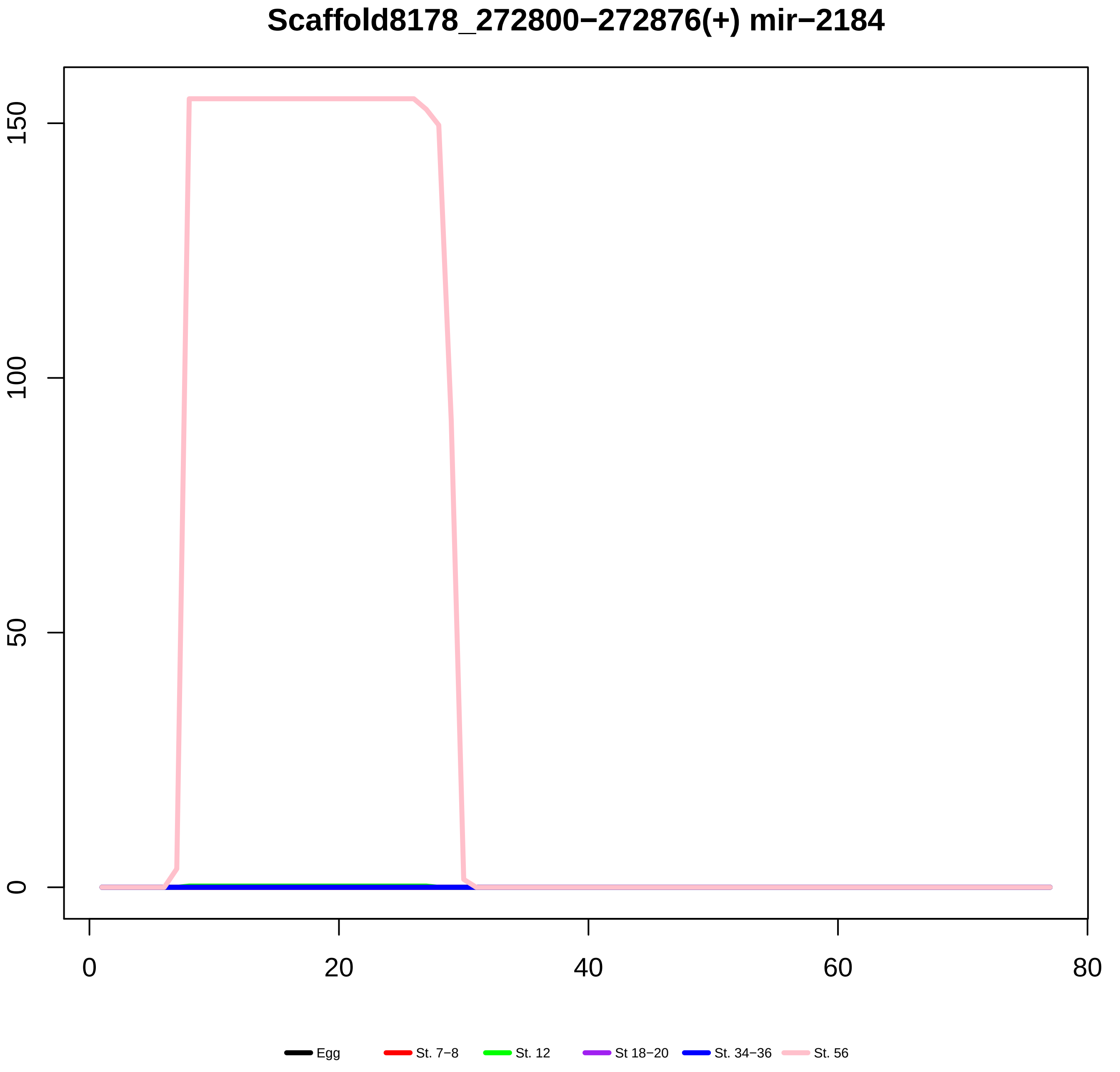

Scaffold8178_272800−272876(+) mir−2184
0
50
100
150
0
20
40
60
80
Egg
St. 7−8
St. 12
St 18−20
St. 34−36
St. 56

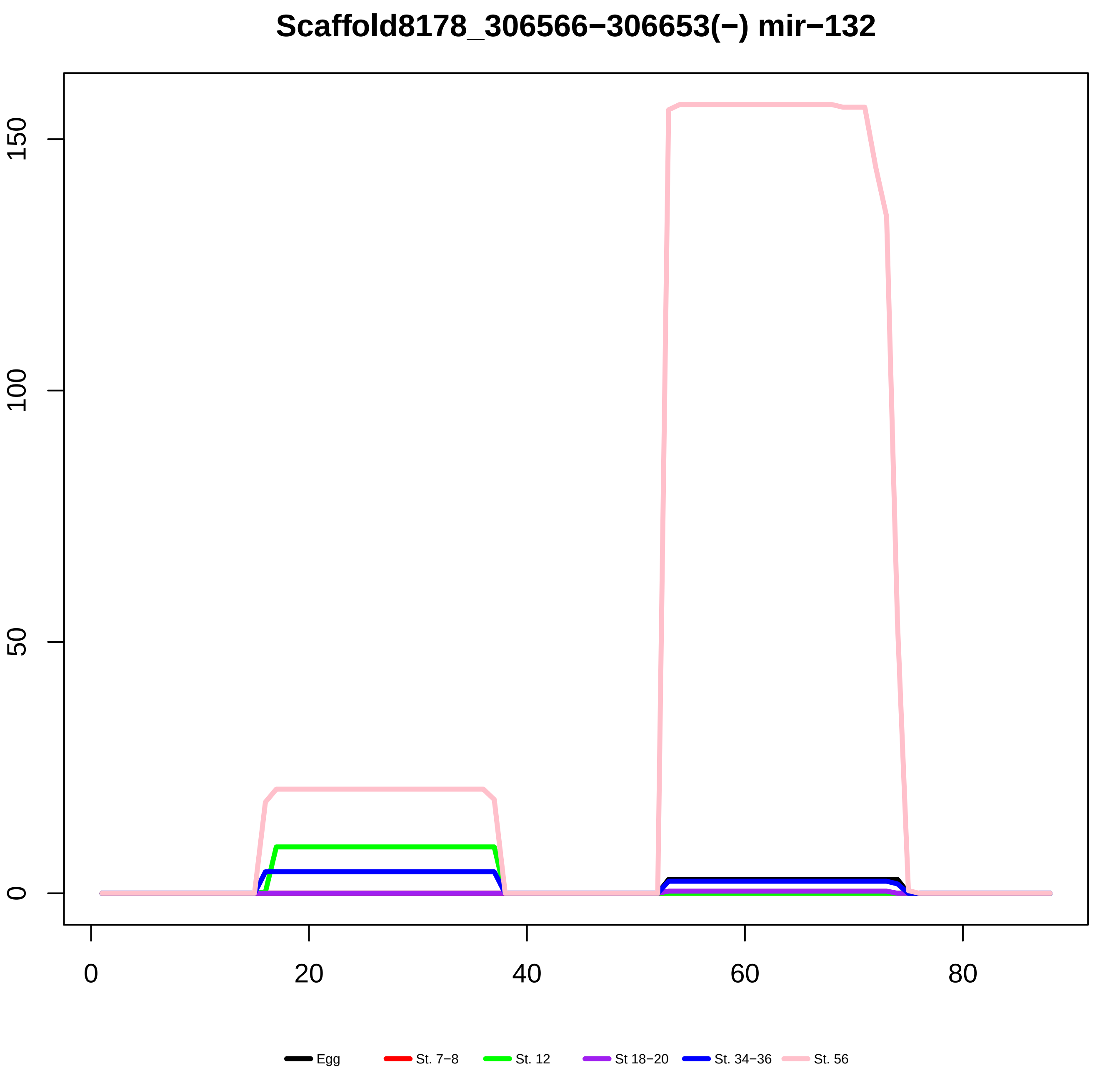

Scaffold8178_306566−306653(−) mir−132
0
50
100
150
0
20
40
60
80
Egg
St. 7−8
St. 12
St 18−20
St. 34−36
St. 56

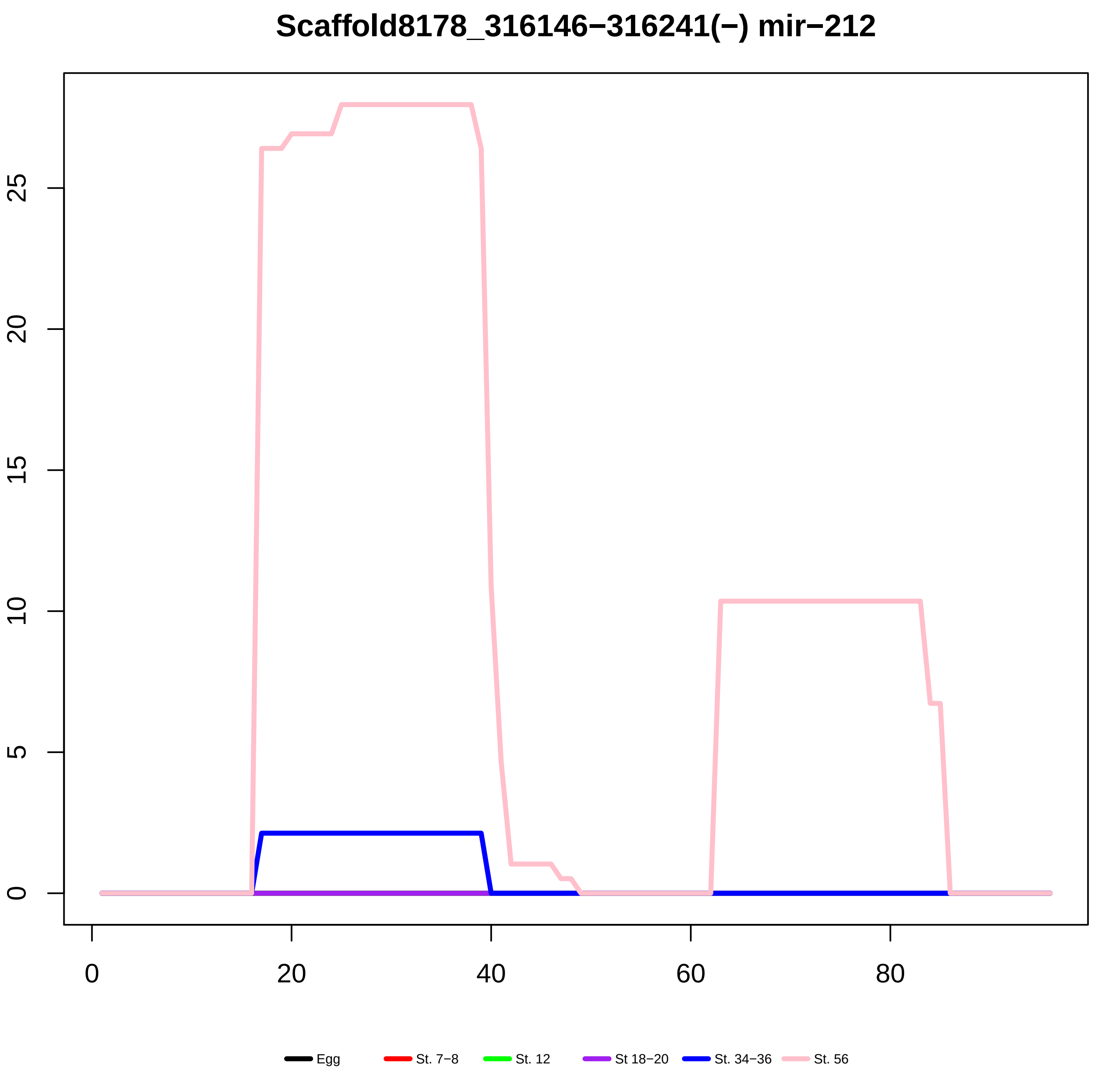

Scaffold8178_316146−316241(−) mir−212
0
5
10
15
20
25
0
20
40
60
80
Egg
St. 7−8
St. 12
St 18−20
St. 34−36
St. 56

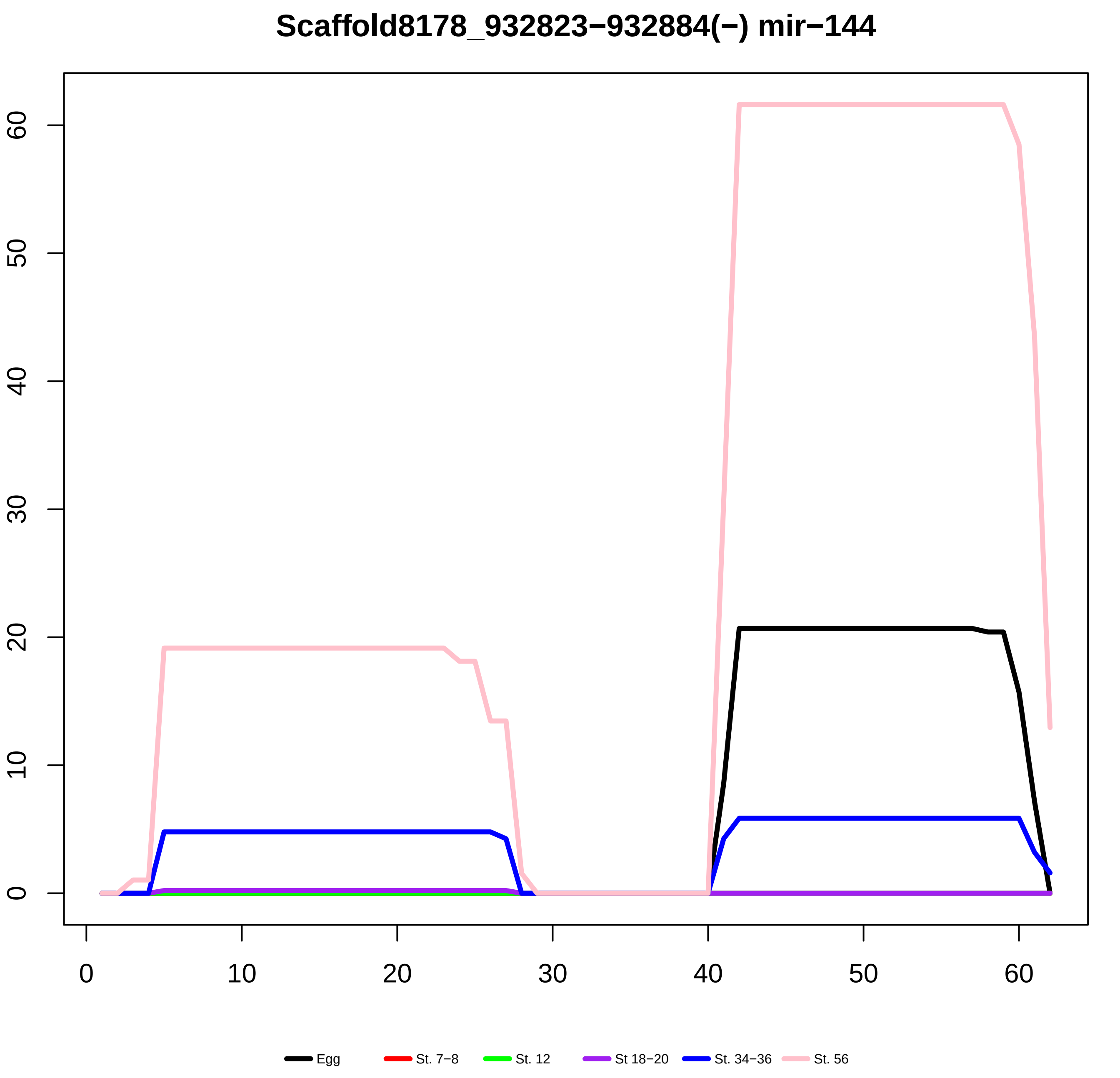

Scaffold8178_932823−932884(−) mir−144
0
10
20
30
40
50
60
0
10
20
30
40
50
60
Egg
St. 7−8
St. 12
St 18−20
St. 34−36
St. 56

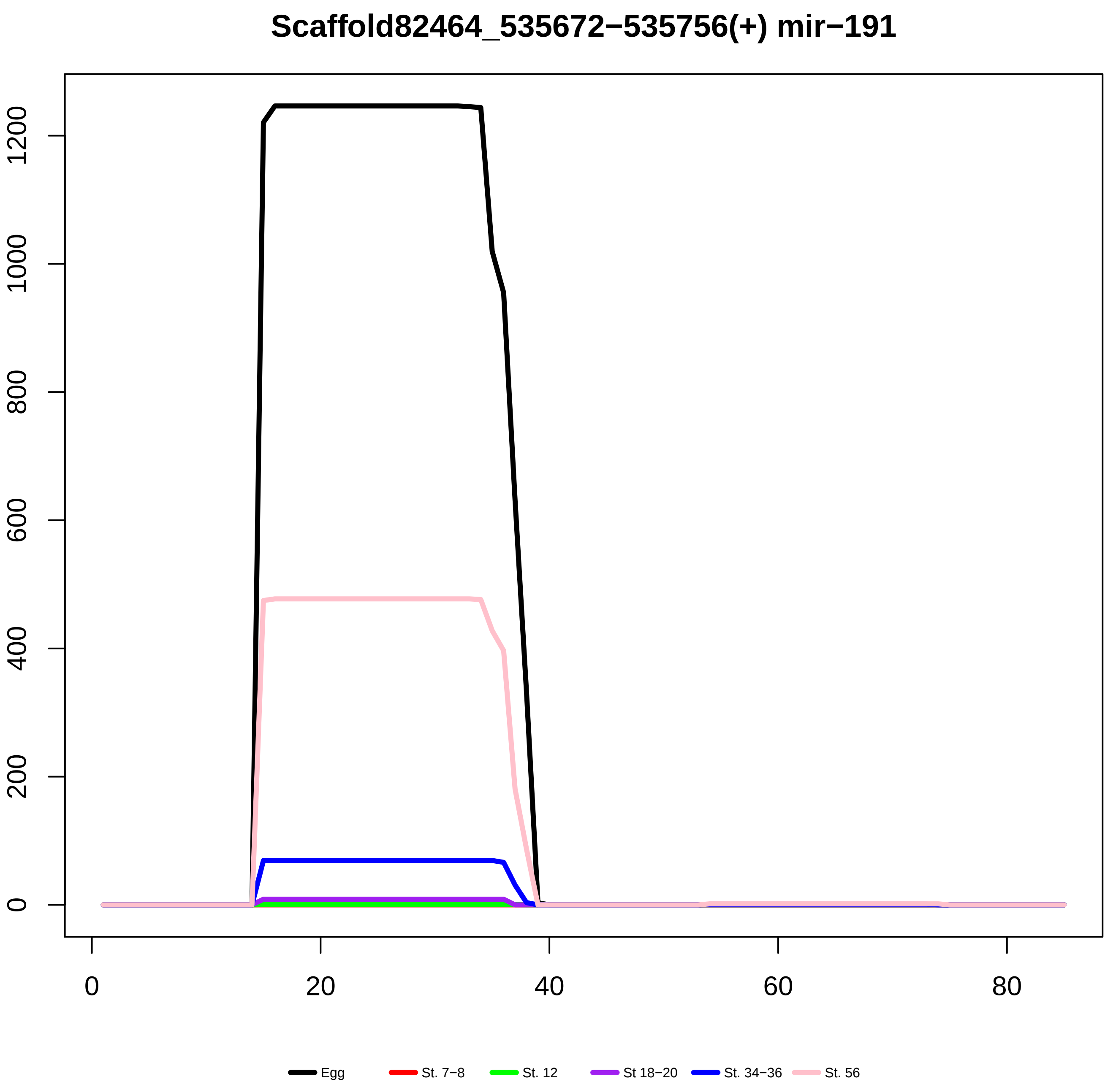

Scaffold82464_535672−535756(+) mir−191
0
200
400
600
800
1000
1200
0
20
40
60
80
Egg
St. 7−8
St. 12
St 18−20
St. 34−36
St. 56

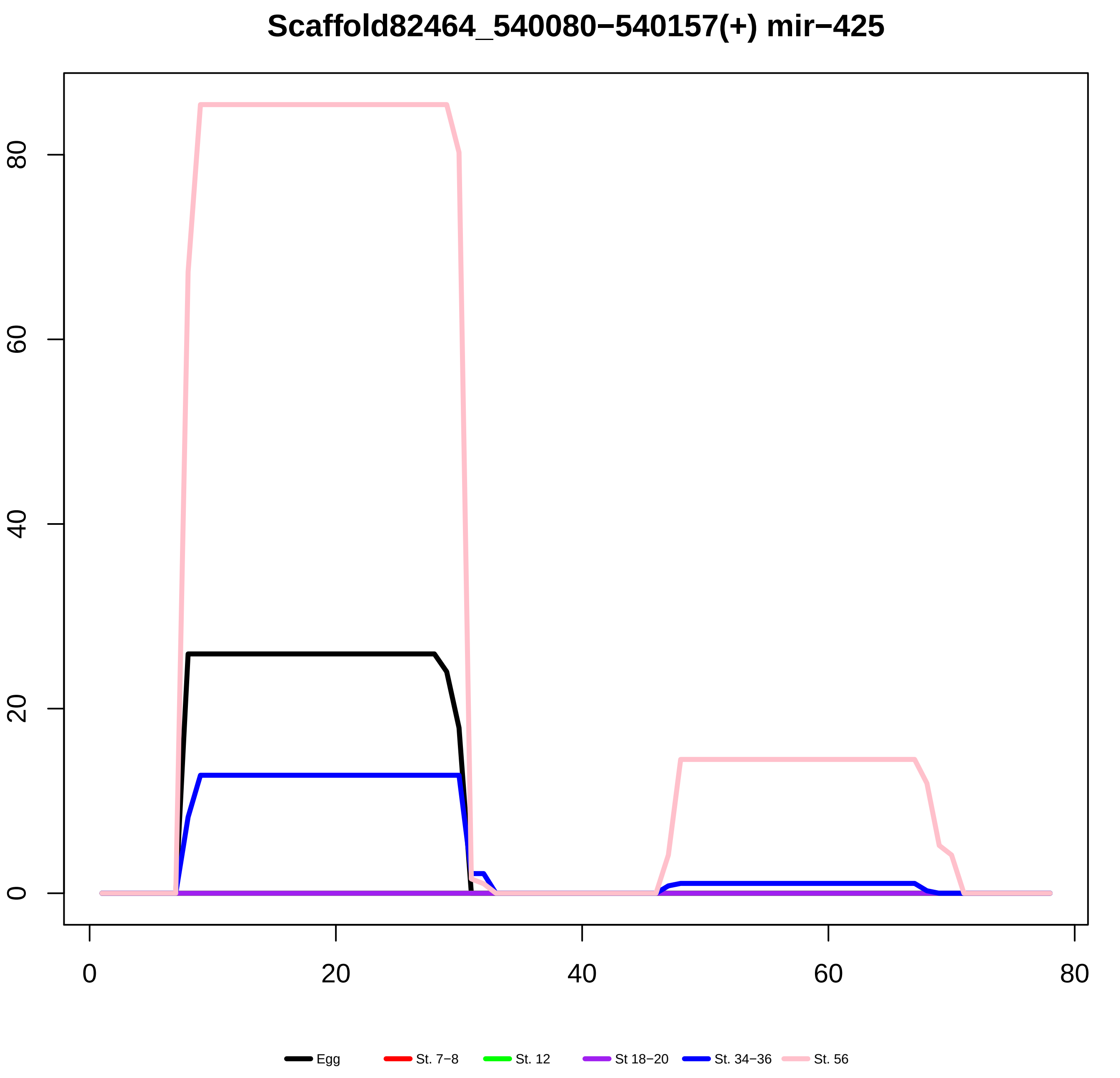
Scaffold82464_540080−540157(+) mir−425
0
20
40
60
80
0
20
40
60
80
Egg
St. 7−8
St. 12
St 18−20
St. 34−36
St. 56

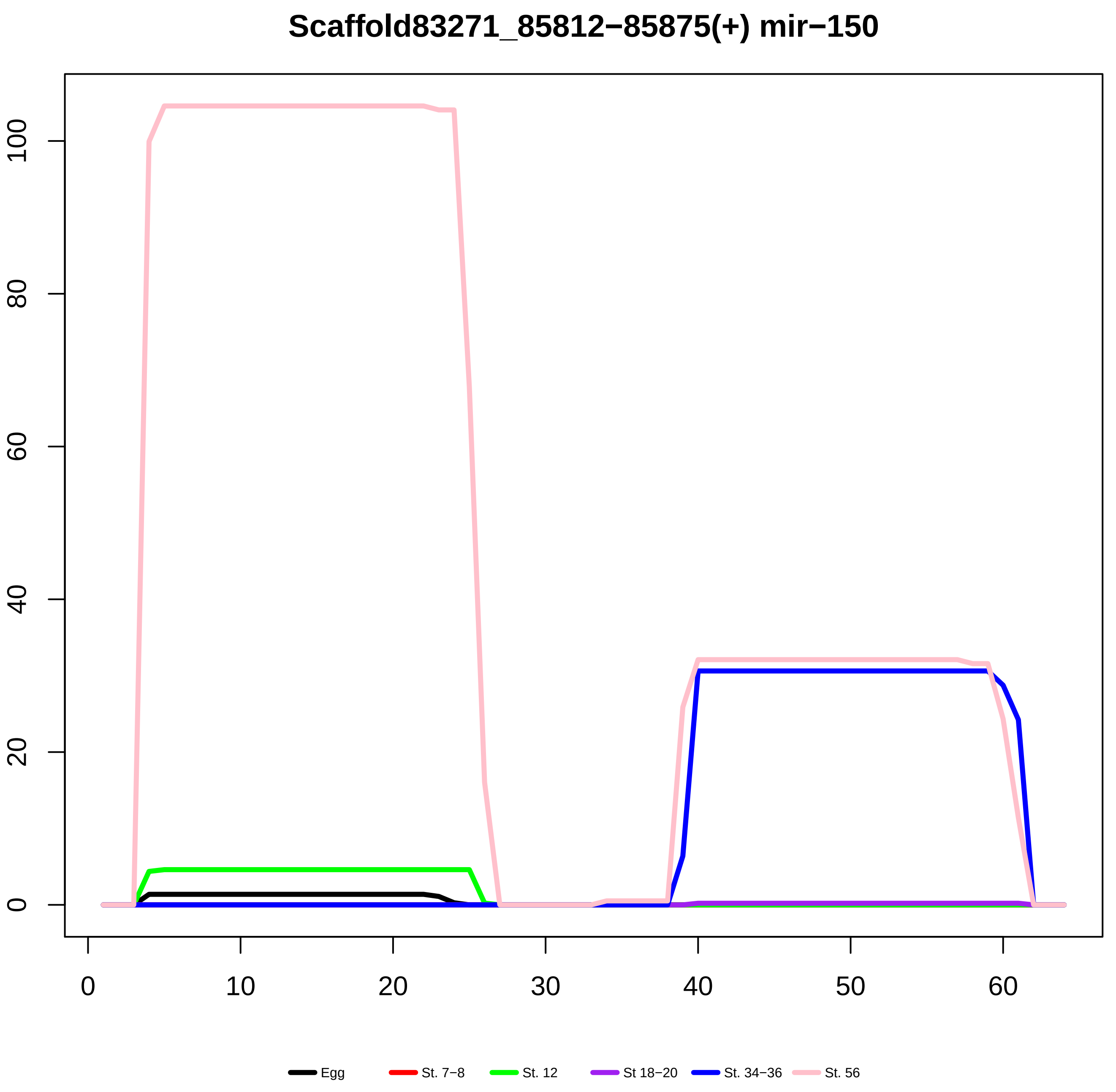

Scaffold83271_85812−85875(+) mir−150
0
20
40
60
80
100
0
10
20
30
40
50
60
Egg
St. 7−8
St. 12
St 18−20
St. 34−36
St. 56

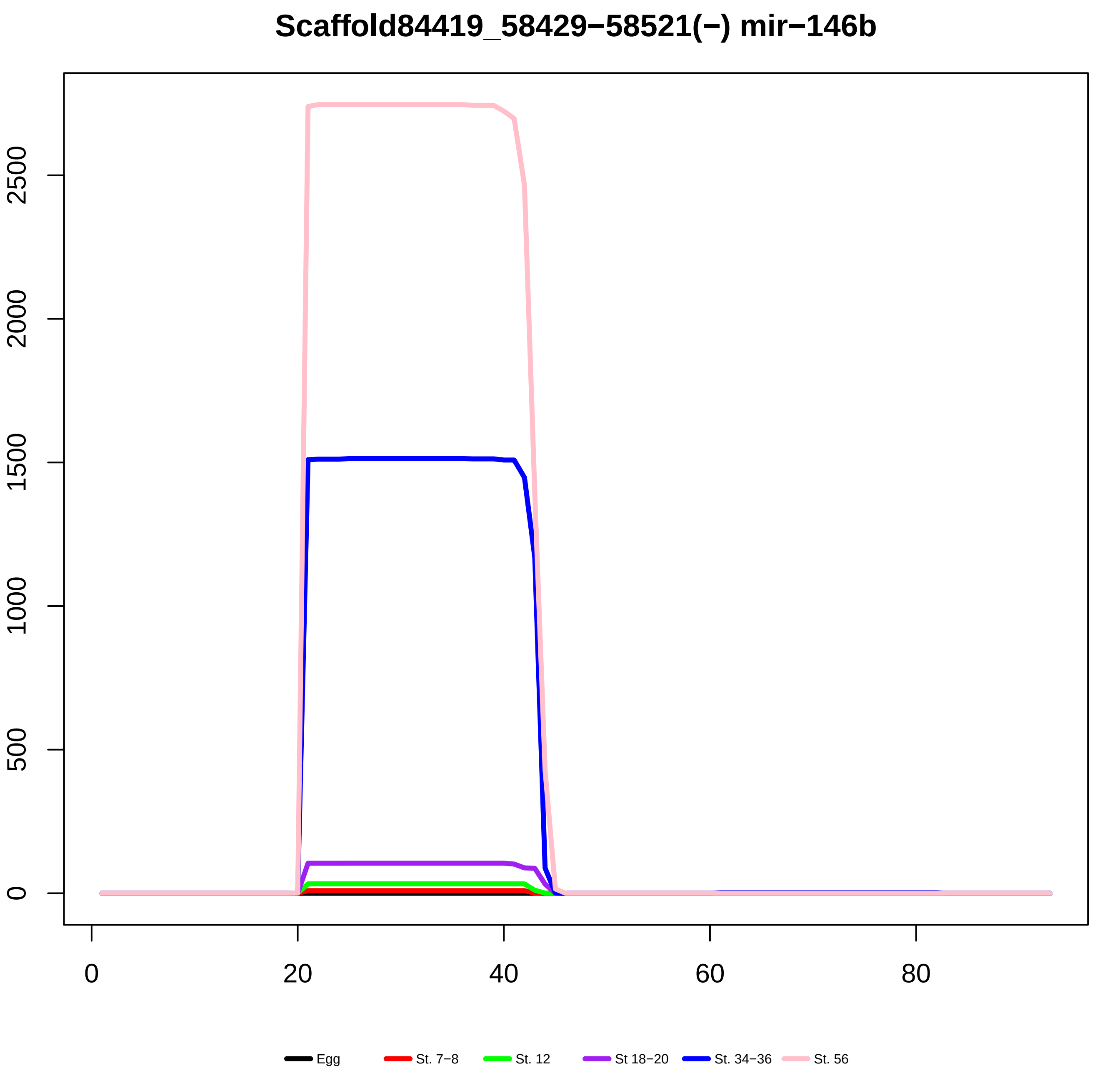
Scaffold84419_58429−58521(−) mir−146b
0
500
1000
1500
2000
2500
0
20
40
60
80
Egg
St. 7−8
St. 12
St 18−20
St. 34−36
St. 56

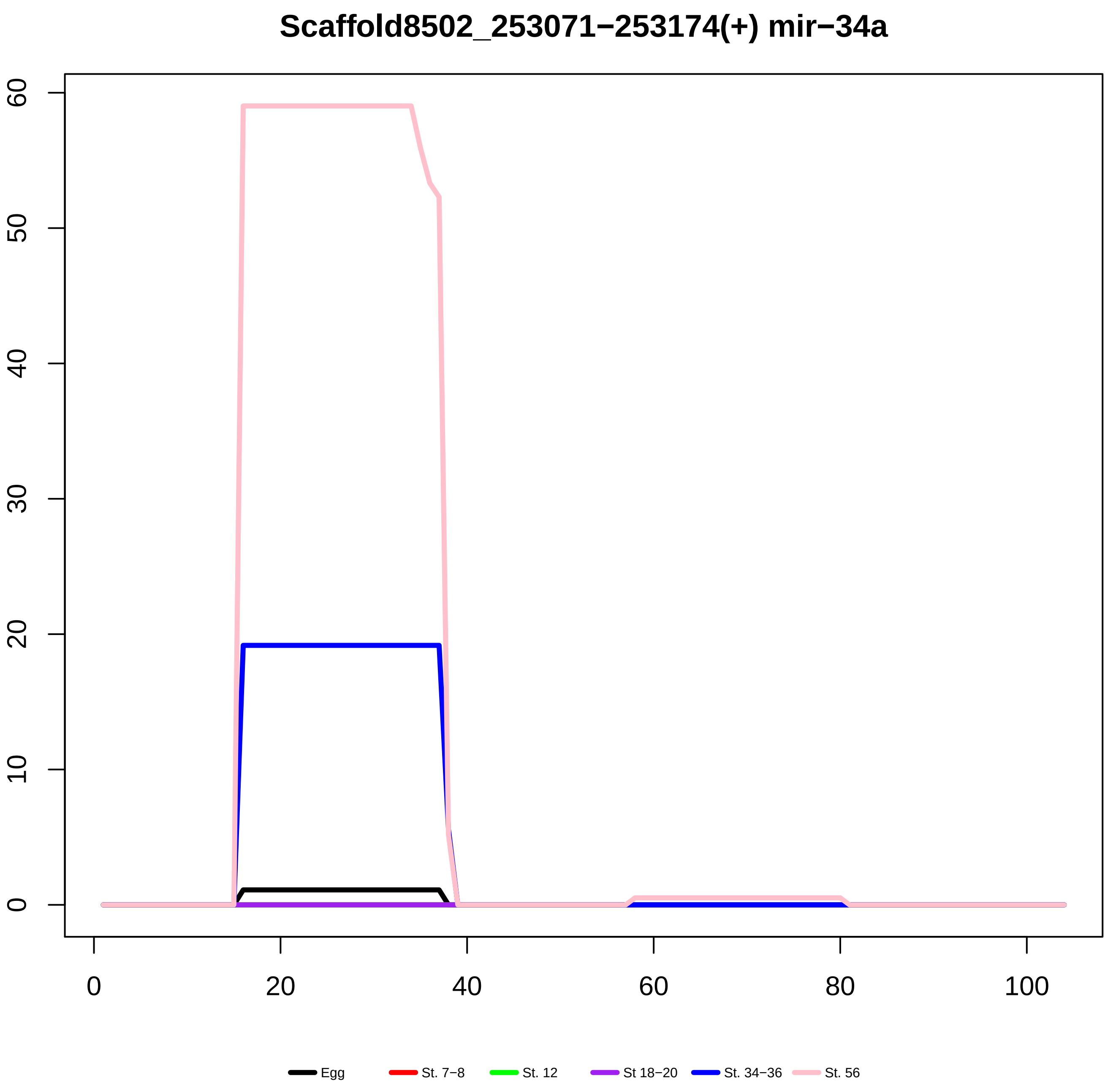

Scaffold8502_253071−253174(+) mir−34a
0
10
20
30
40
50
60
0
20
40
60
80
100
Egg
St. 7−8
St. 12
St 18−20
St. 34−36
St. 56

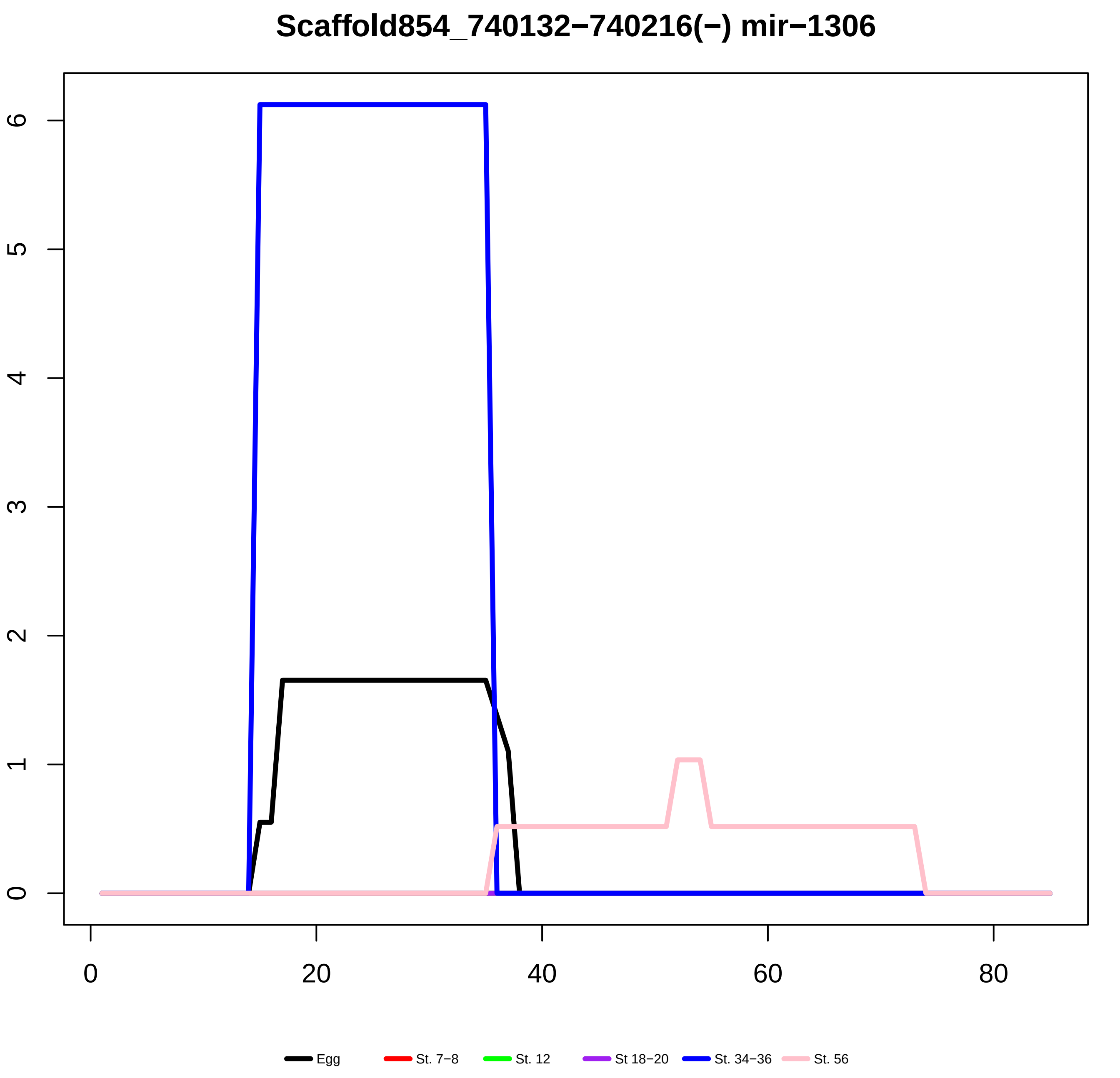

Scaffold854_740132−740216(−) mir−1306
0
1
2
3
4
5
6
0
20
40
60
80
Egg
St. 7−8
St. 12
St 18−20
St. 34−36
St. 56

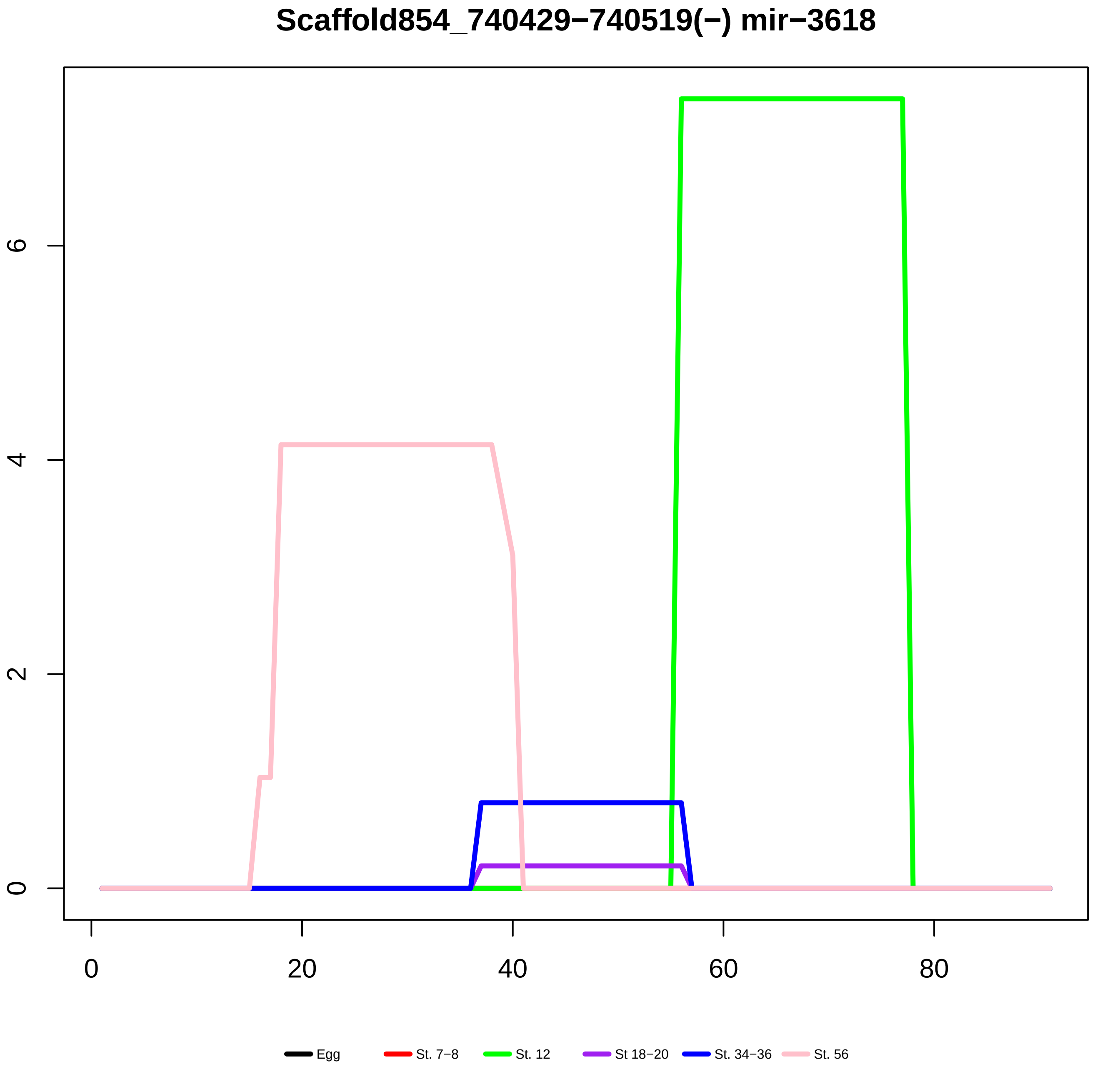
Scaffold854_740429−740519(−) mir−3618
0
2
4
6
0
20
40
60
80
Egg
St. 7−8
St. 12
St 18−20
St. 34−36
St. 56

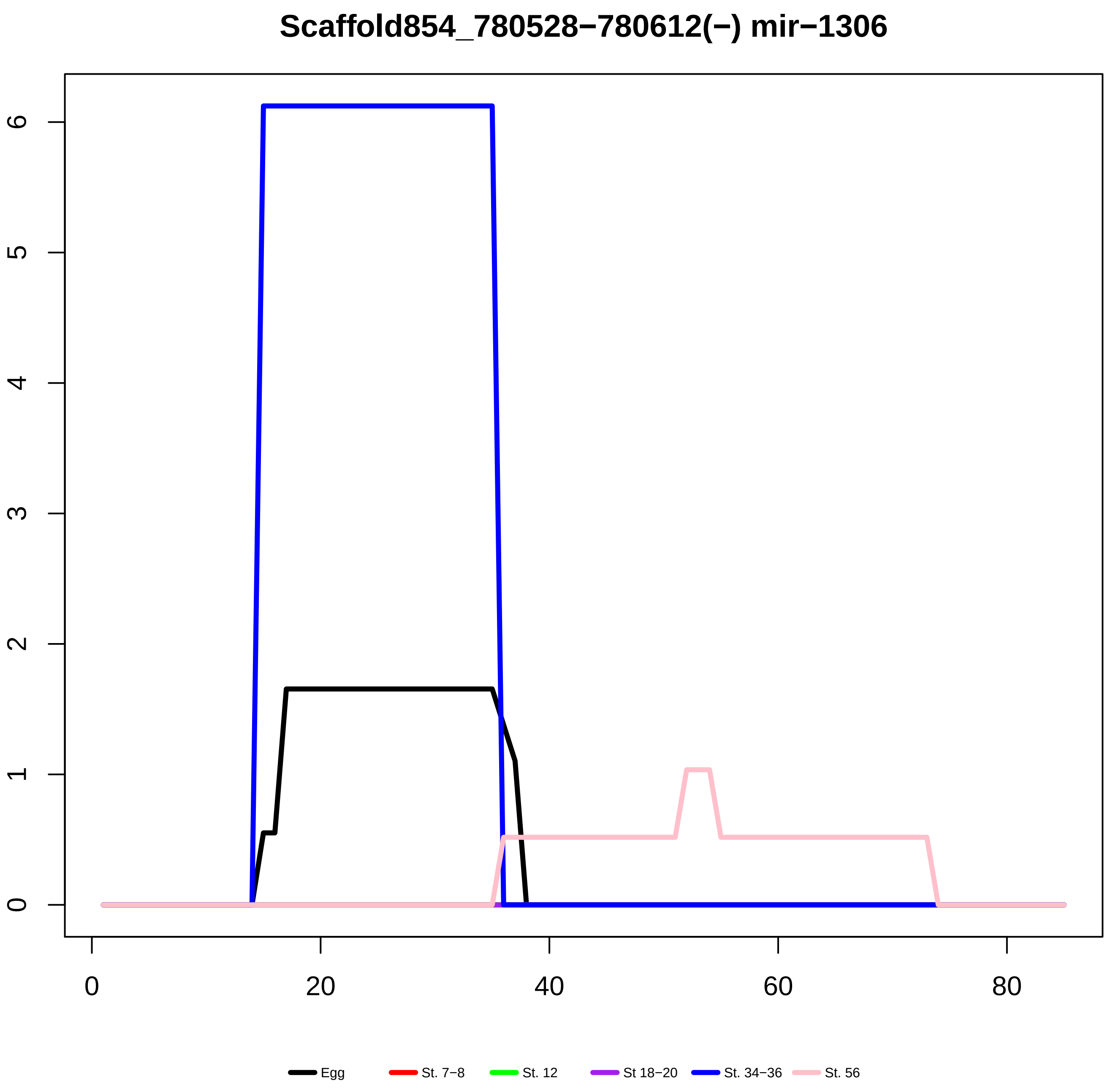
Scaffold854_780528−780612(−) mir−1306
0
1
2
3
4
5
6
0
20
40
60
80
Egg
St. 7−8
St. 12
St 18−20
St. 34−36
St. 56

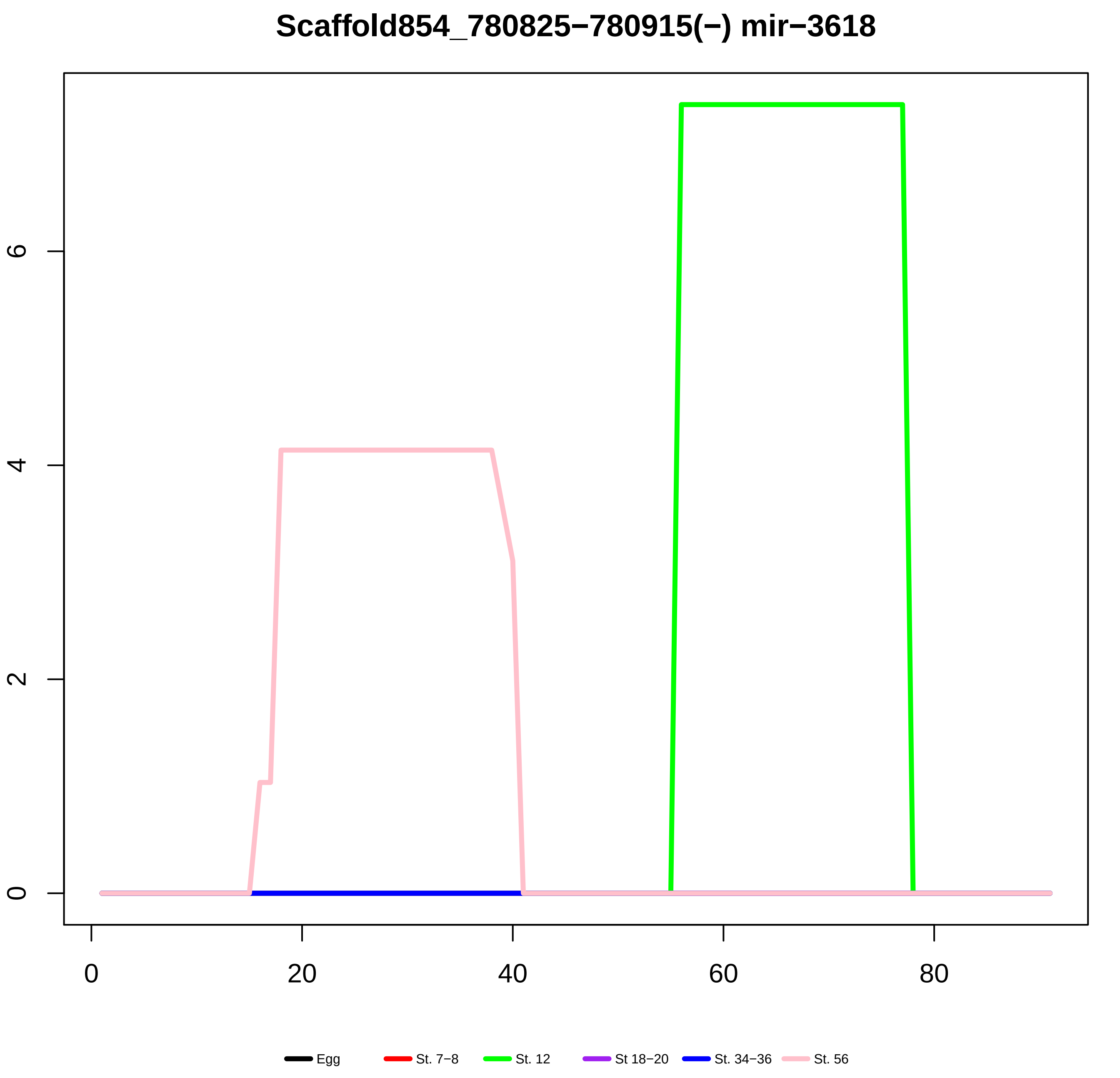

Scaffold854_780825−780915(−) mir−3618
0
2
4
6
0
20
40
60
80
Egg
St. 7−8
St. 12
St 18−20
St. 34−36
St. 56

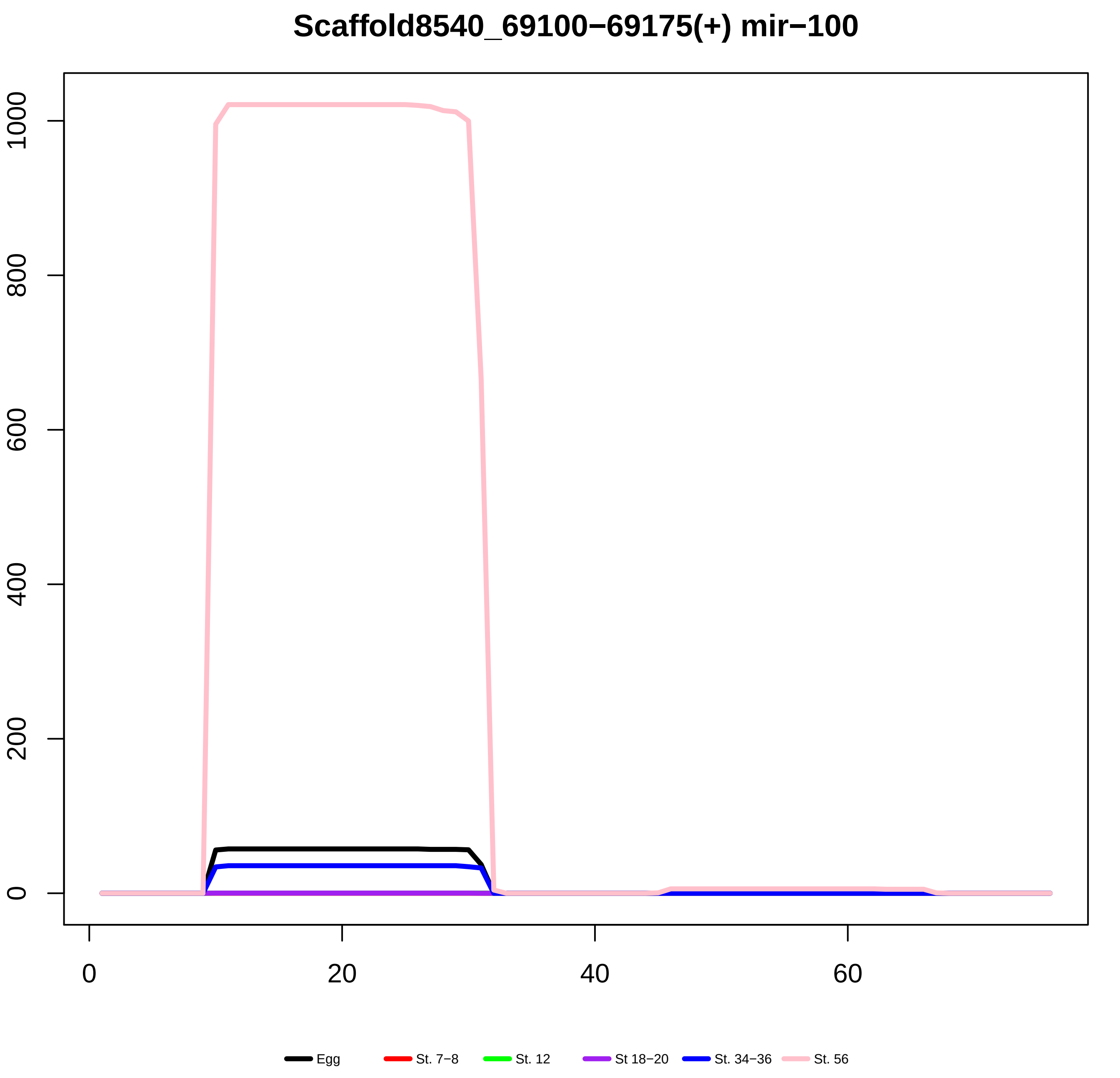

Scaffold8540_69100−69175(+) mir−100
0
200
400
600
800
1000
0
20
40
60
Egg
St. 7−8
St. 12
St 18−20
St. 34−36
St. 56

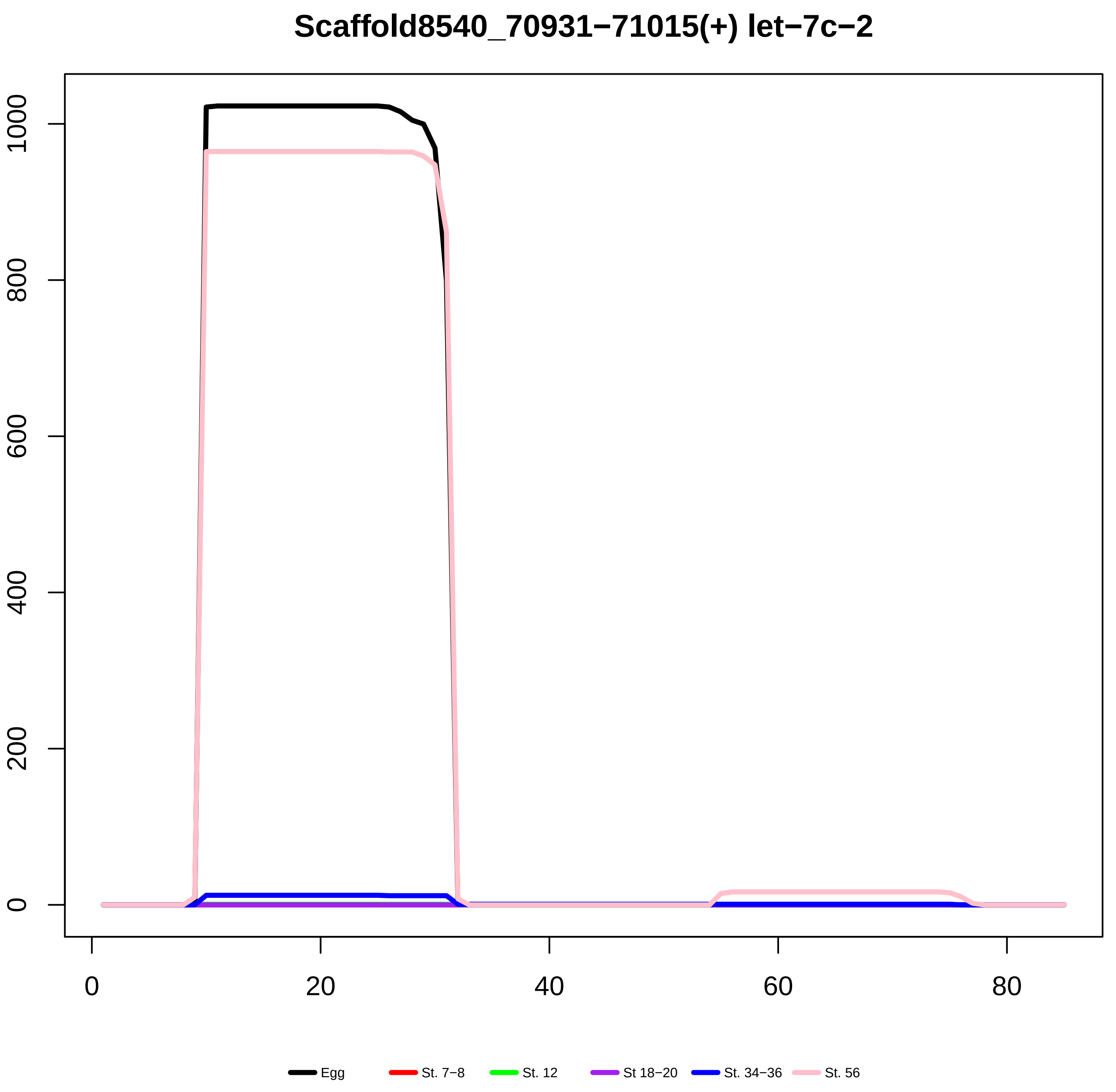
Scaffold8540_70931−71015(+) let−7c−2
0
200
400
600
800
1000
0
20
40
60
80
Egg
St. 7−8
St. 12
St 18−20
St. 34−36
St. 56

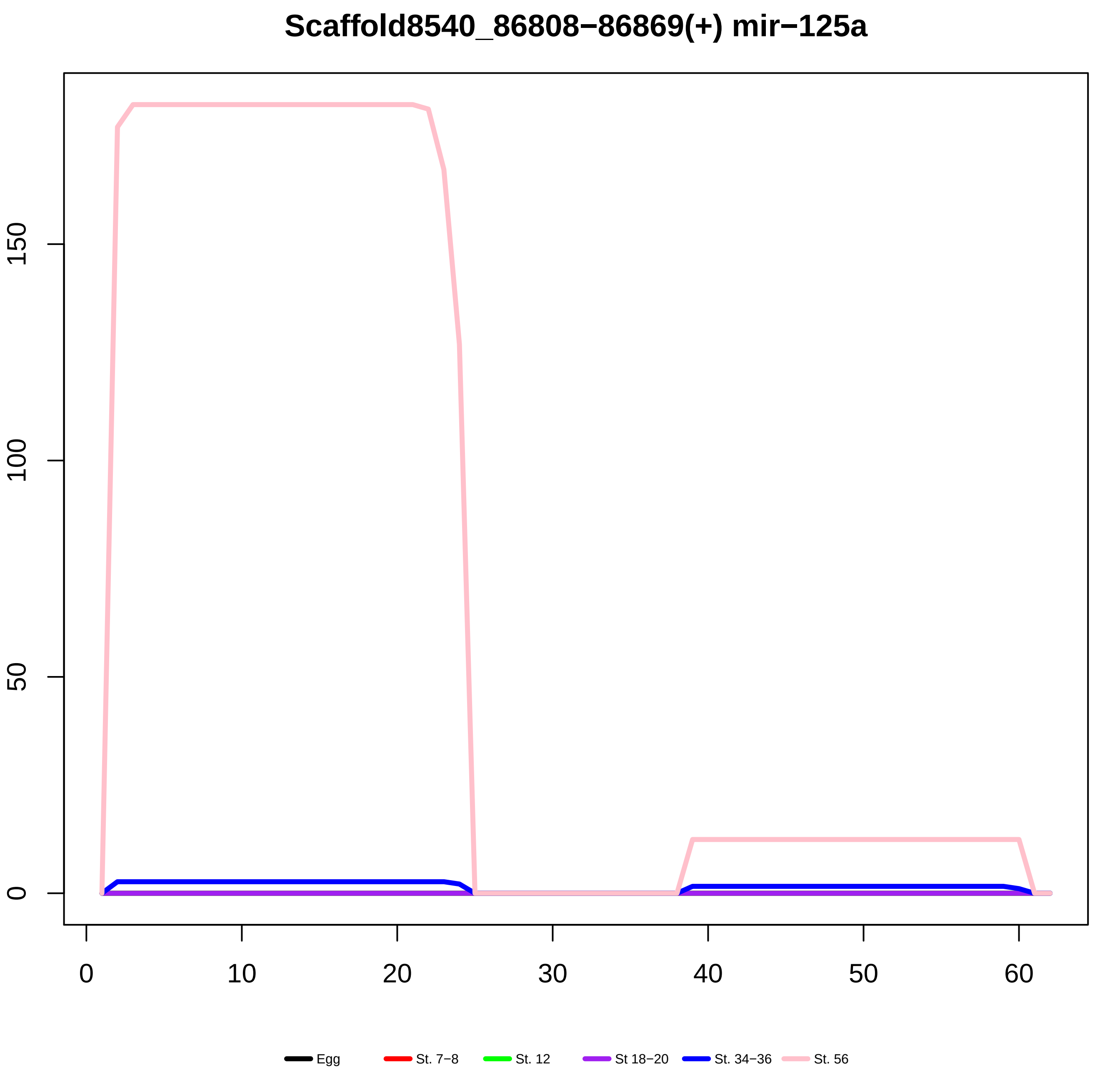

Scaffold8540_86808−86869(+) mir−125a
0
50
100
150
0
10
20
30
40
50
60
Egg
St. 7−8
St. 12
St 18−20
St. 34−36
St. 56

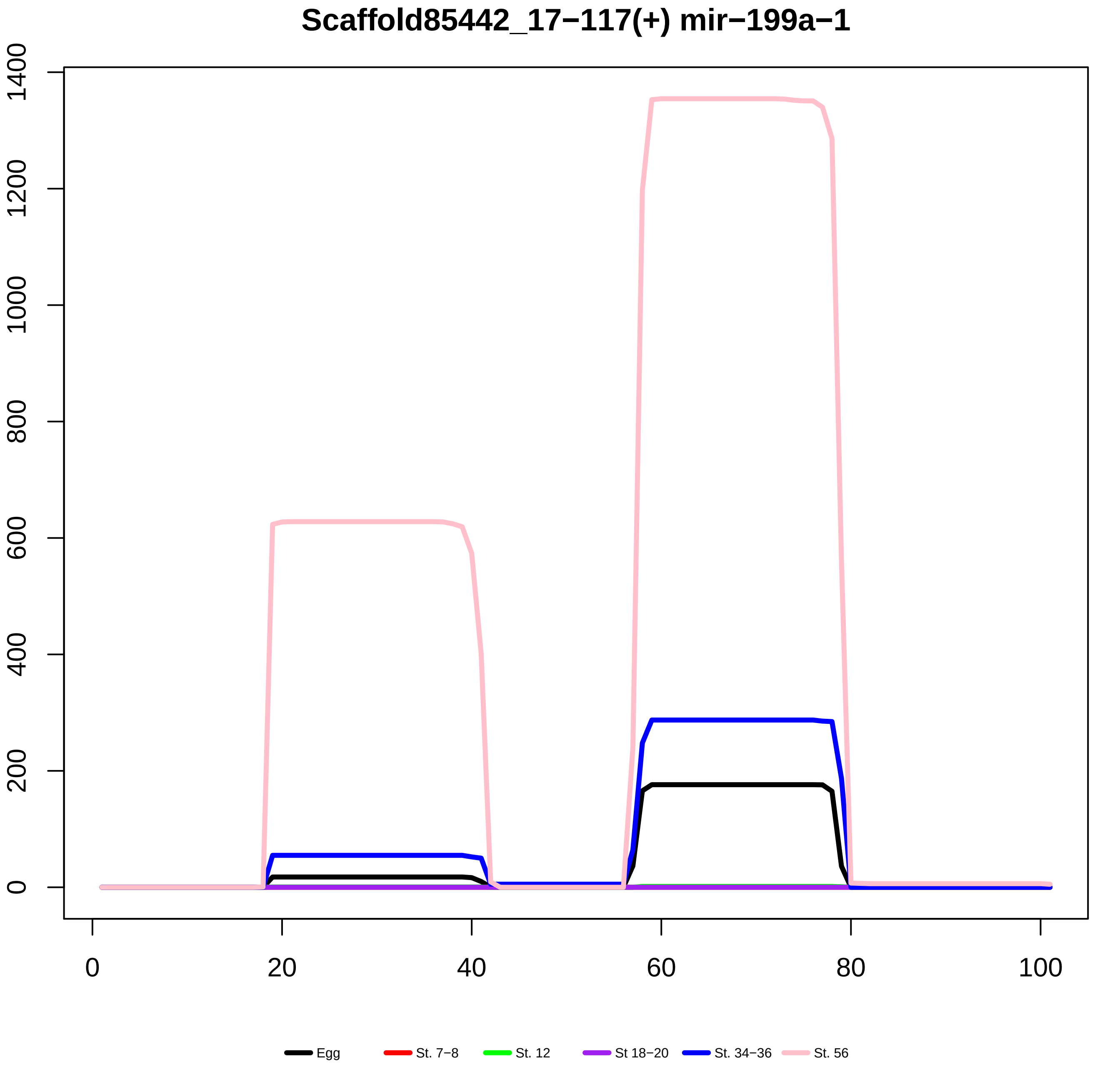

Scaffold85442_17−117(+) mir−199a−1
0
200
400
600
800
1000
1200
1400
0
20
40
60
80
100
Egg
St. 7−8
St. 12
St 18−20
St. 34−36
St. 56

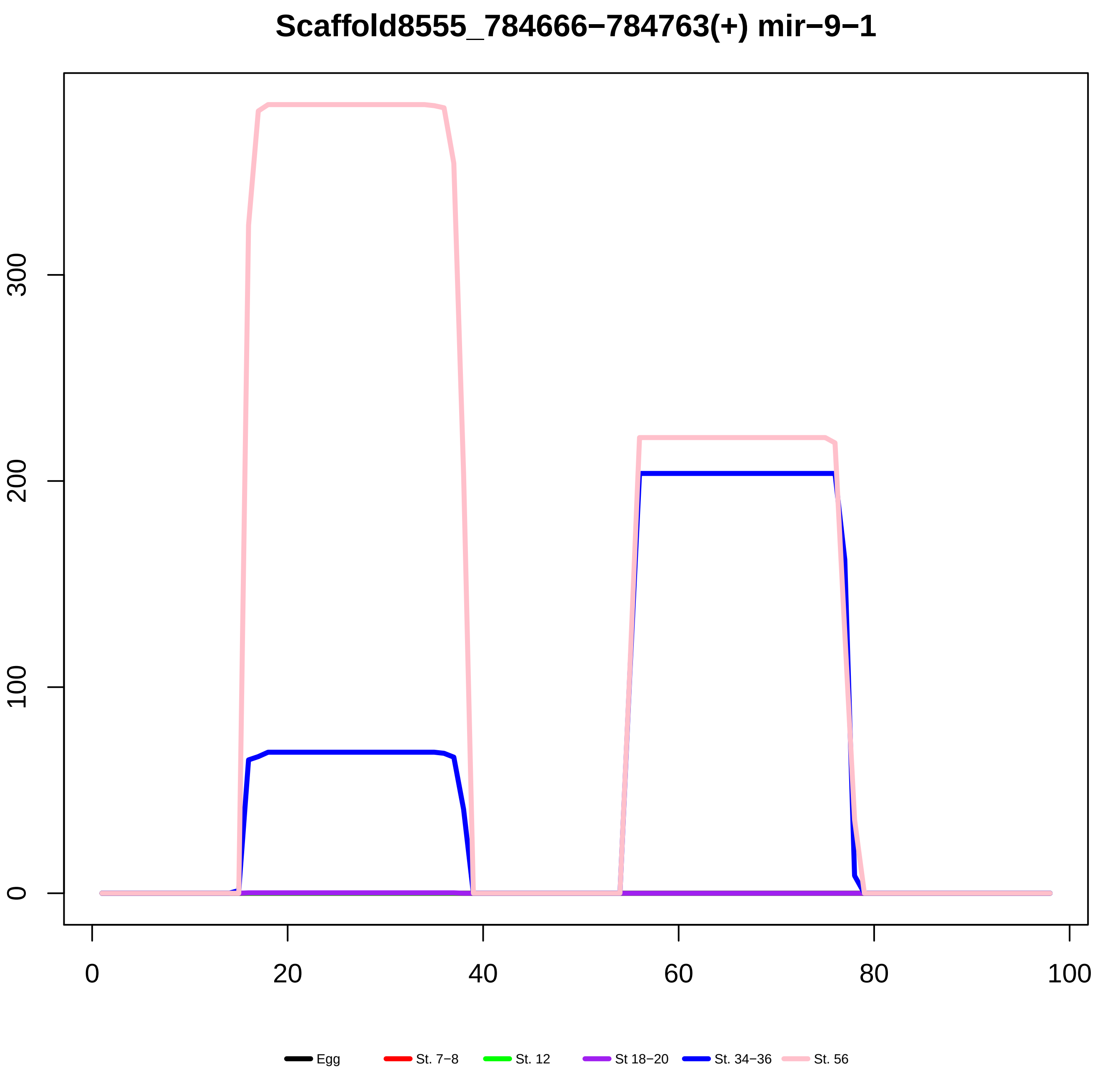

Scaffold8555_784666−784763(+) mir−9−1
0
100
200
300
0
20
40
60
80
100
Egg
St. 7−8
St. 12
St 18−20
St. 34−36
St. 56

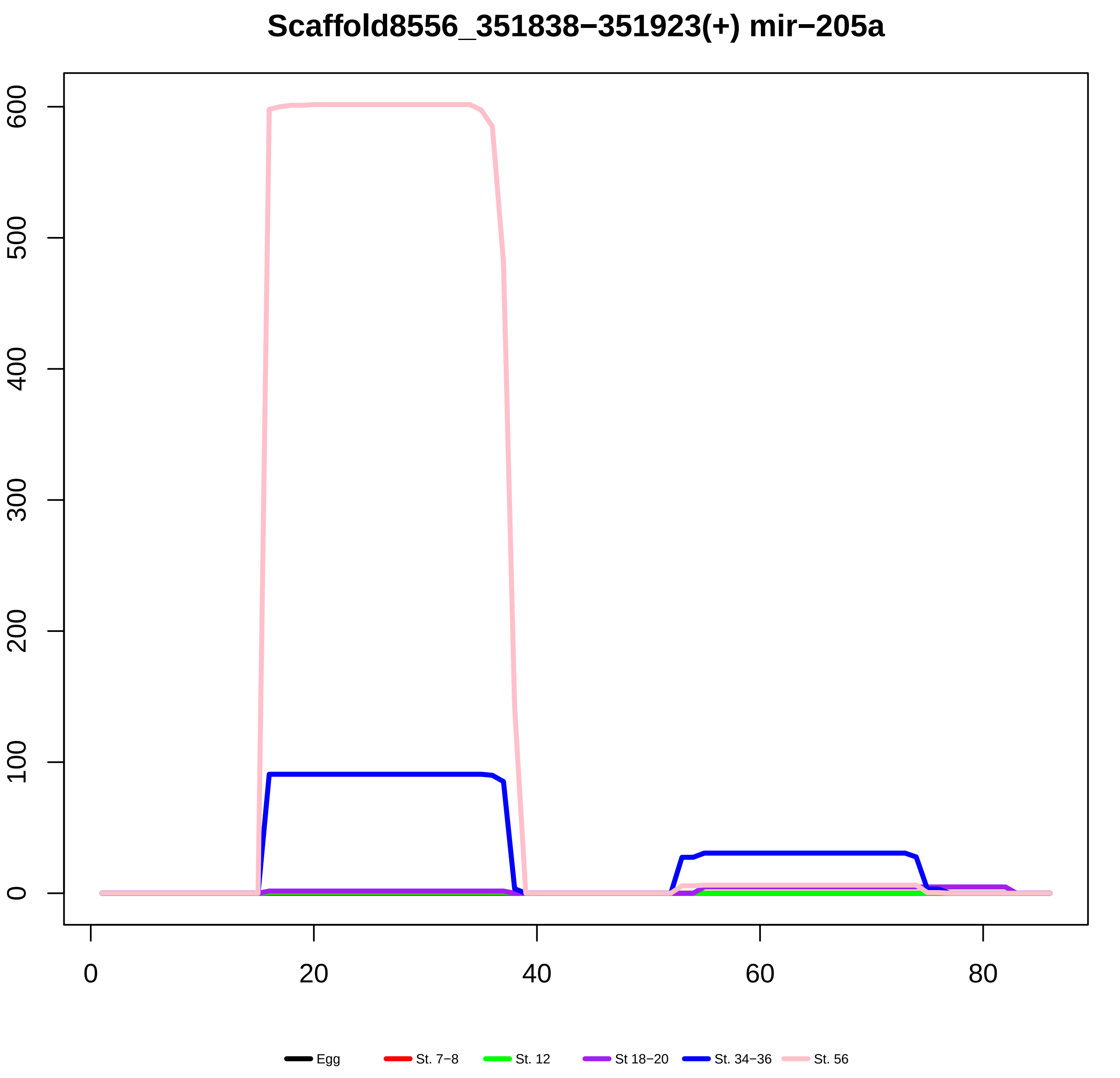
Scaffold8556_351838−351923(+) mir−205a
0
100
200
300
400
500
600
0
20
40
60
80
Egg
St. 7−8
St. 12
St 18−20
St. 34−36
St. 56

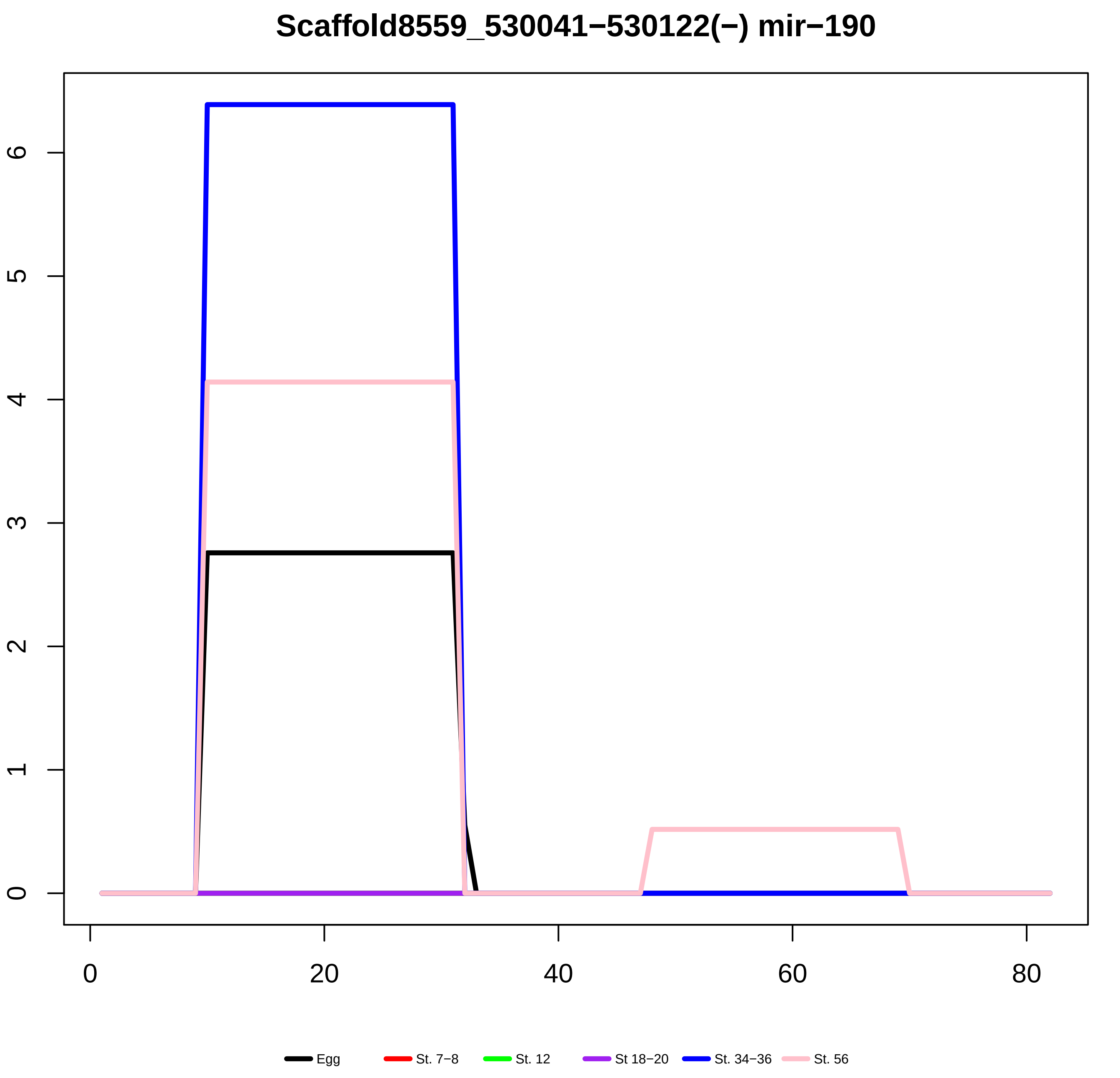
Scaffold8559_530041−530122(−) mir−190
0
1
2
3
4
5
6
0
20
40
60
80
Egg
St. 7−8
St. 12
St 18−20
St. 34−36
St. 56

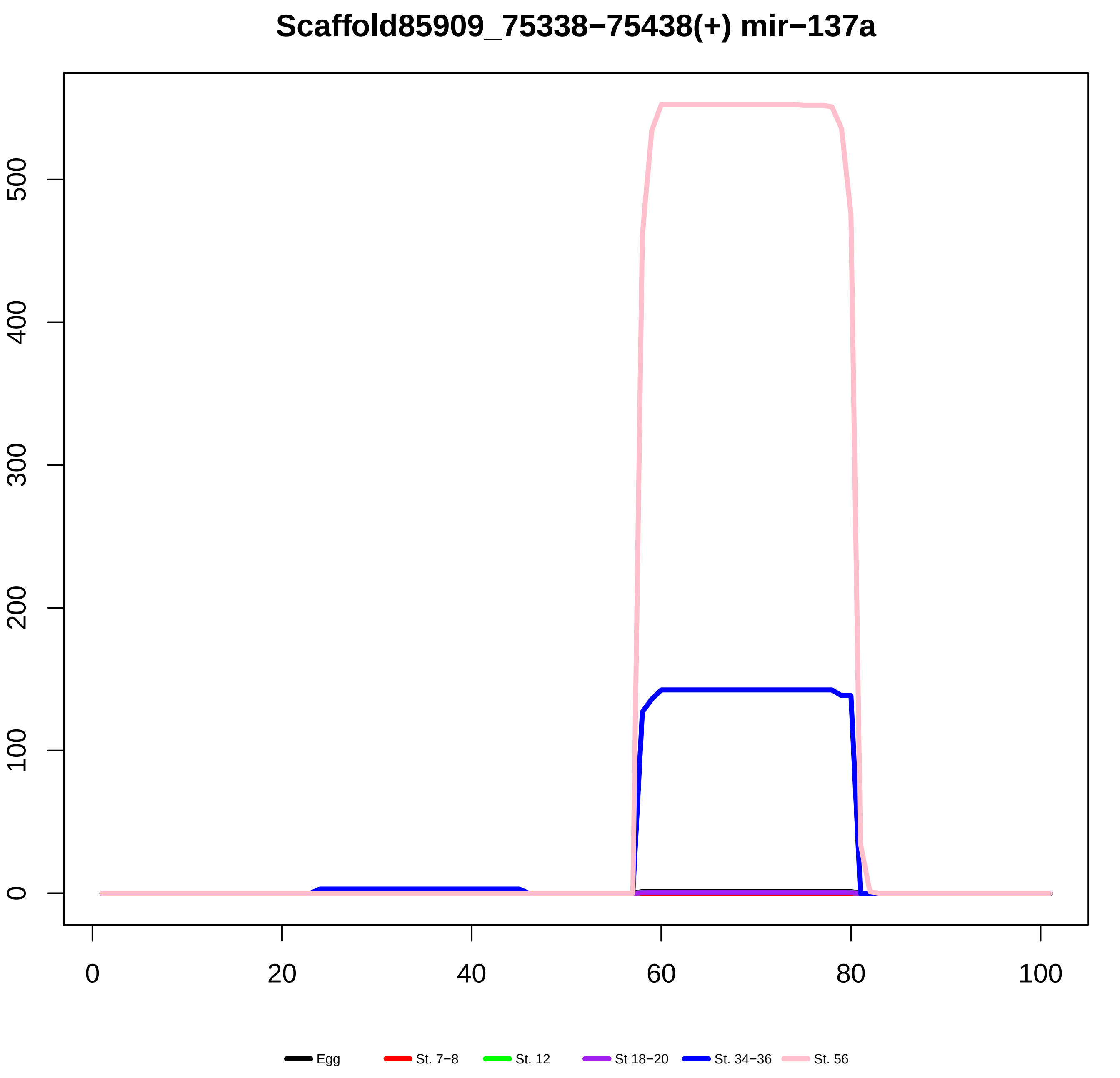

Scaffold85909_75338−75438(+) mir−137a
0
100
200
300
400
500
0
20
40
60
80
100
Egg
St. 7−8
St. 12
St 18−20
St. 34−36
St. 56

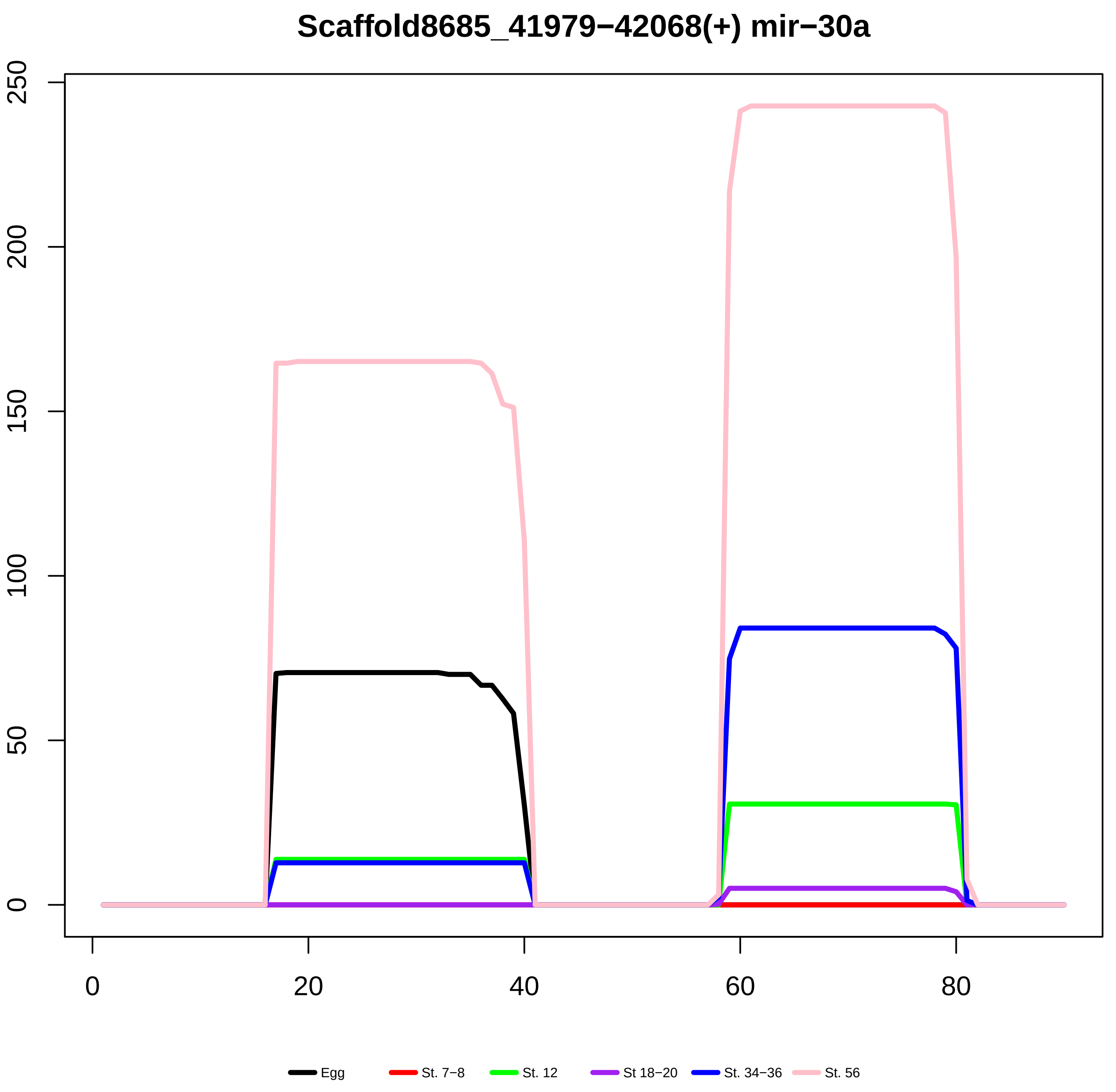
Scaffold8685_41979−42068(+) mir−30a
0
50
100
150
200
250
0
20
40
60
80
Egg
St. 7−8
St. 12
St 18−20
St. 34−36
St. 56

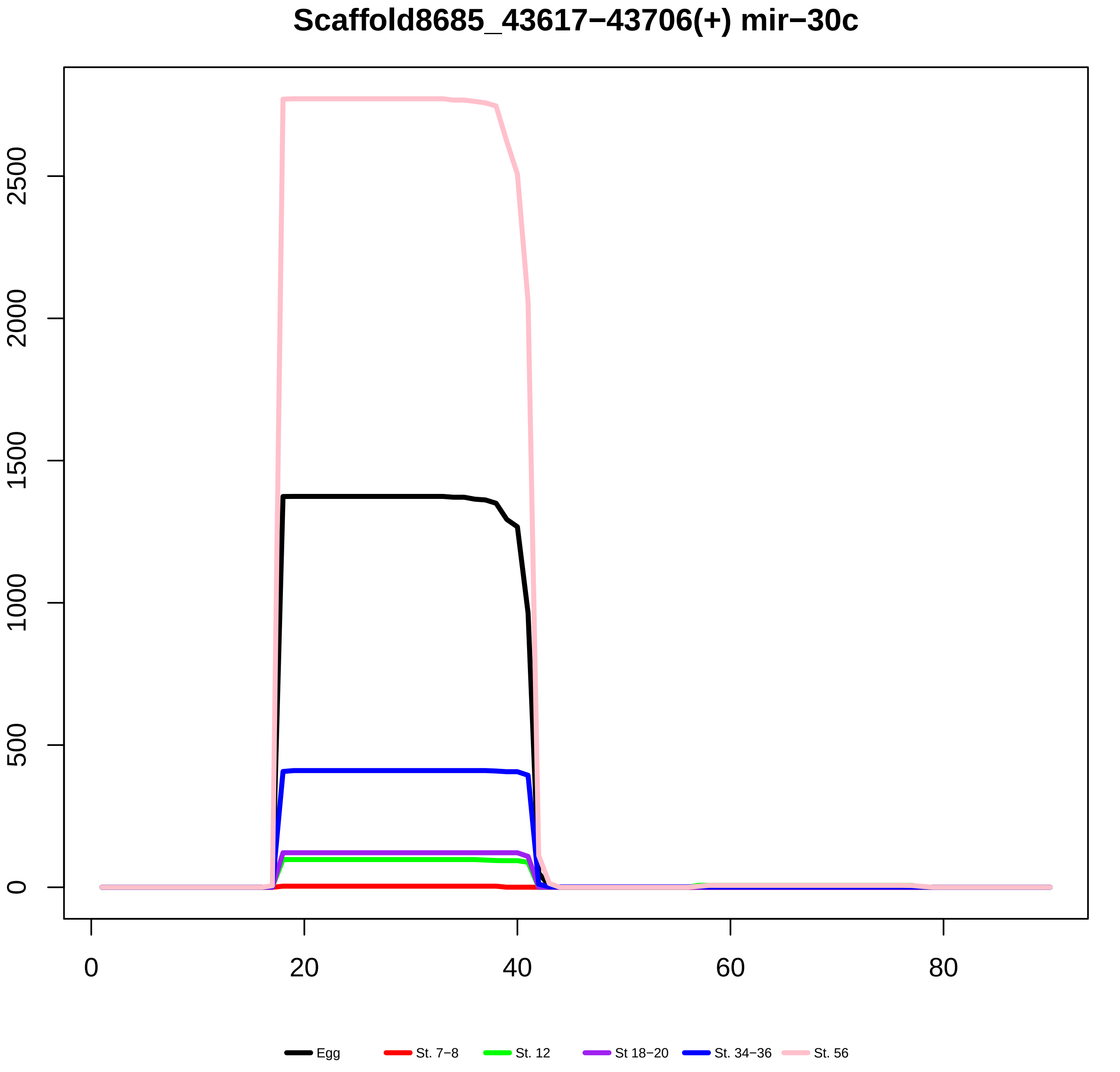

Scaffold8685_43617−43706(+) mir−30c
0
500
1000
1500
2000
2500
0
20
40
60
80
Egg
St. 7−8
St. 12
St 18−20
St. 34−36
St. 56

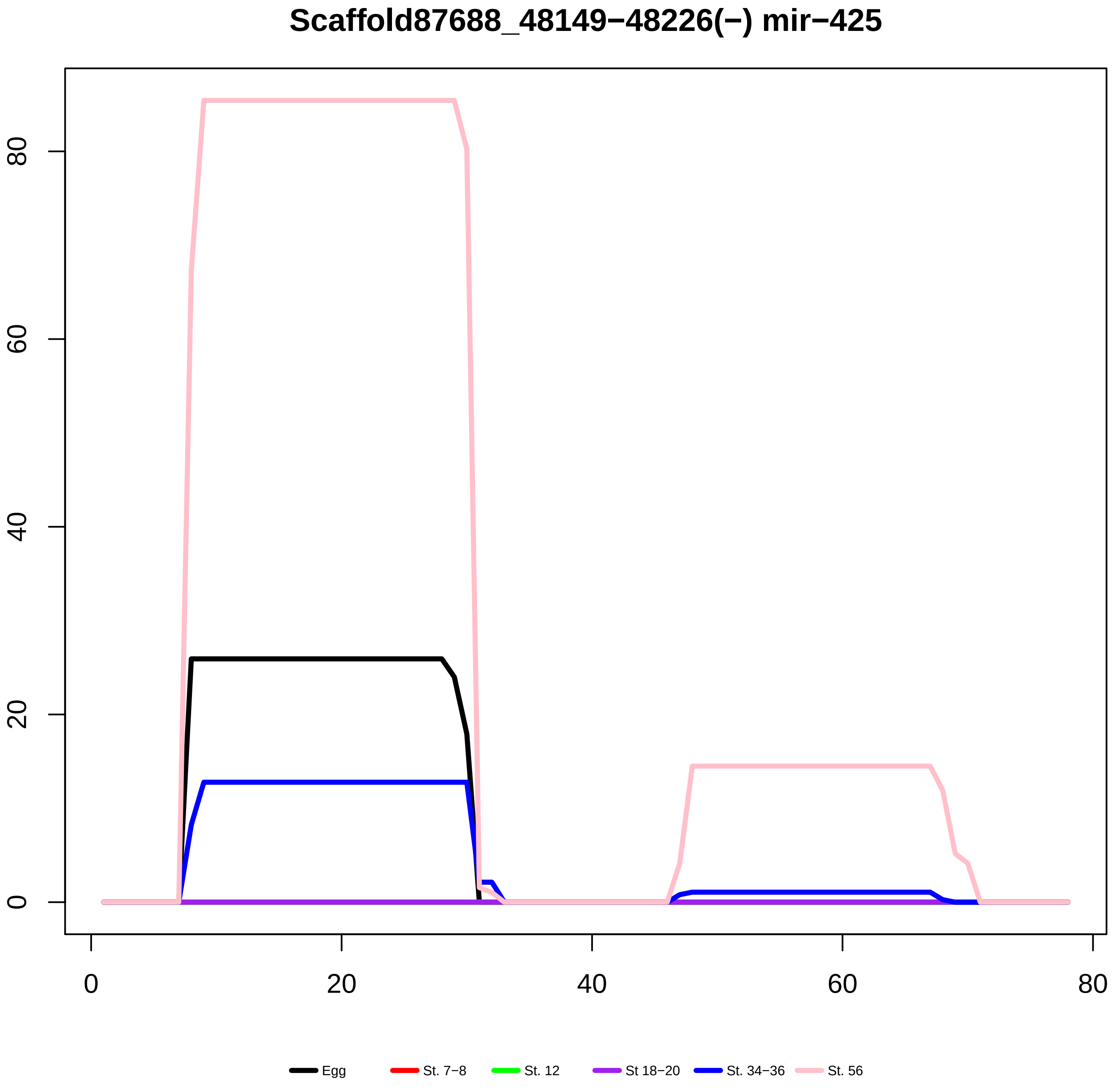

Scaffold87688_48149−48226(−) mir−425
0
20
40
60
80
0
20
40
60
80
Egg
St. 7−8
St. 12
St 18−20
St. 34−36
St. 56

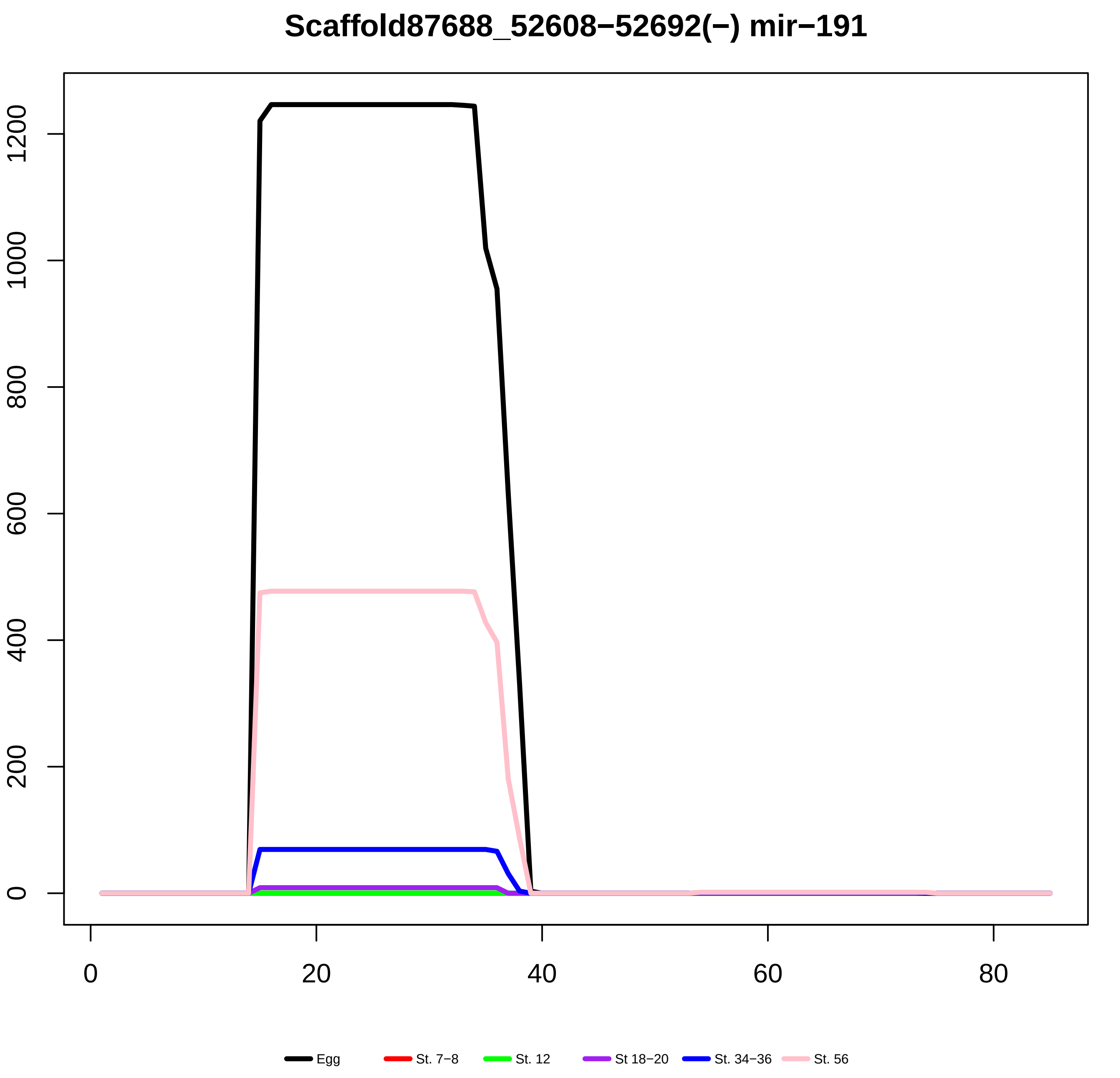

Scaffold87688_52608−52692(−) mir−191
0
200
400
600
800
1000
1200
0
20
40
60
80
Egg
St. 7−8
St. 12
St 18−20
St. 34−36
St. 56

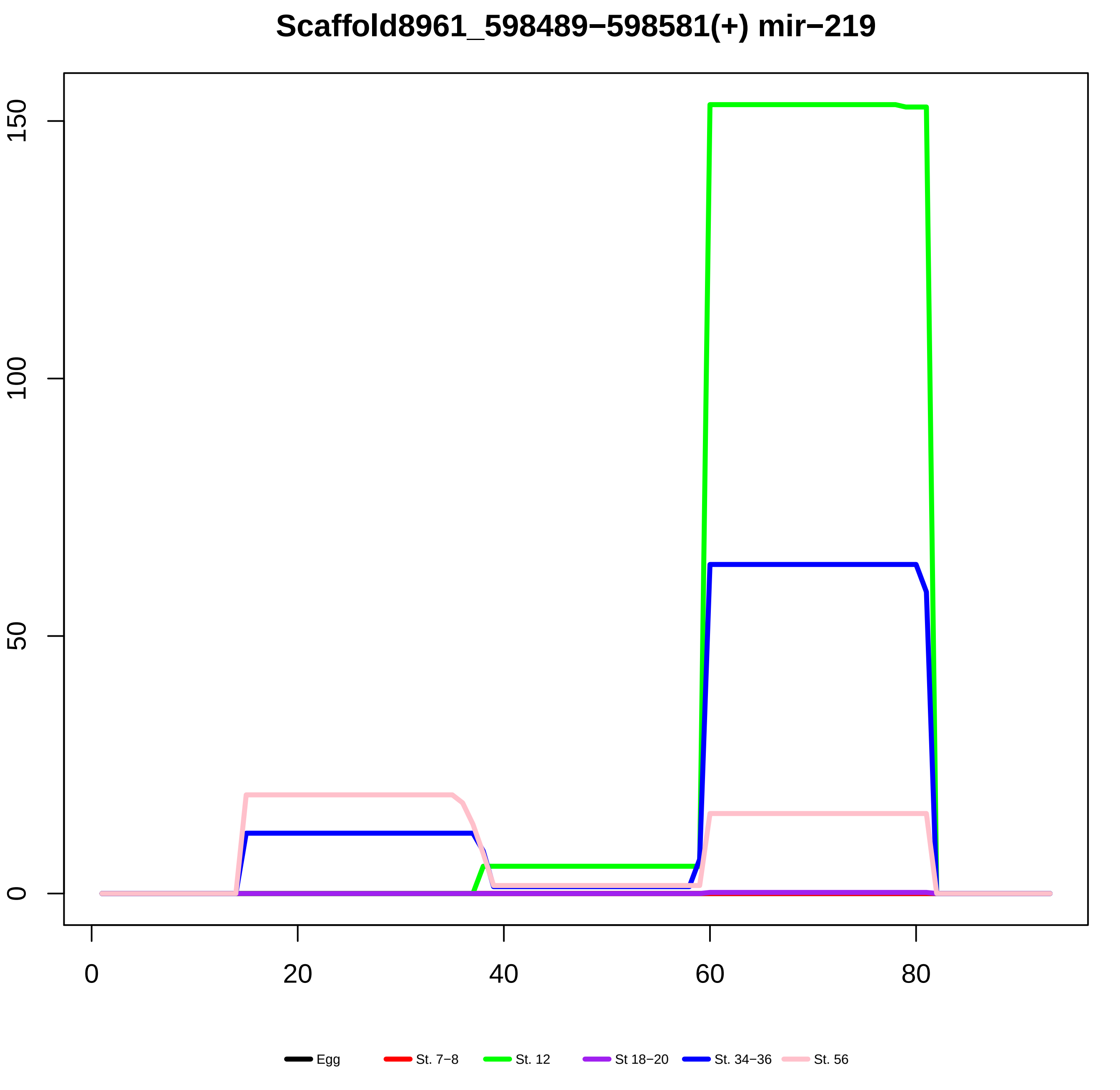

Scaffold8961_598489−598581(+) mir−219
150
100
50
0
0
20
40
60
80
Egg
St. 7−8
St. 12
St 18−20
St. 34−36
St. 56

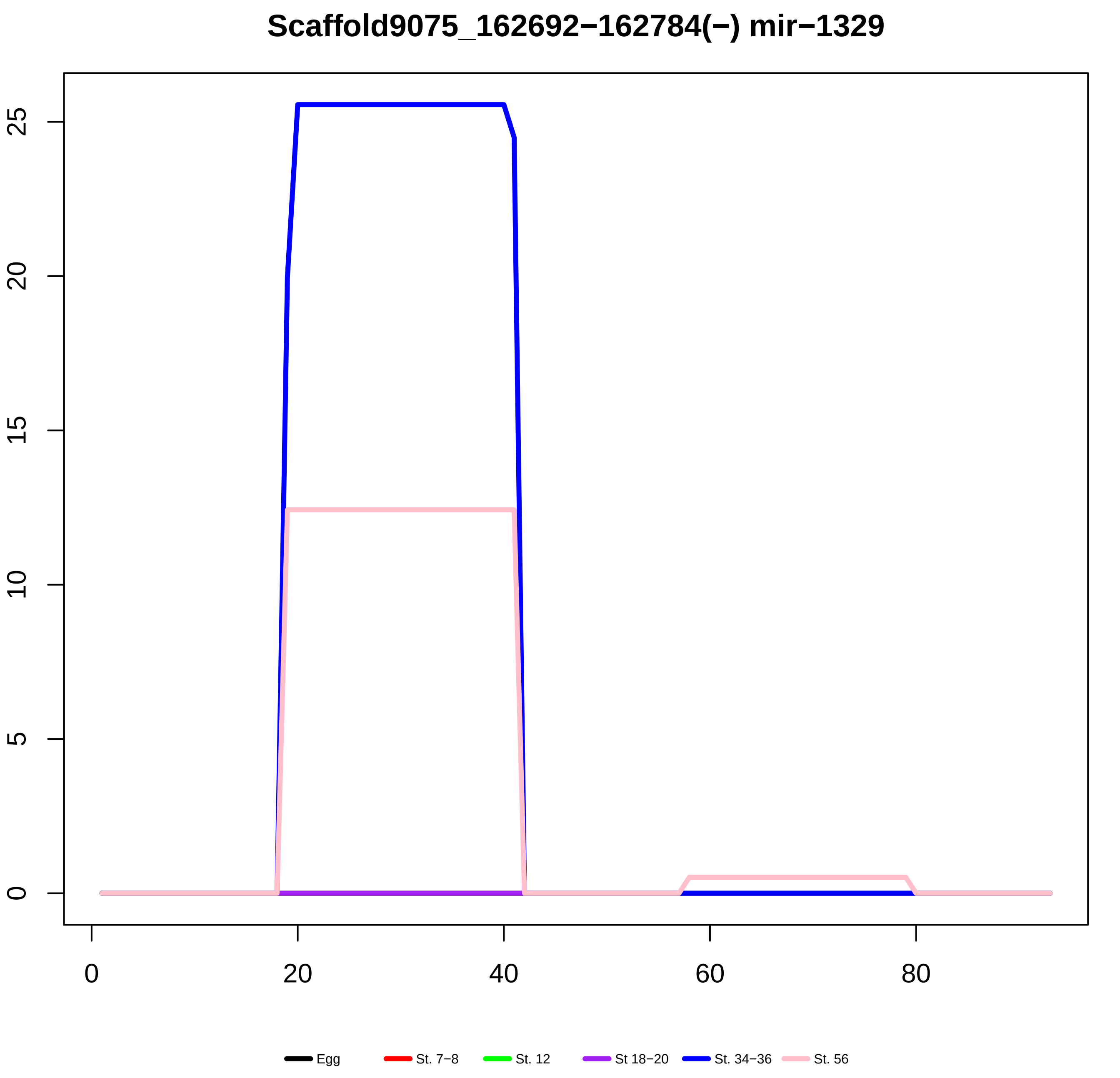
Scaffold9075_162692−162784(−) mir−1329
0
5
10
15
20
25
0
20
40
60
80
Egg
St. 7−8
St. 12
St 18−20
St. 34−36
St. 56

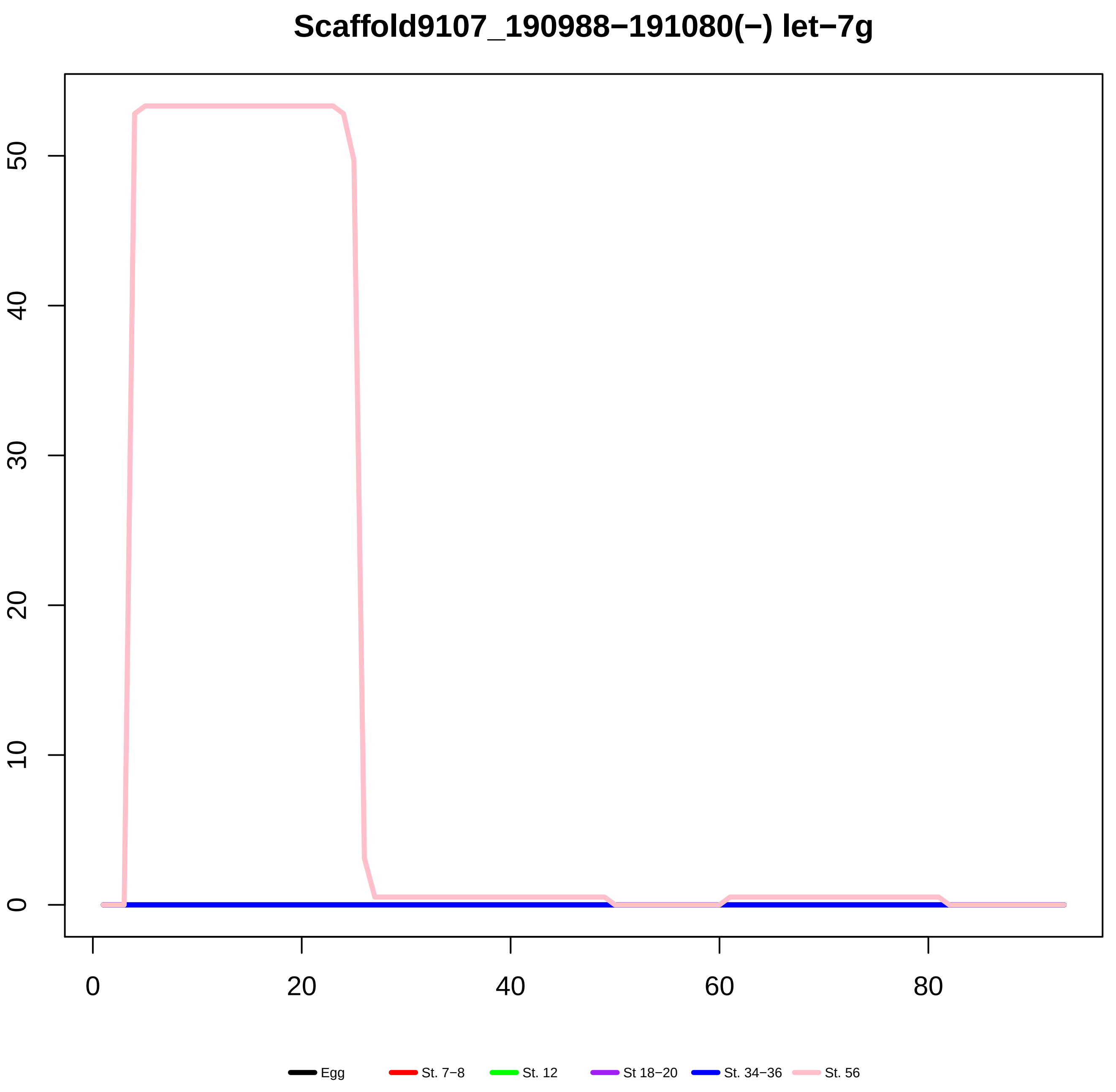

Scaffold9107_190988−191080(−) let−7g
0
10
20
30
40
50
0
20
40
60
80
Egg
St. 7−8
St. 12
St 18−20
St. 34−36
St. 56

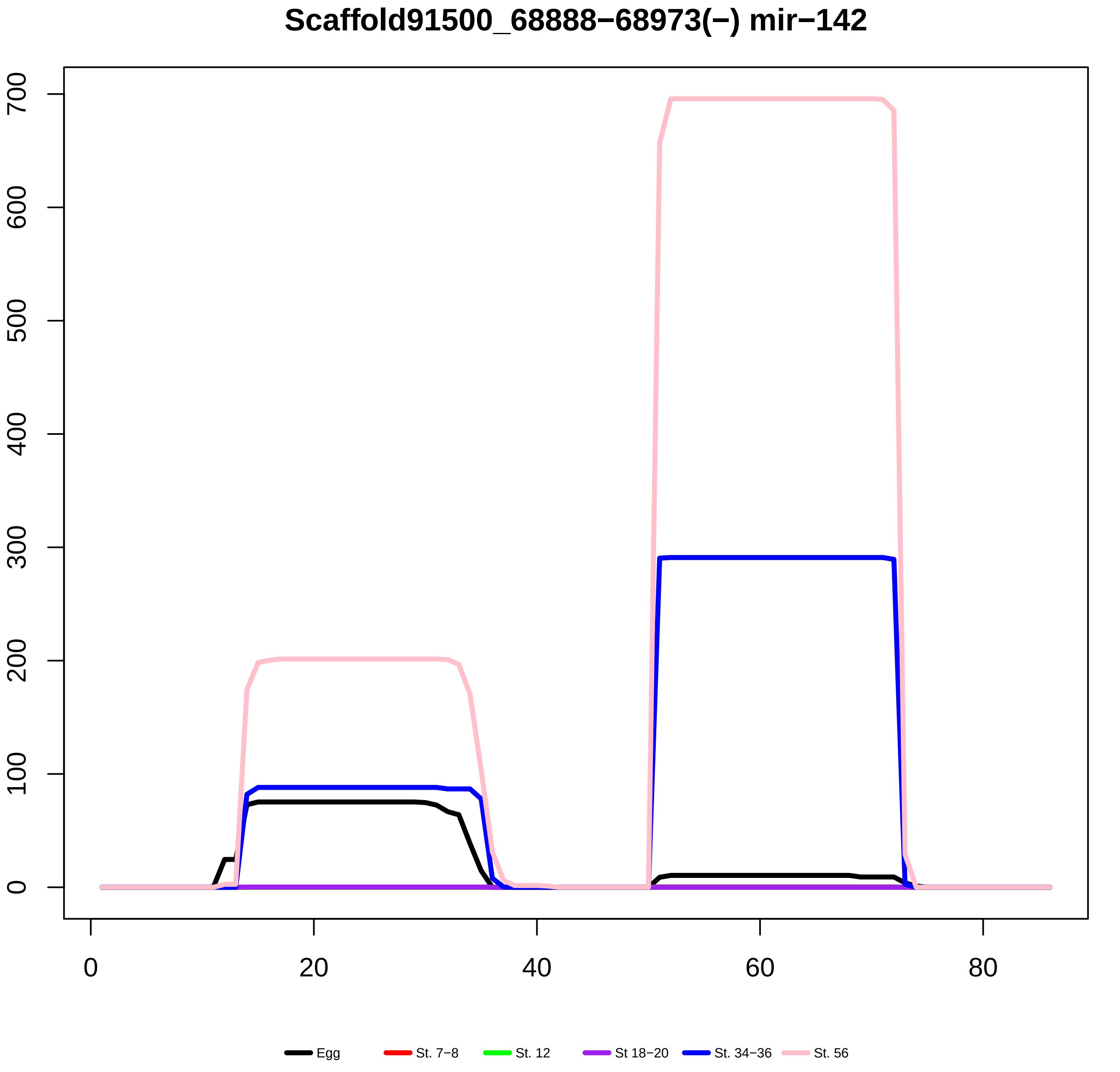
Scaffold91500_68888−68973(−) mir−142
0
100
200
300
400
500
600
700
0
20
40
60
80
Egg
St. 7−8
St. 12
St 18−20
St. 34−36
St. 56

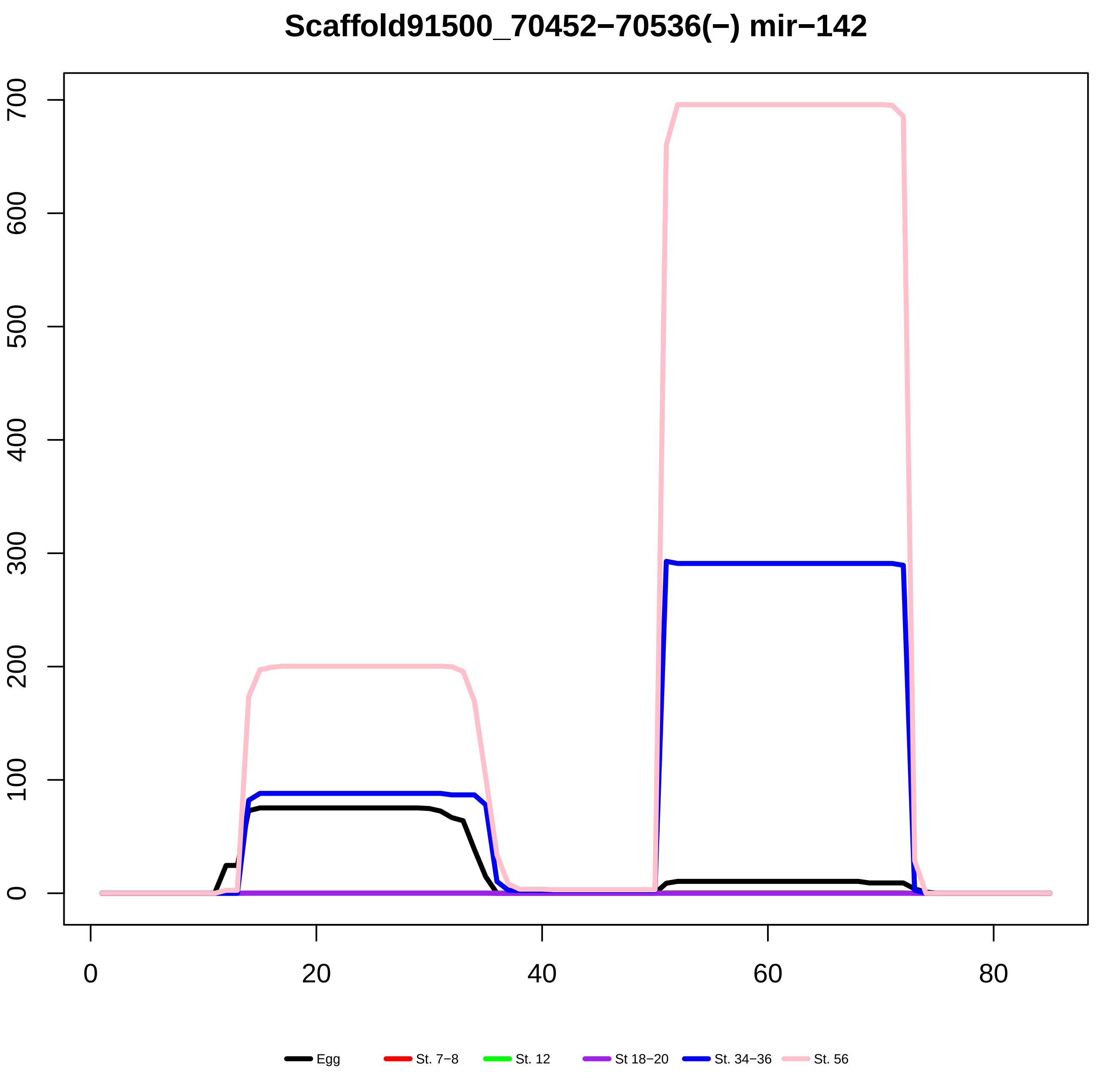

Scaffold91500_70452−70536(−) mir−142
0
100
200
300
400
500
600
700
0
20
40
60
80
Egg
St. 7−8
St. 12
St 18−20
St. 34−36
St. 56

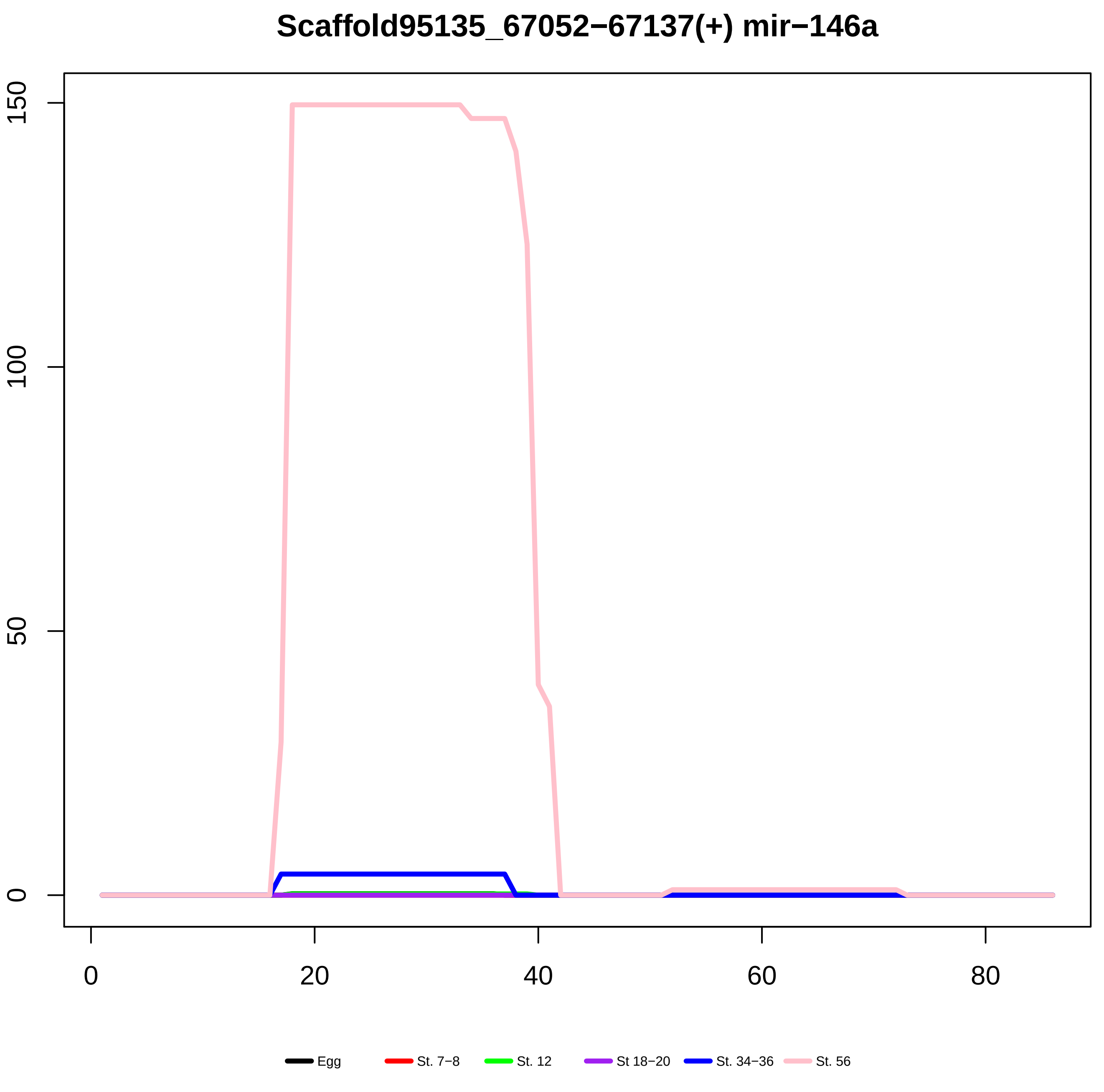

Scaffold95135_67052−67137(+) mir−146a
0
50
100
150
0
20
40
60
80
Egg
St. 7−8
St. 12
St 18−20
St. 34−36
St. 56

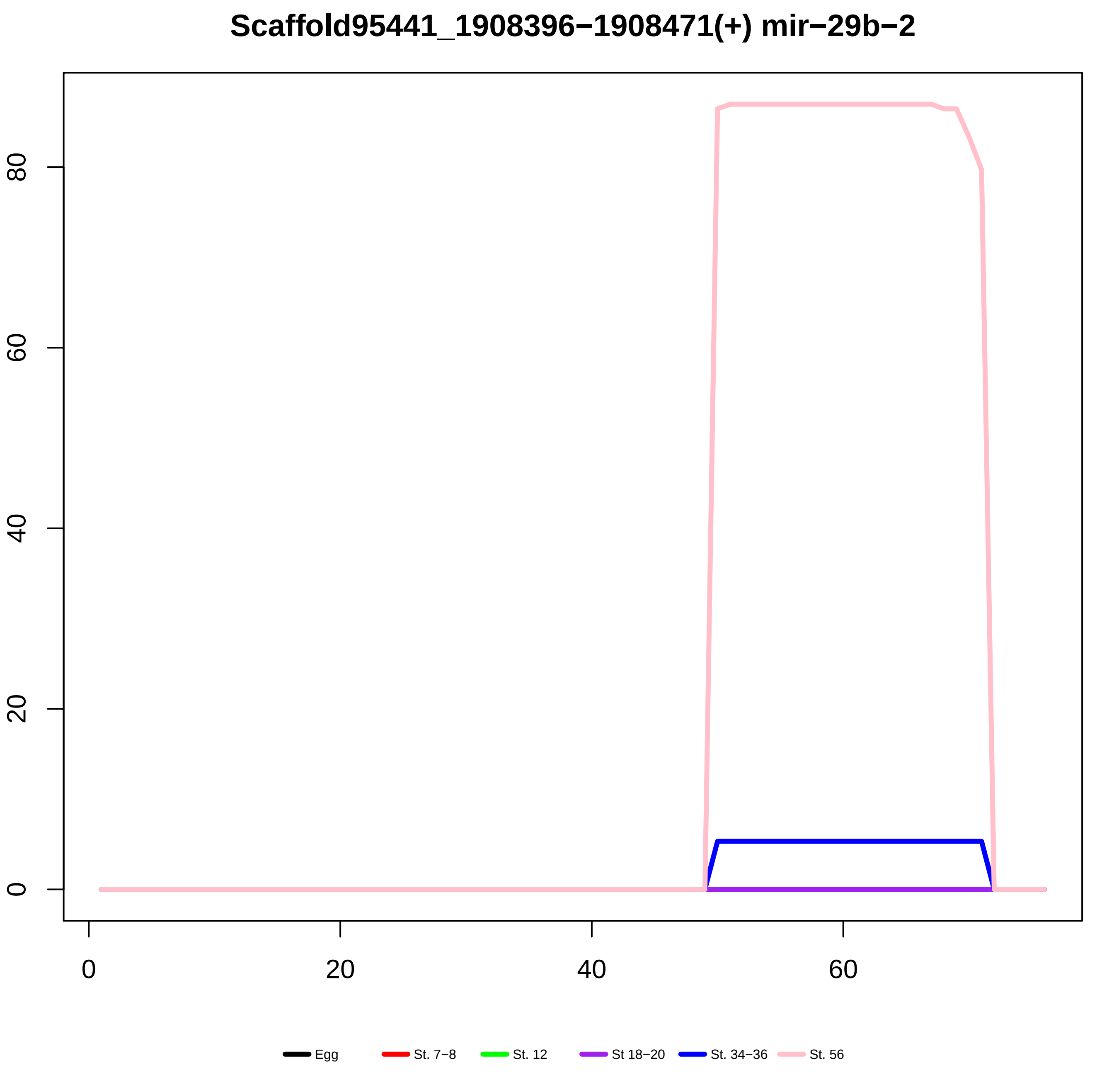
Scaffold95441_1908396−1908471(+) mir−29b−2
0
20
40
60
80
0
20
40
60
Egg
St. 7−8
St. 12
St 18−20
St. 34−36
St. 56

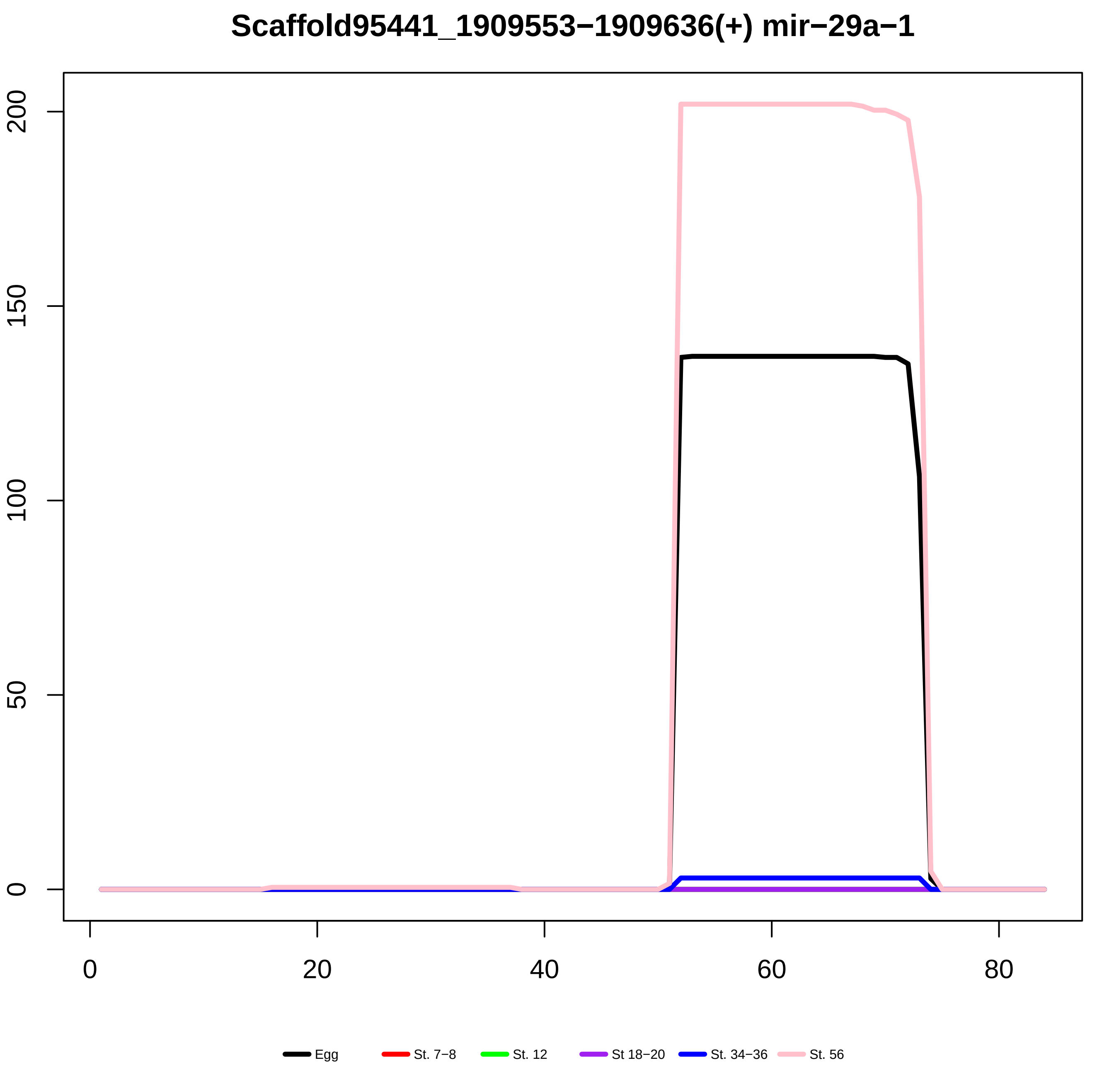
Scaffold95441_1909553−1909636(+) mir−29a−1
0
50
100
150
200
0
20
40
60
80
Egg
St. 7−8
St. 12
St 18−20
St. 34−36
St. 56

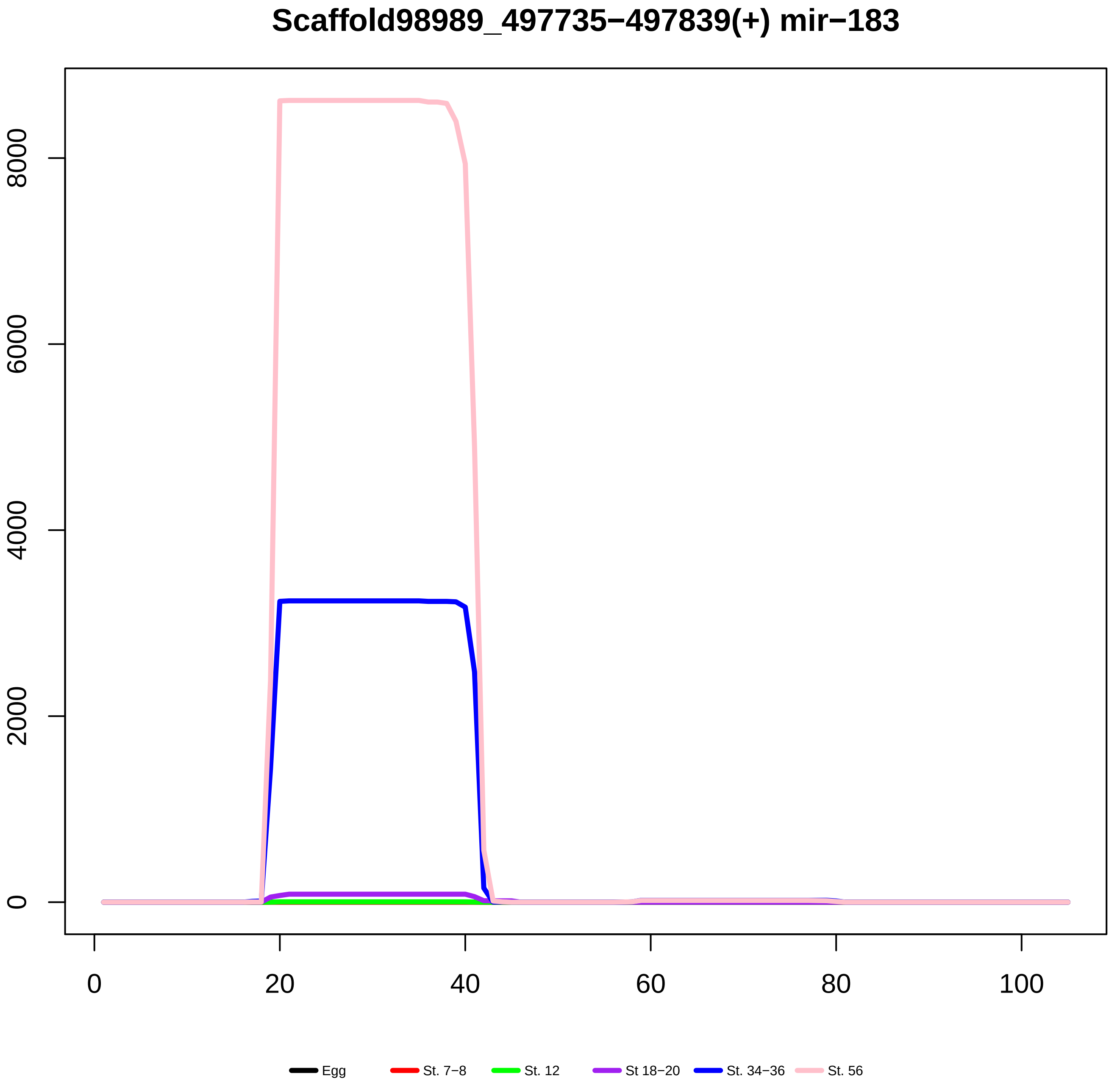
Scaffold98989_497735−497839(+) mir−183
0
2000
4000
6000
8000
0
20
40
60
80
100
Egg
St. 7−8
St. 12
St 18−20
St. 34−36
St. 56
