# Supplementary material for: A Database of microRNA Expression Patterns in Xenopus laevis
Source: PLoS One. 2015 Oct 27;10(10):e0138313. doi: 10.1371/journal.pone.0138313 (PMC4624429; doi:10.1371/journal.pone.0138313)
Supplement: S3 Fig — (PDF) [file pone.0138313.s003.pdf]

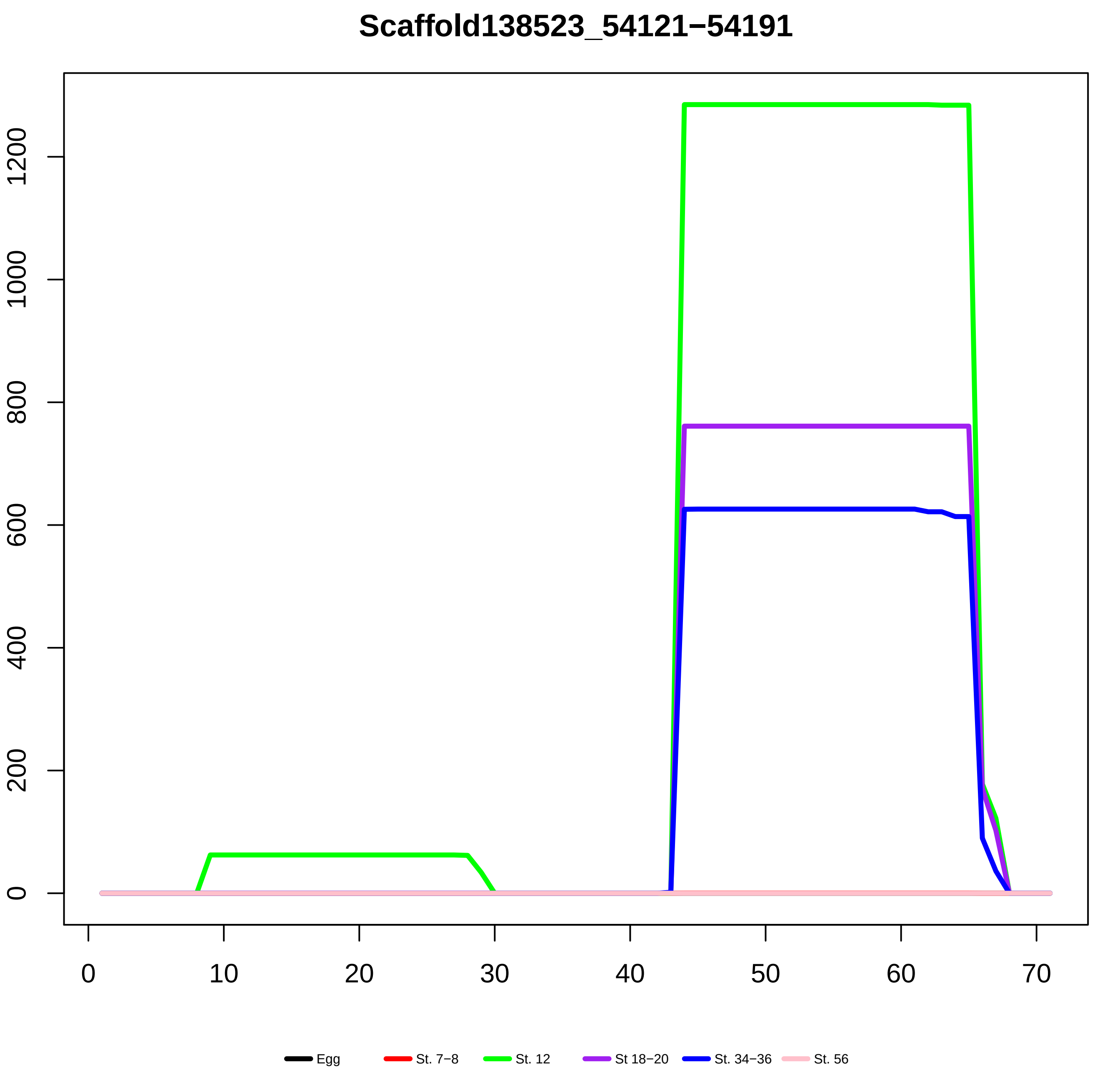

Scaffold138523_54121−54191
0
200
400
600
800
1000
1200
0
10
20
30
40
50
60
70
Egg
St. 7−8
St. 12
St 18−20
St. 34−36
St. 56

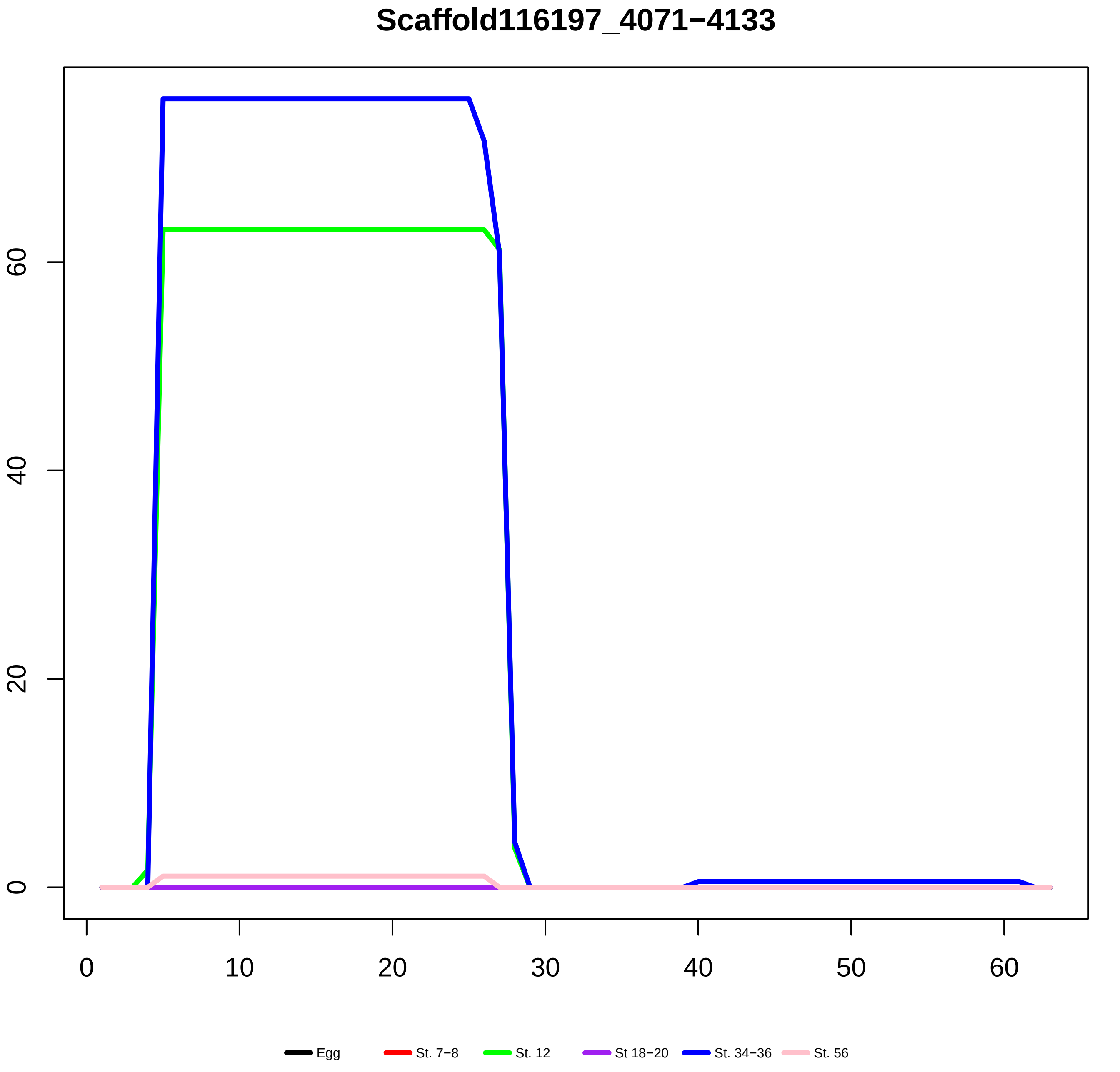

Scaffold116197_4071−4133
0
20
40
60
0
10
20
30
40
50
60
Egg
St. 7−8
St. 12
St 18−20
St. 34−36
St. 56

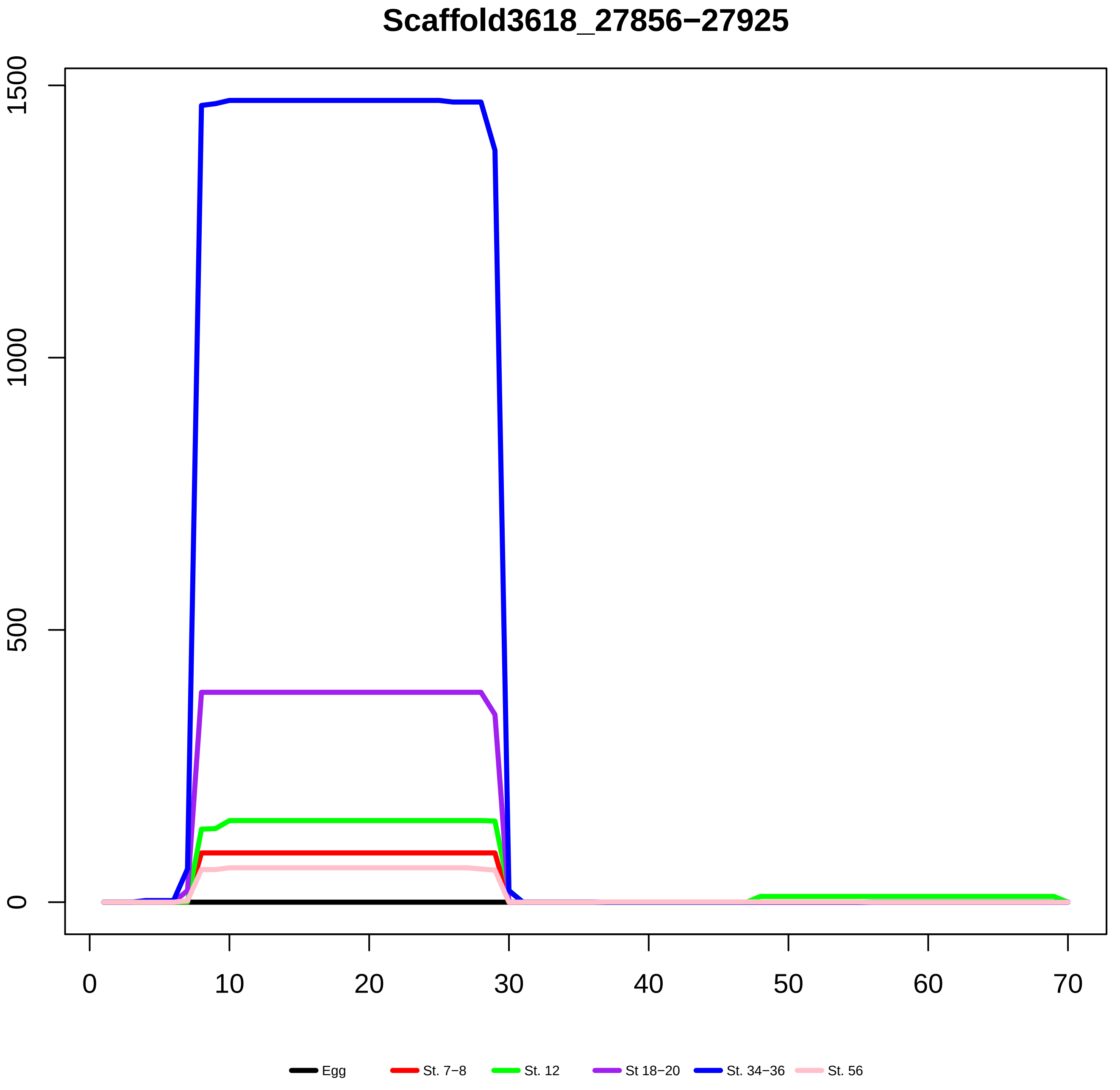

Scaffold3618_27856−27925
0
500
1000
1500
0
10
20
30
40
50
60
70
Egg
St. 7−8
St. 12
St 18−20
St. 34−36
St. 56

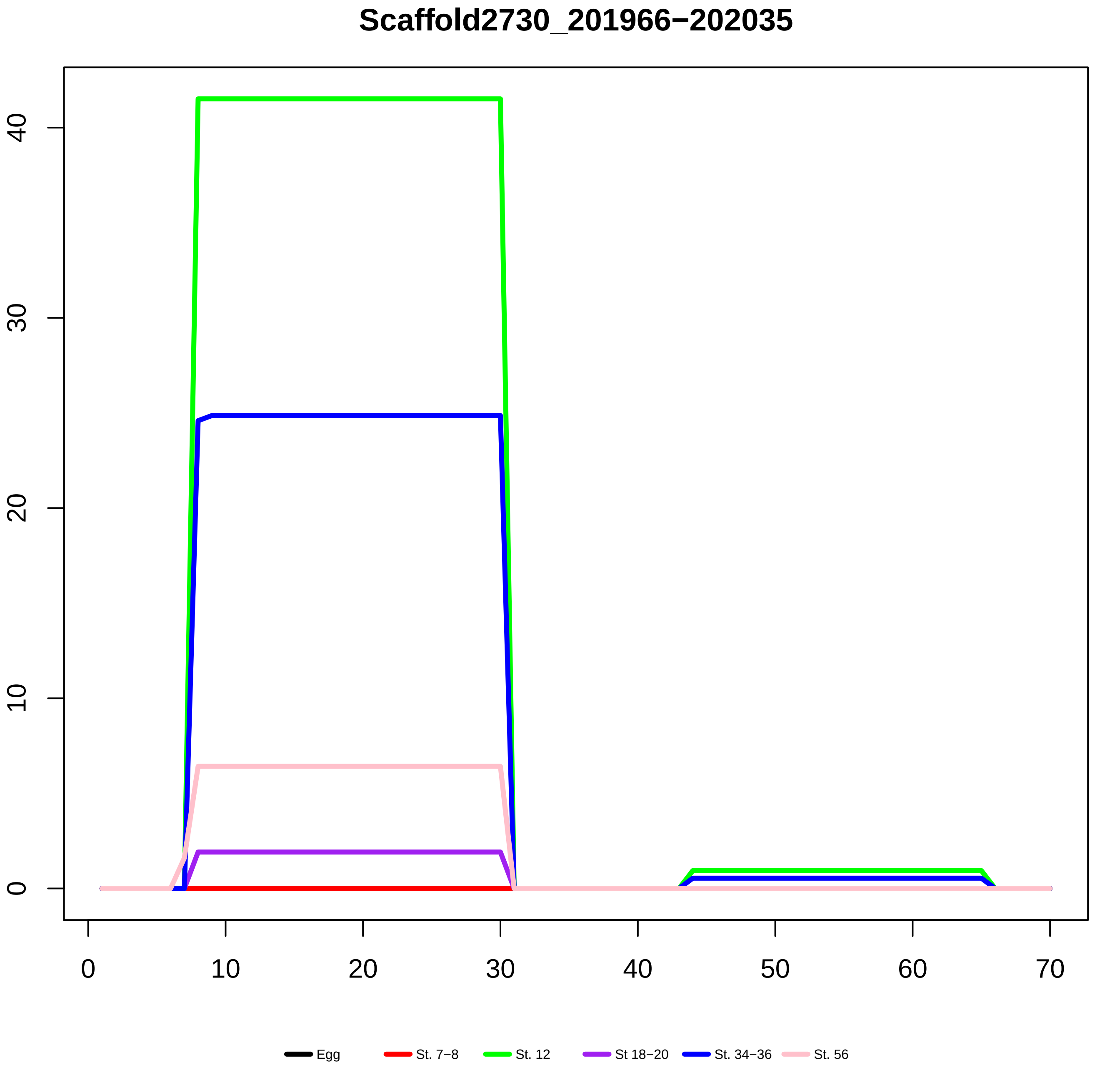

Scaffold2730_201966−202035
0
10
20
30
40
0
10
20
30
40
50
60
70
Egg
St. 7−8
St. 12
St 18−20
St. 34−36
St. 56

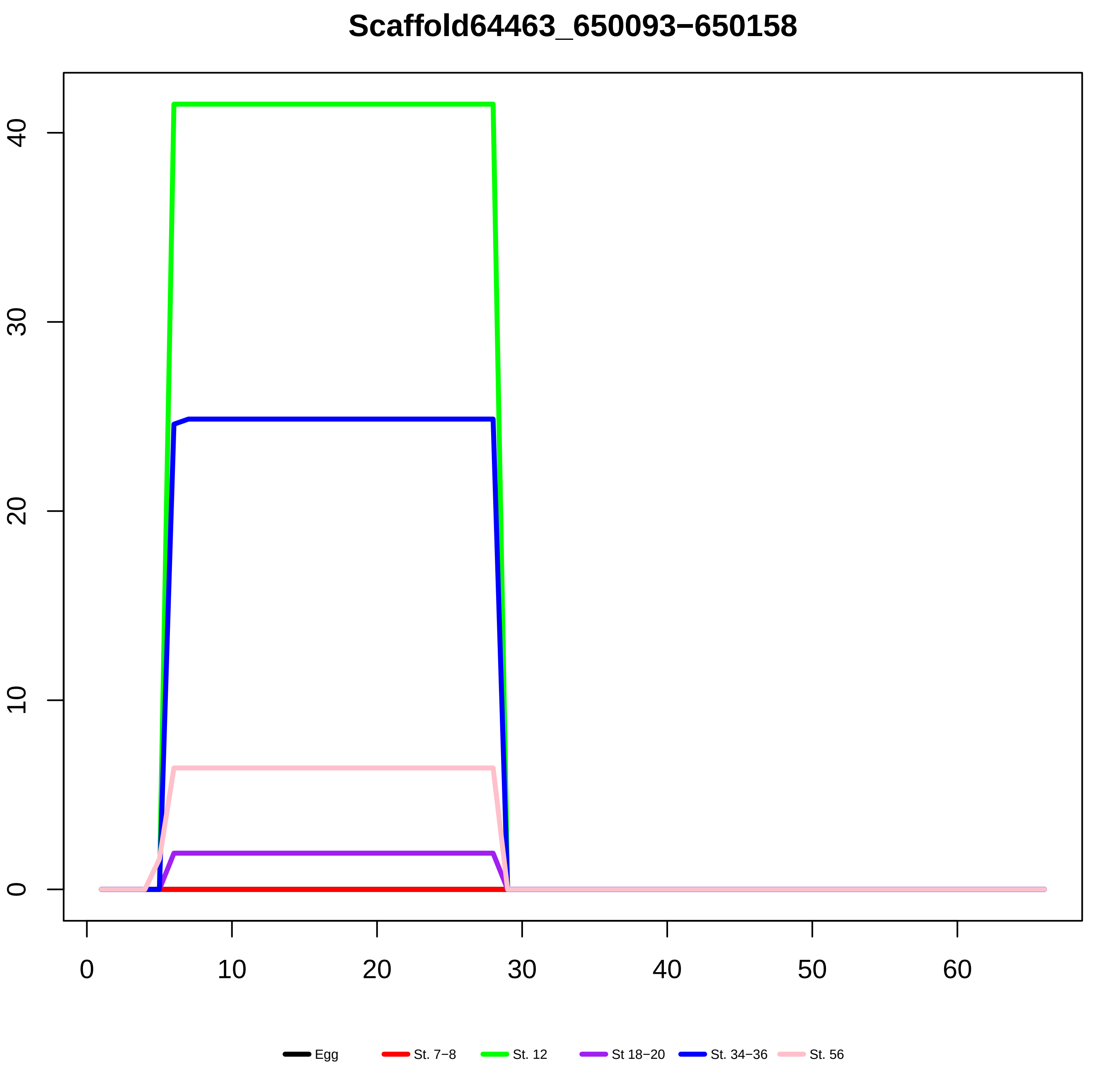

Scaffold64463_650093−650158
0
10
20
30
40
0
10
20
30
40
50
60
Egg
St. 7−8
St. 12
St 18−20
St. 34−36
St. 56

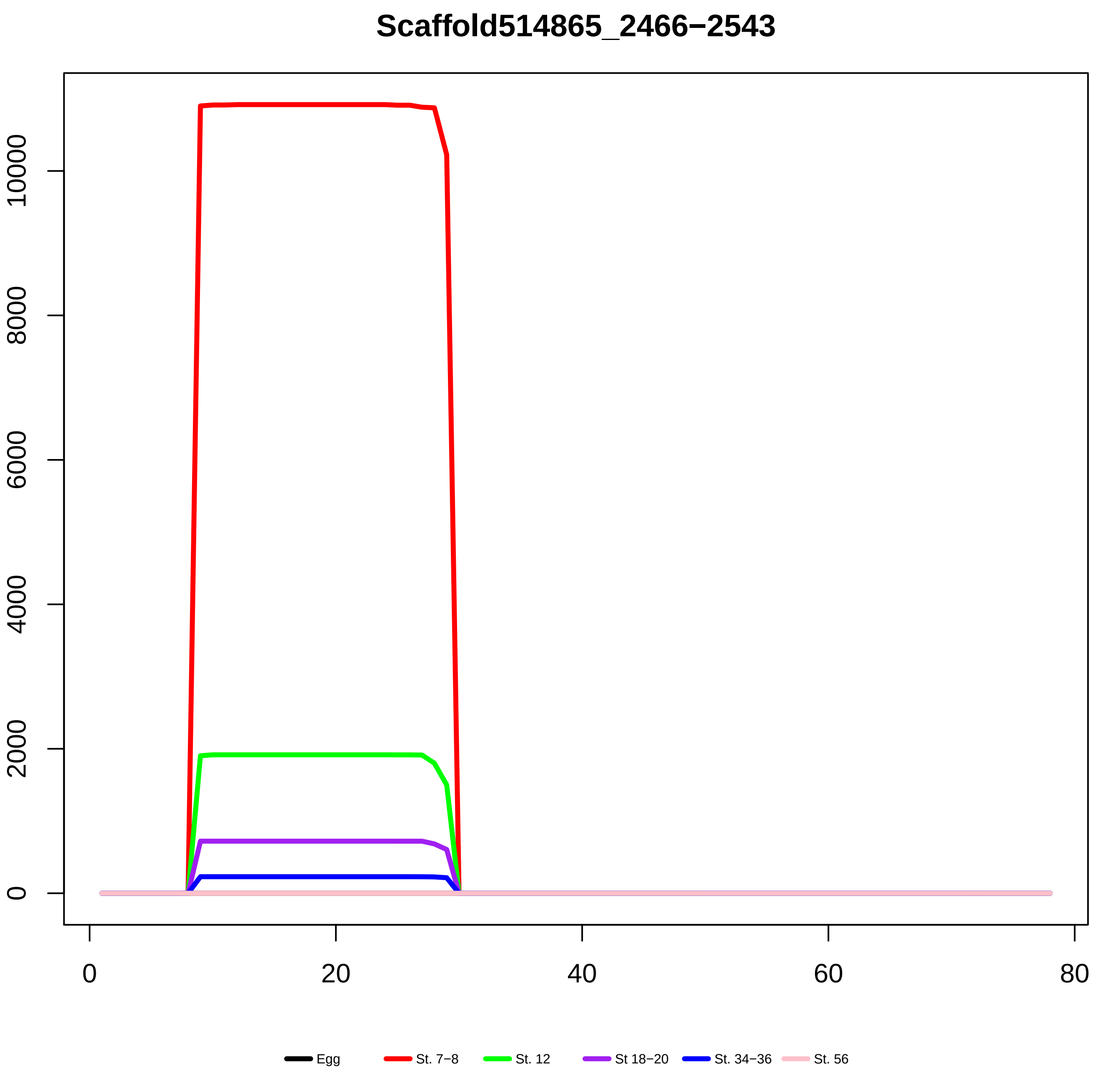

Scaffold514865_2466−2543
0
2000
4000
6000
8000
10000
0
20
40
60
80
Egg
St. 7−8
St. 12
St 18−20
St. 34−36
St. 56

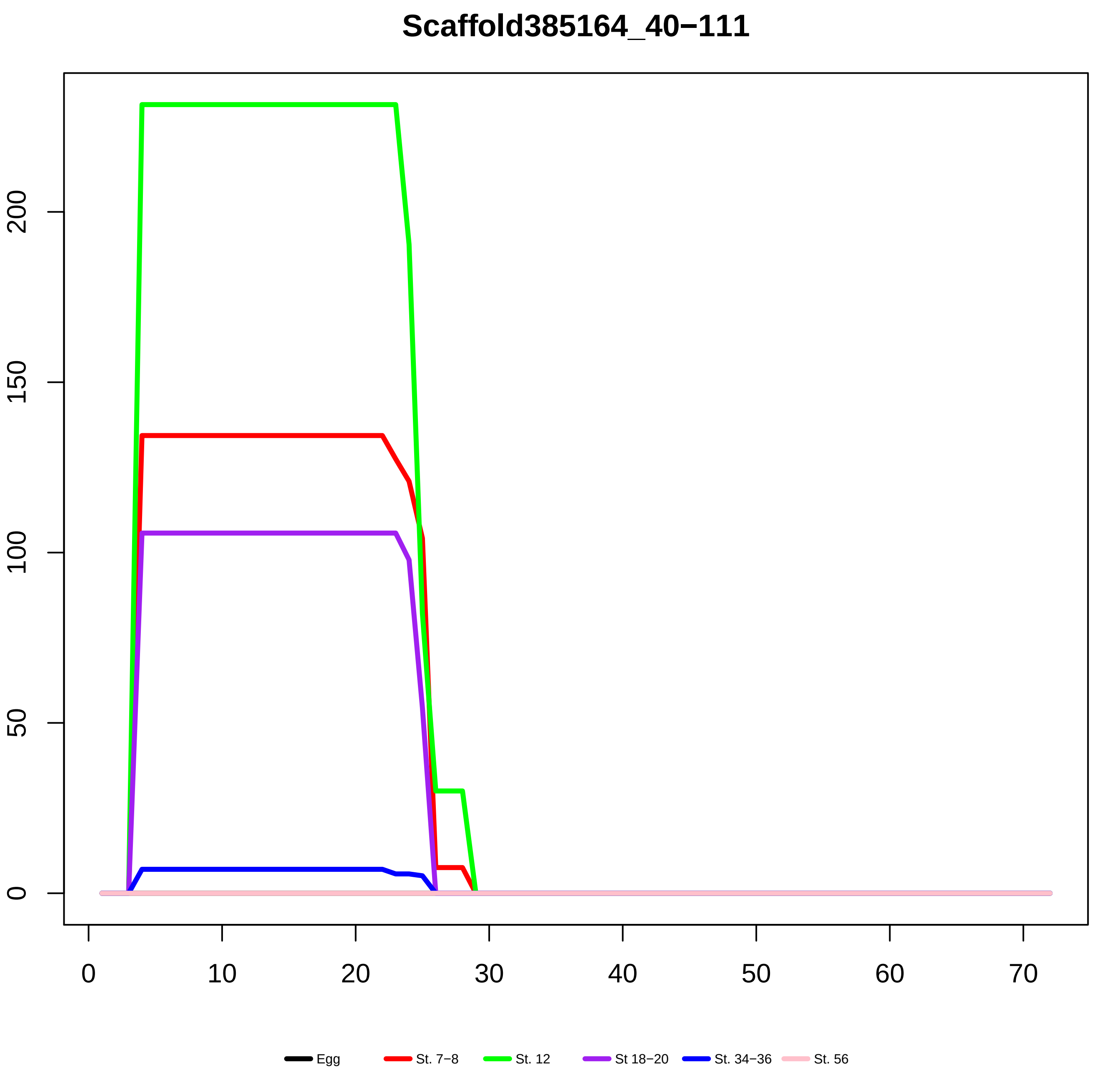
Scaffold385164_40−111
0
50
100
150
200
0
10
20
30
40
50
60
70
Egg
St. 7−8
St. 12
St 18−20
St. 34−36
St. 56

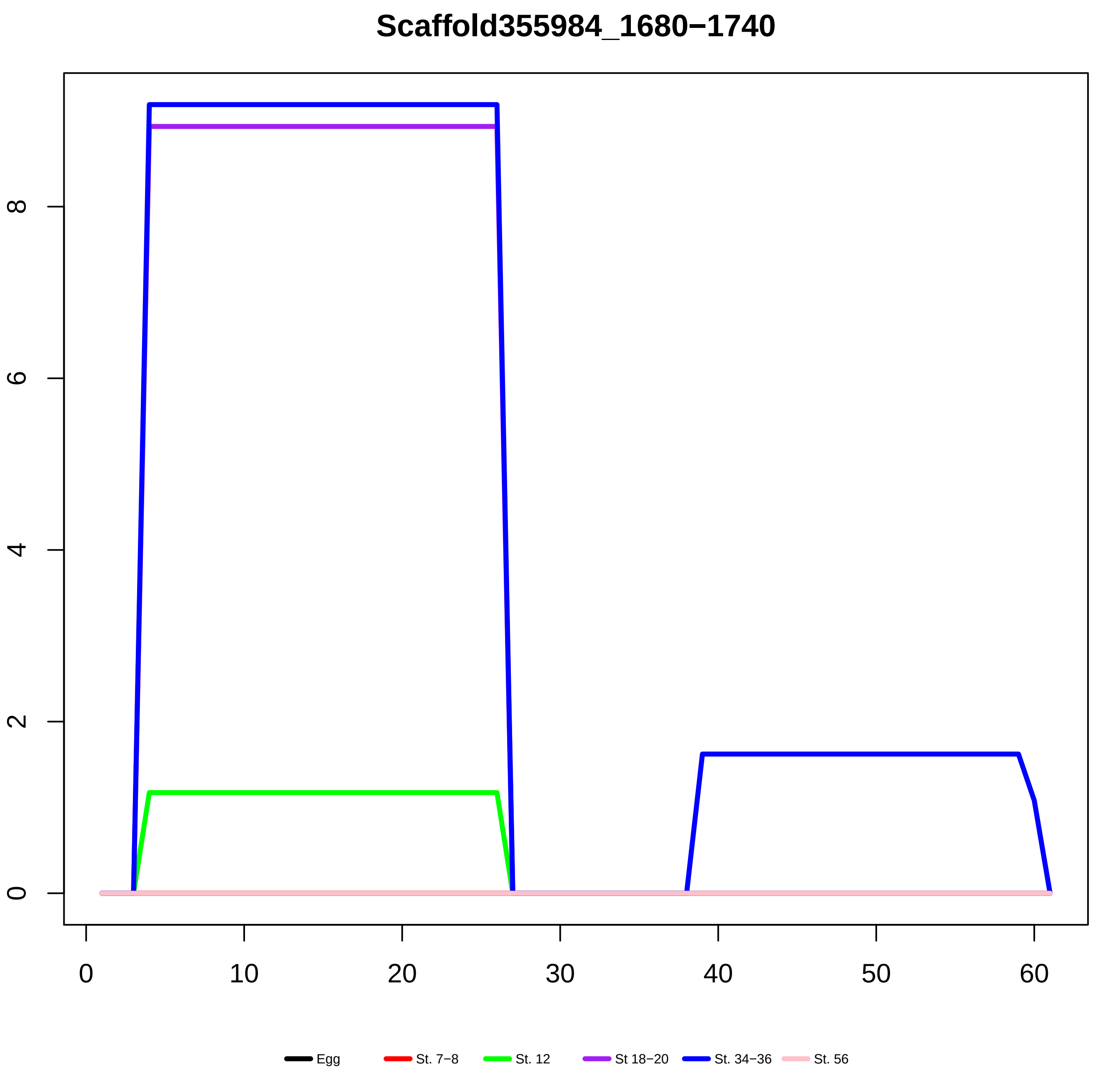

Scaffold355984_1680−1740
0
2
4
6
8
0
10
20
30
40
50
60
Egg
St. 7−8
St. 12
St 18−20
St. 34−36
St. 56

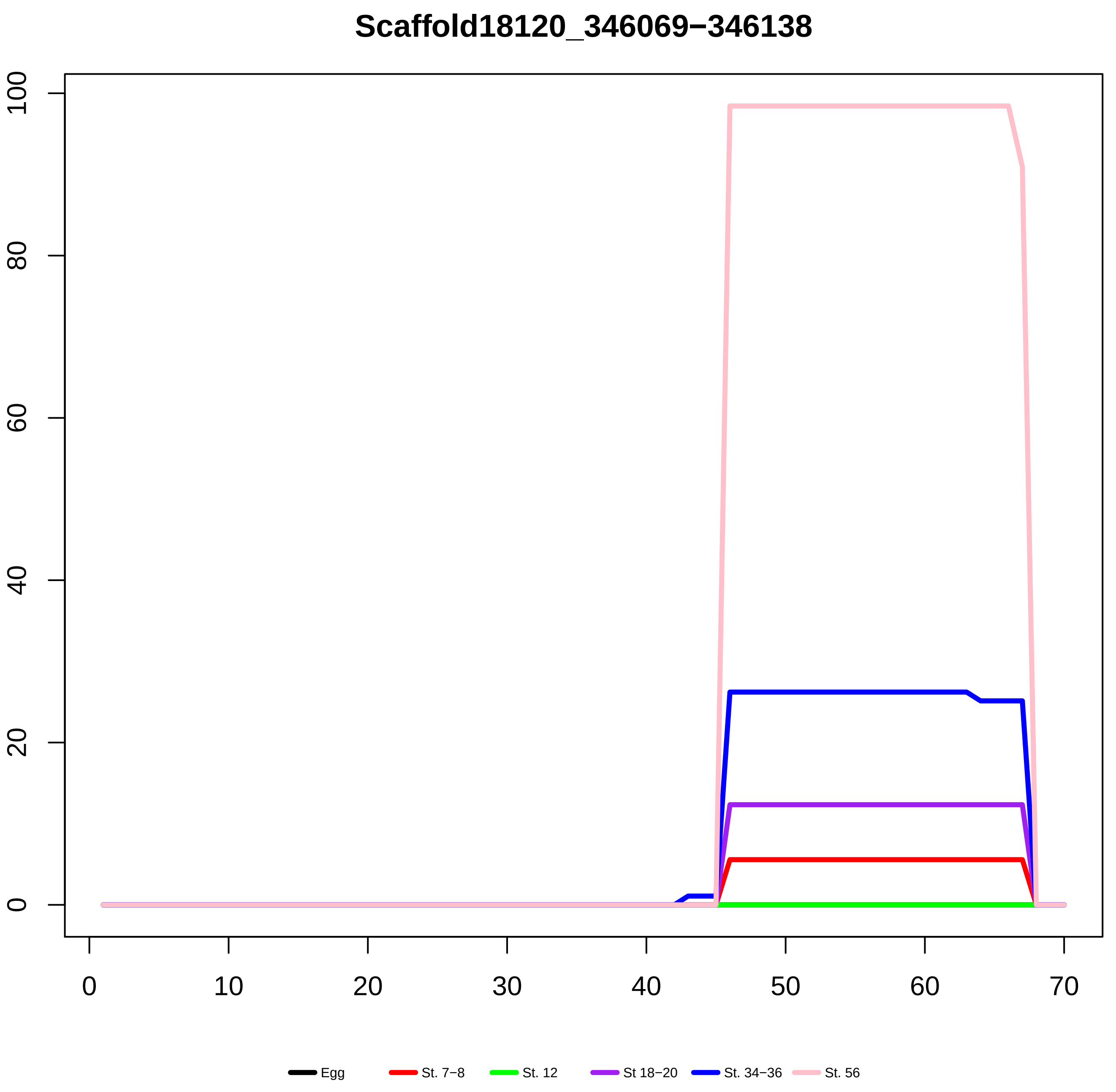

Scaffold18120_346069−346138
0
20
40
60
80
100
0
10
20
30
40
50
60
70
Egg
St. 7−8
St. 12
St 18−20
St. 34−36
St. 56

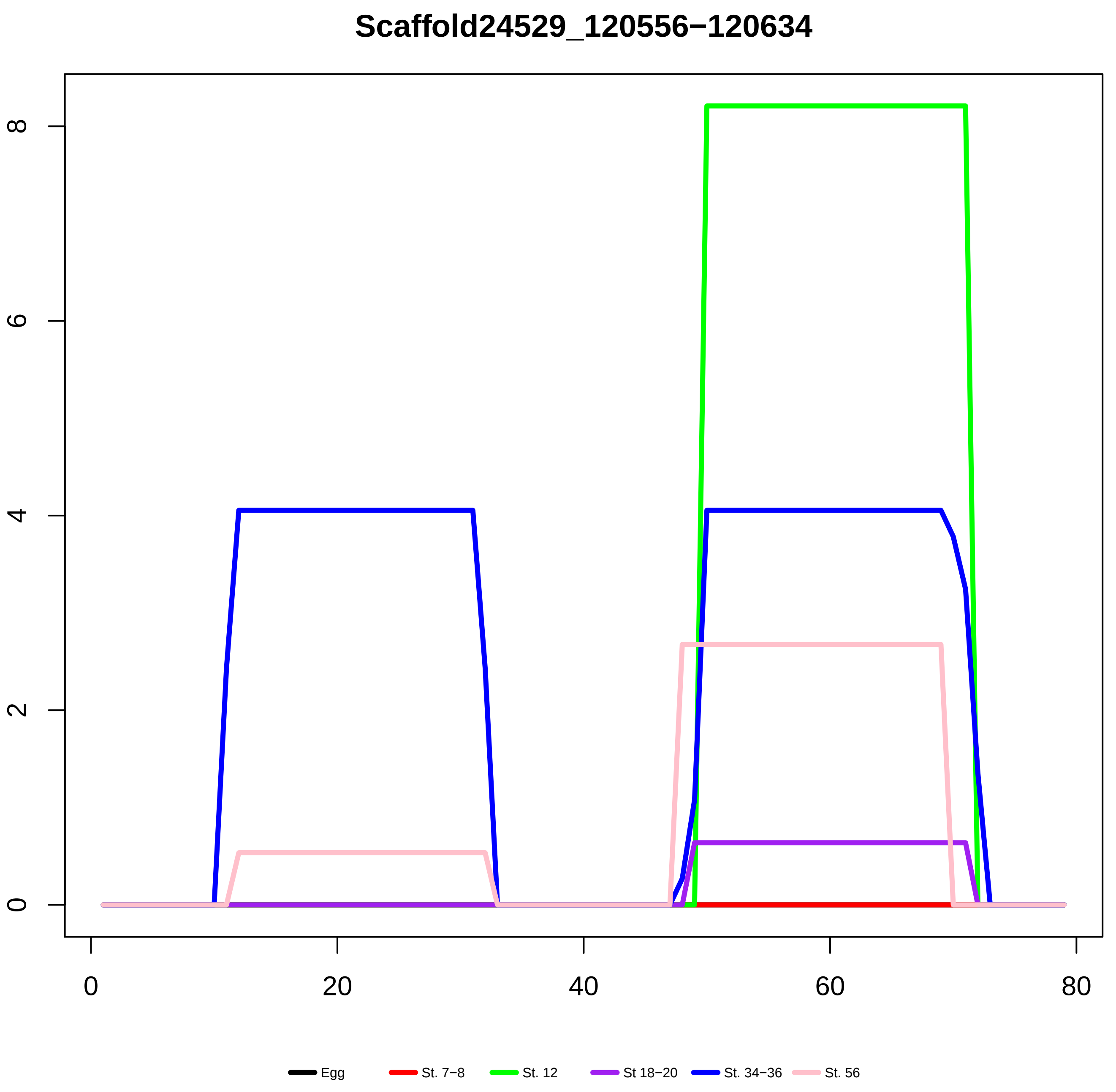
Scaffold24529_120556−120634
0
2
4
6
8
0
20
40
60
80
Egg
St. 7−8
St. 12
St 18−20
St. 34−36
St. 56

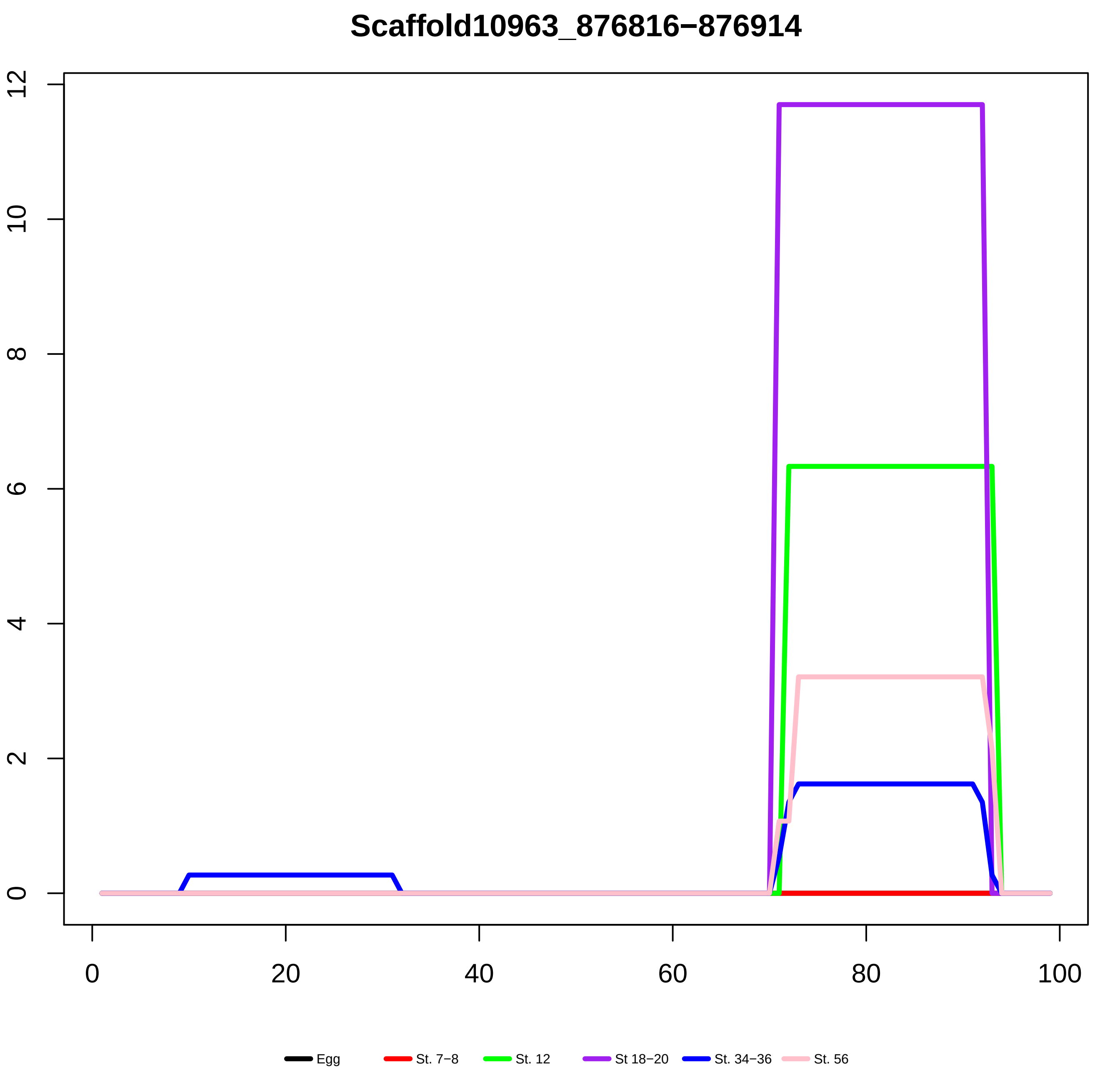

Scaffold10963_876816−876914
0
2
4
6
8
10
12
0
20
40
60
80
100
Egg
St. 7−8
St. 12
St 18−20
St. 34−36
St. 56

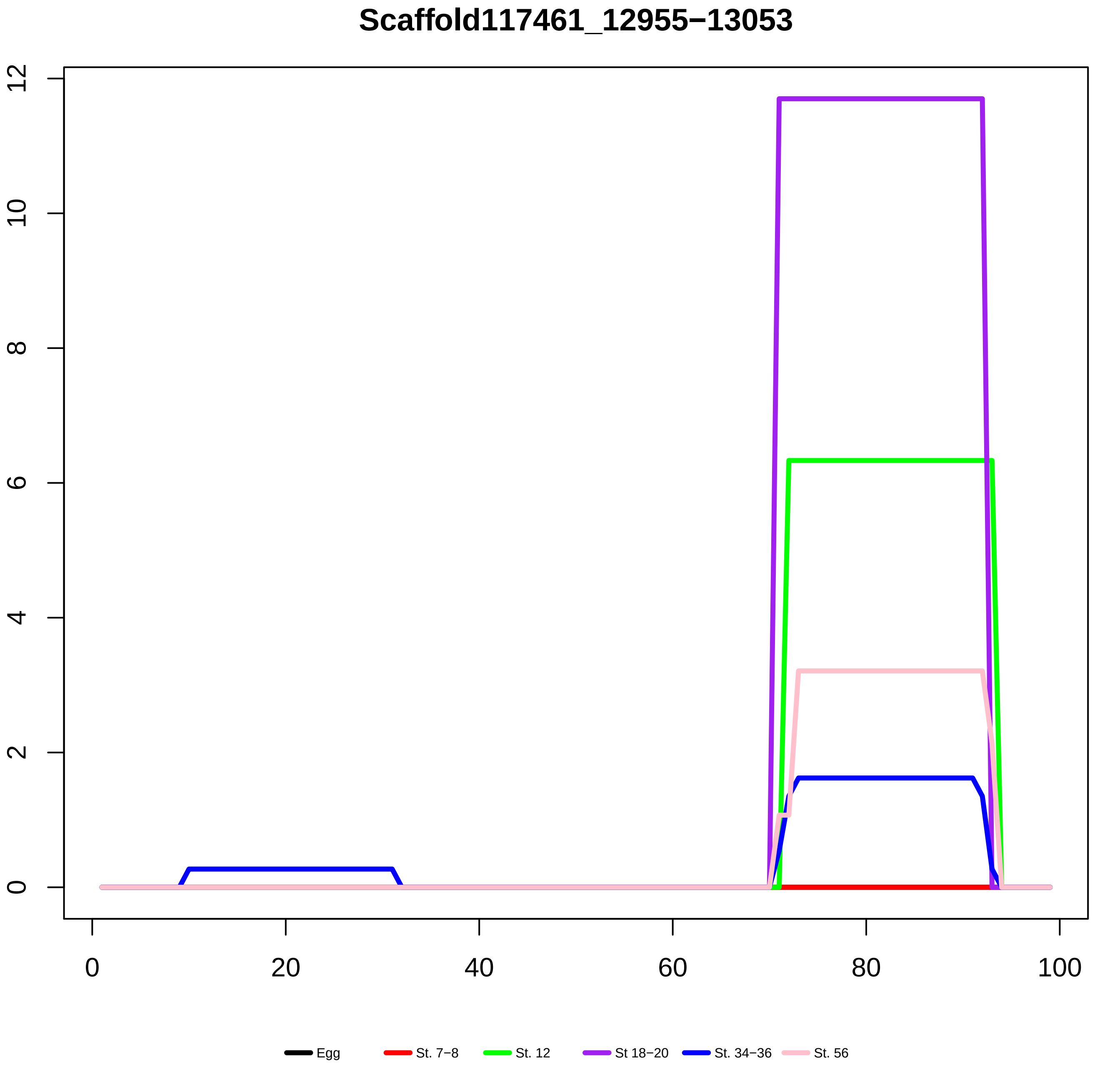
Scaffold117461_12955−13053
0
2
4
6
8
10
12
0
20
40
60
80
100
Egg
St. 7−8
St. 12
St 18−20
St. 34−36
St. 56

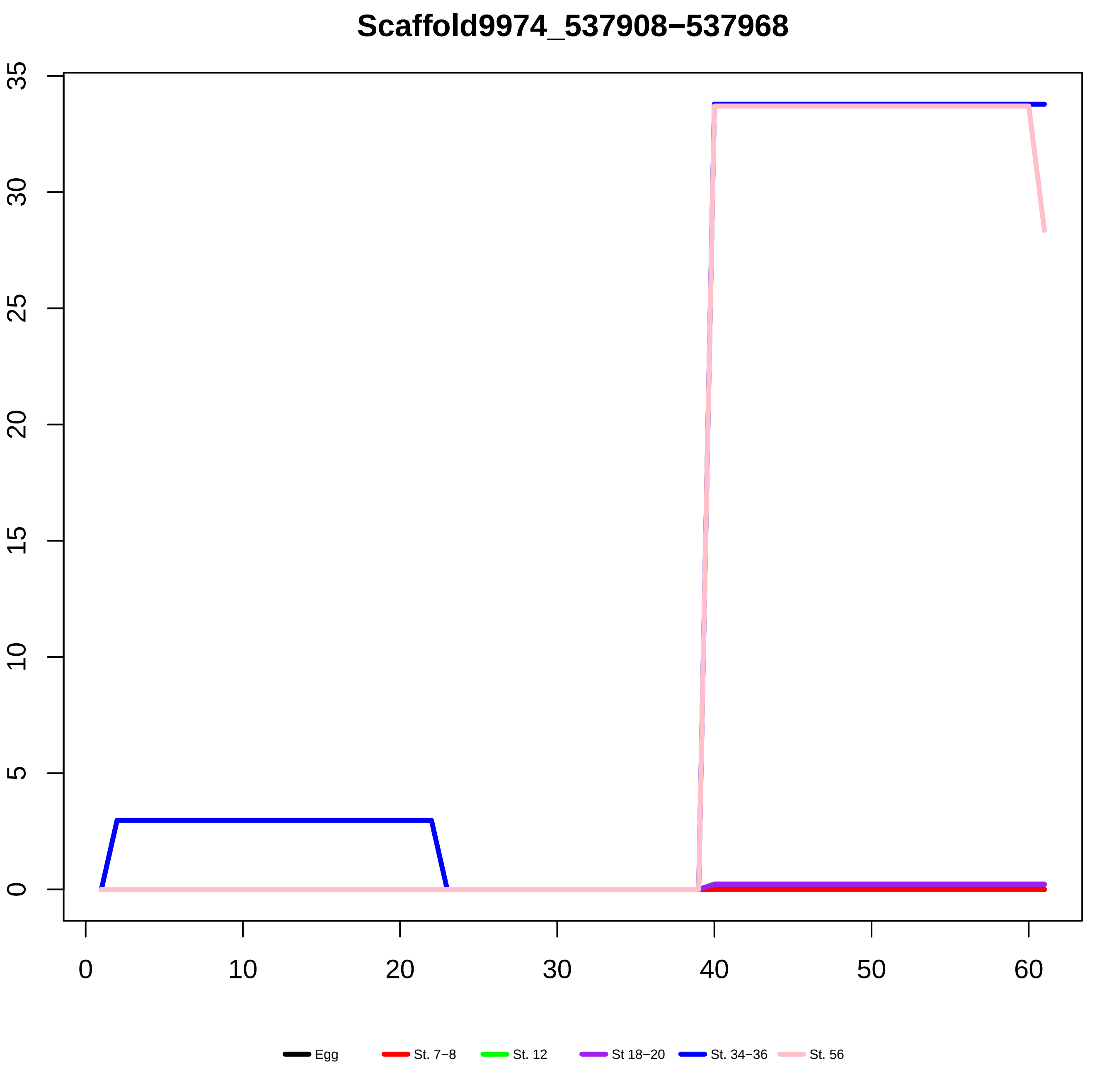
Scaffold9974_537908−537968
35
30
25
20
15
10
5
0
0
10
20
30
40
50
60
Egg
St. 7−8
St. 12
St 18−20
St. 34−36
St. 56

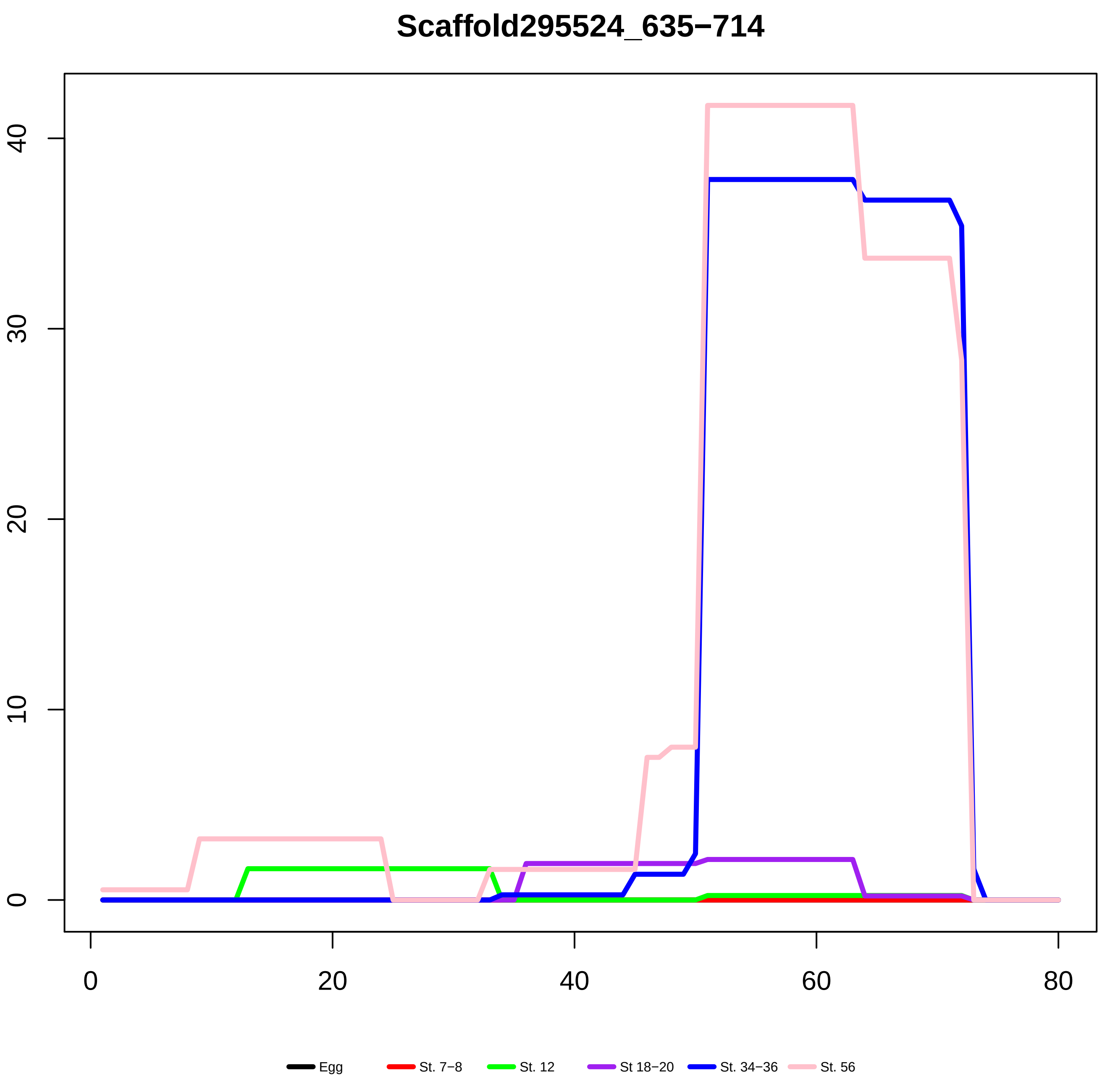
Scaffold295524_635−714
0
10
20
30
40
0
20
40
60
80
Egg
St. 7−8
St. 12
St 18−20
St. 34−36
St. 56

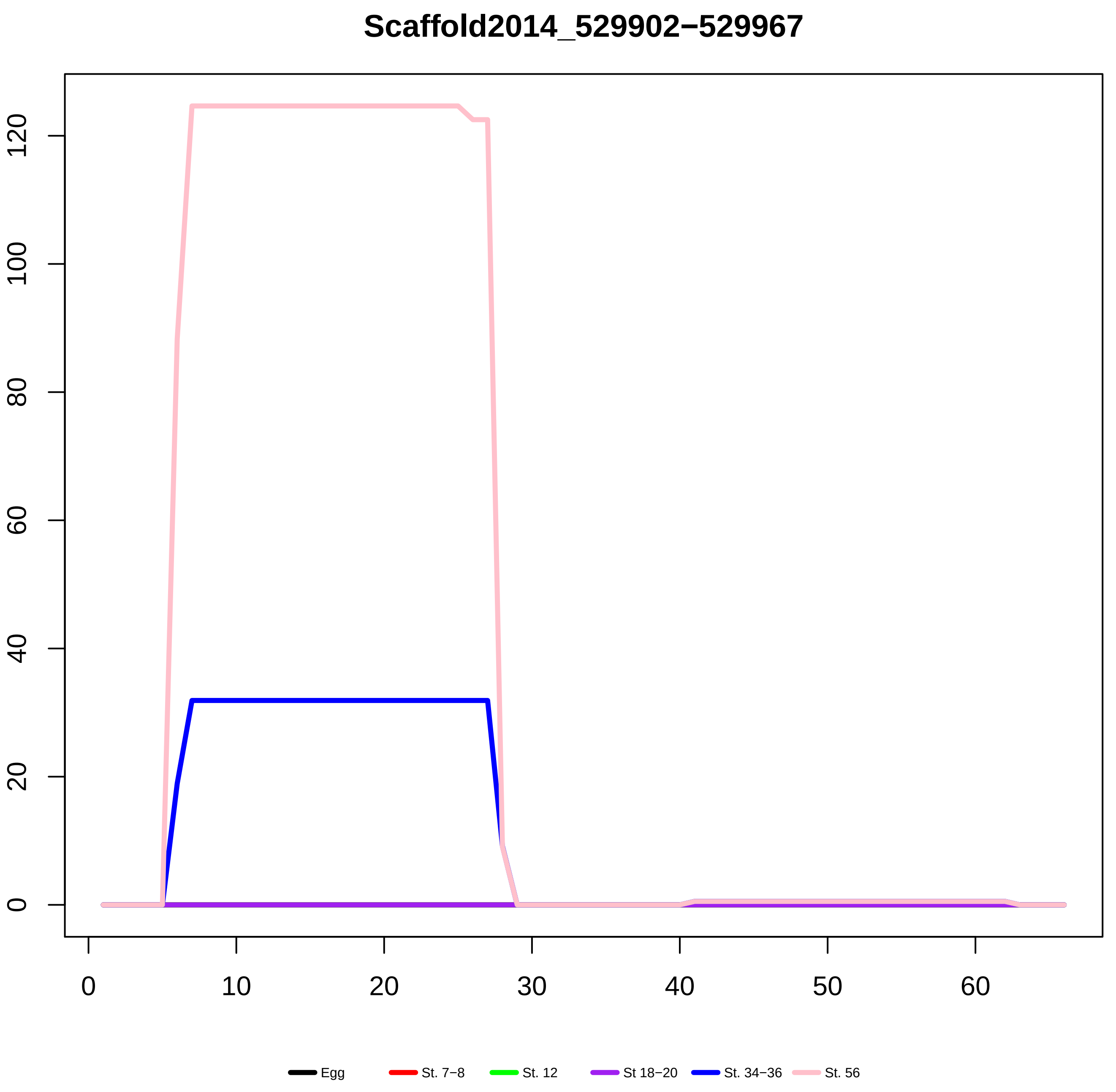

Scaffold2014_529902−529967
0
20
40
60
80
100
120
0
10
20
30
40
50
60
Egg
St. 7−8
St. 12
St 18−20
St. 34−36
St. 56

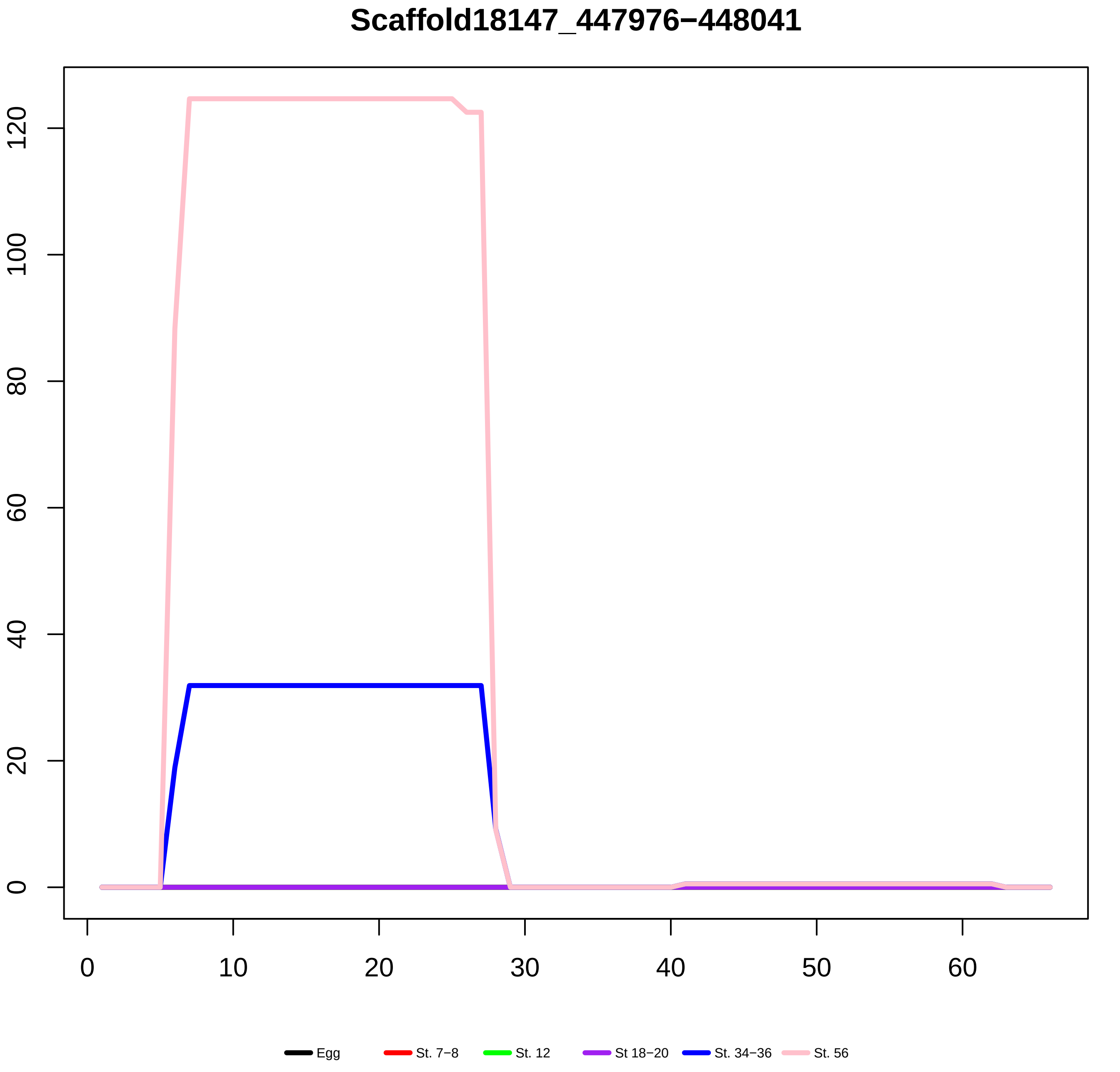

Scaffold18147_447976−448041
0
20
40
60
80
100
120
0
10
20
30
40
50
60
Egg
St. 7−8
St. 12
St 18−20
St. 34−36
St. 56

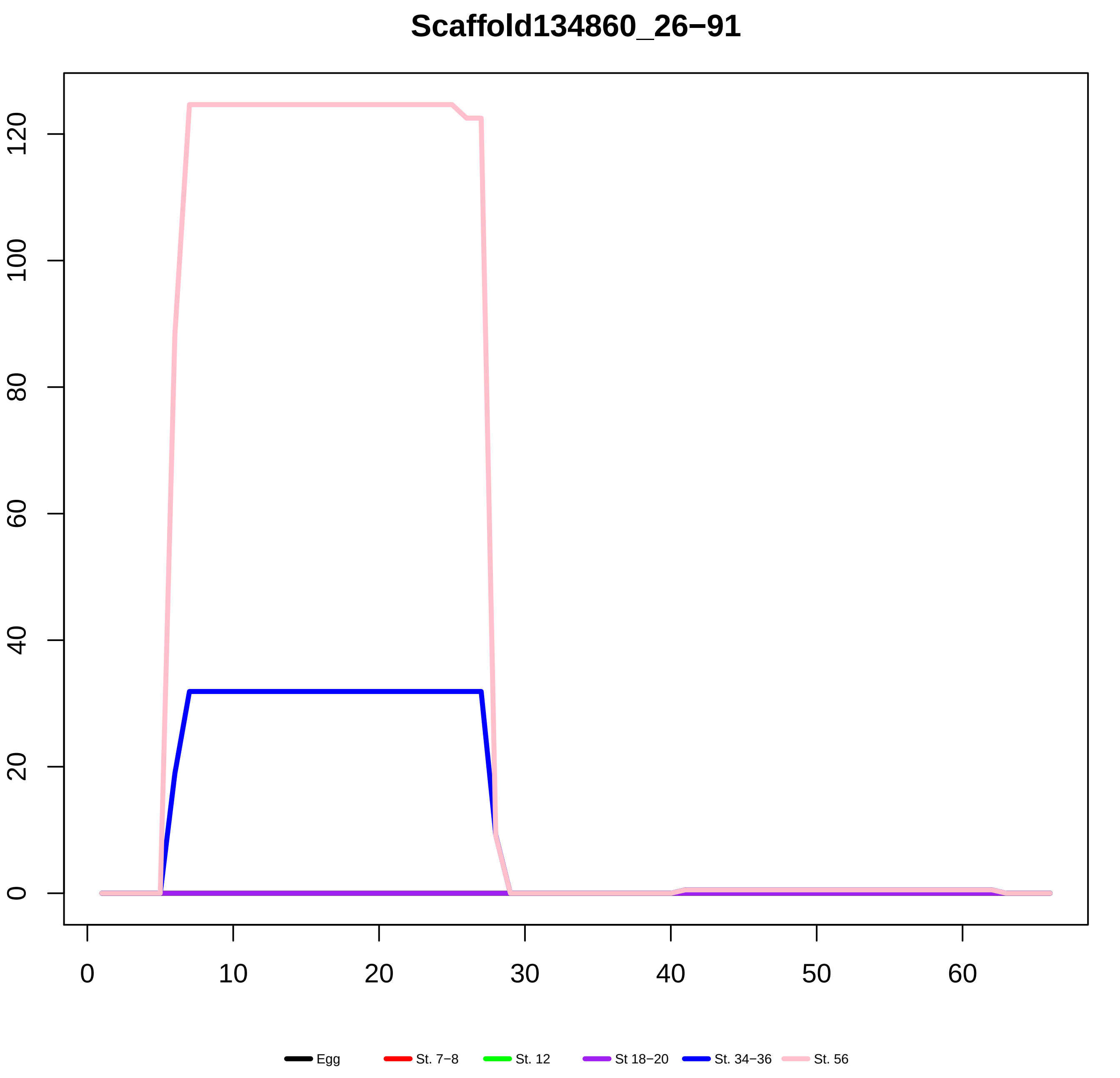
Scaffold134860_26−91
0
20
40
60
80
100
120
0
10
20
30
40
50
60
Egg
St. 7−8
St. 12
St 18−20
St. 34−36
St. 56

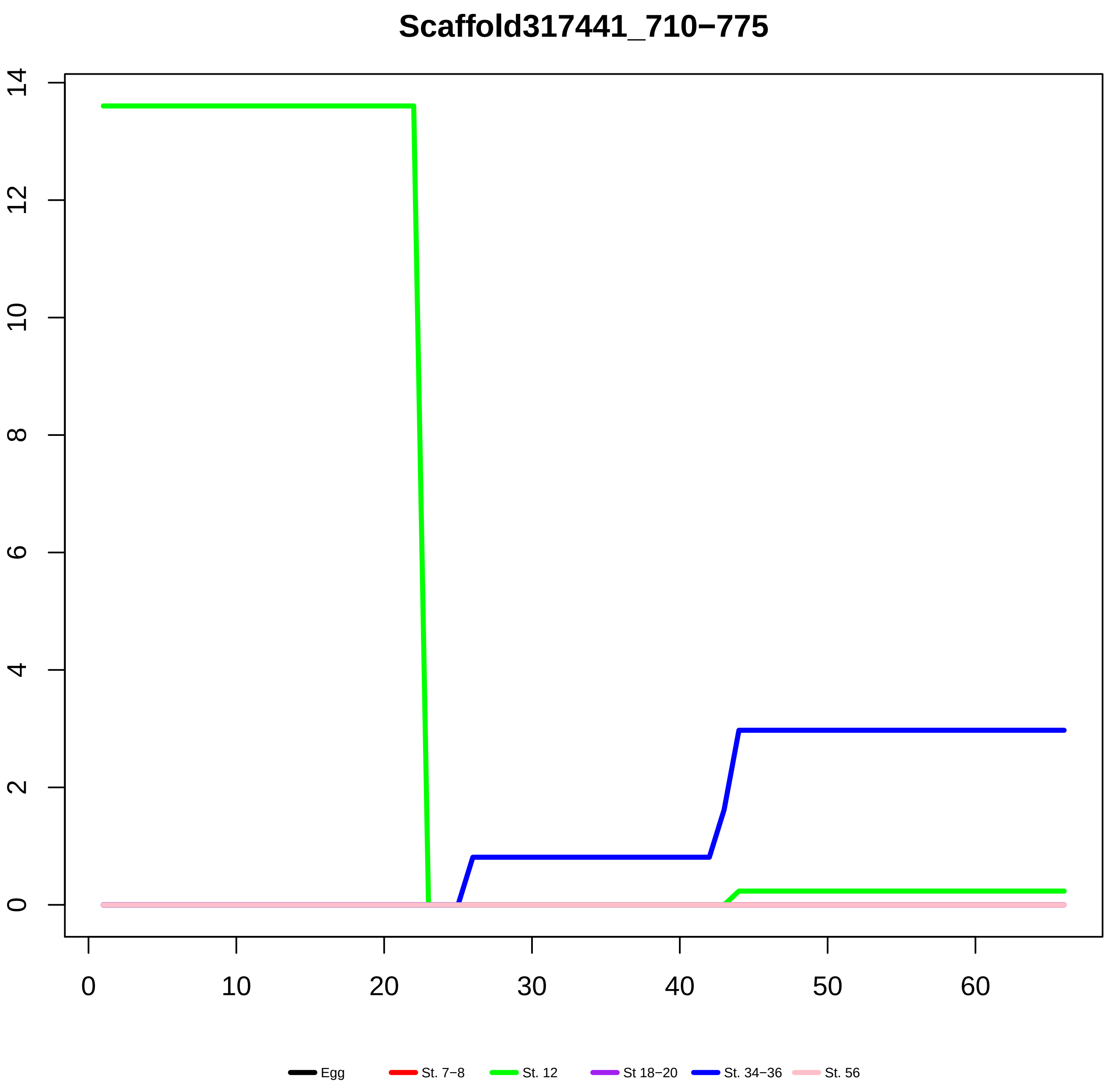

Scaffold317441_710−775
14
12
10
8
6
4
2
0
0
10
20
30
40
50
60
Egg
St. 7−8
St. 12
St 18−20
St. 34−36
St. 56

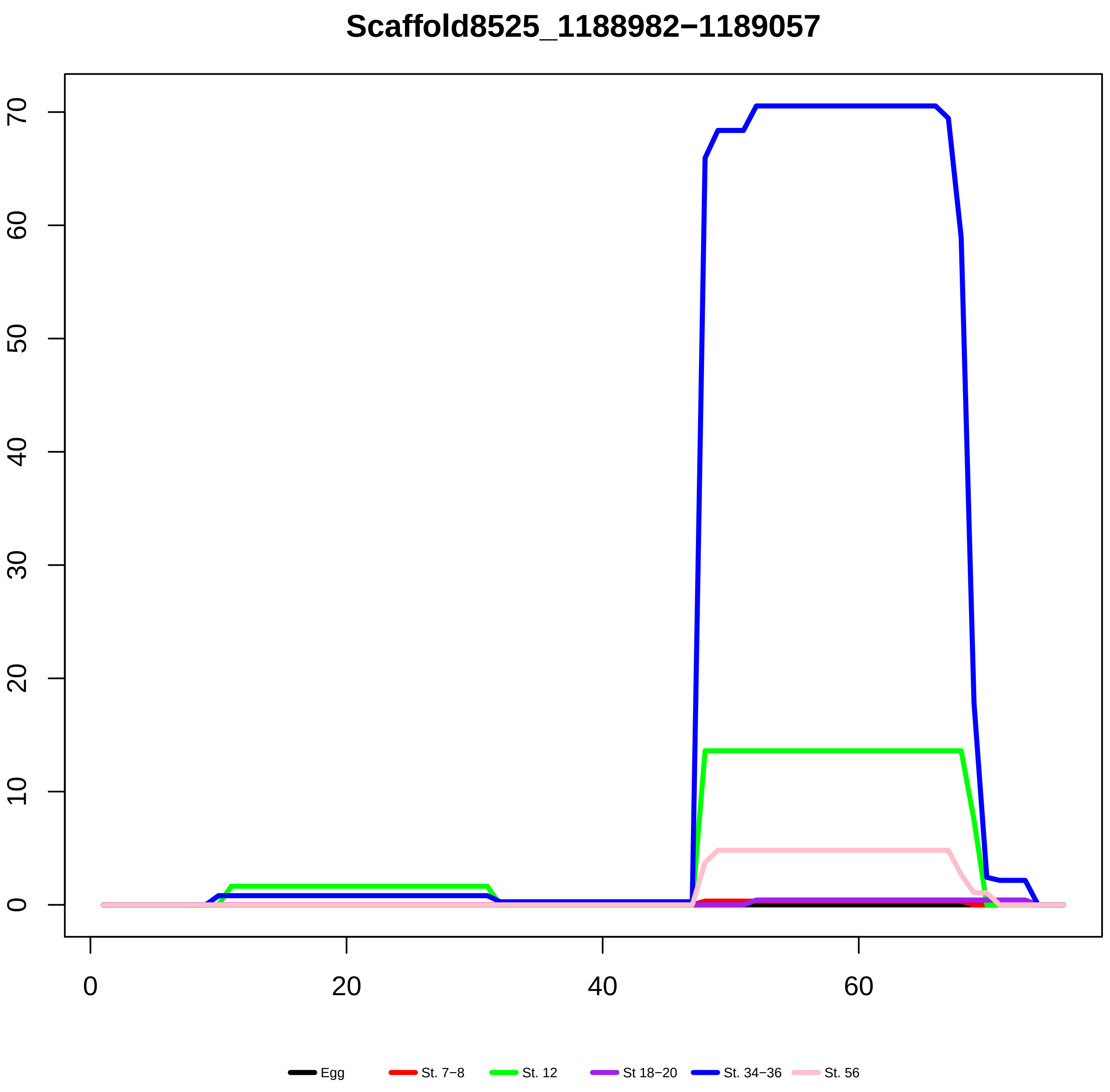

Scaffold8525_1188982−1189057
0
10
20
30
40
50
60
70
0
20
40
60
Egg
St. 7−8
St. 12
St 18−20
St. 34−36
St. 56

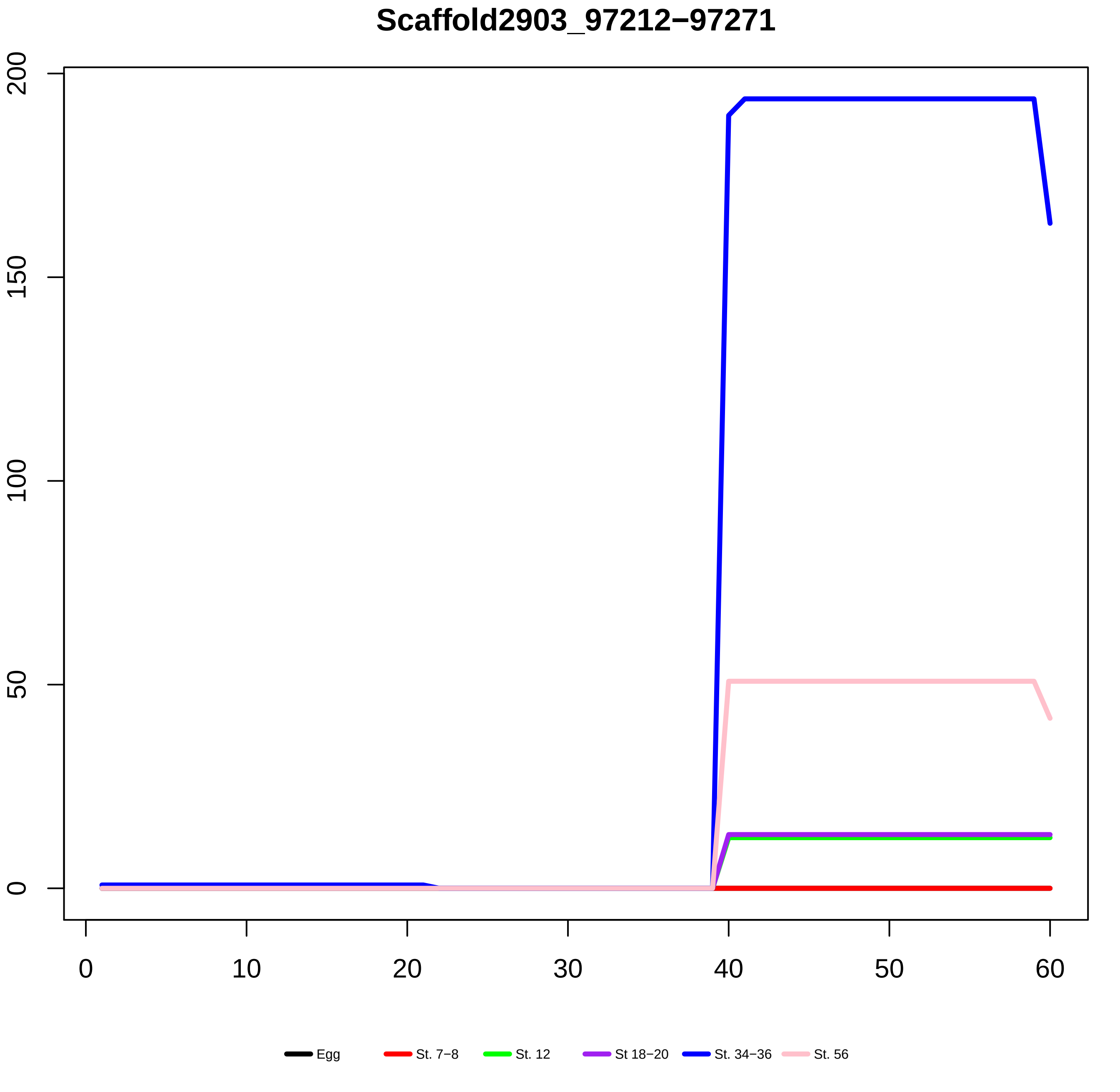
Scaffold2903_97212−97271
200
150
100
50
0
0
10
20
30
40
50
60
Egg
St. 7−8
St. 12
St 18−20
St. 34−36
St. 56

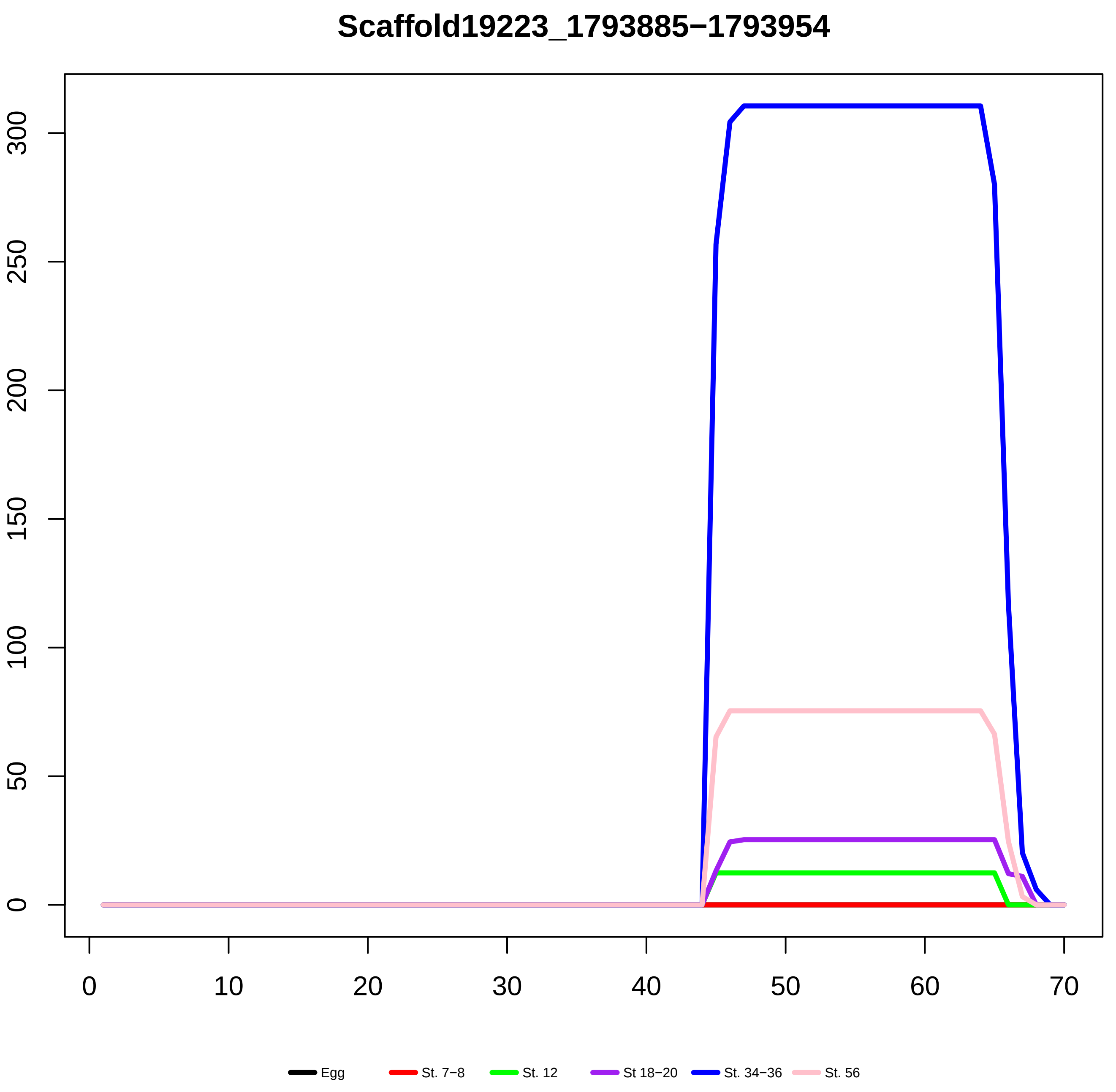

Scaffold19223_1793885−1793954
0
50
100
150
200
250
300
0
10
20
30
40
50
60
70
Egg
St. 7−8
St. 12
St 18−20
St. 34−36
St. 56

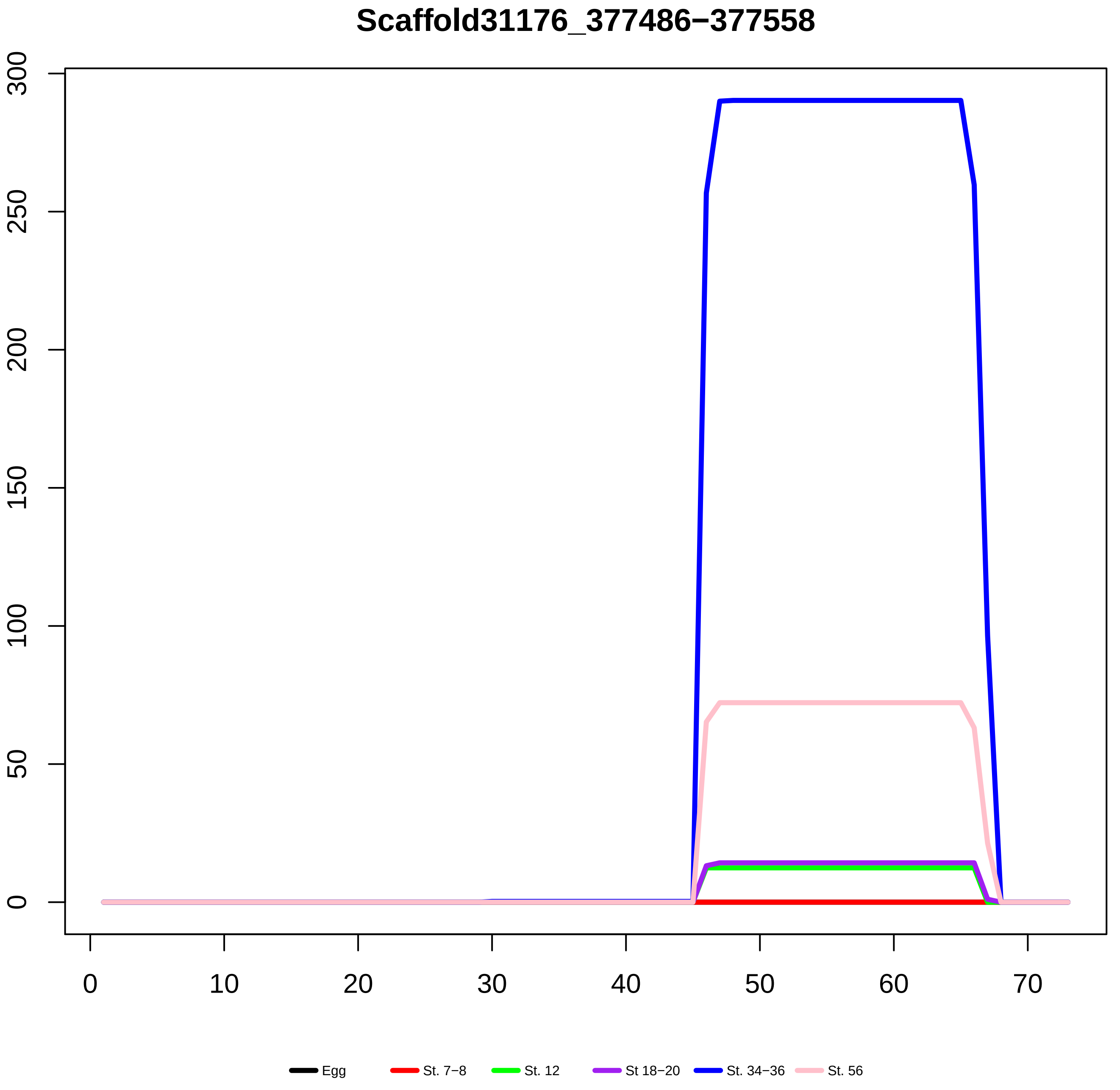

Scaffold31176_377486−377558
300
250
200
150
100
50
0
0
10
20
30
40
50
60
70
Egg
St. 7−8
St. 12
St 18−20
St. 34−36
St. 56

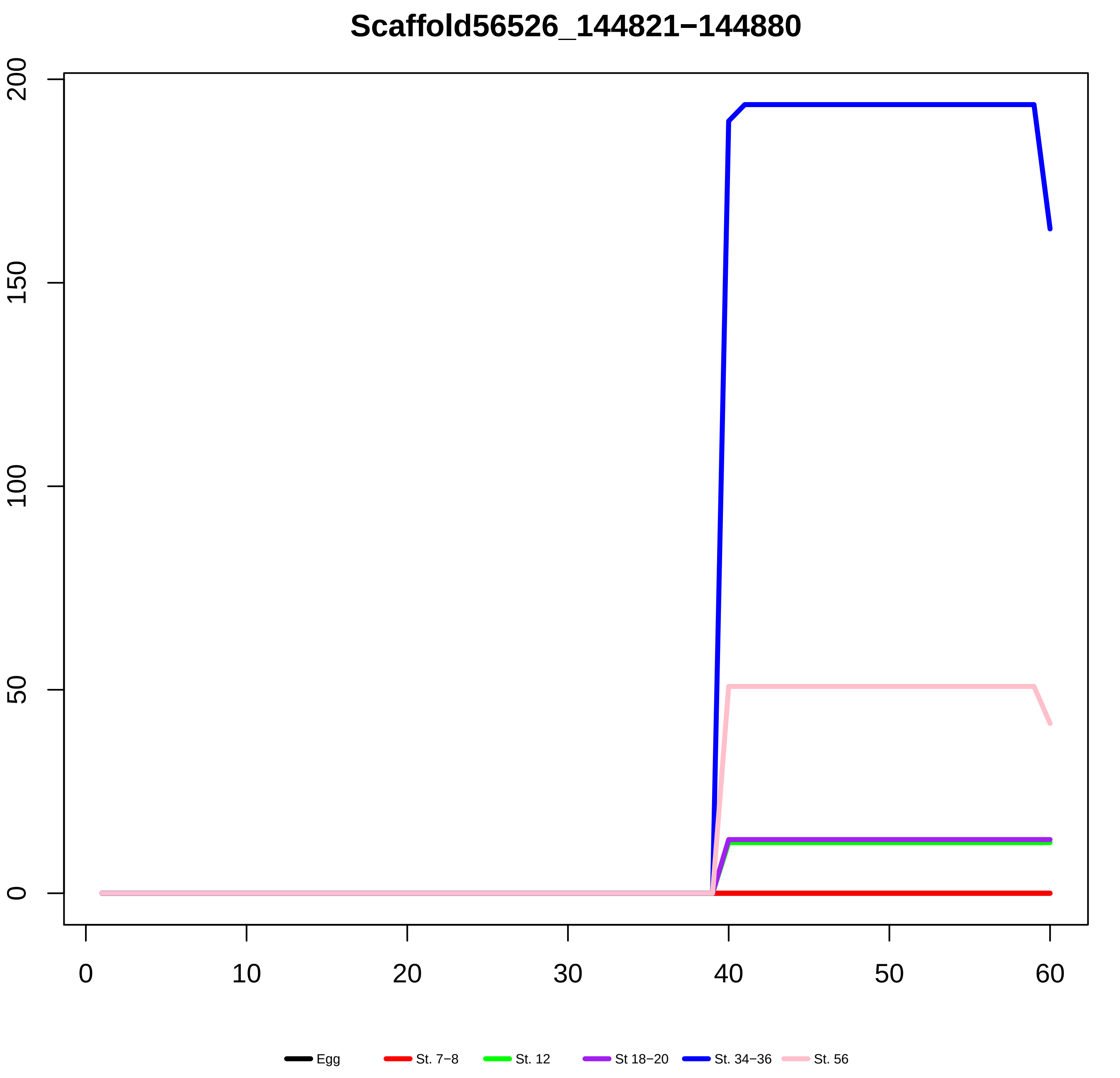

Scaffold56526_144821−144880
200
150
100
50
0
0
10
20
30
40
50
60
Egg
St. 7−8
St. 12
St 18−20
St. 34−36
St. 56

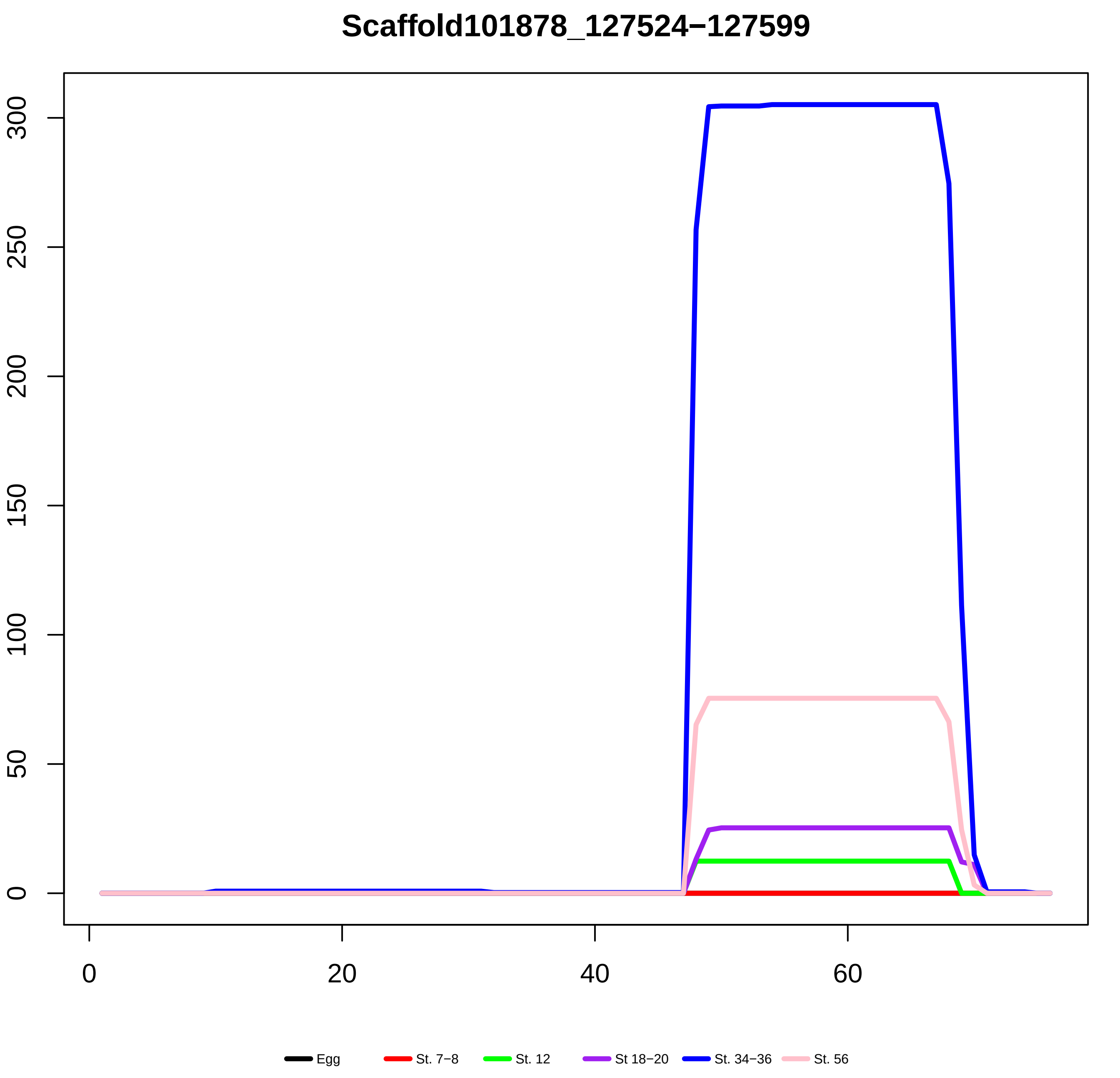

Scaffold101878_127524−127599
0
50
100
150
200
250
300
0
20
40
60
Egg
St. 7−8
St. 12
St 18−20
St. 34−36
St. 56

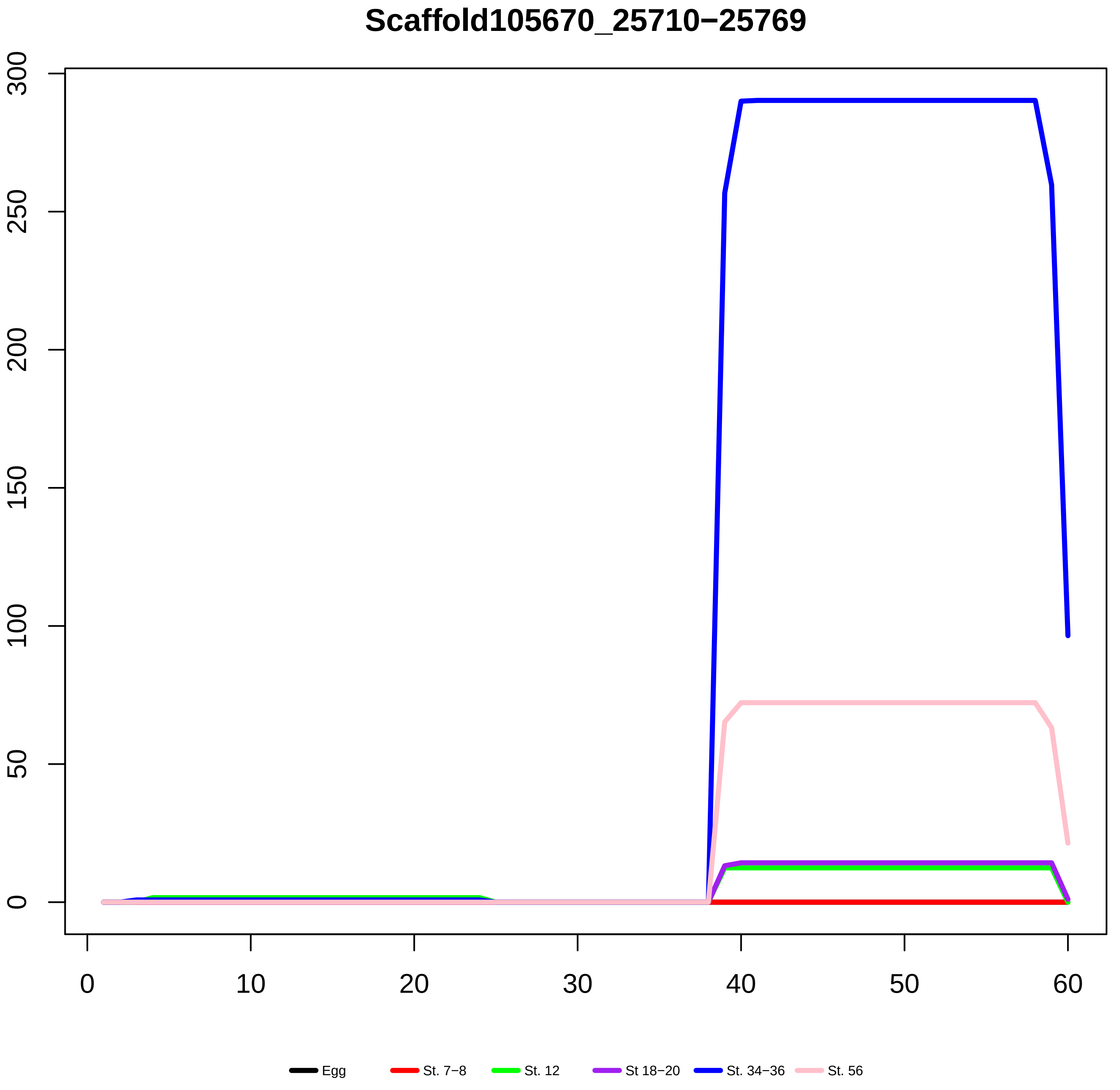

Scaffold105670_25710−25769
0
50
100
150
200
250
300
0
10
20
30
40
50
60
Egg
St. 7−8
St. 12
St 18−20
St. 34−36
St. 56

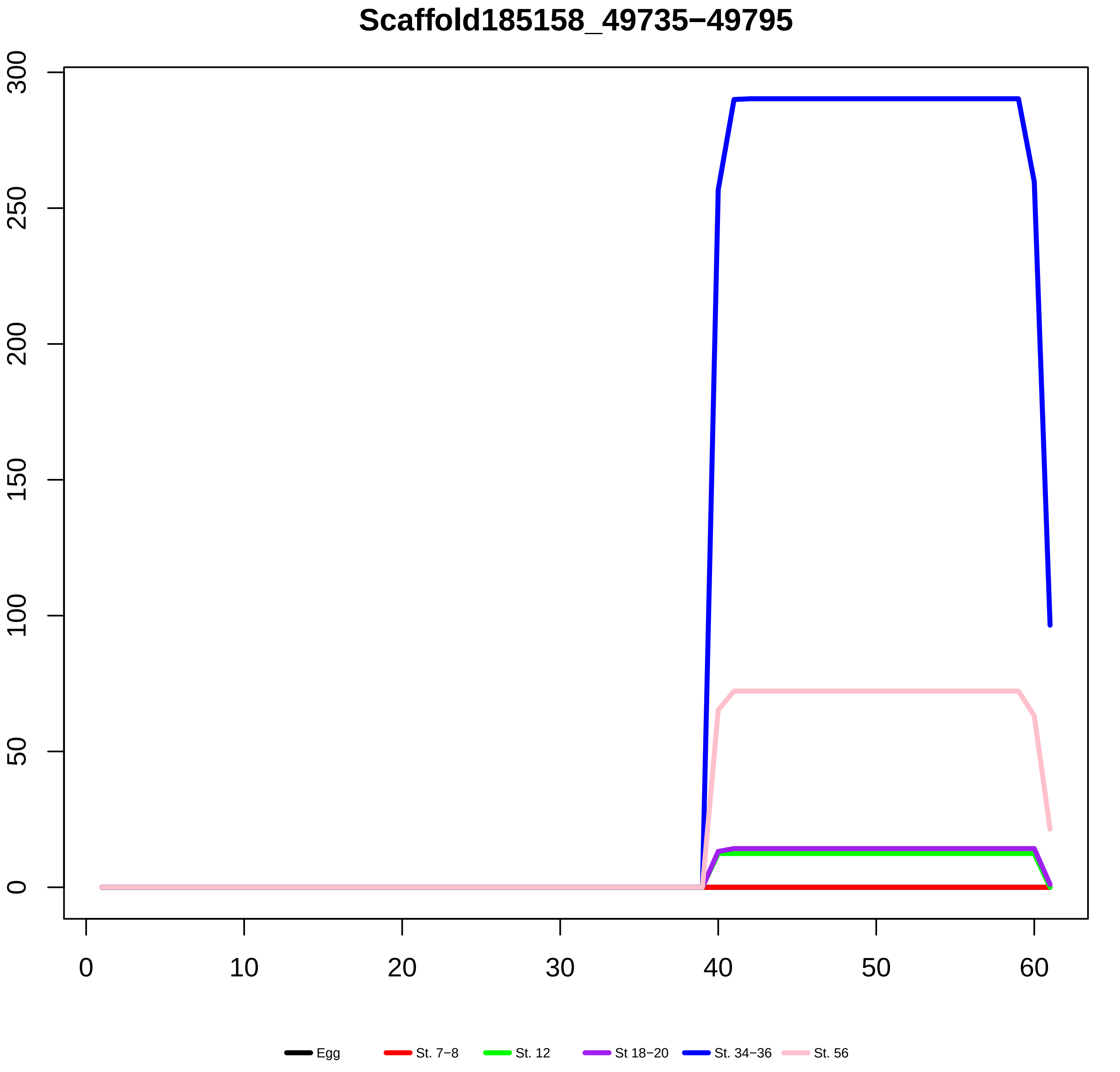
Scaffold185158_49735−49795
0
50
100
150
200
250
300
0
10
20
30
40
50
60
Egg
St. 7−8
St. 12
St 18−20
St. 34−36
St. 56

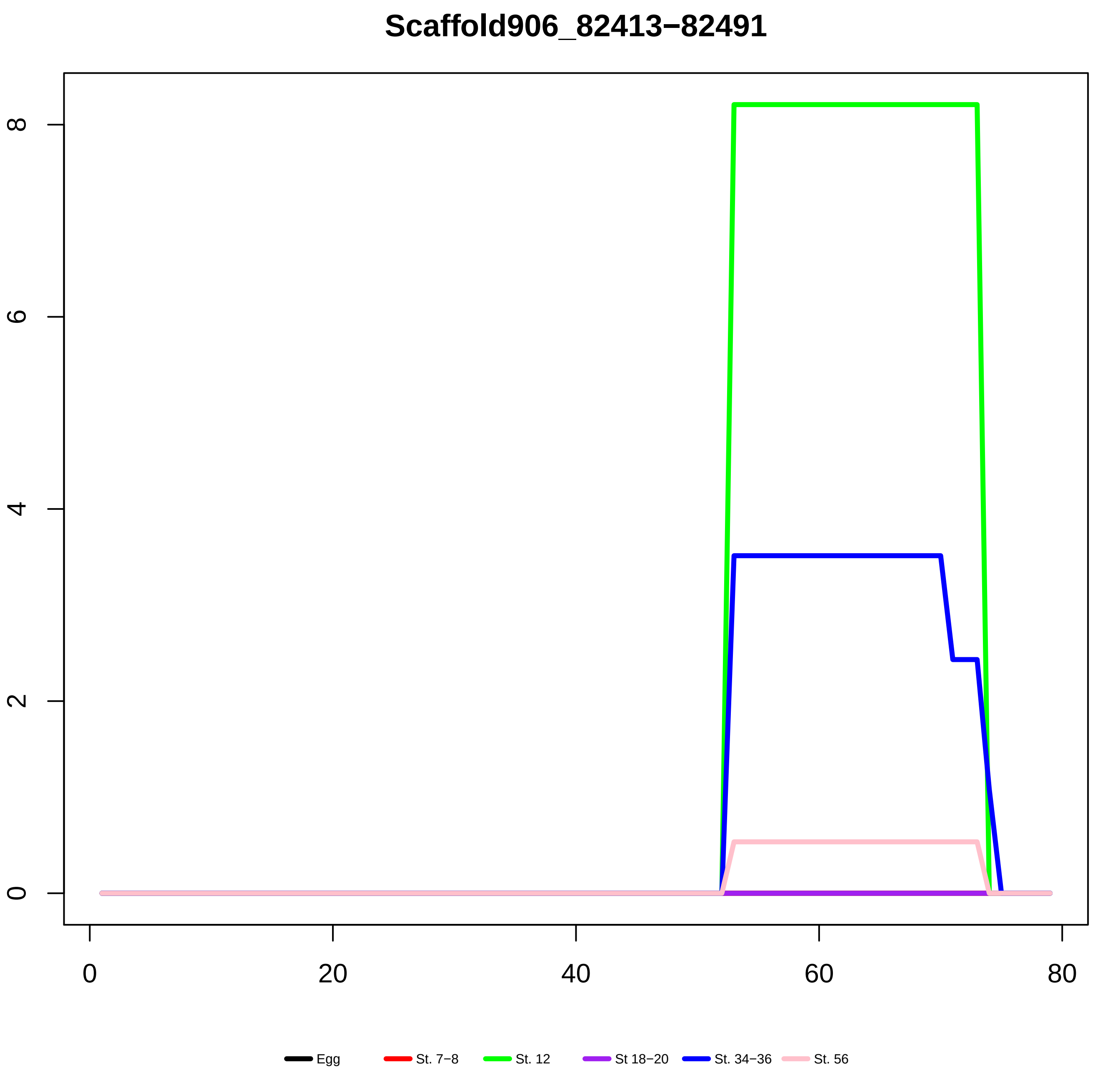
Scaffold906_82413−82491
0
2
4
6
8
0
20
40
60
80
Egg
St. 7−8
St. 12
St 18−20
St. 34−36
St. 56

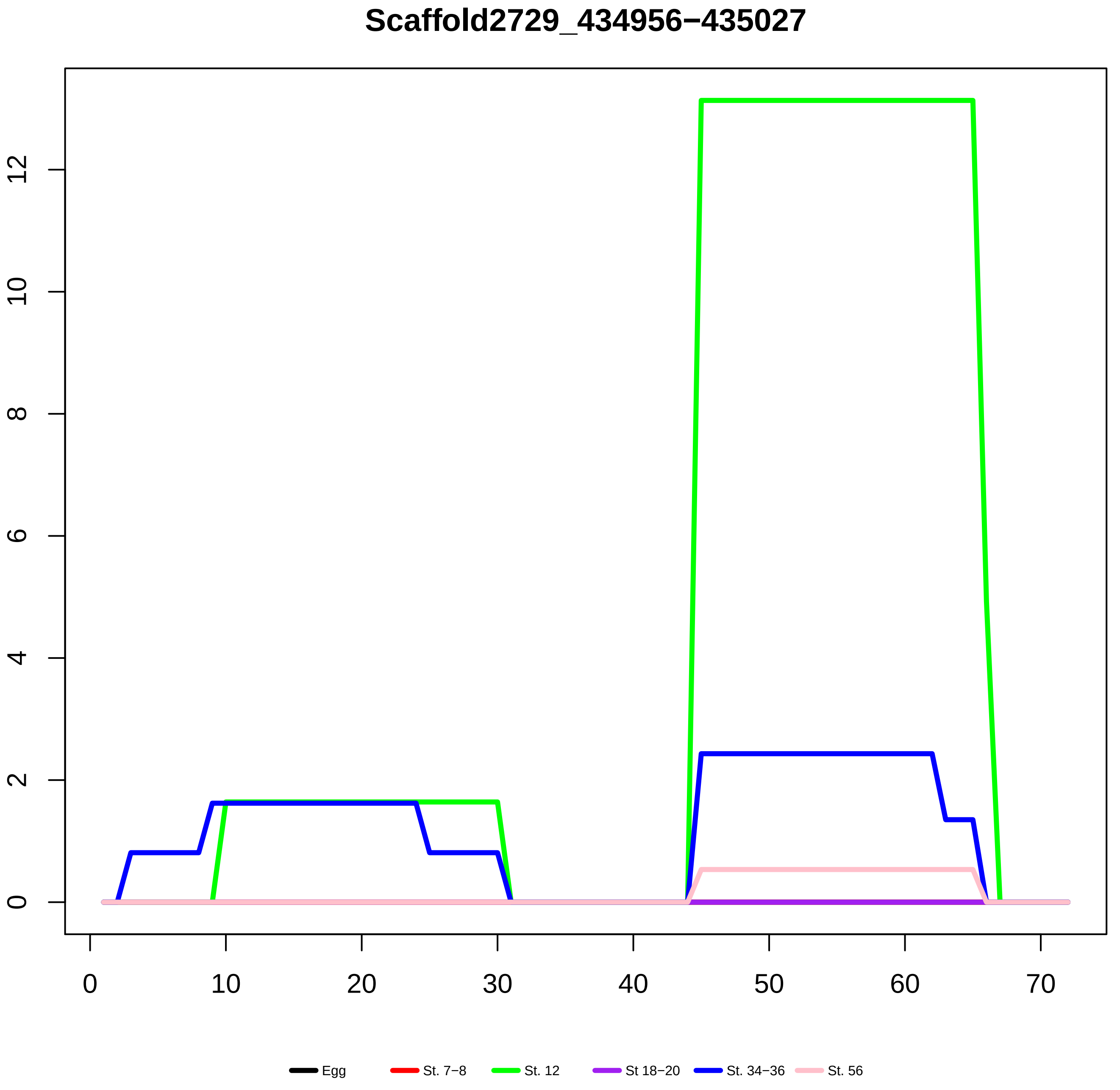

Scaffold2729_434956−435027
0
2
4
6
8
10
12
0
10
20
30
40
50
60
70
Egg
St. 7−8
St. 12
St 18−20
St. 34−36
St. 56

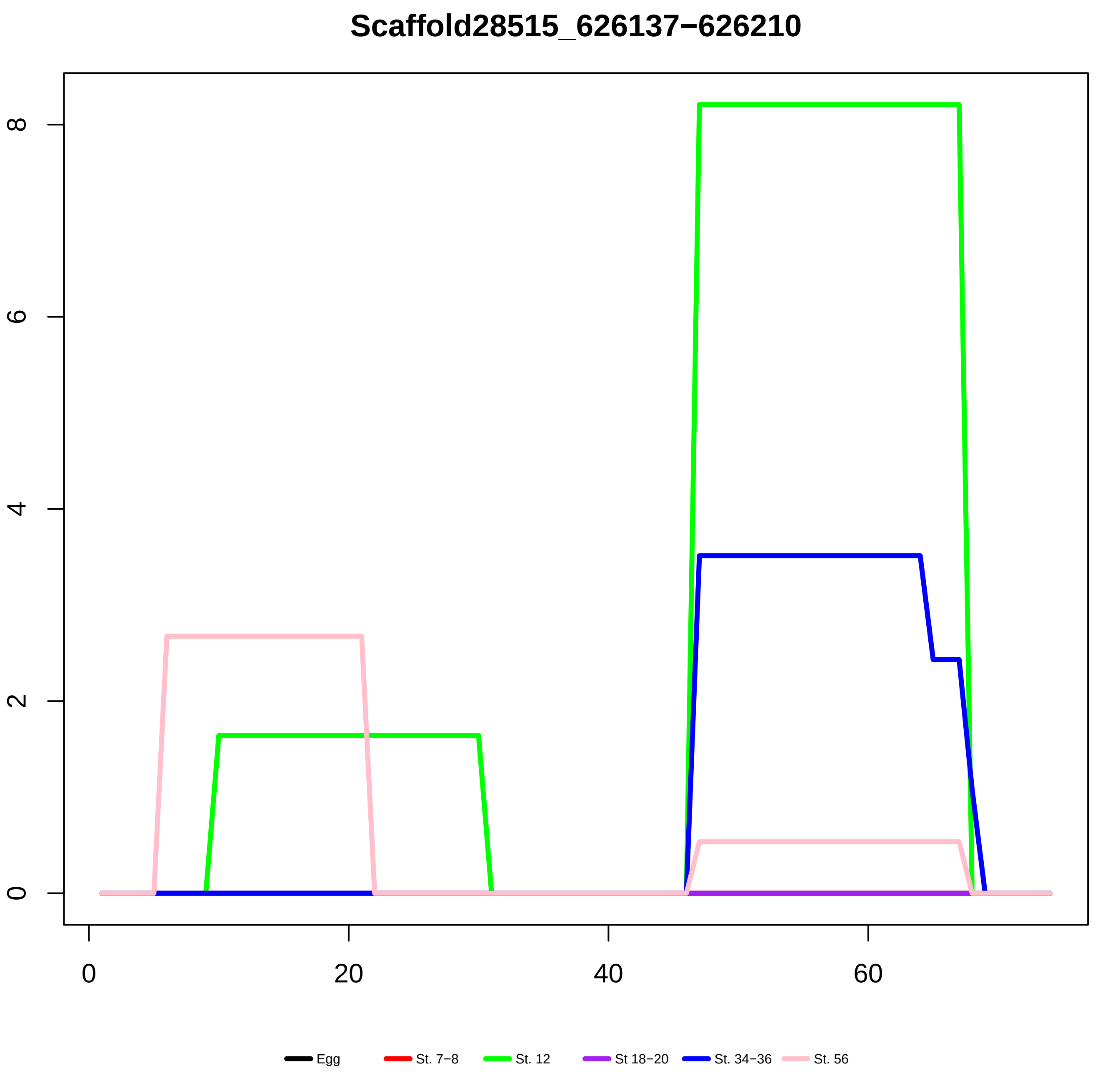
Scaffold28515_626137−626210
0
2
4
6
8
0
20
40
60
Egg
St. 7−8
St. 12
St 18−20
St. 34−36
St. 56

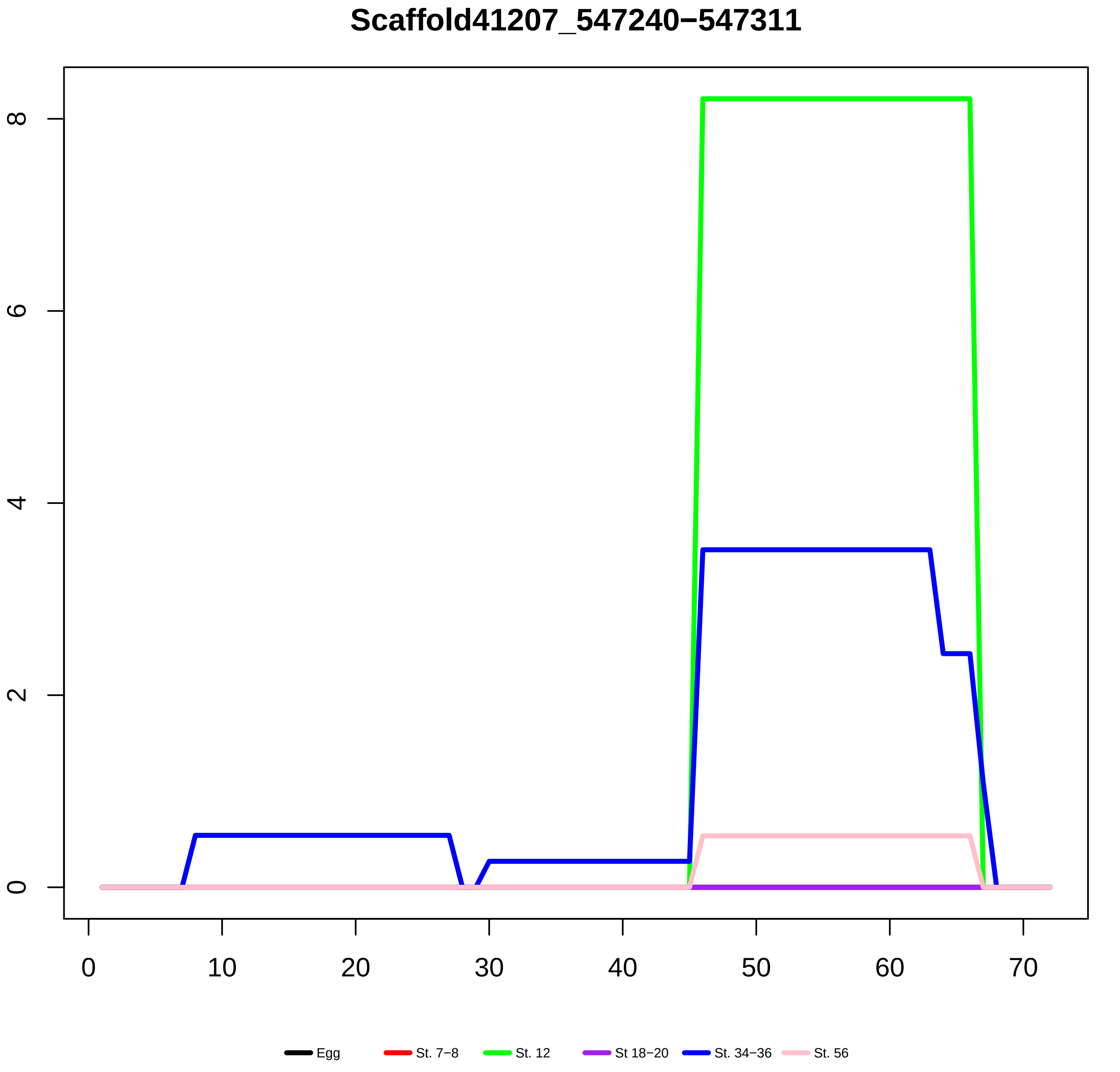
Scaffold41207_547240−547311
0
2
4
6
8
0
10
20
30
40
50
60
70
Egg
St. 7−8
St. 12
St 18−20
St. 34−36
St. 56

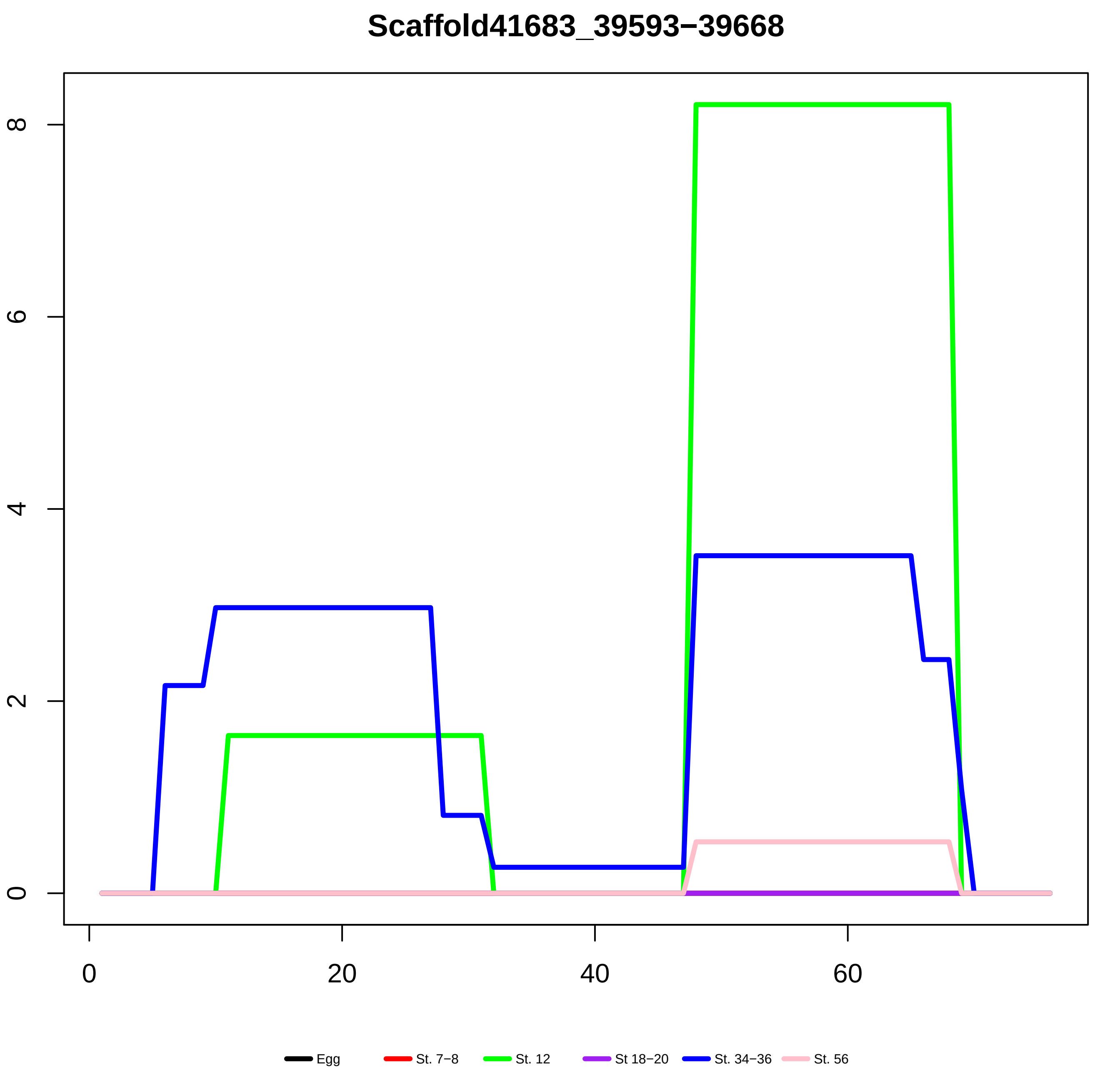

Scaffold41683_39593−39668
0
2
4
6
8
0
20
40
60
Egg
St. 7−8
St. 12
St 18−20
St. 34−36
St. 56

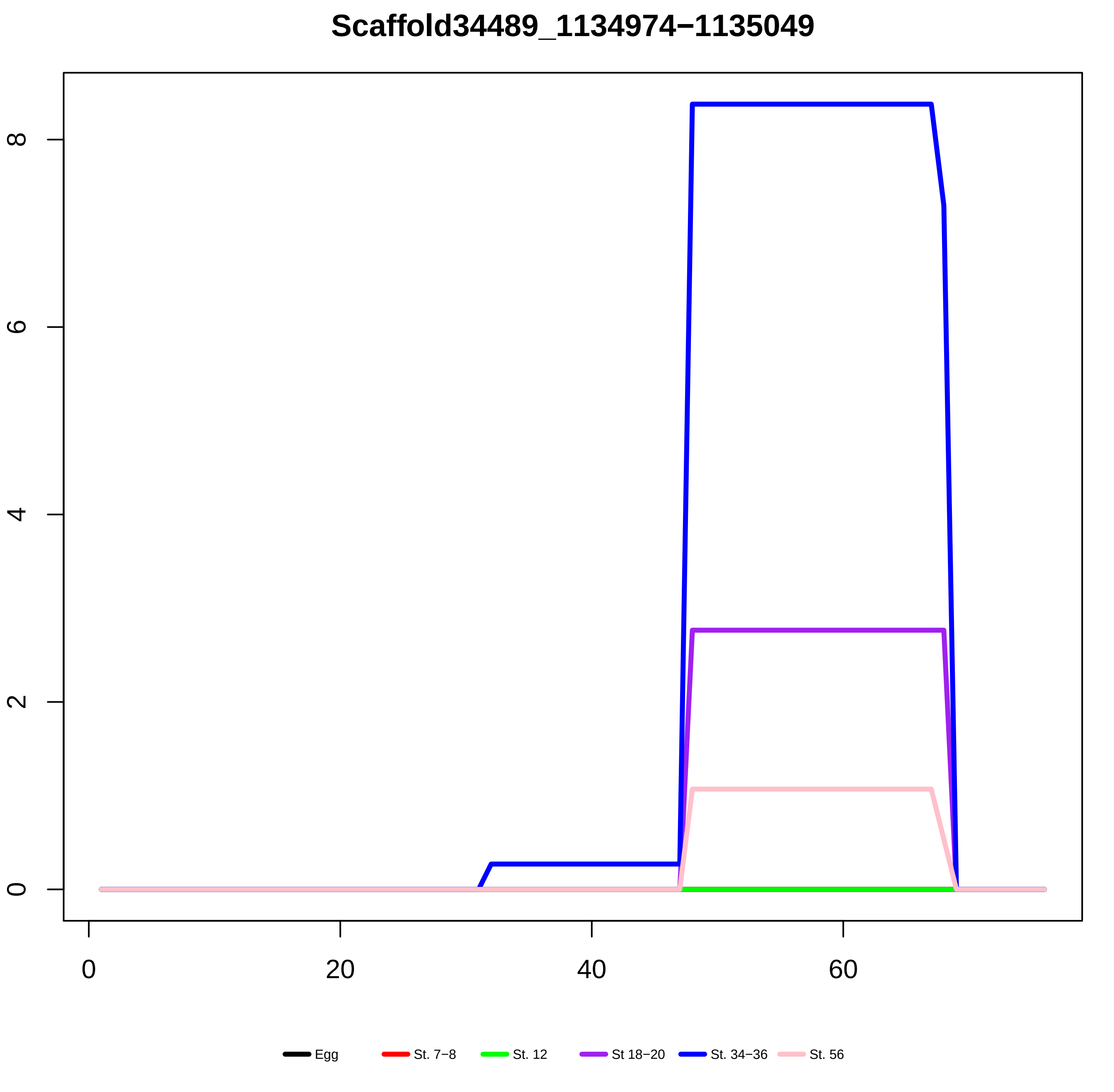

Scaffold34489_1134974−1135049
0
2
4
6
8
0
20
40
60
Egg
St. 7−8
St. 12
St 18−20
St. 34−36
St. 56

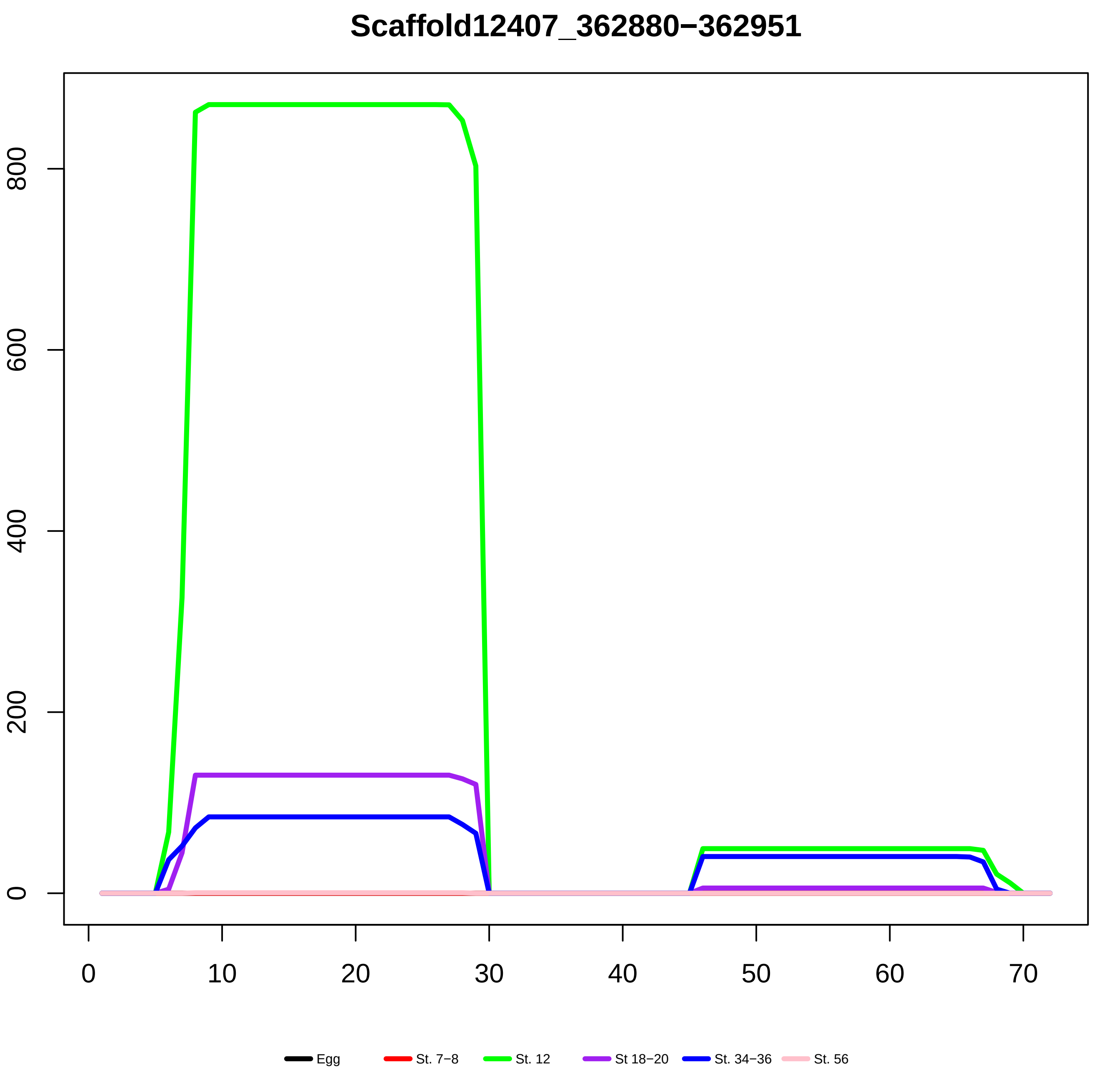
Scaffold12407_362880−362951
0
200
400
600
800
0
10
20
30
40
50
60
70
Egg
St. 7−8
St. 12
St 18−20
St. 34−36
St. 56

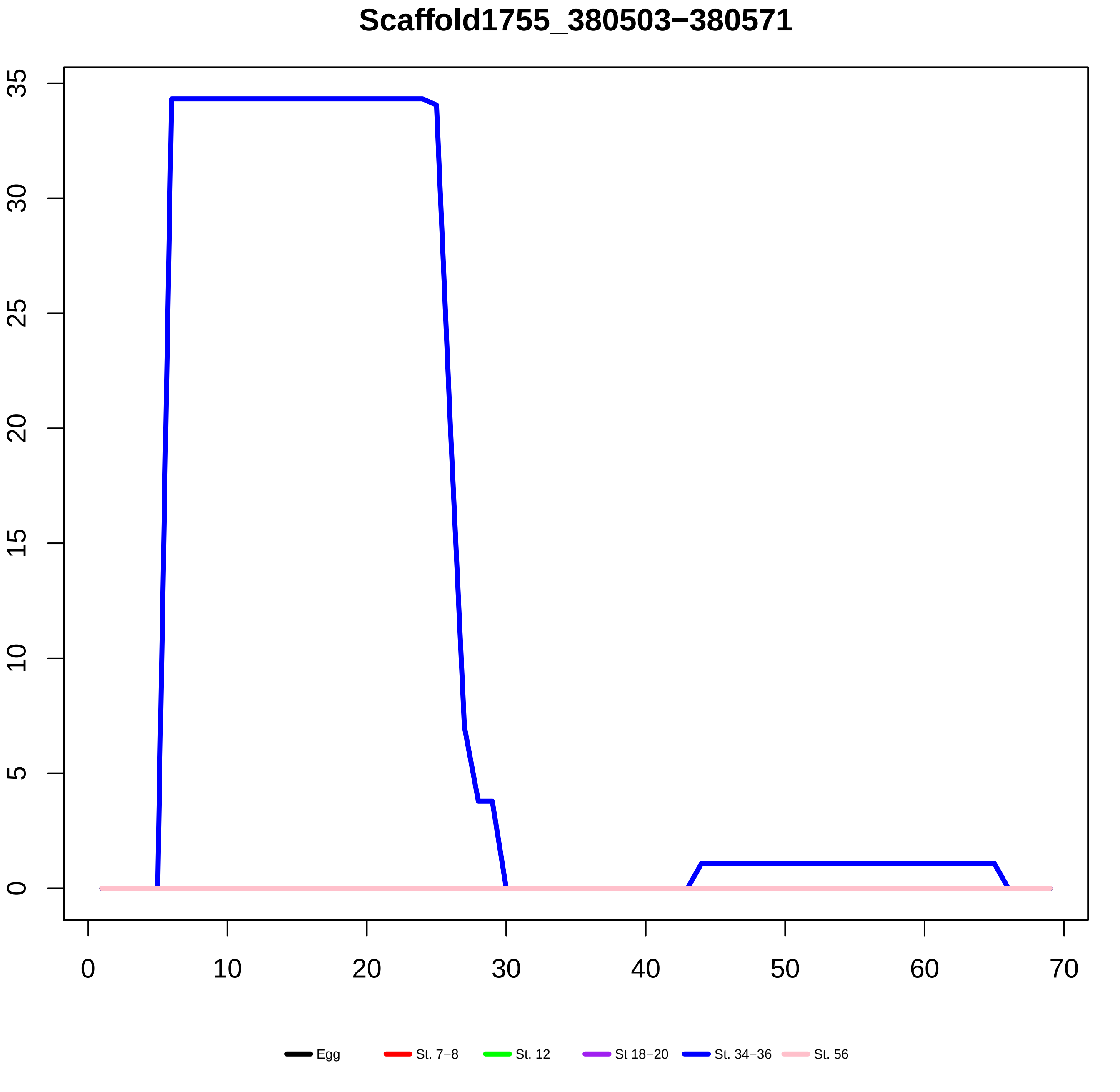

Scaffold1755_380503−380571
0
5
10
15
20
25
30
35
0
10
20
30
40
50
60
70
Egg
St. 7−8
St. 12
St 18−20
St. 34−36
St. 56

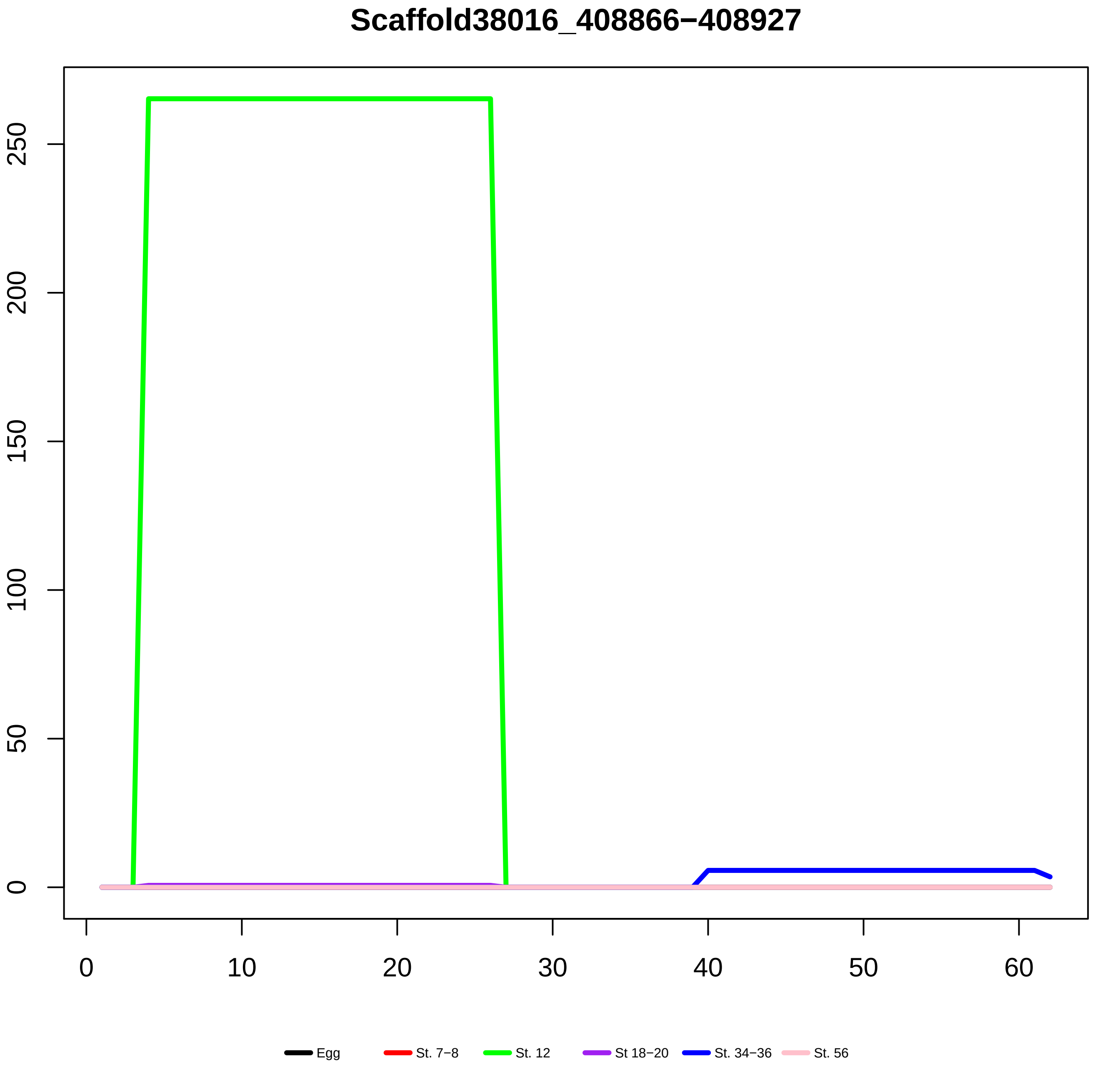

Scaffold38016_408866−408927
0
50
100
150
200
250
0
10
20
30
40
50
60
Egg
St. 7−8
St. 12
St 18−20
St. 34−36
St. 56

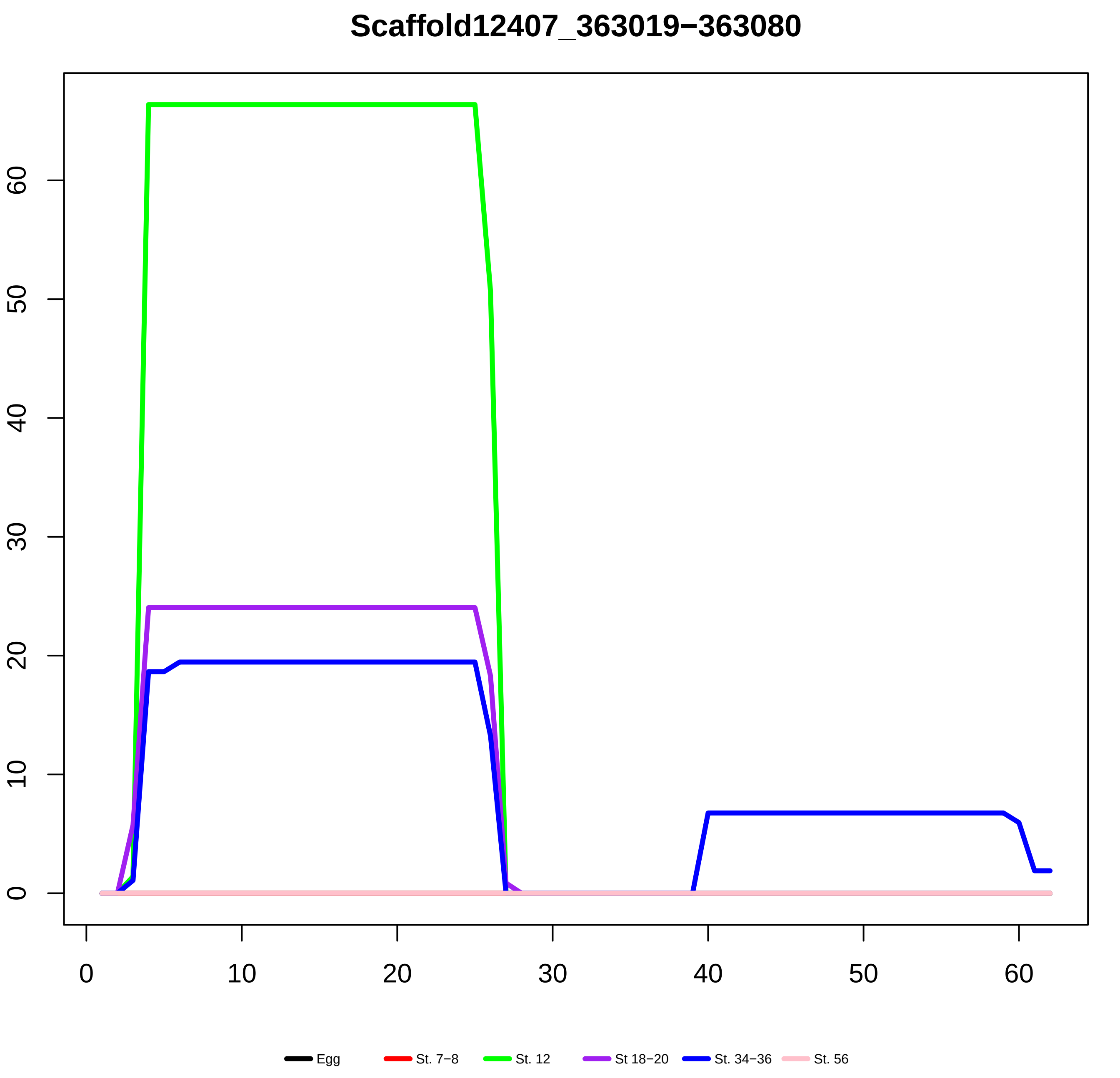

Scaffold12407_363019−363080
0
10
20
30
40
50
60
0
10
20
30
40
50
60
Egg
St. 7−8
St. 12
St 18−20
St. 34−36
St. 56

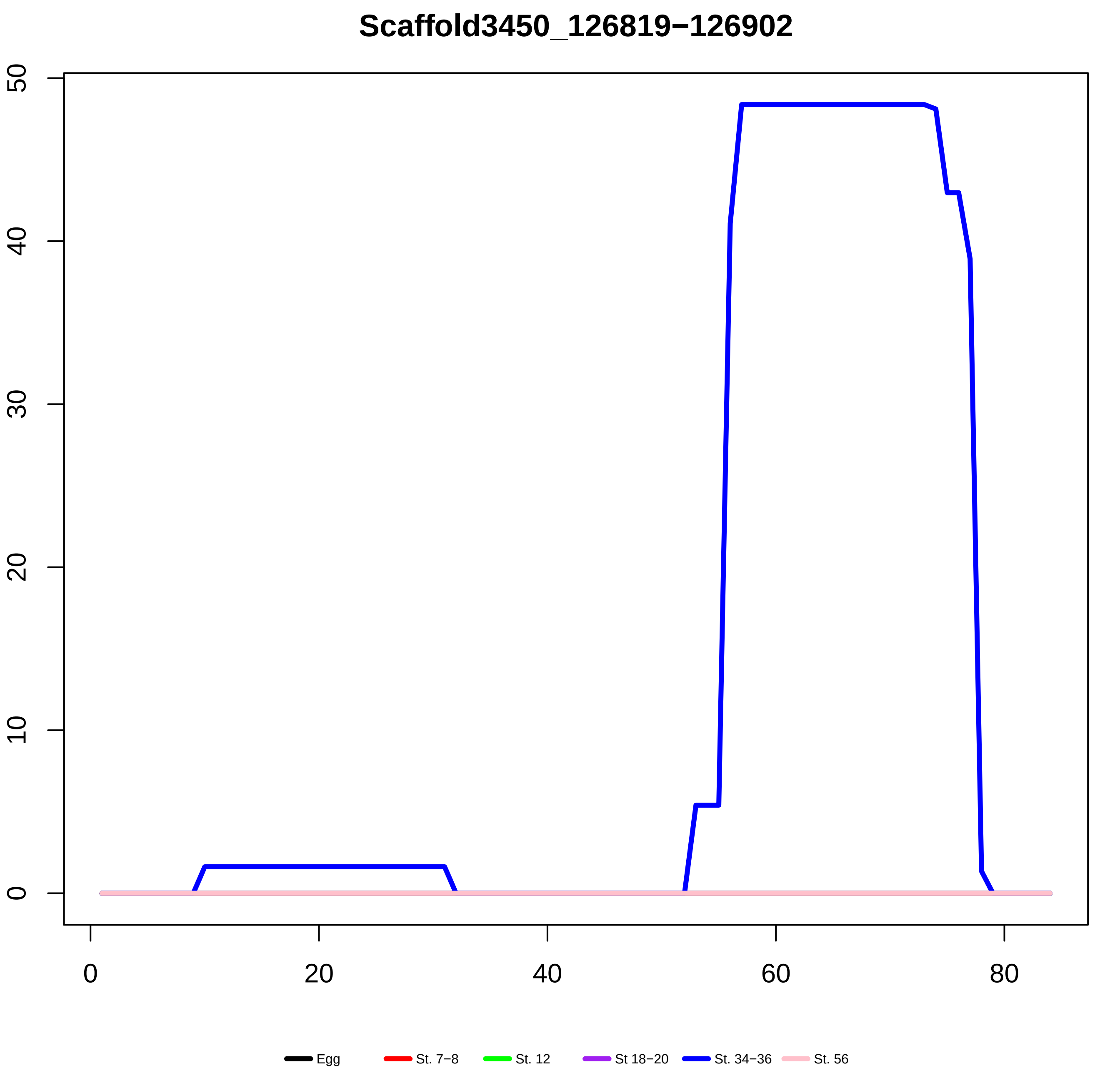

Scaffold3450_126819−126902
0
10
20
30
40
50
0
20
40
60
80
Egg
St. 7−8
St. 12
St 18−20
St. 34−36
St. 56

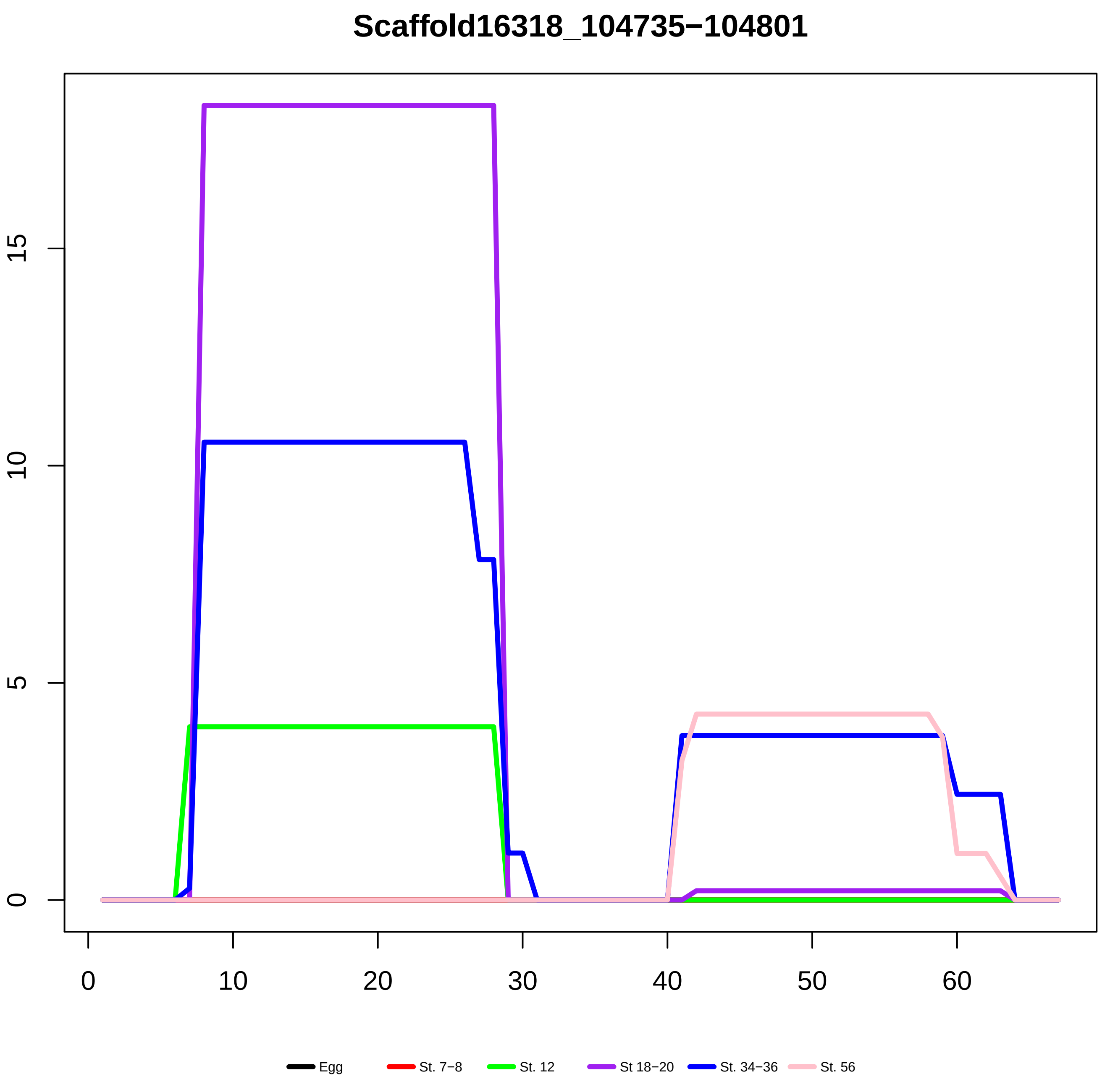

Scaffold16318_104735−104801
0
5
10
15
0
10
20
30
40
50
60
Egg
St. 7−8
St. 12
St 18−20
St. 34−36
St. 56

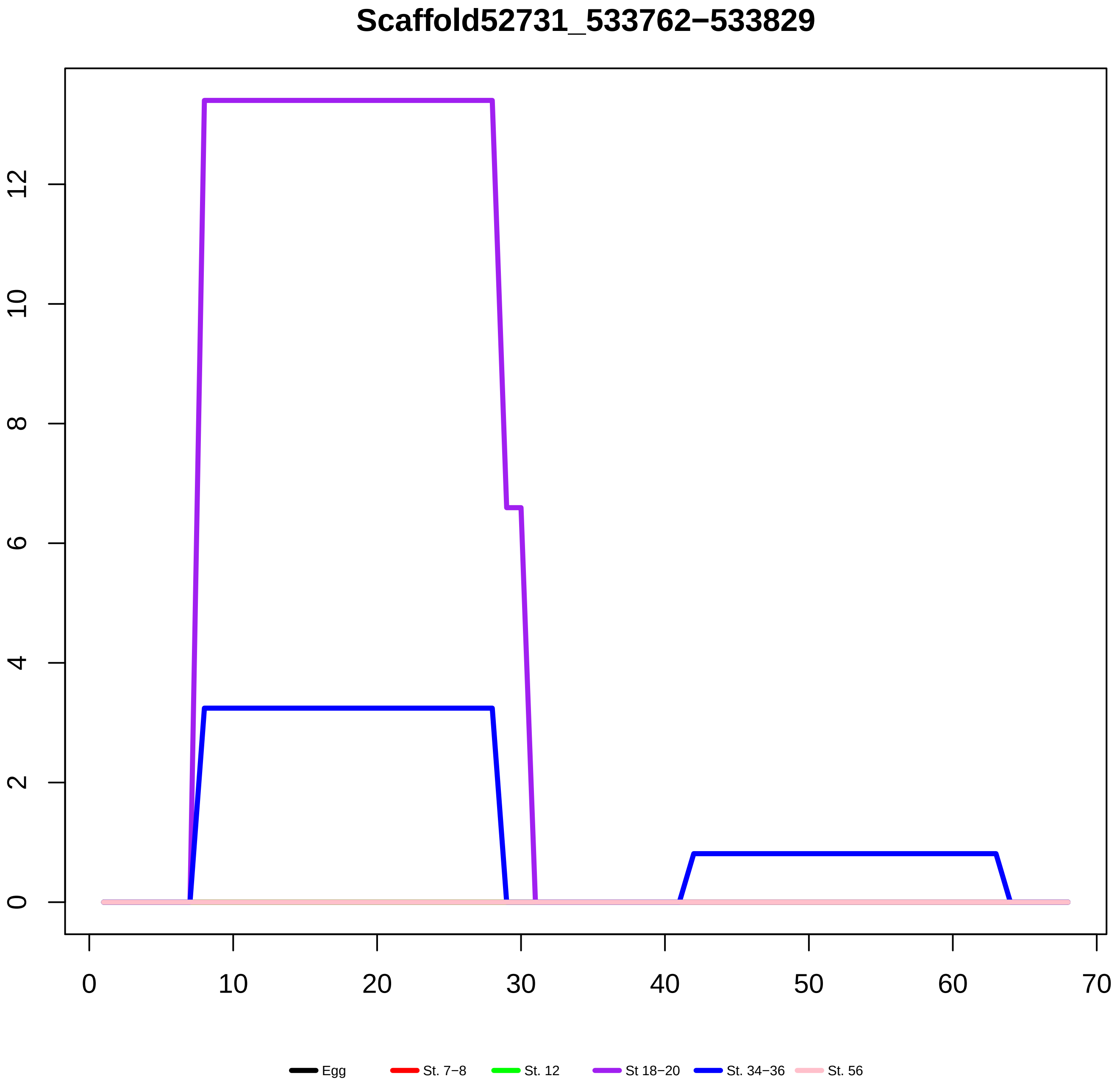

Scaffold52731_533762−533829
0
2
4
6
8
10
12
0
10
20
30
40
50
60
70
Egg
St. 7−8
St. 12
St 18−20
St. 34−36
St. 56

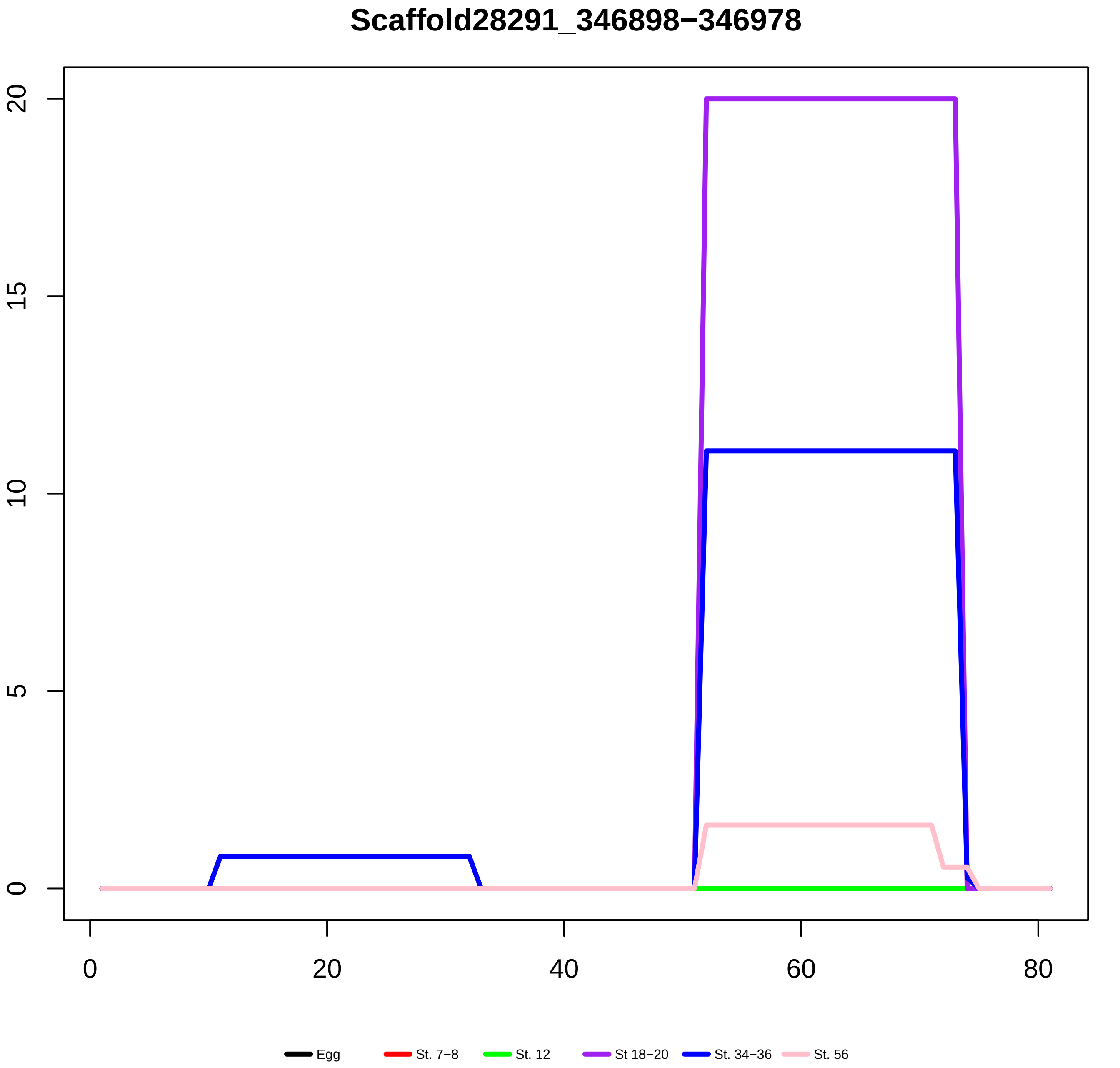

Scaffold28291_346898−346978
0
5
10
15
20
0
20
40
60
80
Egg
St. 7−8
St. 12
St 18−20
St. 34−36
St. 56

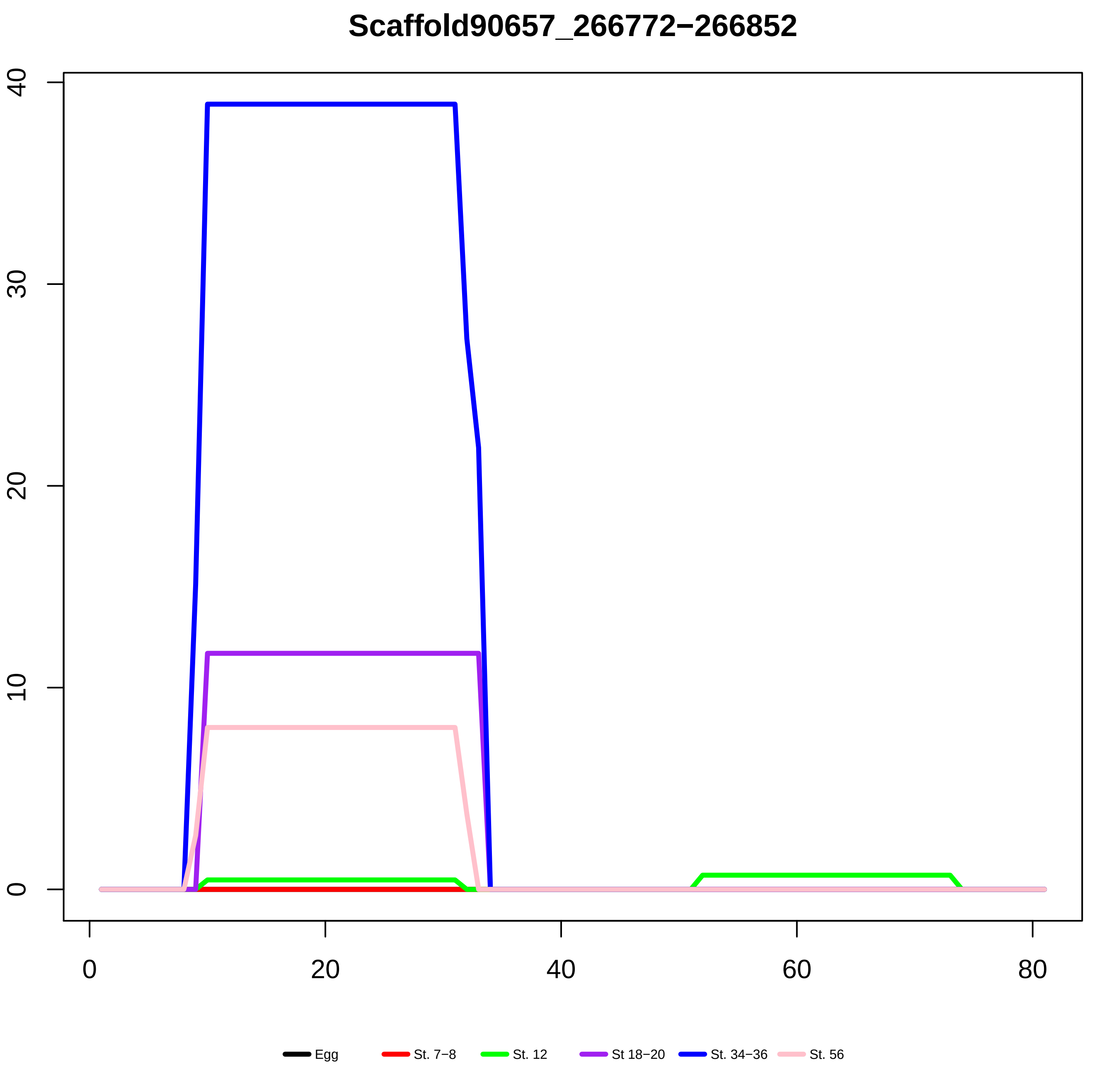
Scaffold90657_266772−266852
0
10
20
30
40
0
20
40
60
80
Egg
St. 7−8
St. 12
St 18−20
St. 34−36
St. 56

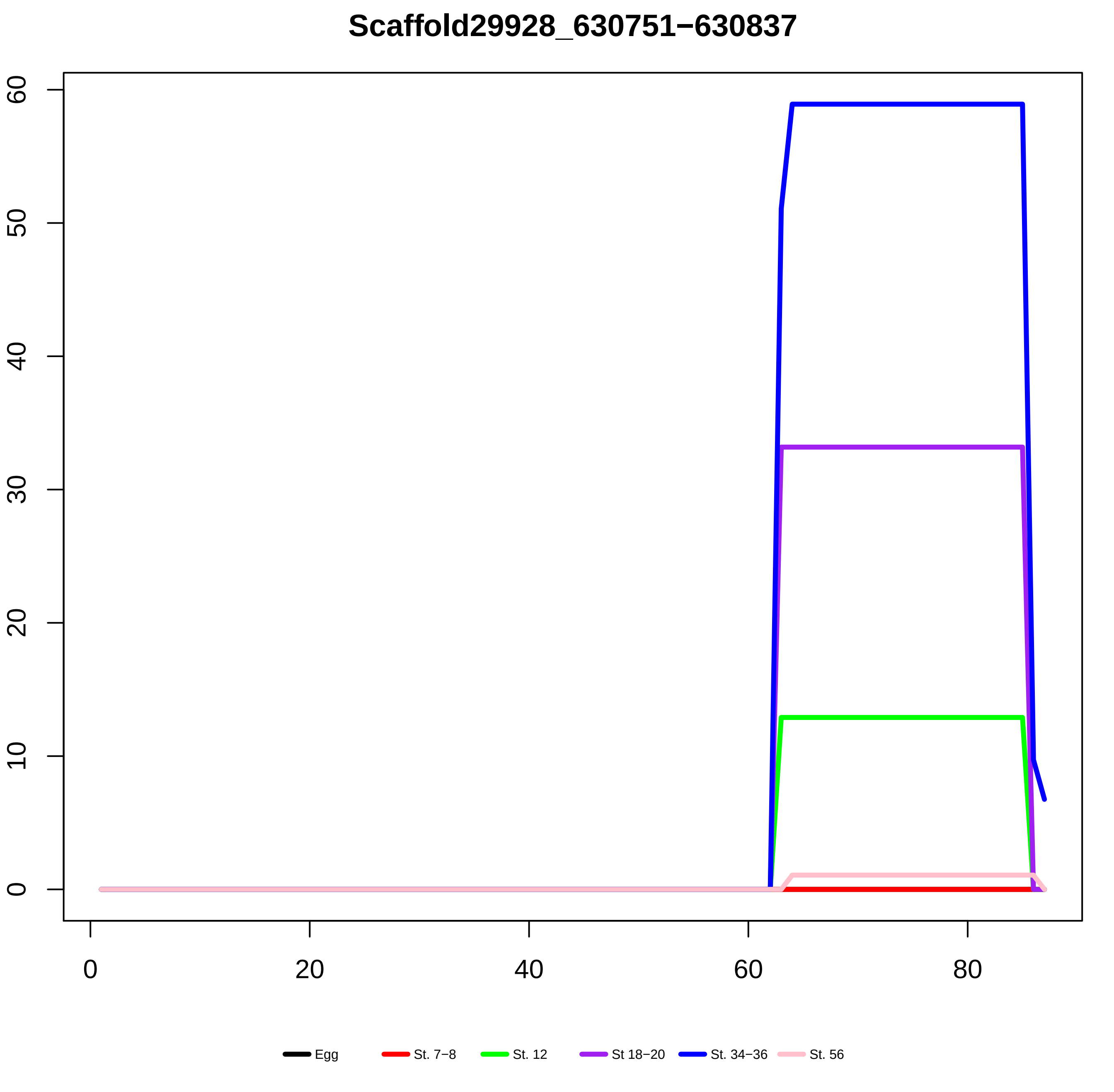
Scaffold29928_630751−630837
0
10
20
30
40
50
60
0
20
40
60
80
Egg
St. 7−8
St. 12
St 18−20
St. 34−36
St. 56

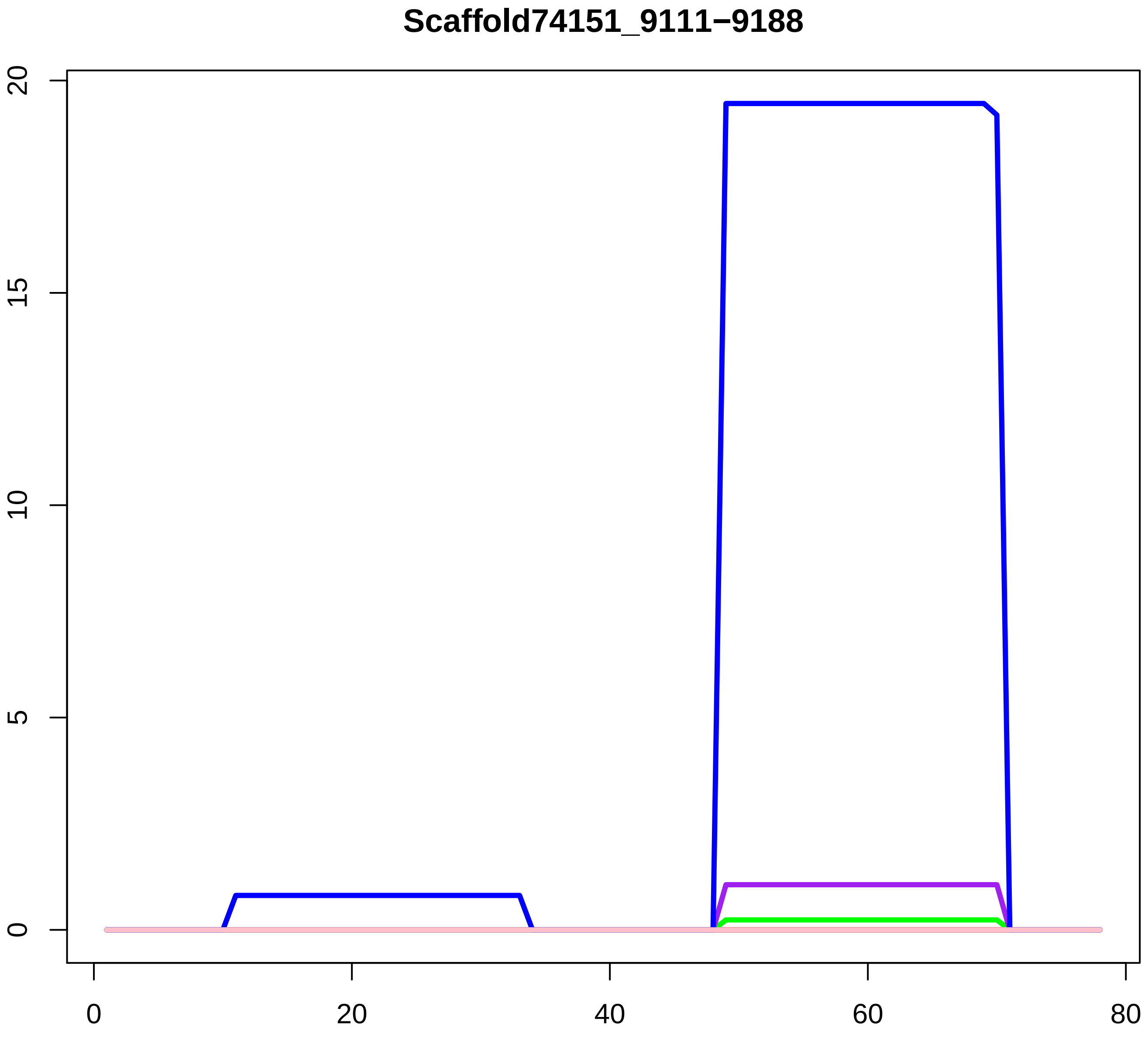

Scaffold74151_9111−9188
0
5
10
15
20
0
20
40
60
80
